# Predicting multiplex subcellular localization of proteins using protein-protein interaction network: a comparative study

**DOI:** 10.1186/1471-2105-13-S10-S20

**Published:** 2012-06-25

**Authors:** Jonathan Q Jiang, Maoying Wu

**Affiliations:** 1 School of Life Science and Biotechnology, Shanghai Jiao Tong University, Shanghai 200240, P.R.China; 2 Department of Computer Science, City University of Hong Kong, 83 Tat Chee Avenue, Kowloon, Hong Kong

## Abstract

**Background:**

Proteins that interact in vivo tend to reside within the same or "adjacent" subcellular compartments. This observation provides opportunities to reveal protein subcellular localization in the context of the protein-protein interaction (PPI) network. However, so far, only a few efforts based on heuristic rules have been made in this regard.

**Results:**

We systematically and quantitatively validate the hypothesis that proteins physically interacting with each other probably share at least one common subcellular localization. With the result, for the first time, four graph-based semi-supervised learning algorithms, Majority, *χ*^2^-score, GenMultiCut and FunFlow originally proposed for protein function prediction, are introduced to assign "multiplex localization" to proteins. We analyze these approaches by performing a large-scale cross validation on a *Saccharomyces cerevisiae *proteome compiled from BioGRID and comparing their predictions for 22 protein subcellular localizations. Furthermore, we build an ensemble classifier to associate 529 unlabeled and 137 ambiguously-annotated proteins with subcellular localizations, most of which have been verified in the previous experimental studies.

**Conclusions:**

Physical interaction of proteins has actually provided an essential clue for their co-localization. Compared to the local approaches, the global algorithms consistently achieve a superior performance.

## Background

Most of the eukaryotic biological processes are carried out by the proteins in a specific compartment or organelle within the cell. Hence, the knowledge of subcellular localizations for an uncharacterized protein provides an insight into the understanding of its function, and thus a guideline for further investigations. With the advent of the high-throughput techniques, the number of newly identified proteins has been increasing explosively. However, although some experimental technologies [1,2] have been developed to identify the subcellular localizations of the proteins, the laboratory techniques to annotate the proteins still fall far behind the rapid accumulation of the protein sequences. As a result, a variety of computational methods have been ongoing proposed, most of which rely on an individual protein's characteristics, e.g. amino acid composition [3-6], physio-chemical properties [5,6], structures [6], and some other character signals [7-9].

Recent studies found that protein interactions in human [10,11], fruitfly [12] and yeast [1,13], are closely related to the localization of proteins. In other words, to interact with each other, proteins necessarily share a common subcellular localization, or an interface between two physically adjacent compartments, at least transiently or conditionally. Specifically, 76% of interactions occurred between proteins located in the same subcellular localizations in a yeast PPI dataset [13], while 52% interactions involved in co-localized proteins were found in human PPI networks derived from public databases and literature curation [14]. Hence, the large amount of proteomic data found in previous research become another important resource for protein subcellular localization prediction. To the best of our knowledge, no systematic study has been implemented towards this direction except for a few seminal investigations based on simple heuristic rules [13,15,16].

Another drawback of the previous approaches [3-5,7-9] is that they focused on the "mono-localization" case in which a given protein is assumed to reside in only one subcellular localization and thus all the multiple-localization proteins were ruled out from the studies. But the truth is that proteins may often simultaneously exist in, or migrate between two or more different subcellular compartments. For example, in the Yeast GFP Fusion Localization database [17], of the 3999 yeast proteins with known localizations, 1247 (31.2%) bear the feature of "multiplex localization". Taken together, all the reasons motive us to carry out a systematic study for associating proteins with multiple localizations based on the PPI network. From a machine learning point of view, classifying nodes in a partially labeled network can be viewed as a graph-based semi-supervised learning problem [18] in which the key idea is to exploit both labeled and unlabeled data by leveraging the relationships provided by the edges. As a proof-of-concept, we introduce four methods as well as their variants, which were originally proposed for inferring protein functions from the PPI networks. Typically, these computational methods basically utilize the "guilt-by-association" principle, which transfers annotations among neighbor nodes in the PPI network, assuming that nodes that are located close to each other tend to share the same functional categories [19]. Two of them, Majority [13] and *χ*^2^-score [20], belong to the *neighborhood counting *method that relied on the local structure of the given PPI network. By contrast, GeneMultiCut (GMC) [21] took the full structure into account by utilizing cut-based methodology so as to minimize the number of times that different annotations are associated with neighboring proteins. In addition, another algorithm, called FunFlow [22], considered both local and global e ects through simulating the spread over time of "functional flow" through the network. See Methods section for more details. Technically, these methods can be applied to predicting subcellular localization of proteins.

The underlying rationale of our study is that physical interactions among proteins may act as an important hint for co-localization. This hypothesis has not yet been verified systematically and quantitatively except for several preliminary intuitive validations, either experimental [1,10-13] or computational [15,23]. To make our study self-consistent, we compile a *Saccharomyces cerevisiae *PPI network, consisting of 3179 proteins with 12413 interactions, from BioGRID database (version 3.1.73, released 25-Jan-2011) [24] and use the reliability [22] as a metric to quantitatively verify this hypothesis. Our results indicate that a pair of proteins physically interacting with each other is much more likely to share a common subcellular localization than two "randomly chosen" proteins. With these results, we systematically analyze the four aforementioned algorithms by performing a large-scale cross validation on this PPI network and comparing their predictions for 22 protein subcellular localizations. The global methods, GenMultiCut and FunFlow always achieve a superior performance than the local counterparts except for two localizations involving protein transport and secretion, i.e., "ER to Gogi" and "lipid particle". In addition, we find that none of methods assign proteins to "bud". Therefore, we design case studies for these localizations, and discover that such predictions are consistent very well with the neighborhood topologies of the proteins which were experimentally annotated with these specific localizations. Furthermore, we build an ensemble classifier based on these four approaches and annotate 529 unlabeled and 137 ambiguous annotated proteins with multiplex subcellular localizations. Fortunately, most of these assignments have been previously characterized in UniProt (release 2011-03) [25] and SGD [26] database.

## Results and discussion

### Yeast PPI network and protein subcellular localizations

The yeast PPI network contains 3179 vertices corresponding to unique proteins, and 12413 edges corresponding to the unique interactions (see Additional File 1 for the full list). The Yeast GFP Fusion Localization database collected 6234 budding yeast proteins which are experimentally classified into 22 distinct subcellular localizations. After filtering out those not in the previous PPI network, 529 proteins are of no subcellular localization annotation, and 137 proteins are annotated with ambiguous localizations (see Additional File 2 for the statistics). We call these 666 proteins as "uncharacterized", which we need to predict in the subsequent sections. The subcellular localization information of the 2513 annotated proteins are given in Table 1 where 1719 (68.79%) proteins were assigned to exactly one subcellular localization, 739 (29.57%) to two, and 55 (2.2%) to at least three.

**Table 1:** The classification of 2513 annotated proteins into 22 subcellular localizations.

order	subcellular localization	number of proteins
1	Actin	30
2	Bud	12
3	Bud neck	51
4	Cell periphery	59
5	Cytoplasm	1195
6	Early Golgi	40
7	Endosome	39
8	Endoplasmic reticulum (ER)	125
9	ER to Golgi	6
10	Golgi	29
11	Late Golgi	36
12	Lipid particle	9
13	Microtubule	15
14	Mitochondrion	206
15	Nuclear periphery	51
16	Nucleolus	145
17	Nucleus	1071
18	Peroxisome	18
19	Punctate composite	96
20	Spindle pole	57
21	Vacuolar membrane	31
22	Vacuole	48

### Physical interaction implies co-localization

Our study is built upon the assumption that proteins physically interacting with each other are likely to share a common subcellular localization. To verify this hypothesis systematically and quantitatively, we split the protein interaction data set into 28 groups according to different experiment systems and throughput levels (Methods). For each group, we count the fraction of interaction pairs that share at least one subcellular localization and more than two localizations, respectively. The former is used to denote the reliability of such interactions (see Methods). The results are summarized in Table 2. From Table 2, we can clearly see that for each group of experiments, the reliability is around 0.4 − 0.6. This discovery is consistent well with the previous studies [13,15].By contrast, the number of interaction pairs that share more than two common localizations dramatically dropped to, say, about 5% for all the experiments (Table 2). This phenomenon can be explained as follows: Proteins found in more than two subcellular localizations often exist at or migrate between these compartments involved in various biological processes at different time points or under distinct environments; PPI network, however, can only capture the instantaneous interactions among proteins. We also calculate the Pearson's correlation coefficient (PCC) between the overlap of the interacting protein pair and the overlap of their functions. Results show that the overall correlation is weak (PCC = 0.09), but very significant (*p *= 1.17 × 10^−15 ^in Fisher's exact test). As a result, we can simply conclude that physical interaction is indeed an important hint for co-localization of proteins.

**Table 2:** Protein co-localization for 28 experiment sources in the BioGRID database.

Experiment system	Throughput technique	number of interactions	number of common l localizations	
			
			≥ 1 (reliability)	≥ 2
Affinity Capture-Luminescence	low throughput	29	0.3448	0
Affnity Capture-MS	high throughput	44399	0.5343	0.0798
	low throughput	5627	0.5873	0.0945
Affnity Capture-RNA	high throughput	3657	0.2625	0.0014
	low throughput	86	0.3140	0.0581
Affinity Capture-Western	high throughput	213	0.6526	0.0798
	low throughput	11257	0.5477	0.0759
Biochemical Activity	high throughput	4211	0.3363	0.0686
	low throughout	3427	0.4471	0.1100
Co-crystal Structure	low throughput	364	0.6593	0.1703
Co-fractionation	high throughput	102	0.0098	0
	low throughput	585	0.4821	0.0598
Co-localization	low throughput	448	0.5357	0.0588
Co-purification	high throughput	11	0.8182	0.5455
	low throughput	1667	0.6155	0.0834
FRET	high throughput	13	0.1538	0
	low throughput	121	0.6364	0.0579
Far Western	low throughput	74	0.5811	0.0541
	high throughput	4738	0.4185	0.0319
PCA	low throughput	409	0.5232	0.0098
	high throughput	9	0.3140	0
Protein-RNA	low throughput	168	0.1116	0.0129
	high throughput	328	0.3333	0
Protein-peptide	low throughput	233	0.4940	0.0833
	high throughput	27	0.5556	0.0370
Reconstructed Complex	low throughput	3347	0.5088	0.0986
	high throughput	6624	0.3578	0.0773
Two-hybrid	low throughput	4622	0.4799	0.1019

### Large-scale cross validation

We compare four graph-based semi-supervised learning algorithms (1) Majority [13], (2) *χ*^2^-score [20], (3) GenMultiCut (GMC) [21] and (4) Functional flow (FunFlow) [22] as well as their variants by performing 5-fold cross validation on the obtained PPI network (see Method). The overall evaluation mean average precision (MAP) of the cross validation are shown in Table 3. From the table, we have the following observations. The global methods, GenMultiCut and FunFlow consistently, sometimes significantly, outperforms the local counterparts, Majority and *χ*^2^-score. In particular, MAP increased about 30% in all the "PPI-only" and "PPI-weight" cases. Consistent with previous work [22], MAP are improved 0.6%, 0.4% and 0.1% for Majority, GMC and FunFlow approaches on the "PPI-weight" scenario. This indicates that edge weights of the PPI network have a crucial influence on the prediction results even if the improvements in our study seem rather slightly. This is possibly because that the interactions used here are required to be supported by at least two publications. Hence, the networks exploited in "PPI-only" and "PPI-weight" experiments does not deviate so significantly from those studies in [22].

**Table 3:** MAP of 5-fold cross validation for four graph-based semi-supervised learning algorithms.

Algorithms	MAP (%)	
	PPI-only	PPI-weight
Majority	42.13	42.39
Merged	32.53	32.53
Common	24.36	24.36
*χ*^2 ^−1	33.07	
*χ*^2 ^−2	19.77	
*χ*^2 ^−3	14.59	
GMC	53.43	53.66
FunFlow	62.07	62.16

The *χ*^2^-score method can be only applied to "PPI-only" case. The GenMultiCut method were performed through ILP as suggested by [22].

**Figure 1:**
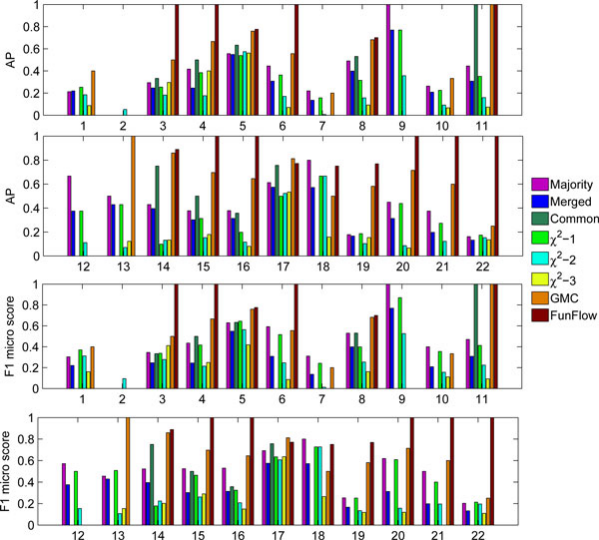
**Average precision and F1 micro score for each subcellular localization in the "PPI-only" scenario**. Different colour bars correspond to the results obtained by different algorithms. The first row is the average precision for the first 11 subcellular localizations; the second one is the average precision for the last 11 subcellular localizations. The similar interpenetrations are used in the third and four rows for F1 micro score.

We further check the average precision (AP) and F1 micro score for each subcellular localization on both experiments. In the "PPI-only" case (Figure 1), all these methods achieve a competitive performance for two subcellular localizations "cytoplasm" and "Nucleus" with which a large number of proteins are experimentally annotated with. For another 11 localizations, i.e., "Bud neck", "cell periphery", "Early Golgi", "Late Golgi", "Microtubule", "Mitochondrion", "Nuclear periphery", "Punctate composite", "Spindle pole", "Vacuolar membrane" and "Vacuole", two global methods always, sometimes significantly, outperform two local approaches. Specially, the performances obtained by FunFlow method are improved significantly, say, about 50% for localization "Bud neck" and about 70% for localization "Vacuole", respectively. However, this method failed to associate proteins with four localizations, "Actin", "Endosome", "Golgi" and "Microtubule", for which, GMC achieve competitive performance with or outperform these two local methods. The superior performance of global methods is expected owing to the fact that the GMC algorithm takes the full structure of the PPI network into account, and FunFlow considers both the global and local effects. The reason for the failure of FunFlow method on four localizations can be explained as follow. The GMC algorithm was implemented here through an ILP as suggested by [22], and hence the solution is {0, 1} vector for each localization, which means that a given protein should be either assigned to this localization or not. By contrast, the FunFlow method substantially belongs to the rank-based classifier and thus the cutting point for positive/negative predictions depends on the corresponding threshold. According to the description in [22], we choose 0 as the threshold, which is similar to SVM where we use *f *(*x*) = 0 as the decision boundary. However, it is not necessarily the best choice for some localization, for example, the four localizations where the failure occurred. How to select an appropriate threshold to obtain a better performance is still a key open question in rank-based multi-label learning [27], which is left for our future study. Moreover, we are surprised to find that two local methods as well as their variants achieved better performance for two localizations, "ER to Golgi" and "lipid particle" which are involved in protein transport and secretion. Finally, it is astonishing that almost all the methods fail to recall the "Bud" localization for proteins, except for the *χ*^2 ^− 2 algorithm with a very low AP value. We design case studies to further analyze these two unexpected phenomena in the following section.

**Figure 2:**
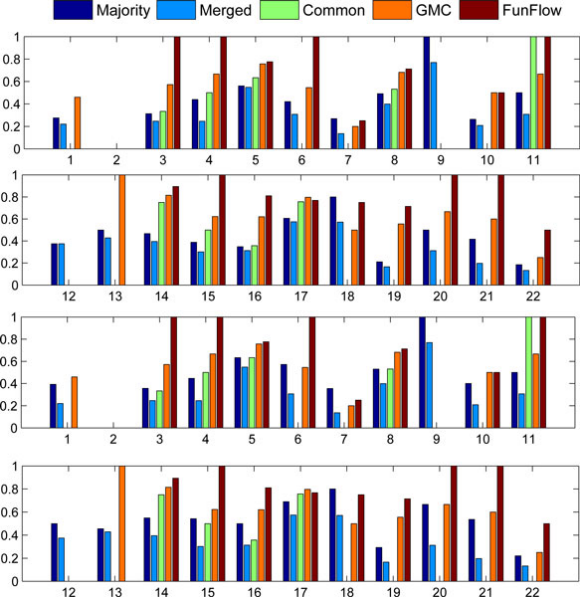
**Average precision and F1 micro score for each subcellular localization in the "PPI-weight" scenario**. Different colour bars correspond to the results obtained by different algorithms. The first row is the average precision for the first 11 subcellular localizations; the second one is the average precision for the last 11 subcellular localizations. The similar interpenetrations are used in the third and four rows for F1 micro score.

Similar results were observed in the "PPI-weight" scenario (Figure 2). All the methods achieved good enough performances for two localizations, "Cytoplasm" and "Nucleus". The global methods always outperform the local counterparts on the 11 localizations, "Bud neck", "cell periphery", "Early Golgi", "Late Golgi", "Microtubule", "Mitochondrion", "Nuclear periphery", "Punctate composite", "Spindle pole", "Vacuolar membrane" and "Vacuole". As a benefit of weighting edges of PPI network, FunFlow can successfully recall the two localizations, "Endosome" and "Golgi", which are a failure in the "PPI-only" case. But it still su ers from the failure of associating proteins with two other localizations, "actin" and "Microtubule". Similar to the "PPI-only" case, local methods show their superiority for two localizations, "ER to Golgi" and "Lipid particle". Unfortunately, all of the methods fail to hit the "Bud".

### Case study: "ER to Golgi" and "Lipid particle" location

**Figure 3:**
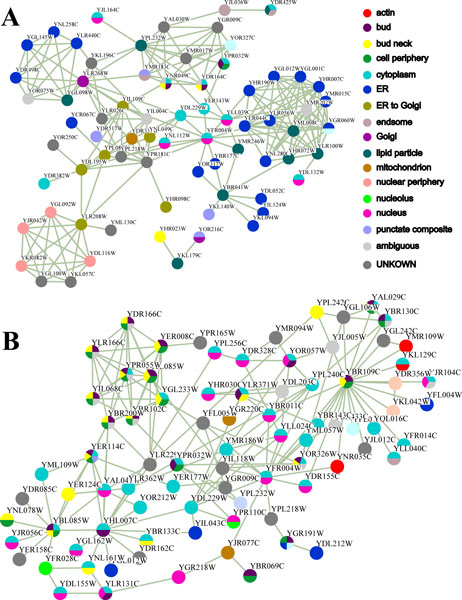
**Subgraphs of the PPI network in our case studies**. (a) the subgraph consists of 72 proteins annotated with localizations "ER to Golgi" and "Lipid particle" as well as their immediate neighbors, and 204 interactions between these proteins. (b) the subgraph consists of 83 proteins annotated with localizations "Bud" as well as their immediate neighbors, and 164 interactions between these proteins.

According to the cross validation results, an interesting question might be naturally raised. Is the superiority of these local methods for two subcellular locations "ER to Golgi" and "Lipid particle" caused by the algorithms themselves or the immediate neighborhood structure of proteins experimentally annotated with the two specific locations? Here, we design a case study to explore the reasonable explanation. We extracted the proteins annotated with locations "ER to Golgi" and "Lipid particle" as well as their immediate neighbors and the physical interactions among them (Additional File 3) from our network. The subnetwork, containing 72 unique proteins and 204 unique interactions, is illustrated in Figure 3A. Clearly, although the 6 proteins that were experimentally annotated with "ER to Golgi" location are linked with each other, they do not form a densely connected community. Instead, they scatter in the subnetwork to bridge two protein cliques that localized in "endoplasmic reticulum" and "Nuclear periphery", which is in accordance with the functions of endoplasmic reticulum and Golgi apparatus. As we all know, amino acids dehydrate to form the peptide in the ribosome attached to the endoplasmic reticulum where the peptide correctly coils and folds with the help of endoplasmic reticulum molecular chaperons. After that, the peptide is transported into Golgi apparatus to be converted into the specific proteins via chemical modification (e.g., Golgi glycosylation, etc.) and then these proteins are further transported to different organelles, such as mitochondrion, or cytomembrane through secretory granule [28]. Therefore, the proteins labeled with "ER to Golgi" are almost secretory proteins and often physically interact with other proteins that localized in "ER", "Golgi" and "Nuclear periphery" (Figure 3A). For example, the protein YLR208W is the component of both the Nup84 nuclear pore sub-complex and the Sec13p-Sec31p complex of the COPII vesicle coat, required for vesicle formation in ER to Golgi transport and nuclear pore complex organization [26]. 4 "Nuclear periphery" proteins and 2 "Unknown" proteins are joined together with it in a tightly-knit fashion (the lower left corner of Figure 3A). Obviously, it will receive more label information from 4 "Nuclear periphery" negative samples than that from 2 "ER to Golgi" positive samples if the global methods are applied. By contrast, if we adopt the local method, the "ER to Golgi" location is one of the two subcellular locations that frequently appear among its neighbors. This subcellular localization, "Lipid particle", has been defined in Gene Ontology [29] as term GO:0005811 with the description that any particle of coalesced lipids in the cytoplasm of a cell and may include associated proteins. As illustrated in Figure 3A, proteins labeled with this localization can usually interact with proteins that localized in smooth endoplasmic reticulum (SER) whose functions include synthesis of steroids and lipids. For instance, the protein YML008C are densely linked to 9 proteins in "ER" and 2 proteins in "Lipid particle" to constitute a clique involved in ergosterol biosynthesis [26]. Hence, it is easily misclassified into "ER" localization if the full structure is taken into account. Contrarily, such localization can be successfully recovered by the local methods since they consider up to three common localizations that the neighbors of a given protein share. Another interesting example is the protein YBR041W, a long chain fatty acid synthetase and transporter. In Figure 3A, it acts as a hub in the subnetwork consisting of 5 "ER" proteins, 2 "Lipid particle" proteins and 1 "punctate composite" protein involved in lipid metabolism and phosphatidic acid biosynthesis [26]. Thus, "ER" localization is far more preferable to "Lipid particle" if the global methods were adopted. From the above analysis, we assert that the superiority of the local algorithms for these two localizations is totally due to the neighborhood topology of these proteins annotated with corresponding localizations.

### Case study: "Bud" location

We are astonished to find that none of these methods can recover the "Bud" location for proteins. To explore the reason, we extracted the subnetwork that comprises the interactions of the proteins labeled with "Bud" location and its immediate neighbors. This subnetwork contains 83 proteins and 164 interactions (Additional File 4). From Figure 3B, we see clearly that three "Bud" proteins YBR109C, YBL085W and YHL007C play a role of hub in the subnetwork and physically interact with a large number of proteins annotated with other locations. Therefore, neither local methods nor global algorithms can successfully assign these proteins to "Bud" location. Although 7 proteins labeled with "Bud" location, YBR200W, YPR102C, YIL068C, YPR055W, YJL085W, YLR166C, YER008C and YDR166C are densely jointed together to form an exocyst complex [26](the upper left corner of Figure 3B). Unfortunately, there are 6 out of them which were experimentally labeled with "ambiguous" in Yeast GFP Fusion Localization database [17] and thus treated as uncharacterized proteins in our study (Methods). In this way, there is so few positive samples for "Bud" location, i.e., the data sparsity problem [18] occurred in the 5-fold cross validation. This is possibly the primary reason why none of these algorithms can associate proteins with "Bud" location.

### Assign subcellular localizations to uncharacterized proteins

There are still 529 unlabeled proteins and 137 proteins labeled with ambiguous localization in our PPI network. Considering that the local methods and global methods have their own particular advantages and disadvantages, we build an ensemble classifier to assign subcellular localizations to these 666 uncharacterized proteins (Methods). According to their annotation situations in [17], the 137 ambiguous localized proteins could be divided into two groups: (1) 60 proteins labeled with localizations besides "ambiguous", and (2) 77 proteins labeled with "ambiguous" only. Our predictions for the first group are listed in Additional File 5 where we split them into four different types, i.e., 9 *Correct *(15%), 21 *Partial Correct *(35%), 18 *Mismatch *(30%) and 12 *Unknown *(20%). In the *Correct *case, for a given protein, our predictions are strictly the same as its another experimentally observed localizations [17]. By contrast, in the Partial Correct case, our predictions and the experimental observation share at least one but not total localizations. *Mismatch *case means that our predictions cannot be found in the experimental observation, while *Unknown *case denotes that a recall failure of the ensemble classifier occurred (Additional File 5). We only give 5 predictions for each type in Table 4. From the table, we can clearly see that most of our assignments are supported by the records in UniProt [25] and SGD database [26]. It is worth noting that these four types correspond to different situation of the match between our predictions and experimental observation for each protein. Hence, they do not mean that such predictions are right or wrong. For example, protein YDR181C in the *Correct *case, were labeled with "Nucleus" localization in UniProt and SGD database which conflicts with our prediction and experimental observation. On the contrary, our predictions of two proteins YDL146W and YDR309C in the *Mismatch *case, "actin", could be found in their annotations in SGD database (Table 4).

**Table 4:** Top 5 predictions of each type for the first group of 60 "ambiguous" annotated proteins.

Type	Protein (ORF)	Annotation	Prediction	UniProt	SGD
Correct	YBL034C	ambiguous spindle pole	spindle pole	Nucleus. Spindle. cytoskeleton. kinetochore.	spindle pole body (IDA)
	
	YDR181C	ambiguous; cytoplasm;	cytoplasm;	Nucleus	nuclear chromatin (IDA) nuclear chromosome, telomeric region (IC)
	
	YGR020C	ambiguous; vacuolar membrane	vacuolar membrane		fungal-type vacuole membrane (TAS) vacuolar proton-transporting V-type ATPase, V1 domain (TAS)
	
	YHR119W	ambiguous; nucleus	nucleus	Nucleus (Probable). Chromosome (Probable).	Set1C/COMPASS complex (IPI)
	
	YHR183W	ambiguous; cytoplasm	cytoplasm	Cytoplasm	cytoplasm (IDA) mitochondrion (IDA)

Partial Correct	YAL029C	ambiguous; cell periphery; bud neck; cytoplasm;bud	Bud	Bud	cellular bud (IDA) cellular bud tip (IDA) filamentous actin (IDA) mitochondrion (IDA)
	
	YBR102C	ambiguous; cell periphery; bud neck;bud	cytoplasm; bud	secretory vesicle. Bud. Bud neck.	cellular bud neck (IDA) cellular bud tip (IDA)
	
	YBR130C	ambiguous; cell periphery; cytoplasm;bud	bud		actin cap (TAS) cellular bud tip (IDA) cytoplasm (IDA)
	
	YBR260C	ambiguous; cytoplasm; bud;bud neck;	cytoplasm	Cytoplasm.	actin cortical patch (IDA) cellular bud (IDA) mating projection tip (IDA)
	
	YFR016C	ambiguous; cytoplasm;bud	cytoplasm		cellular bud (IDA) cytoplasm (IDA)

Mismatch	YAR019C	ambiguous; spindle pole	cytoplasm		cellular bud neck (TAS) spindle pole body (IDA)
	
	YBL105C	ambiguous; cytoplasm; bud neck;bud	actin		cytoplasm (IDA) cytoskeleton (IDA) nucleus (IDA)
	
	YDL146W	ambiguous; cell periphery; cytoplasm; bud neck;bud	actin	Bud. Cytoplasm Bud neck	colocalizes with actin cortical patch (IDA) cellular bud (IDA) cytoplasm (IDA) cellular bud neck (IDA)
	
	YDR309C	ambiguous; cytoplasm; bud	actin	Bud neck (By similarity). Bud tip (By similarity). cell cortex (By similarity) cytoskeleton (By similarity).	actin cap (TAS) cellular bud tip (IDA) incipient cellular bud site (IDA) mating projection tip (IDA) plasma membrane (IGI)
	
	YHR158C	ambiguous; cell periphery; bud neck; bud	cytoplasm; nucleus		cellular bud neck (IDA) cellular bud tip (IDA) mating projection tip (IDA) cytoplasm (IDA)

	YCL024W	ambiguous; cell periphery; bud neck;bud		Bud neck	cellular bud neck (IDA)
	
					cellular bud neck septin collar (IDA) incipient cellular bud site (IDA)
	YDL089W	ambiguous; nuclear periphery		Membrane	nuclear periphery (IDA)
	
Unknown	YDR069C	ambiguous; endosome		Cytoplasm. Late endosome membrane;	endosome (IDA) membrane fraction (IDA) proteasome complex (IPI) mitochondrion (IDA)
	
	YDR507C	ambiguous;bud bud neck;cytoplasm		Cytoplasm. Bud neck.	cellular bud neck (IDA)
	
	YHL019C	ambiguous; late Golgi		coated pit.	AP-1 adaptor complex (IPI)

In this table, "Annotation" denotes the experimentally observed subcellular localizations in Yeast GFP Fusion Localization Database [17]. "UniProt" means the subcellular localization in general annotation (comments) in UniProt Database [25]. "SGD" means the cellular component of GO annotation in SGD database [26]. Each type corresponds to different situation of the match between our prediction and the experiment validation.

We incorporate the second group into 529 unlabeled proteins since they are all lack of prior knowledge. The total predictions for these 606 proteins are given in Additional File 6 where we also split them into four different types, *Correct*, *Partial Correct*, *Mismatch *and *Unknown*. These types correspond to different situation of our predictions supported by annotations in UniProt and SGD database. That is, for a given protein, *Correct *case means that every predicted localization was previously characterized in these two database; in the *Partial Correct *case, at least one of but not all of the predicted localizations could be found in these two database; *Mismatch *case denotes that none of our predicted localizations was supported by these two database currently; in the *Unknown *case, the given protein has not been characterized in these two database or a failure of our ensemble classifier occurred. Similarly, we give 5 predictions for each type in Table 5. Once again, most of the predictions are supported by the localization annotation in Uniprot and SGD database.

**Table 5:** Top 5 predictions of each type for the 606 proteins without prior knowledge.

Type	Protein(ORF)	Prediction	UniProt	SGD
Correct	Q0045	mitochondrion	Mitochondrion inner membrane.	mitochondrion (IDA)
	
	Q0080	mitochondrion	Mitochondrion membrane.	mitochondrion (IDA)
	
	YAL020C	cytoplasm		cytoplasm (IDA, IPI)
	
	YAL029C	bud	Bud.	cellular bud (IDA) cellular bud tip (IDA)
	
	YBL041W	cytoplasm; nucleus	Cytoplasm. Nucleus.	endoplasmic reticulum membrane (IC) nucleus (IC)

Partial Correct	YAL042W	ER	Endoplasmic reticulum membrane; Golgi apparatus membrane	ER to Golgi transport vesicle (IDA) integral to endoplasmic reticulum membrane (IDA) integral to Golgi membrane (IDA)
	
	YBL088C	cytoplasm; nucleus	Nucleus. telomere	nucleus (IC) mitochondrion (IDA)
	
	YBR020W	cytoplasm; nucleus		cytoplasm (IGI)
	
	YBR072W	cytoplasm		cytoplasm (IDA) nucleus (IDA)
	
	YBR108W	actin; cytoplasm	Membrane raft; Peripheral membrane protein	actin cortical patch (IDA) colocalizes-with membrane raft (IDA)

Mismatch	YAL003W	cytoplasm		ribosome (TAS)
	
	YAL028W	cytoplasm; nucleus	Endoplasmic reticulum membrane	endoplasmic reticulum (IDA)
	
	YAL030W	lipid particle	Endomembrane system	cellular bud neck (IDA) endosome (IDA) plasma membrane (IDA) trans-Golgi network (IDA) transport vesicle (IDA)
	
	YAL040C	cytoplasm		nucleus (IDA, IMP)
	
	YAL062W	actin; cytoplasm		nucleus (IDA) mitochondrion (IDA)

Unknown	Q0120		Mitochondrion.	mitochondrion (IDA)
	
	YAL034C	nucleus		
	
	YAR018C	spindle pole		
	
	YAR027W		Nucleus membrane; Cell membrane	nuclear envelope (IDA)
	
	YAR042W		Cytoplasm Golgi apparatus membrane Nucleus outer membrane	early endosome (IDA) endoplasmic reticulum (IDA) Golgi trans cisterna (IDA) nuclear envelope (IDA)

In this table, "UniProt" means the subcellular localization in general annotation (comments) in UniProt Database [25]. "SGD" means the cellular component of GO annotation in SGD database [26]. Each type corresponds to different situation of the match between our prediction and the experiment validation.

There are 46 proteins annotated with more than one subcellular localization sites in the first group. To compare the performance of the ensemble classifier with the 4 basic classifiers, we summarized these predictions in Additional File 7. Table 6 lists 5 examples of proteins and their associated localizations predicted by different methods. We clearly see that the localizations of these 5 proteins identified by the ensemble classifier are almost the same as the annotations of these proteins in the yeast GFP Fusion Localization database. By contrast, the 4 basic classifiers can only predict some of the labels.

**Table 6:** Annotation results of 5 proteins in yeast GFP Fusion Localization database by the ensemble classifier and 4 basic classifiers.

Protein	Annotation	Majority	*χ*^2 ^score	GMC	FunFlow	Ensemble
YAL029C	cell periphery;bud neck; cytoplasm;bud	bud neck; cytoplasm;nucleus	cell periphery; bud neck;bud	nucleus	cytoplasm	bud neck;cytoplasm; nucleus;bud

YBR130C	cell periphery; cytoplasm;bud	cell periphery; cytoplasm;nucleus	cell periphery; bud neck;bud		cytoplasm	cell periphery; cytoplasm;bud

YBR260C	bud neck; cytoplasm;bud	bud neck; cytoplasm	mitochondrion; bud neck;nucleus	cytoplasm		bud neck; cytoplasm

YDR181C	cytoplasm; nucleus	cytoplasm; nucleus	mitochondrion; nucleolus;nucleus	cytoplasm;nucleus	nucleus	cytoplasm; nucleus

YNL298W	cell periphery; cytoplasm;bud	cell periphery; cytoplasm;nucleus	cell periphery; bud neck;cytoplasm	cytoplasm		cell periphery; cytoplasm

Our method can predict all the labels for the proteins, while other approaches can only recover part of the labels.

## Conclusions

Traditionally, individual proteins' physical, chemical and biological characteristics were the major features used for subcellular localization prediction. Different from this idea, we exploit another important resource, i.e., protein-protein interaction network, to address this problem. Our start point is the recent studies that observed the protein interactions in many species are related to the subcellular localization of proteins. The main contribution of this paper is the application of this concept to compare several the state-of-the-art algorithms and their uses as building block of an ensemble classifier.

Firstly, we systematically and quantitatively validate the hypothesis that proteins physically interacting with each other probably share a common subcellular localization. After that, for the first time, four graph-based semi-supervised learning algorithms, Major, *χ*^2^-score, GenMultiCut and FunFlow originally proposed for function assignment, are introduced to associate "multiplex localization" to proteins. In a large-scale cross validation test on a yeast proteome complied from BioGRID database, we show that, compared to local methods, the global approaches consistently, sometimes significantly improve the predictive performance over the 22 protein subcellular localizations, except for two locations, "ER to Golgi" and "lipid particle".

Considering that there are both advantages and disadvantages of each method, we build an ensemble classifier to predict the subcellular localizations for 529 unlabeled and 137 "ambiguous" annotated proteins in the PPI network. Most of these predictions have been experimentally characterized in Uniprot and/or SGD database. The results further illustrate that physical interaction is indeed an important hint for co-localization of proteins.

## Methods

### Data source

The yeast protein interaction dataset were obtained from BioGRID database (version 3.1.73, released 25-Jan-2011) [24]. To reduce the noise and false positive, we used only those interactions that were determined by physical experiment and confirmed by at least two publications. The redundant and self-connecting interactions were excluded and the largest connected component of the resulted network is extracted for our studies. The laboratorially identified localizations of proteins were downloaded from the Yeast GFP Fusion Localization database [17].

### Weighting edges

It is well known that the weights of the edges has a profound influence on the results,even though the networks are based on the same underlying topology [22]. In the context of graph-based algorithms, it is possible to weigh edges by modeling the reliability for each interaction. For every physical interaction, the reliability is in turn based on the experimental sources that contribute to our knowledge about the existence of the interaction. To estimate the values, we follow the approach in [22]. That is, we separate the physical interaction data into 16 groups according to di erent experimental systems and further divide each group into two smaller ones if this experiment system can be implemented as high-throughput and low-throughput, respectively. Then, we allocate one group for the family of all specific experiments and totally obtained 28 groups. We assume that the reliability of different sources are independent, and thus conclude by estimating the reliability of an interaction to be the noisy or of the unreliability of the underlying data sources. Let *r_i _*be the reliability of experimental source *i*, i.e., the fraction of interaction pairs that are from experimental source *i *and share at least one common subcellular localization. For an interaction between a pair of proteins *u *and *v*, we compute the reliability of that interaction using

(1) $$r_{uv}=1-\underset{i\in E_{uv}}{\prod }{\left(1-r_{i}\right)}^{{n_{i}{}_{,\;}}_{uv}}$$

where *E_uv _*is the set of experimental sources in which interaction between *u *and *v *is observed, and *n_i,uv _*is the number of times which interaction between *u *and *v *is observed from experimental source *i*. This treats each *r_i _*as a probability and assumes independence; the product is taken over all experimental sources. We introduce two types of schemes for applying our algorithm. The first variant attempts to capture only qualitative functional links between proteins by PPI. In the second scheme, we weighted each edge by the above-mentioned procedure. In this paper, we call these variants as"PPI-only" and "PPI-weight" network, respectively.

### Graph-based semi-supervised learning algorithms

For a multiplex subcellular localization prediction problem, we have *K *subcellular localizations and a protein set $P={\left\{p_{u}\right\}}_{u=1,\ldots ,n}$. The first *l *proteins are labeled as {y_1_,..., y*_l_*} with *y_uk _*= 1 in case protein *u *is annotated with localization *k*. Our goal is to predict the labels {y*_l_*_+1_,..., y*_n_*} for the remaining unlabel proteins {*p_l_*_+1_,..., *p_n_*}. The PPI network of these proteins can be represented as a graph G=(V,ε,W), with nodes set V=L∪U where  corresponds to labeled proteins and  corresponds to uncharacterized proteins. The element *w_uv _*of the affinity matrix *W*∈ℜ*^n × n ^*indicates the reliability of edge between protein *u *and *v*.

Here, we introduce, analyze and compare four graph-based semi-supervised learning algorithms. Although they were originally proposed for inferring protein function from PPI networks, these methods can also be applied to our problem as far as the functions are replaced with different subcellular localizations. We briefly describe the four methods in terms of our problem.

#### Majority

It is the simplest and most straightforward algorithm that determines the subcellular localization of a protein based on the known localization of proteins lying in its immediate neighborhood. We consider all neighboring proteins and sum up the number of times each annotation occurs for each protein. As suggested by [13], we predict a given protein up to three subcellular localizations that are common among its neighbors. In the case of "PPI-weight", we simply extend the method by taking a weighted sum instead. For each protein, the score of a particular function is the corresponding sum. Two variants, Merged and Common have been proposed in [15] for comparison. In the Merged variant, for each protein, a subcellular localization is assigned based on the union of localization annotations for all its interaction partners. In contrast, for the Common variant method, when a protein interacts with more than one other protein only those subcellular localizations common to all its interaction partners are employed as a prediction.

#### χ ^2^-score

For each protein, we consider all other proteins within a radius *σ *as described in [20], and then for each subcellular location, we use a *χ*^2^-test to determine if it is over-represented. More precisely, for a protein *u*, each subcellular location *k *is assigned a score

(2) $$f_{uk}=\frac{{\left(n_{k}-e_{k}\right)}^{2}}{e_{k}}$$

where *n_k _*is the number of proteins in the *σ*-neighborhood of protein *u *that resides in the subcellular compartments *k *and *e_k _*is the expected number based on the overall frequency of subcellular location *k *within the network. Neighborhoods within radius *σ *= 1, 2, 3 are considered, referred to as *χ*^2 ^- 1, *χ*^2 ^- 2 and *χ*^2 ^- 3, respectively. However, this method can not extend naturally to the case of weighted interaction graphs.

#### GenMultiCut

The method utilize cut-based methodology so as to maximize the number of times the same annotations are associated with neighboring proteins [21]. Thus, it is global and takes the full structure of the network into account. Precisely, it tries to maximize

(3) $$\underset{\left(u,v\right)\in E^{\prime }}{\sum }\delta \left(f_{u},f_{v}\right)+\underset{u\in V}{\sum }h_{v}\left(f_{v}\right)$$

where *E*' is the set of edges incident on two unannotated proteins, *δ *is a function that equals to 1 if *x *= *y *and 0 otherwise, and *h_v_*(*k*) denotes the number of neighbors of *v *previously annotated with subcellular localization *k*. This optimization problem, which generalizes the NP-hard problem of minimum multiway cut [22], can be heuristically solved using simulated annealing for multiple runs [21]. To find a good approximation, Karaoz et al. [30] applied a local search procedure in which for every vertex in turn (until convergence), the state of the vertex is changed according to the majority of the states of its neighbors. In addition, they also consider the case where edges are weighted using gene expression profiles. An integer linear programming (ILP) reformulation of this problem suggested by [22] allows solving the problem in practice.

#### Functional flow

Nabieva et al. [22] proposed a graph-based algorithm that simulates functional flow between proteins. Proteins are initially assigned infinite potential for a subcellular localization if a given protein is annotated with the specific subcellular localization and 0 potential otherwise, i.e.,

(4) $$R_{0}^{k}\left(u\right)=\left\{\begin{array}{cc} \infty & \text{if}\;u\;\text{annotated}\;\text{with}\;k\\ 0 & \text{otherwise} \end{array}\right)$$

Labels are then simulated to flow from proteins with higher potential to their neighbors that have lower potential

(5) $$R_{t}^{k}\left(u\right)=R_{t-1}^{k}\left(u\right)+\underset{\left(u,v\right)\in E}{\sum }\left(g_{t}^{k}\left(v,\;u\right)-g_{t}^{k}\left(u,\;v\right)\right)$$

where $g_{t}^{k}\left(u,\;v\right)$ represent the flow of subcellular localization *k *at time *t *from protein *u *to protein *v*. subsequent time step, the amount of flow is influenced by the strength of the interactions between interaction partners and satisfies the capacity constraints

(6) $$g_{t}^{k}\left(u,\;v\right)=\left\{\begin{array}{cc} 0 & \text{if}\;R_{t-1}^{k}\left(u\right)<R_{t-1}^{k}\left(v\right)\\ \text{min}\;\left(w_{uv},\;\frac{w_{uv}}{\Sigma_{\left(u.z\right)\in E}w_{uz}}\right) & \text{otherwise} \end{array}\right)$$

The score for associating protein *u *with subcellular localization *k *over *d *iterations is calculated as the total amount of flow that the protein received

(7) $$f_{uk}=\overset{d}{\underset{t=1}{\sum }}\underset{\left(u,v\right)\in E}{\sum }g_{t}^{k}\left(v,\;u\right)$$

### Evaluation of learning methods

We test the performance using 5-fold cross-validation. In the "mono-localization" case, the standard evaluation criteria is the receiver operating characteristic (ROC) which plot the numbers of true positives (TPs) as a function of the number of false positives (FPs) as the scoring threshold vary. By contrast, in the "multiplex localization" scenario, we adopt the TRECVID performance metric [31], Average Precision (AP) to evaluate and compare the approaches on each subcellular localization. Through averaging the AP over all subcellular localizations, we can obtain the mean average precision (MAP), an overall evaluation. In addition, we also use the F1 micro score to evaluation both the precision and recall together. The F1 micro score for the subcellular localization *k *is defined as

(8) $$F1\left(k\right)=\frac{2p_{k}r_{k}}{p_{k}+r_{k}}$$

where *p_k _*and *r_k _*are the precision and recall of the subcellular localization *k*, respectively. And they can be calculated by using the following equations

(9) $$p_{k}=\frac{\sum_{i=1}^{n}y_{ik}f_{ik}}{\sum_{i=1}^{n}f_{ik}}$$

(10) $$r_{k}=\frac{\sum_{i=1}^{n}y_{ik}f_{ik}}{\sum_{i=1}^{n}y_{ik}}$$

where *y_ik _*and *f_ik _*are the true label and predicted label, respectively.

### Ensemble classifier and predictions

**Figure 4:**
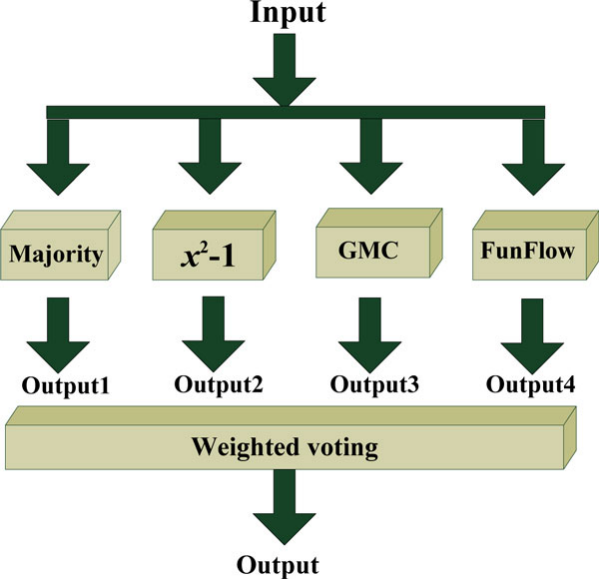
**Flowchart to show the ensemble classifier**. The ensemble classifier ℂ is formed by fusing four basic individual classifiers ℂ_1_, ℂ_2_, ℂ_3 _and ℂ_4 _derived from four graph-based semi-supervised learning.

Considering that all these methods have both advantages and disadvantages (see Methods), we build an ensemble classifier by combining the four classifiers together so as to make predictions for the 667 uncharacterized proteins. This framework can reduce the variance caused by the peculiarities of a single training dataset and hence be able to learn a more comprehensive concept than any single classifier. Figure 4 illustrates the basic framework for the ensemble classifier that consists of these 4 basic classifiers. The final output of the ensemble is the weighted fusion of the outputs produced by the 4 individual classifiers, as formulated below.

The ensemble classifier ℂ is represented as

(11) $$\mathbb{C}={\mathbb{C}}_{1}⊕{\mathbb{C}}_{2}⊕{\mathbb{C}}_{3}⊕{\mathbb{C}}_{4}$$

where ℂ_1_, ℂ_2_, ℂ_3 _and ℂ_4 _represent the 4 basic classifiers, Majority, *χ*^2^-score, GenMultiCut, and FunFlow respectively. The symbol ⊗ denotes the fusing operator. Thus, the process of how the ensemble classifier ℂ works can be formulated

(12) $$f_{uk}=\overset{4}{\underset{c=1}{\sum }}w_{c}f_{uk}^{c}$$

where *f_uk _*is the confidence score that protein *u *should be annotated with the *k*-th localization site, $f_{uk}^{c}$ is the prediction for protein *u *annotated with subcellular localization *k *of the basic classifier ℂ_c_, *c *= 1,..., 4, and *w_c _*is the weighting factor, which was assigned in this study with the value of the AP obtained by the basic classifier ℂ*_c_*. In other words, we define the weighting factor as

(13) $$w_{c}=\frac{{\text{AP}}_{k}^{c}}{\sum_{c=1}^{4}{\text{AP}}_{k}^{c}}$$

where ${\text{AP}}_{k}^{c}$ is the average precision of the basic classifier ℂ*_c _*for subcellular localization *k*.

## Competing interests

The authors declare that they have no competing interests.

## Authors' contributions

JQJ conceived this study, JQJ and MW processed the data and analyzed the result. The manuscript were written by JQJ, reviewed and revised by MW. All authors read and approved the final manuscript.

## Supplementary Material

Additional file 1 **The yeast proteome complied from the BioGRID database**. Click here for file

Additional file 2 **The subcellular localization annotations of 3165 proteins in the PPI network collected by the Yeast Gtp Fusion Localization database**. Click here for file

Additional file 3 **The subnetwork consists of 72 proteins and 204 interactions**. Click here for file

Additional file 4 **The subnetwork consists of 83 proteins and 164 interactions**. Click here for file

Additional file 5 **Prediction for the first group of 60 "ambiguous" proteins**. Click here for file

Additional file 6 **Prediction for the 606 proteins without prior knowledge**. Click here for file

Additional file 7 **Comparison of the four basic classifiers and the ensemble classifier for 46 "ambiguous" proteins that was annotated with more than one subcellular localization sites**. Click here for file
