# Supplementary material for: Predicting multiplex subcellular localization of proteins using protein-protein interaction network: a comparative study
Source: BMC Bioinformatics. 2012 Jun 25;13(Suppl 10):S20. doi: 10.1186/1471-2105-13-S10-S20 (PMC3314587; doi:10.1186/1471-2105-13-S10-S20)
Supplement: Additional file 1 — The yeast proteome complied from the BioGRID database. [file 1471-2105-13-S10-S20-S1.pdf]

Supplementary Table 1

The yeast proteome complied from BioGRID database and contains 3179 proteins and 12413 interactions.

Protein A	Protein B	Weight
Q0045	Q0250	0.9191
Q0045	Q0275	0.9773
Q0045	YGR112W	0.9609
Q0045	YJL062W-A	0.9215
Q0045	YLR203C	1.0000
Q0045	YML129C	0.9938
Q0080	YJR121W	0.9747
Q0080	YLR203C	0.8953
Q0080	YPL078C	0.9555
Q0085	YBL099W	0.9215
Q0085	YER154W	0.9215
Q0085	YJR121W	0.9960
Q0085	YLR203C	0.8953
Q0085	YPL078C	0.9799
Q0105	YLR203C	0.9215
Q0120	YLR382C	0.7031
Q0120	YPL160W	0.8965
Q0130	YBL099W	0.9215
Q0130	YER154W	0.9249
Q0130	YLR203C	0.8953
Q0130	YPL078C	0.9555
Q0250	YBL030C	0.9707
Q0250	YBL045C	0.9215
Q0250	YBR037C	0.9191
Q0250	YER154W	0.9215
Q0250	YLR203C	0.9918
Q0250	YML129C	0.9780
Q0275	YER154W	0.9215
Q0275	YLR203C	0.9707
Q0275	YML129C	0.9215
YAL001C	YBR123C	0.9996
YAL001C	YDR362C	0.9266
YAL001C	YGR047C	0.9266
YAL001C	YOR110W	0.9624
YAL001C	YPL007C	0.7380
YAL002W	YDR495C	0.8953
YAL002W	YLR148W	0.9648
YAL002W	YLR396C	0.9624
YAL002W	YMR231W	0.9895
YAL002W	YOR089C	0.9723

YAL002W	YPL045W	0.9808
YAL003W	YBR118W	0.9960
YAL003W	YFR004W	0.7380
YAL003W	YKL081W	0.9879
YAL003W	YLR249W	0.9539
YAL003W	YPL048W	0.7380
YAL003W	YPR080W	0.9960
YAL005C	YAR002W	0.8566
YAL005C	YAR015W	0.7380
YAL005C	YBL023C	0.7380
YAL005C	YBL057C	0.7380
YAL005C	YBL058W	0.7380
YAL005C	YBL067C	0.7380
YAL005C	YBR017C	0.7380
YAL005C	YBR101C	0.9771
YAL005C	YBR115C	0.7380
YAL005C	YBR159W	0.7380
YAL005C	YBR169C	0.9266
YAL005C	YBR215W	0.7380
YAL005C	YBR231C	0.7380
YAL005C	YBR251W	0.7380
YAL005C	YCL037C	0.7380
YAL005C	YCR008W	0.7380
YAL005C	YDL019C	0.7380
YAL005C	YDL058W	0.7380
YAL005C	YDL139C	0.7380
YAL005C	YDL140C	0.7380
YAL005C	YDL229W	0.9021
YAL005C	YDR060W	0.7380
YAL005C	YDR083W	0.7380
YAL005C	YDR141C	0.7380
YAL005C	YDR168W	0.7380
YAL005C	YDR172W	1.0000
YAL005C	YDR214W	0.7380
YAL005C	YDR301W	0.7380
YAL005C	YDR324C	0.7380
YAL005C	YDR328C	0.8566
YAL005C	YDR337W	0.7380
YAL005C	YDR338C	0.7380
YAL005C	YDR359C	0.7380
YAL005C	YDR385W	0.7380
YAL005C	YDR390C	0.7380
YAL005C	YDR450W	0.7380
YAL005C	YDR460W	0.7380
YAL005C	YDR495C	0.7380
YAL005C	YDR502C	0.7380
YAL005C	YDR510W	0.8659

YAL005C	YDR532C	0.7380
YAL005C	YEL025C	0.7380
YAL005C	YEL071W	0.7380
YAL005C	YER103W	0.7380
YAL005C	YER132C	0.7380
YAL005C	YER165W	0.9790
YAL005C	YFL007W	0.7380
YAL005C	YFL008W	0.7380
YAL005C	YFL044C	0.7380
YAL005C	YFR004W	0.7380
YAL005C	YFR010W	0.7380
YAL005C	YFR019W	0.7380
YAL005C	YFR031C	0.7380
YAL005C	YGL043W	0.7380
YAL005C	YGL064C	0.7380
YAL005C	YGL206C	0.7380
YAL005C	YGL207W	0.7380
YAL005C	YGR071C	0.7380
YAL005C	YGR090W	0.7380
YAL005C	YGR095C	0.7380
YAL005C	YGR198W	0.7380
YAL005C	YGR237C	0.7380
YAL005C	YGR240C	0.7380
YAL005C	YGR264C	0.7380
YAL005C	YHR061C	0.7380
YAL005C	YHR077C	0.7380
YAL005C	YHR113W	0.7380
YAL005C	YHR127W	0.7380
YAL005C	YHR165C	0.7380
YAL005C	YHR200W	0.8087
YAL005C	YIL026C	0.7380
YAL005C	YIL035C	0.7380
YAL005C	YIL043C	0.7380
YAL005C	YIL053W	0.7380
YAL005C	YIL075C	0.7380
YAL005C	YIL091C	0.7380
YAL005C	YIR002C	0.7380
YAL005C	YJL084C	0.7380
YAL005C	YJL109C	0.7380
YAL005C	YJR032W	0.9794
YAL005C	YJR064W	0.7380
YAL005C	YJR073C	0.7380
YAL005C	YJR096W	0.7380
YAL005C	YJR112W	0.7380
YAL005C	YJR140C	0.7380
YAL005C	YKL012W	0.7380
YAL005C	YKL014C	0.7380

YAL005C	YKL073W	0.7380
YAL005C	YKR008W	0.7380
YAL005C	YLL010C	0.7380
YAL005C	YLL024C	0.9717
YAL005C	YLL039C	0.7380
YAL005C	YLR180W	0.7380
YAL005C	YLR187W	0.7380
YAL005C	YLR189C	0.7380
YAL005C	YLR200W	0.7380
YAL005C	YLR209C	0.7380
YAL005C	YLR319C	0.7380
YAL005C	YLR377C	0.8953
YAL005C	YLR384C	0.7380
YAL005C	YLR398C	0.7380
YAL005C	YLR418C	0.7380
YAL005C	YLR424W	0.7380
YAL005C	YML026C	0.7380
YAL005C	YML062C	0.7380
YAL005C	YML088W	0.8953
YAL005C	YMR012W	0.7380
YAL005C	YMR066W	0.7380
YAL005C	YMR076C	0.7380
YAL005C	YMR099C	0.7380
YAL005C	YMR109W	0.7380
YAL005C	YMR128W	0.7380
YAL005C	YMR165C	0.7380
YAL005C	YMR259C	0.7380
YAL005C	YMR261C	0.7380
YAL005C	YNL004W	0.7380
YAL005C	YNL007C	0.9991
YAL005C	YNL064C	0.9999
YAL005C	YNL078W	0.7380
YAL005C	YNL136W	0.7380
YAL005C	YNL220W	0.7380
YAL005C	YNR003C	0.7380
YAL005C	YNR034W	0.7380
YAL005C	YOL006C	0.7380
YAL005C	YOL021C	0.7380
YAL005C	YOL033W	0.7380
YAL005C	YOL097C	0.7380
YAL005C	YOR027W	0.9932
YAL005C	YOR038C	0.7380
YAL005C	YOR048C	0.7380
YAL005C	YOR061W	0.7380
YAL005C	YOR086C	0.7380
YAL005C	YOR110W	0.7380
YAL005C	YOR117W	0.8659

YAL005C	YOR124C	0.7380
YAL005C	YOR132W	0.7380
YAL005C	YOR133W	0.7380
YAL005C	YOR136W	0.7380
YAL005C	YOR140W	0.7380
YAL005C	YOR155C	0.7380
YAL005C	YOR164C	0.8087
YAL005C	YOR227W	0.7380
YAL005C	YPL001W	0.7380
YAL005C	YPL002C	0.7380
YAL005C	YPL004C	0.7380
YAL005C	YPL012W	0.7380
YAL005C	YPL023C	0.7380
YAL005C	YPL028W	0.7380
YAL005C	YPL104W	0.7380
YAL005C	YPL106C	1.0000
YAL005C	YPL108W	0.7380
YAL005C	YPL110C	0.7380
YAL005C	YPL137C	0.7380
YAL005C	YPL165C	0.7380
YAL005C	YPL167C	0.7380
YAL005C	YPL240C	0.8659
YAL005C	YPR131C	0.7380
YAL005C	YPR135W	0.7380
YAL005C	YPR140W	0.7380
YAL005C	YPR163C	0.7380
YAL007C	YAR002C-A	0.9996
YAL007C	YDR087C	0.7380
YAL007C	YGL200C	0.9483
YAL007C	YLR342W	0.7380
YAL007C	YML012W	0.9313
YAL010C	YHR083W	0.9215
YAL010C	YLL006W	0.9780
YAL010C	YOL009C	0.9780
YAL011W	YBR231C	0.8659
YAL011W	YDR190C	0.7380
YAL011W	YDR334W	0.9971
YAL011W	YDR485C	0.7380
YAL011W	YDR510W	0.8566
YAL011W	YJL081C	0.9021
YAL011W	YLR085C	0.7380
YAL011W	YML041C	0.7380
YAL011W	YNL107W	0.9598
YAL011W	YOL012C	0.9478
YAL011W	YPL235W	0.7380
YAL013W	YBR095C	0.9634
YAL013W	YDL076C	0.8087

YAL013W	YDR207C	0.8603
YAL013W	YIL084C	0.9021
YAL013W	YKL185W	0.8603
YAL013W	YMR263W	0.9813
YAL013W	YNL097C	0.8087
YAL013W	YNL330C	0.9997
YAL013W	YOL004W	0.9733
YAL013W	YPL139C	0.9813
YAL013W	YPL181W	0.9634
YAL014C	YER133W	0.9470
YAL015C	YDR510W	0.9266
YAL016W	YAL024C	0.7380
YAL016W	YDL134C	1.0000
YAL016W	YDL188C	0.9998
YAL016W	YFR004W	0.7380
YAL016W	YGL190C	0.9996
YAL016W	YGR161C	0.9820
YAL016W	YGR281W	0.7380
YAL016W	YHR158C	0.7380
YAL016W	YIL153W	0.9215
YAL016W	YML109W	0.9984
YAL016W	YMR273C	0.9808
YAL016W	YNL127W	0.5774
YAL016W	YOR014W	0.9908
YAL016W	YPL152W	0.9942
YAL017W	YDR099W	0.7380
YAL017W	YGL112C	0.7380
YAL017W	YOL045W	0.7380
YAL017W	YPR160W	0.7839
YAL020C	YBL071W-A	0.9927
YAL020C	YKR048C	0.9747
YAL021C	YCR093W	1.0000
YAL021C	YDL165W	0.9988
YAL021C	YDR252W	0.9884
YAL021C	YDR443C	0.9099
YAL021C	YER068W	0.9991
YAL021C	YGR092W	0.9985
YAL021C	YGR134W	0.9980
YAL021C	YIL038C	0.9409
YAL021C	YNL288W	1.0000
YAL021C	YNR052C	1.0000
YAL021C	YPR072W	0.9779
YAL023C	YDL095W	0.9890
YAL023C	YGL200C	0.8953
YAL024C	YBR160W	0.6042
YAL024C	YHR158C	0.9965
YAL024C	YNL098C	0.9981

YAL026C	YCR094W	0.9974
YAL026C	YDR497C	0.8798
YAL026C	YEL022W	0.9099
YAL026C	YJL153C	0.8798
YAL026C	YKL212W	0.8798
YAL026C	YLR447C	0.7380
YAL026C	YML072C	0.8798
YAL026C	YNL321W	0.8798
YAL027W	YDR381W	0.7380
YAL027W	YML032C	0.9249
YAL027W	YML095C	0.8659
YAL027W	YOL090W	0.9249
YAL027W	YPL022W	0.9991
YAL028W	YBR160W	0.3202
YAL029C	YBR109C	0.9298
YAL029C	YBR119W	0.8953
YAL029C	YBR130C	1.0000
YAL029C	YFL039C	0.9973
YAL029C	YGL106W	0.9313
YAL029C	YHR023W	0.8659
YAL029C	YKL130C	0.9998
YAL029C	YOR035C	0.9758
YAL030W	YBL050W	0.9249
YAL030W	YER143W	0.9215
YAL030W	YGR009C	0.9995
YAL030W	YJL036W	0.8179
YAL030W	YLL039C	0.8566
YAL030W	YMR183C	0.9215
YAL030W	YNR049C	0.9780
YAL030W	YPL232W	0.9997
YAL031C	YCL027W	0.5774
YAL031C	YER133W	0.7380
YAL032C	YDL209C	0.9869
YAL032C	YDR364C	0.8659
YAL032C	YDR416W	0.9856
YAL032C	YDR510W	0.8566
YAL032C	YER172C	0.7380
YAL032C	YGL120C	0.9021
YAL032C	YGL128C	0.8659
YAL032C	YGR278W	0.8659
YAL032C	YHR165C	0.7380
YAL032C	YJL203W	0.7380
YAL032C	YKL173W	0.8659
YAL032C	YLL036C	0.9944
YAL032C	YLR117C	0.9958
YAL032C	YLR345W	0.5774
YAL032C	YLR424W	0.8353

YAL032C	YML049C	0.8659
YAL032C	YMR213W	0.9313
YAL032C	YMR288W	0.8659
YAL032C	YPL151C	0.9999
YAL032C	YPL213W	0.9499
YAL032C	YPR101W	0.7380
YAL033W	YBL018C	0.9871
YAL033W	YBR167C	0.9266
YAL033W	YBR257W	0.9912
YAL033W	YGR030C	0.9539
YAL033W	YHR062C	0.9934
YAL033W	YIR015W	0.8566
YAL033W	YLR145W	0.9598
YAL033W	YNL221C	0.9021
YAL033W	YNL282W	0.9266
YAL034C	YER110C	0.9648
YAL034C	YOL004W	0.7380
YAL034W-A	YBR211C	0.8179
YAL034W-A	YDR318W	0.9215
YAL034W-A	YER018C	0.9598
YAL034W-A	YGL093W	0.9266
YAL034W-A	YIL144W	0.9266
YAL034W-A	YIR010W	0.9998
YAL034W-A	YJR112W	1.0000
YAL034W-A	YKL049C	0.9730
YAL034W-A	YMR117C	0.9068
YAL034W-A	YOL069W	0.6672
YAL034W-A	YPL018W	0.9215
YAL034W-A	YPL233W	0.9963
YAL034W-A	YPR046W	0.8953
YAL035W	YBR079C	0.8566
YAL035W	YBR084W	0.7380
YAL035W	YDL014W	0.8659
YAL035W	YDL082W	0.7380
YAL035W	YDR429C	0.7380
YAL035W	YDR496C	0.9972
YAL035W	YER025W	0.9312
YAL035W	YER036C	0.9021
YAL035W	YFR004W	0.7380
YAL035W	YFR031C-A	0.9313
YAL035W	YGL076C	0.7380
YAL035W	YGL130W	0.7380
YAL035W	YJL190C	0.7380
YAL035W	YJR007W	0.8655
YAL035W	YJR123W	0.7380
YAL035W	YJR145C	0.7380
YAL035W	YLL045C	0.7380

YAL035W	YLR175W	0.9313
YAL035W	YMR260C	0.9994
YAL035W	YMR309C	0.9313
YAL035W	YNL178W	0.7380
YAL035W	YOR063W	0.7380
YAL035W	YOR361C	0.9808
YAL035W	YPL237W	0.8655
YAL035W	YPR041W	0.8659
YAL036C	YDR152W	0.9619
YAL036C	YGR162W	0.7380
YAL036C	YOR091W	0.9982
YAL038W	YDR510W	0.7380
YAL038W	YER151C	0.7380
YAL038W	YFR004W	0.7380
YAL038W	YHR200W	0.8087
YAL038W	YJL164C	0.9756
YAL038W	YNL307C	0.8456
YAL038W	YPL203W	0.8714
YAL040C	YBR160W	0.9986
YAL040C	YNL197C	0.7420
YAL041W	YBL085W	0.9464
YAL041W	YBR200W	1.0000
YAL041W	YDR103W	0.9790
YAL041W	YDR379W	0.9464
YAL041W	YER114C	0.9726
YAL041W	YER118C	0.9215
YAL041W	YGR152C	0.9843
YAL041W	YHL007C	0.5107
YAL041W	YJL157C	0.9998
YAL041W	YLR229C	0.9986
YAL041W	YNL298W	0.8655
YAL041W	YOR212W	0.9998
YAL042W	YML067C	0.9584
YAL042W	YML130C	0.7380
YAL043C	YBL076C	0.7380
YAL043C	YBR119W	0.7380
YAL043C	YDR195W	0.9998
YAL043C	YDR228C	0.8998
YAL043C	YDR301W	0.9995
YAL043C	YER133W	1.0000
YAL043C	YGR156W	0.9999
YAL043C	YJL033W	0.7380
YAL043C	YJR093C	0.9999
YAL043C	YKL018W	0.9974
YAL043C	YKL059C	1.0000
YAL043C	YKR002W	0.9999
YAL043C	YLR115W	1.0000

YAL043C	YLR277C	1.0000
YAL043C	YMR039C	0.9249
YAL043C	YMR061W	0.9499
YAL043C	YNL222W	1.0000
YAL043C	YNL317W	1.0000
YAL043C	YOR179C	0.9982
YAL043C	YPL008W	0.8603
YAL043C	YPR107C	0.9993
YAL047C	YGL212W	0.7909
YAL047C	YGR218W	0.9723
YAL047C	YHR172W	1.0000
YAL047C	YLR045C	0.9978
YAL047C	YLR212C	0.9982
YAL047C	YMR001C	0.9890
YAL047C	YNL126W	0.9980
YAL047C	YNL188W	0.9704
YAL047C	YOR373W	0.9747
YAL049C	YNL094W	0.5774
YAL056W	YER020W	0.9975
YAL059W	YDR395W	0.9266
YAL059W	YMR308C	0.8566
YAL062W	YDR388W	0.9099
YAL062W	YLL039C	0.7380
YAR002C-A	YGL200C	0.9992
YAR002C-A	YGR284C	0.7826
YAR002C-A	YLR026C	0.9555
YAR002C-A	YML012W	0.9988
YAR002W	YDL229W	0.8566
YAR002W	YDR159W	0.7380
YAR002W	YER110C	0.9624
YAR002W	YER165W	0.8566
YAR002W	YGL122C	0.5164
YAR002W	YLR335W	0.9999
YAR002W	YLR347C	1.0000
YAR002W	YMR047C	0.9186
YAR002W	YNL189W	1.0000
YAR002W	YPL169C	0.9298
YAR003W	YBR010W	0.8655
YAR003W	YBR175W	0.9977
YAR003W	YBR258C	0.9285
YAR003W	YDR069C	0.7380
YAR003W	YDR469W	0.9904
YAR003W	YHR119W	1.0000
YAR003W	YJR113C	0.7380
YAR003W	YKL018W	0.9021
YAR003W	YLR015W	0.9999
YAR003W	YNL031C	0.8655

YAR003W	YPL138C	0.9982
YAR007C	YBL002W	0.7380
YAR007C	YBL003C	0.7380
YAR007C	YBL023C	0.9215
YAR007C	YBR136W	0.9999
YAR007C	YCR012W	0.7380
YAR007C	YCR066W	0.9978
YAR007C	YDR097C	0.9743
YAR007C	YDR499W	0.9507
YAR007C	YER095W	0.7380
YAR007C	YFL013C	0.8603
YAR007C	YGL207W	0.9249
YAR007C	YHR164C	0.9994
YAR007C	YIR002C	0.9266
YAR007C	YJL173C	0.9995
YAR007C	YLR103C	0.9215
YAR007C	YLR347C	0.8659
YAR007C	YML032C	0.9987
YAR007C	YML069W	0.9790
YAR007C	YNL031C	0.7380
YAR007C	YNL312W	0.9999
YAR007C	YOL090W	0.9313
YAR007C	YOR304W	0.8087
YAR009C	YMR186W	0.7380
YAR009C	YNL209W	0.7380
YAR014C	YDR122W	0.6147
YAR014C	YDR195W	0.5774
YAR014C	YER133W	0.9996
YAR014C	YER155C	0.9157
YAR014C	YJL187C	0.5779
YAR014C	YLR096W	0.8718
YAR014C	YMR071C	0.5774
YAR014C	YNL333W	0.5774
YAR015W	YPL240C	0.6672
YAR018C	YIL144W	0.9249
YAR019C	YFR028C	0.8894
YAR019C	YGR092W	0.9433
YAR019C	YML064C	1.0000
YAR027W	YAR028W	0.5107
YAR027W	YGR284C	0.5107
YAR027W	YLL039C	0.8566
YAR042W	YER120W	0.7380
YAR042W	YHR195W	0.9730
YBL002W	YBL003C	0.9624
YBL002W	YBR009C	0.7380
YBL002W	YBR089C-A	0.9187
YBL002W	YBR111W-A	0.8655

YBL002W	YBR245C	0.8087
YBL002W	YDL229W	0.7380
YBL002W	YDR075W	0.8087
YBL002W	YDR225W	0.9929
YBL002W	YDR510W	0.7380
YBL002W	YER030W	0.9754
YBL002W	YGL241W	0.8659
YBL002W	YKR048C	0.8659
YBL002W	YLL039C	0.9464
YBL002W	YML069W	0.7380
YBL002W	YMR072W	0.7380
YBL002W	YNL201C	0.8087
YBL002W	YOL012C	0.9950
YBL002W	YOL054W	0.7380
YBL003C	YBL046W	0.8566
YBL003C	YBL052C	0.7380
YBL003C	YBR009C	0.9808
YBL003C	YBR010W	0.9624
YBL003C	YBR089C-A	0.9187
YBL003C	YBR245C	0.9499
YBL003C	YCR057C	0.7380
YBL003C	YDL002C	0.8087
YBL003C	YDR075W	0.9464
YBL003C	YDR174W	0.7380
YBL003C	YDR224C	0.9624
YBL003C	YDR225W	0.9871
YBL003C	YDR303C	0.7380
YBL003C	YGL097W	0.8998
YBL003C	YGL207W	0.7380
YBL003C	YGL241W	0.9948
YBL003C	YGR270W	0.7380
YBL003C	YGR275W	0.7380
YBL003C	YHR099W	0.8566
YBL003C	YIL126W	0.8087
YBL003C	YJL081C	0.8628
YBL003C	YKL049C	0.9215
YBL003C	YKL139W	0.9249
YBL003C	YKR048C	0.9986
YBL003C	YLR033W	0.8659
YBL003C	YLR274W	0.7380
YBL003C	YLR432W	0.7380
YBL003C	YML065W	0.9266
YBL003C	YMR072W	0.8659
YBL003C	YNL030W	0.9266
YBL003C	YNL031C	0.8566
YBL003C	YNL088W	0.7380
YBL003C	YNL201C	0.8087

YBL003C	YNL290W	0.7380
YBL003C	YOL004W	0.7380
YBL003C	YOL012C	0.9634
YBL003C	YOR207C	0.7380
YBL003C	YOR217W	0.8659
YBL003C	YOR244W	0.7543
YBL003C	YPL082C	0.8659
YBL003C	YPR019W	0.8087
YBL004W	YBL038W	0.7380
YBL004W	YBR247C	0.9313
YBL004W	YBR251W	0.7380
YBL004W	YCL059C	0.9313
YBL004W	YCR057C	0.9313
YBL004W	YDL148C	0.8659
YBL004W	YDL213C	0.9313
YBL004W	YDR060W	0.7380
YBL004W	YDR280W	0.8659
YBL004W	YDR449C	0.8659
YBL004W	YER082C	0.9313
YBL004W	YGR090W	0.9313
YBL004W	YGR220C	0.7380
YBL004W	YHR196W	0.8659
YBL004W	YJL109C	0.8659
YBL004W	YJR002W	0.9464
YBL004W	YNL110C	0.8659
YBL004W	YNL284C	0.7380
YBL004W	YOL010W	0.8659
YBL004W	YOR078W	0.7380
YBL004W	YOR310C	0.7380
YBL004W	YPL043W	0.9313
YBL004W	YPR137W	0.9313
YBL004W	YPR144C	0.8659
YBL005W-A	YFR004W	0.7380
YBL007C	YBL105C	0.6672
YBL007C	YCL034W	0.9922
YBL007C	YCR030C	0.8297
YBL007C	YCR088W	0.9995
YBL007C	YDL146W	0.7909
YBL007C	YDR172W	0.8965
YBL007C	YDR388W	0.9571
YBL007C	YER125W	0.9249
YBL007C	YER133W	0.9911
YBL007C	YFR024C-A	0.9573
YBL007C	YGL181W	0.9099
YBL007C	YGL206C	0.9435
YBL007C	YGR268C	0.7253
YBL007C	YHR016C	0.9706

YBL007C	YHR114W	0.7380
YBL007C	YIL095W	0.9970
YBL007C	YIR006C	0.9747
YBL007C	YJL201W	0.8297
YBL007C	YKR001C	0.9747
YBL007C	YLL039C	0.8628
YBL007C	YLR064W	0.5774
YBL007C	YNL020C	0.7974
YBL007C	YNL084C	0.9322
YBL007C	YNL094W	0.5774
YBL007C	YNL106C	0.8741
YBL007C	YNL238W	0.8928
YBL007C	YNL243W	0.9351
YBL007C	YOR181W	0.9999
YBL007C	YPR171W	0.8594
YBL008W	YBR215W	0.9634
YBL008W	YGR063C	0.9215
YBL008W	YJL115W	0.9983
YBL008W	YJR140C	0.8603
YBL008W	YOR038C	0.9944
YBL010C	YKR022C	0.5774
YBL014C	YER148W	0.9138
YBL014C	YJL025W	0.9997
YBL014C	YKL125W	0.9747
YBL014C	YML043C	0.9852
YBL016W	YBR083W	0.9907
YBL016W	YDL159W	1.0000
YBL016W	YDR103W	1.0000
YBL016W	YDR388W	0.8714
YBL016W	YDR480W	0.9999
YBL016W	YER075C	0.9944
YBL016W	YER167W	0.8566
YBL016W	YGL158W	0.7380
YBL016W	YGL178W	0.9710
YBL016W	YHR005C	0.9980
YBL016W	YHR084W	0.8820
YBL016W	YJL157C	0.9974
YBL016W	YLR310C	0.9640
YBL016W	YLR362W	0.9994
YBL016W	YNL053W	0.9939
YBL016W	YOL130W	0.5779
YBL016W	YPL049C	0.9998
YBL016W	YPR115W	0.5779
YBL017C	YDR432W	0.7380
YBL017C	YJL029C	0.9191
YBL017C	YJL053W	0.7826
YBL017C	YJL154C	0.7826

YBL017C	YPL049C	0.7380
YBL018C	YBR257W	0.9464
YBL018C	YHR062C	0.9598
YBL018C	YNL221C	0.8087
YBL018C	YNL282W	0.9794
YBL019W	YBR088C	0.9489
YBL021C	YGL237C	0.9878
YBL021C	YHR187W	0.9574
YBL021C	YKL109W	0.9808
YBL021C	YOR358W	0.9878
YBL023C	YBR126C	0.7380
YBL023C	YBR202W	0.9842
YBL023C	YCL061C	0.8953
YBL023C	YDL017W	0.9989
YBL023C	YDR052C	0.9993
YBL023C	YDR099W	0.9215
YBL023C	YEL032W	0.9998
YBL023C	YGL201C	0.9734
YBL023C	YIL150C	0.8965
YBL023C	YJL072C	0.9215
YBL023C	YJL194W	0.9138
YBL023C	YJR046W	0.9249
YBL023C	YLR103C	0.9998
YBL023C	YLR274W	0.9960
YBL023C	YNL262W	0.8566
YBL023C	YPR019W	0.9996
YBL024W	YFR004W	0.7380
YBL025W	YDL113C	0.7909
YBL025W	YLR141W	0.9834
YBL025W	YMR270C	0.8640
YBL026W	YBR055C	0.7380
YBL026W	YCR077C	0.9958
YBL026W	YDL160C	0.9128
YBL026W	YDR378C	0.9984
YBL026W	YDR473C	0.7380
YBL026W	YER029C	0.7380
YBL026W	YER112W	0.9992
YBL026W	YER146W	0.9984
YBL026W	YER172C	0.9996
YBL026W	YGL173C	0.9128
YBL026W	YGR074W	0.7380
YBL026W	YGR075C	0.7380
YBL026W	YGR091W	0.9313
YBL026W	YGR158C	0.5774
YBL026W	YHR165C	0.9743
YBL026W	YJL124C	1.0000
YBL026W	YJR022W	0.9999

YBL026W	YKL173W	0.8659
YBL026W	YLR275W	0.8893
YBL026W	YLR438C-A	0.9999
YBL026W	YMR268C	0.9937
YBL026W	YNL147W	0.9960
YBL026W	YOL149W	0.8179
YBL026W	YOR308C	0.7380
YBL026W	YPR178W	0.8659
YBL027W	YFR004W	0.7380
YBL030C	YBL045C	0.9918
YBL030C	YEL024W	0.8953
YBL030C	YFR004W	0.7380
YBL030C	YGL187C	0.8953
YBL030C	YNL052W	0.8953
YBL030C	YNR017W	0.9215
YBL030C	YOR065W	0.8953
YBL030C	YPR191W	0.9918
YBL032W	YBR084W	0.7380
YBL032W	YDR025W	0.5164
YBL032W	YGL049C	0.7524
YBL032W	YGL122C	0.1074
YBL032W	YGR023W	0.1301
YBL032W	YHR135C	0.6261
YBL032W	YKL185W	0.1991
YBL032W	YLR150W	0.7380
YBL032W	YLR175W	0.7380
YBL032W	YLR332W	0.1301
YBL032W	YML117W	0.7380
YBL032W	YMR310C	0.7524
YBL032W	YNL005C	0.7380
YBL032W	YNL112W	0.7380
YBL032W	YOL139C	0.7380
YBL032W	YPL237W	0.7380
YBL034C	YOL069W	0.7413
YBL035C	YBR087W	0.7380
YBL035C	YDL058W	0.7380
YBL035C	YDR082W	0.9975
YBL035C	YIR008C	0.8659
YBL035C	YKL045W	0.9313
YBL035C	YNL102W	0.9980
YBL035C	YNL290W	0.7380
YBL035C	YNR052C	0.7380
YBL035C	YOL094C	0.7380
YBL035C	YOR217W	0.7380
YBL037W	YJR005W	0.8087
YBL037W	YJR058C	0.6672
YBL037W	YOL062C	0.8659

YBL038W	YCL014W	0.7380
YBL038W	YCR046C	0.7380
YBL038W	YDR164C	0.7380
YBL038W	YDR237W	0.8659
YBL038W	YDR296W	0.7380
YBL038W	YDR322W	0.8659
YBL038W	YDR462W	0.8659
YBL038W	YGR220C	0.7380
YBL038W	YJL063C	0.8659
YBL038W	YJR138W	0.7380
YBL038W	YKR006C	0.7380
YBL038W	YKR085C	0.8659
YBL038W	YLR189C	0.7380
YBL038W	YLR439W	0.8659
YBL038W	YML025C	0.9313
YBL038W	YMR024W	0.8659
YBL038W	YNL005C	0.8659
YBL038W	YNL284C	0.9648
YBL038W	YOR150W	0.8659
YBL038W	YPR100W	0.7380
YBL039C	YBL105C	0.7696
YBL039C	YDR388W	0.8353
YBL039C	YDR394W	0.7380
YBL039C	YFR004W	0.9313
YBL039C	YGL048C	0.7380
YBL039C	YHR027C	0.7380
YBL039C	YHR200W	0.7380
YBL039C	YJR103W	0.5774
YBL039C	YKR059W	0.7380
YBL039C	YLR180W	0.7380
YBL039C	YMR012W	0.7380
YBL039C	YMR024W	0.7380
YBL039C	YOR151C	0.7380
YBL039C	YOR261C	0.7380
YBL041W	YDL147W	0.7380
YBL041W	YER012W	0.9989
YBL041W	YFR050C	0.7380
YBL041W	YGL011C	0.7380
YBL041W	YGR135W	0.7380
YBL041W	YGR253C	0.7380
YBL041W	YHR200W	0.7380
YBL041W	YJL001W	0.8659
YBL041W	YML092C	0.8659
YBL041W	YMR314W	0.7380
YBL041W	YOL038W	0.9313
YBL041W	YOR157C	0.7380
YBL041W	YOR362C	0.8659

YBL041W	YPR103W	0.9891
YBL045C	YFR004W	0.7380
YBL045C	YGL187C	0.9780
YBL045C	YGR112W	0.9707
YBL045C	YJL143W	0.9215
YBL045C	YNR017W	0.9780
YBL045C	YOR065W	0.9215
YBL045C	YPR191W	0.9958
YBL046W	YDL112W	0.7380
YBL046W	YDR075W	1.0000
YBL046W	YDR225W	0.8566
YBL046W	YDR293C	0.8087
YBL046W	YDR533C	0.7380
YBL046W	YGR063C	0.8603
YBL046W	YML010W	0.9964
YBL046W	YNL201C	1.0000
YBL047C	YBR059C	0.5779
YBL047C	YCR030C	0.9996
YBL047C	YDL161W	0.9138
YBL047C	YDR348C	0.7380
YBL047C	YGR241C	0.7909
YBL047C	YLL039C	0.9754
YBL047C	YNL243W	0.7380
YBL047C	YPL204W	0.7380
YBL049W	YCL039W	0.7380
YBL050W	YBR080C	0.9783
YBL050W	YDR189W	0.6996
YBL050W	YDR468C	0.9780
YBL050W	YGL212W	0.9215
YBL050W	YKL196C	0.9938
YBL050W	YLR026C	0.9997
YBL050W	YLR078C	0.9215
YBL050W	YLR093C	0.9936
YBL050W	YLR268W	0.9790
YBL050W	YMR197C	0.9997
YBL050W	YOL018C	0.9875
YBL050W	YOR106W	1.0000
YBL051C	YPL153C	0.9099
YBL051C	YPL204W	0.7380
YBL052C	YGL207W	0.9215
YBL052C	YGR270W	0.8087
YBL052C	YOR064C	0.9730
YBL052C	YPL129W	0.9999
YBL052C	YPR031W	0.7380
YBL056W	YBR097W	0.7380
YBL056W	YDR071C	0.9974
YBL056W	YGR205W	0.6147

YBL056W	YNL250W	0.7380
YBL057C	YEL037C	0.9215
YBL057C	YMR276W	0.9836
YBL058W	YBL078C	0.9151
YBL058W	YDL126C	1.0000
YBL058W	YDL190C	0.9852
YBL058W	YFL044C	0.9512
YBL058W	YKL213C	0.9772
YBL058W	YNL155W	0.6672
YBL060W	YOR094W	0.8927
YBL061C	YBR023C	0.9893
YBL061C	YIL007C	0.5774
YBL066C	YDR247W	0.7839
YBL066C	YPL026C	0.5779
YBL067C	YOL087C	0.7380
YBL068W	YOL061W	0.7909
YBL071W-A	YDR385W	0.8566
YBL071W-A	YGR200C	0.9409
YBL071W-A	YIL103W	0.9794
YBL071W-A	YKL191W	0.9794
YBL071W-A	YLR384C	0.9772
YBL071W-A	YPL086C	0.7891
YBL072C	YBR079C	0.7380
YBL072C	YBR247C	0.8659
YBL072C	YCL059C	0.9499
YBL072C	YDL148C	0.7380
YBL072C	YDR449C	0.8659
YBL072C	YDR510W	0.7380
YBL072C	YFR004W	0.7380
YBL072C	YGL120C	0.7380
YBL072C	YGR090W	0.7380
YBL072C	YHL034C	0.7380
YBL072C	YHR196W	0.7380
YBL072C	YMR229C	0.7380
YBL072C	YNL207W	0.8521
YBL072C	YNL308C	0.7380
YBL072C	YOR078W	0.7380
YBL072C	YOR310C	0.7380
YBL072C	YOR361C	0.7380
YBL072C	YPL012W	0.7380
YBL072C	YPR144C	0.8659
YBL074C	YDR283C	0.7380
YBL074C	YER029C	0.7380
YBL074C	YGR074W	0.7380
YBL074C	YHR165C	0.9999
YBL074C	YKL173W	0.9243
YBL075C	YDL229W	0.7380

YBL075C	YER103W	0.7380
YBL075C	YGL130W	0.7380
YBL075C	YNL209W	0.7380
YBL075C	YOR250C	0.7380
YBL076C	YDL100C	0.7380
YBL076C	YFR004W	0.7380
YBL078C	YHR171W	0.9990
YBL078C	YIL004C	0.9215
YBL078C	YIL146C	0.9099
YBL078C	YLR093C	0.9215
YBL078C	YNL223W	0.9925
YBL078C	YNR007C	0.9985
YBL078C	YOL082W	0.9998
YBL078C	YOL083W	0.7909
YBL079W	YDL088C	0.9249
YBL079W	YDL116W	0.8087
YBL079W	YGR128C	0.8953
YBL079W	YMR153W	0.9970
YBL084C	YDL008W	0.9609
YBL084C	YDR118W	0.9986
YBL084C	YDR260C	0.9927
YBL084C	YFR036W	0.9707
YBL084C	YGL003C	0.9983
YBL084C	YGL116W	0.9191
YBL084C	YGL240W	0.9808
YBL084C	YHR166C	0.9999
YBL084C	YIR025W	0.9980
YBL084C	YKL022C	1.0000
YBL084C	YLR102C	0.7570
YBL084C	YLR127C	0.9996
YBL084C	YNL172W	0.9989
YBL084C	YOR249C	0.9813
YBL084C	YPL106C	0.7380
YBL085W	YBR200W	0.9412
YBL085W	YDR085C	0.5774
YBL085W	YER124C	0.7253
YBL085W	YER158C	0.5774
YBL085W	YFR028C	0.8566
YBL085W	YHL007C	0.6516
YBL085W	YJR056C	0.5774
YBL085W	YML109W	0.7253
YBL085W	YNL078W	0.5774
YBL087C	YDL171C	0.7380
YBL087C	YDR361C	0.9648
YBL087C	YER103W	0.7380
YBL087C	YER110C	0.7380
YBL087C	YKL104C	0.7380

YBL087C	YOL098C	0.7380
YBL087C	YOR206W	0.7380
YBL087C	YPL208W	0.8659
YBL088C	YDR369C	0.9894
YBL088C	YGR099W	0.9780
YBL090W	YBR251W	0.8659
YBL090W	YHL004W	0.9499
YBL090W	YIL093C	0.7380
YBL090W	YPL013C	0.7380
YBL093C	YBR193C	0.9787
YBL093C	YBR253W	0.7380
YBL093C	YDL005C	0.7380
YBL093C	YDR308C	0.8659
YBL093C	YER022W	0.9961
YBL093C	YGL025C	0.9966
YBL093C	YGL127C	0.7380
YBL093C	YGL151W	0.7380
YBL093C	YGR104C	0.9948
YBL093C	YHR041C	0.7380
YBL093C	YHR058C	0.9895
YBL093C	YLR071C	0.9726
YBL093C	YML007W	0.8566
YBL093C	YMR112C	0.7380
YBL093C	YNL236W	0.8659
YBL093C	YNR010W	0.9895
YBL093C	YOL051W	0.9593
YBL093C	YOL135C	0.9583
YBL093C	YOR174W	0.8659
YBL093C	YPL042C	0.7380
YBL093C	YPR070W	0.8659
YBL093C	YPR168W	0.8659
YBL097W	YFR031C	0.9266
YBL097W	YLR086W	0.8566
YBL097W	YLR272C	0.9021
YBL099W	YFR004W	0.7380
YBL099W	YJL180C	0.9099
YBL099W	YJR121W	0.9998
YBL101C	YER125W	0.9670
YBL103C	YBR112C	0.9138
YBL103C	YOL067C	0.9968
YBL103C	YOL108C	0.9099
YBL104C	YGL190C	0.7380
YBL105C	YEL002C	0.9099
YBL105C	YGL022W	0.9099
YBL105C	YGR254W	0.7380
YBL105C	YML109W	0.8640
YBL105C	YMR149W	0.9099

YBL105C	YPR165W	0.9996
YBR003W	YJR022W	0.5774
YBR005W	YER125W	0.9968
YBR009C	YBR010W	0.9999
YBR009C	YBR034C	0.9354
YBR009C	YBR084W	0.7380
YBR009C	YBR089C-A	0.9187
YBR009C	YBR245C	0.9266
YBR009C	YDL042C	0.9778
YBR009C	YDL058W	0.7380
YBR009C	YDR174W	0.7380
YBR009C	YDR190C	0.7380
YBR009C	YDR224C	0.9298
YBR009C	YDR225W	0.9998
YBR009C	YDR227W	0.9799
YBR009C	YDR300C	0.7380
YBR009C	YDR303C	0.9313
YBR009C	YDR510W	0.8566
YBR009C	YEL056W	0.9946
YBR009C	YER110C	0.9892
YBR009C	YFR006W	0.7380
YBR009C	YGL019W	0.7380
YBR009C	YGL097W	0.9626
YBR009C	YGL150C	0.8603
YBR009C	YGL190C	0.8659
YBR009C	YGL207W	0.7380
YBR009C	YGR170W	0.7380
YBR009C	YGR252W	0.9917
YBR009C	YIL126W	0.9716
YBR009C	YJL081C	0.8783
YBR009C	YJL115W	0.9999
YBR009C	YJR132W	0.7380
YBR009C	YJR138W	0.7380
YBR009C	YKL049C	0.8179
YBR009C	YLL022C	0.9987
YBR009C	YLR106C	0.7380
YBR009C	YLR180W	0.7380
YBR009C	YLR340W	0.7380
YBR009C	YLR357W	0.7380
YBR009C	YLR399C	0.9960
YBR009C	YLR442C	1.0000
YBR009C	YLR449W	0.7983
YBR009C	YML065W	0.9266
YBR009C	YML069W	0.8659
YBR009C	YMR072W	0.8659
YBR009C	YMR127C	0.8818
YBR009C	YMR176W	0.7380

YBR009C	YMR308C	0.9897
YBR009C	YNL030W	0.8028
YBR009C	YNL031C	0.9896
YBR009C	YNL132W	0.7380
YBR009C	YNL206C	0.9892
YBR009C	YNL246W	0.9249
YBR009C	YOL012C	0.9900
YBR009C	YOL054W	0.9648
YBR009C	YOR064C	0.8294
YBR009C	YOR244W	0.9620
YBR009C	YOR304W	0.8818
YBR009C	YPL001W	0.9985
YBR009C	YPL082C	0.9266
YBR009C	YPR010C	0.7380
YBR009C	YPR018W	0.9215
YBR010W	YBR089C-A	0.9187
YBR010W	YBR175W	0.8655
YBR010W	YBR245C	0.8566
YBR010W	YDL042C	0.9940
YBR010W	YDR099W	0.9702
YBR010W	YDR225W	0.9999
YBR010W	YDR227W	0.9799
YBR010W	YDR440W	0.9966
YBR010W	YEL056W	0.7380
YBR010W	YER051W	0.9947
YBR010W	YER110C	0.9799
YBR010W	YER177W	0.8889
YBR010W	YGL058W	0.7696
YBR010W	YGL097W	0.8998
YBR010W	YGL150C	0.8603
YBR010W	YGR252W	0.9991
YBR010W	YGR270W	0.9543
YBR010W	YHR090C	0.9985
YBR010W	YHR119W	0.7696
YBR010W	YIL126W	0.9716
YBR010W	YJL081C	0.8783
YBR010W	YJL115W	1.0000
YBR010W	YJL168C	0.9037
YBR010W	YJR119C	0.7696
YBR010W	YKL005C	0.9947
YBR010W	YKL018W	0.8854
YBR010W	YKR008W	0.9754
YBR010W	YKR029C	0.9892
YBR010W	YKR048C	0.8566
YBR010W	YLL002W	0.9981
YBR010W	YLL022C	0.7380
YBR010W	YLR399C	0.9616

YBR010W	YLR442C	1.0000
YBR010W	YML065W	0.9598
YBR010W	YML102W	0.9215
YBR010W	YMR308C	0.9632
YBR010W	YNL030W	0.9669
YBR010W	YNL031C	0.8240
YBR010W	YNL097C	0.9985
YBR010W	YNL206C	0.9970
YBR010W	YNL246W	0.9249
YBR010W	YOL012C	0.9733
YBR010W	YOR064C	1.0000
YBR010W	YOR244W	0.7696
YBR010W	YPL138C	0.9991
YBR010W	YPL153C	0.9623
YBR010W	YPL181W	0.9947
YBR010W	YPL209C	0.8894
YBR010W	YPR018W	0.9941
YBR010W	YPR052C	0.8448
YBR011C	YBR109C	0.8628
YBR011C	YDL203C	0.9313
YBR011C	YFR004W	0.7380
YBR011C	YLR371W	0.9648
YBR011C	YLR425W	0.9313
YBR011C	YPL066W	0.9313
YBR017C	YDL014W	0.9037
YBR017C	YDR062W	0.7380
YBR017C	YDR192C	0.8353
YBR017C	YDR224C	0.9282
YBR017C	YDR225W	0.9936
YBR017C	YER110C	0.7380
YBR017C	YGL092W	0.9099
YBR017C	YGL122C	1.0000
YBR017C	YGL195W	0.7380
YBR017C	YGR005C	0.9632
YBR017C	YGR119C	0.9099
YBR017C	YHL030W	0.7380
YBR017C	YJR077C	0.7380
YBR017C	YKL068W	0.9464
YBR017C	YLR293C	0.9897
YBR017C	YMR012W	0.7380
YBR017C	YMR047C	0.8566
YBR017C	YOL123W	0.9999
YBR017C	YPR093C	0.9107
YBR020W	YML051W	0.9489
YBR023C	YJL099W	0.9215
YBR023C	YJR117W	0.9221
YBR025C	YFR004W	0.7380

YBR025C	YGL195W	0.7380
YBR025C	YHR027C	0.7380
YBR025C	YKL081W	0.8659
YBR025C	YNL088W	0.7380
YBR026C	YDL229W	0.7380
YBR030W	YLR244C	0.6672
YBR031W	YBR142W	0.7380
YBR031W	YCL059C	0.8087
YBR031W	YER082C	0.7380
YBR031W	YFR004W	0.7380
YBR031W	YFR031C-A	0.7380
YBR031W	YJL109C	0.7380
YBR031W	YLL039C	0.7380
YBR031W	YNL132W	0.7380
YBR031W	YOL120C	0.7380
YBR034C	YDR432W	0.9954
YBR034C	YGL122C	0.7696
YBR034C	YHL034C	0.8206
YBR034C	YIL061C	0.6880
YBR034C	YNL030W	0.9354
YBR034C	YOL123W	0.9469
YBR035C	YLL039C	0.8566
YBR036C	YBR161W	0.7891
YBR036C	YDL212W	0.4335
YBR038W	YBR160W	0.3202
YBR038W	YPR106W	0.6672
YBR039W	YJR121W	0.9960
YBR041W	YBR177C	0.7826
YBR041W	YDL052C	0.7826
YBR041W	YHR072W	0.7826
YBR041W	YIL124W	0.7826
YBR041W	YKL094W	0.7826
YBR041W	YKL140W	0.7826
YBR041W	YMR246W	0.9773
YBR041W	YOR317W	0.9927
YBR045C	YER133W	0.9983
YBR048W	YBR247C	0.7380
YBR048W	YCL059C	0.8087
YBR048W	YDL148C	0.7380
YBR048W	YFR004W	0.7380
YBR048W	YLL039C	0.7380
YBR049C	YDR303C	0.7380
YBR049C	YGL234W	0.7380
YBR049C	YGR274C	0.7380
YBR049C	YKR001C	0.7380
YBR049C	YLR357W	0.7380
YBR049C	YMR091C	0.7380

YBR049C	YOL006C	0.7380
YBR049C	YPL082C	0.7380
YBR049C	YPL106C	0.7380
YBR049C	YPR110C	0.7380
YBR050C	YER133W	0.9919
YBR052C	YDR032C	0.9167
YBR055C	YBR152W	0.8659
YBR055C	YDR283C	0.7380
YBR055C	YDR378C	0.7380
YBR055C	YDR473C	0.9648
YBR055C	YER029C	0.8659
YBR055C	YER112W	0.7380
YBR055C	YER146W	0.8659
YBR055C	YER172C	0.9998
YBR055C	YGR074W	0.8659
YBR055C	YGR091W	0.9648
YBR055C	YHR165C	0.9499
YBR055C	YKL173W	0.8659
YBR055C	YLL036C	0.9186
YBR055C	YLR147C	0.7380
YBR055C	YLR438C-A	0.8087
YBR055C	YNL147W	0.8659
YBR055C	YOR159C	0.7380
YBR055C	YOR308C	0.9157
YBR055C	YPR082C	0.8594
YBR055C	YPR178W	0.9944
YBR055C	YPR182W	0.8659
YBR058C-A	YMR296C	0.9215
YBR059C	YER177W	0.7380
YBR059C	YGL213C	0.6672
YBR059C	YLL039C	0.8566
YBR060C	YBR160W	0.6042
YBR060C	YDR052C	0.9907
YBR060C	YDR099W	0.9215
YBR060C	YFR028C	0.9567
YBR060C	YGR024C	0.9099
YBR060C	YGR103W	0.9249
YBR060C	YHR118C	0.9999
YBR060C	YJL194W	0.9282
YBR060C	YJR046W	0.9099
YBR060C	YLL004W	1.0000
YBR060C	YLR117C	0.9099
YBR060C	YML065W	1.0000
YBR060C	YNL261W	1.0000
YBR060C	YPL001W	0.9800
YBR060C	YPL008W	0.9099
YBR060C	YPL138C	0.9747

YBR060C	YPR162C	0.9973
YBR061C	YMR259C	0.8462
YBR061C	YPL183C	0.7380
YBR065C	YDL209C	0.8659
YBR065C	YER172C	0.8659
YBR065C	YHR165C	0.7380
YBR065C	YLL036C	0.9944
YBR065C	YLR117C	0.9499
YBR065C	YMR213W	0.8659
YBR065C	YPL151C	0.9313
YBR065C	YPR101W	0.7380
YBR066C	YDR477W	0.9099
YBR068C	YDL212W	0.4335
YBR069C	YJR077C	0.6147
YBR072W	YBR127C	0.7380
YBR072W	YLL024C	0.7380
YBR072W	YNL064C	0.8659
YBR072W	YOL039W	0.7380
YBR073W	YER095W	0.9249
YBR077C	YGR163W	0.9249
YBR077C	YKR007W	1.0000
YBR077C	YML121W	0.9844
YBR079C	YDR064W	0.7380
YBR079C	YDR091C	0.8659
YBR079C	YDR429C	0.9999
YBR079C	YER025W	0.9624
YBR079C	YGL123W	0.9215
YBR079C	YGR214W	0.8566
YBR079C	YJR007W	0.9313
YBR079C	YLR192C	0.9998
YBR079C	YMR146C	1.0000
YBR079C	YMR309C	1.0000
YBR079C	YNL243W	0.9099
YBR079C	YNL244C	0.9970
YBR079C	YOR039W	0.7380
YBR079C	YOR096W	0.7380
YBR079C	YOR361C	1.0000
YBR079C	YPL081W	0.7380
YBR079C	YPL237W	0.8659
YBR079C	YPR041W	0.9994
YBR080C	YFR004W	0.7380
YBR080C	YGL137W	0.6147
YBR080C	YLR043C	0.9249
YBR081C	YBR111W-A	0.9998
YBR081C	YBR198C	1.0000
YBR081C	YBR253W	0.7380
YBR081C	YCL010C	0.9963

YBR081C	YDR145W	1.0000
YBR081C	YDR167W	1.0000
YBR081C	YDR176W	1.0000
YBR081C	YDR392W	1.0000
YBR081C	YDR448W	1.0000
YBR081C	YEL009C	0.9249
YBR081C	YER148W	0.9507
YBR081C	YGL048C	0.9249
YBR081C	YGL066W	0.9990
YBR081C	YGL112C	1.0000
YBR081C	YGL252C	0.9555
YBR081C	YGR252W	1.0000
YBR081C	YHR099W	1.0000
YBR081C	YLR055C	1.0000
YBR081C	YML007W	0.9313
YBR081C	YMR223W	0.9999
YBR081C	YMR236W	1.0000
YBR081C	YOL148C	1.0000
YBR081C	YPL047W	0.9996
YBR081C	YPL254W	1.0000
YBR082C	YDR059C	0.8566
YBR082C	YER012W	0.9555
YBR082C	YER068W	0.8353
YBR082C	YKL010C	0.7380
YBR082C	YKL034W	0.9099
YBR082C	YLL039C	0.7543
YBR083W	YDR510W	0.9215
YBR083W	YGR040W	0.9266
YBR083W	YHR084W	1.0000
YBR083W	YPL049C	0.9790
YBR084W	YFR031C-A	0.8659
YBR084W	YGL099W	0.7380
YBR084W	YGR285C	0.7380
YBR084W	YHR010W	0.7380
YBR084W	YKL135C	0.7380
YBR084W	YLR075W	0.7380
YBR084W	YLR340W	0.7380
YBR086C	YGR142W	0.9037
YBR086C	YKL130C	0.1282
YBR086C	YLL039C	0.8566
YBR087W	YBR088C	0.9282
YBR087W	YER173W	0.9999
YBR087W	YJR068W	1.0000
YBR087W	YMR078C	0.9998
YBR087W	YNL290W	0.9648
YBR087W	YOL094C	0.9313
YBR087W	YOR144C	0.9992

YBR087W	YOR217W	0.9648
YBR088C	YCR066W	0.9761
YBR088C	YDL064W	0.9259
YBR088C	YDL164C	0.9966
YBR088C	YDR097C	0.9984
YBR088C	YDR409W	0.8894
YBR088C	YDR419W	0.9971
YBR088C	YDR510W	0.9990
YBR088C	YGL058W	0.7696
YBR088C	YHR031C	0.9138
YBR088C	YJL092W	0.9935
YBR088C	YJR043C	0.9997
YBR088C	YJR068W	0.9282
YBR088C	YKL113C	0.9298
YBR088C	YLL039C	0.9974
YBR088C	YLR032W	0.9955
YBR088C	YOL090W	0.9994
YBR089C-A	YCR084C	0.9754
YBR089C-A	YDL092W	0.9187
YBR089C-A	YDR224C	0.9187
YBR089C-A	YDR225W	0.9187
YBR089C-A	YDR311W	0.9187
YBR089C-A	YGL025C	0.9187
YBR089C-A	YGR005C	0.9187
YBR089C-A	YHR154W	0.9187
YBR089C-A	YJL140W	0.9721
YBR089C-A	YNL030W	0.9187
YBR089C-A	YNL031C	0.9187
YBR089C-A	YPL248C	0.9187
YBR089C-A	YPR056W	0.9187
YBR091C	YDL217C	0.9773
YBR091C	YHR005C-A	0.9215
YBR091C	YJL054W	0.9215
YBR094W	YEL015W	0.9313
YBR094W	YOL149W	0.7837
YBR095C	YDL076C	0.9021
YBR095C	YDR207C	0.8603
YBR095C	YIL084C	0.9021
YBR095C	YKL185W	0.8603
YBR095C	YMR263W	0.9813
YBR095C	YNL097C	0.9634
YBR095C	YNL330C	0.9999
YBR095C	YOL004W	0.9964
YBR095C	YPL139C	0.9813
YBR095C	YPL181W	0.9634
YBR095C	YPR023C	0.8087
YBR097W	YBR128C	0.9215

YBR097W	YHR005C	0.9790
YBR097W	YLR240W	0.9963
YBR097W	YLR360W	0.9266
YBR097W	YPL120W	0.9266
YBR098W	YDR386W	0.9999
YBR102C	YDR166C	0.9938
YBR102C	YER008C	0.8659
YBR102C	YGL233W	0.8297
YBR102C	YIL061C	0.9099
YBR102C	YIL068C	0.9946
YBR102C	YJL085W	0.9266
YBR102C	YLR166C	0.9995
YBR102C	YLR423C	0.5774
YBR102C	YML097C	0.7380
YBR102C	YPR032W	0.9758
YBR102C	YPR055W	0.9992
YBR103W	YCR033W	0.9993
YBR103W	YDR155C	0.9981
YBR103W	YGL194C	0.9904
YBR103W	YIL112W	0.9984
YBR103W	YJR141W	0.5774
YBR103W	YKR029C	0.9986
YBR103W	YLR347C	0.8659
YBR103W	YMR273C	0.7380
YBR103W	YOL068C	0.9634
YBR105C	YDL082W	0.7380
YBR105C	YGL227W	0.9021
YBR105C	YIL097W	0.7380
YBR105C	YMR061W	0.7380
YBR105C	YMR135C	0.7380
YBR106W	YBR283C	0.4335
YBR106W	YDR297W	0.4335
YBR106W	YER056C	0.4335
YBR106W	YFR004W	0.7380
YBR106W	YGL055W	0.4335
YBR106W	YGR060W	0.4335
YBR106W	YHR094C	0.4335
YBR106W	YJR010C-A	0.4335
YBR106W	YMR149W	0.4335
YBR106W	YPR156C	0.4335
YBR107C	YBR211C	0.9624
YBR107C	YDR254W	0.9998
YBR107C	YDR318W	0.8659
YBR107C	YDR421W	0.5774
YBR107C	YGL071W	0.5774
YBR107C	YGR179C	0.8566
YBR107C	YHR113W	0.5774

YBR107C	YJR135C	0.8659
YBR107C	YPL018W	0.9748
YBR107C	YPR046W	0.8087
YBR108W	YCR009C	0.9304
YBR108W	YDR388W	0.9973
YBR108W	YGR092W	0.6042
YBR108W	YHR016C	0.7833
YBR109C	YBR130C	0.8659
YBR109C	YDR155C	0.7380
YBR109C	YDR356W	1.0000
YBR109C	YFL004W	0.9249
YBR109C	YFR004W	0.8628
YBR109C	YFR014C	0.9421
YBR109C	YGL106W	0.9128
YBR109C	YGL242C	0.7380
YBR109C	YJL012C	0.9249
YBR109C	YJR104C	0.7380
YBR109C	YKL042W	0.9932
YBR109C	YKL129C	0.8659
YBR109C	YLL040C	0.7380
YBR109C	YLR433C	0.9779
YBR109C	YML057W	0.9816
YBR109C	YMR109W	0.9995
YBR109C	YNR035C	0.9932
YBR109C	YOL016C	0.9298
YBR109C	YOR326W	0.9990
YBR109C	YPL019C	0.9249
YBR109C	YPL242C	0.9151
YBR111W-A	YDR145W	0.9464
YBR111W-A	YDR159W	1.0000
YBR111W-A	YDR176W	0.9285
YBR111W-A	YDR224C	0.8655
YBR111W-A	YDR448W	0.9923
YBR111W-A	YGL066W	0.9925
YBR111W-A	YGL112C	0.9569
YBR111W-A	YGR252W	0.8566
YBR111W-A	YHR099W	0.9994
YBR111W-A	YLR055C	0.8087
YBR111W-A	YMR223W	0.9998
YBR111W-A	YOL072W	1.0000
YBR111W-A	YOL148C	0.7380
YBR111W-A	YOR257W	1.0000
YBR111W-A	YPL047W	1.0000
YBR111W-A	YPL169C	0.8356
YBR112C	YCR084C	1.0000
YBR112C	YDR043C	0.9099
YBR112C	YGL025C	0.9249

YBR112C	YGL035C	0.9099
YBR112C	YGL071W	0.9249
YBR112C	YGL162W	0.9138
YBR112C	YGL194C	0.9938
YBR112C	YJL168C	0.9249
YBR112C	YKL038W	0.9747
YBR112C	YLR176C	0.9282
YBR112C	YOR140W	0.9138
YBR112C	YPL181W	0.9249
YBR114W	YCR028C-A	0.7380
YBR114W	YDR097C	0.7380
YBR114W	YJR052W	1.0000
YBR114W	YKR001C	0.7380
YBR114W	YMR190C	0.9099
YBR114W	YMR201C	0.8628
YBR114W	YMR315W	0.7380
YBR114W	YOL097C	0.7380
YBR114W	YPL046C	0.9875
YBR114W	YPL154C	0.7380
YBR115C	YGL154C	0.7380
YBR117C	YPR074C	0.8659
YBR118W	YDR510W	0.8659
YBR118W	YFR004W	0.7380
YBR118W	YGR092W	0.6042
YBR118W	YKL081W	0.7380
YBR118W	YLR249W	0.9215
YBR119W	YBR130C	0.8953
YBR119W	YDL087C	0.7380
YBR119W	YDR235W	0.9648
YBR119W	YDR240C	0.9648
YBR119W	YDR432W	0.9037
YBR119W	YER029C	0.8659
YBR119W	YFL017W-A	0.7380
YBR119W	YGR013W	0.9933
YBR119W	YGR074W	0.7380
YBR119W	YHR086W	0.9743
YBR119W	YIL061C	0.9808
YBR119W	YKL012W	0.9820
YBR119W	YKL130C	0.8953
YBR119W	YLR147C	0.7380
YBR119W	YLR275W	0.7380
YBR119W	YLR298C	0.9313
YBR119W	YML046W	0.9648
YBR119W	YMR125W	0.9648
YBR119W	YPL178W	0.8659
YBR121C	YDR510W	0.7380
YBR121C	YFR004W	0.7380

YBR122C	YDR296W	0.7380
YBR122C	YGR220C	0.8659
YBR122C	YML025C	0.7380
YBR122C	YNL284C	0.7380
YBR123C	YDR362C	0.9996
YBR123C	YGR047C	0.9998
YBR123C	YKL068W	0.8087
YBR123C	YOR110W	1.0000
YBR123C	YPL007C	0.9648
YBR125C	YDR071C	0.9931
YBR125C	YDR186C	0.9313
YBR125C	YDR247W	0.8659
YBR126C	YBR265W	0.7380
YBR126C	YDR028C	0.7380
YBR126C	YDR074W	0.9942
YBR126C	YFR004W	0.7380
YBR126C	YJR138W	0.7380
YBR126C	YKR059W	0.7380
YBR126C	YML100W	0.9926
YBR126C	YMR261C	0.9926
YBR126C	YNL076W	0.7380
YBR126C	YPR035W	0.7380
YBR127C	YDL185W	1.0000
YBR127C	YDR328C	0.8566
YBR127C	YEL051W	0.8659
YBR127C	YFR004W	0.7380
YBR127C	YGR020C	0.8659
YBR127C	YHR039C-A	0.9998
YBR127C	YJR033C	0.9808
YBR127C	YKL080W	0.9313
YBR127C	YLR447C	0.7380
YBR127C	YOR270C	0.9991
YBR127C	YOR332W	0.9772
YBR127C	YPR036W	0.9539
YBR128C	YLR240W	0.9215
YBR128C	YPL120W	0.9942
YBR130C	YGL106W	0.9648
YBR130C	YKL130C	1.0000
YBR130C	YKL185W	0.3670
YBR130C	YPL240C	0.5774
YBR131W	YGL124C	0.9992
YBR133C	YHL007C	0.9099
YBR133C	YJL187C	1.0000
YBR133C	YKL101W	1.0000
YBR135W	YBR160W	1.0000
YBR135W	YDL155W	0.9940
YBR135W	YGR108W	0.7837

YBR135W	YKR091W	0.7380
YBR135W	YLR079W	0.9648
YBR135W	YMR199W	0.9820
YBR135W	YPL256C	0.9906
YBR135W	YPR119W	0.9950
YBR135W	YPR120C	0.8659
YBR136W	YBR143C	0.7380
YBR136W	YDL101C	0.7696
YBR136W	YDL220C	0.7696
YBR136W	YDR097C	0.7380
YBR136W	YDR499W	1.0000
YBR136W	YJL173C	0.8659
YBR136W	YNL312W	0.9996
YBR136W	YOR027W	0.7380
YBR136W	YPL153C	0.9980
YBR136W	YPR010C	0.8714
YBR137W	YOL111C	0.8087
YBR137W	YOR007C	0.9021
YBR138C	YBR160W	0.3202
YBR138C	YGR092W	0.6042
YBR142W	YDR060W	0.8659
YBR142W	YDR496C	0.8659
YBR142W	YGL076C	0.7380
YBR142W	YGL111W	0.7380
YBR142W	YHR052W	0.8659
YBR142W	YKL014C	0.9021
YBR142W	YLL045C	0.7380
YBR142W	YML073C	0.7380
YBR142W	YMR049C	0.8659
YBR142W	YMR229C	0.7380
YBR142W	YMR290C	0.9624
YBR142W	YNL002C	0.8659
YBR142W	YNL061W	0.9648
YBR142W	YOL041C	0.8659
YBR142W	YOR063W	0.7380
YBR142W	YOR272W	0.9313
YBR142W	YPL043W	0.8659
YBR142W	YPL093W	0.9021
YBR142W	YPL131W	0.7380
YBR142W	YPL198W	0.7380
YBR142W	YPL249C-A	0.7380
YBR143C	YDL058W	0.7380
YBR143C	YDL207W	0.9249
YBR143C	YDR091C	0.9215
YBR143C	YDR172W	1.0000
YBR143C	YFR004W	0.7380
YBR143C	YIL129C	0.7380

YBR143C	YLR371W	0.7380
YBR143C	YLR386W	0.7380
YBR143C	YLR429W	0.7380
YBR143C	YPL083C	0.7380
YBR146W	YBR251W	0.9313
YBR146W	YDR036C	0.8659
YBR146W	YDR041W	0.7380
YBR146W	YDR337W	0.7380
YBR146W	YER050C	0.7380
YBR146W	YGR084C	0.7380
YBR146W	YHL004W	0.9499
YBR146W	YKL155C	0.8566
YBR146W	YNL137C	0.7380
YBR146W	YPL013C	0.7380
YBR149W	YFR004W	0.7380
YBR152W	YDL030W	0.7380
YBR152W	YDL043C	0.7380
YBR152W	YDL098C	0.9313
YBR152W	YDR473C	0.9313
YBR152W	YER029C	0.9313
YBR152W	YER172C	0.9772
YBR152W	YGR075C	0.9856
YBR152W	YGR091W	0.9648
YBR152W	YIR009W	0.7380
YBR152W	YJL203W	0.7380
YBR152W	YKL173W	0.7380
YBR152W	YML049C	0.7380
YBR152W	YMR240C	0.7380
YBR152W	YMR288W	0.7380
YBR152W	YOR308C	0.8659
YBR152W	YPL213W	0.7380
YBR152W	YPR178W	0.8659
YBR154C	YDL115C	0.8659
YBR154C	YDL140C	1.0000
YBR154C	YDL150W	0.8659
YBR154C	YDR156W	0.7380
YBR154C	YDR404C	0.9860
YBR154C	YER125W	0.7380
YBR154C	YFL023W	0.8991
YBR154C	YGL043W	0.7380
YBR154C	YGL070C	0.9969
YBR154C	YGR005C	0.9808
YBR154C	YGR063C	0.7380
YBR154C	YGR186W	0.8659
YBR154C	YGR200C	0.9555
YBR154C	YIL021W	0.9999
YBR154C	YJL011C	0.7380

YBR154C	YJL140W	0.8659
YBR154C	YJL148W	0.9313
YBR154C	YJR063W	0.9313
YBR154C	YKL144C	0.9313
YBR154C	YKR008W	0.9138
YBR154C	YLR200W	0.6147
YBR154C	YML010W	0.8659
YBR154C	YNL113W	0.8659
YBR154C	YNL248C	0.9648
YBR154C	YNR003C	0.9313
YBR154C	YOL005C	0.9021
YBR154C	YOR116C	0.9861
YBR154C	YOR151C	0.9483
YBR154C	YOR207C	0.9813
YBR154C	YOR210W	0.7380
YBR154C	YOR224C	0.9897
YBR154C	YOR340C	0.9808
YBR154C	YOR341W	1.0000
YBR154C	YPL129W	0.9483
YBR154C	YPR010C	0.9483
YBR154C	YPR110C	0.9908
YBR154C	YPR187W	0.9624
YBR154C	YPR190C	0.7380
YBR155W	YGR187C	0.6147
YBR155W	YJR032W	0.9936
YBR155W	YMR186W	0.8566
YBR155W	YPL240C	0.9997
YBR156C	YER016W	0.8258
YBR156C	YFR028C	0.9690
YBR156C	YGR113W	0.9414
YBR156C	YHR199C-A	0.9099
YBR156C	YJR089W	0.9999
YBR156C	YKR037C	0.5774
YBR156C	YPL209C	1.0000
YBR158W	YGR285C	0.7380
YBR158W	YHR064C	0.7380
YBR158W	YML064C	0.9790
YBR160W	YDL106C	0.8456
YBR160W	YDL155W	0.9996
YBR160W	YDL220C	0.8100
YBR160W	YDR113C	0.9125
YBR160W	YDR130C	0.6042
YBR160W	YDR146C	0.4640
YBR160W	YDR168W	0.8965
YBR160W	YDR356W	0.6042
YBR160W	YDR507C	0.7690
YBR160W	YEL032W	0.6737

YBR160W	YER012W	0.9555
YBR160W	YER021W	0.9555
YBR160W	YER041W	0.6042
YBR160W	YER111C	0.9567
YBR160W	YFL029C	0.9996
YBR160W	YFR030W	0.7380
YBR160W	YFR046C	0.6042
YBR160W	YGL003C	0.9937
YBR160W	YGL075C	0.6042
YBR160W	YGL178W	0.9710
YBR160W	YGR108W	0.9487
YBR160W	YGR238C	0.3202
YBR160W	YHR030C	0.6387
YBR160W	YHR118C	0.8100
YBR160W	YIL050W	0.4640
YBR160W	YJL157C	0.9997
YBR160W	YJL187C	0.9993
YBR160W	YJL194W	0.9999
YBR160W	YJR092W	0.3202
YBR160W	YKL048C	0.7347
YBR160W	YKL108W	0.6042
YBR160W	YKL145W	0.9555
YBR160W	YKL185W	0.8100
YBR160W	YLR079W	0.9993
YBR160W	YLR096W	0.5779
YBR160W	YLR182W	0.8727
YBR160W	YLR187W	0.5779
YBR160W	YLR210W	0.9543
YBR160W	YLR425W	0.6042
YBR160W	YML065W	0.5779
YBR160W	YMR001C	0.9088
YBR160W	YMR036C	0.6042
YBR160W	YMR165C	0.6042
YBR160W	YMR199W	0.9955
YBR160W	YNL068C	0.8939
YBR160W	YNL309W	0.8100
YBR160W	YNR047W	0.3202
YBR160W	YOR066W	0.4640
YBR160W	YOR083W	0.8100
YBR160W	YOR177C	0.3202
YBR160W	YOR315W	0.4640
YBR160W	YOR372C	0.8100
YBR160W	YPL194W	0.6042
YBR160W	YPL256C	1.0000
YBR160W	YPL267W	0.6042
YBR160W	YPL269W	0.9259
YBR160W	YPR119W	1.0000

YBR160W	YPR120C	0.9962
YBR160W	YPR141C	0.4640
YBR160W	YPR175W	0.6042
YBR164C	YDL226C	0.9963
YBR164C	YLR309C	0.9780
YBR164C	YNL297C	0.9758
YBR164C	YPR095C	0.9099
YBR167C	YBR257W	0.9987
YBR167C	YGR030C	0.9997
YBR167C	YHR062C	0.9970
YBR167C	YLR145W	0.9215
YBR167C	YNL221C	0.9966
YBR167C	YNL282W	0.9598
YBR168W	YHR150W	0.8965
YBR168W	YLR324W	0.8965
YBR169C	YDL229W	0.9266
YBR169C	YDR171W	0.8659
YBR169C	YJL050W	0.7380
YBR169C	YKL139W	0.7380
YBR169C	YMR176W	0.7380
YBR169C	YNL209W	0.9624
YBR169C	YOL013C	0.7380
YBR169C	YPL106C	0.7380
YBR170C	YBR201W	0.8953
YBR170C	YDL126C	1.0000
YBR170C	YDL190C	0.9852
YBR170C	YFL044C	0.9249
YBR170C	YGR048W	1.0000
YBR170C	YKL213C	0.9555
YBR170C	YML013W	0.8953
YBR171W	YLR292C	0.9918
YBR171W	YOR254C	1.0000
YBR171W	YPL094C	0.9860
YBR172C	YKL074C	0.7570
YBR172C	YKL204W	0.9707
YBR172C	YLR116W	0.7570
YBR173C	YER012W	0.8087
YBR173C	YER094C	0.8087
YBR173C	YGL011C	0.9384
YBR173C	YGR135W	0.8087
YBR173C	YGR253C	0.8087
YBR173C	YKL206C	0.8566
YBR173C	YLR021W	0.9037
YBR173C	YLR199C	0.8566
YBR173C	YML092C	0.8087
YBR173C	YMR314W	0.8087
YBR173C	YOL038W	0.8087

YBR173C	YOR157C	0.9957
YBR173C	YOR362C	0.8087
YBR173C	YPL144W	0.9037
YBR173C	YPR103W	0.9809
YBR175W	YBR258C	0.9285
YBR175W	YDR469W	0.9285
YBR175W	YHR119W	0.9994
YBR175W	YKL018W	0.9021
YBR175W	YLR015W	0.9999
YBR175W	YNL031C	0.8655
YBR175W	YPL138C	0.9982
YBR177C	YFR004W	0.7380
YBR179C	YDR470C	0.9983
YBR179C	YLL039C	0.8566
YBR179C	YOR211C	0.9773
YBR181C	YFR004W	0.7380
YBR182C	YLR113W	0.9567
YBR185C	YLR439W	0.8603
YBR188C	YDR416W	0.9997
YBR188C	YJR050W	0.9215
YBR188C	YLL036C	1.0000
YBR188C	YLR117C	0.9993
YBR188C	YPR101W	0.9598
YBR189W	YBR247C	0.8659
YBR189W	YCR057C	0.7380
YBR189W	YDL060W	0.8659
YBR189W	YFR004W	0.7380
YBR189W	YGR081C	0.7380
YBR189W	YGR214W	0.7380
YBR189W	YHR196W	0.7380
YBR189W	YJR002W	0.8566
YBR189W	YNL132W	0.7380
YBR189W	YOR310C	0.7380
YBR189W	YPL081W	0.7380
YBR191W	YFR004W	0.7380
YBR193C	YBR253W	0.9891
YBR193C	YDL005C	0.9967
YBR193C	YDR308C	0.9883
YBR193C	YER022W	1.0000
YBR193C	YGL025C	0.9994
YBR193C	YGL127C	0.9866
YBR193C	YGL151W	0.9624
YBR193C	YGL253W	0.9922
YBR193C	YGR104C	1.0000
YBR193C	YHR041C	0.9990
YBR193C	YHR058C	0.9883
YBR193C	YKL038W	0.9099

YBR193C	YLR071C	0.9998
YBR193C	YMR112C	0.9967
YBR193C	YNL236W	0.9940
YBR193C	YNR010W	0.9883
YBR193C	YOL051W	0.9777
YBR193C	YOL135C	0.9991
YBR193C	YOR174W	0.9981
YBR193C	YPR070W	0.9983
YBR193C	YPR168W	0.7380
YBR195C	YML102W	0.9624
YBR195C	YNL183C	0.9710
YBR195C	YPR018W	0.9999
YBR196C	YDR164C	0.7380
YBR196C	YFR004W	0.7380
YBR198C	YCL010C	0.9313
YBR198C	YCR042C	0.9808
YBR198C	YDR145W	1.0000
YBR198C	YDR167W	1.0000
YBR198C	YDR176W	0.9999
YBR198C	YDR392W	0.9940
YBR198C	YDR448W	1.0000
YBR198C	YEL009C	0.9780
YBR198C	YER148W	1.0000
YBR198C	YGL066W	0.9648
YBR198C	YGL112C	1.0000
YBR198C	YGR252W	0.9991
YBR198C	YGR274C	1.0000
YBR198C	YHR041C	0.9555
YBR198C	YHR099W	0.8659
YBR198C	YLR055C	0.9998
YBR198C	YML015C	0.9998
YBR198C	YML098W	0.9648
YBR198C	YML114C	0.9871
YBR198C	YMR005W	0.9999
YBR198C	YMR223W	0.9928
YBR198C	YMR227C	0.9962
YBR198C	YMR236W	1.0000
YBR198C	YNL216W	0.8986
YBR198C	YOL148C	0.9984
YBR198C	YPL011C	0.9974
YBR198C	YPL047W	0.9813
YBR198C	YPL082C	0.8953
YBR198C	YPL129W	0.7380
YBR198C	YPL254W	0.9989
YBR200W	YDL028C	0.6672
YBR200W	YDR103W	0.9747
YBR200W	YDR166C	0.9679

YBR200W	YER114C	0.9955
YBR200W	YFL039C	0.9191
YBR200W	YGL233W	0.9994
YBR200W	YHL007C	1.0000
YBR200W	YJL157C	0.9999
YBR200W	YLR166C	0.9574
YBR200W	YLR229C	1.0000
YBR200W	YNL298W	0.8629
YBR200W	YOR181W	0.9191
YBR200W	YPR055W	0.9555
YBR201W	YDL126C	0.9707
YBR201W	YGR048W	0.9707
YBR201W	YLR207W	0.8953
YBR201W	YML013W	0.8953
YBR201W	YML029W	0.9746
YBR201W	YOL013C	0.9609
YBR202W	YCR086W	0.9099
YBR202W	YDR489W	0.9707
YBR202W	YEL032W	1.0000
YBR202W	YGL001C	0.7380
YBR202W	YIL150C	0.9667
YBR202W	YJL194W	0.9891
YBR202W	YLR103C	0.9870
YBR202W	YLR274W	0.9997
YBR202W	YPR019W	1.0000
YBR205W	YDR170C	0.7380
YBR205W	YHR105W	0.7909
YBR205W	YHR181W	0.9730
YBR208C	YBR221C	0.7380
YBR211C	YDR254W	0.9266
YBR211C	YDR318W	0.9788
YBR211C	YDR383C	0.8566
YBR211C	YDR394W	0.7909
YBR211C	YGR179C	0.9932
YBR211C	YIR010W	0.7380
YBR211C	YJR135C	0.7380
YBR211C	YLR315W	0.8297
YBR211C	YPL018W	0.9523
YBR211C	YPL233W	0.8566
YBR211C	YPR046W	0.9464
YBR215W	YDR510W	0.7380
YBR215W	YJL115W	0.9285
YBR215W	YJR140C	0.9634
YBR215W	YOR038C	0.9634
YBR216C	YML007W	0.9980
YBR217W	YHR171W	0.9961
YBR217W	YLL042C	0.9747

YBR217W	YLR423C	0.6672
YBR217W	YMR159C	0.9998
YBR217W	YNR007C	0.9164
YBR217W	YPL149W	0.9973
YBR221C	YDR069C	0.7380
YBR221C	YDR430C	0.7380
YBR221C	YER178W	0.9313
YBR221C	YFL018C	0.9313
YBR221C	YGR193C	0.9313
YBR221C	YMR308C	0.7380
YBR221C	YNL071W	0.9648
YBR221C	YPL151C	0.7380
YBR223C	YDR510W	0.5774
YBR225W	YGL197W	0.8659
YBR225W	YLR310C	0.8659
YBR225W	YNL307C	0.5779
YBR225W	YPL204W	0.7380
YBR228W	YHR154W	0.8566
YBR228W	YLR135W	0.9983
YBR229C	YDR221W	0.9876
YBR229C	YML067C	0.8566
YBR231C	YDR190C	0.8659
YBR231C	YDR334W	0.9999
YBR231C	YDR485C	0.7380
YBR231C	YFL039C	0.7380
YBR231C	YGR002C	0.9021
YBR231C	YJL081C	0.9499
YBR231C	YLR085C	0.7380
YBR231C	YLR385C	0.8659
YBR231C	YLR399C	0.8659
YBR231C	YML041C	0.8659
YBR231C	YNL107W	0.8462
YBR231C	YOL012C	0.8603
YBR231C	YPL235W	0.8659
YBR233W-A	YDR016C	0.9996
YBR233W-A	YKR037C	0.9974
YBR234C	YDL029W	1.0000
YBR234C	YIL062C	0.9743
YBR234C	YJR065C	1.0000
YBR234C	YKL013C	0.9901
YBR234C	YKL129C	0.9747
YBR234C	YLR370C	0.9901
YBR234C	YMR109W	0.9351
YBR234C	YNR035C	0.9993
YBR234C	YOR181W	0.9907
YBR236C	YDL140C	0.9249
YBR236C	YML010W	0.8659

YBR239C	YDR388W	0.7833
YBR239C	YHR016C	0.7833
YBR245C	YCR052W	0.8659
YBR245C	YDR190C	0.8659
YBR245C	YDR224C	0.8087
YBR245C	YDR303C	0.7380
YBR245C	YDR510W	0.7380
YBR245C	YER164W	0.9266
YBR245C	YFR013W	0.9985
YBR245C	YFR037C	0.9313
YBR245C	YGL133W	0.9313
YBR245C	YKR001C	0.8659
YBR245C	YKR008W	0.8659
YBR245C	YLR033W	0.8659
YBR245C	YLR095C	0.9464
YBR245C	YLR357W	0.8659
YBR245C	YMR044W	0.8566
YBR245C	YMR072W	0.7380
YBR245C	YMR091C	0.8659
YBR245C	YNL030W	0.8566
YBR245C	YOL004W	0.9313
YBR245C	YOL017W	0.8659
YBR245C	YOR304W	0.9624
YBR245C	YPL082C	0.9963
YBR247C	YCL059C	0.9981
YBR247C	YCR031C	0.7380
YBR247C	YCR057C	0.9648
YBR247C	YDL014W	0.9808
YBR247C	YDL060W	0.9997
YBR247C	YDL148C	0.9933
YBR247C	YDR064W	0.8659
YBR247C	YDR324C	0.8087
YBR247C	YDR447C	0.7380
YBR247C	YDR449C	0.9313
YBR247C	YDR450W	0.7380
YBR247C	YER082C	0.9499
YBR247C	YGR081C	0.8028
YBR247C	YGR090W	0.9820
YBR247C	YHR148W	0.9313
YBR247C	YJL109C	0.7380
YBR247C	YJR002W	0.9313
YBR247C	YJR145C	0.8659
YBR247C	YKL143W	0.9979
YBR247C	YKL180W	0.7380
YBR247C	YLL011W	0.8659
YBR247C	YLR129W	0.7380
YBR247C	YLR175W	0.8659

YBR247C	YLR186W	0.9313
YBR247C	YLR367W	0.7380
YBR247C	YML024W	0.7380
YBR247C	YML063W	0.7380
YBR247C	YMR093W	0.9313
YBR247C	YMR128W	0.9313
YBR247C	YMR229C	0.7380
YBR247C	YNL075W	0.8659
YBR247C	YNL132W	0.9820
YBR247C	YNL178W	0.8659
YBR247C	YNL207W	0.9994
YBR247C	YOL010W	0.8659
YBR247C	YOR056C	0.9904
YBR247C	YOR078W	0.7380
YBR247C	YOR145C	0.8087
YBR247C	YPL012W	0.9904
YBR247C	YPL204W	0.9995
YBR247C	YPL217C	0.8659
YBR247C	YPL266W	0.9021
YBR247C	YPR144C	0.9933
YBR248C	YFR004W	0.7380
YBR249C	YDR510W	0.6672
YBR251W	YDR036C	0.7380
YBR251W	YDR041W	0.9313
YBR251W	YDR175C	0.9313
YBR251W	YDR337W	0.9313
YBR251W	YDR347W	0.9313
YBR251W	YER155C	0.7380
YBR251W	YGL129C	0.9313
YBR251W	YGR084C	0.8659
YBR251W	YGR165W	0.7380
YBR251W	YGR170W	0.7380
YBR251W	YGR215W	0.8659
YBR251W	YHL004W	0.9933
YBR251W	YIL093C	0.9313
YBR251W	YJR101W	0.9313
YBR251W	YJR113C	0.9313
YBR251W	YKL003C	0.8659
YBR251W	YKL155C	0.9808
YBR251W	YMR128W	0.7380
YBR251W	YNL137C	0.9313
YBR251W	YNL186W	0.7380
YBR251W	YNL306W	0.9313
YBR251W	YNR037C	0.7380
YBR251W	YOL115W	0.7380
YBR251W	YOR158W	0.7380
YBR251W	YOR243C	0.7380

YBR251W	YPL013C	0.8659
YBR251W	YPL118W	0.9313
YBR253W	YCR081W	0.8659
YBR253W	YDL005C	0.7380
YBR253W	YDL140C	0.9616
YBR253W	YDR308C	0.9313
YBR253W	YDR443C	0.8659
YBR253W	YDR448W	0.8659
YBR253W	YER022W	1.0000
YBR253W	YGL025C	0.9313
YBR253W	YGL127C	0.7380
YBR253W	YGL151W	0.9313
YBR253W	YGR104C	0.9998
YBR253W	YGR252W	0.7380
YBR253W	YHR041C	0.9313
YBR253W	YHR058C	0.9313
YBR253W	YKR095W	0.7380
YBR253W	YLR071C	0.9928
YBR253W	YML007W	0.7380
YBR253W	YMR112C	0.9992
YBR253W	YNL236W	0.8659
YBR253W	YNR010W	0.9895
YBR253W	YOL051W	0.9886
YBR253W	YOL135C	0.9944
YBR253W	YOR174W	0.9313
YBR253W	YPL042C	0.8659
YBR253W	YPR070W	0.9648
YBR253W	YPR168W	0.9313
YBR254C	YDR108W	0.9624
YBR254C	YDR246W	0.8659
YBR254C	YDR407C	0.9624
YBR254C	YDR472W	0.9970
YBR254C	YEL048C	0.9707
YBR254C	YGR143W	0.7380
YBR254C	YGR166W	0.9624
YBR254C	YKR068C	1.0000
YBR254C	YLR342W	0.7380
YBR254C	YML077W	0.9568
YBR254C	YMR218C	0.8659
YBR254C	YOR115C	0.9965
YBR257W	YDR478W	0.9729
YBR257W	YGR030C	0.9802
YBR257W	YHR062C	0.9992
YBR257W	YIR015W	0.9828
YBR257W	YLR145W	0.9850
YBR257W	YNL221C	0.9984
YBR257W	YNL282W	0.9952

YBR258C	YDR469W	0.9634
YBR258C	YHR119W	0.9996
YBR258C	YKL018W	0.8087
YBR258C	YLR015W	0.9949
YBR258C	YPL138C	0.9285
YBR260C	YGR196C	0.9128
YBR260C	YGR221C	0.9710
YBR260C	YMR032W	0.5774
YBR260C	YPL140C	0.7380
YBR263W	YFR031C-A	0.7380
YBR264C	YER136W	0.9365
YBR264C	YNL044W	0.9099
YBR264C	YNL263C	0.9138
YBR264C	YOR370C	0.6672
YBR267W	YDR101C	1.0000
YBR267W	YGL099W	0.9962
YBR267W	YGR148C	0.9740
YBR267W	YHR170W	0.9624
YBR267W	YIR026C	0.7891
YBR267W	YJL122W	0.8566
YBR267W	YKR048C	0.8028
YBR267W	YLR387C	0.9773
YBR267W	YNL227C	0.9215
YBR267W	YPR016C	0.9215
YBR270C	YIL105C	0.8867
YBR270C	YKR026C	0.7253
YBR270C	YNL047C	0.8258
YBR272C	YDL007W	0.9598
YBR272C	YDL097C	0.9598
YBR272C	YDL147W	0.9215
YBR272C	YDR394W	0.9887
YBR272C	YDR427W	0.9598
YBR272C	YER021W	0.8566
YBR272C	YFR052W	0.8566
YBR272C	YGL004C	0.8566
YBR272C	YGL048C	0.9887
YBR272C	YHR027C	0.9215
YBR272C	YKL145W	1.0000
YBR272C	YOR117W	0.9995
YBR272C	YOR259C	0.9215
YBR272C	YOR261C	0.9884
YBR272C	YOR362C	0.9191
YBR273C	YDL126C	0.9958
YBR273C	YDR411C	0.8179
YBR274W	YDR113C	0.9921
YBR274W	YDR217C	0.8965
YBR274W	YFR028C	0.8566

YBR275C	YNL216W	0.8965
YBR278W	YDR121W	1.0000
YBR278W	YNL262W	1.0000
YBR278W	YPR175W	0.9986
YBR279W	YDL140C	0.8953
YBR279W	YDR138W	0.9780
YBR279W	YGL207W	0.9951
YBR279W	YGL244W	1.0000
YBR279W	YGR005C	0.9555
YBR279W	YGR104C	0.9215
YBR279W	YIL035C	0.7380
YBR279W	YLR418C	1.0000
YBR279W	YML010W	0.8953
YBR279W	YML069W	0.9964
YBR279W	YOL145C	1.0000
YBR279W	YOR061W	0.7380
YBR279W	YOR123C	1.0000
YBR280C	YDL132W	0.9794
YBR280C	YDR328C	0.9663
YBR280C	YNL141W	0.9157
YBR281C	YFR044C	0.9099
YBR281C	YNL191W	0.9852
YBR282W	YCR046C	0.7380
YBR282W	YLR439W	0.8087
YBR282W	YNL284C	0.7380
YBR283C	YGR284C	0.4335
YBR285W	YGR092W	0.6042
YBR288C	YGR261C	0.9931
YBR288C	YPL195W	0.9313
YBR289W	YDR073W	0.9764
YBR289W	YEL009C	0.9938
YBR289W	YFL049W	0.9624
YBR289W	YHL025W	1.0000
YBR289W	YJL176C	0.9985
YBR289W	YKR001C	0.7380
YBR289W	YMR033W	0.9808
YBR289W	YNR023W	0.9624
YBR289W	YOR290C	1.0000
YBR289W	YPL016W	0.9966
YBR289W	YPL129W	0.9739
YBR289W	YPR034W	0.8566
YBR290W	YER125W	0.9640
YBR290W	YLL039C	0.8566
YBR290W	YPL176C	0.9215
YBR290W	YPL240C	0.5774
YCL008C	YGL045W	0.9758
YCL008C	YGR206W	0.9266

YCL008C	YHL002W	0.8640
YCL008C	YLL039C	0.9780
YCL008C	YLR119W	0.9990
YCL008C	YNR006W	0.9912
YCL008C	YPL065W	0.9998
YCL008C	YPL084W	0.9151
YCL010C	YDR145W	0.9648
YCL010C	YDR167W	0.7380
YCL010C	YDR176W	0.9128
YCL010C	YDR392W	0.7380
YCL010C	YDR448W	0.9991
YCL010C	YGL066W	0.8659
YCL010C	YGL112C	0.9908
YCL010C	YGR252W	0.9808
YCL010C	YHR099W	0.7380
YCL010C	YLR055C	0.9021
YCL010C	YMR223W	0.9499
YCL010C	YMR236W	0.7380
YCL010C	YOL148C	0.9648
YCL010C	YPL047W	0.9285
YCL010C	YPL254W	0.9743
YCL011C	YDL014W	0.7380
YCL011C	YDL084W	0.9634
YCL011C	YDR138W	0.9951
YCL011C	YDR381W	0.9743
YCL011C	YDR432W	0.8756
YCL011C	YGL120C	0.7380
YCL011C	YGR162W	0.7380
YCL011C	YHL034C	0.7380
YCL011C	YHR167W	0.9933
YCL011C	YJR145C	0.7380
YCL011C	YKL139W	0.9726
YCL011C	YLR432W	0.8659
YCL011C	YML062C	0.9499
YCL011C	YMR216C	0.9341
YCL011C	YNL004W	0.8603
YCL011C	YNL139C	0.9743
YCL011C	YNL253W	0.9021
YCL011C	YOL139C	0.7380
YCL011C	YPR161C	0.7380
YCL014W	YDR099W	0.7380
YCL014W	YGR220C	0.7380
YCL014W	YPL106C	0.7380
YCL014W	YPL153C	0.8087
YCL014W	YPR119W	0.9034
YCL016C	YHR191C	0.9266
YCL016C	YMR078C	0.8628

YCL017C	YDL120W	0.9191
YCL017C	YER048W-A	0.9999
YCL017C	YPL135W	0.9586
YCL024W	YCR002C	0.8566
YCL024W	YDL006W	0.4335
YCL024W	YDR162C	0.4335
YCL024W	YHR107C	0.8566
YCL024W	YJR076C	0.9903
YCL024W	YKR048C	0.9828
YCL024W	YLR314C	0.8921
YCL024W	YPL031C	0.6387
YCL027W	YER118C	0.9763
YCL027W	YER149C	0.5774
YCL027W	YIL159W	0.8591
YCL027W	YKL105C	0.5774
YCL027W	YKR101W	0.5774
YCL027W	YLR330W	0.9690
YCL027W	YNL271C	0.7909
YCL027W	YPR008W	0.5774
YCL028W	YNL007C	0.9979
YCL028W	YNL064C	0.9215
YCL028W	YPL140C	0.7380
YCL029C	YER016W	0.9999
YCL029C	YJR066W	0.9747
YCL029C	YLR045C	0.9998
YCL029C	YML085C	0.8894
YCL029C	YPL155C	0.8179
YCL029C	YPL269W	0.9758
YCL030C	YFR004W	0.7380
YCL030C	YGR052W	0.9266
YCL031C	YCR057C	0.9215
YCL031C	YGR090W	0.9648
YCL031C	YIL035C	0.8659
YCL032W	YDR103W	0.8965
YCL032W	YER118C	0.9282
YCL032W	YLR362W	1.0000
YCL032W	YOR212W	0.8965
YCL032W	YPR075C	0.9998
YCL034W	YOR181W	0.7909
YCL037C	YDL229W	0.7380
YCL037C	YGL120C	0.8659
YCL037C	YGR162W	0.8659
YCL037C	YHL034C	0.7380
YCL037C	YJL033W	0.7380
YCL037C	YLR175W	0.7380
YCL037C	YLR256W	0.9215
YCL037C	YLR369W	0.7380

YCL037C	YMR125W	0.8659
YCL037C	YNL064C	0.9215
YCL037C	YNR051C	0.6147
YCL039W	YGL227W	0.9243
YCL039W	YMR135C	0.6672
YCL040W	YFR004W	0.7380
YCL044C	YPR024W	0.9965
YCL050C	YDR510W	0.7380
YCL050C	YFR004W	0.7380
YCL051W	YNL161W	0.9221
YCL054W	YDL014W	0.9215
YCL054W	YER006W	0.9021
YCL054W	YER133W	0.7380
YCL054W	YGL111W	0.8087
YCL054W	YGR103W	0.9285
YCL054W	YKR081C	0.7380
YCL054W	YNL110C	0.9313
YCL057W	YNL064C	0.9215
YCL059C	YCR057C	0.8659
YCL059C	YDL014W	0.9313
YCL059C	YDL148C	0.9648
YCL059C	YDL166C	0.7380
YCL059C	YDL213C	0.9313
YCL059C	YDR299W	0.9313
YCL059C	YDR324C	0.8659
YCL059C	YDR449C	0.8659
YCL059C	YER082C	0.9313
YCL059C	YER102W	0.8087
YCL059C	YGR145W	0.9313
YCL059C	YHR148W	0.9313
YCL059C	YHR203C	0.9021
YCL059C	YIL019W	0.8798
YCL059C	YJL109C	0.8659
YCL059C	YJR002W	0.9928
YCL059C	YJR123W	0.8087
YCL059C	YJR145C	0.9499
YCL059C	YKR060W	0.8659
YCL059C	YLL011W	0.9313
YCL059C	YLR175W	0.9648
YCL059C	YLR186W	0.9313
YCL059C	YLR197W	0.9313
YCL059C	YLR340W	0.7380
YCL059C	YLR409C	0.8659
YCL059C	YMR093W	0.8659
YCL059C	YMR128W	0.9313
YCL059C	YMR229C	0.8659
YCL059C	YMR290C	0.7380

YCL059C	YNL132W	0.9313
YCL059C	YNL178W	0.9021
YCL059C	YNL308C	0.9624
YCL059C	YOR096W	0.9021
YCL059C	YPL126W	0.8659
YCL059C	YPL217C	0.9313
YCL059C	YPR102C	0.8087
YCL059C	YPR137W	0.9313
YCL059C	YPR144C	0.9648
YCL061C	YEL032W	0.8953
YCL061C	YLR103C	0.9983
YCL061C	YMR048W	0.9918
YCL061C	YNL262W	0.9919
YCL061C	YNL273W	0.9890
YCL061C	YOR080W	0.9989
YCL061C	YPL153C	0.9859
YCL061C	YPR019W	0.8953
YCL063W	YEL013W	0.9099
YCL063W	YFR021W	0.9782
YCL063W	YLR423C	0.5774
YCL063W	YOR326W	0.9993
YCR002C	YDL225W	0.9996
YCR002C	YDR507C	0.9794
YCR002C	YHR107C	1.0000
YCR002C	YJR076C	1.0000
YCR002C	YJR092W	0.8659
YCR002C	YLR314C	1.0000
YCR002C	YPL153C	0.9464
YCR003W	YER017C	0.9191
YCR004C	YDR032C	0.7837
YCR005C	YLL039C	0.9021
YCR009C	YDR388W	1.0000
YCR009C	YLR429W	0.7380
YCR009C	YMR192W	0.9494
YCR009C	YMR232W	0.9939
YCR009C	YOR181W	0.9470
YCR009C	YPL249C	0.9656
YCR012W	YDR510W	0.8659
YCR012W	YFR004W	0.7380
YCR012W	YHR200W	0.8087
YCR012W	YLL039C	0.7380
YCR015C	YGR163W	0.8566
YCR020W-B	YCR052W	0.7380
YCR020W-B	YDR303C	0.8566
YCR020W-B	YFR037C	0.9884
YCR020W-B	YGR275W	0.9313
YCR020W-B	YIL126W	0.9266

YCR020W-B	YKR008W	0.7380
YCR020W-B	YLR033W	0.8659
YCR020W-B	YLR321C	0.7380
YCR020W-B	YLR357W	0.7380
YCR020W-B	YML127W	0.7380
YCR020W-B	YMR033W	0.7380
YCR020W-B	YMR091C	0.8659
YCR020W-B	YPR034W	0.7380
YCR027C	YOL083W	0.5774
YCR028C-A	YDR097C	0.7380
YCR028C-A	YML032C	0.7380
YCR028C-A	YNR051C	0.7380
YCR028C-A	YPR161C	0.7380
YCR030C	YFR024C-A	0.5107
YCR030C	YGR220C	0.7380
YCR030C	YJR076C	0.7891
YCR030C	YLR332W	0.7983
YCR030C	YOR181W	0.6147
YCR031C	YFR004W	0.7380
YCR033W	YDR155C	0.9928
YCR033W	YGL194C	0.9634
YCR033W	YIL112W	0.9948
YCR033W	YKR029C	0.9973
YCR033W	YMR273C	0.7380
YCR033W	YOL068C	0.9285
YCR034W	YDL015C	0.7891
YCR035C	YDL111C	0.9499
YCR035C	YDR280W	0.9978
YCR035C	YGR095C	1.0000
YCR035C	YGR158C	0.9808
YCR035C	YGR195W	0.9996
YCR035C	YHR034C	0.9099
YCR035C	YHR069C	0.9743
YCR035C	YHR081W	0.9021
YCR035C	YNL189W	0.8659
YCR035C	YNL232W	0.9986
YCR035C	YNR024W	0.9697
YCR035C	YOL021C	1.0000
YCR035C	YOL142W	0.8659
YCR035C	YOR001W	0.9986
YCR035C	YOR076C	0.9499
YCR035C	YPL211W	0.9710
YCR038C	YGR041W	0.9037
YCR038C	YLR293C	0.8998
YCR038C	YLR353W	0.9037
YCR039C	YCR084C	0.9138
YCR039C	YCR097W	1.0000

YCR039C	YDL013W	0.8655
YCR039C	YMR043W	0.9937
YCR042C	YDR145W	0.7380
YCR042C	YDR167W	0.9794
YCR042C	YER148W	1.0000
YCR042C	YGL112C	0.9779
YCR042C	YGR274C	1.0000
YCR042C	YKL081W	0.7380
YCR042C	YML015C	0.9946
YCR042C	YML098W	0.8659
YCR042C	YML114C	0.9908
YCR042C	YMR005W	0.7380
YCR042C	YMR227C	0.7380
YCR042C	YMR236W	0.7380
YCR042C	YPL011C	0.7380
YCR042C	YPL129W	0.9991
YCR046C	YCR071C	0.7380
YCR046C	YDR116C	0.7380
YCR046C	YDR237W	0.7380
YCR046C	YDR296W	0.8659
YCR046C	YDR322W	0.7380
YCR046C	YDR462W	0.7380
YCR046C	YGR091W	0.7380
YCR046C	YGR220C	0.9313
YCR046C	YJL063C	0.7380
YCR046C	YKL167C	0.7380
YCR046C	YKR006C	0.7380
YCR046C	YKR085C	0.7380
YCR046C	YLR312W-A	0.7380
YCR046C	YLR439W	0.7380
YCR046C	YML025C	0.8659
YCR046C	YMR024W	0.7380
YCR046C	YMR193W	0.7380
YCR046C	YNL005C	0.7380
YCR046C	YNL177C	0.7380
YCR046C	YNL252C	0.7380
YCR046C	YNL284C	0.7380
YCR052W	YDR303C	0.9820
YCR052W	YFR037C	0.9998
YCR052W	YGR056W	0.9624
YCR052W	YGR275W	0.9820
YCR052W	YIL126W	0.9999
YCR052W	YKR008W	0.9908
YCR052W	YLR033W	0.9908
YCR052W	YLR321C	0.9902
YCR052W	YLR357W	0.9999
YCR052W	YML127W	0.9313

YCR052W	YMR033W	0.9648
YCR052W	YMR091C	0.9908
YCR052W	YPL082C	0.8953
YCR052W	YPR034W	0.9648
YCR057C	YDL014W	0.9313
YCR057C	YDL148C	0.8659
YCR057C	YDR025W	0.7380
YCR057C	YDR064W	0.8659
YCR057C	YDR299W	0.7380
YCR057C	YDR324C	0.9313
YCR057C	YDR365C	0.7380
YCR057C	YDR382W	0.7380
YCR057C	YDR449C	0.9966
YCR057C	YER082C	0.9908
YCR057C	YGL120C	0.8659
YCR057C	YGL171W	0.7380
YCR057C	YGR090W	0.9908
YCR057C	YGR128C	0.9743
YCR057C	YGR135W	0.7380
YCR057C	YGR145W	0.7380
YCR057C	YHR148W	0.9313
YCR057C	YHR169W	0.7380
YCR057C	YHR196W	0.9820
YCR057C	YJL033W	0.7380
YCR057C	YJL069C	0.9991
YCR057C	YJL109C	0.9820
YCR057C	YJR002W	0.9743
YCR057C	YJR123W	0.7380
YCR057C	YJR145C	0.8659
YCR057C	YKL099C	0.7380
YCR057C	YKR060W	0.9313
YCR057C	YLL011W	0.9313
YCR057C	YLR129W	0.9901
YCR057C	YLR175W	0.7380
YCR057C	YLR186W	0.9313
YCR057C	YLR197W	0.9313
YCR057C	YLR222C	0.9966
YCR057C	YLR367W	0.7380
YCR057C	YLR409C	0.9987
YCR057C	YML130C	0.7380
YCR057C	YMR093W	0.9313
YCR057C	YMR128W	0.9313
YCR057C	YMR143W	0.7380
YCR057C	YMR229C	0.7380
YCR057C	YNL075W	0.9313
YCR057C	YNL132W	0.9313
YCR057C	YNR043W	0.7380

YCR057C	YNR054C	0.8087
YCR057C	YOR078W	0.7380
YCR057C	YOR096W	0.7380
YCR057C	YOR145C	0.7380
YCR057C	YOR310C	0.9869
YCR057C	YPL012W	0.7380
YCR057C	YPL081W	0.7380
YCR057C	YPL126W	0.9895
YCR057C	YPL217C	0.9313
YCR057C	YPL266W	0.8659
YCR057C	YPR137W	0.9820
YCR057C	YPR144C	0.9908
YCR059C	YFL039C	0.8953
YCR060W	YDR190C	0.7380
YCR060W	YHR034C	0.9988
YCR060W	YMR186W	0.8998
YCR060W	YOR310C	0.8353
YCR060W	YPL235W	0.8659
YCR060W	YPL240C	0.9988
YCR063W	YDL209C	0.7380
YCR063W	YER172C	0.8087
YCR063W	YHR165C	0.9499
YCR063W	YLL036C	0.9844
YCR063W	YMR213W	0.9313
YCR063W	YPL151C	0.7380
YCR063W	YPR101W	0.7380
YCR065W	YDL042C	0.9215
YCR065W	YPL106C	0.7380
YCR066W	YDL064W	0.9099
YCR066W	YDR092W	0.9507
YCR066W	YGL058W	1.0000
YCR066W	YJL092W	0.8965
YCR066W	YLR032W	0.9970
YCR066W	YNL312W	0.9723
YCR067C	YPL085W	0.9099
YCR071C	YDR116C	0.7380
YCR071C	YDR296W	0.7380
YCR071C	YDR322W	0.7380
YCR071C	YDR405W	0.7380
YCR071C	YGR220C	0.8659
YCR071C	YJL063C	0.7380
YCR071C	YLR439W	0.7380
YCR071C	YMR024W	0.7380
YCR071C	YNL284C	0.8659
YCR072C	YDR101C	0.9624
YCR072C	YER006W	0.9499
YCR072C	YER126C	0.8659

YCR072C	YGR245C	0.9313
YCR072C	YHR197W	0.9990
YCR072C	YKL009W	0.9598
YCR072C	YLR074C	0.8659
YCR072C	YLR106C	0.9961
YCR072C	YNL182C	0.9808
YCR072C	YNR053C	0.9266
YCR072C	YPL093W	0.8087
YCR072C	YPR016C	0.8087
YCR073C	YJL128C	0.9539
YCR073C	YLR006C	0.8297
YCR073W-A	YDR247W	0.6042
YCR073W-A	YER133W	0.9313
YCR073W-A	YLR028C	0.7380
YCR076C	YER094C	0.7380
YCR077C	YDL139C	0.5774
YCR077C	YDL160C	0.9972
YCR077C	YDR378C	0.9875
YCR077C	YER112W	0.9952
YCR077C	YER146W	0.9995
YCR077C	YER165W	0.8566
YCR077C	YGL173C	0.9906
YCR077C	YJL124C	1.0000
YCR077C	YJL140W	0.9099
YCR077C	YJR022W	0.5774
YCR077C	YLR438C-A	0.9909
YCR077C	YNL118C	0.9812
YCR077C	YNL145W	0.3670
YCR077C	YNL147W	0.9936
YCR077C	YOL149W	0.9138
YCR077C	YPL240C	0.6672
YCR079W	YOR323C	0.9648
YCR081W	YDR308C	0.7380
YCR081W	YDR443C	0.9313
YCR081W	YER022W	0.8087
YCR081W	YGL025C	0.9186
YCR081W	YGL127C	0.7380
YCR081W	YGL151W	0.7380
YCR081W	YGR104C	0.9800
YCR081W	YLR071C	0.7380
YCR081W	YNL025C	0.9266
YCR081W	YNL236W	0.7380
YCR081W	YOL135C	0.8659
YCR081W	YPL042C	0.7380
YCR081W	YPR070W	0.8659
YCR082W	YGR252W	0.7380
YCR082W	YOR023C	0.7837

YCR084C	YDR308C	0.9758
YCR084C	YDR510W	0.9313
YCR084C	YGL025C	0.9249
YCR084C	YKL181W	0.9187
YCR084C	YLR176C	0.9282
YCR084C	YNL167C	0.9215
YCR084C	YNL330C	0.9215
YCR084C	YOL004W	0.7380
YCR084C	YPL042C	0.9747
YCR084C	YPL228W	0.9249
YCR086W	YDL089W	0.9891
YCR086W	YDR439W	1.0000
YCR086W	YER106W	0.9950
YCR086W	YFR028C	0.8756
YCR086W	YHR152W	0.5774
YCR086W	YIR010W	0.9512
YCR086W	YKR010C	0.9980
YCR086W	YLR086W	0.8965
YCR086W	YML034W	0.9478
YCR086W	YOR264W	0.8214
YCR086W	YOR281C	0.7909
YCR088W	YDR129C	0.8240
YCR088W	YDR388W	0.7909
YCR088W	YFL039C	0.9492
YCR088W	YFR004W	0.7380
YCR088W	YHR016C	0.6672
YCR088W	YIL095W	0.9943
YCR088W	YIR003W	0.9066
YCR088W	YJR065C	0.8521
YCR088W	YNL020C	0.9978
YCR088W	YNL094W	0.9956
YCR088W	YNL106C	0.9930
YCR088W	YNL138W	0.9997
YCR088W	YOR181W	0.8566
YCR088W	YOR284W	0.7253
YCR088W	YOR367W	0.9841
YCR092C	YDR097C	0.7380
YCR092C	YOL090W	0.9939
YCR093W	YDL160C	0.7891
YCR093W	YDL165W	0.9999
YCR093W	YER068W	1.0000
YCR093W	YGR134W	0.9994
YCR093W	YGR274C	0.9960
YCR093W	YHR206W	0.9099
YCR093W	YIL038C	0.9952
YCR093W	YNL288W	1.0000
YCR093W	YNR052C	1.0000

YCR093W	YPL235W	0.8659
YCR093W	YPR072W	0.9999
YCR095C	YHL029C	0.9483
YCR095C	YNL032W	0.8659
YCR095C	YNL099C	0.8297
YDL002C	YDR190C	0.9499
YDL002C	YER092W	0.9915
YDL002C	YFL013C	0.9869
YDL002C	YFL039C	0.9499
YDL002C	YGL133W	0.7380
YDL002C	YGL150C	0.9999
YDL002C	YJL081C	0.9904
YDL002C	YKR001C	0.7380
YDL002C	YLR052W	0.9674
YDL002C	YLR357W	0.7380
YDL002C	YNL059C	0.7380
YDL002C	YOR141C	0.9904
YDL002C	YOR304W	0.7380
YDL002C	YPL129W	0.7380
YDL002C	YPL235W	0.9743
YDL003W	YFL008W	1.0000
YDL003W	YFR027W	0.7696
YDL003W	YGR098C	0.8655
YDL003W	YIL026C	1.0000
YDL003W	YIL126W	0.9215
YDL003W	YJL074C	1.0000
YDL003W	YMR001C	0.9904
YDL003W	YMR076C	0.9935
YDL004W	YJR121W	0.9960
YDL005C	YDR308C	0.7380
YDL005C	YDR443C	0.7380
YDL005C	YER022W	0.9961
YDL005C	YGL025C	0.9999
YDL005C	YGL127C	0.9266
YDL005C	YGL151W	0.8659
YDL005C	YGR104C	0.9998
YDL005C	YHR041C	0.8659
YDL005C	YHR058C	0.7380
YDL005C	YLR071C	0.9923
YDL005C	YMR112C	0.7380
YDL005C	YNL236W	0.8659
YDL005C	YNR010W	0.9942
YDL005C	YOL051W	0.9624
YDL005C	YOL135C	0.9991
YDL005C	YOR174W	0.9995
YDL005C	YPL042C	0.9341
YDL005C	YPR070W	0.8659

YDL005C	YPR168W	0.8659
YDL006W	YDR162C	0.9984
YDL006W	YDR507C	0.4335
YDL006W	YJL095W	0.7100
YDL006W	YJL128C	0.9584
YDL006W	YNL298W	0.3794
YDL006W	YOL113W	0.7380
YDL007W	YDL097C	0.9313
YDL007W	YDL147W	0.9648
YDL007W	YDR363W-A	0.9021
YDL007W	YDR394W	0.9938
YDL007W	YDR427W	0.8659
YDL007W	YEL037C	0.7380
YDL007W	YER021W	0.7380
YDL007W	YFR004W	0.9808
YDL007W	YFR010W	0.9313
YDL007W	YFR052W	0.9313
YDL007W	YGL004C	0.8566
YDL007W	YGL048C	0.8990
YDL007W	YGR232W	0.9464
YDL007W	YHL030W	0.7380
YDL007W	YHR027C	0.9999
YDL007W	YHR200W	0.9743
YDL007W	YIL075C	0.7380
YDL007W	YKL145W	0.9985
YDL007W	YLL039C	0.7380
YDL007W	YLR421C	0.7380
YDL007W	YOR117W	0.8028
YDL007W	YOR259C	0.7380
YDL007W	YOR261C	0.7380
YDL007W	YPR108W	0.9266
YDL008W	YDR118W	0.9854
YDL008W	YDR260C	0.9478
YDL008W	YKL022C	1.0000
YDL008W	YLR127C	0.9746
YDL008W	YNL172W	0.8603
YDL013W	YDL042C	0.9138
YDL013W	YDR510W	0.9988
YDL013W	YER116C	1.0000
YDL013W	YGR140W	0.7909
YDL013W	YHR134W	0.8965
YDL013W	YML032C	0.8655
YDL014W	YDL148C	0.8659
YDL014W	YDL208W	0.9266
YDL014W	YDR060W	0.9313
YDL014W	YDR365C	0.8087
YDL014W	YDR449C	0.7380

YDL014W	YDR496C	0.7380
YDL014W	YEL026W	0.9667
YDL014W	YER082C	0.9313
YDL014W	YFR004W	0.7380
YDL014W	YGL078C	0.7380
YDL014W	YGL120C	0.9743
YDL014W	YGR090W	0.9313
YDL014W	YGR103W	0.9266
YDL014W	YHR052W	0.8659
YDL014W	YHR089C	0.8566
YDL014W	YHR148W	0.8566
YDL014W	YHR196W	0.7380
YDL014W	YJL033W	0.9648
YDL014W	YJL109C	0.8659
YDL014W	YJR002W	0.9464
YDL014W	YLL011W	0.8566
YDL014W	YLR175W	0.9808
YDL014W	YLR197W	0.9993
YDL014W	YMR229C	0.7380
YDL014W	YMR308C	0.9037
YDL014W	YNL061W	0.9313
YDL014W	YNL308C	0.7380
YDL014W	YNR054C	0.8087
YDL014W	YOR206W	0.8659
YDL014W	YOR310C	0.9997
YDL014W	YPL126W	0.8566
YDL014W	YPR016C	0.7380
YDL014W	YPR137W	0.9539
YDL014W	YPR144C	0.8659
YDL015C	YHR195W	0.9730
YDL015C	YLR372W	0.7891
YDL017W	YDL132W	0.7909
YDL017W	YDR052C	1.0000
YDL017W	YEL032W	0.7696
YDL017W	YGL201C	0.9469
YDL017W	YMR001C	0.9890
YDL017W	YPL153C	0.9961
YDL017W	YPR019W	0.9858
YDL018C	YHR110W	0.8297
YDL018C	YML012W	0.8659
YDL019C	YER120W	0.7380
YDL019C	YGR092W	0.6042
YDL019C	YMR109W	0.7495
YDL020C	YFR052W	0.9215
YDL020C	YHR200W	0.9773
YDL020C	YLR024C	0.9972
YDL020C	YMR100W	0.7696

YDL022W	YFR004W	0.7380
YDL028C	YDR162C	0.6672
YDL028C	YDR356W	0.7543
YDL028C	YGL093W	0.7380
YDL028C	YIL106W	0.9963
YDL028C	YIL144W	0.9523
YDL028C	YIR010W	0.7380
YDL028C	YKL042W	0.9312
YDL028C	YMR117C	0.8566
YDL028C	YOL069W	0.9266
YDL028C	YOR257W	0.7380
YDL028C	YPL124W	0.7543
YDL028C	YPL204W	0.8566
YDL029W	YFR004W	0.7380
YDL029W	YIL062C	0.9999
YDL029W	YIL095W	0.9758
YDL029W	YIR006C	0.9249
YDL029W	YJL008C	0.7380
YDL029W	YJR064W	0.7380
YDL029W	YJR065C	1.0000
YDL029W	YKL013C	0.9985
YDL029W	YLL013C	0.8566
YDL029W	YLR370C	1.0000
YDL029W	YLR429W	0.9191
YDL029W	YMR109W	0.9409
YDL029W	YNL189W	0.9215
YDL029W	YNR035C	0.9997
YDL029W	YOR117W	0.8353
YDL029W	YOR181W	0.9915
YDL030W	YDL043C	0.9930
YDL030W	YER029C	0.7380
YDL030W	YER172C	0.7380
YDL030W	YGR074W	0.8659
YDL030W	YHR165C	0.8659
YDL030W	YJL203W	1.0000
YDL030W	YLL036C	0.9787
YDL030W	YLR147C	0.7380
YDL030W	YML049C	0.6672
YDL030W	YMR125W	0.7380
YDL030W	YMR240C	0.8659
YDL030W	YPL213W	0.9021
YDL031W	YDR087C	0.8087
YDL031W	YDR496C	0.7380
YDL031W	YER006W	0.9813
YDL031W	YFL002C	0.8566
YDL031W	YFR001W	0.7380
YDL031W	YGL111W	0.8087

YDL031W	YGR103W	0.9313
YDL031W	YHR052W	0.8659
YDL031W	YKL009W	0.8566
YDL031W	YKR081C	0.8659
YDL031W	YMR290C	0.8566
YDL031W	YNL061W	0.8659
YDL031W	YNL110C	0.9313
YDL031W	YOL077C	0.7380
YDL031W	YOR206W	0.7380
YDL031W	YOR272W	0.7380
YDL031W	YPL106C	0.7380
YDL031W	YPR016C	0.9499
YDL035C	YER020W	0.9970
YDL040C	YFR004W	0.7380
YDL040C	YHR013C	0.9996
YDL040C	YMR116C	0.7380
YDL040C	YOR253W	0.8566
YDL040C	YPL198W	0.7380
YDL042C	YDR110W	0.9747
YDL042C	YDR224C	0.8953
YDL042C	YDR227W	1.0000
YDL042C	YDR363W	0.9667
YDL042C	YDR440W	0.8818
YDL042C	YDR448W	0.9215
YDL042C	YEL032W	0.9099
YDL042C	YFR028C	0.9285
YDL042C	YIL150C	0.9907
YDL042C	YJL076W	1.0000
YDL042C	YKR010C	0.9285
YDL042C	YKR101W	0.9558
YDL042C	YLR442C	1.0000
YDL042C	YMR307W	0.9138
YDL042C	YNL030W	0.9778
YDL042C	YNL031C	0.9940
YDL042C	YOL017W	0.9710
YDL043C	YDR381W	0.7380
YDL043C	YER029C	0.8659
YDL043C	YGL049C	0.8258
YDL043C	YGR162W	0.9108
YDL043C	YHR086W	0.7380
YDL043C	YJL203W	0.9999
YDL043C	YLL036C	0.9186
YDL043C	YLR147C	0.7380
YDL043C	YLR275W	0.7380
YDL043C	YML049C	0.7380
YDL043C	YMR117C	0.5774
YDL043C	YMR240C	0.9313

YDL043C	YMR288W	0.7380
YDL043C	YPL213W	0.8087
YDL044C	YFL036W	0.8798
YDL044C	YLR139C	0.9313
YDL044C	YLR386W	0.5774
YDL045W-A	YIL093C	0.7380
YDL045W-A	YJR113C	0.7380
YDL047W	YDR188W	0.7380
YDL047W	YER155C	0.7380
YDL047W	YFR019W	0.7380
YDL047W	YFR040W	1.0000
YDL047W	YGL197W	0.9266
YDL047W	YGR161C	0.8659
YDL047W	YIL142W	0.8659
YDL047W	YIL153W	0.9995
YDL047W	YJL098W	1.0000
YDL047W	YKR028W	0.9993
YDL047W	YLR310C	0.7380
YDL047W	YLR384C	0.9391
YDL047W	YMR024W	0.7380
YDL047W	YMR028W	1.0000
YDL047W	YNR016C	0.9648
YDL047W	YOR267C	0.7380
YDL047W	YPL152W	0.9215
YDL051W	YDL092W	0.8659
YDL051W	YDR395W	0.9726
YDL051W	YGR162W	0.8659
YDL051W	YLR074C	0.7380
YDL051W	YOL139C	0.7380
YDL051W	YPL210C	0.8659
YDL051W	YPL243W	0.9313
YDL051W	YPR016C	0.7380
YDL051W	YPR088C	0.8659
YDL053C	YGR178C	0.9218
YDL053C	YHR121W	0.9634
YDL053C	YNL157W	0.8566
YDL055C	YDR510W	0.7837
YDL055C	YER086W	0.7380
YDL055C	YFR004W	0.7380
YDL055C	YGL137W	0.7380
YDL055C	YGL245W	0.7380
YDL055C	YHR027C	0.7380
YDL055C	YHR200W	0.8087
YDL055C	YJL074C	0.7380
YDL055C	YJR109C	0.7380
YDL055C	YML124C	0.7380
YDL055C	YMR229C	0.7380

YDL055C	YNR016C	0.7380
YDL055C	YOL098C	0.7380
YDL055C	YOL120C	0.7380
YDL055C	YPL106C	0.7380
YDL055C	YPL235W	0.7380
YDL056W	YHR206W	0.9138
YDL056W	YLR182W	0.9997
YDL056W	YNR009W	0.8953
YDL056W	YOR066W	0.9707
YDL058W	YPL106C	0.7380
YDL059C	YML032C	1.0000
YDL060W	YDR064W	0.7380
YDL060W	YDR447C	0.7380
YDL060W	YGR081C	0.9313
YDL060W	YIL069C	0.7380
YDL060W	YJR145C	0.7380
YDL060W	YKL143W	0.8659
YDL060W	YNL207W	0.9996
YDL060W	YOR056C	0.9813
YDL060W	YOR096W	0.7380
YDL060W	YOR145C	0.9021
YDL060W	YPL012W	0.9499
YDL060W	YPL204W	0.9499
YDL060W	YPL266W	0.9808
YDL064W	YDR409W	0.9998
YDL064W	YDR510W	0.9999
YDL064W	YGR140W	0.7909
YDL064W	YOR156C	0.9138
YDL064W	YPL269W	0.9769
YDL065C	YDR329C	0.9973
YDL065C	YGL153W	0.7718
YDL065C	YLR191W	0.9266
YDL065C	YMR163C	0.7909
YDL065C	YNL214W	0.9727
YDL065C	YOL147C	0.9924
YDL065C	YPL112C	0.7579
YDL066W	YER151C	0.7380
YDL066W	YFR004W	0.7380
YDL070W	YER133W	0.5774
YDL070W	YLR399C	0.9488
YDL072C	YKL065C	0.9215
YDL072C	YOR254C	0.9215
YDL073W	YER118C	0.5774
YDL074C	YGL058W	0.9915
YDL074C	YKL018W	0.9282
YDL074C	YOL069W	0.5774
YDL074C	YPL055C	0.9313

YDL075W	YDR101C	0.7380
YDL075W	YGR285C	0.8953
YDL075W	YNL110C	0.7380
YDL076C	YDR207C	0.9021
YDL076C	YIL084C	0.9313
YDL076C	YLR098C	0.5774
YDL076C	YMR263W	0.9313
YDL076C	YNL330C	0.9964
YDL076C	YOL004W	0.9933
YDL076C	YPL139C	0.9313
YDL076C	YPL181W	0.8659
YDL077C	YDR080W	1.0000
YDL077C	YGL124C	0.8965
YDL077C	YGL212W	0.9967
YDL077C	YLR148W	0.9936
YDL077C	YLR396C	1.0000
YDL077C	YML001W	0.9998
YDL077C	YMR197C	0.9555
YDL077C	YMR231W	0.9997
YDL077C	YOR106W	0.9555
YDL077C	YPL045W	0.9991
YDL078C	YDR244W	0.9954
YDL081C	YDR382W	0.9539
YDL081C	YLR340W	0.9877
YDL082W	YFR031C-A	0.7380
YDL082W	YNL110C	0.7380
YDL082W	YOL077C	0.7380
YDL082W	YOR272W	0.7380
YDL083C	YFR004W	0.7380
YDL083C	YHR200W	0.8087
YDL083C	YKL082C	0.8965
YDL084W	YDR138W	1.0000
YDL084W	YDR159W	0.8566
YDL084W	YDR381W	0.9999
YDL084W	YDR432W	0.7570
YDL084W	YDR510W	0.8297
YDL084W	YER063W	0.5774
YDL084W	YFR004W	0.7380
YDL084W	YGL122C	0.8279
YDL084W	YHR167W	0.9860
YDL084W	YKL214C	0.9818
YDL084W	YML062C	0.9743
YDL084W	YNL004W	0.9285
YDL084W	YNL139C	0.9976
YDL084W	YNL253W	0.8953
YDL087C	YDR235W	0.9313
YDL087C	YDR240C	0.9313

YDL087C	YER029C	0.9313
YDL087C	YGR013W	0.9648
YDL087C	YGR074W	0.7380
YDL087C	YGR162W	0.7380
YDL087C	YHR086W	0.9869
YDL087C	YHR165C	0.7380
YDL087C	YIL061C	0.9820
YDL087C	YKL012W	0.9991
YDL087C	YKL173W	0.8659
YDL087C	YLR147C	0.9313
YDL087C	YLR275W	0.8659
YDL087C	YLR298C	0.9820
YDL087C	YML046W	0.8659
YDL087C	YML049C	0.8659
YDL087C	YMR125W	0.9820
YDL087C	YMR288W	0.7380
YDL087C	YOL139C	0.7380
YDL087C	YPL178W	0.8659
YDL087C	YPR182W	0.8659
YDL088C	YML031W	0.9510
YDL088C	YMR153W	0.8179
YDL089W	YDR439W	0.8603
YDL089W	YLR324W	0.5774
YDL089W	YML034W	0.8603
YDL091C	YDL126C	0.9099
YDL092W	YKL122C	0.8297
YDL092W	YML105C	0.9983
YDL092W	YPL210C	0.9483
YDL092W	YPL243W	0.9313
YDL092W	YPR088C	0.9313
YDL095W	YOL013C	0.8953
YDL097C	YDL147W	1.0000
YDL097C	YDR363W-A	0.9021
YDL097C	YDR394W	0.9648
YDL097C	YDR427W	0.9999
YDL097C	YER021W	0.9983
YDL097C	YFR004W	1.0000
YDL097C	YFR010W	0.9313
YDL097C	YFR052W	0.9995
YDL097C	YGL004C	0.8566
YDL097C	YGL048C	0.9820
YDL097C	YGR232W	0.9726
YDL097C	YHL030W	0.8659
YDL097C	YHR027C	0.9995
YDL097C	YHR200W	0.9999
YDL097C	YIL075C	0.9787
YDL097C	YKL145W	0.9820

YDL097C	YLR421C	0.9313
YDL097C	YOR117W	0.9313
YDL097C	YOR259C	0.9313
YDL097C	YOR261C	0.9992
YDL097C	YPR108W	0.9999
YDL098C	YDR473C	0.7380
YDL098C	YER029C	0.7380
YDL098C	YER172C	0.9993
YDL098C	YGR075C	0.8297
YDL098C	YKL173W	0.7380
YDL098C	YNL147W	0.8659
YDL098C	YPR178W	0.8659
YDL099W	YDR517W	0.9266
YDL100C	YER083C	0.9813
YDL100C	YFR004W	0.7380
YDL100C	YGL020C	0.9725
YDL100C	YHR005C	0.9640
YDL100C	YHR057C	0.5774
YDL100C	YJR040W	0.9747
YDL100C	YLR268W	0.9099
YDL100C	YML101C	0.5774
YDL100C	YOL018C	0.7909
YDL100C	YOL111C	0.9941
YDL100C	YOL126C	0.5774
YDL100C	YOR164C	0.9962
YDL101C	YML058W	0.9941
YDL101C	YPL153C	0.9992
YDL102W	YJR006W	0.9983
YDL102W	YJR043C	0.9950
YDL103C	YDL229W	0.7380
YDL103C	YPL106C	0.7380
YDL105W	YDR288W	0.9997
YDL105W	YLR007W	0.9835
YDL105W	YOL034W	0.9908
YDL106C	YDR146C	0.9996
YDL106C	YFR034C	1.0000
YDL106C	YKR099W	0.9970
YDL108W	YDL125C	0.9099
YDL108W	YDL140C	0.9994
YDL108W	YDR460W	0.9987
YDL108W	YER171W	0.9871
YDL108W	YFL029C	0.7696
YDL108W	YGL134W	0.5774
YDL108W	YLR005W	0.7380
YDL108W	YOR174W	0.8655
YDL108W	YPR025C	0.9993
YDL108W	YPR056W	0.8566

YDL111C	YDR280W	0.9951
YDL111C	YGR095C	0.9996
YDL111C	YGR158C	0.9995
YDL111C	YGR195W	0.9997
YDL111C	YHR069C	0.9904
YDL111C	YNL189W	0.7380
YDL111C	YNL232W	0.9995
YDL111C	YNR024W	0.8087
YDL111C	YOL021C	1.0000
YDL111C	YOL142W	0.9499
YDL111C	YOR001W	0.9964
YDL111C	YOR076C	0.9813
YDL111C	YPR189W	0.7380
YDL112W	YKL182W	0.7380
YDL113C	YGR172C	0.9138
YDL113C	YJL036W	0.9998
YDL113C	YPR049C	0.9099
YDL115C	YDL140C	0.8659
YDL115C	YDR404C	0.8659
YDL115C	YGL070C	0.8659
YDL115C	YIL021W	0.9869
YDL115C	YJL140W	0.8659
YDL115C	YOL005C	0.9499
YDL115C	YOR151C	0.9313
YDL115C	YOR210W	0.8659
YDL115C	YOR224C	0.7380
YDL115C	YPR187W	0.7380
YDL116W	YER105C	0.9431
YDL116W	YER116C	0.9215
YDL116W	YFR002W	0.8087
YDL116W	YGL092W	1.0000
YDL116W	YGL100W	1.0000
YDL116W	YGL172W	0.9941
YDL116W	YGR119C	0.8087
YDL116W	YJR042W	1.0000
YDL116W	YKL057C	1.0000
YDL116W	YKL068W	0.8087
YDL116W	YKR082W	0.9999
YDL116W	YLR208W	1.0000
YDL116W	YMR047C	0.9696
YDL116W	YPL169C	0.9624
YDL117W	YER177W	0.7380
YDL117W	YMR032W	0.5774
YDL117W	YNL152W	0.9811
YDL120W	YER048W-A	0.9215
YDL120W	YGR234W	0.9099
YDL120W	YOR176W	0.9764

YDL120W	YPL135W	1.0000
YDL122W	YKR094C	0.7696
YDL122W	YLR167W	0.7696
YDL123W	YER125W	0.9899
YDL123W	YLL039C	0.8566
YDL126C	YDL190C	1.0000
YDL126C	YDR028C	0.7380
YDL126C	YDR049W	0.9648
YDL126C	YDR330W	0.9539
YDL126C	YDR337W	0.7380
YDL126C	YDR411C	0.9707
YDL126C	YDR510W	0.9624
YDL126C	YEL037C	0.9747
YDL126C	YER151C	0.9598
YDL126C	YFL044C	0.9750
YDL126C	YFR004W	0.7380
YDL126C	YGL108C	0.6672
YDL126C	YGL246C	0.7380
YDL126C	YGR048W	1.0000
YDL126C	YJL048C	0.9710
YDL126C	YKL020C	0.9780
YDL126C	YKL213C	1.0000
YDL126C	YLL039C	0.9976
YDL126C	YLR450W	0.9215
YDL126C	YML013W	0.9996
YDL126C	YMR067C	0.9973
YDL126C	YNL155W	0.6672
YDL126C	YNL271C	0.7380
YDL126C	YNR051C	0.9598
YDL126C	YOL013C	0.9958
YDL126C	YPL256C	0.6996
YDL127W	YDR146C	0.9843
YDL127W	YDR388W	0.8965
YDL127W	YPL031C	0.9978
YDL130W	YLR340W	0.9414
YDL131W	YDL182W	0.8028
YDL131W	YFR004W	0.7380
YDL132W	YDR054C	1.0000
YDL132W	YDR131C	0.8998
YDL132W	YDR139C	0.9992
YDL132W	YDR306C	0.8628
YDL132W	YDR328C	1.0000
YDL132W	YFL009W	1.0000
YDL132W	YGL249W	0.7909
YDL132W	YIL046W	0.9996
YDL132W	YJL149W	0.9856
YDL132W	YJR090C	1.0000

YDL132W	YLR097C	0.9856
YDL132W	YLR100W	0.7909
YDL132W	YLR128W	1.0000
YDL132W	YLR352W	0.9021
YDL132W	YLR368W	0.9512
YDL132W	YML088W	0.9954
YDL132W	YOL025W	0.9968
YDL132W	YOL133W	1.0000
YDL132W	YOR080W	0.9616
YDL132W	YPL256C	0.8566
YDL134C	YDL188C	0.7380
YDL134C	YGL190C	0.9986
YDL134C	YGR161C	0.8659
YDL134C	YKL203C	0.9186
YDL134C	YML109W	0.9157
YDL134C	YMR028W	1.0000
YDL134C	YMR273C	0.7380
YDL134C	YOR014W	0.9946
YDL134C	YPL152W	0.9249
YDL134C	YPR040W	0.9414
YDL135C	YLR229C	0.9849
YDL136W	YHR013C	0.9215
YDL136W	YHR197W	0.6996
YDL136W	YIL133C	0.8566
YDL136W	YNL069C	0.8566
YDL137W	YDL192W	0.6147
YDL137W	YDR358W	0.9710
YDL137W	YHR108W	0.9710
YDL139C	YKL049C	0.9987
YDL140C	YDR059C	0.8655
YDL140C	YDR228C	0.9990
YDL140C	YDR289C	0.9994
YDL140C	YDR293C	0.8998
YDL140C	YDR301W	0.9922
YDL140C	YDR404C	1.0000
YDL140C	YDR432W	0.9707
YDL140C	YDR510W	0.8566
YDL140C	YER022W	0.9997
YDL140C	YER125W	1.0000
YDL140C	YER139C	0.9868
YDL140C	YER148W	0.9911
YDL140C	YER151C	0.9786
YDL140C	YGL043W	0.9021
YDL140C	YGL044C	0.9099
YDL140C	YGL058W	0.9215
YDL140C	YGL070C	1.0000
YDL140C	YGL130W	0.9980

YDL140C	YGR005C	0.9996
YDL140C	YGR047C	0.9249
YDL140C	YGR063C	0.9634
YDL140C	YGR104C	1.0000
YDL140C	YGR116W	0.8087
YDL140C	YGR186W	0.9985
YDL140C	YGR200C	0.9875
YDL140C	YHR058C	0.9786
YDL140C	YIL021W	1.0000
YDL140C	YJL140W	1.0000
YDL140C	YJL168C	0.9999
YDL140C	YJR017C	1.0000
YDL140C	YKL054C	0.8655
YDL140C	YKL110C	0.9773
YDL140C	YKL130C	0.9215
YDL140C	YKL139W	0.7696
YDL140C	YLL039C	0.7380
YDL140C	YLR015W	0.9215
YDL140C	YLR071C	0.9987
YDL140C	YLR113W	0.9944
YDL140C	YLR115W	0.9138
YDL140C	YLR384C	0.9927
YDL140C	YLR418C	0.9936
YDL140C	YLR430W	0.9099
YDL140C	YML010W	0.9999
YDL140C	YMR061W	0.9932
YDL140C	YMR125W	0.8818
YDL140C	YMR277W	0.9571
YDL140C	YNL251C	0.9970
YDL140C	YOL005C	0.9998
YDL140C	YOL051W	0.9881
YDL140C	YOR151C	1.0000
YDL140C	YOR210W	0.9926
YDL140C	YOR224C	1.0000
YDL140C	YPL042C	0.8894
YDL140C	YPL129W	0.9951
YDL140C	YPL204W	0.8998
YDL140C	YPL228W	0.9282
YDL140C	YPR086W	0.9982
YDL140C	YPR093C	0.9953
YDL140C	YPR133C	0.9707
YDL140C	YPR161C	0.9917
YDL140C	YPR187W	1.0000
YDL143W	YDR188W	0.8659
YDL143W	YFR004W	0.7380
YDL143W	YIL142W	0.7380
YDL143W	YJL014W	0.9772

YDL143W	YKL029C	0.7380
YDL143W	YKR026C	0.7380
YDL143W	YOR281C	0.7380
YDL145C	YDR238C	0.9820
YDL145C	YER122C	0.7380
YDL145C	YFL048C	0.9780
YDL145C	YFR004W	0.7380
YDL145C	YFR051C	0.9908
YDL145C	YGL137W	0.9820
YDL145C	YIL076W	0.9820
YDL145C	YNL258C	0.9215
YDL145C	YNL284C	0.7380
YDL145C	YNL287W	0.9648
YDL145C	YPL010W	0.9313
YDL146W	YFR024C-A	0.7909
YDL146W	YHR016C	0.7909
YDL146W	YHR114W	0.9619
YDL147W	YDL216C	0.9313
YDL147W	YDR179C	0.9910
YDL147W	YDR363W-A	0.9726
YDL147W	YDR394W	0.9648
YDL147W	YDR427W	0.9998
YDL147W	YER012W	0.9726
YDL147W	YER021W	0.9808
YDL147W	YFR004W	1.0000
YDL147W	YFR010W	0.9313
YDL147W	YFR052W	0.9902
YDL147W	YGL004C	0.8566
YDL147W	YGL011C	0.7380
YDL147W	YGL048C	0.9648
YDL147W	YGR232W	0.9726
YDL147W	YHL030W	0.8659
YDL147W	YHR027C	0.9971
YDL147W	YHR200W	0.9869
YDL147W	YIL071C	0.8659
YDL147W	YIL075C	0.8659
YDL147W	YJL001W	0.7380
YDL147W	YKL145W	0.9820
YDL147W	YLR421C	0.9313
YDL147W	YMR025W	0.8353
YDL147W	YMR314W	0.7380
YDL147W	YOL117W	0.7380
YDL147W	YOR117W	0.9808
YDL147W	YOR259C	0.9313
YDL147W	YOR261C	0.9997
YDL147W	YPR103W	0.7380
YDL147W	YPR108W	0.9972

YDL148C	YDR299W	0.8659
YDL148C	YER082C	0.7380
YDL148C	YGR090W	0.9820
YDL148C	YGR128C	0.9869
YDL148C	YHR196W	0.7380
YDL148C	YIL069C	0.8659
YDL148C	YJR002W	0.9499
YDL148C	YJR145C	0.8659
YDL148C	YLR186W	0.9966
YDL148C	YLR222C	0.7380
YDL148C	YLR441C	0.7380
YDL148C	YML024W	0.7380
YDL148C	YMR128W	0.8659
YDL148C	YMR229C	0.8659
YDL148C	YNL132W	0.8659
YDL148C	YNL308C	0.8659
YDL148C	YOL010W	0.8659
YDL148C	YOR096W	0.8659
YDL148C	YOR310C	0.9499
YDL148C	YPL012W	0.8659
YDL148C	YPL126W	0.8659
YDL148C	YPL217C	0.8659
YDL148C	YPR137W	0.7380
YDL148C	YPR144C	0.9996
YDL149W	YFR021W	0.9099
YDL149W	YJL178C	0.9968
YDL149W	YLR431C	0.9997
YDL149W	YPR041W	0.7380
YDL149W	YPR049C	0.9710
YDL150W	YDR045C	0.7380
YDL150W	YDR510W	0.8659
YDL150W	YJL011C	0.8659
YDL150W	YKL144C	0.9820
YDL150W	YKR025W	0.9719
YDL150W	YNL113W	0.8659
YDL150W	YNL151C	0.8659
YDL150W	YNR003C	0.9648
YDL150W	YOR116C	0.9313
YDL150W	YOR207C	0.9904
YDL150W	YOR210W	0.7380
YDL150W	YOR224C	0.8659
YDL150W	YPR110C	0.9648
YDL150W	YPR187W	0.8659
YDL150W	YPR190C	0.9313
YDL153C	YDR449C	0.8965
YDL153C	YIL091C	0.9952
YDL153C	YJR002W	0.9613

YDL155W	YFR028C	0.8566
YDL155W	YGL003C	0.8566
YDL155W	YGL197W	0.7380
YDL155W	YGR092W	0.8462
YDL155W	YLR079W	0.9794
YDL155W	YLR131C	0.8462
YDL155W	YLR310C	0.8462
YDL155W	YPL267W	0.7380
YDL156W	YDR097C	0.7380
YDL156W	YJL173C	0.7380
YDL156W	YNL312W	0.7380
YDL159W	YDR103W	1.0000
YDL159W	YFR028C	0.8566
YDL159W	YGR040W	1.0000
YDL159W	YLR113W	0.8566
YDL159W	YLR362W	0.9991
YDL160C	YDR170C	0.7380
YDL160C	YDR378C	0.8028
YDL160C	YEL015W	0.9860
YDL160C	YER112W	0.8028
YDL160C	YFR004W	0.7380
YDL160C	YGL173C	0.8087
YDL160C	YGR178C	0.5107
YDL160C	YJL124C	0.9808
YDL160C	YLR438C-A	0.7187
YDL160C	YNL118C	0.9953
YDL160C	YNL147W	0.9313
YDL160C	YNR052C	0.9747
YDL160C	YOL149W	0.8629
YDL160C	YPR110C	0.7380
YDL160C	YPR129W	0.6147
YDL161W	YDR379W	0.9099
YDL161W	YGL206C	0.9730
YDL161W	YIR006C	0.8965
YDL161W	YOR127W	0.9099
YDL164C	YER173W	0.8714
YDL165W	YER068W	0.9409
YDL165W	YIL038C	0.9409
YDL165W	YNL288W	0.9991
YDL165W	YNR052C	0.9977
YDL165W	YPR072W	0.9985
YDL166C	YJL191W	0.8628
YDL171C	YFR004W	0.7380
YDL175C	YDR432W	0.8756
YDL175C	YIL079C	0.8953
YDL175C	YJL050W	0.9992
YDL175C	YMR125W	0.7380

YDL175C	YNL251C	0.9021
YDL175C	YNL299W	0.8603
YDL175C	YOL115W	1.0000
YDL175C	YPL190C	0.8659
YDL176W	YGL227W	0.8087
YDL178W	YFL039C	0.9999
YDL179W	YPL031C	0.9923
YDL181W	YKR054C	0.5774
YDL184C	YER146W	0.3670
YDL184C	YJL124C	0.3670
YDL185W	YEL051W	0.9984
YDL185W	YER171W	0.8659
YDL185W	YFR004W	0.7380
YDL185W	YGR020C	0.9802
YDL185W	YHR039C-A	0.9970
YDL185W	YJR033C	0.8566
YDL185W	YKL080W	0.9266
YDL185W	YLR447C	0.9249
YDL185W	YMR054W	0.9099
YDL185W	YOR270C	1.0000
YDL185W	YOR332W	0.9788
YDL185W	YPR036W	0.9794
YDL188C	YGL190C	0.9998
YDL188C	YGR161C	0.7380
YDL188C	YJR009C	0.7380
YDL188C	YML109W	0.8659
YDL188C	YMR028W	0.9964
YDL188C	YMR273C	0.9313
YDL188C	YNL209W	0.7380
YDL188C	YOR014W	0.9313
YDL188C	YPL152W	0.9249
YDL188C	YPR040W	0.9414
YDL190C	YEL037C	1.0000
YDL190C	YFL044C	0.9512
YDL190C	YGR048W	0.9555
YDL190C	YLL039C	0.9942
YDL190C	YMR276W	0.9996
YDL191W	YHR013C	0.9215
YDL191W	YHR197W	0.9158
YDL192W	YDL226C	0.9984
YDL192W	YDR358W	0.9921
YDL192W	YEL022W	0.9809
YDL192W	YER122C	0.9754
YDL192W	YER165W	0.9663
YDL192W	YFR004W	0.7380
YDL192W	YHR108W	0.9857
YDL192W	YIL004C	0.9215

YDL192W	YJL099W	0.9249
YDL192W	YLR268W	0.9215
YDL192W	YLR330W	0.9955
YDL192W	YMR237W	0.8953
YDL192W	YOL018C	0.9555
YDL192W	YOR216C	0.9790
YDL194W	YDR277C	0.8965
YDL195W	YDR337W	0.7380
YDL195W	YDR382W	0.7380
YDL195W	YIL004C	0.9226
YDL195W	YIL109C	0.9068
YDL195W	YLR208W	1.0000
YDL195W	YNL112W	0.7380
YDL195W	YOR250C	0.7380
YDL195W	YPL085W	0.9758
YDL195W	YPR181C	0.9939
YDL199C	YPL240C	0.6672
YDL200C	YGR184C	0.8628
YDL200C	YKL010C	0.9249
YDL203C	YER125W	0.9899
YDL203C	YGR058W	0.5774
YDL203C	YLR371W	0.8028
YDL207W	YDR192C	0.9997
YDL207W	YEL024W	0.8965
YDL207W	YMR255W	0.9769
YDL207W	YOR046C	0.9994
YDL207W	YOR361C	0.9249
YDL207W	YPL020C	0.9099
YDL208W	YDL213C	0.6147
YDL208W	YDR510W	0.6672
YDL208W	YGL078C	0.7380
YDL208W	YGL120C	0.7187
YDL208W	YHR072W-A	0.6672
YDL208W	YHR089C	0.9998
YDL208W	YKL014C	0.8087
YDL208W	YLR175W	0.9982
YDL208W	YMR049C	0.7380
YDL208W	YMR310C	0.8659
YDL208W	YNL124W	0.9967
YDL209C	YDR364C	0.9313
YDL209C	YDR416W	0.9743
YDL209C	YDR482C	0.7380
YDL209C	YER029C	0.7380
YDL209C	YER172C	0.9902
YDL209C	YGL120C	0.9021
YDL209C	YGL128C	0.7380
YDL209C	YGR074W	0.7380

YDL209C	YGR129W	0.7380
YDL209C	YGR278W	0.8659
YDL209C	YHR165C	0.9933
YDL209C	YJR050W	0.7380
YDL209C	YKL095W	0.8659
YDL209C	YKL173W	0.9313
YDL209C	YLL036C	1.0000
YDL209C	YLR117C	0.9928
YDL209C	YLR147C	0.7380
YDL209C	YLR424W	0.9313
YDL209C	YML049C	0.7380
YDL209C	YMR125W	0.7380
YDL209C	YMR213W	0.9820
YDL209C	YPL151C	0.9869
YDL209C	YPL213W	0.9499
YDL209C	YPR101W	0.9313
YDL209C	YPR182W	0.7380
YDL212W	YDR508C	0.5107
YDL212W	YGR191W	0.4335
YDL212W	YKR039W	0.9275
YDL212W	YML038C	0.4335
YDL212W	YMR149W	0.4335
YDL213C	YDR324C	0.7380
YDL213C	YJL109C	0.8659
YDL213C	YMR128W	0.7380
YDL213C	YMR229C	0.8659
YDL213C	YOL010W	0.7380
YDL213C	YPL217C	0.7380
YDL215C	YKL171W	0.9312
YDL216C	YDR179C	0.9648
YDL216C	YIL071C	0.9648
YDL216C	YJR084W	0.9893
YDL216C	YMR025W	0.9128
YDL216C	YOL117W	0.9313
YDL217C	YGR033C	0.9574
YDL217C	YHR005C-A	0.9783
YDL217C	YJL054W	0.9790
YDL217C	YOR297C	0.9983
YDL220C	YDR082W	1.0000
YDL220C	YDR227W	0.9138
YDL220C	YGR092W	0.8639
YDL220C	YLR010C	0.9978
YDL220C	YLR233C	0.9994
YDL220C	YML109W	0.9758
YDL220C	YNL075W	0.9758
YDL220C	YNL102W	0.9998
YDL225W	YDR507C	0.9955

YDL225W	YDR510W	0.9670
YDL225W	YHR107C	0.9999
YDL225W	YJR076C	1.0000
YDL225W	YLR314C	0.9996
YDL225W	YNL078W	0.9099
YDL225W	YPL031C	0.7696
YDL225W	YPL153C	0.9464
YDL226C	YFR004W	0.7380
YDL226C	YOR327C	0.7909
YDL227C	YDR335W	0.9790
YDL227C	YML088W	0.9099
YDL227C	YMR308C	0.9138
YDL229W	YDR001C	0.7380
YDL229W	YDR017C	0.7380
YDL229W	YDR060W	0.7380
YDL229W	YDR061W	0.7380
YDL229W	YDR096W	0.7380
YDL229W	YDR097C	0.7380
YDL229W	YDR122W	0.7380
YDL229W	YDR127W	0.7380
YDL229W	YDR138W	0.7380
YDL229W	YDR141C	0.7380
YDL229W	YDR147W	0.7380
YDL229W	YDR153C	0.7380
YDL229W	YDR168W	0.7380
YDL229W	YDR170C	0.7380
YDL229W	YDR172W	0.9249
YDL229W	YDR174W	0.7380
YDL229W	YDR189W	0.7380
YDL229W	YDR201W	0.7380
YDL229W	YDR207C	0.7380
YDL229W	YDR214W	0.7380
YDL229W	YDR258C	0.7380
YDL229W	YDR295C	0.7380
YDL229W	YDR299W	0.7380
YDL229W	YDR301W	0.7380
YDL229W	YDR314C	0.7380
YDL229W	YDR320C	0.7380
YDL229W	YDR324C	0.7380
YDL229W	YDR337W	0.7380
YDL229W	YDR347W	0.7380
YDL229W	YDR359C	0.7380
YDL229W	YDR379W	0.7380
YDL229W	YDR395W	0.7380
YDL229W	YDR432W	0.9021
YDL229W	YDR460W	0.7380
YDL229W	YDR477W	0.9021

YDL229W	YDR490C	0.7380
YDL229W	YDR505C	0.7380
YDL229W	YDR510W	0.7380
YDL229W	YEL025C	0.7380
YDL229W	YEL071W	0.7380
YDL229W	YER022W	0.7380
YDL229W	YER033C	0.7380
YDL229W	YER088C	0.7380
YDL229W	YER103W	0.7380
YDL229W	YER112W	0.7380
YDL229W	YER129W	0.7380
YDL229W	YER132C	0.7380
YDL229W	YER133W	0.7380
YDL229W	YER147C	0.7380
YDL229W	YER162C	0.7380
YDL229W	YER164W	0.7380
YDL229W	YER169W	0.7380
YDL229W	YER172C	0.7380
YDL229W	YFL008W	0.7380
YDL229W	YFL013C	0.7380
YDL229W	YFL018C	0.7380
YDL229W	YFL023W	0.9777
YDL229W	YFL033C	0.7380
YDL229W	YFL052W	0.7380
YDL229W	YFR004W	0.7380
YDL229W	YFR015C	0.7380
YDL229W	YFR016C	0.7380
YDL229W	YFR019W	0.7380
YDL229W	YFR031C	0.7380
YDL229W	YGL005C	0.7380
YDL229W	YGL043W	0.7380
YDL229W	YGL048C	0.7380
YDL229W	YGL076C	0.7380
YDL229W	YGL086W	0.7380
YDL229W	YGL131C	0.7380
YDL229W	YGL207W	0.7380
YDL229W	YGL232W	0.7380
YDL229W	YGR027C	0.7380
YDL229W	YGR086C	0.7380
YDL229W	YGR090W	0.7380
YDL229W	YGR100W	0.7380
YDL229W	YGR130C	0.7380
YDL229W	YGR192C	0.7380
YDL229W	YGR198W	0.7380
YDL229W	YGR204W	0.7380
YDL229W	YGR240C	0.7380
YDL229W	YGR254W	0.7380

YDL229W	YGR264C	0.7380
YDL229W	YGR267C	0.7380
YDL229W	YGR270W	0.7380
YDL229W	YGR278W	0.7380
YDL229W	YHL007C	0.7380
YDL229W	YHL008C	0.7380
YDL229W	YHL009C	0.7380
YDL229W	YHL014C	0.7380
YDL229W	YHL024W	0.7380
YDL229W	YHL029C	0.7380
YDL229W	YHL030W	0.7380
YDL229W	YHL033C	0.7380
YDL229W	YHL039W	0.7380
YDL229W	YHR009C	0.7380
YDL229W	YHR012W	0.7380
YDL229W	YHR024C	0.7380
YDL229W	YHR061C	0.7380
YDL229W	YHR062C	0.7380
YDL229W	YHR064C	0.7380
YDL229W	YHR069C	0.7380
YDL229W	YHR080C	0.7380
YDL229W	YHR085W	0.7380
YDL229W	YHR097C	0.7380
YDL229W	YHR104W	0.7380
YDL229W	YHR111W	0.7380
YDL229W	YHR114W	0.7380
YDL229W	YHR118C	0.7380
YDL229W	YHR150W	0.7380
YDL229W	YHR164C	0.7380
YDL229W	YHR165C	0.7380
YDL229W	YHR174W	0.7380
YDL229W	YHR200W	0.8659
YDL229W	YHR203C	0.7380
YDL229W	YIL005W	0.7380
YDL229W	YIL035C	0.7380
YDL229W	YIL041W	0.7380
YDL229W	YIL043C	0.7380
YDL229W	YIL050W	0.7380
YDL229W	YIL053W	0.7380
YDL229W	YIL061C	0.7380
YDL229W	YIL070C	0.7380
YDL229W	YIL075C	0.7380
YDL229W	YIL078W	0.7380
YDL229W	YIL091C	0.7380
YDL229W	YIL112W	0.7380
YDL229W	YIL126W	0.7380
YDL229W	YIL128W	0.7380

YDL229W	YIR039C	0.7380
YDL229W	YJL006C	0.7380
YDL229W	YJL008C	0.7380
YDL229W	YJL023C	0.7380
YDL229W	YJL050W	0.7380
YDL229W	YJL053W	0.7380
YDL229W	YJL074C	0.7380
YDL229W	YJL092W	0.7380
YDL229W	YJL095W	0.7380
YDL229W	YJL109C	0.7380
YDL229W	YJL110C	0.7380
YDL229W	YJL115W	0.7380
YDL229W	YJL122W	0.7380
YDL229W	YJL157C	0.7380
YDL229W	YJL168C	0.7380
YDL229W	YJL179W	0.7380
YDL229W	YJR041C	0.7380
YDL229W	YJR062C	0.7380
YDL229W	YJR065C	0.7380
YDL229W	YJR093C	0.7380
YDL229W	YJR105W	0.7380
YDL229W	YJR137C	0.7380
YDL229W	YKL014C	0.7380
YDL229W	YKL023W	0.7380
YDL229W	YKL033W	0.7380
YDL229W	YKL060C	0.7380
YDL229W	YKL073W	0.7380
YDL229W	YKL103C	0.7380
YDL229W	YKL113C	0.7380
YDL229W	YKL157W	0.7380
YDL229W	YKL173W	0.7380
YDL229W	YKL195W	0.7380
YDL229W	YKL215C	0.7380
YDL229W	YKR002W	0.7380
YDL229W	YKR008W	0.7380
YDL229W	YKR010C	0.7380
YDL229W	YKR024C	0.7380
YDL229W	YKR038C	0.7380
YDL229W	YKR056W	0.7380
YDL229W	YKR059W	0.7380
YDL229W	YKR088C	0.7380
YDL229W	YKR092C	0.7380
YDL229W	YKR101W	0.7380
YDL229W	YLL013C	0.7380
YDL229W	YLL022C	0.7380
YDL229W	YLL024C	0.7380
YDL229W	YLL035W	0.7380

YDL229W	YLL039C	0.7380
YDL229W	YLL045C	0.7380
YDL229W	YLR015W	0.7380
YDL229W	YLR028C	0.7380
YDL229W	YLR039C	0.7380
YDL229W	YLR069C	0.7380
YDL229W	YLR071C	0.7380
YDL229W	YLR096W	0.7380
YDL229W	YLR176C	0.7380
YDL229W	YLR180W	0.7380
YDL229W	YLR187W	0.7380
YDL229W	YLR189C	0.7380
YDL229W	YLR196W	0.7380
YDL229W	YLR209C	0.7380
YDL229W	YLR222C	0.7380
YDL229W	YLR244C	0.7380
YDL229W	YLR268W	0.7380
YDL229W	YLR274W	0.7380
YDL229W	YLR288C	0.7380
YDL229W	YLR298C	0.7380
YDL229W	YLR304C	0.7380
YDL229W	YLR330W	0.7380
YDL229W	YLR335W	0.8659
YDL229W	YLR347C	0.7380
YDL229W	YLR354C	0.7380
YDL229W	YLR357W	0.7380
YDL229W	YLR371W	0.7380
YDL229W	YLR398C	0.7380
YDL229W	YLR409C	0.7380
YDL229W	YLR410W	0.7380
YDL229W	YLR418C	0.7380
YDL229W	YLR424W	0.7380
YDL229W	YLR442C	0.7380
YDL229W	YLR452C	0.7380
YDL229W	YML004C	0.7380
YDL229W	YML016C	0.7380
YDL229W	YML036W	0.7380
YDL229W	YML048W	0.7380
YDL229W	YML062C	0.7380
YDL229W	YML069W	0.7380
YDL229W	YML070W	0.7380
YDL229W	YML074C	0.7380
YDL229W	YML096W	0.7380
YDL229W	YML115C	0.7380
YDL229W	YML124C	0.7380
YDL229W	YMR014W	0.7380
YDL229W	YMR021C	0.7380

YDL229W	YMR027W	0.7380
YDL229W	YMR031C	0.7380
YDL229W	YMR049C	0.7380
YDL229W	YMR060C	0.7380
YDL229W	YMR066W	0.7380
YDL229W	YMR075W	0.7380
YDL229W	YMR076C	0.7380
YDL229W	YMR093W	0.7380
YDL229W	YMR099C	0.7380
YDL229W	YMR121C	0.7380
YDL229W	YMR128W	0.7380
YDL229W	YMR176W	0.7380
YDL229W	YMR179W	0.7380
YDL229W	YMR186W	0.7380
YDL229W	YMR229C	0.7380
YDL229W	YMR242C	0.7380
YDL229W	YMR259C	0.7380
YDL229W	YMR273C	0.7380
YDL229W	YMR285C	0.7380
YDL229W	YMR288W	0.7380
YDL229W	YMR318C	0.7380
YDL229W	YNL004W	0.7380
YDL229W	YNL007C	0.7380
YDL229W	YNL021W	0.7380
YDL229W	YNL023C	0.7380
YDL229W	YNL032W	0.7380
YDL229W	YNL049C	0.7380
YDL229W	YNL064C	0.7380
YDL229W	YNL088W	0.7380
YDL229W	YNL097C	0.7380
YDL229W	YNL102W	0.7380
YDL229W	YNL106C	0.7380
YDL229W	YNL112W	0.7380
YDL229W	YNL118C	0.7380
YDL229W	YNL132W	0.7380
YDL229W	YNL209W	0.7380
YDL229W	YNL224C	0.7380
YDL229W	YNL251C	0.7380
YDL229W	YNL273W	0.7380
YDL229W	YNL287W	0.7380
YDL229W	YNL288W	0.7380
YDL229W	YNL313C	0.7380
YDL229W	YNL315C	0.7380
YDL229W	YNR003C	0.7380
YDL229W	YNR029C	0.7380
YDL229W	YNR038W	0.7380
YDL229W	YOL004W	0.7380

YDL229W	YOL006C	0.7380
YDL229W	YOL010W	0.7380
YDL229W	YOL012C	0.7380
YDL229W	YOL021C	0.7380
YDL229W	YOL023W	0.7380
YDL229W	YOL056W	0.7380
YDL229W	YOL057W	0.7380
YDL229W	YOL061W	0.7380
YDL229W	YOL080C	0.7380
YDL229W	YOL097C	0.7380
YDL229W	YOL098C	0.7380
YDL229W	YOL151W	0.7380
YDL229W	YOR027W	0.7380
YDL229W	YOR038C	0.7380
YDL229W	YOR061W	0.7380
YDL229W	YOR080W	0.7380
YDL229W	YOR086C	0.7380
YDL229W	YOR089C	0.7380
YDL229W	YOR098C	0.8659
YDL229W	YOR112W	0.7380
YDL229W	YOR124C	0.7380
YDL229W	YOR172W	0.7380
YDL229W	YOR191W	0.7380
YDL229W	YOR207C	0.7380
YDL229W	YOR213C	0.7380
YDL229W	YOR217W	0.7380
YDL229W	YOR227W	0.7380
YDL229W	YOR253W	0.7380
YDL229W	YOR304W	0.7380
YDL229W	YOR310C	0.7380
YDL229W	YOR319W	0.7380
YDL229W	YOR335C	0.7380
YDL229W	YOR341W	0.7380
YDL229W	YOR355W	0.7380
YDL229W	YOR361C	0.7380
YDL229W	YPL001W	0.7380
YDL229W	YPL002C	0.7380
YDL229W	YPL012W	0.7380
YDL229W	YPL022W	0.7380
YDL229W	YPL045W	0.7380
YDL229W	YPL048W	0.7380
YDL229W	YPL064C	0.7380
YDL229W	YPL104W	0.7380
YDL229W	YPL106C	0.9998
YDL229W	YPL126W	0.7380
YDL229W	YPL166W	0.7380
YDL229W	YPL167C	0.7380

YDL229W	YPL181W	0.7380
YDL229W	YPL183C	0.7380
YDL229W	YPL198W	0.7380
YDL229W	YPL207W	0.7380
YDL229W	YPL212C	0.7380
YDL229W	YPL226W	0.7380
YDL229W	YPL240C	0.8659
YDL229W	YPR010C	0.7380
YDL229W	YPR023C	0.7380
YDL229W	YPR025C	0.7380
YDL229W	YPR026W	0.7380
YDL229W	YPR031W	0.7380
YDL229W	YPR041W	0.7380
YDL229W	YPR089W	0.7380
YDL229W	YPR093C	0.7380
YDL229W	YPR095C	0.7380
YDL229W	YPR105C	0.7380
YDL229W	YPR110C	0.7380
YDL229W	YPR112C	0.7380
YDL229W	YPR135W	0.7380
YDL229W	YPR140W	0.7380
YDL229W	YPR163C	0.7380
YDL229W	YPR169W	0.7380
YDL229W	YPR190C	0.7380
YDL229W	YPR193C	0.7380
YDL232W	YEL002C	0.9409
YDL232W	YER087C-B	0.9081
YDL232W	YGL022W	0.9995
YDL232W	YGL226C-A	0.9215
YDL232W	YJL002C	0.9938
YDL232W	YML019W	0.9999
YDL232W	YMR149W	0.9409
YDL232W	YOR085W	1.0000
YDL232W	YOR103C	0.9780
YDL235C	YHR206W	0.9933
YDL235C	YIL147C	1.0000
YDL235C	YLR006C	1.0000
YDL239C	YHR184W	0.9850
YDL239C	YKL103C	0.5774
YDL239C	YLR098C	0.5774
YDL239C	YLR423C	0.5774
YDL239C	YNL225C	0.8965
YDL239C	YOL091W	0.9327
YDL239C	YOR177C	0.8798
YDL239C	YOR373W	0.8965
YDL239C	YPL070W	0.5774
YDL240W	YPR165W	0.9503

YDR001C	YDR099W	0.9986
YDR001C	YER177W	0.9972
YDR001C	YJL164C	0.8939
YDR001C	YLR270W	0.5774
YDR001C	YPL203W	0.8714
YDR002W	YFR004W	0.7380
YDR002W	YGR218W	0.9932
YDR002W	YLR293C	1.0000
YDR003W	YER125W	0.8714
YDR004W	YDR076W	0.9986
YDR004W	YER095W	0.8928
YDR004W	YLR394W	0.9099
YDR005C	YHR186C	0.9215
YDR005C	YHR205W	0.7696
YDR005C	YNR003C	0.9464
YDR005C	YOR116C	1.0000
YDR005C	YOR207C	0.8087
YDR005C	YPR110C	0.9464
YDR005C	YPR190C	0.9988
YDR006C	YFR004W	0.7380
YDR009W	YML051W	1.0000
YDR012W	YDR496C	0.7380
YDR012W	YKR081C	0.7380
YDR012W	YLL039C	0.8087
YDR012W	YNL061W	0.7380
YDR012W	YOL041C	0.7380
YDR012W	YOL120C	0.7380
YDR013W	YDR489W	0.9858
YDR013W	YGL113W	0.9710
YDR013W	YJL072C	0.9895
YDR013W	YJL090C	0.9099
YDR013W	YOL146W	0.9616
YDR013W	YPR135W	0.9723
YDR013W	YPR175W	0.9099
YDR014W	YIL026C	0.9215
YDR014W	YMR076C	0.9929
YDR014W-A	YER095W	0.9723
YDR016C	YDR201W	0.9989
YDR016C	YDR320C-A	0.9871
YDR016C	YGL061C	1.0000
YDR016C	YGR113W	0.9990
YDR016C	YKL052C	0.9968
YDR016C	YKL138C-A	0.9747
YDR016C	YKR037C	1.0000
YDR016C	YKR083C	0.9960
YDR017C	YDR099W	0.6672
YDR020C	YNR012W	0.9086

YDR022C	YGL180W	0.8566
YDR022C	YLR423C	0.9866
YDR022C	YPL166W	0.9539
YDR023W	YFR004W	0.7380
YDR023W	YGR239C	0.9994
YDR025W	YGR090W	0.7380
YDR025W	YMR309C	0.7380
YDR025W	YNL132W	0.7380
YDR025W	YNL308C	0.7380
YDR026C	YDR110W	0.7909
YDR027C	YDR468C	0.9249
YDR027C	YDR484W	1.0000
YDR027C	YJL029C	0.9985
YDR027C	YKR020W	0.9707
YDR027C	YLR262C	0.9790
YDR027C	YOL018C	0.9981
YDR028C	YDR099W	0.9958
YDR028C	YDR477W	0.9999
YDR028C	YER027C	0.7380
YDR028C	YER129W	0.9215
YDR028C	YER133W	1.0000
YDR028C	YER177W	0.9997
YDR028C	YGL115W	0.8659
YDR028C	YJR090C	0.9099
YDR032C	YFR004W	0.7380
YDR032C	YPL106C	0.7380
YDR036C	YDR041W	0.9313
YDR036C	YDR175C	0.8659
YDR036C	YDR337W	0.8659
YDR036C	YDR347W	0.8659
YDR036C	YGL129C	0.8659
YDR036C	YGR084C	0.8659
YDR036C	YHL004W	0.9648
YDR036C	YIL093C	0.8659
YDR036C	YJR113C	0.8659
YDR036C	YKL155C	0.8659
YDR036C	YMR188C	0.8659
YDR036C	YNL137C	0.8659
YDR036C	YNL306W	0.8659
YDR036C	YPL118W	0.8659
YDR037W	YFR004W	0.7380
YDR037W	YJL124C	0.7380
YDR037W	YPR056W	0.7380
YDR041W	YDR337W	0.7380
YDR041W	YDR347W	0.7380
YDR041W	YER050C	0.7380
YDR041W	YGL129C	0.7380

YDR041W	YGR084C	0.7380
YDR041W	YHL004W	0.9743
YDR041W	YJR101W	0.7380
YDR041W	YJR113C	0.7380
YDR041W	YKL155C	0.9624
YDR041W	YMR188C	0.7380
YDR041W	YNL137C	0.8659
YDR041W	YNL306W	0.7380
YDR041W	YOR204W	0.7380
YDR041W	YPL013C	0.7380
YDR041W	YPL118W	0.7380
YDR043C	YIL035C	0.7543
YDR044W	YGL112C	0.7380
YDR045C	YJL011C	0.8353
YDR045C	YNR003C	0.8659
YDR045C	YOR116C	0.8659
YDR045C	YOR207C	0.9962
YDR050C	YFR004W	0.7380
YDR050C	YMR186W	0.7380
YDR052C	YLL004W	0.9747
YDR052C	YMR001C	0.9995
YDR052C	YNL261W	0.9099
YDR052C	YPL153C	0.9993
YDR054C	YDR328C	0.9616
YDR054C	YFL009W	0.9939
YDR054C	YGL252C	0.7380
YDR054C	YIL046W	0.8566
YDR054C	YJL047C	0.9298
YDR054C	YJL187C	0.6042
YDR054C	YLL039C	0.8566
YDR054C	YLR079W	0.9962
YDR057W	YJL034W	0.8953
YDR057W	YLR207W	0.9977
YDR057W	YML013W	0.8953
YDR057W	YML029W	0.8603
YDR057W	YMR297W	0.9215
YDR057W	YOL013C	0.9609
YDR060W	YDR087C	0.8087
YDR060W	YDR382W	0.7380
YDR060W	YGL076C	0.7380
YDR060W	YGR103W	0.9808
YDR060W	YGR281W	0.7380
YDR060W	YHR052W	0.9648
YDR060W	YHR066W	0.9794
YDR060W	YIL133C	0.7380
YDR060W	YKL172W	0.6147
YDR060W	YKR081C	0.8659

YDR060W	YLL045C	0.7380
YDR060W	YLR002C	0.8521
YDR060W	YLR029C	0.7380
YDR060W	YLR221C	0.7380
YDR060W	YLR276C	0.8659
YDR060W	YMR049C	0.8659
YDR060W	YMR229C	0.9950
YDR060W	YMR290C	0.9313
YDR060W	YNL002C	0.8659
YDR060W	YNL061W	0.8659
YDR060W	YNL110C	0.9648
YDR060W	YOL041C	0.8659
YDR060W	YOL077C	0.9313
YDR060W	YOR063W	0.7380
YDR060W	YOR206W	1.0000
YDR060W	YOR272W	0.8659
YDR060W	YPL012W	0.8659
YDR060W	YPL043W	0.9648
YDR060W	YPL131W	0.7380
YDR060W	YPL211W	0.8659
YDR062W	YDR099W	0.7380
YDR062W	YDR502C	0.7380
YDR062W	YEL022W	0.7380
YDR062W	YER177W	0.7380
YDR062W	YGR038W	0.8953
YDR062W	YGR218W	0.7380
YDR062W	YHL030W	0.7380
YDR062W	YJR077C	0.7380
YDR062W	YLR180W	0.7380
YDR062W	YLR350W	0.9707
YDR062W	YMR296C	0.9996
YDR064W	YFR004W	0.7380
YDR064W	YGR090W	0.8659
YDR064W	YHR196W	0.7380
YDR064W	YJL109C	0.7380
YDR064W	YNL132W	0.7380
YDR064W	YNL207W	0.7380
YDR064W	YNL308C	0.7380
YDR064W	YOR310C	0.8659
YDR064W	YPR137W	0.7380
YDR064W	YPR144C	0.8659
YDR064W	YPR189W	0.7380
YDR069C	YLR025W	0.9025
YDR069C	YPL084W	0.9998
YDR071C	YER089C	0.9974
YDR073W	YHL025W	0.9999
YDR073W	YJL176C	0.9879

YDR073W	YOR290C	0.9962
YDR073W	YPL129W	0.8566
YDR074W	YFR004W	0.7380
YDR074W	YML100W	0.9779
YDR074W	YMR261C	0.9887
YDR075W	YDR225W	0.9464
YDR075W	YDR293C	0.8087
YDR075W	YGR192C	0.7380
YDR075W	YML010W	0.9634
YDR075W	YMR028W	0.9249
YDR075W	YNL201C	1.0000
YDR076W	YER095W	0.9859
YDR076W	YPL153C	0.8456
YDR077W	YEL065W	0.9099
YDR077W	YOR330C	0.9099
YDR079C-A	YPL122C	1.0000
YDR079C-A	YPR056W	0.9834
YDR080W	YDR495C	0.9215
YDR080W	YGL212W	0.9215
YDR080W	YLR148W	0.9997
YDR080W	YLR396C	1.0000
YDR080W	YML001W	0.9932
YDR080W	YMR231W	0.9989
YDR080W	YOR106W	0.9929
YDR080W	YPL045W	0.9994
YDR080W	YPL195W	0.9994
YDR082W	YLR010C	1.0000
YDR084C	YGL161C	0.8816
YDR084C	YGL198W	0.9784
YDR085C	YER133W	0.9864
YDR085C	YHR107C	0.9710
YDR086C	YEL002C	0.9742
YDR086C	YER087C-B	0.9780
YDR086C	YLR378C	0.9964
YDR086C	YMR149W	0.9151
YDR086C	YPL094C	0.8953
YDR087C	YGL111W	0.9285
YDR087C	YGR103W	0.9285
YDR087C	YHR052W	0.9499
YDR087C	YHR066W	0.8603
YDR087C	YHR088W	0.8087
YDR087C	YJL095W	0.5779
YDR087C	YKR081C	0.9499
YDR087C	YLL008W	0.8087
YDR087C	YMR049C	0.8087
YDR087C	YMR290C	0.8087
YDR087C	YNL002C	0.8087

YDR087C	YNL061W	0.9743
YDR087C	YNL110C	0.8087
YDR087C	YOL077C	0.9021
YDR087C	YOR063W	0.9707
YDR087C	YOR206W	0.8087
YDR087C	YOR272W	0.9021
YDR087C	YPL093W	0.8603
YDR087C	YPL141C	0.5779
YDR087C	YPL211W	0.8087
YDR087C	YPR016C	0.9285
YDR091C	YDR172W	0.9215
YDR091C	YFR004W	0.7380
YDR091C	YGL076C	0.8087
YDR091C	YLL045C	0.8087
YDR091C	YLR192C	0.9921
YDR091C	YML024W	0.8087
YDR091C	YML063W	0.8087
YDR091C	YMR309C	0.8659
YDR091C	YNL260C	0.7380
YDR091C	YOR096W	0.7380
YDR091C	YPR041W	0.8659
YDR092W	YGL087C	1.0000
YDR092W	YLR032W	0.9990
YDR092W	YMR140W	0.6147
YDR096W	YFL023W	0.9099
YDR096W	YML114C	0.9099
YDR096W	YPR169W	0.9099
YDR097C	YDR499W	0.7380
YDR097C	YIR002C	0.7380
YDR097C	YJL173C	0.7380
YDR097C	YKR001C	0.7380
YDR097C	YML032C	0.7380
YDR097C	YNL312W	0.7380
YDR097C	YOL090W	1.0000
YDR098C	YER174C	0.9138
YDR098C	YGL071W	0.9949
YDR098C	YGL220W	0.9978
YDR098C	YGR262C	0.7380
YDR099W	YDR130C	0.9991
YDR099W	YDR373W	0.8953
YDR099W	YER133W	0.7380
YDR099W	YER177W	0.9964
YDR099W	YFR004W	0.7380
YDR099W	YFR014C	0.5779
YDR099W	YGL003C	0.8603
YDR099W	YGL252C	0.9950
YDR099W	YJR090C	0.9099

YDR099W	YKL182W	0.8566
YDR099W	YLR177W	0.7909
YDR099W	YNL031C	0.9702
YDR099W	YNL267W	0.9993
YDR099W	YPL231W	0.8566
YDR100W	YMR071C	0.9215
YDR101C	YER006W	0.9869
YDR101C	YER036C	0.9464
YDR101C	YER126C	0.9624
YDR101C	YFR031C-A	0.8659
YDR101C	YGL030W	0.7380
YDR101C	YGL099W	0.9902
YDR101C	YGR103W	0.9994
YDR101C	YGR245C	0.9820
YDR101C	YHR052W	0.9313
YDR101C	YHR170W	0.9999
YDR101C	YHR197W	0.9963
YDR101C	YIR026C	0.9215
YDR101C	YJL122W	1.0000
YDR101C	YKL009W	0.9984
YDR101C	YKL068W	0.9947
YDR101C	YKR081C	0.8659
YDR101C	YLL045C	0.8659
YDR101C	YLR009W	0.9707
YDR101C	YLR074C	0.8659
YDR101C	YLR106C	0.7380
YDR101C	YLR340W	0.8566
YDR101C	YMR047C	0.9138
YDR101C	YMR308C	0.9266
YDR101C	YNL002C	0.7380
YDR101C	YNL110C	0.8659
YDR101C	YNL182C	0.7380
YDR101C	YNR053C	0.9895
YDR101C	YOR063W	0.9803
YDR101C	YPL093W	0.9994
YDR101C	YPL131W	0.9887
YDR101C	YPL169C	0.9249
YDR101C	YPL211W	0.7380
YDR101C	YPL249C-A	0.7380
YDR101C	YPR016C	0.9984
YDR103W	YGR040W	0.9769
YDR103W	YLL039C	0.8566
YDR103W	YLR362W	1.0000
YDR103W	YOR212W	1.0000
YDR106W	YHR129C	0.9986
YDR106W	YLL049W	0.9099
YDR106W	YMR294W	0.9710

YDR106W	YPL174C	0.9790
YDR108W	YDR246W	0.8566
YDR108W	YDR472W	0.9624
YDR108W	YKR068C	0.9999
YDR108W	YML077W	0.8566
YDR108W	YOR115C	0.9970
YDR110W	YFR028C	0.8953
YDR110W	YHR152W	0.9414
YDR110W	YJL076W	0.9974
YDR110W	YKR010C	0.8603
YDR113C	YGL003C	0.7696
YDR113C	YGL116W	0.9997
YDR113C	YGR098C	0.9985
YDR113C	YIL005W	0.9215
YDR115W	YKL142W	0.5774
YDR116C	YDR296W	0.7380
YDR116C	YGR220C	0.8659
YDR116C	YJL041W	0.7380
YDR116C	YML025C	0.7380
YDR116C	YNL284C	0.7380
YDR118W	YDR260C	0.9998
YDR118W	YFR036W	0.8603
YDR118W	YGL240W	0.9972
YDR118W	YHR166C	0.9991
YDR118W	YIR025W	0.9998
YDR118W	YKL022C	1.0000
YDR118W	YLR102C	0.9962
YDR118W	YLR127C	0.9996
YDR118W	YNL172W	0.9925
YDR118W	YOR249C	0.9984
YDR121W	YGL133W	0.9726
YDR121W	YJL065C	0.9950
YDR121W	YNL262W	1.0000
YDR121W	YOR304W	0.9996
YDR121W	YPR175W	0.9990
YDR122W	YGR238C	0.6147
YDR122W	YHR158C	0.6147
YDR122W	YJL187C	0.8566
YDR122W	YNL161W	0.7100
YDR122W	YOL082W	0.6672
YDR123C	YHL020C	0.9995
YDR123C	YJL168C	0.9282
YDR123C	YOL108C	1.0000
YDR123C	YPR086W	0.9215
YDR127W	YDR427W	0.7380
YDR127W	YER095W	0.7380
YDR127W	YFR004W	0.7380

YDR127W	YGR234W	0.7380
YDR127W	YGR240C	0.8659
YDR127W	YJL008C	0.7380
YDR127W	YKL104C	0.8659
YDR127W	YLR180W	0.7380
YDR127W	YMR186W	0.8659
YDR127W	YPL235W	0.7380
YDR129C	YFL039C	0.9998
YDR129C	YFR004W	0.7380
YDR130C	YDR201W	0.7807
YDR130C	YDR318W	0.7807
YDR130C	YER133W	0.9739
YDR130C	YER177W	0.9802
YDR130C	YGL093W	0.7807
YDR130C	YGR113W	0.7807
YDR130C	YIL144W	0.9319
YDR130C	YIR010W	0.8877
YDR130C	YKL052C	0.7807
YDR130C	YOL069W	0.7807
YDR131C	YDR328C	0.9298
YDR135C	YGL181W	0.8965
YDR135C	YLR425W	0.9151
YDR137W	YLR039C	0.9808
YDR137W	YLR262C	0.8087
YDR138W	YDR381W	0.9987
YDR138W	YER125W	0.8714
YDR138W	YHR167W	0.9997
YDR138W	YML062C	0.9999
YDR138W	YNL004W	0.9904
YDR138W	YNL139C	1.0000
YDR138W	YNL253W	0.9726
YDR138W	YPL169C	0.9998
YDR139C	YDR328C	0.7909
YDR139C	YLR306W	0.9249
YDR139C	YPR066W	0.9249
YDR140W	YNR046W	0.9606
YDR141C	YNL209W	0.7380
YDR141C	YNL297C	0.9958
YDR142C	YDR244W	0.9919
YDR142C	YGL153W	1.0000
YDR142C	YGR077C	0.8965
YDR142C	YGR239C	0.9924
YDR142C	YHR160C	1.0000
YDR142C	YIL160C	0.9987
YDR142C	YJR012C	0.7380
YDR142C	YLR191W	0.9922
YDR142C	YNL214W	0.9780

YDR143C	YLL039C	0.8659
YDR145W	YDR167W	1.0000
YDR145W	YDR176W	0.9808
YDR145W	YDR392W	0.9803
YDR145W	YDR448W	1.0000
YDR145W	YEL009C	0.9249
YDR145W	YER148W	1.0000
YDR145W	YGL066W	0.9313
YDR145W	YGL112C	1.0000
YDR145W	YGR252W	0.9946
YDR145W	YGR274C	1.0000
YDR145W	YHR099W	0.9996
YDR145W	YLR055C	0.9979
YDR145W	YML015C	0.9950
YDR145W	YML098W	0.9648
YDR145W	YML114C	0.9871
YDR145W	YMR005W	0.9999
YDR145W	YMR223W	0.9860
YDR145W	YMR227C	0.9764
YDR145W	YMR236W	1.0000
YDR145W	YNL236W	0.8566
YDR145W	YOL051W	0.9762
YDR145W	YOL148C	0.9986
YDR145W	YPL011C	0.9820
YDR145W	YPL047W	0.9634
YDR145W	YPL082C	0.9464
YDR145W	YPL129W	0.7380
YDR145W	YPL248C	0.9642
YDR145W	YPL254W	0.9993
YDR145W	YPR072W	0.8566
YDR146C	YDR335W	0.5774
YDR146C	YER022W	0.9079
YDR146C	YOR290C	0.9079
YDR148C	YFL018C	0.9313
YDR148C	YFR049W	0.8297
YDR148C	YIL125W	0.9894
YDR150W	YDR247W	0.7380
YDR150W	YER120W	0.7380
YDR150W	YLL001W	0.9454
YDR152W	YGL195W	0.9099
YDR152W	YGR173W	0.9982
YDR153C	YGL206C	0.8953
YDR153C	YHR108W	0.9976
YDR153C	YKL135C	0.8953
YDR153C	YPR029C	0.9138
YDR155C	YDR510W	0.7380
YDR155C	YFR004W	0.7380

YDR155C	YGL194C	0.9726
YDR155C	YIL112W	0.9499
YDR155C	YKR029C	0.8953
YDR155C	YOR290C	0.7380
YDR156W	YJL148W	0.7380
YDR156W	YJR063W	0.7380
YDR156W	YNL248C	0.7380
YDR156W	YOR210W	0.7380
YDR156W	YOR340C	1.0000
YDR156W	YOR341W	0.9726
YDR156W	YPR010C	0.7380
YDR156W	YPR110C	0.7380
YDR158W	YFR004W	0.7380
YDR159W	YDR363W-A	0.9035
YDR159W	YDR381W	0.7380
YDR159W	YDR448W	0.8566
YDR159W	YJL041W	0.8566
YDR159W	YKL186C	0.9616
YDR159W	YKR095W	0.8566
YDR159W	YMR047C	0.8566
YDR159W	YMR125W	0.7380
YDR159W	YOL072W	0.9999
YDR159W	YOR046C	0.8566
YDR159W	YOR098C	0.8566
YDR159W	YOR257W	0.9999
YDR159W	YPL169C	0.9945
YDR162C	YDR507C	0.8112
YDR162C	YHL007C	0.9853
YDR162C	YJL095W	0.9033
YDR162C	YJL128C	0.9976
YDR162C	YKR048C	0.8178
YDR162C	YNL094W	0.5774
YDR162C	YNL298W	0.9165
YDR162C	YOL113W	0.6672
YDR162C	YPL161C	0.8112
YDR164C	YGR009C	0.9999
YDR164C	YMR183C	0.9994
YDR164C	YNR049C	1.0000
YDR164C	YOR327C	1.0000
YDR164C	YPL232W	1.0000
YDR166C	YER008C	1.0000
YDR166C	YGL233W	0.9717
YDR166C	YIL068C	0.9992
YDR166C	YJL085W	0.9979
YDR166C	YLR166C	1.0000
YDR166C	YPR055W	1.0000
YDR167W	YDR176W	0.9940

YDR167W	YDR392W	0.9583
YDR167W	YDR448W	1.0000
YDR167W	YER148W	1.0000
YDR167W	YGL066W	0.8659
YDR167W	YGL112C	1.0000
YDR167W	YGR252W	1.0000
YDR167W	YGR274C	1.0000
YDR167W	YHR099W	0.9583
YDR167W	YLR055C	0.9844
YDR167W	YML015C	0.9995
YDR167W	YML098W	0.9986
YDR167W	YML114C	1.0000
YDR167W	YMR005W	0.9997
YDR167W	YMR223W	0.9860
YDR167W	YMR227C	0.9989
YDR167W	YMR236W	1.0000
YDR167W	YOL148C	0.9971
YDR167W	YPL011C	1.0000
YDR167W	YPL047W	0.9634
YDR167W	YPL129W	0.7380
YDR167W	YPL248C	0.9642
YDR167W	YPL254W	0.9920
YDR168W	YER161C	0.8965
YDR168W	YGL201C	0.8965
YDR168W	YGR040W	0.9215
YDR168W	YLR362W	0.9929
YDR168W	YMR186W	0.9624
YDR168W	YOR027W	0.9249
YDR168W	YPL240C	0.9990
YDR169C	YDR510W	0.8659
YDR170C	YER125W	0.9876
YDR170C	YFR009W	0.7380
YDR170C	YFR044C	0.7380
YDR170C	YGL238W	0.7380
YDR170C	YHR020W	0.7380
YDR170C	YJR077C	0.7380
YDR170C	YKL104C	0.7380
YDR170C	YLR180W	0.7380
YDR170C	YLR342W	0.8659
YDR170C	YOR136W	0.7380
YDR171W	YDR430C	0.7380
YDR171W	YDR510W	0.6672
YDR171W	YFR028C	0.7380
YDR171W	YGL120C	0.7380
YDR171W	YIL126W	0.7380
YDR171W	YLL024C	0.7380
YDR171W	YLL039C	0.7380

YDR171W	YMR304W	0.7380
YDR171W	YOR151C	0.7380
YDR171W	YPL106C	0.7380
YDR171W	YPR035W	0.8659
YDR172W	YER165W	0.9981
YDR172W	YFR004W	0.7380
YDR172W	YGR072W	0.9249
YDR172W	YHR077C	0.9249
YDR172W	YLL024C	0.9707
YDR172W	YLL026W	0.9981
YDR172W	YML117W	0.7380
YDR172W	YMR080C	0.9983
YDR172W	YNL007C	0.9249
YDR172W	YNL064C	0.9799
YDR172W	YNL243W	0.9099
YDR172W	YOR069W	0.7380
YDR172W	YOR150W	0.7380
YDR172W	YPL237W	0.7380
YDR173C	YMR042W	0.9942
YDR173C	YMR043W	0.9942
YDR174W	YDR510W	0.8297
YDR174W	YGR274C	0.9099
YDR174W	YML015C	0.8179
YDR174W	YMR242C	0.7380
YDR174W	YPR104C	0.6672
YDR175C	YDR337W	0.7380
YDR175C	YDR347W	0.7380
YDR175C	YGR165W	0.7380
YDR175C	YHL004W	0.9021
YDR175C	YIL093C	0.7380
YDR175C	YJR113C	0.7380
YDR175C	YKL155C	0.9266
YDR175C	YNL137C	0.7380
YDR175C	YOR158W	0.7380
YDR175C	YPL013C	0.7380
YDR176W	YDR392W	0.9794
YDR176W	YDR448W	1.0000
YDR176W	YER148W	0.9616
YDR176W	YER151C	0.8965
YDR176W	YGL013C	0.9919
YDR176W	YGL066W	0.7380
YDR176W	YGL112C	1.0000
YDR176W	YGR252W	1.0000
YDR176W	YHR099W	0.9941
YDR176W	YLR055C	0.9979
YDR176W	YMR223W	0.9961
YDR176W	YMR236W	0.9983

YDR176W	YOL148C	0.9996
YDR176W	YOR023C	0.9782
YDR176W	YPL047W	0.9813
YDR176W	YPL254W	0.9860
YDR179C	YIL071C	0.9313
YDR179C	YJR084W	0.9893
YDR179C	YMR025W	0.9991
YDR179C	YOL117W	0.8659
YDR180W	YER147C	0.9726
YDR180W	YIL026C	0.9215
YDR181C	YJL115W	0.9747
YDR181C	YMR127C	1.0000
YDR181C	YOR213C	1.0000
YDR181C	YPR018W	0.9099
YDR188W	YER151C	0.7380
YDR188W	YGL190C	0.7380
YDR188W	YIL142W	0.7380
YDR188W	YJL014W	0.9883
YDR188W	YJL111W	0.7380
YDR188W	YKL029C	0.7380
YDR188W	YNL212W	0.7380
YDR188W	YOR281C	0.7380
YDR189W	YIL004C	0.9790
YDR189W	YKL196C	0.9195
YDR189W	YLR026C	1.0000
YDR189W	YLR078C	0.9983
YDR189W	YLR268W	0.9249
YDR189W	YOR075W	0.9993
YDR190C	YDR334W	0.9998
YDR190C	YDR485C	0.7380
YDR190C	YER092W	0.8659
YDR190C	YER148W	0.9249
YDR190C	YFL013C	0.9313
YDR190C	YFL039C	0.9021
YDR190C	YFR004W	0.7380
YDR190C	YGL150C	0.9990
YDR190C	YGR002C	0.9860
YDR190C	YHR034C	0.9909
YDR190C	YJL081C	0.9634
YDR190C	YLR052W	0.8659
YDR190C	YLR385C	0.7380
YDR190C	YLR399C	0.8566
YDR190C	YML041C	0.8659
YDR190C	YMR072W	0.7380
YDR190C	YNL059C	0.9313
YDR190C	YNL107W	0.9464
YDR190C	YNL215W	0.8659

YDR190C	YOL012C	0.9900
YDR190C	YOR141C	0.9499
YDR190C	YOR189W	0.8087
YDR190C	YPL129W	0.7380
YDR190C	YPL193W	0.8965
YDR190C	YPL235W	1.0000
YDR190C	YPL240C	0.7380
YDR192C	YER110C	0.8353
YDR192C	YGL122C	0.5164
YDR192C	YGR218W	0.9945
YDR192C	YLR293C	0.8353
YDR192C	YLR347C	0.9882
YDR192C	YMR255W	0.8965
YDR194C	YFL036W	0.8206
YDR195W	YDR301W	0.9313
YDR195W	YER133W	0.9994
YDR195W	YGR156W	0.9904
YDR195W	YJL033W	0.7380
YDR195W	YJR093C	0.9813
YDR195W	YKL018W	0.9930
YDR195W	YKL059C	0.9904
YDR195W	YKR002W	0.9499
YDR195W	YLR115W	0.9743
YDR195W	YLR277C	0.9869
YDR195W	YLR448W	0.7380
YDR195W	YMR061W	0.9021
YDR195W	YNL222W	0.9634
YDR195W	YNL317W	0.9904
YDR195W	YOR179C	0.9951
YDR195W	YPR107C	0.9869
YDR200C	YFR008W	0.8353
YDR200C	YMR052W	0.9811
YDR201W	YDR320C-A	0.6672
YDR201W	YGL061C	0.8297
YDR201W	YGR113W	0.9984
YDR201W	YKL052C	0.9897
YDR201W	YKL138C-A	0.8967
YDR201W	YKR037C	1.0000
YDR201W	YKR083C	0.9825
YDR202C	YDR328C	0.9266
YDR202C	YJR033C	0.9961
YDR202C	YKL080W	0.7579
YDR202C	YOR332W	0.8566
YDR206W	YOL149W	0.8179
YDR207C	YIL084C	0.9021
YDR207C	YJR094C	0.9989
YDR207C	YKL185W	0.8603

YDR207C	YMR139W	0.9817
YDR207C	YNL330C	0.9925
YDR207C	YOL004W	0.9970
YDR207C	YOR304W	0.9249
YDR207C	YPL181W	0.8087
YDR211W	YER025W	0.9998
YDR211W	YGR083C	1.0000
YDR211W	YJR007W	0.9998
YDR211W	YKR026C	1.0000
YDR211W	YLR291C	1.0000
YDR211W	YOR260W	1.0000
YDR211W	YPL237W	1.0000
YDR211W	YPR010C	0.7380
YDR212W	YGL116W	0.8566
YDR212W	YGL190C	0.7380
YDR212W	YJL014W	0.9772
YDR214W	YFR004W	0.7380
YDR214W	YGL195W	0.8659
YDR214W	YHR027C	0.7380
YDR214W	YMR186W	0.9999
YDR214W	YNL281W	0.9717
YDR214W	YPL240C	1.0000
YDR216W	YER177W	0.9764
YDR216W	YGL112C	0.8566
YDR217C	YJL090C	0.9099
YDR217C	YPL153C	1.0000
YDR218C	YJR076C	0.5774
YDR219C	YDR328C	0.8566
YDR219C	YHR117W	0.8953
YDR223W	YPR104C	0.9099
YDR224C	YDR225W	1.0000
YDR224C	YDR303C	0.7380
YDR224C	YDR510W	0.9266
YDR224C	YER030W	0.9754
YDR224C	YER110C	0.9282
YDR224C	YGL133W	0.7380
YDR224C	YGL150C	0.8603
YDR224C	YGL241W	0.9986
YDR224C	YIL126W	0.9855
YDR224C	YJL081C	0.8783
YDR224C	YKL139W	0.9790
YDR224C	YKR048C	0.9794
YDR224C	YLR357W	0.7380
YDR224C	YLR442C	0.9780
YDR224C	YML112W	0.9249
YDR224C	YMR308C	0.9799
YDR224C	YOL012C	0.9981

YDR224C	YOR116C	0.7380
YDR224C	YOR304W	0.7380
YDR224C	YPL082C	0.7380
YDR225W	YDR334W	0.8953
YDR225W	YDR448W	0.9186
YDR225W	YDR510W	0.8566
YDR225W	YER110C	0.9936
YDR225W	YGL097W	0.8998
YDR225W	YGL150C	0.8603
YDR225W	YGL207W	0.7380
YDR225W	YGL241W	1.0000
YDR225W	YGR252W	0.9237
YDR225W	YJL081C	0.9974
YDR225W	YKL049C	0.9215
YDR225W	YKL139W	0.9249
YDR225W	YKR048C	0.9997
YDR225W	YLR347C	0.9933
YDR225W	YLR357W	0.7380
YDR225W	YLR442C	0.9780
YDR225W	YML065W	0.8566
YDR225W	YMR061W	0.7380
YDR225W	YMR125W	0.7380
YDR225W	YMR308C	0.9982
YDR225W	YNL030W	0.9929
YDR225W	YNL031C	0.9981
YDR225W	YNL201C	0.8087
YDR225W	YOL012C	0.9994
YDR225W	YOR244W	0.7696
YDR226W	YDR510W	0.7380
YDR226W	YFR004W	0.7380
YDR226W	YGR092W	0.6042
YDR227W	YJL019W	0.9099
YDR227W	YKR101W	0.8965
YDR227W	YLR293C	0.9249
YDR227W	YLR442C	1.0000
YDR227W	YML109W	0.9138
YDR227W	YMR106C	0.9099
YDR227W	YMR219W	0.9667
YDR227W	YMR284W	0.8965
YDR227W	YNL186W	0.8353
YDR227W	YNL206C	0.9249
YDR227W	YNL216W	0.9993
YDR228C	YDR301W	0.8953
YDR228C	YDR381W	0.9215
YDR228C	YGL044C	1.0000
YDR228C	YHR216W	0.7380
YDR228C	YJR022W	0.5774

YDR228C	YKL059C	0.7380
YDR228C	YKR002W	0.7380
YDR228C	YLR115W	0.9021
YDR228C	YLR277C	0.9021
YDR228C	YMR061W	0.9999
YDR228C	YOR250C	0.9993
YDR229W	YMR001C	0.7380
YDR229W	YMR047C	0.5774
YDR233C	YER087C-B	0.9780
YDR233C	YFR004W	0.7380
YDR233C	YLL023C	0.9215
YDR233C	YNL044W	0.9215
YDR233C	YPL154C	0.9213
YDR235W	YDR240C	0.8659
YDR235W	YER029C	0.7380
YDR235W	YGR013W	0.9313
YDR235W	YHR086W	0.9743
YDR235W	YIL061C	0.9648
YDR235W	YKL012W	0.9648
YDR235W	YLR298C	0.8659
YDR235W	YML046W	0.8659
YDR235W	YMR125W	0.9648
YDR235W	YPR182W	0.7380
YDR237W	YDR296W	0.7380
YDR237W	YDR322W	0.7380
YDR237W	YDR462W	0.7380
YDR237W	YGR220C	0.8659
YDR237W	YML025C	0.7380
YDR237W	YNL005C	0.7380
YDR237W	YNL284C	0.8659
YDR238C	YDR372C	0.9282
YDR238C	YER122C	0.8659
YDR238C	YFR004W	0.7380
YDR238C	YFR051C	0.9887
YDR238C	YGL137W	0.9820
YDR238C	YIL076W	0.9313
YDR238C	YML067C	0.6147
YDR238C	YNL284C	0.7380
YDR238C	YNL287W	0.8659
YDR238C	YPL010W	0.8990
YDR240C	YER029C	0.8659
YDR240C	YGR013W	0.9869
YDR240C	YGR074W	0.9648
YDR240C	YHR086W	0.9917
YDR240C	YHR165C	0.7380
YDR240C	YIL061C	0.9950
YDR240C	YKL012W	0.9313

YDR240C	YLR147C	0.8659
YDR240C	YLR275W	0.7380
YDR240C	YLR298C	0.9313
YDR240C	YLR347C	0.7380
YDR240C	YML046W	0.9313
YDR240C	YMR125W	0.9640
YDR240C	YNL210W	0.8965
YDR240C	YPL178W	0.8659
YDR240C	YPR182W	0.7380
YDR244W	YDR265W	0.9780
YDR244W	YGL153W	1.0000
YDR244W	YGL205W	0.9138
YDR244W	YGR077C	0.9892
YDR244W	YJL210W	0.9707
YDR244W	YKL197C	0.9165
YDR244W	YLR191W	1.0000
YDR244W	YML042W	0.7909
YDR244W	YMR026C	0.9965
YDR244W	YNL214W	0.9979
YDR245W	YEL036C	0.9895
YDR245W	YJL183W	0.9932
YDR245W	YJR075W	0.9624
YDR245W	YPL050C	0.9624
YDR246W	YDR407C	0.8566
YDR246W	YDR472W	0.9729
YDR246W	YKR068C	1.0000
YDR246W	YML077W	0.8353
YDR246W	YOR115C	0.9157
YDR247W	YFR028C	0.8566
YDR247W	YJL005W	0.8566
YDR252W	YGR134W	0.9739
YDR252W	YNL288W	0.9191
YDR253C	YNL103W	0.9138
YDR254W	YGR140W	0.8818
YDR254W	YGR179C	0.9266
YDR254W	YIR010W	0.7380
YDR254W	YJR135C	0.7380
YDR254W	YLR315W	0.7380
YDR254W	YLR381W	0.9748
YDR254W	YPL018W	0.9748
YDR255C	YGL227W	0.8087
YDR255C	YMR135C	0.9021
YDR258C	YER151C	0.7380
YDR258C	YGL043W	0.7380
YDR258C	YGL048C	0.7380
YDR258C	YGL197W	0.7380
YDR258C	YGR094W	0.7380

YDR258C	YGR240C	0.7380
YDR258C	YHR023W	0.7380
YDR258C	YHR165C	0.7380
YDR258C	YKR008W	0.7380
YDR258C	YLR180W	0.7380
YDR258C	YLR187W	0.7380
YDR258C	YLR380W	0.7380
YDR258C	YLR418C	0.7380
YDR258C	YMR176W	0.7380
YDR258C	YOR117W	0.7380
YDR258C	YOR326W	0.7380
YDR258C	YPL138C	0.7380
YDR258C	YPL190C	0.7380
YDR259C	YLR423C	0.5774
YDR260C	YFR036W	0.9478
YDR260C	YGL240W	0.8603
YDR260C	YHR166C	0.9982
YDR260C	YIR025W	0.9973
YDR260C	YKL022C	1.0000
YDR260C	YLR102C	0.9805
YDR260C	YLR127C	0.9927
YDR260C	YNL172W	0.9944
YDR260C	YOR249C	0.9945
YDR264C	YHR135C	0.5107
YDR264C	YNL154C	0.8927
YDR264C	YOR212W	0.9861
YDR265W	YGL153W	0.8953
YDR265W	YJL210W	0.9707
YDR265W	YLR191W	0.9762
YDR265W	YMR026C	0.9989
YDR265W	YOL044W	0.9124
YDR267C	YHR122W	0.8594
YDR267C	YIL128W	0.7380
YDR277C	YDR456W	0.9138
YDR277C	YJR022W	0.5774
YDR277C	YJR090C	0.9215
YDR277C	YKL038W	0.9922
YDR280W	YDR381W	0.9215
YDR280W	YGR090W	0.7380
YDR280W	YGR095C	0.9999
YDR280W	YGR158C	0.9869
YDR280W	YGR195W	1.0000
YDR280W	YHR069C	0.9933
YDR280W	YHR081W	0.9813
YDR280W	YJL109C	0.8659
YDR280W	YLR398C	0.8659
YDR280W	YMR128W	0.8659

YDR280W	YNL189W	0.7380
YDR280W	YNL232W	0.9995
YDR280W	YNL248C	0.7380
YDR280W	YNR024W	0.8087
YDR280W	YOL021C	1.0000
YDR280W	YOL142W	0.9933
YDR280W	YOR001W	0.9995
YDR280W	YOR076C	0.9951
YDR280W	YPR189W	0.9313
YDR283C	YFR009W	0.9249
YDR283C	YGL195W	0.9999
YDR283C	YGR091W	0.7380
YDR283C	YKL173W	0.7380
YDR283C	YNL183C	0.8566
YDR283C	YOR308C	0.7380
YDR283C	YPL204W	0.9021
YDR283C	YPL240C	0.9490
YDR283C	YPR178W	0.7380
YDR285W	YDR510W	0.9138
YDR285W	YLR394W	0.9099
YDR285W	YOL069W	0.5774
YDR288W	YEL019C	0.7380
YDR288W	YLR007W	0.9889
YDR288W	YOL034W	0.8659
YDR289C	YGL246C	0.8087
YDR289C	YNL189W	0.7380
YDR289C	YOR048C	0.9021
YDR289C	YOR151C	0.8087
YDR293C	YER033C	0.9598
YDR293C	YKR028W	0.7380
YDR293C	YNL161W	0.8353
YDR293C	YNL201C	0.8087
YDR293C	YPL031C	0.6042
YDR295C	YMR117C	0.5774
YDR295C	YNL021W	0.9974
YDR295C	YOL069W	0.7253
YDR295C	YOL090W	0.7380
YDR295C	YPR179C	0.9972
YDR296W	YDR322W	0.7380
YDR296W	YDR462W	0.7380
YDR296W	YGR220C	0.9313
YDR296W	YJL063C	0.7380
YDR296W	YKL167C	0.7380
YDR296W	YKR006C	0.7380
YDR296W	YKR085C	0.7380
YDR296W	YLR312W-A	0.7380
YDR296W	YLR439W	0.7380

YDR296W	YML025C	0.8659
YDR296W	YMR024W	0.7380
YDR296W	YNL005C	0.7380
YDR296W	YNL177C	0.7380
YDR296W	YNL252C	0.7380
YDR296W	YNL284C	0.7380
YDR296W	YOR150W	0.7380
YDR298C	YJR121W	0.9960
YDR298C	YPL078C	0.9574
YDR299W	YDR365C	0.7380
YDR299W	YER127W	0.8893
YDR299W	YGR090W	0.8659
YDR299W	YGR145W	0.7380
YDR299W	YJL033W	0.7380
YDR299W	YNL132W	0.7380
YDR301W	YER133W	0.9982
YDR301W	YGR156W	0.9313
YDR301W	YJR093C	0.9928
YDR301W	YKL018W	0.9499
YDR301W	YKL059C	0.9933
YDR301W	YKR002W	0.9986
YDR301W	YLR115W	0.9992
YDR301W	YLR277C	0.9950
YDR301W	YMR061W	0.9906
YDR301W	YNL317W	0.9996
YDR301W	YPR107C	0.9808
YDR303C	YFR037C	0.9950
YDR303C	YGR056W	0.9787
YDR303C	YGR275W	0.8659
YDR303C	YHR056C	0.9266
YDR303C	YIL126W	0.9992
YDR303C	YKR001C	0.7380
YDR303C	YKR008W	0.9648
YDR303C	YLR033W	0.9908
YDR303C	YLR321C	0.9648
YDR303C	YLR357W	1.0000
YDR303C	YML127W	0.9820
YDR303C	YMR033W	0.9648
YDR303C	YMR091C	0.9974
YDR303C	YPL082C	0.9021
YDR303C	YPL106C	0.8659
YDR303C	YPR034W	0.9820
YDR306C	YDR328C	0.8628
YDR308C	YDR443C	0.7380
YDR308C	YEL009C	0.9780
YDR308C	YER022W	0.9839
YDR308C	YGL025C	0.9866

YDR308C	YGL127C	0.9452
YDR308C	YGL151W	0.8659
YDR308C	YGR104C	0.9800
YDR308C	YHR058C	0.9980
YDR308C	YLR071C	0.9989
YDR308C	YNL236W	0.8659
YDR308C	YNR010W	0.8566
YDR308C	YOL051W	0.7380
YDR308C	YOL135C	1.0000
YDR308C	YOR174W	0.9999
YDR308C	YPL042C	0.8566
YDR308C	YPL248C	0.9151
YDR308C	YPR070W	0.9794
YDR308C	YPR168W	1.0000
YDR309C	YHR061C	0.5774
YDR309C	YHR107C	0.9440
YDR309C	YJR076C	0.9099
YDR309C	YKL007W	0.5774
YDR309C	YLR229C	1.0000
YDR310C	YOL068C	0.9984
YDR310C	YOR279C	0.8566
YDR311W	YDR337W	0.7380
YDR311W	YDR460W	0.8566
YDR311W	YER171W	1.0000
YDR311W	YGR120C	0.5774
YDR311W	YGR258C	0.9574
YDR311W	YKL028W	0.8297
YDR311W	YKL103C	0.5774
YDR311W	YLR005W	0.9997
YDR311W	YOL082W	0.5774
YDR311W	YPL122C	0.9406
YDR311W	YPR025C	0.9186
YDR311W	YPR056W	0.9997
YDR313C	YLL039C	0.7380
YDR318W	YGR179C	0.9990
YDR318W	YIR010W	0.8566
YDR318W	YJR135C	0.7380
YDR318W	YKL089W	0.9099
YDR318W	YLR315W	0.7380
YDR318W	YLR381W	0.7380
YDR318W	YPL018W	0.9756
YDR318W	YPR046W	0.9021
YDR320C	YEL032W	0.7380
YDR320C	YGL195W	0.7380
YDR320C	YGL206C	0.9249
YDR320C	YGR155W	0.7380
YDR320C	YGR167W	0.9215

YDR320C	YHR165C	0.7380
YDR320C	YJR135C	0.7380
YDR320C	YPL226W	0.7380
YDR320C-A	YGL061C	0.6672
YDR320C-A	YKL052C	0.9958
YDR320C-A	YKR037C	0.9916
YDR320C-A	YKR083C	0.5774
YDR322W	YDR405W	0.7380
YDR322W	YDR462W	0.8659
YDR322W	YGR091W	0.7380
YDR322W	YGR220C	0.8659
YDR322W	YJL041W	0.7380
YDR322W	YLR312W-A	0.7380
YDR322W	YML025C	0.7380
YDR322W	YNL005C	0.7380
YDR322W	YNL252C	0.7380
YDR322W	YNL284C	0.8659
YDR323C	YGL095C	0.9811
YDR323C	YLR148W	0.9099
YDR323C	YMR231W	0.9099
YDR323C	YOR089C	0.9907
YDR324C	YDR398W	0.9796
YDR324C	YDR449C	0.8659
YDR324C	YEL055C	0.8659
YDR324C	YER082C	0.9313
YDR324C	YGR090W	0.9313
YDR324C	YGR128C	0.9999
YDR324C	YHR196W	0.9953
YDR324C	YJL069C	0.9483
YDR324C	YJL109C	0.9965
YDR324C	YJR002W	0.8087
YDR324C	YLR409C	0.8659
YDR324C	YMR093W	0.9908
YDR324C	YMR128W	0.8659
YDR324C	YMR229C	0.7380
YDR324C	YNL075W	0.7380
YDR324C	YOR078W	0.7380
YDR324C	YOR310C	0.9021
YDR324C	YPL126W	0.9908
YDR324C	YPR137W	0.8659
YDR324C	YPR144C	0.9313
YDR325W	YFR031C	0.9266
YDR325W	YLR086W	0.8566
YDR325W	YLR272C	0.9598
YDR328C	YFL009W	1.0000
YDR328C	YGR188C	0.9790
YDR328C	YIL046W	1.0000

YDR328C	YJL149W	0.9616
YDR328C	YJL204C	0.9989
YDR328C	YJR033C	0.9934
YDR328C	YJR090C	1.0000
YDR328C	YKR014C	0.8659
YDR328C	YLL024C	0.8566
YDR328C	YLR097C	0.9907
YDR328C	YLR224W	0.8258
YDR328C	YLR267W	0.6672
YDR328C	YLR352W	0.6672
YDR328C	YLR368W	0.8867
YDR328C	YML088W	0.9979
YDR328C	YMR094W	1.0000
YDR328C	YMR168C	0.9719
YDR328C	YMR258C	0.9946
YDR328C	YNL311C	0.9298
YDR328C	YOL025W	0.9249
YDR328C	YOL133W	0.9707
YDR328C	YOR057W	1.0000
YDR328C	YOR080W	0.9965
YDR328C	YPL256C	0.8566
YDR329C	YMR204C	0.9934
YDR329C	YOL147C	0.7826
YDR334W	YDR485C	0.9997
YDR334W	YFL039C	1.0000
YDR334W	YGR002C	1.0000
YDR334W	YJL081C	1.0000
YDR334W	YLR085C	0.9999
YDR334W	YLR385C	0.9860
YDR334W	YLR399C	0.9978
YDR334W	YML041C	0.9997
YDR334W	YNL107W	1.0000
YDR334W	YOL012C	1.0000
YDR334W	YPL235W	0.9998
YDR335W	YER103W	0.9742
YDR335W	YFR034C	0.9946
YDR335W	YGL071W	0.9099
YDR335W	YGR128C	0.9921
YDR335W	YHR196W	0.9215
YDR335W	YJL157C	0.9710
YDR335W	YKL068W	0.8087
YDR335W	YLR293C	0.9512
YDR335W	YMR047C	0.9577
YDR337W	YGL129C	0.7380
YDR337W	YGR165W	0.7380
YDR337W	YHL004W	0.9499
YDR337W	YJR113C	0.7380

YDR337W	YKL155C	0.9266
YDR337W	YNL137C	0.7380
YDR337W	YNL306W	0.7380
YDR337W	YPL013C	0.7380
YDR341C	YFR004W	0.7380
YDR345C	YFR004W	0.7380
YDR346C	YIL035C	0.8655
YDR346C	YOR061W	0.8655
YDR347W	YGL129C	0.7380
YDR347W	YGR165W	0.7380
YDR347W	YHL004W	0.9743
YDR347W	YIL093C	0.7380
YDR347W	YJR101W	0.7380
YDR347W	YKL155C	0.8566
YDR347W	YNL137C	0.7380
YDR347W	YOR158W	0.7380
YDR347W	YPL013C	0.7380
YDR347W	YPL118W	0.7380
YDR356W	YHR172W	1.0000
YDR356W	YIL149C	0.8953
YDR356W	YKL042W	0.9999
YDR356W	YLL003W	0.8953
YDR356W	YLR212C	1.0000
YDR356W	YNL126W	0.9999
YDR356W	YNL225C	0.9963
YDR356W	YOR373W	0.9555
YDR356W	YPL124W	0.9997
YDR357C	YGL079W	0.5774
YDR357C	YKL061W	0.7380
YDR358W	YGL206C	0.9249
YDR358W	YLL039C	0.9539
YDR359C	YEL018W	0.9946
YDR359C	YFL024C	1.0000
YDR359C	YFL039C	0.9598
YDR359C	YGR002C	0.9997
YDR359C	YHR090C	0.9946
YDR359C	YHR099W	0.9871
YDR359C	YJL081C	0.9961
YDR359C	YJL098W	0.7380
YDR359C	YJR082C	0.8659
YDR359C	YML007W	0.7380
YDR359C	YNL107W	0.9992
YDR359C	YNL136W	0.9820
YDR359C	YOR244W	0.9999
YDR359C	YPR023C	0.9808
YDR361C	YER117W	0.7380
YDR361C	YPL208W	0.9554

YDR362C	YGR047C	0.9266
YDR362C	YOR110W	0.8659
YDR362C	YPL007C	0.9612
YDR362C	YPL106C	0.7380
YDR363W	YJL047C	0.9215
YDR363W	YPR164W	0.9747
YDR363W-A	YDR394W	0.9021
YDR363W-A	YDR427W	0.9923
YDR363W-A	YER021W	0.9021
YDR363W-A	YFR004W	0.9992
YDR363W-A	YFR010W	0.7380
YDR363W-A	YFR052W	0.9021
YDR363W-A	YGL048C	0.9021
YDR363W-A	YGR232W	0.8566
YDR363W-A	YHR027C	0.9923
YDR363W-A	YHR200W	0.9021
YDR363W-A	YIL075C	0.9726
YDR363W-A	YJR084W	0.9453
YDR363W-A	YKL145W	0.9726
YDR363W-A	YLR421C	0.7380
YDR363W-A	YOL072W	0.9730
YDR363W-A	YOR117W	0.9726
YDR363W-A	YOR259C	0.9425
YDR363W-A	YOR261C	0.9021
YDR363W-A	YPR045C	0.6996
YDR363W-A	YPR108W	0.9923
YDR364C	YLL036C	0.9554
YDR364C	YMR213W	0.8659
YDR364C	YPR101W	0.7380
YDR365C	YDR381W	0.7380
YDR365C	YGR145W	0.8659
YDR365C	YHR089C	0.7380
YDR365C	YJL033W	0.8659
YDR365C	YLR340W	0.8087
YDR365C	YNR054C	0.9499
YDR369C	YGL090W	0.9975
YDR369C	YMR224C	1.0000
YDR369C	YNL250W	0.9983
YDR372C	YDR483W	0.9769
YDR372C	YFR051C	0.9282
YDR373W	YER177W	0.8953
YDR373W	YNL267W	1.0000
YDR377W	YJR121W	0.9960
YDR377W	YPL078C	0.9886
YDR378C	YDR473C	0.9648
YDR378C	YER112W	0.9994
YDR378C	YER146W	0.9941

YDR378C	YER172C	0.9993
YDR378C	YGL173C	0.9313
YDR378C	YGR091W	0.9648
YDR378C	YHR165C	0.9743
YDR378C	YJL124C	1.0000
YDR378C	YJR022W	0.9979
YDR378C	YKL173W	0.7380
YDR378C	YLR147C	0.7380
YDR378C	YLR275W	0.8297
YDR378C	YLR438C-A	0.9997
YDR378C	YMR268C	0.9878
YDR378C	YNL147W	0.9984
YDR378C	YOR308C	0.7380
YDR378C	YPR082C	0.7380
YDR378C	YPR178W	0.8659
YDR379W	YNL201C	0.7380
YDR379W	YNL307C	0.5779
YDR381W	YDR432W	0.8087
YDR381W	YER110C	0.9021
YDR381W	YER165W	0.8087
YDR381W	YGL049C	0.8087
YDR381W	YGL099W	0.8087
YDR381W	YGL122C	0.9991
YDR381W	YGL173C	0.8087
YDR381W	YGR162W	0.8087
YDR381W	YHR127W	0.9021
YDR381W	YHR167W	0.8659
YDR381W	YIL149C	0.8566
YDR381W	YJL006C	0.7380
YDR381W	YKL214C	0.8087
YDR381W	YKR048C	0.9961
YDR381W	YKR095W	0.8566
YDR381W	YML010W	0.7380
YDR381W	YML062C	0.9808
YDR381W	YML065W	0.7380
YDR381W	YML112W	0.7380
YDR381W	YMR237W	0.8087
YDR381W	YMR308C	0.9866
YDR381W	YNL004W	0.9021
YDR381W	YNL061W	0.8087
YDR381W	YNL088W	0.7380
YDR381W	YNL112W	0.8087
YDR381W	YNL139C	0.9266
YDR381W	YNL308C	0.8087
YDR381W	YNR051C	0.7380
YDR381W	YOR080W	0.8953
YDR381W	YPL169C	0.9992

YDR381W	YPL178W	0.8566
YDR382W	YLR340W	0.9539
YDR382W	YOL039W	0.9539
YDR383C	YGR179C	0.9624
YDR383C	YIR010W	0.7380
YDR383C	YJR135C	0.7380
YDR383C	YLR315W	0.9578
YDR383C	YPR046W	0.8087
YDR385W	YDR510W	0.8659
YDR385W	YFR004W	0.7380
YDR385W	YGR187C	0.7380
YDR385W	YIL103W	0.7380
YDR385W	YKL191W	0.8028
YDR385W	YOR133W	0.7380
YDR386W	YGL163C	0.9758
YDR388W	YER125W	0.9984
YDR388W	YFL039C	0.9327
YDR388W	YGL060W	0.8640
YDR388W	YGL252C	0.7380
YDR388W	YIL156W	0.6672
YDR388W	YJR083C	0.9084
YDR388W	YLL039C	0.7380
YDR388W	YLR144C	0.9958
YDR388W	YLR337C	0.6819
YDR388W	YMR109W	0.9619
YDR388W	YMR192W	0.9998
YDR388W	YMR287C	0.7909
YDR388W	YNL094W	0.9627
YDR388W	YOR181W	1.0000
YDR388W	YPL031C	0.7696
YDR388W	YPL249C	0.9999
YDR388W	YPL249C-A	0.8353
YDR388W	YPR055W	0.7909
YDR388W	YPR171W	0.9084
YDR389W	YPR165W	0.9758
YDR390C	YDR510W	0.9899
YDR390C	YKR002W	0.9099
YDR390C	YPL106C	0.7380
YDR390C	YPR180W	0.9816
YDR392W	YDR448W	1.0000
YDR392W	YEL009C	0.9187
YDR392W	YER148W	1.0000
YDR392W	YGL066W	0.7380
YDR392W	YGL112C	0.9986
YDR392W	YGR252W	0.9624
YDR392W	YHR099W	0.9895
YDR392W	YLR055C	0.9979

YDR392W	YMR223W	0.9860
YDR392W	YMR236W	0.9787
YDR392W	YOL148C	0.9948
YDR392W	YPL047W	0.9285
YDR392W	YPL248C	0.8958
YDR392W	YPL254W	0.9860
YDR394W	YDR427W	0.9779
YDR394W	YEL037C	0.9266
YDR394W	YER012W	0.8087
YDR394W	YER021W	0.9583
YDR394W	YER095W	0.7380
YDR394W	YER171W	0.7380
YDR394W	YFR004W	0.9902
YDR394W	YFR010W	0.9648
YDR394W	YFR052W	0.9313
YDR394W	YGL004C	0.9266
YDR394W	YGL048C	0.9981
YDR394W	YGR232W	1.0000
YDR394W	YHL030W	0.7380
YDR394W	YHR027C	0.9998
YDR394W	YHR200W	0.9933
YDR394W	YIL007C	0.9215
YDR394W	YIL075C	0.7380
YDR394W	YJR109C	0.7380
YDR394W	YKL145W	0.9993
YDR394W	YLR421C	0.7380
YDR394W	YNL250W	0.7380
YDR394W	YOL038W	0.7380
YDR394W	YOR117W	0.9996
YDR394W	YOR259C	0.9975
YDR394W	YOR261C	0.8659
YDR394W	YPR108W	0.9624
YDR395W	YKL068W	0.8087
YDR395W	YNL069C	0.8087
YDR397C	YER148W	0.9981
YDR397C	YER159C	0.9932
YDR397C	YPL082C	0.9972
YDR398W	YGR128C	0.9623
YDR398W	YHR196W	0.8990
YDR398W	YJL109C	0.9675
YDR398W	YJR002W	0.8953
YDR398W	YMR093W	0.9867
YDR398W	YNR051C	0.7380
YDR398W	YPL126W	0.9266
YDR404C	YER022W	0.9574
YDR404C	YER125W	0.8566
YDR404C	YER139C	0.8603

YDR404C	YGL070C	0.9989
YDR404C	YGR005C	0.9624
YDR404C	YGR063C	0.8659
YDR404C	YGR186W	0.8659
YDR404C	YIL021W	1.0000
YDR404C	YJL140W	1.0000
YDR404C	YML010W	0.9648
YDR404C	YOL005C	0.9904
YDR404C	YOR151C	0.9980
YDR404C	YOR210W	0.9021
YDR404C	YOR224C	0.9930
YDR404C	YPL129W	0.7380
YDR404C	YPR180W	0.7380
YDR404C	YPR187W	0.9726
YDR405W	YER154W	0.9215
YDR405W	YGR220C	0.7380
YDR405W	YKR006C	0.8566
YDR405W	YLR439W	0.9021
YDR405W	YML025C	0.7380
YDR405W	YMR024W	0.9021
YDR405W	YNL005C	0.7380
YDR405W	YPL173W	0.9464
YDR407C	YDR472W	0.9266
YDR407C	YGR166W	0.9988
YDR407C	YKR068C	1.0000
YDR407C	YML077W	0.9892
YDR407C	YMR218C	0.9598
YDR407C	YOR115C	0.9981
YDR409W	YDR510W	0.9990
YDR409W	YLR314C	0.9138
YDR411C	YML029W	0.7570
YDR412W	YER133W	0.6672
YDR412W	YMR047C	0.9186
YDR412W	YMR049C	0.7380
YDR412W	YNL110C	0.7380
YDR415C	YKL210W	0.7380
YDR416W	YER172C	0.7380
YDR416W	YGR129W	0.9987
YDR416W	YGR278W	0.7380
YDR416W	YHR165C	0.9648
YDR416W	YJR050W	1.0000
YDR416W	YKL095W	0.9903
YDR416W	YLL036C	1.0000
YDR416W	YLR117C	0.9997
YDR416W	YLR424W	0.9266
YDR416W	YMR213W	0.9990
YDR416W	YPL151C	0.7380

YDR416W	YPL213W	0.9021
YDR416W	YPR101W	0.9794
YDR418W	YFR004W	0.7380
YDR418W	YHR170W	0.9215
YDR418W	YLR075W	0.8566
YDR418W	YLR340W	0.8566
YDR419W	YOR346W	0.9215
YDR422C	YDR477W	1.0000
YDR422C	YER129W	0.7380
YDR422C	YGL115W	0.9942
YDR423C	YGL158W	0.9099
YDR423C	YLR109W	0.9099
YDR424C	YDR488C	0.8297
YDR424C	YIL115C	0.8998
YDR425W	YJL036W	0.9996
YDR427W	YEL037C	0.9266
YDR427W	YER021W	0.8659
YDR427W	YFR004W	0.9995
YDR427W	YFR010W	0.9648
YDR427W	YFR052W	0.9902
YDR427W	YGL004C	0.8566
YDR427W	YGL048C	0.9808
YDR427W	YGR232W	0.9860
YDR427W	YHL030W	0.8659
YDR427W	YHR027C	0.9944
YDR427W	YHR200W	0.9994
YDR427W	YIL075C	0.8659
YDR427W	YKL145W	0.9902
YDR427W	YLR421C	0.9313
YDR427W	YMR191W	0.7380
YDR427W	YMR314W	0.7380
YDR427W	YOR117W	0.9624
YDR427W	YOR259C	0.8659
YDR427W	YOR261C	0.9984
YDR427W	YPR108W	0.9808
YDR429C	YER025W	0.9586
YDR429C	YLR192C	0.9944
YDR429C	YMR012W	0.9138
YDR429C	YMR146C	1.0000
YDR429C	YMR309C	0.9972
YDR429C	YNL244C	0.9899
YDR429C	YOR204W	0.8659
YDR429C	YOR361C	1.0000
YDR429C	YPR041W	0.9743
YDR430C	YER178W	0.7380
YDR430C	YFL018C	0.7380
YDR430C	YGR193C	0.7380

YDR430C	YNL071W	0.7380
YDR432W	YER125W	0.8714
YDR432W	YGL122C	0.3858
YDR432W	YGL173C	0.8603
YDR432W	YGL207W	0.8953
YDR432W	YHR086W	0.9266
YDR432W	YIL021W	0.9215
YDR432W	YIL061C	0.9215
YDR432W	YIL079C	0.9108
YDR432W	YKL139W	0.9021
YDR432W	YMR125W	0.9897
YDR432W	YMR216C	0.9982
YDR432W	YNL016W	0.8087
YDR432W	YOL123W	0.8953
YDR432W	YOR001W	0.9099
YDR432W	YOR048C	0.8603
YDR432W	YOR160W	0.9984
YDR432W	YOR204W	0.8603
YDR432W	YPL106C	0.7380
YDR432W	YPL169C	0.8628
YDR432W	YPL178W	0.8659
YDR436W	YKL088W	0.6147
YDR436W	YKL193C	0.8659
YDR436W	YKR072C	0.6147
YDR436W	YML016C	0.7380
YDR436W	YMR311C	0.7380
YDR436W	YOR054C	0.7380
YDR439W	YER106W	0.8603
YDR439W	YFR028C	0.8087
YDR439W	YJL076W	0.8603
YDR439W	YKR010C	0.8603
YDR439W	YML034W	0.9478
YDR439W	YPL204W	0.8603
YDR440W	YLL039C	0.8940
YDR440W	YNL031C	0.9966
YDR443C	YER022W	0.9685
YDR443C	YGL043W	0.9099
YDR443C	YGL151W	0.7380
YDR443C	YGR104C	0.9897
YDR443C	YKR036C	0.9099
YDR443C	YLR071C	0.8659
YDR443C	YNL236W	0.7380
YDR443C	YOL135C	0.8659
YDR443C	YPL042C	0.9266
YDR443C	YPR070W	0.8659
YDR446W	YDR510W	0.7253
YDR447C	YFR004W	0.7380

YDR447C	YGL246C	0.7380
YDR447C	YGR214W	0.7380
YDR447C	YJL109C	0.7380
YDR447C	YNL132W	0.7380
YDR447C	YOR310C	0.7380
YDR447C	YPR144C	0.7380
YDR448W	YEL009C	0.9944
YDR448W	YER018C	0.5774
YDR448W	YER148W	0.8566
YDR448W	YGL066W	1.0000
YDR448W	YGL112C	1.0000
YDR448W	YGR252W	1.0000
YDR448W	YGR274C	0.9640
YDR448W	YHR099W	1.0000
YDR448W	YLR055C	1.0000
YDR448W	YML007W	0.7380
YDR448W	YMR223W	1.0000
YDR448W	YMR236W	0.9996
YDR448W	YOL072W	0.8566
YDR448W	YOL135C	0.8659
YDR448W	YOL148C	1.0000
YDR448W	YOR023C	0.9574
YDR448W	YPL047W	0.9993
YDR448W	YPL248C	0.9799
YDR448W	YPL254W	1.0000
YDR448W	YPR070W	0.7380
YDR449C	YER082C	0.9820
YDR449C	YGL171W	0.8659
YDR449C	YGR090W	0.9820
YDR449C	YGR128C	0.9021
YDR449C	YGR145W	0.8659
YDR449C	YHR148W	0.8659
YDR449C	YHR196W	0.9648
YDR449C	YJL069C	0.9953
YDR449C	YJL109C	0.9648
YDR449C	YJR002W	0.9828
YDR449C	YLL011W	0.8659
YDR449C	YLR129W	0.9313
YDR449C	YLR186W	0.8659
YDR449C	YLR222C	0.9648
YDR449C	YLR409C	0.9990
YDR449C	YLR441C	0.7380
YDR449C	YMR093W	0.8659
YDR449C	YMR229C	0.7380
YDR449C	YNL132W	0.8659
YDR449C	YOR310C	0.9021
YDR449C	YPL126W	0.8659

YDR449C	YPR137W	0.9648
YDR449C	YPR144C	0.9648
YDR450W	YFR004W	0.7380
YDR450W	YHR027C	0.7380
YDR450W	YHR200W	0.8087
YDR453C	YML028W	0.6672
YDR456W	YJL044C	0.9099
YDR457W	YLL039C	0.9598
YDR460W	YER171W	0.9999
YDR460W	YLR005W	0.9266
YDR460W	YPR025C	0.9994
YDR460W	YPR056W	0.9967
YDR462W	YGR091W	0.7380
YDR462W	YGR220C	0.8659
YDR462W	YNL005C	0.7380
YDR462W	YNL252C	0.7380
YDR462W	YNL284C	0.9313
YDR464W	YDR510W	0.7380
YDR465C	YGL172W	0.9215
YDR465C	YGR119C	0.9215
YDR465C	YKL068W	0.9215
YDR468C	YGL095C	0.9598
YDR468C	YGR009C	0.6000
YDR468C	YKR020W	0.9998
YDR468C	YMR197C	0.9997
YDR468C	YOL018C	0.9999
YDR468C	YOR036W	0.9881
YDR468C	YOR327C	0.9249
YDR469W	YDR510W	0.7380
YDR469W	YHR119W	1.0000
YDR469W	YKL018W	0.7380
YDR469W	YLR015W	1.0000
YDR469W	YPL138C	0.9634
YDR470C	YOR211C	0.9938
YDR471W	YFR004W	0.7380
YDR471W	YNL110C	0.7380
YDR472W	YEL048C	0.9707
YDR472W	YGR166W	0.7380
YDR472W	YKR068C	1.0000
YDR472W	YML077W	0.9470
YDR472W	YMR218C	0.7380
YDR472W	YOR115C	0.9875
YDR473C	YER029C	0.7380
YDR473C	YER112W	0.8659
YDR473C	YER172C	0.9998
YDR473C	YFL017W-A	0.7380
YDR473C	YGR074W	0.7380

YDR473C	YGR075C	0.7380
YDR473C	YGR091W	0.9648
YDR473C	YHR165C	0.9869
YDR473C	YJR022W	0.8087
YDR473C	YKL173W	0.9648
YDR473C	YLR147C	0.7380
YDR473C	YLR275W	0.7380
YDR473C	YLR423C	0.5774
YDR473C	YLR438C-A	0.9021
YDR473C	YOR308C	0.8659
YDR473C	YPR082C	0.7380
YDR473C	YPR178W	0.9997
YDR475C	YER133W	0.8659
YDR477W	YER027C	0.9999
YDR477W	YER040W	0.9567
YDR477W	YER129W	1.0000
YDR477W	YFR028C	0.8566
YDR477W	YGL035C	0.9995
YDR477W	YGL073W	0.9259
YDR477W	YGL115W	1.0000
YDR477W	YGL208W	1.0000
YDR477W	YGL253W	0.9215
YDR477W	YGR252W	0.8714
YDR477W	YJL089W	0.9990
YDR477W	YKL139W	0.9099
YDR477W	YLL039C	0.9021
YDR477W	YLR182W	0.9215
YDR477W	YML006C	0.8566
YDR477W	YMR140W	0.9099
YDR477W	YNL025C	0.9099
YDR477W	YNL183C	0.8566
YDR477W	YNL236W	0.9099
YDR477W	YNL257C	0.8965
YDR477W	YOR047C	0.9976
YDR477W	YPL042C	0.9099
YDR477W	YPL133C	0.6042
YDR478W	YHR062C	0.8353
YDR478W	YLR145W	0.9598
YDR478W	YNL221C	0.9979
YDR479C	YLR324W	0.9563
YDR480W	YGR040W	0.9988
YDR480W	YHR084W	1.0000
YDR480W	YNL189W	0.6672
YDR480W	YPL049C	0.8816
YDR482C	YHR165C	0.9539
YDR482C	YMR213W	0.7380
YDR482C	YPL151C	0.7380

YDR483W	YLR026C	0.9747
YDR484W	YJL029C	0.9998
YDR484W	YKR020W	0.9968
YDR484W	YLR262C	0.9921
YDR485C	YDR510W	0.8566
YDR485C	YJL081C	0.8087
YDR485C	YNL107W	0.9850
YDR485C	YOL012C	0.9961
YDR485C	YPL235W	0.7380
YDR486C	YLR025W	0.5107
YDR486C	YLR181C	0.9983
YDR488C	YPL174C	0.7253
YDR489W	YGL207W	0.8953
YDR489W	YJL072C	0.9996
YDR489W	YLR103C	0.9707
YDR489W	YMR048W	0.9707
YDR489W	YOL146W	0.9738
YDR489W	YPL077C	0.5774
YDR489W	YPR019W	0.9890
YDR489W	YPR135W	0.9997
YDR490C	YGR086C	0.9685
YDR490C	YKL126W	0.7696
YDR490C	YPL004C	0.8742
YDR495C	YLR396C	0.8953
YDR495C	YMR231W	0.8953
YDR496C	YER006W	0.7380
YDR496C	YFL002C	0.7380
YDR496C	YGL111W	0.9021
YDR496C	YHR052W	0.9313
YDR496C	YHR066W	0.7380
YDR496C	YHR088W	0.7380
YDR496C	YKL014C	0.7380
YDR496C	YKL130C	0.9215
YDR496C	YKL185W	0.5293
YDR496C	YKR081C	0.8659
YDR496C	YLL008W	0.7380
YDR496C	YLL034C	0.7380
YDR496C	YLL045C	0.7380
YDR496C	YLR175W	0.8659
YDR496C	YLR276C	0.7380
YDR496C	YLR449W	0.8659
YDR496C	YMR049C	0.7380
YDR496C	YMR229C	0.7380
YDR496C	YMR242C	0.7380
YDR496C	YMR290C	0.7380
YDR496C	YNL002C	0.7380
YDR496C	YNL061W	0.8659

YDR496C	YNL175C	0.8659
YDR496C	YOL077C	0.7380
YDR496C	YOL127W	0.7380
YDR496C	YOR063W	0.7380
YDR496C	YOR272W	0.7380
YDR496C	YPL012W	0.7380
YDR496C	YPL043W	0.9313
YDR496C	YPL093W	0.9021
YDR496C	YPL131W	0.7380
YDR496C	YPL198W	0.7380
YDR496C	YPL211W	0.7380
YDR496C	YPL220W	0.7380
YDR496C	YPR016C	0.9499
YDR498C	YGL098W	0.9887
YDR498C	YGL145W	0.9967
YDR498C	YLR268W	0.8566
YDR498C	YLR440C	0.9598
YDR498C	YNL258C	0.9598
YDR498C	YOR075W	0.9887
YDR499W	YGR218W	0.9138
YDR499W	YHR164C	0.7380
YDR502C	YFR004W	0.7380
YDR502C	YLL039C	0.8566
YDR502C	YLR180W	0.9128
YDR505C	YGL122C	0.3858
YDR507C	YHR107C	0.9794
YDR507C	YJR076C	0.9598
YDR507C	YKL101W	0.6147
YDR507C	YKR048C	0.9996
YDR507C	YLR314C	0.9871
YDR507C	YMR139W	0.8659
YDR507C	YNL166C	0.8566
YDR507C	YOR231W	0.8566
YDR510W	YER032W	0.9700
YDR510W	YER047C	0.7837
YDR510W	YER116C	0.8456
YDR510W	YER120W	0.7380
YDR510W	YFL039C	0.8297
YDR510W	YFL045C	0.6672
YDR510W	YFR028C	0.8566
YDR510W	YFR037C	0.7380
YDR510W	YGL009C	0.7380
YDR510W	YGL026C	0.8297
YDR510W	YGL157W	0.7380
YDR510W	YGL250W	0.5774
YDR510W	YGR048W	0.6672
YDR510W	YGR140W	0.9778

YDR510W	YGR186W	0.9313
YDR510W	YGR192C	0.8659
YDR510W	YGR214W	0.7380
YDR510W	YGR254W	0.7380
YDR510W	YGR264C	0.7380
YDR510W	YHR084W	0.8566
YDR510W	YHR134W	0.9432
YDR510W	YHR187W	0.8566
YDR510W	YHR193C	0.6672
YDR510W	YIL036W	0.7380
YDR510W	YIL053W	0.8659
YDR510W	YJL092W	0.9304
YDR510W	YJL138C	0.7380
YDR510W	YJL140W	0.9266
YDR510W	YJR076C	0.9940
YDR510W	YJR089W	0.9266
YDR510W	YJR104C	0.9266
YDR510W	YKL032C	0.6672
YDR510W	YKL043W	0.5774
YDR510W	YKL060C	0.8659
YDR510W	YKL104C	0.7380
YDR510W	YKL112W	0.7380
YDR510W	YKL142W	0.7380
YDR510W	YKL152C	0.7380
YDR510W	YKL172W	0.8566
YDR510W	YKL211C	0.6672
YDR510W	YKR025W	0.7380
YDR510W	YKR062W	0.7380
YDR510W	YLR033W	0.9624
YDR510W	YLR044C	0.7380
YDR510W	YLR086W	0.7380
YDR510W	YLR150W	0.8659
YDR510W	YLR175W	0.7380
YDR510W	YLR276C	0.7380
YDR510W	YLR277C	0.7380
YDR510W	YLR314C	0.9974
YDR510W	YLR328W	0.6672
YDR510W	YLR335W	0.8297
YDR510W	YLR350W	0.6672
YDR510W	YLR357W	0.8566
YDR510W	YML010W	0.7380
YDR510W	YML023C	0.5774
YDR510W	YML032C	0.9790
YDR510W	YML069W	0.9266
YDR510W	YML074C	0.7380
YDR510W	YML126C	0.7380
YDR510W	YMR111C	0.9128

YDR510W	YMR186W	0.9313
YDR510W	YMR190C	0.8353
YDR510W	YMR219W	0.6672
YDR510W	YMR233W	0.6672
YDR510W	YNL021W	0.6672
YDR510W	YNL030W	0.8566
YDR510W	YNL042W	0.9128
YDR510W	YNL061W	0.8566
YDR510W	YNL078W	0.9619
YDR510W	YNL088W	0.9921
YDR510W	YNL167C	0.7380
YDR510W	YNL178W	0.7380
YDR510W	YNL189W	0.5774
YDR510W	YNL216W	0.7380
YDR510W	YOL006C	0.7380
YDR510W	YOL034W	0.8297
YDR510W	YOL086C	0.8659
YDR510W	YOR032C	0.6672
YDR510W	YOR144C	0.6672
YDR510W	YOR156C	0.8929
YDR510W	YOR191W	0.9846
YDR510W	YOR194C	0.8659
YDR510W	YOR207C	0.7380
YDR510W	YOR290C	0.7380
YDR510W	YOR340C	0.7380
YDR510W	YOR341W	0.8659
YDR510W	YPL020C	0.9942
YDR510W	YPL106C	0.7380
YDR510W	YPL129W	0.8297
YDR510W	YPL228W	0.8659
YDR510W	YPL240C	0.7380
YDR510W	YPL269W	0.9099
YDR510W	YPR103W	0.8566
YDR510W	YPR110C	0.7380
YDR510W	YPR180W	0.9899
YDR510W	YPR183W	0.7380
YDR510W	YPR190C	0.7380
YDR517W	YIL109C	0.9623
YDR517W	YPR181C	0.9499
YDR527W	YOR210W	0.5774
YDR532C	YGL093W	0.9960
YDR532C	YIL144W	0.5774
YDR533C	YPL240C	0.6672
YEL002C	YER087C-B	0.9742
YEL002C	YGL022W	0.9999
YEL002C	YGL226C-A	0.9762
YEL002C	YJL002C	1.0000

YEL002C	YML019W	0.9997
YEL002C	YMR149W	1.0000
YEL002C	YOR085W	0.9989
YEL002C	YOR103C	0.9762
YEL003W	YGR078C	0.9808
YEL003W	YJL179W	0.7380
YEL003W	YLR200W	0.9808
YEL003W	YML094W	0.9266
YEL003W	YNL153C	0.9313
YEL005C	YEL013W	0.9099
YEL005C	YGL079W	0.6672
YEL005C	YNL086W	0.6672
YEL009C	YER022W	0.9215
YEL009C	YER040W	0.9037
YEL009C	YGL025C	0.9703
YEL009C	YGL112C	0.9215
YEL009C	YHR041C	0.9780
YEL009C	YHR200W	0.9799
YEL009C	YMR236W	0.9780
YEL009C	YNR023W	0.9215
YEL009C	YOL051W	0.9981
YEL009C	YOR290C	0.9918
YEL009C	YOR298C-A	0.9249
YEL009C	YPL016W	0.9215
YEL009C	YPL031C	0.8894
YEL013W	YHR195W	0.9099
YEL013W	YKL196C	0.9808
YEL013W	YKR007W	0.7826
YEL013W	YNL326C	0.9208
YEL013W	YOR106W	0.9249
YEL013W	YPR185W	0.9974
YEL015W	YGL173C	0.6672
YEL015W	YJR022W	0.7253
YEL015W	YNL118C	1.0000
YEL015W	YOL149W	0.9952
YEL015W	YPR129W	0.6672
YEL018W	YFL024C	0.9985
YEL018W	YFL039C	0.8566
YEL018W	YGR002C	0.9808
YEL018W	YHR090C	0.9794
YEL018W	YHR099W	0.9624
YEL018W	YJL081C	0.9726
YEL018W	YJR082C	0.7380
YEL018W	YNL107W	0.9726
YEL018W	YNL136W	0.9313
YEL018W	YOR244W	0.9983
YEL018W	YPR023C	0.9624

YEL019C	YOL034W	0.9987
YEL020W-A	YHR005C-A	1.0000
YEL020W-A	YKL195W	0.9997
YEL022W	YJR077C	0.7380
YEL024W	YML129C	0.9215
YEL026W	YER172C	0.9974
YEL026W	YLR197W	0.9952
YEL026W	YOR310C	0.9876
YEL026W	YPL193W	0.9138
YEL027W	YGR105W	0.9609
YEL027W	YHR026W	0.7891
YEL027W	YOR270C	0.9773
YEL030W	YOR232W	0.9707
YEL032W	YFR028C	0.8655
YEL032W	YGL201C	0.9037
YEL032W	YIL150C	0.9099
YEL032W	YLR103C	0.8603
YEL032W	YLR274W	0.9999
YEL032W	YPR019W	0.9994
YEL034W	YER025W	0.8655
YEL034W	YFR004W	0.7380
YEL034W	YHR068W	0.9836
YEL034W	YJR007W	0.8655
YEL034W	YJR070C	0.9099
YEL034W	YLR340W	0.8087
YEL034W	YNR051C	0.7380
YEL034W	YPL237W	0.9623
YEL036C	YGR132C	0.7380
YEL036C	YGR231C	0.7380
YEL036C	YJL183W	0.9970
YEL036C	YJR075W	0.9794
YEL036C	YLR026C	0.9871
YEL036C	YLR342W	0.7380
YEL036C	YPL050C	1.0000
YEL036C	YPL094C	0.7380
YEL037C	YER012W	0.9215
YEL037C	YER143W	0.9978
YEL037C	YER148W	0.9215
YEL037C	YER162C	1.0000
YEL037C	YFR004W	0.9895
YEL037C	YFR052W	0.8566
YEL037C	YGL048C	0.9999
YEL037C	YHL025W	0.9215
YEL037C	YHR027C	0.9998
YEL037C	YHR200W	0.9899
YEL037C	YKL145W	1.0000
YEL037C	YLL039C	0.9999

YEL037C	YMR201C	0.9249
YEL037C	YOR117W	0.9967
YEL037C	YOR259C	0.9772
YEL037C	YPL096W	1.0000
YEL043W	YGR089W	0.5774
YEL044W	YGL150C	0.8087
YEL046C	YFR004W	0.7380
YEL046C	YLL039C	0.8566
YEL047C	YFR004W	0.7380
YEL048C	YKR068C	0.8953
YEL048C	YMR218C	0.9319
YEL048C	YOR115C	0.8953
YEL051W	YGR020C	0.9924
YEL051W	YHR039C-A	0.9894
YEL051W	YJR033C	0.7891
YEL051W	YKL080W	0.8659
YEL051W	YOR270C	0.9829
YEL051W	YOR332W	0.9891
YEL051W	YPR036W	0.9568
YEL054C	YGL120C	0.7380
YEL054C	YHR170W	0.9215
YEL055C	YGR128C	0.8659
YEL055C	YHR196W	0.8659
YEL055C	YJL109C	0.7380
YEL055C	YLR196W	0.7380
YEL055C	YMR093W	0.7380
YEL055C	YPL126W	0.7380
YEL056W	YHR118C	0.9285
YEL056W	YLL004W	0.8566
YEL056W	YLL022C	0.9990
YEL056W	YML065W	0.9021
YEL056W	YNL030W	0.9624
YEL056W	YNL031C	0.9266
YEL056W	YNL261W	0.8087
YEL056W	YPL001W	0.9999
YEL056W	YPR162C	0.8087
YEL058W	YFR004W	0.7380
YEL058W	YNL218W	0.7380
YEL062W	YJR090C	0.8953
YEL065W	YGL071W	0.9758
YEL071W	YFR004W	0.7380
YEL071W	YLL039C	0.8566
YER002W	YPR016C	0.8087
YER005W	YPR036W	0.9099
YER006W	YER126C	0.9313
YER006W	YFL002C	0.7380
YER006W	YFR001W	0.7380

YER006W	YFR031C-A	0.7380
YER006W	YGL030W	0.7380
YER006W	YGL111W	0.8087
YER006W	YGR103W	0.9993
YER006W	YGR245C	0.9933
YER006W	YHR010W	0.7380
YER006W	YHR052W	0.9869
YER006W	YHR088W	0.7380
YER006W	YHR197W	0.9992
YER006W	YKL009W	0.9958
YER006W	YKR081C	0.8659
YER006W	YLL045C	0.7380
YER006W	YLR002C	0.9985
YER006W	YLR074C	0.8659
YER006W	YLR106C	0.9860
YER006W	YLR325C	0.7380
YER006W	YLR449W	0.7380
YER006W	YMR049C	0.9904
YER006W	YMR290C	0.9634
YER006W	YNL002C	0.8087
YER006W	YNL061W	0.9951
YER006W	YNL110C	0.9648
YER006W	YNL182C	0.7380
YER006W	YNR053C	0.9313
YER006W	YOL077C	0.7380
YER006W	YOL127W	0.7380
YER006W	YOR063W	0.7380
YER006W	YOR206W	0.9860
YER006W	YOR272W	0.8659
YER006W	YPL043W	0.8659
YER006W	YPL093W	0.9964
YER006W	YPL131W	0.9266
YER006W	YPL146C	0.8087
YER006W	YPL198W	0.7380
YER006W	YPL211W	0.9499
YER006W	YPR016C	0.9860
YER007C-A	YJR014W	0.9975
YER007C-A	YKL028W	0.7380
YER007W	YHR027C	0.8566
YER007W	YML085C	0.9099
YER007W	YOR349W	0.8566
YER008C	YGL233W	0.7380
YER008C	YIL068C	0.9624
YER008C	YJL085W	0.9266
YER008C	YLR166C	0.9813
YER008C	YLR229C	0.9799
YER008C	YPR055W	0.9994

YER008C	YPR165W	0.9999
YER012W	YER021W	0.9696
YER012W	YER094C	0.9989
YER012W	YFR004W	0.8953
YER012W	YFR050C	0.9993
YER012W	YFR052W	1.0000
YER012W	YGL011C	0.9961
YER012W	YGL048C	0.9834
YER012W	YGR135W	0.9989
YER012W	YGR253C	0.9997
YER012W	YHR027C	0.9707
YER012W	YHR134W	0.9215
YER012W	YHR200W	1.0000
YER012W	YJL001W	0.9994
YER012W	YKL145W	1.0000
YER012W	YKL206C	0.7380
YER012W	YML092C	0.9985
YER012W	YMR314W	0.9989
YER012W	YOL038W	0.9999
YER012W	YOR117W	0.9953
YER012W	YOR157C	0.9942
YER012W	YOR259C	0.8953
YER012W	YOR261C	0.9918
YER012W	YOR362C	1.0000
YER012W	YPR103W	1.0000
YER012W	YPR108W	0.8087
YER013W	YLR117C	0.9613
YER013W	YMR213W	0.8659
YER016W	YGL061C	0.7253
YER016W	YLR045C	0.9939
YER016W	YPL209C	0.9456
YER016W	YPL269W	1.0000
YER017C	YGR132C	0.9598
YER017C	YGR231C	0.8566
YER017C	YMR089C	0.9998
YER018C	YFR046C	0.5774
YER018C	YIL144W	1.0000
YER018C	YIR010W	0.8297
YER018C	YMR117C	1.0000
YER018C	YOL069W	0.9994
YER019C-A	YML019W	0.9151
YER020W	YJL106W	0.9975
YER020W	YOR371C	0.9215
YER021W	YFR004W	0.9994
YER021W	YFR010W	0.9313
YER021W	YFR052W	0.9929
YER021W	YGL004C	0.8566

YER021W	YGL048C	0.7380
YER021W	YGR232W	0.9860
YER021W	YHL030W	0.8659
YER021W	YHR027C	0.9969
YER021W	YHR200W	0.9869
YER021W	YIL075C	0.7380
YER021W	YKL145W	0.9313
YER021W	YLR421C	0.7380
YER021W	YOL038W	0.7380
YER021W	YOR259C	0.7380
YER021W	YOR261C	0.8659
YER021W	YPL106C	0.7380
YER021W	YPR108W	0.9949
YER022W	YER148W	0.9574
YER022W	YGL025C	0.9987
YER022W	YGL127C	0.9794
YER022W	YGL151W	0.9928
YER022W	YGR005C	0.9747
YER022W	YGR104C	1.0000
YER022W	YGR186W	0.9929
YER022W	YHR041C	1.0000
YER022W	YHR058C	1.0000
YER022W	YJL140W	0.9574
YER022W	YLR071C	1.0000
YER022W	YMR112C	0.9999
YER022W	YNL236W	0.9959
YER022W	YNR010W	0.9997
YER022W	YOL005C	0.9555
YER022W	YOL051W	0.9988
YER022W	YOL135C	0.9998
YER022W	YOR174W	0.9989
YER022W	YPL042C	0.9021
YER022W	YPL129W	0.9871
YER022W	YPL248C	0.9933
YER022W	YPR070W	0.9928
YER022W	YPR168W	0.7380
YER023W	YFR004W	0.7380
YER023W	YGL137W	0.6147
YER023W	YLR291C	0.6672
YER025W	YFR004W	0.7380
YER025W	YGR083C	0.9902
YER025W	YJR007W	0.9962
YER025W	YKR026C	0.9648
YER025W	YLR215C	0.9875
YER025W	YLR291C	0.9981
YER025W	YMR146C	0.8566
YER025W	YMR309C	0.9313

YER025W	YOR260W	0.9987
YER025W	YOR361C	0.8566
YER025W	YPL237W	0.9999
YER025W	YPR041W	0.9999
YER027C	YER129W	0.7380
YER027C	YGL115W	0.9907
YER027C	YJL089W	0.9138
YER027C	YJL217W	0.9138
YER029C	YER112W	0.9729
YER029C	YER172C	0.9996
YER029C	YFL017W-A	0.7380
YER029C	YGL120C	0.7380
YER029C	YGL128C	0.7380
YER029C	YGR013W	0.8659
YER029C	YGR074W	0.9648
YER029C	YGR091W	0.9313
YER029C	YHR086W	0.9313
YER029C	YHR165C	0.9648
YER029C	YIL061C	0.7380
YER029C	YIR009W	0.7380
YER029C	YJL203W	0.7380
YER029C	YJR022W	0.7380
YER029C	YKL012W	0.9313
YER029C	YKL173W	0.9313
YER029C	YLL036C	0.9186
YER029C	YLR117C	0.9021
YER029C	YLR147C	0.9452
YER029C	YLR275W	0.8659
YER029C	YLR298C	0.9648
YER029C	YLR424W	0.9313
YER029C	YLR438C-A	0.8953
YER029C	YML046W	0.7380
YER029C	YML049C	0.7380
YER029C	YMR125W	0.8659
YER029C	YMR240C	0.9648
YER029C	YMR288W	0.7380
YER029C	YOR159C	0.7380
YER029C	YOR308C	0.7380
YER029C	YPL151C	0.7380
YER029C	YPL213W	0.9743
YER029C	YPR082C	0.7380
YER029C	YPR101W	0.8659
YER029C	YPR178W	0.8659
YER029C	YPR182W	0.8659
YER030W	YOL012C	0.9998
YER031C	YER136W	0.9999
YER031C	YGR172C	0.9710

YER031C	YJL204C	0.9099
YER031C	YNL044W	0.9099
YER031C	YNL263C	0.9723
YER031C	YOR326W	0.9138
YER032W	YGR140W	0.7909
YER032W	YMR117C	0.7909
YER036C	YFR004W	0.7380
YER036C	YGL099W	0.8953
YER036C	YGR285C	0.8953
YER036C	YHL033C	0.8087
YER036C	YJL080C	0.8953
YER036C	YML073C	0.8087
YER036C	YOR204W	0.8953
YER036C	YPR016C	0.8953
YER036C	YPR036W	0.7380
YER038C	YML023C	0.9999
YER038C	YOL034W	0.9835
YER040W	YJR066W	0.9667
YER040W	YNL189W	0.9138
YER040W	YNL229C	0.9997
YER042W	YIR037W	0.9609
YER043C	YFL028C	0.6672
YER043C	YFR004W	0.7380
YER044C	YGL001C	0.9923
YER044C	YGL012W	0.9151
YER044C	YGR060W	0.9998
YER044C	YGR175C	0.9099
YER044C	YHR007C	0.9993
YER044C	YHR072W	0.9933
YER044C	YHR190W	0.9151
YER044C	YLR056W	0.9762
YER044C	YLR100W	0.9993
YER044C	YML008C	1.0000
YER044C	YMR202W	0.9762
YER044C	YNL280C	0.9151
YER047C	YER161C	0.9138
YER047C	YGR140W	0.7909
YER047C	YMR032W	0.5774
YER047C	YOL034W	0.5774
YER048C	YFR004W	0.7380
YER049W	YLR249W	0.7380
YER050C	YGL129C	0.7380
YER050C	YKL155C	0.8566
YER050C	YOR158W	0.7380
YER050C	YPL118W	0.7380
YER051W	YNL031C	0.9947
YER052C	YFR004W	0.7380

YER052C	YNL135C	0.9813
YER054C	YER133W	0.9913
YER054C	YML088W	0.8998
YER059W	YPL031C	0.9968
YER059W	YPL046C	0.9099
YER062C	YER171W	0.7380
YER062C	YPL201C	0.5774
YER067W	YLL039C	0.8566
YER068W	YIL038C	0.9947
YER068W	YNL288W	0.9989
YER068W	YNR052C	0.9834
YER068W	YPL037C	0.9099
YER068W	YPR072W	0.9834
YER069W	YFR004W	0.7380
YER070W	YFR004W	0.7380
YER070W	YHR169W	0.7380
YER070W	YIL066C	0.7380
YER070W	YJL026W	0.9963
YER070W	YML058W	0.9975
YER071C	YIR003W	0.9128
YER071C	YKL007W	0.8659
YER073W	YFR004W	0.7380
YER075C	YHR030C	0.9382
YER075C	YLR113W	0.9215
YER081W	YFR004W	0.7380
YER081W	YIL074C	0.9667
YER082C	YER118C	0.5774
YER082C	YGR090W	0.9908
YER082C	YGR128C	0.9743
YER082C	YHR196W	0.8659
YER082C	YJL109C	0.9820
YER082C	YJR002W	0.9980
YER082C	YLL011W	0.6147
YER082C	YLR129W	0.9313
YER082C	YLR197W	0.9648
YER082C	YLR222C	0.9313
YER082C	YLR409C	0.9313
YER082C	YMR093W	0.9313
YER082C	YMR229C	0.7380
YER082C	YNL132W	0.9648
YER082C	YOR078W	0.7380
YER082C	YPL126W	0.9648
YER082C	YPL217C	0.9313
YER082C	YPR112C	0.7380
YER086W	YER095W	0.7380
YER086W	YKL029C	0.7380
YER086W	YKL081W	0.7380

YER086W	YLR259C	0.8659
YER087C-B	YGL022W	0.9081
YER087C-B	YLR378C	0.9990
YER087C-B	YOR085W	0.9742
YER087C-B	YOR103C	0.9081
YER087C-B	YPL094C	0.8953
YER088C	YJL164C	0.8456
YER089C	YHR079C	0.9586
YER089C	YPL153C	0.9979
YER090W	YFR004W	0.7380
YER090W	YHR027C	0.7380
YER090W	YKL211C	0.9771
YER090W	YLR180W	0.7380
YER091C	YFR004W	0.7380
YER092W	YFL013C	0.8659
YER092W	YGL150C	0.9499
YER092W	YJL081C	0.7380
YER092W	YLR052W	0.9554
YER092W	YLR423C	0.5774
YER092W	YOR141C	0.8659
YER092W	YOR189W	0.8087
YER092W	YPL129W	0.8659
YER092W	YPL235W	0.8659
YER093C	YGR040W	0.7380
YER093C	YKL203C	0.9999
YER093C	YNL006W	0.9707
YER093C	YOL078W	0.9215
YER094C	YFR050C	0.9770
YER094C	YGL011C	0.9313
YER094C	YGR135W	0.7380
YER094C	YHR200W	0.7380
YER094C	YJL001W	0.9770
YER094C	YML092C	0.9313
YER094C	YOL038W	0.8659
YER094C	YOR157C	0.9976
YER094C	YOR362C	0.7380
YER094C	YPR103W	0.9994
YER095W	YGL163C	1.0000
YER095W	YGL245W	0.7380
YER095W	YGR234W	0.7380
YER095W	YJL092W	1.0000
YER095W	YJR109C	0.7380
YER095W	YKL104C	0.7380
YER095W	YML032C	1.0000
YER095W	YNL085W	0.7380
YER095W	YOR151C	0.7380
YER095W	YPR010C	0.7380

YER099C	YKL181W	0.8977
YER099C	YMR139W	0.8353
YER099C	YOL061W	0.9425
YER100W	YLL039C	0.7380
YER102W	YHL034C	0.8087
YER103W	YGL130W	0.7380
YER103W	YGR128C	0.7380
YER103W	YGR192C	0.7380
YER103W	YLL024C	0.7380
YER103W	YLL039C	0.7380
YER103W	YOR039W	0.7380
YER103W	YOR304W	0.7380
YER103W	YPL106C	0.7380
YER105C	YGL092W	0.9127
YER105C	YKL057C	0.9127
YER105C	YMR153W	0.8258
YER106W	YPL204W	0.9746
YER107C	YGL122C	0.5164
YER107C	YIL115C	0.7380
YER107C	YJL041W	0.7380
YER107C	YJL061W	0.8659
YER107C	YMR047C	1.0000
YER110C	YFR004W	0.7380
YER110C	YGL195W	0.8659
YER110C	YGR119C	0.8353
YER110C	YIL094C	0.7380
YER110C	YJR123W	0.7380
YER110C	YKL068W	0.8087
YER110C	YLL039C	0.7380
YER110C	YLR293C	0.9997
YER110C	YLR335W	0.9743
YER110C	YLR347C	0.8659
YER110C	YLR441C	0.7380
YER110C	YML007W	0.9099
YER110C	YMR047C	0.9186
YER110C	YNL030W	0.9249
YER110C	YNL031C	0.9944
YER110C	YNL189W	0.8659
YER110C	YOL120C	0.7380
YER110C	YOR098C	0.9099
YER110C	YPL235W	0.7380
YER110C	YPR093C	0.9107
YER110C	YPR174C	0.8659
YER111C	YHR030C	0.9640
YER111C	YLR182W	1.0000
YER111C	YOR066W	0.9215
YER111C	YOR083W	0.9960

YER111C	YPL153C	0.8087
YER111C	YPR119W	0.8953
YER112W	YER146W	0.9951
YER112W	YER165W	0.8566
YER112W	YER172C	0.9993
YER112W	YGL173C	0.9128
YER112W	YGR091W	0.8659
YER112W	YGR162W	0.8566
YER112W	YHR165C	0.7380
YER112W	YJL124C	1.0000
YER112W	YJR022W	0.9996
YER112W	YKL173W	0.7380
YER112W	YLR147C	0.7380
YER112W	YLR275W	0.8297
YER112W	YLR438C-A	0.9999
YER112W	YMR268C	0.9282
YER112W	YNL118C	0.5107
YER112W	YNL147W	0.9986
YER112W	YOL149W	0.8816
YER112W	YOR204W	0.7891
YER112W	YOR308C	0.7380
YER112W	YPR082C	0.7380
YER112W	YPR178W	0.7380
YER114C	YER124C	0.7253
YER114C	YER177W	0.7380
YER114C	YHL007C	0.5774
YER114C	YML109W	0.5774
YER118C	YGR014W	0.9938
YER118C	YJL128C	0.9971
YER118C	YLR096W	0.5774
YER118C	YLR353W	0.9215
YER118C	YLR362W	0.9984
YER118C	YOR181W	0.9530
YER120W	YFR004W	0.7380
YER120W	YGR086C	0.7380
YER120W	YHL020C	0.9891
YER120W	YHR200W	0.7380
YER122C	YFR004W	0.7380
YER122C	YGL137W	0.7380
YER122C	YLR078C	0.9215
YER122C	YNL287W	0.9266
YER124C	YLR362W	0.8965
YER125W	YFR022W	0.9899
YER125W	YFR028C	0.8566
YER125W	YGL144C	0.9790
YER125W	YGR068C	0.9948
YER125W	YGR136W	0.7543

YER125W	YGR268C	0.8816
YER125W	YHL002W	0.9758
YER125W	YHL024W	0.8655
YER125W	YHR131C	0.9182
YER125W	YIL021W	0.7380
YER125W	YIR033W	0.9995
YER125W	YJL084C	0.9986
YER125W	YJL151C	1.0000
YER125W	YJL172W	0.8655
YER125W	YKL020C	0.9987
YER125W	YKR021W	0.9906
YER125W	YLL039C	0.9598
YER125W	YLR392C	0.9939
YER125W	YMR171C	0.7543
YER125W	YMR275C	1.0000
YER125W	YMR316W	0.9848
YER125W	YOR018W	0.9951
YER125W	YOR124C	0.9961
YER125W	YOR138C	0.9945
YER125W	YOR322C	0.9068
YER125W	YOR385W	0.5774
YER125W	YPL176C	0.7696
YER125W	YPR030W	0.9312
YER125W	YPR187W	0.7380
YER126C	YGL111W	0.8953
YER126C	YGR103W	0.8953
YER126C	YGR245C	0.8659
YER126C	YHR197W	0.9887
YER126C	YKL009W	0.9895
YER126C	YKR081C	0.8659
YER126C	YLR009W	0.8953
YER126C	YLR074C	0.9313
YER126C	YMR049C	0.8659
YER126C	YMR290C	0.8659
YER126C	YNL002C	0.8659
YER126C	YNL110C	0.9313
YER126C	YNR053C	0.9624
YER126C	YOR063W	0.7380
YER126C	YOR206W	0.7380
YER126C	YPL093W	0.9955
YER126C	YPR016C	0.9963
YER128W	YPR173C	0.5774
YER129W	YFR028C	0.8566
YER129W	YGL115W	0.9313
YER129W	YIL035C	0.8566
YER131W	YFR004W	0.7380
YER131W	YLR435W	0.8297

YER132C	YMR139W	0.7380
YER133W	YER177W	0.9313
YER133W	YFL023W	0.8965
YER133W	YFR003C	0.9999
YER133W	YFR004W	0.7380
YER133W	YFR015C	0.7380
YER133W	YGL111W	0.7380
YER133W	YGR103W	0.7380
YER133W	YGR156W	0.9931
YER133W	YHR052W	0.7380
YER133W	YHR158C	0.7380
YER133W	YIL070C	0.7380
YER133W	YIR006C	0.6880
YER133W	YJL033W	0.7380
YER133W	YJL042W	0.9674
YER133W	YJR007W	0.9266
YER133W	YJR093C	0.9759
YER133W	YKL018W	0.9977
YER133W	YKL059C	0.9499
YER133W	YKL193C	1.0000
YER133W	YKR002W	0.9743
YER133W	YLR028C	0.9648
YER133W	YLR115W	0.9860
YER133W	YLR258W	0.8659
YER133W	YLR263W	0.9963
YER133W	YLR277C	0.9965
YER133W	YLR430W	0.9995
YER133W	YLR449W	0.7380
YER133W	YML074C	0.9266
YER133W	YMR049C	0.7380
YER133W	YMR140W	0.9099
YER133W	YMR311C	0.9976
YER133W	YNL222W	0.8087
YER133W	YNL233W	0.9999
YER133W	YNL317W	0.9813
YER133W	YOR178C	1.0000
YER133W	YOR179C	0.9021
YER133W	YOR227W	0.9743
YER133W	YOR329C	0.9998
YER133W	YPL074W	0.8965
YER133W	YPL137C	0.9743
YER133W	YPL237W	0.7380
YER133W	YPR107C	0.8659
YER136W	YFL005W	0.9990
YER136W	YFL038C	1.0000
YER136W	YGL210W	0.8760
YER136W	YKR014C	0.9568

YER136W	YLR262C	0.9834
YER136W	YML001W	0.9903
YER136W	YOR089C	0.9975
YER139C	YIL021W	0.9886
YER139C	YKR081C	0.7380
YER139C	YOL005C	0.8603
YER139C	YOR151C	0.8087
YER139C	YOR224C	0.9285
YER143W	YFR004W	0.7380
YER143W	YJR141W	0.5774
YER143W	YLL039C	0.9852
YER143W	YML088W	0.9758
YER143W	YOR327C	0.9747
YER143W	YPL232W	0.9790
YER144C	YMR032W	0.7253
YER146W	YER172C	0.9986
YER146W	YGL173C	0.9313
YER146W	YHR165C	0.9743
YER146W	YJL124C	1.0000
YER146W	YJR022W	0.9885
YER146W	YKL173W	0.8659
YER146W	YLR275W	0.6672
YER146W	YLR438C-A	0.9993
YER146W	YMR268C	0.9533
YER146W	YNL145W	0.3670
YER146W	YNL147W	0.9923
YER146W	YPR178W	0.8659
YER148W	YER159C	0.9980
YER148W	YFR034C	0.9249
YER148W	YGL019W	0.9249
YER148W	YGL048C	0.9790
YER148W	YGL112C	1.0000
YER148W	YGL241W	0.9985
YER148W	YGR056W	0.8566
YER148W	YGR104C	0.9215
YER148W	YGR246C	1.0000
YER148W	YGR274C	1.0000
YER148W	YHR041C	0.9555
YER148W	YKL058W	1.0000
YER148W	YKR001C	0.7380
YER148W	YLR055C	0.9974
YER148W	YLR357W	0.8566
YER148W	YLR399C	0.9598
YER148W	YLR418C	0.9124
YER148W	YML015C	1.0000
YER148W	YML098W	0.9995
YER148W	YML114C	0.9990

YER148W	YMR005W	1.0000
YER148W	YMR125W	0.8818
YER148W	YMR227C	0.9995
YER148W	YMR236W	0.9990
YER148W	YMR270C	0.9992
YER148W	YNL039W	0.9282
YER148W	YNL216W	0.9790
YER148W	YOL148C	0.9507
YER148W	YOR039W	0.8566
YER148W	YOR047C	0.9138
YER148W	YOR061W	0.8655
YER148W	YOR194C	1.0000
YER148W	YOR298C-A	0.9799
YER148W	YPL011C	0.9948
YER148W	YPL082C	1.0000
YER148W	YPL129W	1.0000
YER148W	YPL235W	0.9249
YER148W	YPL248C	1.0000
YER148W	YPL254W	0.8628
YER148W	YPR072W	0.9215
YER148W	YPR086W	1.0000
YER149C	YLL021W	0.9934
YER149C	YLR319C	0.7909
YER149C	YMR124W	0.7253
YER151C	YER162C	0.9215
YER151C	YFR004W	0.9773
YER151C	YGL106W	0.7380
YER151C	YKL054C	0.9186
YER151C	YLL024C	0.9186
YER151C	YLR150W	0.7380
YER151C	YLR441C	0.7380
YER151C	YNR051C	1.0000
YER151C	YOL082W	0.9138
YER151C	YOR151C	0.9186
YER151C	YPR181C	0.9640
YER154W	YPL173W	0.9215
YER155C	YJL098W	0.7380
YER155C	YNL201C	0.7380
YER155C	YNL207W	0.8659
YER155C	YPR088C	0.7380
YER157W	YGL005C	0.9266
YER157W	YGL223C	0.9934
YER157W	YGR120C	1.0000
YER157W	YLR026C	0.9249
YER157W	YML071C	0.9904
YER157W	YNL041C	0.9717
YER157W	YNL051W	0.9624

YER157W	YPR105C	0.9994
YER159C	YPL082C	0.9972
YER161C	YGL201C	0.9327
YER161C	YHR166C	0.9723
YER161C	YNL189W	0.7380
YER162C	YHL025W	0.9215
YER162C	YJR052W	0.9861
YER164W	YGL019W	0.9869
YER164W	YGL207W	0.9813
YER164W	YGL244W	0.9919
YER164W	YIL035C	0.9987
YER164W	YKR001C	0.7380
YER164W	YML010W	0.9215
YER164W	YML069W	0.9948
YER164W	YNL007C	0.7380
YER164W	YOR039W	0.9966
YER164W	YOR061W	0.9987
YER164W	YOR304W	0.7380
YER165W	YFR004W	0.7380
YER165W	YGL044C	0.9927
YER165W	YGL049C	1.0000
YER165W	YGL120C	0.7380
YER165W	YGL122C	0.8645
YER165W	YGR162W	0.9999
YER165W	YGR178C	0.8965
YER165W	YGR218W	0.9985
YER165W	YHL034C	0.7380
YER165W	YIR001C	0.9539
YER165W	YJL033W	0.7380
YER165W	YJL080C	0.9215
YER165W	YKL068W	0.8087
YER165W	YML117W	0.7380
YER165W	YMR125W	0.8659
YER165W	YNL251C	0.8087
YER165W	YOL139C	0.7380
YER165W	YOL149W	0.9249
YER165W	YPL169C	0.9782
YER165W	YPL190C	0.7380
YER167W	YGR040W	0.9266
YER167W	YGR229C	0.8965
YER167W	YHR158C	0.8566
YER167W	YLR096W	0.8566
YER168C	YGR128C	0.9249
YER171W	YGL048C	0.7380
YER171W	YIL128W	0.9820
YER171W	YIL143C	1.0000
YER171W	YLR005W	1.0000

YER171W	YMR112C	0.9099
YER171W	YNL064C	0.7380
YER171W	YPL122C	0.9747
YER171W	YPR025C	0.9186
YER171W	YPR056W	0.9598
YER172C	YFL017W-A	0.9871
YER172C	YGL120C	0.9499
YER172C	YGL128C	0.8659
YER172C	YGR013W	0.7380
YER172C	YGR074W	0.9996
YER172C	YGR075C	0.9986
YER172C	YGR091W	0.9998
YER172C	YGR278W	0.7380
YER172C	YHR156C	0.8566
YER172C	YHR165C	1.0000
YER172C	YIL061C	0.9414
YER172C	YJL203W	0.8659
YER172C	YJR022W	0.9995
YER172C	YKL173W	1.0000
YER172C	YKR086W	0.9710
YER172C	YLL036C	0.9891
YER172C	YLR117C	0.9499
YER172C	YLR147C	0.9987
YER172C	YLR275W	0.9975
YER172C	YLR310C	0.7909
YER172C	YLR424W	0.9313
YER172C	YLR438C-A	0.9977
YER172C	YML046W	0.7380
YER172C	YML049C	0.7380
YER172C	YMR125W	0.8659
YER172C	YMR213W	0.9313
YER172C	YMR240C	0.9128
YER172C	YMR288W	0.7380
YER172C	YNL147W	0.9956
YER172C	YNR011C	0.8965
YER172C	YOR159C	0.9871
YER172C	YOR308C	1.0000
YER172C	YPL151C	0.9313
YER172C	YPL213W	0.9499
YER172C	YPR082C	0.9993
YER172C	YPR101W	0.8659
YER172C	YPR178W	0.9999
YER172C	YPR182W	0.9934
YER173W	YJR068W	0.9999
YER173W	YNL290W	0.9999
YER173W	YOL094C	0.9967
YER173W	YPL194W	0.8894

YER174C	YGL071W	0.9922
YER174C	YGL220W	0.9616
YER174C	YGR262C	0.9990
YER177W	YFR004W	0.7380
YER177W	YFR017C	0.7380
YER177W	YGL003C	0.9609
YER177W	YGL115W	0.7380
YER177W	YGL252C	0.9624
YER177W	YGR097W	0.7380
YER177W	YIR003W	0.7380
YER177W	YKL168C	0.6672
YER177W	YKL182W	0.8566
YER177W	YLR028C	0.7380
YER177W	YNL031C	0.8889
YER177W	YNL076W	0.9794
YER177W	YNL267W	0.9993
YER177W	YNL330C	0.9215
YER177W	YOR244W	0.9215
YER177W	YPL231W	0.8566
YER177W	YPL267W	0.9215
YER177W	YPR030W	0.7380
YER178W	YFR004W	0.7380
YER178W	YIL042C	0.9191
YER178W	YMR308C	0.7380
YER178W	YNL071W	0.9313
YER179W	YNR016C	0.8353
YER179W	YPL121C	0.9099
YFL002C	YFR001W	0.7380
YFL002C	YGL111W	0.8087
YFL002C	YHR052W	0.9313
YFL002C	YHR088W	0.7380
YFL002C	YHR197W	0.9313
YFL002C	YKR081C	0.7380
YFL002C	YLR002C	0.7380
YFL002C	YLR449W	0.7380
YFL002C	YMR049C	0.8659
YFL002C	YMR290C	0.7380
YFL002C	YNL002C	0.7380
YFL002C	YNL061W	0.9313
YFL002C	YNL110C	0.8659
YFL002C	YOL077C	0.8659
YFL002C	YOL127W	0.7380
YFL002C	YOR063W	0.7380
YFL002C	YOR206W	0.8659
YFL002C	YOR272W	0.8659
YFL002C	YPL093W	0.8087
YFL002C	YPL131W	0.7380

YFL002C	YPL211W	0.7380
YFL005W	YFR004W	0.7380
YFL005W	YGL233W	0.9927
YFL005W	YGR009C	0.9151
YFL005W	YNL044W	0.9099
YFL005W	YNL263C	0.9723
YFL005W	YNL272C	1.0000
YFL005W	YNL293W	0.7696
YFL005W	YNR049C	0.9772
YFL005W	YOR326W	0.9780
YFL005W	YOR370C	0.7891
YFL005W	YPR017C	0.9539
YFL005W	YPR032W	0.9979
YFL007W	YGL011C	0.8659
YFL007W	YJL001W	0.7380
YFL007W	YML092C	0.8659
YFL007W	YOL038W	0.9021
YFL007W	YOR362C	0.7380
YFL007W	YPR103W	0.7380
YFL008W	YGR089W	0.5774
YFL008W	YIL026C	0.9768
YFL008W	YJL074C	1.0000
YFL008W	YKR054C	0.5774
YFL008W	YLR196W	0.7380
YFL008W	YMR001C	0.9021
YFL008W	YMR076C	0.9768
YFL008W	YMR117C	0.5774
YFL008W	YOL069W	0.5774
YFL008W	YPR007C	0.8566
YFL008W	YPR141C	0.5774
YFL009W	YJL194W	0.9989
YFL009W	YKL159C	0.8965
YFL009W	YKL185W	0.8714
YFL009W	YLR079W	0.9974
YFL009W	YOL025W	0.9249
YFL009W	YOL133W	0.9995
YFL011W	YPL240C	0.6672
YFL013C	YFL039C	0.7380
YFL013C	YGL150C	0.9904
YFL013C	YJL081C	0.9021
YFL013C	YLR052W	0.7380
YFL013C	YNL059C	0.8659
YFL013C	YNL215W	0.8659
YFL013C	YOR141C	0.8659
YFL013C	YOR189W	0.9285
YFL013C	YPL129W	0.7380
YFL013C	YPL235W	0.8659

YFL016C	YJR045C	0.9995
YFL017W-A	YGR013W	0.7380
YFL017W-A	YGR074W	0.8659
YFL017W-A	YGR091W	0.7380
YFL017W-A	YIL061C	0.7380
YFL017W-A	YKL012W	0.7380
YFL017W-A	YKL173W	0.7380
YFL017W-A	YLL036C	0.9186
YFL017W-A	YLR147C	0.8659
YFL017W-A	YLR275W	0.7380
YFL017W-A	YMR125W	0.7380
YFL017W-A	YOR159C	0.8929
YFL017W-A	YPL213W	0.8087
YFL018C	YFR049W	0.8659
YFL018C	YGR193C	0.7380
YFL018C	YIL125W	0.9313
YFL022C	YFR004W	0.7380
YFL022C	YLR060W	0.9648
YFL023W	YKL081W	0.9151
YFL023W	YLR200W	0.9664
YFL023W	YNL135C	0.9151
YFL023W	YPR080W	0.9151
YFL024C	YFL039C	0.9895
YFL024C	YGR002C	0.9961
YFL024C	YHR090C	0.9990
YFL024C	YHR099W	0.9946
YFL024C	YJL081C	0.9999
YFL024C	YJL098W	0.7380
YFL024C	YJR082C	0.7380
YFL024C	YML007W	0.7380
YFL024C	YNL030W	0.7543
YFL024C	YNL107W	0.9998
YFL024C	YNL136W	0.9808
YFL024C	YOR244W	1.0000
YFL024C	YPR023C	0.9985
YFL025C	YMR307W	0.9215
YFL026W	YHR005C	0.9710
YFL026W	YOR212W	0.8965
YFL026W	YPL187W	0.9719
YFL030W	YPL031C	0.5779
YFL031W	YHR079C	0.1485
YFL033C	YHR205W	0.8655
YFL033C	YJL164C	0.8353
YFL033C	YLR096W	0.8566
YFL033C	YNL157W	0.8891
YFL033C	YPL031C	0.7543
YFL034C-B	YIL106W	0.9249

YFL034C-B	YNL161W	0.9996
YFL034W	YHL019C	0.7380
YFL036W	YMR228W	0.9997
YFL037W	YFR004W	0.7380
YFL037W	YGR218W	0.7380
YFL037W	YJL014W	0.8566
YFL037W	YLR045C	0.9138
YFL037W	YML085C	0.9327
YFL037W	YOR265W	1.0000
YFL038C	YGR172C	0.9978
YFL038C	YLR026C	0.9981
YFL038C	YNL044W	0.9099
YFL038C	YNL263C	0.9723
YFL038C	YOR370C	0.9960
YFL038C	YPR017C	0.9803
YFL038C	YPR176C	0.9603
YFL039C	YFR004W	0.7380
YFL039C	YGL150C	0.9999
YFL039C	YGR002C	0.8087
YFL039C	YGR080W	0.9981
YFL039C	YHR016C	0.9138
YFL039C	YHR023W	0.9298
YFL039C	YHR090C	0.8566
YFL039C	YIL138C	0.9282
YFL039C	YJL005W	0.7380
YFL039C	YJL014W	0.8566
YFL039C	YJL081C	0.9464
YFL039C	YKL196C	0.9215
YFL039C	YLL050C	1.0000
YFL039C	YLR093C	0.9215
YFL039C	YLR196W	0.7380
YFL039C	YLR337C	0.9966
YFL039C	YLR429W	0.9447
YFL039C	YML041C	0.7380
YFL039C	YMR072W	0.7380
YFL039C	YMR092C	0.9981
YFL039C	YMR109W	0.9539
YFL039C	YNL059C	0.7380
YFL039C	YNL079C	0.9574
YFL039C	YNL107W	0.9285
YFL039C	YNL138W	0.9999
YFL039C	YNL243W	0.9037
YFL039C	YNR031C	0.9099
YFL039C	YOL012C	0.9805
YFL039C	YOR122C	0.9992
YFL039C	YOR141C	0.8087
YFL039C	YOR181W	0.9916

YFL039C	YOR244W	0.9989
YFL039C	YOR326W	0.9946
YFL039C	YOR367W	0.9993
YFL039C	YPL129W	0.7380
YFL039C	YPL235W	0.8087
YFL039C	YPL242C	0.9249
YFL044C	YGR048W	0.9249
YFL044C	YKL213C	0.9249
YFL045C	YFR004W	0.7380
YFL045C	YGL195W	0.7380
YFL045C	YHR200W	0.8087
YFL045C	YPL235W	0.7380
YFL048C	YLR080W	0.9553
YFL048C	YNL287W	0.9191
YFL049W	YHL025W	0.9874
YFL049W	YJL176C	0.9895
YFL049W	YMR033W	0.7380
YFL049W	YNR023W	0.8566
YFL049W	YOR290C	0.9956
YFL049W	YPL016W	0.7380
YFL049W	YPR034W	0.7380
YFL050C	YOL130W	0.9081
YFL059W	YFL060C	0.5774
YFL059W	YMR095C	0.7253
YFL059W	YMR096W	0.5774
YFL059W	YNL333W	0.7253
YFL059W	YNL334C	0.7253
YFL060C	YMR096W	0.5774
YFR001W	YGR103W	0.8087
YFR001W	YHR052W	0.7380
YFR001W	YKL172W	0.8760
YFR001W	YKL185W	0.5293
YFR001W	YLL008W	0.7380
YFR001W	YLL045C	0.7380
YFR001W	YLR002C	0.7380
YFR001W	YMR229C	0.7380
YFR001W	YMR290C	0.7380
YFR001W	YNL002C	0.7380
YFR001W	YNL061W	0.9313
YFR001W	YOL077C	0.6147
YFR001W	YOR063W	0.7380
YFR001W	YOR206W	0.6147
YFR001W	YPL093W	0.7380
YFR001W	YPR016C	0.9743
YFR002W	YGL172W	0.9994
YFR002W	YGR119C	0.9993
YFR002W	YJL039C	0.8566

YFR002W	YJL041W	0.9999
YFR002W	YMR153W	0.9999
YFR002W	YMR308C	0.9768
YFR003C	YKL193C	0.9822
YFR003C	YML016C	0.9559
YFR004W	YFR010W	0.9995
YFR004W	YFR052W	0.9999
YFR004W	YGL004C	0.9968
YFR004W	YGL008C	0.7380
YFR004W	YGL009C	0.7380
YFR004W	YGL026C	0.7380
YFR004W	YGL030W	0.7380
YFR004W	YGL037C	0.7380
YFR004W	YGL048C	0.9902
YFR004W	YGL076C	0.7380
YFR004W	YGL123W	0.7380
YFR004W	YGL135W	0.7380
YFR004W	YGL137W	0.7380
YFR004W	YGL147C	0.7380
YFR004W	YGL148W	0.7380
YFR004W	YGL157W	0.7380
YFR004W	YGL195W	0.7380
YFR004W	YGL202W	0.7380
YFR004W	YGL207W	0.7380
YFR004W	YGL234W	0.7380
YFR004W	YGL253W	0.7380
YFR004W	YGR061C	0.7380
YFR004W	YGR085C	0.7380
YFR004W	YGR086C	0.7380
YFR004W	YGR094W	0.7380
YFR004W	YGR124W	0.7380
YFR004W	YGR155W	0.7380
YFR004W	YGR180C	0.7380
YFR004W	YGR187C	0.7380
YFR004W	YGR192C	0.7380
YFR004W	YGR209C	0.7380
YFR004W	YGR211W	0.7380
YFR004W	YGR214W	0.7380
YFR004W	YGR232W	0.9988
YFR004W	YGR234W	0.7380
YFR004W	YGR240C	0.7380
YFR004W	YGR254W	0.7380
YFR004W	YGR264C	0.7380
YFR004W	YGR285C	0.7380
YFR004W	YHL001W	0.7380
YFR004W	YHL015W	0.7380
YFR004W	YHL030W	0.9313

YFR004W	YHR019C	0.7380
YFR004W	YHR020W	0.7380
YFR004W	YHR021C	0.7380
YFR004W	YHR027C	0.9984
YFR004W	YHR039C	0.7380
YFR004W	YHR063C	0.7380
YFR004W	YHR064C	0.7380
YFR004W	YHR068W	0.7380
YFR004W	YHR074W	0.7380
YFR004W	YHR104W	0.7380
YFR004W	YHR108W	0.7380
YFR004W	YHR128W	0.7380
YFR004W	YHR170W	0.7380
YFR004W	YHR174W	0.7380
YFR004W	YHR179W	0.7380
YFR004W	YHR183W	0.7380
YFR004W	YHR190W	0.7380
YFR004W	YHR193C	0.7380
YFR004W	YHR200W	1.0000
YFR004W	YHR203C	0.7380
YFR004W	YHR208W	0.7380
YFR004W	YIL053W	0.7380
YFR004W	YIL075C	0.9895
YFR004W	YIL078W	0.7380
YFR004W	YIL109C	0.7380
YFR004W	YIL118W	0.7380
YFR004W	YIL142W	0.7380
YFR004W	YJL008C	0.7380
YFR004W	YJL014W	0.7380
YFR004W	YJL052W	0.7380
YFR004W	YJL088W	0.7380
YFR004W	YJL101C	0.7380
YFR004W	YJL111W	0.7380
YFR004W	YJL138C	0.7380
YFR004W	YJL167W	0.7380
YFR004W	YJL190C	0.7380
YFR004W	YJR007W	0.7380
YFR004W	YJR009C	0.7380
YFR004W	YJR025C	0.7380
YFR004W	YJR065C	0.7380
YFR004W	YJR070C	0.7380
YFR004W	YJR072C	0.7380
YFR004W	YJR077C	0.7380
YFR004W	YJR105W	0.7380
YFR004W	YJR109C	0.7380
YFR004W	YJR121W	0.7380
YFR004W	YJR123W	0.7380

YFR004W	YJR137C	0.7380
YFR004W	YKL035W	0.7380
YFR004W	YKL056C	0.7380
YFR004W	YKL060C	0.7380
YFR004W	YKL081W	0.7380
YFR004W	YKL104C	0.7380
YFR004W	YKL120W	0.7380
YFR004W	YKL145W	0.9999
YFR004W	YKL152C	0.7380
YFR004W	YKL157W	0.7380
YFR004W	YKL181W	0.7380
YFR004W	YKL182W	0.7380
YFR004W	YKL210W	0.7380
YFR004W	YKL211C	0.7380
YFR004W	YLL018C	0.7380
YFR004W	YLL024C	0.7380
YFR004W	YLL026W	0.7380
YFR004W	YLL045C	0.7380
YFR004W	YLL050C	0.7380
YFR004W	YLR028C	0.7380
YFR004W	YLR044C	0.7380
YFR004W	YLR060W	0.7380
YFR004W	YLR075W	0.7380
YFR004W	YLR109W	0.7380
YFR004W	YLR150W	0.7380
YFR004W	YLR153C	0.7380
YFR004W	YLR179C	0.7380
YFR004W	YLR180W	0.7380
YFR004W	YLR192C	0.7380
YFR004W	YLR197W	0.7380
YFR004W	YLR216C	0.7380
YFR004W	YLR249W	0.7380
YFR004W	YLR270W	0.7380
YFR004W	YLR293C	0.7380
YFR004W	YLR300W	0.7380
YFR004W	YLR342W	0.7380
YFR004W	YLR354C	0.7380
YFR004W	YLR355C	0.7380
YFR004W	YLR359W	0.7380
YFR004W	YLR372W	0.7380
YFR004W	YLR421C	0.8566
YFR004W	YLR432W	0.7380
YFR004W	YML008C	0.7380
YFR004W	YML022W	0.7380
YFR004W	YML028W	0.7380
YFR004W	YML057W	0.7380
YFR004W	YML063W	0.7380

YFR004W	YML085C	0.7380
YFR004W	YML086C	0.7380
YFR004W	YML100W	0.7380
YFR004W	YML106W	0.7380
YFR004W	YML126C	0.7380
YFR004W	YMR012W	0.7380
YFR004W	YMR079W	0.7380
YFR004W	YMR099C	0.7380
YFR004W	YMR116C	0.7380
YFR004W	YMR186W	0.7380
YFR004W	YMR191W	0.7380
YFR004W	YMR202W	0.7380
YFR004W	YMR205C	0.7380
YFR004W	YMR217W	0.7380
YFR004W	YMR226C	0.7380
YFR004W	YMR235C	0.7380
YFR004W	YMR242C	0.7380
YFR004W	YMR246W	0.7380
YFR004W	YMR261C	0.7380
YFR004W	YMR276W	0.9266
YFR004W	YMR309C	0.7380
YFR004W	YMR314W	0.8566
YFR004W	YNL007C	0.7380
YFR004W	YNL010W	0.7380
YFR004W	YNL014W	0.7380
YFR004W	YNL055C	0.7380
YFR004W	YNL064C	0.7380
YFR004W	YNL071W	0.7380
YFR004W	YNL079C	0.7380
YFR004W	YNL104C	0.7380
YFR004W	YNL112W	0.7380
YFR004W	YNL121C	0.7380
YFR004W	YNL138W	0.7380
YFR004W	YNL178W	0.7380
YFR004W	YNL209W	0.7380
YFR004W	YNL239W	0.7380
YFR004W	YNL241C	0.7380
YFR004W	YNL281W	0.7380
YFR004W	YNL287W	0.7380
YFR004W	YNR001C	0.7380
YFR004W	YNR035C	0.7380
YFR004W	YNR043W	0.7380
YFR004W	YOL040C	0.7380
YFR004W	YOL058W	0.7380
YFR004W	YOL061W	0.7380
YFR004W	YOL086C	0.7380
YFR004W	YOL097C	0.7380

YFR004W	YOL109W	0.7380
YFR004W	YOR007C	0.7380
YFR004W	YOR026W	0.7380
YFR004W	YOR027W	0.7380
YFR004W	YOR046C	0.7380
YFR004W	YOR117W	0.9995
YFR004W	YOR151C	0.7380
YFR004W	YOR168W	0.7380
YFR004W	YOR184W	0.7380
YFR004W	YOR187W	0.7380
YFR004W	YOR204W	0.7380
YFR004W	YOR259C	0.9266
YFR004W	YOR261C	1.0000
YFR004W	YOR303W	0.7380
YFR004W	YOR317W	0.7380
YFR004W	YOR323C	0.7380
YFR004W	YOR335C	0.7380
YFR004W	YOR341W	0.7380
YFR004W	YOR362C	0.9780
YFR004W	YOR375C	0.7380
YFR004W	YPL004C	0.7380
YFR004W	YPL028W	0.7380
YFR004W	YPL048W	0.7380
YFR004W	YPL061W	0.7380
YFR004W	YPL106C	0.7380
YFR004W	YPL111W	0.7380
YFR004W	YPL131W	0.7380
YFR004W	YPL145C	0.7380
YFR004W	YPL160W	0.7380
YFR004W	YPL218W	0.7380
YFR004W	YPL226W	0.7380
YFR004W	YPL231W	0.7380
YFR004W	YPL235W	0.7380
YFR004W	YPL240C	0.7380
YFR004W	YPL262W	0.7380
YFR004W	YPR010C	0.7380
YFR004W	YPR033C	0.7380
YFR004W	YPR036W	0.7380
YFR004W	YPR041W	0.7380
YFR004W	YPR069C	0.7380
YFR004W	YPR074C	0.7380
YFR004W	YPR108W	0.9989
YFR004W	YPR145W	0.7380
YFR004W	YPR163C	0.7380
YFR004W	YPR181C	0.7380
YFR004W	YPR183W	0.7380
YFR008W	YMR029C	0.7909

YFR008W	YMR052W	0.9716
YFR008W	YPR046W	0.5774
YFR009W	YGL195W	0.9997
YFR009W	YKL029C	0.7380
YFR010W	YFR052W	0.7380
YFR010W	YGL048C	0.9743
YFR010W	YHL030W	0.9787
YFR010W	YHR027C	0.9648
YFR010W	YHR200W	0.9743
YFR010W	YIL075C	0.9313
YFR010W	YKL145W	0.9973
YFR010W	YLL039C	0.7380
YFR010W	YLR421C	0.7380
YFR010W	YMR314W	0.7380
YFR010W	YOR117W	0.7380
YFR010W	YOR259C	0.9313
YFR010W	YOR261C	0.9648
YFR010W	YPR108W	0.8659
YFR011C	YNL113W	0.6672
YFR013W	YGL133W	0.7380
YFR013W	YOL004W	0.7380
YFR013W	YPL082C	0.7380
YFR015C	YJL137C	0.6672
YFR015C	YLR258W	0.9000
YFR015C	YLR369W	0.7380
YFR017C	YPL031C	0.6042
YFR017C	YPR184W	0.6672
YFR019W	YLR386W	0.9997
YFR019W	YNL325C	0.9941
YFR019W	YOL135C	0.7380
YFR021W	YFR040W	0.7380
YFR021W	YLR386W	0.7579
YFR021W	YNL242W	0.9946
YFR024C-A	YGL181W	0.5774
YFR024C-A	YGR268C	0.7253
YFR024C-A	YHR016C	0.6147
YFR024C-A	YJR083C	0.8591
YFR024C-A	YLR144C	0.8591
YFR024C-A	YMR192W	0.8591
YFR024C-A	YNL094W	0.7253
YFR024C-A	YNL243W	0.7380
YFR024C-A	YOR042W	0.9167
YFR024C-A	YOR181W	0.9997
YFR024C-A	YPR171W	0.9084
YFR027W	YJL019W	0.9843
YFR027W	YJL074C	0.7696
YFR027W	YMR076C	0.8655

YFR027W	YPL008W	0.9249
YFR028C	YGR052W	0.8566
YFR028C	YGR092W	0.9155
YFR028C	YHR061C	0.9138
YFR028C	YHR118C	0.9567
YFR028C	YIL035C	0.8566
YFR028C	YJL076W	1.0000
YFR028C	YJL095W	0.8566
YFR028C	YJL187C	0.8566
YFR028C	YJL194W	0.8655
YFR028C	YJR053W	0.9884
YFR028C	YJR063W	0.7380
YFR028C	YKL001C	0.8566
YFR028C	YKR010C	0.9954
YFR028C	YLR079W	0.9341
YFR028C	YML064C	0.9758
YFR028C	YMR001C	0.9899
YFR028C	YMR055C	0.9037
YFR028C	YMR270C	0.6672
YFR028C	YNL161W	0.8566
YFR028C	YNL307C	0.8566
YFR028C	YOR058C	0.8456
YFR028C	YOR061W	0.8566
YFR028C	YPL267W	0.9623
YFR031C	YIL144W	0.9099
YFR031C	YKL068W	0.8087
YFR031C	YLR086W	0.9939
YFR031C	YLR272C	0.9266
YFR031C-A	YGL031C	0.7380
YFR031C-A	YGL076C	0.7380
YFR031C-A	YGL099W	0.7380
YFR031C-A	YGL103W	0.7380
YFR031C-A	YGR085C	0.7380
YFR031C-A	YGR103W	0.9499
YFR031C-A	YGR204W	0.7380
YFR031C-A	YHR010W	0.7380
YFR031C-A	YJL191W	0.7380
YFR031C-A	YKL009W	0.9215
YFR031C-A	YKL180W	0.7380
YFR031C-A	YLL045C	0.7380
YFR031C-A	YLR029C	0.7380
YFR031C-A	YLR075W	0.7380
YFR031C-A	YLR340W	0.9266
YFR031C-A	YLR367W	0.7380
YFR031C-A	YML073C	0.7380
YFR031C-A	YMR142C	0.7380
YFR031C-A	YMR242C	0.7380

YFR031C-A	YNL069C	0.7380
YFR031C-A	YNL112W	0.7380
YFR031C-A	YNL178W	0.7380
YFR031C-A	YOL120C	0.8659
YFR031C-A	YOL127W	0.7380
YFR031C-A	YOR048C	0.7380
YFR031C-A	YOR063W	0.7380
YFR031C-A	YOR204W	0.7380
YFR031C-A	YPL009C	0.7380
YFR031C-A	YPL093W	0.7380
YFR031C-A	YPL143W	0.7380
YFR031C-A	YPL198W	0.7380
YFR031C-A	YPL249C-A	0.7380
YFR034C	YMR308C	0.9799
YFR034C	YOL001W	0.9998
YFR034C	YOL108C	0.9099
YFR034C	YPL031C	1.0000
YFR034C	YPR086W	0.9249
YFR036W	YHR166C	0.9887
YFR036W	YKL022C	1.0000
YFR036W	YNL172W	0.8566
YFR037C	YGR056W	0.9313
YFR037C	YGR275W	0.9908
YFR037C	YHR056C	0.8659
YFR037C	YIL126W	0.9999
YFR037C	YKR008W	0.9908
YFR037C	YLR033W	0.9953
YFR037C	YLR321C	0.9820
YFR037C	YLR347C	0.7380
YFR037C	YLR357W	0.9996
YFR037C	YML127W	0.9940
YFR037C	YMR033W	0.9820
YFR037C	YMR072W	0.7380
YFR037C	YMR091C	0.9969
YFR037C	YNR003C	0.7380
YFR037C	YOL004W	0.7380
YFR037C	YOR116C	0.7380
YFR037C	YPL082C	0.8087
YFR037C	YPR034W	0.9820
YFR040W	YPR040W	0.9447
YFR042W	YPR159W	0.8179
YFR044C	YNL191W	0.9215
YFR046C	YMR117C	0.7909
YFR047C	YNL189W	0.5774
YFR049W	YIL125W	0.7380
YFR050C	YGL011C	0.9313
YFR050C	YGR135W	0.7380

YFR050C	YGR253C	0.7380
YFR050C	YHR052W	0.9191
YFR050C	YHR200W	0.7380
YFR050C	YJL001W	0.9990
YFR050C	YML092C	0.9313
YFR050C	YMR314W	0.7380
YFR050C	YOL038W	0.9624
YFR050C	YOR157C	0.9980
YFR050C	YOR261C	0.9266
YFR050C	YOR362C	0.9624
YFR050C	YPR103W	0.9787
YFR051C	YGL137W	0.9908
YFR051C	YIL076W	0.9648
YFR051C	YKR067W	0.7380
YFR051C	YNL258C	0.9710
YFR051C	YNL284C	0.7380
YFR051C	YNL287W	0.8659
YFR051C	YNL304W	0.9249
YFR051C	YPL010W	0.8990
YFR052W	YGL048C	0.9648
YFR052W	YGR232W	0.9850
YFR052W	YHL030W	0.9313
YFR052W	YHR027C	0.9944
YFR052W	YHR200W	0.9997
YFR052W	YIL075C	0.9624
YFR052W	YKL145W	0.9999
YFR052W	YLR421C	0.7380
YFR052W	YMR314W	0.8659
YFR052W	YNL103W	0.9249
YFR052W	YOR117W	0.8659
YFR052W	YOR259C	0.9313
YFR052W	YOR261C	0.9970
YFR052W	YPR108W	0.9992
YGL001C	YGL012W	0.9928
YGL001C	YGR060W	0.9923
YGL001C	YGR175C	0.9151
YGL001C	YHR007C	0.9151
YGL001C	YHR072W	0.9762
YGL001C	YHR190W	0.9762
YGL001C	YLR056W	0.9762
YGL001C	YLR100W	0.9151
YGL001C	YML008C	0.9762
YGL001C	YMR202W	0.9762
YGL001C	YNL280C	0.9928
YGL003C	YHR166C	0.9780
YGL003C	YIL142W	0.8566
YGL003C	YKL022C	0.9215

YGL003C	YKL101W	1.0000
YGL003C	YMR001C	0.9894
YGL003C	YPL256C	0.8566
YGL003C	YPL267W	1.0000
YGL003C	YPR119W	0.9991
YGL004C	YGL048C	0.9977
YGL004C	YGR232W	0.9739
YGL004C	YHR027C	0.8566
YGL004C	YIL007C	0.9215
YGL004C	YKL145W	0.9958
YGL004C	YOR117W	0.9887
YGL004C	YOR259C	0.9616
YGL004C	YOR261C	0.9598
YGL004C	YPR108W	0.8566
YGL005C	YGL223C	0.7380
YGL005C	YGR120C	0.9850
YGL005C	YML071C	0.9948
YGL005C	YNL041C	0.9764
YGL005C	YNL051W	0.9879
YGL005C	YPR105C	0.9266
YGL008C	YKR039W	0.9165
YGL008C	YLL039C	0.9266
YGL011C	YGR135W	0.9313
YGL011C	YGR253C	0.7380
YGL011C	YHR200W	0.7380
YGL011C	YIL075C	0.8628
YGL011C	YJL001W	0.9648
YGL011C	YML092C	0.9931
YGL011C	YMR308C	0.7380
YGL011C	YMR314W	0.9313
YGL011C	YOL038W	0.9950
YGL011C	YOR157C	0.7380
YGL011C	YOR362C	0.9915
YGL011C	YPR103W	0.9820
YGL012W	YGR060W	0.9762
YGL012W	YGR175C	0.9151
YGL012W	YHL007C	0.9151
YGL012W	YHR007C	0.9151
YGL012W	YLR100W	0.9762
YGL012W	YML008C	0.9762
YGL012W	YMR015C	0.9151
YGL012W	YNL280C	0.9151
YGL013C	YHR178W	0.9249
YGL013C	YMR308C	0.9215
YGL013C	YOL051W	0.9982
YGL015C	YLR319C	0.5774
YGL016W	YKL058W	0.9249

YGL016W	YMR047C	0.9186
YGL016W	YOR194C	0.9249
YGL016W	YOR230W	0.9730
YGL017W	YLR234W	0.9099
YGL019W	YGL207W	0.9869
YGL019W	YGR068C	0.6672
YGL019W	YGR090W	0.9820
YGL019W	YGR116W	0.7380
YGL019W	YIL035C	0.9985
YGL019W	YKL160W	0.8998
YGL019W	YLR418C	0.7380
YGL019W	YML069W	0.8659
YGL019W	YMR172W	0.9313
YGL019W	YOL004W	0.7380
YGL019W	YOL145C	0.8659
YGL019W	YOR039W	0.9931
YGL019W	YOR061W	0.9865
YGL022W	YJL002C	1.0000
YGL022W	YML019W	0.9989
YGL022W	YMR149W	0.9987
YGL022W	YOR085W	0.9999
YGL022W	YOR103C	0.9780
YGL025C	YGL127C	0.7380
YGL025C	YGL151W	0.9583
YGL025C	YGR104C	0.9992
YGL025C	YHR041C	0.9871
YGL025C	YHR058C	0.9981
YGL025C	YLR071C	1.0000
YGL025C	YNL236W	0.9934
YGL025C	YNR010W	0.9266
YGL025C	YOL051W	0.9994
YGL025C	YOL135C	0.9994
YGL025C	YOR174W	0.9991
YGL025C	YPL248C	0.9215
YGL025C	YPR070W	0.9966
YGL025C	YPR168W	0.7380
YGL030W	YGR090W	0.7380
YGL030W	YGR103W	0.7380
YGL030W	YHR052W	0.8659
YGL030W	YNL110C	0.8659
YGL030W	YNL132W	0.7380
YGL030W	YOR310C	0.7380
YGL031C	YGR214W	0.7380
YGL031C	YHL033C	0.8566
YGL031C	YKL009W	0.9215
YGL031C	YOR063W	0.8566
YGL035C	YGL253W	0.9993

YGL037C	YNL189W	0.5774
YGL043W	YGL070C	0.8028
YGL043W	YGR005C	0.9186
YGL043W	YIL021W	0.7380
YGL043W	YJR132W	0.9215
YGL043W	YLR055C	0.9099
YGL043W	YOL005C	0.7380
YGL044C	YGR047C	0.9626
YGL044C	YMR061W	1.0000
YGL044C	YOR250C	0.9904
YGL044C	YPR086W	0.9215
YGL045W	YPL065W	0.9249
YGL048C	YGR184C	0.9790
YGL048C	YGR232W	0.9985
YGL048C	YHL030W	0.7380
YGL048C	YHR027C	1.0000
YGL048C	YHR200W	0.9979
YGL048C	YIL007C	0.9747
YGL048C	YIL075C	0.7380
YGL048C	YKL010C	0.9983
YGL048C	YKL145W	1.0000
YGL048C	YLR421C	0.9021
YGL048C	YOR117W	0.9822
YGL048C	YOR259C	0.9892
YGL048C	YOR261C	0.8659
YGL048C	YPL248C	0.9995
YGL048C	YPR108W	0.9808
YGL049C	YGR162W	0.7380
YGL049C	YHL034C	0.7380
YGL049C	YHR086W	0.7380
YGL049C	YIR001C	0.9313
YGL049C	YJL138C	0.9298
YGL049C	YLR175W	0.8659
YGL049C	YMR125W	0.9313
YGL049C	YNL016W	0.6147
YGL049C	YNL244C	0.9790
YGL049C	YNL251C	0.7380
YGL049C	YOL139C	0.9648
YGL049C	YPL178W	0.9298
YGL049C	YPR041W	0.9249
YGL058W	YGR184C	0.9999
YGL058W	YLR024C	0.9902
YGL058W	YMR100W	0.8566
YGL061C	YGR113W	1.0000
YGL061C	YKL052C	0.9726
YGL061C	YKL138C-A	0.8967
YGL061C	YKR037C	1.0000

YGL061C	YKR083C	0.9822
YGL066W	YGL112C	0.9648
YGL066W	YGR252W	0.9743
YGL066W	YHR099W	0.9499
YGL066W	YLR055C	0.9726
YGL066W	YMR223W	0.9904
YGL066W	YMR236W	0.9499
YGL066W	YOL148C	0.9313
YGL066W	YPL047W	0.9980
YGL066W	YPL082C	0.8953
YGL066W	YPL254W	0.8659
YGL070C	YGR005C	0.9648
YGL070C	YGR063C	0.7380
YGL070C	YGR186W	0.9313
YGL070C	YIL021W	0.9999
YGL070C	YJL140W	0.9969
YGL070C	YML010W	0.7380
YGL070C	YOL005C	0.9956
YGL070C	YOR151C	0.9997
YGL070C	YOR210W	0.9266
YGL070C	YOR224C	0.9984
YGL070C	YPL129W	0.9648
YGL070C	YPR093C	0.8087
YGL070C	YPR187W	0.9984
YGL071W	YPR052C	0.9249
YGL073W	YOR178C	0.9249
YGL075C	YJL019W	0.9249
YGL075C	YLR457C	0.9690
YGL075C	YMR117C	0.9997
YGL075C	YNL107W	0.8965
YGL075C	YPL255W	0.9975
YGL076C	YGL111W	0.7380
YGL076C	YGR103W	0.7380
YGL076C	YHR088W	0.7380
YGL076C	YHR200W	0.8087
YGL076C	YLR432W	0.7380
YGL076C	YOL120C	0.7380
YGL076C	YOR206W	0.7380
YGL076C	YPL093W	0.7380
YGL078C	YGR159C	0.7380
YGL078C	YHR089C	0.8028
YGL078C	YLR175W	0.7380
YGL078C	YLR197W	0.7380
YGL086W	YGR188C	0.9215
YGL086W	YJL030W	1.0000
YGL086W	YMR153W	0.9790
YGL086W	YOR026W	0.9938

YGL087C	YMR140W	0.6147
YGL090W	YLR265C	1.0000
YGL090W	YLR424W	0.9414
YGL090W	YOR005C	1.0000
YGL092W	YGL100W	1.0000
YGL092W	YGL172W	0.8087
YGL092W	YJR042W	1.0000
YGL092W	YKL057C	1.0000
YGL092W	YKL068W	0.8087
YGL092W	YKR082W	0.7380
YGL092W	YLR208W	1.0000
YGL092W	YMR047C	0.9841
YGL093W	YIR010W	0.9841
YGL093W	YPL233W	0.7380
YGL095C	YOL018C	0.9994
YGL095C	YOR036W	0.9215
YGL096W	YJR022W	0.5774
YGL097W	YLR293C	0.9997
YGL097W	YLR335W	0.9783
YGL097W	YNL030W	0.9626
YGL097W	YNL031C	0.8998
YGL097W	YNL189W	0.9107
YGL097W	YOR185C	0.9285
YGL098W	YGL145W	0.9895
YGL098W	YKL196C	0.9266
YGL098W	YLR268W	0.9598
YGL098W	YLR440C	0.9941
YGL098W	YNL258C	0.9895
YGL098W	YOR075W	0.9984
YGL099W	YGR245C	0.8659
YGL099W	YHR010W	0.7380
YGL099W	YHR170W	1.0000
YGL099W	YIR026C	0.9845
YGL099W	YLL045C	0.9266
YGL099W	YLR075W	0.8566
YGL099W	YLR340W	0.9942
YGL099W	YMR194W	0.7380
YGL099W	YOR063W	0.9624
YGL099W	YPL131W	0.9794
YGL099W	YPR016C	0.9793
YGL100W	YJR042W	1.0000
YGL100W	YKL057C	0.9946
YGL100W	YKR082W	0.7380
YGL100W	YLR208W	0.9902
YGL100W	YMR047C	0.9406
YGL100W	YPL169C	0.8566
YGL103W	YHR052W	0.7380

YGL103W	YML072C	0.7380
YGL103W	YNL110C	0.8659
YGL103W	YPL012W	0.7380
YGL105W	YGL245W	1.0000
YGL105W	YGR264C	1.0000
YGL106W	YHR023W	0.9970
YGL106W	YLR429W	0.7380
YGL106W	YOR326W	1.0000
YGL106W	YPL242C	0.9977
YGL106W	YPR171W	0.7380
YGL111W	YGR103W	0.9971
YGL111W	YHR052W	0.9021
YGL111W	YHR066W	0.9285
YGL111W	YHR088W	0.9743
YGL111W	YKL172W	0.8087
YGL111W	YKR081C	0.9021
YGL111W	YLL008W	0.8087
YGL111W	YLL034C	0.9871
YGL111W	YLR002C	0.9850
YGL111W	YLR276C	0.8087
YGL111W	YMR049C	0.9743
YGL111W	YMR290C	0.9021
YGL111W	YNL002C	0.8087
YGL111W	YNL061W	0.9021
YGL111W	YNL110C	0.9743
YGL111W	YOL077C	0.9021
YGL111W	YOR063W	0.9464
YGL111W	YOR206W	0.9021
YGL111W	YOR272W	0.9897
YGL111W	YPL093W	0.9989
YGL111W	YPL131W	0.9021
YGL111W	YPL211W	0.7380
YGL111W	YPR016C	0.9726
YGL112C	YGR094W	0.7380
YGL112C	YGR252W	0.9993
YGL112C	YGR274C	1.0000
YGL112C	YHR099W	0.9313
YGL112C	YLR055C	1.0000
YGL112C	YML007W	0.7380
YGL112C	YML015C	0.9972
YGL112C	YML098W	0.9820
YGL112C	YML114C	0.9852
YGL112C	YMR005W	0.9990
YGL112C	YMR223W	0.9928
YGL112C	YMR227C	0.9980
YGL112C	YMR236W	1.0000
YGL112C	YOL148C	0.9999

YGL112C	YPL011C	0.9648
YGL112C	YPL047W	0.9904
YGL112C	YPL082C	0.9707
YGL112C	YPL129W	0.7380
YGL112C	YPL248C	0.9669
YGL112C	YPL254W	0.9933
YGL112C	YPR072W	0.8566
YGL113W	YJL090C	0.9977
YGL113W	YLR103C	0.9974
YGL113W	YPL153C	0.7696
YGL113W	YPR120C	0.7543
YGL115W	YGL208W	0.9999
YGL115W	YOR018W	0.8566
YGL115W	YOR267C	0.7380
YGL115W	YPL046C	0.9099
YGL116W	YGR188C	0.8640
YGL116W	YHR166C	0.9825
YGL116W	YIL142W	0.8566
YGL116W	YJL013C	1.0000
YGL116W	YJL030W	1.0000
YGL116W	YKL022C	0.9215
YGL116W	YKL101W	0.9981
YGL116W	YOR026W	0.9586
YGL116W	YPL106C	0.7380
YGL116W	YPR119W	0.9215
YGL120C	YGL128C	0.9869
YGL120C	YGL173C	0.9021
YGL120C	YGR159C	0.8087
YGL120C	YGR280C	0.8798
YGL120C	YHR052W	0.8087
YGL120C	YHR089C	0.7187
YGL120C	YHR165C	0.9743
YGL120C	YJL076W	0.9099
YGL120C	YJL148W	0.7187
YGL120C	YJL190C	0.7380
YGL120C	YJR145C	0.7380
YGL120C	YKL014C	0.8603
YGL120C	YKL173W	0.9743
YGL120C	YKR022C	0.9988
YGL120C	YKR092C	0.8087
YGL120C	YLL036C	0.9464
YGL120C	YLR117C	0.9499
YGL120C	YLR197W	0.8087
YGL120C	YLR424W	1.0000
YGL120C	YMR049C	0.8087
YGL120C	YMR116C	0.8659
YGL120C	YMR213W	0.7380

YGL120C	YNL112W	0.7187
YGL120C	YNL132W	0.8087
YGL120C	YNL224C	0.9991
YGL120C	YOR096W	0.7380
YGL120C	YOR119C	0.8087
YGL120C	YOR310C	0.9743
YGL120C	YOR341W	0.8953
YGL120C	YPL151C	0.9499
YGL122C	YHR135C	0.2210
YGL122C	YIL092W	0.5774
YGL122C	YIL149C	0.8566
YGL122C	YKR095W	1.0000
YGL122C	YLR150W	0.7524
YGL122C	YLR177W	0.3858
YGL122C	YLR335W	0.5164
YGL122C	YML103C	0.5164
YGL122C	YMR129W	0.5164
YGL122C	YMR255W	1.0000
YGL122C	YNL016W	0.9836
YGL122C	YNL139C	0.8645
YGL122C	YOL123W	0.9273
YGL122C	YPL169C	0.9975
YGL122C	YPL178W	0.9258
YGL125W	YPL023C	0.9597
YGL127C	YGR104C	0.9850
YGL127C	YHR041C	0.7380
YGL127C	YHR058C	0.7380
YGL127C	YLR071C	0.7380
YGL127C	YNL236W	0.7380
YGL127C	YNR010W	0.7380
YGL127C	YOL051W	0.9266
YGL127C	YOL135C	0.9989
YGL127C	YOR174W	0.9523
YGL127C	YPR070W	0.9266
YGL127C	YPR168W	0.9824
YGL128C	YHR165C	0.9313
YGL128C	YKL173W	0.8659
YGL128C	YKR022C	0.7380
YGL128C	YLL036C	0.9313
YGL128C	YLR117C	0.9021
YGL128C	YLR275W	0.7380
YGL128C	YLR424W	0.9999
YGL128C	YMR213W	0.9313
YGL128C	YPL151C	0.7380
YGL129C	YGR165W	0.7380
YGL129C	YHL004W	0.9743
YGL129C	YJR113C	0.7380

YGL129C	YKL155C	0.8566
YGL129C	YNL137C	0.7380
YGL129C	YPL013C	0.7380
YGL130W	YGL207W	0.7380
YGL130W	YHR121W	0.7380
YGL130W	YML010W	0.7380
YGL130W	YMR309C	0.7380
YGL130W	YNL088W	0.7380
YGL130W	YPL228W	1.0000
YGL133W	YJL065C	0.9794
YGL133W	YOR304W	0.9971
YGL133W	YPL082C	0.9464
YGL134W	YLR258W	0.9778
YGL134W	YPL031C	0.9991
YGL135W	YLL039C	0.7380
YGL137W	YIL076W	0.9820
YGL137W	YKL081W	0.7380
YGL137W	YNL284C	0.7380
YGL137W	YNL287W	0.9648
YGL137W	YPL004C	0.7187
YGL137W	YPL010W	0.9483
YGL145W	YLR268W	0.7380
YGL145W	YLR440C	0.9895
YGL145W	YNL258C	1.0000
YGL145W	YOR075W	0.9895
YGL147C	YPR016C	0.7380
YGL150C	YJL081C	0.9999
YGL150C	YLR052W	0.9973
YGL150C	YNL030W	0.8603
YGL150C	YNL031C	0.8603
YGL150C	YNL059C	0.9974
YGL150C	YOR141C	1.0000
YGL150C	YOR189W	0.9285
YGL150C	YPL129W	0.9997
YGL150C	YPL235W	1.0000
YGL151W	YGR104C	0.9996
YGL151W	YHR041C	0.9266
YGL151W	YHR058C	0.9266
YGL151W	YLR071C	0.9266
YGL151W	YML007W	0.7380
YGL151W	YMR112C	0.7380
YGL151W	YNL025C	0.7380
YGL151W	YNL236W	0.9779
YGL151W	YNR010W	0.8659
YGL151W	YOL051W	0.8659
YGL151W	YOL135C	0.9648
YGL151W	YOR174W	0.8659

YGL151W	YPR070W	0.9779
YGL151W	YPR168W	0.7380
YGL153W	YGR077C	0.8603
YGL153W	YJL210W	0.9707
YGL153W	YLR191W	1.0000
YGL153W	YMR026C	0.9834
YGL153W	YNL214W	0.9965
YGL153W	YNL329C	0.7826
YGL153W	YOL044W	0.9124
YGL154C	YGL254W	0.7253
YGL156W	YKL103C	0.6672
YGL156W	YOL082W	0.9490
YGL156W	YOL083W	0.7909
YGL156W	YPR110C	0.7380
YGL158W	YLR113W	0.6672
YGL161C	YGL198W	0.9839
YGL161C	YGL212W	0.7909
YGL161C	YHR105W	0.7909
YGL162W	YHL007C	0.9099
YGL164C	YLR293C	0.9758
YGL170C	YMR047C	0.5774
YGL171W	YGR090W	0.8659
YGL172W	YGR119C	0.9997
YGL172W	YGR218W	0.9470
YGL172W	YJL041W	1.0000
YGL172W	YJR042W	0.9842
YGL172W	YKL057C	0.8087
YGL172W	YLR335W	0.6147
YGL172W	YLR347C	0.8921
YGL172W	YMR047C	0.9541
YGL173C	YGR054W	0.8659
YGL173C	YGR086C	0.7380
YGL173C	YGR162W	0.7380
YGL173C	YHL034C	0.8659
YGL173C	YJL124C	0.9313
YGL173C	YJR022W	0.7837
YGL173C	YKL023W	0.7380
YGL173C	YLR175W	0.8659
YGL173C	YLR438C-A	0.9634
YGL173C	YML056C	0.7380
YGL173C	YNL118C	0.8297
YGL173C	YNL147W	0.8659
YGL173C	YNL175C	0.7380
YGL173C	YOL139C	0.8659
YGL173C	YPL004C	0.7380
YGL174W	YIR005W	1.0000
YGL175C	YMR001C	0.9138

YGL178W	YGR040W	0.8965
YGL178W	YLR452C	0.9907
YGL178W	YNR052C	0.7891
YGL180W	YLR423C	0.9999
YGL180W	YNL272C	0.4640
YGL180W	YPR049C	0.9996
YGL180W	YPR185W	1.0000
YGL181W	YGR241C	0.7909
YGL181W	YHR016C	0.7909
YGL181W	YIL122W	0.5774
YGL181W	YIR006C	0.9099
YGL181W	YLL003W	0.9099
YGL181W	YLR188W	0.8965
YGL187C	YML129C	0.9215
YGL189C	YLR435W	0.7570
YGL190C	YGR161C	0.7380
YGL190C	YIL142W	0.7380
YGL190C	YJL014W	0.7380
YGL190C	YJR064W	0.7380
YGL190C	YJR138W	0.7380
YGL190C	YML109W	0.9998
YGL190C	YMR109W	0.6672
YGL190C	YMR273C	0.9808
YGL190C	YOR349W	0.7380
YGL194C	YIL112W	0.9813
YGL194C	YKR029C	0.9897
YGL194C	YMR273C	0.7380
YGL194C	YOL068C	0.8603
YGL195W	YGR173W	0.7891
YGL195W	YIL094C	0.8659
YGL195W	YKL029C	0.7380
YGL195W	YKL104C	0.7380
YGL195W	YLR180W	0.8659
YGL195W	YOR136W	0.7380
YGL197W	YJL098W	0.8659
YGL197W	YLR310C	0.9128
YGL197W	YMR308C	0.7380
YGL197W	YPL204W	0.7380
YGL198W	YHR105W	0.9138
YGL198W	YNL263C	0.9138
YGL200C	YLR026C	0.9555
YGL200C	YML012W	0.9855
YGL200C	YOR016C	0.6147
YGL200C	YPL218W	0.9881
YGL201C	YIL150C	0.9414
YGL201C	YLR103C	0.8603
YGL201C	YLR274W	0.7380

YGL201C	YPR019W	0.9991
YGL202W	YPL106C	0.7380
YGL206C	YGR167W	1.0000
YGL206C	YGR241C	0.9037
YGL206C	YHR108W	0.9616
YGL206C	YHR161C	0.9037
YGL206C	YIL094C	0.7380
YGL206C	YJR125C	0.8953
YGL206C	YKL135C	0.9215
YGL206C	YLR206W	0.9037
YGL206C	YNL243W	0.9168
YGL206C	YOR109W	0.8566
YGL206C	YOR181W	0.7380
YGL206C	YPL106C	0.8659
YGL206C	YPR029C	0.7891
YGL207W	YGL244W	0.9904
YGL207W	YGL252C	0.7380
YGL207W	YGR270W	0.8087
YGL207W	YIL021W	0.8566
YGL207W	YIL035C	0.9933
YGL207W	YJL047C	0.9640
YGL207W	YKL028W	0.9249
YGL207W	YLL039C	0.8566
YGL207W	YLR182W	0.9215
YGL207W	YLR418C	0.9975
YGL207W	YML010W	0.8566
YGL207W	YML069W	1.0000
YGL207W	YNL102W	0.9929
YGL207W	YNL282W	0.8603
YGL207W	YOL006C	0.7380
YGL207W	YOL054W	0.9499
YGL207W	YOL145C	0.9975
YGL207W	YOR039W	0.9966
YGL207W	YOR061W	0.9933
YGL207W	YOR123C	0.9990
YGL207W	YPR052C	0.9215
YGL210W	YJL204C	0.8965
YGL210W	YNL044W	0.9099
YGL210W	YNL263C	0.9138
YGL210W	YNL272C	0.9723
YGL210W	YOR070C	0.9138
YGL210W	YOR326W	0.9994
YGL211W	YHR111W	0.8603
YGL211W	YIL008W	0.9551
YGL211W	YNL119W	0.9613
YGL212W	YKL196C	0.9875
YGL212W	YLR093C	0.9997

YGL212W	YLR148W	0.9967
YGL212W	YLR396C	1.0000
YGL212W	YMR197C	1.0000
YGL212W	YMR231W	0.9435
YGL212W	YNL263C	0.7909
YGL212W	YOR106W	1.0000
YGL212W	YPL045W	0.9215
YGL212W	YPL246C	0.7909
YGL213C	YHL022C	0.9784
YGL213C	YLR398C	0.9996
YGL213C	YOR076C	0.9215
YGL213C	YPR189W	1.0000
YGL215W	YPL031C	0.9440
YGL220W	YLL029W	0.8566
YGL223C	YGR120C	0.9996
YGL223C	YLR026C	0.9249
YGL223C	YML071C	0.9933
YGL223C	YNL041C	0.9764
YGL223C	YNL051W	0.9879
YGL223C	YPR105C	0.9157
YGL226C-A	YJL002C	0.9215
YGL226C-A	YML019W	0.9555
YGL226C-A	YOR085W	0.9865
YGL227W	YIL017C	0.9021
YGL227W	YIL097W	0.9021
YGL227W	YMR135C	0.9634
YGL229C	YPR040W	0.6672
YGL233W	YIL068C	0.9991
YGL233W	YJL085W	0.9266
YGL233W	YLR166C	1.0000
YGL233W	YPR055W	1.0000
YGL237C	YKL109W	0.9808
YGL237C	YLR423C	0.6672
YGL237C	YOR358W	0.9991
YGL238W	YLR293C	1.0000
YGL238W	YNL189W	1.0000
YGL240W	YHR166C	0.9674
YGL240W	YIR025W	0.9285
YGL240W	YKL022C	1.0000
YGL240W	YLR102C	0.7570
YGL240W	YLR127C	0.9987
YGL240W	YNL172W	0.9964
YGL240W	YOR249C	0.8603
YGL241W	YKR048C	1.0000
YGL241W	YLR293C	0.9282
YGL241W	YOL012C	0.9989
YGL241W	YPR086W	0.9282

YGL241W	YPR093C	0.9107
YGL242C	YMR109W	0.8028
YGL244W	YIL035C	0.8659
YGL244W	YLR418C	0.9999
YGL244W	YML069W	0.9904
YGL244W	YOL145C	1.0000
YGL244W	YOR123C	0.9999
YGL245W	YGR040W	0.5779
YGL245W	YGR264C	0.9648
YGL245W	YPR010C	0.7380
YGL246C	YLL045C	0.7380
YGL246C	YLR340W	0.8087
YGL246C	YMR242C	0.8087
YGL246C	YOR048C	0.9990
YGL252C	YNL076W	0.9992
YGL252C	YNL132W	0.7380
YGL253W	YGR218W	0.9249
YGL253W	YKL038W	0.9099
YGR002C	YHR090C	0.9980
YGR002C	YHR099W	0.9726
YGR002C	YJL081C	0.9948
YGR002C	YJR082C	0.8659
YGR002C	YLR385C	0.9021
YGR002C	YLR399C	0.8566
YGR002C	YNL107W	0.9998
YGR002C	YNL136W	0.9313
YGR002C	YOL012C	0.9805
YGR002C	YOR244W	0.9997
YGR002C	YPL235W	0.9499
YGR002C	YPR023C	0.9464
YGR003W	YJR052W	0.9555
YGR003W	YOL133W	0.7909
YGR004W	YLR324W	0.8965
YGR005C	YGR104C	0.9780
YGR005C	YGR186W	1.0000
YGR005C	YIL021W	0.9963
YGR005C	YJL140W	0.9266
YGR005C	YLR418C	0.9555
YGR005C	YML010W	0.8659
YGR005C	YOL005C	0.9860
YGR005C	YOR151C	0.9860
YGR005C	YOR210W	0.7380
YGR005C	YOR224C	0.7380
YGR005C	YPL129W	0.9989
YGR005C	YPR187W	0.9266
YGR009C	YLR096W	0.8655
YGR009C	YMR183C	0.9249

YGR009C	YNR049C	0.9780
YGR009C	YOR327C	0.9948
YGR009C	YPL232W	1.0000
YGR009C	YPR032W	0.9983
YGR010W	YLR328W	0.9821
YGR013W	YGR074W	0.9499
YGR013W	YHR086W	0.9951
YGR013W	YIL061C	0.9963
YGR013W	YKL012W	0.9985
YGR013W	YLR147C	0.9021
YGR013W	YLR275W	0.9499
YGR013W	YLR298C	0.9869
YGR013W	YML046W	0.9499
YGR013W	YMR125W	0.9648
YGR013W	YPL178W	0.9313
YGR013W	YPR182W	0.8659
YGR014W	YLR229C	0.9758
YGR017W	YLR410W	0.7380
YGR020C	YHR039C-A	0.9972
YGR020C	YLL039C	0.7380
YGR020C	YLR447C	0.9483
YGR020C	YOR270C	0.9988
YGR020C	YOR332W	0.9313
YGR020C	YPR036W	0.9855
YGR029W	YJR048W	0.9827
YGR029W	YKL195W	1.0000
YGR030C	YHR062C	0.9266
YGR030C	YIR015W	0.9099
YGR030C	YLR145W	0.9598
YGR030C	YNL221C	0.9984
YGR032W	YLL039C	0.7380
YGR033C	YIL022W	0.9780
YGR033C	YJL104W	0.9780
YGR033C	YJL143W	0.9999
YGR033C	YLR008C	0.9780
YGR033C	YNL131W	0.9997
YGR033C	YNR017W	1.0000
YGR033C	YPL063W	0.9995
YGR038W	YKL212W	0.8953
YGR038W	YLR350W	0.8603
YGR038W	YMR296C	0.9918
YGR040W	YHR084W	0.9995
YGR040W	YKR048C	0.5779
YGR040W	YLR310C	0.8655
YGR040W	YLR362W	0.9893
YGR040W	YNL053W	0.9459
YGR040W	YPL049C	0.9999

YGR040W	YPL115C	0.6147
YGR040W	YPR115W	0.7380
YGR041W	YLR353W	0.9215
YGR041W	YOR301W	0.9215
YGR047C	YGR156W	0.8940
YGR047C	YGR246C	0.9999
YGR047C	YHR143W-A	0.8783
YGR047C	YLR115W	0.8998
YGR047C	YLR277C	0.8998
YGR047C	YNL039W	0.9926
YGR047C	YOR110W	0.9879
YGR047C	YOR250C	0.8998
YGR047C	YPL007C	0.7380
YGR048W	YKL020C	0.9215
YGR048W	YKL213C	0.9555
YGR048W	YKR002W	0.9099
YGR048W	YML013W	0.9409
YGR052W	YJR066W	0.8566
YGR054W	YNL207W	0.8659
YGR056W	YGR275W	0.8659
YGR056W	YHR056C	0.9583
YGR056W	YIL126W	0.9995
YGR056W	YKR008W	0.9891
YGR056W	YLR033W	0.9313
YGR056W	YLR321C	0.9891
YGR056W	YLR357W	0.9871
YGR056W	YML127W	0.8659
YGR056W	YMR033W	0.9940
YGR056W	YMR091C	0.9313
YGR056W	YMR224C	0.9747
YGR056W	YPR034W	0.9891
YGR058W	YGR136W	0.5774
YGR060W	YGR175C	0.9361
YGR060W	YHR007C	0.9806
YGR060W	YHR072W	0.9151
YGR060W	YHR190W	0.9762
YGR060W	YJL117W	0.4335
YGR060W	YLR056W	0.9946
YGR060W	YLR100W	0.9993
YGR060W	YML008C	0.9928
YGR060W	YMR015C	0.9762
YGR060W	YMR202W	0.9151
YGR060W	YPL227C	0.4335
YGR061C	YLR386W	0.5774
YGR063C	YGR116W	0.7380
YGR063C	YIL021W	0.9021
YGR063C	YJL140W	0.7380

YGR063C	YML010W	1.0000
YGR063C	YNL201C	0.9551
YGR063C	YOL005C	0.8087
YGR063C	YOR151C	0.9021
YGR063C	YPR187W	0.7380
YGR071C	YPL128C	0.7380
YGR072W	YHR077C	0.9830
YGR072W	YMR080C	0.9099
YGR074W	YGR091W	0.8659
YGR074W	YHR086W	0.8087
YGR074W	YHR165C	0.9499
YGR074W	YJL203W	0.8659
YGR074W	YJR022W	0.7380
YGR074W	YKL012W	0.8659
YGR074W	YKL173W	0.9648
YGR074W	YLL036C	0.9186
YGR074W	YLR147C	0.9313
YGR074W	YLR275W	0.7380
YGR074W	YLR298C	0.8659
YGR074W	YML046W	0.8659
YGR074W	YMR125W	0.9313
YGR074W	YMR240C	0.7380
YGR074W	YOR159C	0.7380
YGR074W	YPL213W	0.9743
YGR074W	YPR057W	0.7380
YGR074W	YPR182W	0.7380
YGR076C	YGR220C	0.7380
YGR077C	YJL210W	0.8603
YGR077C	YLR191W	0.9910
YGR077C	YMR026C	0.8953
YGR078C	YJL179W	0.9313
YGR078C	YLR200W	0.9981
YGR078C	YML094W	0.9926
YGR078C	YNL153C	0.9962
YGR080W	YIL034C	0.9249
YGR080W	YNL138W	0.6147
YGR081C	YMR308C	0.9648
YGR081C	YPL012W	0.7380
YGR082W	YMR203W	1.0000
YGR082W	YNL121C	0.9930
YGR082W	YNL131W	1.0000
YGR083C	YJR007W	0.9970
YGR083C	YKR026C	0.9962
YGR083C	YLR291C	0.9999
YGR083C	YOR260W	1.0000
YGR083C	YPL237W	0.9598
YGR084C	YHL004W	0.9499

YGR084C	YMR188C	0.7380
YGR084C	YNL137C	0.7380
YGR084C	YOR158W	0.7380
YGR084C	YPL013C	0.7380
YGR085C	YKR081C	0.9883
YGR085C	YNL061W	0.7380
YGR085C	YOR294W	0.9996
YGR086C	YGR130C	0.8603
YGR086C	YKL142W	0.9788
YGR086C	YLR429W	0.7380
YGR086C	YMR031C	0.9285
YGR086C	YMR086W	0.9285
YGR086C	YOL100W	0.8151
YGR086C	YPL004C	0.9988
YGR086C	YPL074W	0.7380
YGR090W	YGR128C	0.9928
YGR090W	YGR145W	0.8659
YGR090W	YHR148W	0.9313
YGR090W	YHR196W	0.7380
YGR090W	YIL035C	0.9908
YGR090W	YIL069C	0.7380
YGR090W	YJL069C	0.9313
YGR090W	YJL109C	0.9820
YGR090W	YJR002W	0.9928
YGR090W	YJR123W	0.7380
YGR090W	YJR145C	0.8659
YGR090W	YKR060W	0.8659
YGR090W	YLR003C	0.7380
YGR090W	YLR129W	0.9313
YGR090W	YLR186W	0.9313
YGR090W	YLR223C	0.9794
YGR090W	YLR409C	0.9313
YGR090W	YML024W	0.7380
YGR090W	YML063W	0.7380
YGR090W	YMR116C	0.7380
YGR090W	YMR128W	0.9313
YGR090W	YMR229C	0.9313
YGR090W	YNL061W	0.7380
YGR090W	YNL132W	0.9313
YGR090W	YOR039W	0.9908
YGR090W	YOR061W	0.9313
YGR090W	YOR078W	0.7380
YGR090W	YOR096W	0.8659
YGR090W	YOR145C	0.7380
YGR090W	YOR206W	0.7380
YGR090W	YOR310C	0.7380
YGR090W	YOR312C	0.7380

YGR090W	YPL126W	0.9313
YGR090W	YPL266W	0.8659
YGR090W	YPR137W	0.7380
YGR090W	YPR144C	0.9820
YGR091W	YHR034C	0.8965
YGR091W	YHR165C	0.9648
YGR091W	YJL063C	0.7380
YGR091W	YKL173W	0.9313
YGR091W	YLR147C	0.7380
YGR091W	YLR275W	0.8659
YGR091W	YLR438C-A	0.8603
YGR091W	YLR439W	0.7380
YGR091W	YML025C	0.7380
YGR091W	YMR024W	0.7380
YGR091W	YNL005C	0.7380
YGR091W	YNL147W	0.7380
YGR091W	YOR308C	0.9313
YGR091W	YPR082C	0.8659
YGR091W	YPR178W	0.9820
YGR091W	YPR182W	0.7380
YGR092W	YIL106W	0.9999
YGR092W	YIL135C	0.6042
YGR092W	YJL076W	0.7974
YGR092W	YJL108C	0.6042
YGR092W	YKL168C	0.6042
YGR092W	YMR001C	0.8818
YGR092W	YMR165C	0.6042
YGR092W	YMR184W	0.6042
YGR092W	YMR229C	0.6042
YGR092W	YMR239C	0.6042
YGR092W	YNL101W	0.6042
YGR092W	YNL155W	0.6042
YGR092W	YNR006W	0.6042
YGR092W	YNR047W	0.6042
YGR092W	YNR052C	0.9099
YGR092W	YOR362C	0.6042
YGR092W	YPL042C	0.9099
YGR092W	YPL150W	0.6042
YGR092W	YPR091C	0.6042
YGR094W	YGR128C	0.8953
YGR095C	YGR158C	0.9920
YGR095C	YGR195W	1.0000
YGR095C	YHR069C	0.9989
YGR095C	YHR081W	0.9904
YGR095C	YNL189W	0.7380
YGR095C	YNL232W	0.9999
YGR095C	YNR024W	0.8087

YGR095C	YOL021C	1.0000
YGR095C	YOL142W	0.9989
YGR095C	YOR001W	0.9991
YGR095C	YOR076C	0.9951
YGR097W	YNL025C	0.9099
YGR098C	YOR195W	0.9215
YGR099W	YKL033W	0.8258
YGR100W	YLR262C	0.8655
YGR103W	YGR245C	0.9743
YGR103W	YHR052W	0.9859
YGR103W	YHR066W	0.9850
YGR103W	YHR088W	0.8087
YGR103W	YHR197W	0.9995
YGR103W	YIL018W	0.8087
YGR103W	YKL009W	0.9958
YGR103W	YKL021C	0.8087
YGR103W	YKL172W	0.8953
YGR103W	YKR081C	0.9902
YGR103W	YLL004W	0.9574
YGR103W	YLL008W	0.9726
YGR103W	YLL045C	0.8087
YGR103W	YLR002C	0.9266
YGR103W	YLR009W	0.9478
YGR103W	YLR074C	0.8659
YGR103W	YLR106C	0.8659
YGR103W	YLR347C	0.7380
YGR103W	YML065W	0.9960
YGR103W	YMR049C	1.0000
YGR103W	YMR229C	0.9313
YGR103W	YMR290C	0.9743
YGR103W	YNL002C	0.9499
YGR103W	YNL061W	0.7380
YGR103W	YNL110C	0.9869
YGR103W	YNL175C	0.7380
YGR103W	YNL189W	0.8659
YGR103W	YNR053C	0.9726
YGR103W	YOL077C	0.9860
YGR103W	YOR061W	0.7380
YGR103W	YOR063W	0.9961
YGR103W	YOR206W	0.8659
YGR103W	YOR272W	1.0000
YGR103W	YPL043W	0.9624
YGR103W	YPL093W	0.9998
YGR103W	YPL131W	0.9726
YGR103W	YPL211W	0.9464
YGR103W	YPR016C	0.9964
YGR104C	YHR041C	1.0000

YGR104C	YHR058C	1.0000
YGR104C	YIL021W	0.9875
YGR104C	YLR071C	1.0000
YGR104C	YLR418C	0.9215
YGR104C	YMR112C	0.9998
YGR104C	YNL025C	0.9834
YGR104C	YNL236W	0.9999
YGR104C	YNR010W	0.9971
YGR104C	YOL051W	1.0000
YGR104C	YOL135C	1.0000
YGR104C	YOR174W	0.9995
YGR104C	YPL042C	0.9999
YGR104C	YPR070W	0.9999
YGR104C	YPR086W	0.9555
YGR104C	YPR168W	0.9897
YGR105W	YGR106C	0.7187
YGR105W	YHR026W	0.8603
YGR105W	YLR447C	0.9705
YGR105W	YOR270C	0.9779
YGR105W	YPL234C	0.9609
YGR112W	YHR051W	0.8603
YGR112W	YIL157C	0.9991
YGR112W	YJL062W-A	0.9918
YGR112W	YLR203C	0.9969
YGR112W	YML129C	0.9969
YGR112W	YNL052W	0.8603
YGR113W	YGR140W	0.9079
YGR113W	YIL144W	0.9902
YGR113W	YKL049C	0.9079
YGR113W	YKL052C	0.9726
YGR113W	YKL138C-A	0.9710
YGR113W	YKR037C	1.0000
YGR113W	YKR083C	0.8353
YGR113W	YLR423C	0.5774
YGR113W	YPL209C	0.9690
YGR116W	YIL035C	0.7380
YGR116W	YML010W	0.9726
YGR116W	YML069W	0.8521
YGR116W	YOR061W	0.7380
YGR116W	YPR133C	0.9944
YGR119C	YGR218W	0.8353
YGR119C	YJL041W	1.0000
YGR119C	YLL024C	0.7380
YGR119C	YLR335W	0.6147
YGR119C	YLR347C	0.8566
YGR119C	YLR423C	0.5774
YGR119C	YMR308C	0.6672

YGR119C	YPL169C	0.8628
YGR120C	YLR026C	0.9799
YGR120C	YLR423C	0.5774
YGR120C	YML071C	0.9985
YGR120C	YNL041C	0.9988
YGR120C	YNL051W	0.9975
YGR120C	YNL086W	0.5774
YGR120C	YPR105C	1.0000
YGR122W	YLR025W	0.5774
YGR123C	YKL117W	0.7909
YGR123C	YMR186W	0.7380
YGR123C	YPL240C	0.9581
YGR128C	YGR185C	0.9910
YGR128C	YGR218W	0.8953
YGR128C	YHR196W	1.0000
YGR128C	YJL069C	0.8953
YGR128C	YJL109C	0.9994
YGR128C	YJR002W	0.9944
YGR128C	YKL205W	0.9790
YGR128C	YLR222C	0.9464
YGR128C	YLR293C	0.9790
YGR128C	YLR409C	0.8953
YGR128C	YMR047C	0.8953
YGR128C	YMR093W	0.9974
YGR128C	YMR129W	0.8953
YGR128C	YNL132W	0.8087
YGR128C	YOR078W	0.7380
YGR128C	YOR310C	0.8603
YGR128C	YPL126W	0.9999
YGR128C	YPR144C	0.9499
YGR129W	YLL036C	0.9994
YGR129W	YLR117C	0.9828
YGR129W	YMR213W	0.8353
YGR130C	YMR031C	0.9634
YGR130C	YPL004C	0.9033
YGR132C	YGR231C	0.9726
YGR132C	YJL183W	0.7380
YGR132C	YMR089C	0.8566
YGR132C	YPL050C	0.7380
YGR134W	YJR011C	0.8297
YGR134W	YNL288W	0.9989
YGR134W	YNR052C	0.9499
YGR134W	YPR072W	0.9099
YGR135W	YGR253C	0.7380
YGR135W	YHR200W	0.7380
YGR135W	YJL001W	0.7380
YGR135W	YLL039C	0.8087

YGR135W	YLR021W	0.9773
YGR135W	YML092C	0.9915
YGR135W	YMR314W	0.9157
YGR135W	YOL038W	0.9313
YGR135W	YOR157C	0.9266
YGR135W	YOR261C	0.9266
YGR135W	YOR362C	0.9909
YGR135W	YPL144W	0.9191
YGR135W	YPR103W	0.9891
YGR136W	YLL039C	0.8566
YGR136W	YLR144C	0.6672
YGR136W	YOR181W	0.9911
YGR140W	YJR089W	0.9998
YGR140W	YMR094W	0.9935
YGR140W	YMR168C	0.9282
YGR140W	YNL078W	0.7909
YGR140W	YNL307C	0.8456
YGR140W	YPL209C	0.7543
YGR140W	YPL268W	0.9099
YGR142W	YNL263C	0.9099
YGR144W	YNL189W	0.5774
YGR145W	YNL132W	0.8659
YGR145W	YNR054C	0.9021
YGR145W	YPR144C	0.8659
YGR148C	YKL009W	0.9215
YGR152C	YLR229C	0.9941
YGR155W	YJR045C	0.7380
YGR156W	YJL033W	0.7380
YGR156W	YJR093C	0.9743
YGR156W	YKL018W	0.9634
YGR156W	YKL059C	0.9813
YGR156W	YKR002W	0.9743
YGR156W	YLR115W	0.9743
YGR156W	YLR277C	0.9021
YGR156W	YMR061W	0.9457
YGR156W	YNL222W	0.9634
YGR156W	YNL317W	0.9904
YGR156W	YOR179C	0.9743
YGR156W	YPR107C	0.9743
YGR158C	YGR195W	0.9996
YGR158C	YHR069C	0.9861
YGR158C	YHR081W	0.7380
YGR158C	YJR022W	0.7253
YGR158C	YLR345W	0.7253
YGR158C	YNL232W	0.9996
YGR158C	YOL021C	0.9999
YGR158C	YOL142W	0.8659

YGR158C	YOR001W	0.9813
YGR158C	YOR076C	0.9499
YGR158C	YPR189W	0.7380
YGR159C	YJL010C	0.8087
YGR159C	YOR310C	0.7380
YGR161C	YJL098W	0.9648
YGR162W	YGR285C	0.7380
YGR162W	YHL034C	0.9313
YGR162W	YIL061C	0.7380
YGR162W	YIR001C	0.8659
YGR162W	YJL138C	1.0000
YGR162W	YJL190C	0.7380
YGR162W	YKR059W	1.0000
YGR162W	YLR175W	0.9313
YGR162W	YMR116C	0.7380
YGR162W	YMR125W	0.9820
YGR162W	YNL005C	0.7380
YGR162W	YNL262W	0.7380
YGR162W	YOL139C	1.0000
YGR162W	YOR204W	0.7380
YGR162W	YOR243C	0.7380
YGR163W	YGR203W	0.8566
YGR163W	YKR007W	0.9962
YGR163W	YML121W	1.0000
YGR163W	YPL235W	0.8566
YGR165W	YHL004W	0.8087
YGR165W	YIL070C	0.7380
YGR165W	YIL093C	0.7380
YGR165W	YJR113C	0.7380
YGR165W	YNL137C	0.8659
YGR165W	YPL013C	0.7380
YGR165W	YPL118W	0.7380
YGR166W	YKR068C	0.9997
YGR166W	YML077W	0.8566
YGR166W	YMR218C	0.9852
YGR166W	YOR115C	0.9884
YGR167W	YJR125C	0.8953
YGR167W	YNL243W	0.9975
YGR170W	YNL264C	0.8566
YGR172C	YHR105W	0.9138
YGR172C	YNL263C	0.9998
YGR172C	YOR132W	0.9138
YGR172C	YPR028W	0.9710
YGR175C	YHR020W	0.7380
YGR175C	YLL039C	0.9726
YGR175C	YMR015C	0.9151
YGR175C	YMR202W	0.9151

YGR175C	YNL280C	0.9151
YGR178C	YHR121W	0.9896
YGR178C	YNL157W	0.8566
YGR179C	YIR010W	0.8659
YGR179C	YJR135C	0.9624
YGR179C	YKL049C	0.9037
YGR179C	YLR315W	0.8893
YGR179C	YLR381W	0.9313
YGR179C	YPL018W	0.9990
YGR179C	YPL233W	0.9068
YGR179C	YPR046W	0.9464
YGR180C	YJL026W	1.0000
YGR180C	YLR437C	0.9282
YGR180C	YOR229W	0.9710
YGR180C	YOR230W	0.9993
YGR181W	YJR135W-A	0.9937
YGR181W	YKL195W	0.9799
YGR181W	YNR017W	0.9901
YGR184C	YPL177C	0.9985
YGR185C	YGR229C	0.9976
YGR186W	YIL021W	0.9963
YGR186W	YML010W	0.8659
YGR186W	YMR277W	0.9282
YGR186W	YOL005C	0.9021
YGR186W	YOR151C	0.9499
YGR186W	YOR210W	0.7380
YGR186W	YPL129W	1.0000
YGR186W	YPR187W	0.7380
YGR188C	YIR010W	0.6672
YGR188C	YOR026W	1.0000
YGR191W	YPL218W	0.9555
YGR192C	YHR200W	0.8087
YGR192C	YIR001C	0.7380
YGR195W	YHR069C	0.9999
YGR195W	YJL109C	0.7380
YGR195W	YMR128W	0.7380
YGR195W	YNL189W	0.7380
YGR195W	YNL232W	1.0000
YGR195W	YOL021C	1.0000
YGR195W	YOL142W	0.9992
YGR195W	YOR001W	0.9995
YGR195W	YOR076C	0.9986
YGR195W	YPR072W	0.9773
YGR198W	YLR305C	0.9937
YGR198W	YMR212C	0.7983
YGR200C	YHR187W	1.0000
YGR200C	YIL021W	0.9555

YGR200C	YJL140W	0.9555
YGR200C	YKL110C	0.9887
YGR200C	YLR384C	1.0000
YGR200C	YMR312W	0.9952
YGR200C	YOR151C	0.9555
YGR200C	YPL086C	1.0000
YGR200C	YPL101W	0.9998
YGR202C	YNL189W	0.9157
YGR206W	YLR119W	0.7380
YGR209C	YLR109W	0.8798
YGR209C	YML028W	0.8798
YGR209C	YPR167C	0.8798
YGR215W	YHL004W	0.9499
YGR215W	YKL155C	0.9266
YGR215W	YMR188C	0.7380
YGR215W	YOR158W	0.7380
YGR218W	YGR262C	0.8353
YGR218W	YHR170W	0.9215
YGR218W	YIL063C	0.9954
YGR218W	YIL115C	0.9966
YGR218W	YJR077C	0.7380
YGR218W	YJR134C	0.8965
YGR218W	YKL068W	0.8087
YGR218W	YKL143W	0.9281
YGR218W	YKL145W	0.7380
YGR218W	YLL039C	0.7380
YGR218W	YLR131C	0.9992
YGR218W	YLR180W	0.7380
YGR218W	YLR293C	0.9999
YGR218W	YML007W	0.9939
YGR218W	YMR047C	0.6672
YGR218W	YMR124W	0.7909
YGR218W	YMR180C	0.9138
YGR218W	YMR235C	0.8087
YGR218W	YOL123W	0.8894
YGR220C	YJL063C	0.8659
YGR220C	YKL167C	0.8659
YGR220C	YKR006C	0.8659
YGR220C	YKR085C	0.8659
YGR220C	YLR189C	0.7380
YGR220C	YLR312W-A	0.7380
YGR220C	YLR371W	0.7380
YGR220C	YLR439W	0.8659
YGR220C	YML009C	0.7380
YGR220C	YML025C	0.9313
YGR220C	YMR193W	0.9313
YGR220C	YMR225C	0.7380

YGR220C	YNL005C	0.8659
YGR220C	YNL252C	0.7380
YGR220C	YPL183W-A	0.7380
YGR229C	YHR030C	0.9970
YGR229C	YOR028C	0.8965
YGR231C	YJL183W	0.7380
YGR231C	YLR342W	0.7380
YGR231C	YPL050C	0.7380
YGR232W	YHR027C	0.9860
YGR232W	YHR200W	0.9980
YGR232W	YIL007C	0.9215
YGR232W	YIL075C	0.9860
YGR232W	YKL145W	1.0000
YGR232W	YLR421C	0.9464
YGR232W	YOR117W	0.9961
YGR232W	YOR259C	0.9464
YGR232W	YOR261C	0.9961
YGR232W	YPR108W	0.9464
YGR233C	YIL050W	0.9747
YGR233C	YJL005W	0.9313
YGR233C	YNL138W	0.7380
YGR233C	YOL001W	1.0000
YGR233C	YPL031C	1.0000
YGR234W	YPL106C	0.7380
YGR238C	YHR158C	0.9884
YGR238C	YLR096W	0.8028
YGR239C	YLR191W	0.7909
YGR239C	YMR267W	0.5774
YGR240C	YLR180W	0.7380
YGR240C	YLR212C	0.7380
YGR240C	YMR205C	0.9959
YGR241C	YIL095W	0.6672
YGR241C	YIR006C	0.8965
YGR241C	YNL020C	0.6672
YGR241C	YNL084C	0.7909
YGR245C	YHR197W	0.9986
YGR245C	YKL009W	0.8566
YGR245C	YKR048C	0.9968
YGR245C	YKR081C	0.7380
YGR245C	YLR074C	0.9313
YGR245C	YLR106C	0.9313
YGR245C	YNL002C	0.8659
YGR245C	YNL110C	0.9313
YGR245C	YNL182C	0.9951
YGR245C	YNR053C	0.9313
YGR245C	YOR063W	0.7380
YGR245C	YPL093W	0.9869

YGR245C	YPL131W	0.8566
YGR245C	YPR016C	0.9648
YGR246C	YJL011C	0.9723
YGR246C	YNL039W	0.9948
YGR246C	YNR003C	0.8965
YGR249W	YPL203W	0.7347
YGR250C	YIR001C	0.9888
YGR252W	YHR079C	0.9758
YGR252W	YHR099W	0.9999
YGR252W	YIL149C	0.9249
YGR252W	YKR095W	0.9249
YGR252W	YLR055C	0.9962
YGR252W	YMR223W	0.9999
YGR252W	YMR236W	0.9944
YGR252W	YNL030W	0.9827
YGR252W	YNL031C	0.9981
YGR252W	YOL012C	0.8655
YGR252W	YOL148C	0.9902
YGR252W	YOR023C	0.9888
YGR252W	YPL047W	0.9973
YGR252W	YPL082C	0.8953
YGR252W	YPL254W	0.9998
YGR253C	YJL001W	0.7380
YGR253C	YLR021W	0.9754
YGR253C	YML092C	0.8659
YGR253C	YMR314W	0.7380
YGR253C	YOL038W	0.7380
YGR253C	YOR157C	0.7380
YGR253C	YOR362C	0.9822
YGR253C	YPL144W	0.9934
YGR253C	YPR103W	0.9787
YGR254W	YHR200W	0.8087
YGR254W	YJR045C	0.7380
YGR254W	YLL039C	0.7380
YGR258C	YIL143C	0.9574
YGR258C	YLR430W	0.9099
YGR258C	YOL090W	0.9099
YGR261C	YHR174W	0.7380
YGR261C	YJL024C	0.7380
YGR261C	YOR106W	0.9555
YGR261C	YPL195W	0.9962
YGR262C	YHR205W	0.8655
YGR262C	YHR216W	0.8353
YGR262C	YKR038C	1.0000
YGR262C	YML036W	0.9993
YGR262C	YML056C	0.8353
YGR267C	YNL189W	0.5774

YGR268C	YHL002W	0.9249
YGR268C	YHR016C	0.7253
YGR268C	YOR124C	0.8816
YGR268C	YOR138C	0.8179
YGR270W	YIL126W	0.8087
YGR270W	YJL167W	0.9071
YGR270W	YLR455W	0.8087
YGR270W	YNL031C	0.9107
YGR270W	YNL088W	0.8087
YGR270W	YOL012C	0.7380
YGR270W	YPL153C	0.9021
YGR274C	YHR023W	0.7380
YGR274C	YKR001C	0.7380
YGR274C	YML015C	1.0000
YGR274C	YML098W	1.0000
YGR274C	YML114C	1.0000
YGR274C	YMR005W	1.0000
YGR274C	YMR227C	1.0000
YGR274C	YMR236W	1.0000
YGR274C	YNL236W	0.7380
YGR274C	YPL011C	1.0000
YGR274C	YPL129W	1.0000
YGR274C	YPR086W	0.8240
YGR275W	YHL025W	0.9976
YGR275W	YIL126W	0.9820
YGR275W	YJL176C	0.9624
YGR275W	YKR008W	0.9313
YGR275W	YLR033W	0.9908
YGR275W	YLR321C	0.9313
YGR275W	YLR357W	0.9648
YGR275W	YML127W	0.8659
YGR275W	YMR033W	0.9952
YGR275W	YMR091C	0.9648
YGR275W	YOR290C	0.9313
YGR275W	YPL016W	0.7380
YGR275W	YPL129W	0.7380
YGR275W	YPR034W	0.9906
YGR278W	YHR165C	0.9313
YGR278W	YKL173W	0.8659
YGR278W	YLL036C	0.8659
YGR278W	YLR117C	0.8659
YGR278W	YMR213W	0.9648
YGR278W	YMR288W	0.7380
YGR278W	YPL151C	0.7380
YGR283C	YHR089C	0.8659
YGR283C	YMR310C	0.8659
YGR284C	YHR110W	0.4335

YGR284C	YML067C	0.4335
YGR285C	YHR064C	1.0000
YGR285C	YJL080C	0.8659
YGR285C	YLR406C	0.8953
YGR285C	YMR116C	0.7380
YGR285C	YPR189W	0.8659
YHL002W	YIL156W	0.9911
YHL002W	YKL213C	0.8816
YHL002W	YNR006W	1.0000
YHL002W	YOR076C	0.5774
YHL003C	YKL008C	0.9711
YHL003C	YMR298W	0.9668
YHL004W	YIL093C	0.9499
YHL004W	YJR101W	0.9499
YHL004W	YJR113C	0.9499
YHL004W	YKL155C	0.9860
YHL004W	YLR423C	0.5774
YHL004W	YMR158W	0.9499
YHL004W	YMR188C	0.9499
YHL004W	YNL137C	0.9743
YHL004W	YNL306W	0.9499
YHL004W	YNR037C	0.9499
YHL004W	YOR158W	0.9743
YHL004W	YOR204W	0.7380
YHL004W	YOR243C	0.7380
YHL004W	YPL013C	0.7380
YHL004W	YPL118W	0.8659
YHL007C	YIL043C	0.9151
YHL007C	YLR229C	1.0000
YHL007C	YLR362W	0.8894
YHL007C	YNL161W	0.8353
YHL007C	YOR212W	0.9790
YHL007C	YPL256C	0.9464
YHL010C	YLL039C	0.9598
YHL011C	YKL181W	0.9644
YHL011C	YMR139W	0.8353
YHL015W	YLL039C	0.9266
YHL018W	YNL113W	0.5774
YHL019C	YKL135C	0.9958
YHL019C	YLR170C	0.9624
YHL019C	YPR029C	0.8659
YHL020C	YOL004W	0.8965
YHL022C	YHR157W	0.9667
YHL022C	YLR329W	0.9929
YHL025W	YJL176C	1.0000
YHL025W	YMR033W	0.9999
YHL025W	YNR023W	1.0000

YHL025W	YOR290C	1.0000
YHL025W	YPL016W	1.0000
YHL025W	YPL129W	1.0000
YHL025W	YPR034W	0.9999
YHL027W	YMR032W	0.5774
YHL029C	YNL032W	0.8659
YHL029C	YNL099C	0.7380
YHL030W	YHR027C	0.8659
YHL030W	YHR200W	0.9313
YHL030W	YIL075C	0.7380
YHL030W	YKL145W	0.9313
YHL030W	YLR421C	0.8659
YHL030W	YML092C	0.7380
YHL030W	YOL038W	0.9021
YHL030W	YOR117W	0.7380
YHL030W	YOR259C	0.7380
YHL030W	YOR261C	0.9313
YHL030W	YPR108W	0.7380
YHL031C	YKL006C-A	0.9799
YHL031C	YKL196C	0.9799
YHL031C	YLR026C	0.9999
YHL031C	YLR078C	0.9282
YHL033C	YHR170W	0.9780
YHL033C	YKL009W	0.9191
YHL033C	YKR002W	0.7380
YHL033C	YLL039C	0.7380
YHL033C	YLR075W	0.8566
YHL034C	YJL190C	0.7380
YHL034C	YJR145C	0.9021
YHL034C	YLL045C	0.8087
YHL034C	YLR175W	0.7380
YHL034C	YLR432W	0.8087
YHL034C	YMR125W	0.7380
YHL034C	YOL077C	0.7380
YHL034C	YOL139C	0.9313
YHL034C	YOR312C	0.8087
YHL034C	YPL198W	0.8087
YHL034C	YPR080W	0.9021
YHR001W	YPR173C	0.9138
YHR005C	YJR086W	0.9935
YHR005C	YLR240W	0.9249
YHR005C	YLR452C	0.9996
YHR005C	YOR212W	1.0000
YHR005C	YPR141C	0.9707
YHR005C-A	YKL195W	0.9795
YHR007C	YHR072W	0.9928
YHR007C	YHR190W	0.9151

YHR007C	YJR117W	0.4335
YHR007C	YKL008C	0.4335
YHR007C	YLL039C	0.7380
YHR007C	YLR056W	0.9151
YHR007C	YLR100W	0.9928
YHR007C	YML008C	0.9742
YHR007C	YNL280C	0.9151
YHR010W	YHR052W	0.8659
YHR010W	YNL110C	0.7380
YHR010W	YPL093W	0.7380
YHR010W	YPR189W	0.7380
YHR012W	YJL053W	0.9942
YHR012W	YJL154C	1.0000
YHR012W	YOR069W	0.9915
YHR012W	YOR132W	0.9447
YHR013C	YOR253W	0.9266
YHR014W	YMR001C	0.8953
YHR016C	YJR083C	0.8591
YHR016C	YLR144C	0.8591
YHR016C	YMR192W	0.8591
YHR016C	YNL094W	0.5774
YHR016C	YOR042W	0.5107
YHR016C	YOR181W	0.9999
YHR016C	YOR284W	0.5774
YHR016C	YPR171W	0.8591
YHR019C	YHR200W	0.7380
YHR020W	YLR180W	0.7380
YHR023W	YKL179C	0.6000
YHR023W	YNL272C	0.6000
YHR023W	YOR326W	0.8659
YHR023W	YPR188C	0.9921
YHR024C	YLR163C	0.9944
YHR025W	YNL189W	0.5774
YHR027C	YHR169W	0.7380
YHR027C	YHR200W	1.0000
YHR027C	YIL075C	1.0000
YHR027C	YKL145W	1.0000
YHR027C	YKL152C	0.7380
YHR027C	YLL039C	0.7380
YHR027C	YLR153C	0.7380
YHR027C	YLR180W	0.7380
YHR027C	YLR421C	0.9313
YHR027C	YMR091C	0.7380
YHR027C	YNL055C	0.7380
YHR027C	YOR117W	0.9993
YHR027C	YOR259C	0.9991
YHR027C	YOR261C	0.9971

YHR027C	YOR362C	0.9555
YHR027C	YPR108W	0.9969
YHR030C	YHR084W	0.8566
YHR030C	YHR102W	0.9266
YHR030C	YIL113W	0.9714
YHR030C	YJL095W	0.9287
YHR030C	YLL021W	0.8894
YHR030C	YLR096W	0.7380
YHR030C	YLR182W	0.9640
YHR030C	YLR187W	0.7380
YHR030C	YLR371W	0.7543
YHR030C	YLR442C	0.9382
YHR030C	YNL053W	0.9998
YHR030C	YNR031C	0.8566
YHR030C	YOR208W	0.9382
YHR030C	YOR231W	0.9833
YHR030C	YPL089C	0.9809
YHR030C	YPL140C	0.9964
YHR030C	YPL240C	0.9592
YHR030C	YPR054W	0.7380
YHR034C	YJR022W	0.5774
YHR034C	YLR323C	0.9710
YHR034C	YOR310C	0.9907
YHR034C	YPL146C	0.9723
YHR034C	YPL235W	0.9764
YHR035W	YJR022W	0.5774
YHR039C-A	YKL080W	0.8603
YHR039C-A	YOR270C	0.9215
YHR039C-A	YOR332W	0.9992
YHR039C-A	YPR036W	0.9634
YHR041C	YHR058C	0.9964
YHR041C	YLR071C	0.9988
YHR041C	YML007W	0.7380
YHR041C	YMR112C	0.7380
YHR041C	YNL236W	0.7380
YHR041C	YNR010W	0.9266
YHR041C	YOL051W	0.9940
YHR041C	YOL135C	0.9787
YHR041C	YOR174W	0.7380
YHR041C	YPR070W	0.7380
YHR041C	YPR168W	0.7380
YHR042W	YNL289W	0.9884
YHR047C	YLR148W	0.7380
YHR052W	YHR066W	0.7380
YHR052W	YHR085W	0.7380
YHR052W	YHR088W	0.8659
YHR052W	YHR197W	0.9808

YHR052W	YKL009W	0.9970
YHR052W	YKL021C	0.8087
YHR052W	YKL145W	0.9191
YHR052W	YKL172W	0.9313
YHR052W	YKR081C	0.9648
YHR052W	YLL008W	0.8659
YHR052W	YLL034C	0.7380
YHR052W	YLL045C	0.7380
YHR052W	YLR002C	0.7380
YHR052W	YLR009W	0.8087
YHR052W	YLR074C	0.8659
YHR052W	YLR106C	0.8659
YHR052W	YML065W	0.8087
YHR052W	YMR049C	0.9648
YHR052W	YMR229C	0.9648
YHR052W	YMR242C	0.7380
YHR052W	YMR290C	0.9313
YHR052W	YNL002C	0.7380
YHR052W	YNL061W	0.9648
YHR052W	YNL110C	0.9931
YHR052W	YNL112W	0.6147
YHR052W	YNL182C	0.7380
YHR052W	YOL038W	0.9191
YHR052W	YOL041C	0.8659
YHR052W	YOL077C	0.9313
YHR052W	YOL127W	0.7380
YHR052W	YOR063W	0.8659
YHR052W	YOR206W	0.9648
YHR052W	YOR272W	0.9869
YHR052W	YOR310C	0.7380
YHR052W	YPL012W	0.7380
YHR052W	YPL043W	0.9648
YHR052W	YPL093W	0.9499
YHR052W	YPL131W	0.9808
YHR052W	YPL211W	0.8659
YHR052W	YPR016C	0.9021
YHR056C	YIL126W	0.8087
YHR056C	YLR033W	0.7380
YHR056C	YLR357W	0.9794
YHR056C	YMR091C	0.9266
YHR058C	YLR071C	0.9994
YHR058C	YML007W	0.7380
YHR058C	YNR010W	0.9887
YHR058C	YOL051W	0.9583
YHR058C	YOL135C	0.9991
YHR058C	YOR174W	0.9883
YHR058C	YPR070W	0.8659

YHR058C	YPR168W	0.9157
YHR060W	YLR447C	0.8214
YHR061C	YHR107C	0.9440
YHR061C	YJR053W	0.9138
YHR061C	YJR076C	0.9099
YHR061C	YLR229C	1.0000
YHR061C	YMR055C	0.9636
YHR062C	YLR145W	0.9794
YHR062C	YNL221C	0.9975
YHR062C	YNL282W	0.9946
YHR064C	YHR069C	0.7380
YHR064C	YIL002C	0.7380
YHR064C	YKL116C	0.7380
YHR064C	YKR038C	0.7380
YHR064C	YKR101W	0.7380
YHR064C	YMR121C	0.7380
YHR064C	YNL209W	0.9313
YHR064C	YOR091W	0.7380
YHR064C	YPL110C	0.7380
YHR064C	YPL240C	0.8659
YHR064C	YPR025C	0.7380
YHR066W	YHR088W	0.8087
YHR066W	YKL014C	0.7380
YHR066W	YKL082C	0.8087
YHR066W	YKL172W	0.8087
YHR066W	YKR081C	0.9021
YHR066W	YLL008W	0.8087
YHR066W	YLR276C	0.8087
YHR066W	YMR049C	0.8087
YHR066W	YMR290C	0.8087
YHR066W	YNL002C	0.8087
YHR066W	YNL061W	0.8087
YHR066W	YOR206W	0.7380
YHR066W	YOR272W	0.8953
YHR066W	YPL043W	0.8087
YHR066W	YPL093W	0.9988
YHR066W	YPL211W	0.8087
YHR066W	YPR016C	0.9850
YHR066W	YPR143W	0.6672
YHR069C	YHR081W	0.9928
YHR069C	YNL189W	0.7380
YHR069C	YNL232W	0.9973
YHR069C	YNR024W	0.9021
YHR069C	YOL021C	1.0000
YHR069C	YOL142W	0.9313
YHR069C	YOR001W	0.9999
YHR069C	YOR076C	0.9860

YHR071W	YPL031C	0.9884
YHR072W	YLR056W	0.9151
YHR072W	YLR100W	0.9996
YHR072W	YML008C	0.9151
YHR072W	YMR015C	0.9151
YHR072W-A	YHR089C	0.7891
YHR072W-A	YLR175W	0.9616
YHR072W-A	YNL124W	0.9809
YHR074W	YLR216C	0.7380
YHR076W	YMR079W	0.8297
YHR077C	YMR080C	1.0000
YHR077C	YOL123W	0.9215
YHR079C	YJL034W	0.9938
YHR079C	YLR361C	0.8655
YHR079C-A	YNL312W	0.9282
YHR079C-A	YPL121C	0.9799
YHR081W	YNL232W	0.9499
YHR081W	YNR024W	0.8087
YHR081W	YOL021C	0.9963
YHR081W	YOL142W	0.7380
YHR081W	YOR001W	0.9995
YHR082C	YHR186C	0.7380
YHR082C	YJR066W	0.8566
YHR083W	YMR060C	0.9780
YHR083W	YMR203W	0.9249
YHR083W	YNL026W	0.9780
YHR084W	YMR043W	0.9282
YHR084W	YMR308C	0.9191
YHR084W	YPL049C	1.0000
YHR085W	YHR197W	1.0000
YHR085W	YLR074C	0.7380
YHR085W	YLR106C	0.9021
YHR085W	YNL182C	0.9648
YHR085W	YNR053C	0.7380
YHR085W	YPL093W	0.9021
YHR086W	YIL061C	0.9963
YHR086W	YKL012W	0.9869
YHR086W	YKL173W	0.7380
YHR086W	YLR147C	0.9499
YHR086W	YLR275W	0.9743
YHR086W	YLR298C	0.9648
YHR086W	YML046W	0.9743
YHR086W	YMR125W	0.8659
YHR086W	YPL178W	0.9648
YHR088W	YLL045C	0.7380
YHR088W	YMR290C	0.8659
YHR088W	YNL061W	0.8659

YHR088W	YNL110C	0.9313
YHR088W	YOL077C	0.7380
YHR088W	YOR206W	0.7380
YHR088W	YOR272W	0.8659
YHR088W	YPL093W	0.8087
YHR088W	YPL125W	0.9138
YHR088W	YPL131W	0.7380
YHR088W	YPR016C	0.9499
YHR089C	YLR175W	0.9986
YHR089C	YMR239C	0.9138
YHR089C	YMR310C	0.7380
YHR089C	YNL124W	0.9707
YHR089C	YNL308C	0.7380
YHR090C	YHR099W	0.9871
YHR090C	YJL081C	0.9726
YHR090C	YNL031C	0.9985
YHR090C	YNL107W	0.9971
YHR090C	YNL136W	0.9313
YHR090C	YOR244W	0.9995
YHR090C	YPR023C	0.9266
YHR098C	YPR181C	0.9956
YHR099W	YJL081C	0.9860
YHR099W	YJL098W	0.7380
YHR099W	YJR082C	0.7380
YHR099W	YLR055C	0.9499
YHR099W	YMR223W	0.9464
YHR099W	YMR236W	0.9969
YHR099W	YNL107W	0.9998
YHR099W	YNL136W	0.8659
YHR099W	YOL148C	0.9313
YHR099W	YOR244W	1.0000
YHR099W	YPL047W	0.9285
YHR099W	YPL082C	0.8087
YHR099W	YPL248C	0.9768
YHR099W	YPL254W	0.9989
YHR099W	YPR023C	0.8659
YHR102W	YKL189W	0.9853
YHR102W	YOR257W	0.9099
YHR102W	YOR353C	0.9710
YHR105W	YNL263C	0.9440
YHR105W	YOR034C	0.7909
YHR105W	YPL246C	0.7909
YHR107C	YJR076C	1.0000
YHR107C	YKL101W	0.9099
YHR107C	YLR314C	1.0000
YHR107C	YMR117C	0.5774
YHR107C	YNL166C	0.9138

YHR107C	YNL298W	0.6880
YHR107C	YPL153C	0.8087
YHR108W	YJR125C	0.9976
YHR108W	YKR094C	0.8965
YHR108W	YLL039C	0.9958
YHR108W	YLR167W	0.8965
YHR110W	YML012W	0.6147
YHR111W	YIL008W	0.9994
YHR111W	YNL119W	0.8603
YHR113W	YOL082W	0.5774
YHR114W	YIL156W	0.5774
YHR114W	YMR109W	0.6819
YHR114W	YNL094W	0.7253
YHR114W	YOR181W	1.0000
YHR115C	YLR215C	0.9619
YHR115C	YNL311C	0.7380
YHR115C	YPL031C	0.6042
YHR118C	YJR046W	0.9758
YHR118C	YLL004W	0.9999
YHR118C	YML065W	1.0000
YHR118C	YNL261W	0.9984
YHR118C	YPL001W	0.9285
YHR118C	YPR120C	0.9471
YHR118C	YPR162C	0.9813
YHR119W	YKL018W	1.0000
YHR119W	YLR015W	1.0000
YHR119W	YLR288C	0.9747
YHR119W	YNL031C	0.7696
YHR119W	YPL138C	1.0000
YHR121W	YNL157W	0.8566
YHR121W	YPL240C	0.7380
YHR122W	YIL128W	0.8718
YHR124W	YJL106W	0.9469
YHR128W	YNR012W	0.7837
YHR129C	YLL049W	0.9927
YHR129C	YMR294W	0.9978
YHR129C	YPL174C	0.9974
YHR133C	YLR450W	0.9665
YHR135C	YNL154C	0.7380
YHR135C	YOR177C	0.5779
YHR135C	YPR115W	0.5779
YHR143W-A	YIL021W	0.9982
YHR143W-A	YOR207C	0.8603
YHR143W-A	YOR224C	0.9285
YHR143W-A	YOR340C	0.9266
YHR143W-A	YOR341W	0.9464
YHR143W-A	YPR010C	0.9729

YHR144C	YJL090C	0.9138
YHR144C	YMR201C	0.9099
YHR146W	YPL154C	0.7380
YHR147C	YNL284C	0.7380
YHR148W	YHR196W	0.7380
YHR148W	YJL109C	0.8659
YHR148W	YJR002W	0.9539
YHR148W	YNL075W	0.8566
YHR148W	YOR078W	0.7380
YHR148W	YPL126W	0.8659
YHR152W	YMR308C	0.9616
YHR154W	YJL047C	0.9598
YHR154W	YJL090C	0.9598
YHR154W	YLR135W	0.9987
YHR154W	YLR320W	0.9764
YHR154W	YLR383W	0.8566
YHR154W	YPR164W	0.8566
YHR156C	YHR165C	0.9779
YHR157W	YLR329W	0.9747
YHR158C	YJL187C	0.9266
YHR158C	YLR096W	0.8028
YHR158C	YLR452C	0.7909
YHR158C	YOR269W	0.5774
YHR160C	YIL160C	0.9997
YHR160C	YLR191W	0.9857
YHR161C	YIL095W	0.8403
YHR161C	YIR006C	0.9710
YHR164C	YNL312W	0.7380
YHR165C	YIL061C	0.9934
YHR165C	YJL203W	0.7380
YHR165C	YJR022W	0.8603
YHR165C	YKL012W	0.8965
YHR165C	YKL173W	1.0000
YHR165C	YKR022C	0.9266
YHR165C	YLL036C	0.9891
YHR165C	YLR117C	0.9743
YHR165C	YLR147C	0.9743
YHR165C	YLR275W	0.9021
YHR165C	YLR424W	0.9869
YHR165C	YLR438C-A	0.8603
YHR165C	YML046W	0.8353
YHR165C	YML049C	0.8659
YHR165C	YMR125W	0.8659
YHR165C	YMR213W	0.9648
YHR165C	YMR240C	0.7380
YHR165C	YNL147W	0.7380
YHR165C	YOR159C	0.7380

YHR165C	YOR308C	0.9313
YHR165C	YPL151C	0.7380
YHR165C	YPL213W	0.9021
YHR165C	YPR101W	0.7380
YHR165C	YPR178W	0.9743
YHR165C	YPR182W	0.7380
YHR166C	YIR025W	0.9478
YHR166C	YKL022C	1.0000
YHR166C	YLR102C	0.8087
YHR166C	YLR127C	1.0000
YHR166C	YLR451W	0.5774
YHR166C	YNL172W	0.9923
YHR166C	YOR249C	0.9813
YHR166C	YPR119W	0.9138
YHR167W	YML062C	0.9991
YHR167W	YMR125W	0.7380
YHR167W	YNL004W	0.9743
YHR167W	YNL139C	0.9999
YHR167W	YNL253W	0.9624
YHR168W	YPL106C	0.7380
YHR169W	YNR054C	0.9758
YHR170W	YIR026C	0.8566
YHR170W	YLL045C	0.8566
YHR170W	YLR075W	0.9215
YHR170W	YLR340W	0.8566
YHR170W	YMR080C	0.8965
YHR171W	YLL042C	0.7909
YHR171W	YNR007C	0.9993
YHR172W	YLR212C	1.0000
YHR172W	YNL126W	1.0000
YHR185C	YIL007C	0.7253
YHR185C	YPL204W	0.8973
YHR186C	YJR066W	1.0000
YHR186C	YLR403W	0.9215
YHR186C	YNL006W	0.9609
YHR186C	YPL180W	0.8087
YHR187W	YKL110C	1.0000
YHR187W	YLR384C	1.0000
YHR187W	YMR312W	0.9577
YHR187W	YPL086C	1.0000
YHR187W	YPL101W	0.9813
YHR190W	YLR056W	0.9151
YHR190W	YLR100W	0.9762
YHR190W	YML008C	0.9151
YHR191C	YMR078C	0.9899
YHR193C	YPL037C	0.9999
YHR196W	YJL109C	0.9976

YHR196W	YJR002W	0.9860
YHR196W	YJR123W	0.7380
YHR196W	YJR145C	0.7380
YHR196W	YLL011W	0.8659
YHR196W	YLR129W	0.8659
YHR196W	YLR175W	0.7380
YHR196W	YLR186W	0.8659
YHR196W	YLR197W	0.8659
YHR196W	YLR441C	0.7380
YHR196W	YMR093W	0.9978
YHR196W	YMR229C	0.7380
YHR196W	YNL132W	0.7380
YHR196W	YOR096W	0.7380
YHR196W	YOR310C	0.9021
YHR196W	YPL012W	0.7380
YHR196W	YPL126W	0.9987
YHR196W	YPL217C	0.8659
YHR196W	YPL266W	0.7380
YHR196W	YPR137W	0.8659
YHR196W	YPR144C	0.8659
YHR197W	YJL122W	0.8566
YHR197W	YKL009W	0.9598
YHR197W	YKR081C	0.8659
YHR197W	YLR002C	0.7380
YHR197W	YLR074C	0.8659
YHR197W	YLR106C	0.9988
YHR197W	YLR423C	0.5774
YHR197W	YMR049C	0.7380
YHR197W	YMR290C	0.8659
YHR197W	YNL002C	0.8566
YHR197W	YNL061W	0.7380
YHR197W	YNL110C	0.9808
YHR197W	YNL182C	1.0000
YHR197W	YNR053C	0.9961
YHR197W	YOL077C	0.7380
YHR197W	YOL127W	0.8566
YHR197W	YOR063W	0.9598
YHR197W	YOR206W	0.7380
YHR197W	YOR272W	0.8462
YHR197W	YPL093W	0.9998
YHR197W	YPL131W	0.9780
YHR197W	YPL146C	0.8087
YHR197W	YPL211W	0.7380
YHR197W	YPR016C	0.9946
YHR199C-A	YJR089W	0.9099
YHR199C-A	YPL209C	0.9099
YHR200W	YIL075C	0.9743

YHR200W	YJR009C	0.8087
YHR200W	YJR123W	0.8087
YHR200W	YKL104C	0.7380
YHR200W	YKL145W	0.9999
YHR200W	YKL152C	0.8087
YHR200W	YLL024C	0.8087
YHR200W	YLL039C	0.9898
YHR200W	YLR044C	0.8087
YHR200W	YLR293C	0.8087
YHR200W	YLR340W	0.8087
YHR200W	YLR421C	0.9813
YHR200W	YML092C	0.9266
YHR200W	YMR276W	0.9993
YHR200W	YMR314W	0.9499
YHR200W	YNL067W	0.8087
YHR200W	YNL103W	0.9249
YHR200W	YNL209W	0.8087
YHR200W	YOL038W	0.7380
YHR200W	YOL040C	0.8087
YHR200W	YOR096W	0.8087
YHR200W	YOR117W	0.9921
YHR200W	YOR259C	0.9743
YHR200W	YOR261C	0.9933
YHR200W	YOR303W	0.8566
YHR200W	YPL031C	0.5779
YHR200W	YPR103W	0.7380
YHR200W	YPR108W	0.9981
YHR203C	YLL039C	0.9266
YHR203C	YOR310C	0.7380
YHR205W	YJR066W	0.7696
YHR205W	YNL167C	0.8655
YHR206W	YIL147C	0.8894
YHR208W	YJR148W	0.9313
YHR216W	YLR432W	0.9313
YHR216W	YML056C	0.8659
YHR216W	YOR250C	0.7380
YIL004C	YIL109C	0.9995
YIL004C	YKL196C	0.9986
YIL004C	YLR026C	1.0000
YIL004C	YLR078C	0.9999
YIL004C	YLR268W	0.9991
YIL004C	YPL218W	0.9941
YIL004C	YPR181C	0.9995
YIL007C	YOR117W	0.9981
YIL007C	YOR259C	0.9747
YIL008W	YNL119W	0.8798
YIL017C	YIL097W	0.7380

YIL017C	YMR135C	0.9021
YIL019W	YKL082C	0.8965
YIL021W	YJL140W	0.9999
YIL021W	YJL168C	0.8953
YIL021W	YLR071C	0.9834
YIL021W	YML010W	0.9904
YIL021W	YNR010W	0.9215
YIL021W	YOL005C	1.0000
YIL021W	YOR151C	1.0000
YIL021W	YOR210W	0.9948
YIL021W	YOR224C	1.0000
YIL021W	YPL129W	0.9499
YIL021W	YPR093C	0.9021
YIL021W	YPR187W	0.9999
YIL022W	YJL104W	0.9999
YIL022W	YJL143W	0.9995
YIL022W	YJR045C	1.0000
YIL022W	YLR008C	0.9941
YIL022W	YNR017W	1.0000
YIL022W	YOR232W	0.9881
YIL026C	YIL126W	0.9215
YIL026C	YJL074C	0.9967
YIL026C	YMR001C	0.7380
YIL030C	YML013W	0.8953
YIL033C	YJL164C	0.9993
YIL033C	YKL166C	0.9706
YIL033C	YPL203W	0.9996
YIL034C	YIR003W	0.8028
YIL034C	YKL007W	0.9866
YIL035C	YIL084C	0.7380
YIL035C	YJR093C	0.9382
YIL035C	YJR144W	0.7380
YIL035C	YKL088W	0.9313
YIL035C	YKL112W	0.9655
YIL035C	YKL160W	0.9797
YIL035C	YLR196W	0.7380
YIL035C	YLR403W	0.8566
YIL035C	YLR418C	0.8659
YIL035C	YML069W	0.9648
YIL035C	YML074C	0.7543
YIL035C	YMR172W	0.9313
YIL035C	YNL330C	0.7380
YIL035C	YOL004W	0.7380
YIL035C	YOL145C	0.9648
YIL035C	YOR039W	0.9997
YIL035C	YOR061W	0.9996
YIL035C	YOR119C	0.8566

YIL035C	YPR143W	0.8087
YIL038C	YNL288W	0.9969
YIL038C	YNR052C	0.9409
YIL038C	YPR072W	0.9929
YIL043C	YLL039C	0.7380
YIL045W	YLR258W	0.8353
YIL046W	YNL103W	1.0000
YIL050W	YPL031C	0.9999
YIL053W	YPL106C	0.7380
YIL053W	YPL201C	0.7253
YIL061C	YKL012W	0.9902
YIL061C	YLR275W	0.7380
YIL061C	YLR298C	0.9313
YIL061C	YML046W	0.9808
YIL061C	YMR125W	0.9820
YIL061C	YPL178W	0.8659
YIL061C	YPR182W	0.7380
YIL062C	YJR065C	0.9998
YIL062C	YKL013C	0.9861
YIL062C	YLR370C	0.9999
YIL062C	YMR109W	0.7891
YIL062C	YNR035C	0.9984
YIL063C	YLR293C	0.9977
YIL063C	YML121W	0.9138
YIL065C	YJL112W	1.0000
YIL065C	YKR036C	1.0000
YIL065C	YLL001W	0.9249
YIL068C	YJL085W	0.9887
YIL068C	YLR166C	0.9998
YIL068C	YPR055W	1.0000
YIL070C	YKL108W	0.7380
YIL070C	YNL137C	0.8659
YIL070C	YOR326W	0.8659
YIL071C	YJR007W	0.9249
YIL071C	YMR025W	0.8659
YIL071C	YOL117W	0.7380
YIL071C	YOR361C	0.9790
YIL072W	YLR263W	0.9994
YIL072W	YOR351C	0.8456
YIL074C	YNL311C	0.5774
YIL075C	YKL145W	0.9994
YIL075C	YLR180W	0.8659
YIL075C	YLR421C	0.9344
YIL075C	YMR191W	0.7380
YIL075C	YMR314W	0.8628
YIL075C	YOL038W	0.8628
YIL075C	YOR117W	0.7380

YIL075C	YOR259C	0.7380
YIL075C	YOR261C	0.9313
YIL075C	YPR108W	0.9624
YIL076W	YNL287W	0.8659
YIL076W	YPL010W	0.8659
YIL079C	YJL050W	0.8566
YIL079C	YOL115W	0.9985
YIL084C	YMR128W	0.7380
YIL084C	YMR263W	0.9743
YIL084C	YNL097C	0.9860
YIL084C	YNL330C	1.0000
YIL084C	YOL004W	0.9999
YIL084C	YPL139C	0.7380
YIL084C	YPL181W	0.9743
YIL091C	YJR002W	0.9852
YIL093C	YJR101W	0.7380
YIL093C	YJR113C	0.7380
YIL093C	YKL155C	0.8566
YIL093C	YNL081C	0.7380
YIL093C	YOR158W	0.7380
YIL093C	YPL013C	0.7380
YIL094C	YOR136W	0.7380
YIL095W	YIR006C	0.9979
YIL095W	YNL243W	0.7543
YIL095W	YOR181W	0.7543
YIL097W	YMR135C	0.9021
YIL098C	YJL180C	0.7380
YIL103W	YKL191W	0.9433
YIL103W	YOR133W	0.7380
YIL104C	YLR175W	0.8566
YIL104C	YNL124W	0.8258
YIL105C	YJL058C	0.9249
YIL105C	YKL203C	0.9790
YIL105C	YLR433C	0.9710
YIL105C	YMR068W	0.9963
YIL105C	YNL047C	0.8179
YIL105C	YPL059W	0.5774
YIL106W	YNR052C	0.9099
YIL106W	YPR111W	0.9266
YIL108W	YPR154W	0.5774
YIL109C	YLR026C	0.9929
YIL109C	YLR268W	0.9768
YIL109C	YPL085W	0.9991
YIL109C	YPL218W	0.9969
YIL109C	YPR181C	1.0000
YIL112W	YKR029C	0.9948
YIL112W	YMR273C	0.7380

YIL112W	YOL068C	0.9800
YIL115C	YJL041W	0.9998
YIL115C	YJL061W	1.0000
YIL115C	YLL024C	0.8087
YIL115C	YLR293C	0.9138
YIL115C	YLR347C	0.9780
YIL115C	YMR047C	0.9828
YIL115C	YMR235C	0.8998
YIL115C	YOR046C	0.9965
YIL115C	YPL169C	0.8566
YIL118W	YJL085W	1.0000
YIL118W	YOR326W	0.9723
YIL124W	YPL106C	0.7380
YIL126W	YKR001C	0.7380
YIL126W	YKR008W	0.9999
YIL126W	YLR033W	0.9908
YIL126W	YLR321C	0.9981
YIL126W	YLR357W	1.0000
YIL126W	YML127W	0.9891
YIL126W	YMR033W	0.9998
YIL126W	YMR091C	0.9974
YIL126W	YPL082C	0.9726
YIL126W	YPL129W	0.9932
YIL126W	YPR034W	0.9998
YIL126W	YPR052C	0.9215
YIL129C	YNL161W	0.9996
YIL133C	YNL178W	0.8566
YIL133C	YOR206W	0.7380
YIL139C	YLR288C	0.9932
YIL139C	YOR346W	1.0000
YIL139C	YPL167C	0.9998
YIL139C	YPL194W	0.9917
YIL140W	YLR229C	0.9249
YIL142W	YJL014W	0.9772
YIL142W	YJL111W	0.7380
YIL142W	YNL212W	0.7380
YIL142W	YOR281C	0.7380
YIL142W	YPL226W	0.7380
YIL143C	YOL090W	0.9099
YIL143C	YOR352W	0.9213
YIL143C	YPL122C	0.9758
YIL143C	YPL240C	0.9249
YIL143C	YPR056W	0.9099
YIL144W	YIR010W	0.9266
YIL144W	YMR117C	1.0000
YIL144W	YOL069W	1.0000
YIL144W	YPL174C	0.5774

YIL146C	YPR049C	0.9099
YIL147C	YIR004W	0.9138
YIL147C	YJR074W	0.9138
YIL149C	YKL042W	0.8953
YIL149C	YKR095W	0.8603
YIL149C	YPL169C	0.8566
YIL150C	YKL045W	0.9215
YIL150C	YLR442C	0.8965
YIL153W	YMR028W	0.9215
YIL153W	YNL201C	0.7380
YIL156W	YKL129C	0.8640
YIL156W	YMR109W	0.5774
YIL157C	YLR203C	0.9936
YIL157C	YML129C	0.9780
YIL159W	YKL079W	0.9138
YIL159W	YKL129C	0.7909
YIL159W	YKR055W	0.9138
YIL159W	YLR191W	0.5774
YIL159W	YLR319C	0.9138
YIL159W	YMR032W	0.9883
YIL159W	YMR109W	0.5774
YIL159W	YOR122C	0.7909
YIL161W	YNL023C	0.9313
YIL173W	YJR022W	0.5774
YIR001C	YNL016W	0.8028
YIR001C	YNL138W	0.7380
YIR002C	YOL034W	0.9932
YIR003W	YJL020C	0.6819
YIR003W	YKL007W	0.9313
YIR005W	YLL036C	0.9186
YIR005W	YLR016C	0.9928
YIR005W	YMR240C	0.8603
YIR005W	YPR094W	0.9478
YIR006C	YKL129C	0.9758
YIR006C	YMR109W	0.9758
YIR006C	YNL020C	0.7543
YIR006C	YNL084C	1.0000
YIR006C	YOR329C	0.9758
YIR008C	YKL045W	0.9583
YIR008C	YNL102W	0.9927
YIR008C	YNL262W	0.9574
YIR008C	YNR052C	0.7380
YIR008C	YPR019W	0.8953
YIR009W	YLL036C	0.9186
YIR009W	YNL091W	0.7909
YIR009W	YPL213W	0.9999
YIR010W	YJR112W	0.9998

YIR010W	YJR135C	0.7380
YIR010W	YKL049C	0.9598
YIR010W	YKL089W	0.9850
YIR010W	YLR381W	0.7380
YIR010W	YMR117C	0.7380
YIR010W	YOL069W	0.7380
YIR010W	YPL018W	0.9866
YIR010W	YPL233W	0.9996
YIR011C	YNL189W	0.9099
YIR012W	YLR075W	0.9987
YIR015W	YNL221C	0.8566
YIR017C	YJR060W	0.9138
YIR017C	YNL103W	0.9978
YIR022W	YJR010C-A	0.9388
YIR025W	YKL022C	0.9999
YIR025W	YLR102C	0.8603
YIR025W	YLR127C	0.9945
YIR025W	YNL172W	0.9805
YIR025W	YOR249C	0.9900
YIR034C	YLL039C	0.7380
YIR035C	YNL232W	0.7380
YIR037W	YJR009C	0.9719
YIR037W	YML007W	0.9249
YIR037W	YML070W	0.8998
YIR037W	YPR035W	0.9962
YJL001W	YML092C	0.8659
YJL001W	YMR314W	0.9157
YJL001W	YOL038W	0.8659
YJL001W	YOR157C	0.9926
YJL001W	YOR362C	0.9313
YJL001W	YPR103W	0.9997
YJL002C	YML019W	0.9999
YJL002C	YML130C	0.7380
YJL002C	YMR146C	0.7380
YJL002C	YMR149W	0.9992
YJL002C	YOR085W	1.0000
YJL002C	YOR103C	0.9875
YJL005W	YNL098C	0.8655
YJL005W	YNL138W	0.9999
YJL005W	YOR057W	0.9215
YJL006C	YKL139W	1.0000
YJL006C	YML112W	0.9990
YJL008C	YJL014W	0.9772
YJL008C	YJL074C	0.7380
YJL008C	YLL039C	0.8566
YJL008C	YMR128W	0.7380
YJL010C	YLR197W	0.8087

YJL011C	YKL144C	0.9779
YJL011C	YKR025W	0.8659
YJL011C	YNL113W	0.7380
YJL011C	YNL151C	0.9157
YJL011C	YNR003C	0.9313
YJL011C	YOR116C	0.9648
YJL011C	YOR207C	0.9904
YJL011C	YPR110C	0.8659
YJL011C	YPR190C	0.8659
YJL012C	YLL039C	0.7380
YJL013C	YJL030W	0.9215
YJL013C	YOR026W	0.9998
YJL014W	YJL111W	0.9772
YJL014W	YJR064W	0.9772
YJL019W	YJL073W	0.8965
YJL019W	YLR233C	0.8258
YJL019W	YMR001C	0.5774
YJL019W	YOL012C	0.5774
YJL019W	YOL104C	0.9099
YJL020C	YKL129C	0.9883
YJL020C	YLR337C	0.5774
YJL020C	YMR032W	0.5774
YJL020C	YMR109W	0.9950
YJL020C	YNL094W	0.5774
YJL020C	YOR181W	0.9896
YJL024C	YPL195W	0.8659
YJL025W	YML043C	0.9938
YJL025W	YMR270C	0.9138
YJL026W	YLR437C	0.9282
YJL026W	YOR229W	0.9915
YJL026W	YOR230W	0.9996
YJL029C	YKR020W	0.9707
YJL029C	YLR262C	0.9921
YJL030W	YOR370C	0.7696
YJL030W	YPR176C	0.7696
YJL031C	YOR370C	0.9779
YJL031C	YPR176C	0.9729
YJL033W	YJR093C	0.7380
YJL033W	YKR002W	0.7380
YJL033W	YLR115W	0.7380
YJL033W	YLR175W	0.7380
YJL033W	YLR197W	0.8659
YJL033W	YLR277C	0.7380
YJL033W	YMR061W	0.7380
YJL033W	YMR290C	0.8659
YJL033W	YNL317W	0.7380
YJL033W	YOL041C	0.7380

YJL033W	YOR310C	0.7380
YJL034W	YJL073W	0.9903
YJL034W	YKL073W	0.9983
YJL034W	YMR200W	0.9215
YJL034W	YMR214W	0.8659
YJL034W	YMR307W	0.9215
YJL034W	YOL031C	0.9999
YJL034W	YOR254C	0.9994
YJL036W	YLR423C	0.9414
YJL041W	YJL061W	1.0000
YJL041W	YJL063C	0.7380
YJL041W	YLR293C	0.9249
YJL041W	YLR312W-A	0.7380
YJL041W	YLR347C	0.9780
YJL041W	YMR024W	0.7380
YJL041W	YMR047C	0.9984
YJL041W	YMR193W	0.7380
YJL041W	YMR308C	0.9215
YJL041W	YPL169C	0.9616
YJL044C	YLR262C	0.9503
YJL047C	YLR320W	0.9968
YJL047C	YOL063C	0.8566
YJL047C	YOL133W	0.9700
YJL047C	YPR164W	0.9780
YJL048C	YKL130C	0.5774
YJL050W	YNL299W	0.9993
YJL050W	YOL115W	1.0000
YJL050W	YOR001W	0.9464
YJL050W	YPL190C	0.9313
YJL052W	YJR009C	0.7380
YJL052W	YLL039C	0.7380
YJL053W	YJL154C	0.9999
YJL053W	YOR069W	0.9988
YJL053W	YOR132W	0.9892
YJL054W	YOR297C	0.9780
YJL054W	YPR024W	0.9215
YJL058C	YKL203C	0.8953
YJL058C	YNL047C	0.9249
YJL061W	YKL057C	0.9949
YJL061W	YLR423C	0.5774
YJL061W	YMR047C	1.0000
YJL062W-A	YLR203C	0.9938
YJL062W-A	YML129C	0.9938
YJL063C	YML025C	0.7380
YJL065C	YOR304W	0.9971
YJL069C	YJL109C	0.9648
YJL069C	YJR002W	0.9707

YJL069C	YLR129W	0.9908
YJL069C	YLR222C	0.9976
YJL069C	YLR409C	0.9984
YJL069C	YMR093W	0.9313
YJL069C	YOR059C	0.7380
YJL070C	YML035C	0.8297
YJL072C	YOL146W	0.9640
YJL072C	YPR019W	0.9707
YJL072C	YPR135W	0.9939
YJL074C	YMR001C	0.9021
YJL074C	YOL041C	0.7380
YJL074C	YOL069W	0.5774
YJL074C	YPR007C	0.8566
YJL074C	YPR068C	0.9973
YJL076W	YJR057W	0.8566
YJL076W	YKR010C	0.9285
YJL076W	YOR341W	0.9598
YJL076W	YPR010C	0.9598
YJL080C	YJR123W	0.7380
YJL080C	YMR116C	0.8659
YJL080C	YMR125W	0.7380
YJL080C	YOR198C	0.9938
YJL081C	YLR052W	0.7380
YJL081C	YLR399C	0.8566
YJL081C	YML041C	0.7380
YJL081C	YNL059C	0.9499
YJL081C	YNL107W	0.9989
YJL081C	YNL136W	0.8659
YJL081C	YOL012C	0.9900
YJL081C	YOR141C	0.9634
YJL081C	YOR189W	0.8087
YJL081C	YOR244W	1.0000
YJL081C	YPL129W	0.9499
YJL081C	YPL235W	0.9021
YJL081C	YPR023C	0.9499
YJL082W	YLL039C	0.7380
YJL084C	YPL031C	0.6042
YJL085W	YLR166C	0.9946
YJL085W	YLR229C	0.9215
YJL085W	YPR055W	0.9994
YJL088W	YPL111W	0.9723
YJL089W	YMR280C	0.8965
YJL089W	YPL042C	0.9099
YJL090C	YKL108W	1.0000
YJL090C	YNL262W	0.9099
YJL090C	YPL194W	0.9723
YJL092W	YLR265C	0.9099

YJL092W	YMR190C	0.9747
YJL092W	YMR224C	0.9747
YJL092W	YOR355W	0.7909
YJL093C	YLR113W	0.8655
YJL095W	YKL129C	0.7837
YJL095W	YLL008W	0.5779
YJL095W	YMR109W	0.5774
YJL095W	YMR229C	0.5779
YJL095W	YOR231W	0.8760
YJL095W	YPL140C	0.9157
YJL098W	YLR310C	0.7380
YJL098W	YOR061W	0.7380
YJL098W	YOR267C	0.8659
YJL098W	YPL204W	0.8659
YJL098W	YPR040W	0.7380
YJL099W	YKR027W	0.9215
YJL099W	YLR330W	0.9999
YJL099W	YMR237W	0.9186
YJL099W	YOR299W	0.9794
YJL100W	YOR181W	0.8965
YJL104W	YJL143W	1.0000
YJL104W	YLR008C	1.0000
YJL104W	YNR017W	1.0000
YJL104W	YPL063W	0.9780
YJL106W	YJL194W	0.9099
YJL106W	YJR094C	0.8456
YJL106W	YLR079W	0.7696
YJL106W	YNL312W	0.7696
YJL109C	YJL190C	0.7380
YJL109C	YJR002W	0.9743
YJL109C	YJR145C	0.7380
YJL109C	YLL011W	0.8659
YJL109C	YLR186W	0.8659
YJL109C	YLR197W	0.8659
YJL109C	YLR222C	0.8659
YJL109C	YLR409C	0.9313
YJL109C	YLR441C	0.7380
YJL109C	YMR093W	0.9985
YJL109C	YMR186W	0.7380
YJL109C	YMR229C	0.7380
YJL109C	YOL010W	0.7380
YJL109C	YOR078W	0.7380
YJL109C	YOR310C	0.9743
YJL109C	YPL043W	0.7380
YJL109C	YPL126W	0.9990
YJL109C	YPL217C	0.8659
YJL109C	YPR137W	0.9648

YJL109C	YPR144C	0.8659
YJL110C	YNL021W	0.5774
YJL111W	YOR281C	0.8659
YJL112W	YKR036C	0.9710
YJL112W	YLL001W	1.0000
YJL115W	YJL168C	0.9707
YJL115W	YJR140C	0.9634
YJL115W	YLL002W	0.9755
YJL115W	YMR127C	0.9747
YJL115W	YNL030W	0.9998
YJL115W	YNL031C	1.0000
YJL115W	YOR038C	0.9946
YJL115W	YOR213C	0.9099
YJL115W	YPL153C	1.0000
YJL122W	YMR308C	0.9927
YJL124C	YKL173W	0.6672
YJL124C	YLR438C-A	1.0000
YJL124C	YMR080C	0.7380
YJL124C	YNL118C	0.9624
YJL124C	YNL145W	0.3670
YJL124C	YNL147W	1.0000
YJL124C	YOR308C	0.5276
YJL124C	YOR375C	0.5774
YJL125C	YNL062C	1.0000
YJL128C	YLR113W	0.9962
YJL128C	YLR362W	0.9894
YJL128C	YNR031C	0.9922
YJL137C	YLR258W	0.9879
YJL138C	YKR059W	0.7380
YJL138C	YOL139C	0.7380
YJL140W	YLR071C	0.9834
YJL140W	YML010W	0.9499
YJL140W	YOL005C	0.9021
YJL140W	YOR151C	0.7380
YJL140W	YOR210W	0.7380
YJL140W	YOR224C	0.9634
YJL140W	YPL129W	0.8659
YJL140W	YPR052C	0.8958
YJL140W	YPR187W	0.9157
YJL141C	YJL164C	0.7347
YJL141C	YNR052C	0.8655
YJL141C	YPL203W	0.5779
YJL141C	YPL247C	0.7380
YJL143W	YJR045C	0.9780
YJL143W	YKR065C	0.9215
YJL143W	YLR008C	0.9995
YJL143W	YNR017W	1.0000

YJL143W	YPL063W	0.9983
YJL146W	YPL154C	0.8659
YJL148W	YJR063W	0.7380
YJL148W	YMR239C	0.9099
YJL148W	YNL113W	0.8659
YJL148W	YNL248C	0.9981
YJL148W	YOR210W	0.7380
YJL148W	YOR340C	0.9624
YJL148W	YOR341W	0.9928
YJL148W	YPR010C	0.8659
YJL148W	YPR110C	0.9313
YJL148W	YPR187W	0.8659
YJL151C	YLL039C	0.9598
YJL154C	YOR069W	1.0000
YJL154C	YOR132W	0.9987
YJL155C	YLR345W	0.9554
YJL157C	YOR212W	0.9758
YJL157C	YPL256C	0.9968
YJL157C	YPR120C	0.8462
YJL164C	YKL166C	0.8894
YJL164C	YLR133W	0.9257
YJL164C	YLR310C	0.9567
YJL164C	YMR196W	0.5779
YJL164C	YNL027W	0.8891
YJL164C	YOR347C	0.9382
YJL164C	YPL203W	0.9710
YJL164C	YPL232W	0.7696
YJL168C	YNL031C	0.9037
YJL168C	YOR151C	0.9890
YJL172W	YLL039C	0.8566
YJL173C	YLR347C	0.8659
YJL173C	YML032C	0.9157
YJL173C	YNL312W	0.9976
YJL176C	YMR033W	0.9996
YJL176C	YNL216W	0.9249
YJL176C	YNR023W	0.9970
YJL176C	YOR290C	1.0000
YJL176C	YPL016W	0.9946
YJL176C	YPL129W	0.9808
YJL176C	YPR034W	0.9970
YJL178C	YLR431C	0.9990
YJL179W	YKL215C	0.7380
YJL179W	YLR189C	0.7380
YJL179W	YLR216C	0.7380
YJL179W	YLR424W	0.7380
YJL179W	YML094W	0.9313
YJL179W	YNL153C	0.9313

YJL183W	YJR075W	0.9794
YJL183W	YPL050C	0.9992
YJL184W	YKR038C	0.9869
YJL184W	YML036W	0.9499
YJL184W	YOR117W	0.7253
YJL187C	YKL042W	0.5779
YJL187C	YMR001C	0.9998
YJL187C	YPL240C	0.8655
YJL187C	YPR082C	0.4640
YJL187C	YPR119W	0.9934
YJL190C	YMR125W	0.7380
YJL194W	YKL145W	0.7380
YJL194W	YLR274W	0.8818
YJL194W	YML065W	0.9799
YJL194W	YNL189W	0.9107
YJL194W	YNL261W	0.8998
YJL194W	YPR119W	0.9937
YJL203W	YKL173W	0.7380
YJL203W	YLL036C	0.9186
YJL203W	YML049C	0.8659
YJL203W	YMR240C	0.9648
YJL203W	YMR288W	0.7380
YJL203W	YOR319W	0.7380
YJL203W	YPL213W	0.9021
YJL203W	YPR178W	0.7380
YJL207C	YPR029C	0.8804
YJL208C	YKR079C	0.9648
YJL208C	YMR099C	0.8659
YJL210W	YLR191W	0.9707
YJL210W	YMR026C	0.9707
YJR002W	YLR129W	0.8087
YJR002W	YLR186W	0.9464
YJR002W	YLR222C	0.8087
YJR002W	YLR409C	0.9850
YJR002W	YMR093W	0.9464
YJR002W	YMR128W	0.8566
YJR002W	YNL075W	0.9852
YJR002W	YOR078W	0.9499
YJR002W	YOR096W	0.8566
YJR002W	YOR310C	0.9499
YJR002W	YPL126W	0.8087
YJR002W	YPR137W	0.9624
YJR002W	YPR144C	0.9464
YJR005W	YJR058C	0.9464
YJR005W	YOL062C	0.9726
YJR006W	YJR043C	0.9999
YJR007W	YKR026C	0.9891

YJR007W	YLR291C	0.9860
YJR007W	YOR260W	0.9999
YJR007W	YPL237W	0.9943
YJR007W	YPR041W	0.9989
YJR009C	YML053C	0.5774
YJR010C-A	YML055W	0.6147
YJR010C-A	YML067C	0.4335
YJR010W	YNL189W	0.5774
YJR022W	YKL173W	0.8087
YJR022W	YLR147C	0.8087
YJR022W	YLR275W	0.7380
YJR022W	YLR438C-A	0.9985
YJR022W	YMR268C	0.9954
YJR022W	YNL050C	0.5774
YJR022W	YNL118C	0.5774
YJR022W	YNL147W	0.9977
YJR022W	YNR050C	0.5774
YJR022W	YNR053C	0.5774
YJR022W	YOR076C	0.5774
YJR022W	YOR308C	0.8087
YJR022W	YOR319W	0.8839
YJR022W	YPR178W	0.8756
YJR031C	YKR030W	0.9099
YJR031C	YNL287W	0.9758
YJR032W	YLL026W	0.9790
YJR032W	YMR186W	0.8566
YJR032W	YNL330C	0.9138
YJR032W	YPL240C	0.9995
YJR033C	YKL080W	0.9099
YJR033C	YOR332W	0.9764
YJR041C	YKL014C	0.9860
YJR041C	YLR221C	0.8566
YJR041C	YOL144W	0.8566
YJR042W	YKL057C	0.9907
YJR042W	YKL068W	0.8087
YJR042W	YKR082W	0.7380
YJR042W	YLR208W	0.9996
YJR042W	YLR293C	0.8603
YJR042W	YMR047C	0.9902
YJR042W	YPL169C	0.9640
YJR043C	YNL102W	0.8965
YJR043C	YOR346W	0.9799
YJR045C	YKR048C	0.7380
YJR045C	YLR203C	0.9624
YJR045C	YNL310C	0.9996
YJR045C	YNR017W	1.0000
YJR045C	YOR232W	1.0000

YJR046W	YLR274W	0.9249
YJR046W	YMR078C	0.9215
YJR046W	YNL261W	0.9138
YJR046W	YPR019W	0.9574
YJR048W	YKR066C	0.9961
YJR049C	YPL026C	0.5779
YJR050W	YLL036C	1.0000
YJR050W	YLR117C	0.9996
YJR050W	YMR213W	0.9774
YJR050W	YPL151C	0.7380
YJR050W	YPL213W	0.8798
YJR050W	YPR101W	0.7380
YJR052W	YLR442C	0.9820
YJR052W	YPL046C	0.9983
YJR053W	YML064C	1.0000
YJR053W	YMR001C	0.9929
YJR053W	YMR055C	0.9999
YJR053W	YOR233W	0.8655
YJR053W	YOR373W	0.9037
YJR058C	YOL062C	0.8566
YJR060W	YNL103W	0.9981
YJR063W	YNL113W	0.8659
YJR063W	YNL248C	0.7380
YJR063W	YOR210W	0.9313
YJR063W	YOR224C	0.8659
YJR063W	YOR340C	0.9902
YJR063W	YOR341W	0.9981
YJR063W	YPR010C	0.9973
YJR063W	YPR110C	0.9820
YJR063W	YPR187W	0.9313
YJR064W	YNR016C	0.7380
YJR065C	YKL013C	0.9946
YJR065C	YLR370C	0.9998
YJR065C	YNR035C	0.9992
YJR065C	YNR053C	0.7380
YJR065C	YOR181W	0.8566
YJR066W	YKL161C	0.8566
YJR066W	YKL171W	0.8566
YJR066W	YMR028W	0.9555
YJR066W	YMR216C	0.8566
YJR066W	YNL006W	1.0000
YJR066W	YNL076W	0.8566
YJR066W	YNL135C	0.9249
YJR066W	YNL183C	0.8566
YJR066W	YPL180W	0.9936
YJR067C	YNL260C	0.8659
YJR068W	YMR078C	0.9991

YJR068W	YNL290W	1.0000
YJR068W	YOL094C	1.0000
YJR068W	YOR144C	0.9942
YJR068W	YOR217W	0.9999
YJR072C	YNL289W	0.9884
YJR074W	YLR293C	0.9996
YJR074W	YOR185C	0.7837
YJR075W	YPL050C	0.9999
YJR076C	YJR092W	0.7380
YJR076C	YLR314C	1.0000
YJR076C	YMR001C	0.9099
YJR076C	YNL166C	0.9723
YJR076C	YPL153C	0.9464
YJR076C	YPL161C	0.7909
YJR077C	YKL182W	0.7380
YJR082C	YNL107W	0.9021
YJR082C	YNL136W	0.8659
YJR082C	YOR244W	0.9787
YJR082C	YPL106C	0.7380
YJR082C	YPR023C	0.7380
YJR084W	YMR025W	0.9893
YJR084W	YOL117W	0.9893
YJR086W	YOR212W	0.9996
YJR089W	YPL209C	0.9964
YJR090C	YML006C	0.9099
YJR090C	YMR032W	0.8894
YJR090C	YNL076W	0.9790
YJR090C	YOL133W	0.9922
YJR090C	YPL256C	0.9994
YJR091C	YOR265W	0.5774
YJR092W	YPL153C	0.8087
YJR092W	YPL242C	0.9099
YJR093C	YKL018W	0.9813
YJR093C	YKL059C	0.9991
YJR093C	YKR002W	1.0000
YJR093C	YLR115W	0.9969
YJR093C	YLR221C	0.7380
YJR093C	YLR277C	0.9995
YJR093C	YMR061W	0.9902
YJR093C	YNL222W	0.9733
YJR093C	YNL317W	0.9974
YJR093C	YOR061W	0.7696
YJR093C	YPR107C	1.0000
YJR094C	YMR139W	0.9986
YJR099W	YLL039C	0.9764
YJR101W	YKL155C	0.8566
YJR101W	YOR158W	0.7380

YJR101W	YPL013C	0.7380
YJR102C	YLR417W	0.9880
YJR102C	YMR077C	0.9636
YJR102C	YPL002C	0.9960
YJR102C	YPL065W	0.9414
YJR104C	YMR038C	0.9994
YJR109C	YOR303W	0.8659
YJR112W	YKL049C	0.9215
YJR112W	YOL069W	0.8179
YJR112W	YPL233W	0.9979
YJR112W	YPR141C	0.5774
YJR113C	YKL155C	0.9266
YJR113C	YNL306W	0.7380
YJR113C	YOR158W	0.7380
YJR113C	YPL013C	0.7380
YJR119C	YNL031C	0.7696
YJR121W	YKL016C	0.9960
YJR121W	YLR295C	0.9960
YJR121W	YNL315C	0.9071
YJR121W	YPL078C	0.9994
YJR121W	YPL271W	0.9960
YJR123W	YMR093W	0.7380
YJR123W	YPR137W	0.7380
YJR123W	YPR189W	0.7380
YJR125C	YMR197C	0.9723
YJR125C	YOR036W	0.8798
YJR125C	YOR111W	0.5774
YJR125C	YPR029C	0.9351
YJR132W	YLR293C	0.9249
YJR132W	YOR326W	0.7380
YJR134C	YLR262C	0.9918
YJR134C	YOR216C	0.6000
YJR134C	YOR326W	0.6000
YJR135C	YLR381W	0.9690
YJR135C	YPL018W	0.7380
YJR135C	YPR046W	0.9979
YJR135W-A	YNR017W	0.9703
YJR136C	YKL033W	0.5774
YJR137C	YPL106C	0.7380
YJR140C	YOR038C	0.9285
YJR145C	YLR175W	0.7380
YJR145C	YML056C	0.7380
YJR145C	YMR229C	0.7380
YJR145C	YNL132W	0.7380
YJR145C	YNL308C	0.7380
YJR145C	YOL120C	0.7380
YJR145C	YOR310C	0.7380

YJR145C	YPL012W	0.7380
YJR145C	YPR137W	0.7380
YJR145C	YPR144C	0.8659
YKL001C	YNL311C	0.7253
YKL002W	YKL041W	0.9938
YKL002W	YLR025W	0.7891
YKL002W	YMR077C	0.6000
YKL002W	YPR173C	0.9997
YKL005C	YNL031C	0.9947
YKL006C-A	YKL196C	0.9282
YKL006C-A	YLR026C	0.9984
YKL006C-A	YLR078C	0.9282
YKL006C-A	YLR268W	0.9282
YKL008C	YMR298W	0.9904
YKL009W	YKR081C	0.8566
YKL009W	YLL045C	0.9586
YKL009W	YLR002C	0.8566
YKL009W	YLR009W	0.8953
YKL009W	YLR074C	0.9266
YKL009W	YMR049C	0.9266
YKL009W	YMR290C	0.9598
YKL009W	YNL002C	0.8566
YKL009W	YNL061W	0.9895
YKL009W	YNL110C	0.9266
YKL009W	YNL175C	0.8566
YKL009W	YNL182C	0.8566
YKL009W	YNR053C	0.9794
YKL009W	YOL077C	0.7380
YKL009W	YOR063W	0.8566
YKL009W	YOR206W	0.7380
YKL009W	YOR272W	0.7380
YKL009W	YPL093W	0.9850
YKL009W	YPL131W	0.9598
YKL009W	YPR016C	0.9961
YKL010C	YOR259C	0.9099
YKL012W	YLR116W	0.9099
YKL012W	YLR117C	0.9138
YKL012W	YLR147C	0.8659
YKL012W	YLR275W	0.7380
YKL012W	YLR298C	0.9313
YKL012W	YML046W	0.9313
YKL012W	YMR125W	0.9313
YKL012W	YOR159C	0.7380
YKL012W	YPR182W	0.8659
YKL013C	YKL129C	0.9747
YKL013C	YLR370C	0.9918
YKL013C	YMR109W	0.9099

YKL013C	YNR035C	0.9717
YKL013C	YOR181W	0.6147
YKL014C	YMR290C	0.8087
YKL014C	YNL061W	0.9499
YKL014C	YOL144W	0.9021
YKL014C	YPL043W	0.9021
YKL018W	YKL059C	0.9930
YKL018W	YKR002W	0.9499
YKL018W	YLR015W	0.9995
YKL018W	YLR115W	0.9813
YKL018W	YLR277C	0.9813
YKL018W	YMR061W	0.7380
YKL018W	YNL031C	0.8854
YKL018W	YNL222W	0.9634
YKL018W	YNL317W	0.9813
YKL018W	YOR179C	0.9813
YKL018W	YPL138C	0.9930
YKL018W	YPR107C	0.9499
YKL021C	YLR009W	0.9609
YKL021C	YMR049C	0.8087
YKL021C	YNL061W	0.8087
YKL021C	YPR016C	0.9285
YKL022C	YLR102C	1.0000
YKL022C	YLR127C	1.0000
YKL022C	YNL172W	1.0000
YKL022C	YOR249C	1.0000
YKL023W	YKR092C	0.5774
YKL023W	YLR398C	0.7380
YKL028W	YKR062W	0.9925
YKL028W	YOL051W	0.9939
YKL029C	YKL104C	0.7380
YKL029C	YLR106C	0.7380
YKL029C	YML124C	0.7380
YKL029C	YMR012W	0.7380
YKL035W	YOL045W	0.7543
YKL038W	YOR047C	0.9995
YKL041W	YLR025W	0.9653
YKL041W	YMR077C	0.9983
YKL041W	YPL002C	0.9215
YKL042W	YNL225C	0.9998
YKL042W	YPL124W	0.9978
YKL042W	YPR120C	0.8353
YKL045W	YNL102W	0.8659
YKL045W	YNR052C	0.7380
YKL049C	YKL089W	0.9640
YKL049C	YPL018W	0.9644
YKL051W	YLR305C	0.9215

YKL052C	YKL138C-A	0.9919
YKL052C	YKR037C	0.9999
YKL052C	YKR083C	0.9992
YKL054C	YPL106C	0.7380
YKL057C	YKL068W	0.8087
YKL057C	YKR082W	0.9298
YKL057C	YLR208W	0.9648
YKL057C	YMR047C	0.9406
YKL058W	YML015C	0.9723
YKL058W	YOR194C	1.0000
YKL059C	YKR002W	0.9975
YKL059C	YLR115W	0.9987
YKL059C	YLR277C	0.9987
YKL059C	YMR061W	0.7380
YKL059C	YNL222W	0.9285
YKL059C	YNL317W	0.9982
YKL059C	YPR107C	0.9933
YKL060C	YOR116C	0.7380
YKL061W	YNL086W	0.7253
YKL065C	YOR254C	0.9215
YKL067W	YML064C	0.6672
YKL067W	YPR010C	0.7380
YKL068W	YKR082W	0.8087
YKL068W	YLL024C	0.8087
YKL068W	YLR335W	0.9464
YKL068W	YLR347C	0.9992
YKL068W	YML103C	0.8087
YKL068W	YMR047C	0.9940
YKL068W	YMR080C	0.9099
YKL068W	YMR308C	0.8087
YKL068W	YNL189W	0.8087
YKL068W	YNL273W	0.8087
YKL068W	YOL123W	0.8087
YKL068W	YPL169C	0.9487
YKL074C	YLR116W	1.0000
YKL074C	YLR117C	0.9971
YKL074C	YMR240C	0.8965
YKL075C	YNL091W	0.7837
YKL078W	YNL186W	0.8028
YKL079W	YLR309C	0.6000
YKL079W	YOR326W	0.9978
YKL079W	YPR049C	0.6000
YKL080W	YOR270C	0.9773
YKL080W	YOR332W	0.9895
YKL081W	YMR186W	0.8659
YKL081W	YPL048W	0.7380
YKL081W	YPL226W	0.7380

YKL081W	YPR080W	0.8659
YKL082C	YKL099C	0.8965
YKL082C	YKL172W	0.8965
YKL082C	YMR143W	0.8965
YKL084W	YKL195W	0.9249
YKL088W	YKR072C	0.9483
YKL088W	YML016C	0.9483
YKL088W	YOR039W	0.9313
YKL088W	YOR054C	0.9803
YKL088W	YOR061W	0.9313
YKL090W	YLL039C	0.9764
YKL095W	YLL036C	0.9899
YKL095W	YLR117C	0.9539
YKL095W	YMR213W	0.8566
YKL095W	YPL151C	0.7380
YKL095W	YPR101W	0.9266
YKL101W	YKR048C	0.6823
YKL101W	YLR113W	0.8655
YKL101W	YLR314C	0.9215
YKL101W	YNL132W	0.7380
YKL103C	YOL082W	0.9999
YKL104C	YLR180W	0.7380
YKL104C	YMR308C	0.7380
YKL104C	YOR151C	0.7380
YKL104C	YPR010C	0.7380
YKL108W	YPR120C	0.7183
YKL109W	YOR290C	0.9215
YKL109W	YOR358W	0.9282
YKL110C	YLR384C	0.9875
YKL110C	YPL086C	1.0000
YKL110C	YPL204W	0.9215
YKL116C	YPL031C	0.5779
YKL117W	YLR216C	0.9138
YKL117W	YMR186W	0.9921
YKL117W	YPL240C	1.0000
YKL119C	YLR447C	0.7380
YKL119C	YOR270C	0.8566
YKL122C	YML105C	0.9991
YKL122C	YPL210C	0.8659
YKL122C	YPL243W	0.7380
YKL122C	YPR088C	0.9313
YKL125W	YML126C	0.7380
YKL125W	YNL102W	0.9875
YKL125W	YOR341W	0.9215
YKL126W	YKL203C	0.6537
YKL126W	YNR047W	0.7696
YKL129C	YLR337C	1.0000

YKL129C	YMR109W	0.7837
YKL129C	YNL271C	0.6185
YKL129C	YOR035C	0.9710
YKL129C	YOR181W	0.9997
YKL129C	YOR247W	0.5774
YKL130C	YKL185W	0.8711
YKL130C	YNL189W	0.9850
YKL135C	YLR170C	0.9902
YKL135C	YOL108C	0.7909
YKL135C	YPL259C	0.9990
YKL135C	YPR029C	0.9952
YKL138C-A	YKR037C	0.9998
YKL138C-A	YKR083C	0.8967
YKL139W	YML112W	0.9997
YKL139W	YNL004W	0.9266
YKL139W	YNL102W	0.9249
YKL142W	YMR210W	0.5774
YKL142W	YPL004C	0.8297
YKL143W	YML121W	0.8998
YKL143W	YNL178W	0.9670
YKL143W	YNL207W	0.9959
YKL143W	YPL204W	0.9021
YKL143W	YPL239W	0.8353
YKL144C	YKR025W	0.9648
YKL144C	YNL113W	0.8659
YKL144C	YNL151C	0.8659
YKL144C	YNR003C	0.9820
YKL144C	YOR116C	0.9865
YKL144C	YOR207C	0.9951
YKL144C	YOR210W	0.7380
YKL144C	YOR224C	0.8659
YKL144C	YPR110C	0.9648
YKL144C	YPR187W	0.9313
YKL144C	YPR190C	0.9648
YKL145W	YLL039C	0.9298
YKL145W	YLR421C	0.9726
YKL145W	YMR276W	0.9938
YKL145W	YOL038W	0.8521
YKL145W	YOR056C	0.9555
YKL145W	YOR117W	0.9992
YKL145W	YOR257W	0.9249
YKL145W	YOR259C	0.9717
YKL145W	YOR261C	0.9808
YKL145W	YPL096W	0.9215
YKL145W	YPR108W	0.9946
YKL152C	YLL039C	0.9021
YKL155C	YNL137C	0.8566

YKL155C	YNR037C	0.9266
YKL155C	YOR158W	0.8566
YKL155C	YPL013C	0.7380
YKL155C	YPL118W	0.8566
YKL159C	YLR433C	0.9559
YKL160W	YOR061W	0.8714
YKL161C	YLR096W	0.8566
YKL161C	YNL053W	0.6147
YKL161C	YPL089C	0.8760
YKL161C	YPL140C	0.8566
YKL166C	YNL227C	0.5779
YKL166C	YPL203W	0.9859
YKL166C	YPR160W	0.7380
YKL167C	YML025C	0.8659
YKL167C	YOL027C	0.8521
YKL167C	YPR125W	0.8521
YKL171W	YKL203C	0.8566
YKL171W	YNL229C	0.8921
YKL171W	YPL204W	0.8566
YKL172W	YKR081C	0.9624
YKL172W	YLR196W	0.8028
YKL172W	YMR049C	0.7380
YKL172W	YNL002C	0.6147
YKL172W	YNL110C	0.7380
YKL172W	YOL077C	0.9447
YKL172W	YOR156C	0.9099
YKL172W	YOR206W	0.6147
YKL172W	YOR272W	0.8087
YKL172W	YOR294W	0.9930
YKL173W	YLL036C	0.9787
YKL173W	YLR117C	0.9499
YKL173W	YLR147C	0.7380
YKL173W	YLR275W	0.8659
YKL173W	YLR424W	0.8659
YKL173W	YLR438C-A	0.9634
YKL173W	YMR213W	0.9313
YKL173W	YNL147W	0.9313
YKL173W	YOR159C	0.7380
YKL173W	YOR308C	0.7380
YKL173W	YPL151C	0.7380
YKL173W	YPL213W	0.8087
YKL173W	YPR082C	0.7380
YKL173W	YPR101W	0.7380
YKL173W	YPR178W	0.9313
YKL173W	YPR182W	0.7380
YKL179C	YOR216C	0.6000
YKL180W	YNL132W	0.7380

YKL182W	YPL231W	0.7380
YKL185W	YNL097C	0.8087
YKL185W	YNL330C	0.9900
YKL185W	YOL004W	0.8603
YKL186C	YPL169C	0.9997
YKL189W	YOR353C	0.7909
YKL190W	YLR433C	0.9955
YKL190W	YML057W	0.9648
YKL193C	YML016C	0.9648
YKL193C	YPL179W	0.7380
YKL195W	YLL009C	0.9881
YKL195W	YLL018C-A	0.9555
YKL196C	YLR026C	1.0000
YKL196C	YLR078C	0.9282
YKL196C	YLR093C	0.9780
YKL196C	YLR268W	0.9808
YKL196C	YLR440C	0.7380
YKL196C	YMR197C	1.0000
YKL196C	YOR036W	0.9803
YKL196C	YOR106W	1.0000
YKL196C	YPL232W	0.6000
YKL197C	YNL329C	0.9999
YKL197C	YOL044W	0.9759
YKL203C	YMR028W	0.9966
YKL203C	YMR068W	0.9994
YKL203C	YNL006W	1.0000
YKL203C	YNL047C	0.9249
YKL203C	YNL135C	0.9099
YKL203C	YNL183C	0.8566
YKL203C	YOL078W	0.9998
YKL203C	YPL268W	0.9099
YKL204W	YOL139C	0.9624
YKL205W	YLR293C	0.9976
YKL205W	YLR335W	0.9099
YKL205W	YOR112W	0.9249
YKL206C	YLR199C	0.9266
YKL209C	YLL039C	0.8566
YKL210W	YOR184W	0.7380
YKL212W	YLR350W	0.8953
YKL212W	YPR183W	0.9079
YKL213C	YLL039C	0.9997
YKL214C	YMR125W	0.9313
YKL214C	YPL178W	0.7380
YKL217W	YLL039C	0.9598
YKR001C	YMR072W	0.7380
YKR001C	YOR106W	0.9249
YKR001C	YPL082C	0.8659

YKR002W	YLR115W	0.9992
YKR002W	YLR277C	0.9950
YKR002W	YMR061W	0.9648
YKR002W	YNL222W	0.9930
YKR002W	YNL317W	0.9993
YKR002W	YPL031C	0.9710
YKR002W	YPR107C	0.9820
YKR006C	YLR439W	0.9464
YKR006C	YML025C	0.8659
YKR006C	YPL173W	0.8953
YKR007W	YML121W	0.9998
YKR008W	YLR033W	0.9953
YKR008W	YLR321C	0.9820
YKR008W	YLR357W	1.0000
YKR008W	YML127W	0.9820
YKR008W	YMR033W	0.9648
YKR008W	YMR091C	0.9908
YKR008W	YNL031C	0.9754
YKR008W	YPR034W	0.9820
YKR010C	YOL006C	0.9099
YKR014C	YLR026C	0.9555
YKR014C	YMR258C	0.9554
YKR014C	YNL044W	0.9099
YKR014C	YOR370C	0.8718
YKR022C	YLR424W	1.0000
YKR024C	YMR229C	0.7380
YKR025W	YNL151C	0.7380
YKR025W	YNR003C	0.8659
YKR025W	YOR116C	0.9648
YKR025W	YOR207C	0.9813
YKR025W	YPR110C	0.9313
YKR025W	YPR190C	0.8659
YKR026C	YLR291C	0.9998
YKR026C	YOR260W	0.9998
YKR026C	YPL237W	0.9624
YKR027W	YLR330W	0.9940
YKR027W	YMR237W	0.8566
YKR027W	YOR299W	0.9215
YKR029C	YMR273C	0.7380
YKR029C	YNL031C	0.9892
YKR029C	YOL068C	0.9800
YKR035W-A	YLR181C	0.9747
YKR035W-A	YNL265C	0.9806
YKR035W-A	YPR173C	1.0000
YKR036C	YLL001W	0.8894
YKR037C	YKR083C	0.9990
YKR037C	YLR045C	0.5774

YKR038C	YKR095W-A	0.8986
YKR038C	YML036W	0.9903
YKR039W	YLL039C	0.9598
YKR048C	YLL002W	0.9249
YKR048C	YLR410W	0.7380
YKR048C	YLR457C	0.9667
YKR048C	YMR139W	0.9771
YKR048C	YNL078W	0.9991
YKR048C	YOL012C	0.9999
YKR048C	YOL070C	0.8659
YKR048C	YPL150W	0.7839
YKR048C	YPL180W	0.8921
YKR048C	YPR119W	0.9632
YKR054C	YMR117C	0.5774
YKR055W	YPL161C	0.8640
YKR058W	YLR258W	0.6147
YKR059W	YNL132W	0.7380
YKR060W	YOR310C	0.7380
YKR060W	YPR144C	0.7380
YKR062W	YOL051W	0.9939
YKR062W	YPL106C	0.7380
YKR065C	YNR017W	0.9977
YKR068C	YLR342W	0.7380
YKR068C	YML077W	1.0000
YKR068C	YMR218C	0.9997
YKR068C	YOR115C	1.0000
YKR068C	YPL240C	0.6672
YKR071C	YLL039C	0.9021
YKR071C	YPR048W	0.9884
YKR072C	YML016C	0.9947
YKR079C	YMR099C	0.8659
YKR081C	YLL008W	0.8659
YKR081C	YLL034C	0.7380
YKR081C	YLR002C	0.8659
YKR081C	YLR074C	0.7380
YKR081C	YLR276C	0.8659
YKR081C	YLR449W	0.7380
YKR081C	YMR049C	0.9266
YKR081C	YMR229C	0.7380
YKR081C	YMR290C	0.8659
YKR081C	YNL002C	0.8659
YKR081C	YNL061W	0.8659
YKR081C	YNL110C	0.9648
YKR081C	YNR053C	0.9313
YKR081C	YOL077C	0.8659
YKR081C	YOL127W	0.7380
YKR081C	YOR063W	0.7380

YKR081C	YOR206W	0.9313
YKR081C	YOR272W	0.9726
YKR081C	YOR294W	1.0000
YKR081C	YPL093W	0.9499
YKR081C	YPL131W	0.9956
YKR081C	YPL198W	0.7380
YKR081C	YPL211W	0.7380
YKR081C	YPR016C	0.9648
YKR081C	YPR102C	0.9999
YKR082W	YLR208W	0.7380
YKR082W	YML103C	0.9932
YKR082W	YMR047C	0.9186
YKR083C	YLR423C	0.5774
YKR085C	YML025C	0.7380
YKR085C	YMR193W	0.7380
YKR095W	YPL169C	0.8566
YKR096W	YPL204W	0.8087
YKR099W	YLR058C	0.5774
YKR101W	YLR442C	0.9805
YKR101W	YML065W	1.0000
YKR101W	YPR018W	0.9922
YLL001W	YPL204W	0.7380
YLL002W	YNL031C	0.9981
YLL002W	YNL246W	1.0000
YLL003W	YOR257W	0.9944
YLL004W	YML065W	1.0000
YLL004W	YNL261W	0.9998
YLL004W	YPL001W	0.9944
YLL004W	YPL008W	0.9099
YLL004W	YPL138C	0.9215
YLL004W	YPR162C	0.9967
YLL006W	YOL009C	0.9834
YLL008W	YMR049C	0.7380
YLL008W	YNL061W	0.8659
YLL008W	YNL110C	0.9313
YLL008W	YOL077C	0.7380
YLL008W	YOR272W	0.9021
YLL008W	YPL043W	0.8659
YLL008W	YPR016C	0.9499
YLL009C	YLL013C	0.9251
YLL010C	YOR043W	0.9921
YLL011W	YMR308C	0.9904
YLL011W	YNL132W	0.7380
YLL011W	YPR137W	0.7380
YLL011W	YPR144C	0.8659
YLL013C	YOL009C	0.9099
YLL013C	YPL204W	0.7380

YLL017W	YNL098C	0.9382
YLL021W	YMR308C	0.7380
YLL021W	YNL271C	0.9138
YLL021W	YNL293W	0.9440
YLL021W	YOL112W	0.8258
YLL021W	YOR231W	0.9434
YLL021W	YOR326W	0.8953
YLL022C	YNL030W	0.9794
YLL022C	YNL031C	0.8028
YLL022C	YPL001W	0.9996
YLL024C	YLL029W	0.7380
YLL024C	YLL039C	0.9021
YLL024C	YLR071C	0.7380
YLL024C	YLR180W	0.7380
YLL024C	YLR187W	0.7380
YLL024C	YLR189C	0.7380
YLL024C	YLR195C	0.7380
YLL024C	YLR209C	0.7380
YLL024C	YLR384C	0.7380
YLL024C	YLR386W	0.7380
YLL024C	YLR398C	0.7380
YLL024C	YLR410W	0.7380
YLL024C	YLR418C	0.7380
YLL024C	YLR442C	0.7380
YLL024C	YML062C	0.7380
YLL024C	YML088W	0.8953
YLL024C	YMR027W	0.7380
YLL024C	YMR047C	0.7380
YLL024C	YMR128W	0.7380
YLL024C	YMR129W	0.7380
YLL024C	YMR186W	0.9021
YLL024C	YMR229C	0.7380
YLL024C	YMR237W	0.7380
YLL024C	YMR273C	0.7380
YLL024C	YNL004W	0.7380
YLL024C	YNL007C	0.7380
YLL024C	YNL102W	0.7380
YLL024C	YNR053C	0.7380
YLL024C	YOL006C	0.7380
YLL024C	YOL021C	0.7380
YLL024C	YOL033W	0.7380
YLL024C	YOL145C	0.7380
YLL024C	YOR027W	0.9934
YLL024C	YOR043W	0.7380
YLL024C	YOR057W	0.7380
YLL024C	YOR090C	0.7380
YLL024C	YOR133W	0.7380

YLL024C	YOR191W	0.7380
YLL024C	YOR341W	0.7380
YLL024C	YPL012W	0.7380
YLL024C	YPL022W	0.7380
YLL024C	YPL104W	0.7380
YLL024C	YPL106C	0.9923
YLL024C	YPL110C	0.7380
YLL024C	YPL167C	0.7380
YLL024C	YPL181W	0.7380
YLL024C	YPL233W	0.8659
YLL024C	YPR010C	0.7380
YLL024C	YPR025C	0.7380
YLL024C	YPR135W	0.7380
YLL024C	YPR140W	0.7380
YLL024C	YPR144C	0.7380
YLL024C	YPR171W	0.7380
YLL026W	YLR418C	0.7380
YLL026W	YNL139C	0.7380
YLL026W	YOR027W	0.9249
YLL034C	YLR449W	0.7380
YLL034C	YMR242C	0.7380
YLL034C	YNL110C	0.8659
YLL034C	YOL077C	0.7380
YLL034C	YOR063W	0.7380
YLL034C	YOR206W	0.7380
YLL034C	YOR272W	0.7380
YLL034C	YOR341W	0.7380
YLL034C	YPL211W	0.7380
YLL036C	YLR117C	1.0000
YLL036C	YLR132C	0.8297
YLL036C	YLR147C	0.9583
YLL036C	YLR275W	0.9186
YLL036C	YLR424W	0.7380
YLL036C	YML049C	0.9583
YLL036C	YMR213W	1.0000
YLL036C	YMR240C	0.9583
YLL036C	YMR288W	0.9787
YLL036C	YNL245C	0.7380
YLL036C	YPL151C	0.9985
YLL036C	YPL213W	0.9920
YLL036C	YPR101W	1.0000
YLL036C	YPR178W	0.9555
YLL036C	YPR182W	0.9583
YLL039C	YLR032W	0.7543
YLL039C	YLR075W	0.7380
YLL039C	YLR079W	0.9249
YLL039C	YLR153C	0.7380

YLL039C	YLR175W	0.7380
YLL039C	YLR180W	0.9021
YLL039C	YLR342W	0.7380
YLL039C	YLR441C	0.7380
YLL039C	YLR448W	0.7380
YLL039C	YML063W	0.7380
YLL039C	YML097C	0.9980
YLL039C	YML123C	0.7380
YLL039C	YMR015C	0.7380
YLL039C	YMR210W	0.7380
YLL039C	YMR246W	0.7380
YLL039C	YMR276W	0.9924
YLL039C	YMR307W	0.7380
YLL039C	YNL014W	0.7380
YLL039C	YNL064C	0.9021
YLL039C	YNL103W	0.9968
YLL039C	YNL178W	0.7380
YLL039C	YNL209W	0.8087
YLL039C	YNL301C	0.7380
YLL039C	YNR006W	0.9973
YLL039C	YOL081W	0.8566
YLL039C	YOL122C	0.8566
YLL039C	YOR027W	0.9021
YLL039C	YOR151C	0.7380
YLL039C	YOR207C	0.7380
YLL039C	YOR341W	0.7380
YLL039C	YOR368W	0.9215
YLL039C	YOR369C	0.7380
YLL039C	YOR375C	0.9021
YLL039C	YPL096W	0.7380
YLL039C	YPL240C	0.8566
YLL039C	YPR010C	0.7380
YLL039C	YPR035W	0.7380
YLL039C	YPR074C	0.8087
YLL039C	YPR154W	0.6672
YLL040C	YMR078C	0.7380
YLL043W	YLR113W	0.9567
YLL045C	YLR075W	0.8566
YLL045C	YLR432W	0.7380
YLL045C	YNL061W	0.7380
YLL045C	YNL110C	0.7380
YLL045C	YOL041C	0.7380
YLL045C	YOL077C	0.7380
YLL045C	YOR272W	0.7380
YLL045C	YPL043W	0.7380
YLL045C	YPL211W	0.7380
YLL045C	YPR016C	0.7380

YLL049W	YLR423C	0.5774
YLL049W	YMR294W	0.9987
YLL049W	YNR069C	0.5774
YLL049W	YPL174C	0.9980
YLL050C	YLR429W	0.8521
YLL050C	YMR092C	0.9830
YLL050C	YNL079C	0.8953
YLL050C	YNL138W	0.7380
YLR002C	YLR449W	0.7380
YLR002C	YMR049C	0.7380
YLR002C	YNL061W	0.7380
YLR002C	YNL110C	0.7380
YLR002C	YOL077C	0.7380
YLR002C	YOR206W	0.9972
YLR002C	YOR243C	0.7380
YLR002C	YOR272W	0.9021
YLR002C	YOR294W	0.7380
YLR002C	YPR016C	0.9499
YLR005W	YPR056W	0.9983
YLR006C	YNR031C	0.9993
YLR008C	YNR017W	1.0000
YLR009W	YNL061W	0.8087
YLR009W	YNL110C	0.8087
YLR009W	YNR053C	0.9805
YLR009W	YPL093W	1.0000
YLR009W	YPR016C	0.8953
YLR015W	YPL138C	0.9999
YLR018C	YML031W	0.8603
YLR018C	YMR129W	0.8603
YLR021W	YPL144W	1.0000
YLR022C	YPL211W	0.9922
YLR025W	YLR181C	0.9322
YLR025W	YMR077C	0.9954
YLR025W	YMR154C	0.8179
YLR025W	YOR275C	0.9939
YLR025W	YPL084W	0.9992
YLR025W	YPR173C	0.9984
YLR026C	YLR078C	1.0000
YLR026C	YLR268W	1.0000
YLR026C	YML012W	0.9555
YLR026C	YML067C	0.9555
YLR026C	YML115C	0.9927
YLR026C	YMR197C	0.9996
YLR026C	YNL044W	0.8967
YLR026C	YOR327C	0.9282
YLR026C	YPL218W	0.9555
YLR028C	YMR120C	0.8028

YLR029C	YMR229C	0.7380
YLR029C	YOL041C	0.7380
YLR029C	YOL077C	0.7380
YLR031W	YMR124W	0.5774
YLR033W	YLR321C	0.9313
YLR033W	YLR357W	0.9953
YLR033W	YML127W	0.9648
YLR033W	YMR033W	0.9648
YLR033W	YMR091C	0.9953
YLR033W	YPL082C	0.8087
YLR033W	YPR034W	0.9820
YLR035C	YMR167W	0.9961
YLR035C	YOR261C	0.7380
YLR039C	YLR262C	0.9464
YLR039C	YOR361C	0.7380
YLR043C	YLR109W	0.8798
YLR043C	YPR167C	0.8798
YLR045C	YML085C	0.9440
YLR045C	YML124C	0.8929
YLR045C	YPL269W	0.9919
YLR052W	YNL059C	0.7380
YLR052W	YNL215W	0.7380
YLR052W	YOR141C	0.9021
YLR052W	YOR189W	0.8087
YLR052W	YPL129W	0.7380
YLR052W	YPL235W	0.8659
YLR055C	YMR223W	0.9936
YLR055C	YMR236W	0.9844
YLR055C	YOL148C	0.9993
YLR055C	YPL047W	0.9733
YLR055C	YPL254W	0.9948
YLR056W	YLR100W	0.9928
YLR056W	YML008C	0.9151
YLR056W	YNL280C	0.9151
YLR071C	YMR112C	0.9850
YLR071C	YNL236W	1.0000
YLR071C	YNR010W	0.9997
YLR071C	YOL051W	0.9999
YLR071C	YOL135C	0.9999
YLR071C	YOR151C	0.9834
YLR071C	YOR174W	0.9961
YLR071C	YPL042C	0.8659
YLR071C	YPL129W	0.8566
YLR071C	YPR070W	0.9994
YLR071C	YPR168W	0.9975
YLR074C	YLR106C	0.7380
YLR074C	YLR325C	0.7380

YLR074C	YNL002C	0.7380
YLR074C	YNL110C	0.7380
YLR074C	YNL182C	0.7380
YLR074C	YNR053C	0.8659
YLR074C	YOL127W	0.7380
YLR074C	YPL093W	0.9313
YLR074C	YPR016C	0.7380
YLR075W	YLR150W	0.8087
YLR075W	YLR180W	0.7380
YLR075W	YNL132W	0.7380
YLR075W	YPL220W	0.7380
YLR078C	YLR268W	0.9994
YLR079W	YLR113W	0.9758
YLR079W	YLR210W	0.7380
YLR079W	YLR361C	0.8655
YLR079W	YPL256C	0.8894
YLR079W	YPR119W	0.9685
YLR079W	YPR120C	0.9996
YLR085C	YML041C	0.8659
YLR085C	YOL012C	0.9478
YLR086W	YLR272C	0.8566
YLR090W	YPR189W	0.7380
YLR093C	YMR197C	0.9990
YLR093C	YOR106W	1.0000
YLR096W	YML006C	0.7380
YLR096W	YNL161W	0.7100
YLR100W	YML008C	0.9151
YLR100W	YMR015C	0.9762
YLR100W	YMR202W	0.9215
YLR100W	YNL280C	0.9151
YLR102C	YLR127C	0.8603
YLR102C	YMR001C	0.8206
YLR102C	YOR249C	0.9021
YLR103C	YLR274W	0.9944
YLR103C	YNL273W	0.9924
YLR103C	YPL153C	0.9919
YLR103C	YPR019W	0.9969
YLR105C	YPL083C	0.9071
YLR106C	YLR180W	0.7380
YLR106C	YNL110C	0.8659
YLR106C	YNL182C	0.8087
YLR106C	YNR053C	0.7380
YLR106C	YOR272W	0.9729
YLR106C	YPL043W	0.7380
YLR106C	YPL093W	0.7380
YLR113W	YLR138W	0.8655
YLR113W	YLR248W	1.0000

YLR113W	YMR172W	0.9746
YLR113W	YNL167C	0.9924
YLR113W	YOR208W	0.9992
YLR115W	YLR277C	0.9992
YLR115W	YMR061W	0.8659
YLR115W	YNL222W	0.9994
YLR115W	YNL317W	0.9993
YLR115W	YPR107C	0.9313
YLR117C	YLR147C	0.8087
YLR117C	YLR424W	0.9860
YLR117C	YML049C	0.9499
YLR117C	YMR213W	0.9998
YLR117C	YMR240C	0.9568
YLR117C	YMR288W	0.9384
YLR117C	YPL151C	0.9839
YLR117C	YPL213W	0.9285
YLR117C	YPR101W	0.9963
YLR119W	YPL065W	0.9993
YLR127C	YNL172W	0.9990
YLR127C	YOR249C	0.9813
YLR128W	YLR306W	0.9282
YLR129W	YLR222C	0.9313
YLR129W	YLR409C	0.8659
YLR129W	YNR054C	0.8087
YLR129W	YPR144C	0.8659
YLR131C	YNL161W	0.9746
YLR135W	YPL022W	0.9619
YLR138W	YML132W	0.9191
YLR141W	YMR270C	0.9834
YLR145W	YNL221C	0.9598
YLR145W	YNL282W	0.9598
YLR146C	YPR069C	0.9128
YLR147C	YLR275W	0.7380
YLR147C	YLR298C	0.7380
YLR147C	YML046W	0.7380
YLR147C	YML049C	0.7380
YLR147C	YMR125W	0.9313
YLR147C	YMR213W	0.8659
YLR147C	YMR240C	0.9313
YLR147C	YOR159C	0.7380
YLR147C	YOR308C	0.8087
YLR147C	YPL213W	0.9021
YLR147C	YPR101W	0.7380
YLR147C	YPR182W	0.8659
YLR148W	YLR396C	1.0000
YLR148W	YMR231W	0.9995
YLR148W	YOR106W	0.9983

YLR148W	YPL045W	0.9946
YLR150W	YNR051C	0.7380
YLR150W	YOL127W	0.9285
YLR153C	YPL106C	0.8659
YLR166C	YNL272C	0.9249
YLR166C	YPR055W	1.0000
YLR170C	YPL259C	0.9808
YLR170C	YPR029C	0.9782
YLR172C	YOR133W	0.6147
YLR175W	YLR197W	0.9648
YLR175W	YMR229C	0.7380
YLR175W	YMR290C	0.8659
YLR175W	YMR309C	0.7380
YLR175W	YMR310C	0.8659
YLR175W	YNL124W	0.9980
YLR175W	YNL308C	0.7380
YLR175W	YPL012W	0.8659
YLR175W	YPL043W	0.7380
YLR175W	YPL240C	0.7380
YLR178C	YMR297W	0.9341
YLR178C	YOL081W	0.9922
YLR180W	YMR012W	0.7380
YLR180W	YOR151C	0.7380
YLR181C	YPR173C	1.0000
YLR182W	YML069W	0.9215
YLR182W	YNL309W	0.9790
YLR182W	YOR083W	0.9960
YLR182W	YPL153C	0.9139
YLR182W	YPL204W	0.8655
YLR186W	YOR310C	0.8659
YLR186W	YPR144C	0.8659
YLR190W	YMR117C	0.7253
YLR190W	YPL031C	0.7380
YLR191W	YML035C	0.5774
YLR191W	YMR026C	0.9989
YLR191W	YNL214W	0.9151
YLR191W	YOL044W	0.9124
YLR192C	YMR146C	0.8521
YLR192C	YMR309C	0.9902
YLR192C	YNL244C	0.8566
YLR192C	YOR361C	0.9999
YLR192C	YPR041W	0.9624
YLR196W	YLR449W	0.7380
YLR196W	YOL041C	0.9313
YLR196W	YOL077C	0.8990
YLR196W	YOR206W	0.6147
YLR196W	YPL126W	0.7380

YLR196W	YPL207W	0.7380
YLR197W	YNL061W	0.7380
YLR197W	YOR078W	0.9313
YLR197W	YOR310C	0.9997
YLR197W	YPL043W	0.8659
YLR197W	YPR137W	0.8659
YLR200W	YML094W	0.9875
YLR200W	YNL153C	0.9735
YLR203C	YML129C	1.0000
YLR207W	YML029W	0.8953
YLR207W	YOL013C	0.9977
YLR208W	YML130C	0.7380
YLR208W	YPL085W	0.9816
YLR208W	YPR181C	0.9797
YLR212C	YNL126W	1.0000
YLR215C	YNL116W	0.8179
YLR216C	YMR186W	0.9962
YLR216C	YNL330C	0.9138
YLR216C	YOR027W	0.9790
YLR216C	YPL240C	0.9999
YLR221C	YOL144W	0.8659
YLR222C	YLR409C	0.9820
YLR222C	YNL075W	0.7380
YLR222C	YOR078W	0.7380
YLR222C	YOR310C	0.8087
YLR222C	YPR137W	0.8659
YLR222C	YPR144C	0.8659
YLR223C	YPR104C	0.9929
YLR226W	YMR125W	0.7380
YLR226W	YPR161C	1.0000
YLR229C	YNL271C	0.9966
YLR229C	YNL298W	1.0000
YLR229C	YOL113W	0.9667
YLR229C	YOR127W	0.9972
YLR229C	YPL115C	0.9667
YLR229C	YPL161C	0.9476
YLR229C	YPL242C	0.9686
YLR233C	YLR318W	0.9215
YLR234W	YMR190C	1.0000
YLR234W	YPL024W	1.0000
YLR238W	YMR052W	0.9710
YLR240W	YLR360W	0.9266
YLR240W	YPL120W	0.9860
YLR245C	YLR291C	0.5774
YLR245C	YNL189W	0.5774
YLR245C	YPL070W	0.5774
YLR248W	YMR243C	0.9099

YLR248W	YOR018W	0.7347
YLR249W	YMR186W	0.7380
YLR249W	YPR080W	0.9215
YLR249W	YPR180W	0.7380
YLR254C	YOR269W	0.9230
YLR256W	YPL240C	0.9533
YLR257W	YLR345W	0.5107
YLR258W	YPL031C	0.9632
YLR259C	YNL055C	0.7380
YLR259C	YNL085W	0.8659
YLR262C	YNL044W	0.9099
YLR262C	YNL293W	0.7696
YLR262C	YOR370C	0.5107
YLR263W	YOR351C	0.9990
YLR264W	YOR310C	0.7380
YLR265C	YMR106C	0.9215
YLR265C	YMR284W	0.9215
YLR268W	YMR183C	0.6000
YLR268W	YNL287W	0.9191
YLR268W	YOR075W	0.9598
YLR268W	YPL218W	0.9875
YLR268W	YPL232W	0.8179
YLR268W	YPR181C	0.9768
YLR270W	YOR173W	0.9569
YLR274W	YNL273W	0.8087
YLR274W	YPR019W	0.9696
YLR275W	YLR298C	0.9313
YLR275W	YMR125W	0.9313
YLR275W	YMR213W	0.8659
YLR275W	YMR240C	0.7380
YLR275W	YNL147W	0.9313
YLR275W	YOR159C	0.8659
YLR275W	YPL178W	0.8659
YLR275W	YPL213W	0.9813
YLR275W	YPR057W	0.7380
YLR275W	YPR178W	0.7380
YLR275W	YPR182W	0.9280
YLR276C	YMR308C	0.8659
YLR276C	YPL043W	0.8659
YLR277C	YMR061W	0.8659
YLR277C	YNL222W	0.9925
YLR277C	YNL317W	0.9998
YLR277C	YPR107C	0.9986
YLR287C	YLR340W	0.7380
YLR288C	YMR159C	0.5774
YLR288C	YOR368W	1.0000
YLR288C	YPL194W	0.9997

YLR291C	YNL244C	0.9249
YLR291C	YOR260W	1.0000
YLR291C	YOR284W	0.5774
YLR291C	YPL070W	0.7253
YLR291C	YPL237W	0.9961
YLR292C	YOR254C	0.9999
YLR292C	YPL094C	0.9860
YLR293C	YLR347C	1.0000
YLR293C	YMR235C	0.8742
YLR293C	YMR308C	0.9990
YLR293C	YNL189W	0.9282
YLR293C	YOR160W	0.9341
YLR293C	YPL125W	0.9850
YLR298C	YML046W	0.9313
YLR298C	YMR125W	0.9648
YLR309C	YOR216C	0.6000
YLR310C	YNL098C	1.0000
YLR310C	YOR101W	0.9769
YLR310C	YPL204W	0.7380
YLR312W-A	YMR024W	0.7380
YLR313C	YLR362W	0.8965
YLR314C	YMR055C	0.9138
YLR314C	YOR156C	0.8456
YLR314C	YPL153C	0.8087
YLR315W	YPR046W	0.7570
YLR319C	YLR362W	0.8965
YLR320W	YPR135W	0.9710
YLR320W	YPR164W	0.9215
YLR321C	YLR345W	0.5774
YLR321C	YLR357W	0.9985
YLR321C	YML127W	0.9313
YLR321C	YMR033W	0.8659
YLR321C	YMR091C	0.9820
YLR321C	YPR034W	0.9313
YLR328W	YML064C	0.5774
YLR330W	YMR237W	0.9969
YLR330W	YOR299W	0.9852
YLR335W	YLR347C	1.0000
YLR335W	YMR047C	0.9186
YLR335W	YNL189W	1.0000
YLR336C	YPL268W	0.9099
YLR337C	YMR032W	0.9850
YLR337C	YMR109W	1.0000
YLR337C	YOR181W	1.0000
YLR340W	YOL039W	0.9871
YLR340W	YPL220W	0.8566
YLR342W	YML130C	0.7380

YLR342W	YPL094C	0.7380
YLR342W	YPR165W	0.9541
YLR347C	YMR001C	0.8087
YLR347C	YMR047C	1.0000
YLR347C	YMR125W	0.7380
YLR347C	YMR273C	0.7380
YLR347C	YMR308C	0.9808
YLR347C	YNL139C	0.7380
YLR347C	YNL189W	1.0000
YLR347C	YOR001W	0.9634
YLR347C	YOR098C	1.0000
YLR347C	YPL020C	0.9895
YLR347C	YPL106C	0.7380
YLR347C	YPL153C	0.7380
YLR347C	YPL178W	0.9313
YLR347C	YPR093C	0.9107
YLR350W	YMR296C	0.9918
YLR353W	YOR301W	0.9215
YLR357W	YML127W	0.9972
YLR357W	YMR033W	0.9996
YLR357W	YMR091C	0.9974
YLR357W	YMR224C	0.9747
YLR357W	YPR034W	0.9992
YLR360W	YPL120W	0.9499
YLR362W	YMR186W	0.9249
YLR362W	YPL240C	0.9962
YLR367W	YNL207W	0.7380
YLR367W	YOL139C	0.7380
YLR367W	YOR056C	0.7380
YLR367W	YOR310C	0.7380
YLR369W	YOR232W	0.9921
YLR370C	YMR109W	0.7891
YLR370C	YNR035C	0.9984
YLR370C	YOR181W	0.6147
YLR373C	YPL128C	0.9313
YLR377C	YNL189W	0.5774
YLR377C	YPL180W	0.8953
YLR378C	YMR149W	0.7718
YLR378C	YOR254C	0.9997
YLR378C	YPL094C	0.9779
YLR381W	YPL018W	0.9539
YLR381W	YPR046W	0.8953
YLR383W	YOL034W	0.9489
YLR384C	YMR312W	0.9915
YLR384C	YNL272C	0.9138
YLR384C	YPL086C	1.0000
YLR384C	YPL101W	0.9988

YLR384C	YPL204W	0.9464
YLR385C	YLR399C	0.7380
YLR385C	YML041C	0.7380
YLR385C	YNL107W	0.9266
YLR386W	YNL054W	0.9099
YLR386W	YNL325C	0.9995
YLR396C	YML001W	0.9924
YLR396C	YMR197C	0.9555
YLR396C	YMR231W	1.0000
YLR396C	YOR106W	0.9999
YLR396C	YPL045W	1.0000
YLR398C	YPR189W	0.9998
YLR399C	YMR227C	0.9956
YLR399C	YNL030W	0.9099
YLR399C	YNL107W	0.9464
YLR399C	YOL012C	0.9800
YLR399C	YPL235W	0.8566
YLR403W	YNL006W	0.9215
YLR403W	YOR370C	0.9698
YLR407W	YOR061W	0.7380
YLR409C	YMR093W	0.8659
YLR409C	YOR310C	0.7380
YLR410W	YML069W	0.7380
YLR410W	YPL031C	0.6042
YLR417W	YMR077C	0.9955
YLR417W	YPL002C	1.0000
YLR417W	YPL065W	0.9954
YLR418C	YML069W	0.9634
YLR418C	YMR186W	0.8659
YLR418C	YOL051W	0.9762
YLR418C	YOL145C	1.0000
YLR418C	YOR039W	0.7380
YLR418C	YOR061W	0.8659
YLR418C	YOR123C	0.9998
YLR418C	YPR086W	0.9518
YLR421C	YNL073W	0.8965
YLR421C	YOR117W	0.7380
YLR421C	YOR259C	0.7380
YLR421C	YOR261C	0.8659
YLR421C	YPR108W	0.7380
YLR423C	YML064C	0.5774
YLR423C	YNL182C	0.7837
YLR423C	YOR158W	0.5774
YLR423C	YPL077C	0.5774
YLR423C	YPL166W	0.9999
YLR423C	YPR049C	0.9779
YLR423C	YPR185W	0.9747

YLR424W	YMR213W	0.8566
YLR424W	YPL151C	0.8659
YLR424W	YPR101W	0.9266
YLR424W	YPR182W	0.7380
YLR425W	YPL066W	0.8659
YLR429W	YPL032C	0.5774
YLR430W	YMR125W	0.7380
YLR430W	YMR239C	0.9099
YLR430W	YNL251C	0.9813
YLR430W	YPL190C	0.9685
YLR432W	YML056C	0.9828
YLR432W	YPL131W	0.7380
YLR432W	YPL198W	0.7380
YLR432W	YPR175W	0.7380
YLR433C	YML057W	0.9574
YLR433C	YNL047C	0.9327
YLR433C	YOR324C	0.8965
YLR436C	YMR304W	0.9869
YLR438C-A	YMR268C	0.9974
YLR438C-A	YNL147W	0.9991
YLR438C-A	YOR159C	0.7909
YLR438C-A	YPR178W	0.9285
YLR438W	YPL204W	0.5779
YLR439W	YML025C	0.7380
YLR439W	YNL284C	0.8659
YLR440C	YNL258C	0.9956
YLR440C	YOR075W	0.9266
YLR442C	YNL030W	1.0000
YLR442C	YNL031C	1.0000
YLR442C	YNL216W	1.0000
YLR447C	YOR270C	1.0000
YLR448W	YMR061W	0.7380
YLR449W	YMR049C	0.8659
YLR449W	YMR242C	0.7380
YLR449W	YNL061W	0.9313
YLR449W	YOR063W	0.7380
YLR449W	YOR206W	0.8028
YLR449W	YPL093W	0.9021
YLR449W	YPL131W	0.7380
YLR449W	YPL146C	0.8087
YLR450W	YMR022W	0.8714
YLR450W	YOL013C	0.9640
YLR453C	YNL216W	0.7909
YML001W	YNL263C	0.9138
YML001W	YPL045W	0.9215
YML007W	YML028W	0.9640
YML007W	YMR308C	0.9948

YML007W	YNL236W	0.7380
YML007W	YOR244W	0.9313
YML007W	YPR070W	0.8659
YML008C	YMR015C	0.9151
YML008C	YMR202W	0.9762
YML008C	YNL280C	0.9151
YML009C	YNL284C	0.7380
YML010W	YML069W	0.9266
YML010W	YML088W	0.8998
YML010W	YNL201C	0.9996
YML010W	YOL005C	0.9499
YML010W	YOR151C	0.9499
YML010W	YOR224C	0.7380
YML010W	YOR341W	0.8566
YML010W	YPR010C	0.8087
YML010W	YPR187W	0.8659
YML013W	YOL013C	0.9707
YML014W	YNR046W	0.9970
YML015C	YML098W	0.9960
YML015C	YML114C	0.9266
YML015C	YMR005W	0.9902
YML015C	YMR227C	0.9808
YML015C	YMR236W	0.9950
YML015C	YPL011C	0.9950
YML015C	YPL129W	0.7380
YML016C	YOR054C	0.9711
YML019W	YMR149W	0.9555
YML019W	YOR085W	0.9215
YML019W	YOR103C	0.9962
YML025C	YMR024W	0.7380
YML025C	YMR193W	0.7380
YML025C	YNL005C	0.7380
YML025C	YNL177C	0.7380
YML025C	YNL252C	0.7380
YML027W	YMR043W	0.9790
YML028W	YPL106C	0.7380
YML029W	YOL013C	0.9890
YML031W	YMR129W	0.9887
YML031W	YMR153W	0.8867
YML032C	YNL312W	0.9934
YML041C	YNL107W	0.7380
YML041C	YOL012C	0.9945
YML041C	YPL235W	0.8659
YML046W	YMR125W	0.7380
YML046W	YPL213W	0.8087
YML046W	YPR182W	0.8659
YML049C	YMR125W	0.8659

YML049C	YMR213W	0.9313
YML049C	YMR240C	0.9904
YML049C	YMR288W	0.9933
YML049C	YOR159C	0.7380
YML049C	YOR319W	0.9743
YML049C	YPL178W	0.7380
YML049C	YPL213W	0.9634
YML049C	YPR094W	0.9478
YML051W	YPL248C	1.0000
YML055W	YPL094C	0.5107
YML056C	YOL139C	0.8659
YML056C	YPL198W	0.7380
YML057W	YNL047C	0.9440
YML057W	YOR324C	0.9099
YML058W	YPL153C	0.8655
YML062C	YNL004W	0.9499
YML062C	YNL139C	1.0000
YML062C	YNL253W	0.9624
YML063W	YOL120C	0.7380
YML063W	YPL012W	0.7380
YML064C	YMR055C	0.9996
YML064C	YOR020C	0.5774
YML064C	YOR284W	0.5774
YML064C	YPL049C	0.5774
YML065W	YNL030W	0.8566
YML065W	YNL031C	0.9598
YML065W	YNL069C	0.7380
YML065W	YNL261W	1.0000
YML065W	YOR310C	0.8087
YML065W	YPL001W	0.9634
YML065W	YPL093W	0.8087
YML065W	YPL127C	0.8566
YML065W	YPR162C	1.0000
YML067C	YML130C	0.7380
YML069W	YNL030W	0.7380
YML069W	YOL054W	0.7380
YML069W	YOL145C	0.9904
YML069W	YOR039W	0.9313
YML069W	YOR061W	0.9313
YML069W	YOR123C	0.9990
YML069W	YPR019W	0.8953
YML071C	YNL041C	0.9966
YML071C	YNL051W	0.9973
YML071C	YPR105C	0.9904
YML074C	YOR340C	0.9555
YML074C	YPR073C	0.9899
YML077W	YMR218C	0.9892

YML077W	YOR115C	0.9700
YML085C	YML124C	0.7380
YML085C	YNL148C	0.9099
YML085C	YOL111C	0.9215
YML086C	YNL121C	0.7826
YML088W	YOR151C	0.8998
YML088W	YPL231W	0.8998
YML091C	YNL118C	0.9157
YML091C	YPL204W	0.9499
YML092C	YMR308C	0.7380
YML092C	YMR314W	0.9648
YML092C	YOL038W	0.9820
YML092C	YOR157C	0.9568
YML092C	YOR362C	0.9624
YML092C	YPR103W	0.9891
YML094W	YNL153C	0.9266
YML094W	YOL076W	0.7380
YML095C	YMR201C	0.9780
YML095C	YOL090W	0.9710
YML095C	YPL022W	1.0000
YML097C	YOR089C	0.9567
YML098W	YML114C	0.9624
YML098W	YMR005W	0.9950
YML098W	YMR227C	0.9648
YML098W	YMR236W	0.9820
YML098W	YPL011C	0.9313
YML098W	YPL129W	0.8990
YML098W	YPR072W	0.9138
YML099C	YMR042W	0.9970
YML099C	YMR043W	0.9907
YML100W	YMR261C	0.7380
YML102W	YNL031C	0.9215
YML102W	YNL206C	0.9215
YML102W	YPR018W	0.9986
YML103C	YMR047C	0.9869
YML103C	YOR098C	0.9932
YML105C	YPL210C	0.9940
YML105C	YPL243W	0.9983
YML105C	YPR088C	0.9983
YML109W	YMR273C	0.7837
YML114C	YMR005W	0.7380
YML114C	YMR227C	0.8659
YML114C	YMR236W	0.7380
YML114C	YPL011C	0.9266
YML114C	YPL129W	0.9648
YML115C	YPL050C	1.0000
YML117W	YNL251C	0.7380

YML117W	YPL178W	0.7380
YML121W	YNL113W	0.9843
YML121W	YPL180W	0.9099
YML124C	YMR012W	0.7380
YML124C	YNL148C	0.9747
YML127W	YMR033W	0.9313
YML127W	YMR091C	0.9820
YML127W	YPR034W	0.8659
YMR001C	YMR036C	0.7696
YMR001C	YNL068C	0.8714
YMR001C	YNL189W	0.8087
YMR001C	YOR195W	0.8655
YMR001C	YOR373W	0.7696
YMR001C	YPL153C	0.9623
YMR001C	YPL255W	0.9138
YMR001C	YPR007C	0.9950
YMR005W	YMR227C	0.8659
YMR005W	YMR236W	0.9984
YMR005W	YNL216W	0.8986
YMR005W	YPL011C	0.9950
YMR005W	YPL129W	0.7380
YMR005W	YPL248C	0.9187
YMR017W	YOR327C	0.9249
YMR017W	YPL232W	0.9944
YMR022W	YMR264W	0.9875
YMR024W	YNL284C	0.8659
YMR028W	YPL152W	0.9215
YMR028W	YPR040W	0.9747
YMR031C	YPL004C	0.9342
YMR032W	YNL152W	0.9995
YMR032W	YOR181W	0.8640
YMR033W	YMR091C	0.9950
YMR033W	YNR023W	0.7380
YMR033W	YOR290C	0.9994
YMR033W	YPL016W	0.9313
YMR033W	YPL129W	0.9624
YMR033W	YPR034W	0.9640
YMR036C	YOR061W	0.7543
YMR036C	YPL204W	0.7543
YMR042W	YMR043W	0.9907
YMR043W	YNL068C	0.9986
YMR047C	YMR080C	0.9099
YMR047C	YMR308C	0.9881
YMR047C	YNL189W	0.9186
YMR047C	YOR112W	0.9512
YMR047C	YPL125W	0.9186
YMR047C	YPL169C	0.9999

YMR048W	YNL273W	1.0000
YMR048W	YPR019W	0.8953
YMR049C	YMR229C	0.8659
YMR049C	YMR290C	0.9313
YMR049C	YNL002C	0.9313
YMR049C	YNL061W	0.9820
YMR049C	YNL110C	0.9908
YMR049C	YOL077C	0.9648
YMR049C	YOR063W	0.7380
YMR049C	YOR206W	0.9648
YMR049C	YOR272W	1.0000
YMR049C	YPL043W	0.8659
YMR049C	YPL093W	0.9813
YMR049C	YPR016C	0.9743
YMR052W	YNL127W	0.8353
YMR054W	YPR036W	0.7891
YMR055C	YOR373W	0.9690
YMR061W	YNL317W	0.9640
YMR061W	YOR250C	0.9648
YMR064W	YOL123W	0.7380
YMR066W	YPL106C	0.7380
YMR068W	YNL006W	0.8953
YMR068W	YNL047C	0.9512
YMR068W	YOL078W	0.9215
YMR072W	YOL004W	0.7380
YMR072W	YPL082C	0.7380
YMR075W	YNL330C	0.9998
YMR075W	YOL004W	0.9993
YMR075W	YPL082C	0.8953
YMR075W	YPL139C	0.9964
YMR075W	YPR023C	0.9987
YMR077C	YPL002C	0.9980
YMR077C	YPL065W	0.9947
YMR077C	YPR173C	0.9999
YMR078C	YNL262W	0.7380
YMR078C	YNL290W	0.9999
YMR078C	YOL094C	0.9999
YMR078C	YPR175W	0.7380
YMR080C	YNL112W	0.8965
YMR080C	YOL123W	0.9790
YMR080C	YOL149W	0.9099
YMR080C	YPL106C	0.7380
YMR086W	YPL004C	0.9342
YMR091C	YPL082C	0.8087
YMR091C	YPR034W	0.9950
YMR092C	YPL106C	0.7380
YMR093W	YOR078W	0.7380

YMR093W	YOR310C	0.9021
YMR093W	YPL012W	0.8659
YMR093W	YPL126W	0.9950
YMR093W	YPR137W	0.8659
YMR093W	YPR144C	0.8659
YMR094W	YMR168C	0.9925
YMR094W	YOR057W	0.9799
YMR095C	YMR096W	0.9846
YMR095C	YNL333W	0.5774
YMR096W	YNL333W	0.8640
YMR104C	YMR109W	0.9138
YMR106C	YMR284W	0.9945
YMR109W	YOR035C	0.9922
YMR109W	YOR181W	0.9996
YMR109W	YOR247W	0.5774
YMR112C	YNR010W	0.9887
YMR112C	YOL135C	0.9624
YMR112C	YPR070W	0.7380
YMR112C	YPR168W	0.7380
YMR116C	YNL308C	0.7380
YMR116C	YPR189W	0.7380
YMR117C	YOL069W	1.0000
YMR120C	YNR034W	0.6147
YMR125W	YMR240C	0.8659
YMR125W	YNL139C	0.7380
YMR125W	YNL189W	0.9820
YMR125W	YNL251C	0.9869
YMR125W	YOL139C	0.9313
YMR125W	YPL082C	0.9079
YMR125W	YPL151C	0.7380
YMR125W	YPL169C	0.7380
YMR125W	YPL178W	1.0000
YMR125W	YPL190C	0.9648
YMR125W	YPR057W	0.7380
YMR125W	YPR161C	0.8659
YMR125W	YPR182W	0.7380
YMR127C	YNL030W	0.8818
YMR127C	YOR213C	0.9998
YMR127C	YPR018W	0.9099
YMR128W	YMR229C	0.7380
YMR128W	YNL097C	0.7380
YMR128W	YOL010W	0.8659
YMR128W	YOR078W	0.7380
YMR128W	YOR310C	0.8087
YMR128W	YPL139C	0.7380
YMR128W	YPR137W	0.8659
YMR128W	YPR144C	0.8659

YMR129W	YPL106C	0.7380
YMR131C	YOR063W	0.8566
YMR139W	YNL078W	0.5774
YMR139W	YNL199C	0.7380
YMR139W	YOL061W	0.8353
YMR146C	YMR309C	1.0000
YMR146C	YNL062C	0.9215
YMR146C	YNL244C	0.9995
YMR146C	YOR361C	1.0000
YMR146C	YPL105C	0.6672
YMR146C	YPR041W	1.0000
YMR149W	YOR085W	0.9555
YMR153W	YMR308C	1.0000
YMR153W	YPL020C	0.9249
YMR153W	YPL204W	0.8655
YMR158W	YPL013C	0.7380
YMR159C	YPL149W	1.0000
YMR163C	YOR326W	0.9975
YMR165C	YPL031C	0.6042
YMR167W	YMR190C	0.9990
YMR167W	YNL082W	1.0000
YMR167W	YOL043C	0.9970
YMR167W	YOL090W	0.9886
YMR167W	YOR033C	0.9999
YMR167W	YPL164C	0.9997
YMR172W	YOR039W	0.9648
YMR172W	YOR061W	0.8659
YMR183C	YNR049C	0.9762
YMR183C	YOR106W	0.6000
YMR183C	YOR327C	0.9215
YMR183C	YPL232W	0.9249
YMR186W	YNL262W	0.7380
YMR186W	YNL281W	0.8353
YMR186W	YOR027W	1.0000
YMR186W	YOR057W	0.9892
YMR186W	YPL240C	0.9313
YMR186W	YPR181C	0.8659
YMR190C	YMR224C	0.9780
YMR190C	YPL024W	0.9995
YMR190C	YPL153C	0.9690
YMR191W	YPR108W	0.7380
YMR192W	YPL249C	0.9999
YMR192W	YPL249C-A	0.7380
YMR192W	YPR154W	0.5774
YMR193W	YNL252C	0.7380
YMR193W	YNL284C	0.7380
YMR194W	YNL132W	0.7380

YMR197C	YOL018C	0.9790
YMR197C	YOR036W	0.9790
YMR197C	YOR106W	1.0000
YMR197C	YOR327C	0.9282
YMR198W	YPR141C	0.9993
YMR199W	YPL031C	0.9138
YMR201C	YOL090W	0.9099
YMR201C	YPL022W	0.9991
YMR202W	YNL280C	0.9928
YMR203W	YNL055C	0.9773
YMR203W	YNL121C	0.9982
YMR203W	YNL131W	1.0000
YMR203W	YNR017W	0.9980
YMR203W	YPL063W	0.8521
YMR203W	YPR133W-A	0.9191
YMR213W	YMR288W	0.9313
YMR213W	YPL151C	0.9820
YMR213W	YPL213W	0.8087
YMR213W	YPR101W	0.9820
YMR216C	YNL004W	0.7543
YMR218C	YOR115C	0.8566
YMR223W	YMR236W	0.9726
YMR223W	YOL148C	0.9726
YMR223W	YPL047W	1.0000
YMR223W	YPL254W	0.9948
YMR224C	YNL250W	1.0000
YMR225C	YNL284C	0.7380
YMR227C	YMR236W	0.9648
YMR227C	YPL011C	0.9891
YMR227C	YPL129W	0.7380
YMR229C	YMR290C	0.8659
YMR229C	YNL061W	0.8659
YMR229C	YNL110C	0.7380
YMR229C	YNL175C	0.8659
YMR229C	YNR051C	0.7380
YMR229C	YOL041C	0.8659
YMR229C	YOL077C	0.8990
YMR229C	YOR078W	0.7380
YMR229C	YOR206W	0.9735
YMR229C	YOR272W	0.7380
YMR229C	YOR310C	0.9021
YMR229C	YPL043W	0.7380
YMR229C	YPL126W	0.8659
YMR229C	YPR137W	0.7380
YMR229C	YPR144C	0.7380
YMR231W	YPL045W	0.9970
YMR233W	YOL006C	0.5774

YMR236W	YOL148C	0.9891
YMR236W	YPL011C	0.9820
YMR236W	YPL047W	0.9285
YMR236W	YPL129W	0.7380
YMR236W	YPL254W	0.9787
YMR239C	YNL189W	0.6672
YMR239C	YNL251C	0.8087
YMR240C	YMR288W	0.9928
YMR240C	YOR319W	1.0000
YMR240C	YPL151C	0.8659
YMR240C	YPL213W	0.9869
YMR240C	YPR094W	0.9478
YMR242C	YNL110C	0.7380
YMR242C	YOL041C	0.7380
YMR242C	YOL077C	0.7380
YMR242C	YOR206W	0.7380
YMR242C	YPL093W	0.7380
YMR255W	YMR273C	0.9138
YMR255W	YOR046C	0.9858
YMR263W	YNL097C	0.9985
YMR263W	YNL330C	1.0000
YMR263W	YOL004W	0.9982
YMR263W	YPL139C	0.9743
YMR263W	YPL181W	0.9904
YMR268C	YNL147W	0.9937
YMR273C	YOR046C	0.9138
YMR288W	YNL286W	0.8297
YMR288W	YOR159C	0.7380
YMR288W	YPL213W	0.9499
YMR288W	YPR094W	0.8603
YMR290C	YNL061W	0.8659
YMR290C	YNL110C	0.9313
YMR290C	YNL308C	0.7380
YMR290C	YOL041C	0.7380
YMR290C	YOL077C	0.7380
YMR290C	YOR206W	0.7380
YMR290C	YOR272W	0.9021
YMR290C	YPL043W	0.9313
YMR290C	YPL093W	0.9634
YMR290C	YPL211W	0.7380
YMR290C	YPL220W	0.7380
YMR290C	YPR016C	0.9499
YMR294W	YOL069W	0.5774
YMR294W	YPL174C	0.9974
YMR308C	YMR310C	0.7380
YMR308C	YMR314W	0.7380
YMR308C	YNL031C	0.9616

YMR308C	YNL189W	0.9313
YMR308C	YOL038W	0.7380
YMR308C	YOL108C	0.7380
YMR308C	YOR098C	0.9734
YMR308C	YOR117W	0.7380
YMR308C	YOR185C	0.7570
YMR308C	YPL012W	0.7380
YMR308C	YPL020C	0.9993
YMR308C	YPR093C	0.9107
YMR309C	YNL244C	1.0000
YMR309C	YNR051C	0.7380
YMR309C	YOR039W	0.7380
YMR309C	YOR204W	0.7380
YMR309C	YOR361C	1.0000
YMR309C	YPR041W	1.0000
YMR311C	YPL031C	0.9027
YMR312W	YPL086C	0.9696
YMR312W	YPL101W	0.9537
YMR314W	YOL038W	0.9313
YMR314W	YOR157C	0.7380
YMR314W	YOR261C	0.9624
YMR314W	YOR362C	0.9879
YMR314W	YPR103W	0.9891
YNL002C	YNL110C	0.9313
YNL002C	YNR053C	0.8087
YNL002C	YOL077C	0.7380
YNL002C	YOR206W	0.7380
YNL002C	YOR272W	0.9021
YNL002C	YPL043W	0.8659
YNL002C	YPL093W	0.9285
YNL002C	YPR016C	0.9499
YNL004W	YNL139C	0.9499
YNL004W	YNL253W	0.8087
YNL004W	YPR161C	0.7380
YNL005C	YNL252C	0.7380
YNL005C	YNL284C	0.8659
YNL006W	YOL078W	0.9707
YNL007C	YPL106C	0.7380
YNL014W	YOR117W	0.9186
YNL020C	YNL243W	0.8353
YNL021W	YOL090W	0.7380
YNL021W	YPR179C	0.9974
YNL023C	YNL135C	0.9852
YNL025C	YNL094W	0.5774
YNL025C	YNL236W	0.7380
YNL025C	YPL031C	0.7909
YNL025C	YPL042C	1.0000

YNL025C	YPL248C	0.8456
YNL027W	YPL031C	0.6042
YNL027W	YPL204W	0.8963
YNL030W	YNL031C	0.9896
YNL030W	YNL206C	0.9616
YNL030W	YNL246W	0.9249
YNL030W	YOL012C	0.9478
YNL030W	YOL054W	0.7380
YNL030W	YOR244W	0.9620
YNL030W	YOR304W	0.8818
YNL030W	YPL001W	0.9794
YNL031C	YNL097C	0.9985
YNL031C	YNL206C	0.9970
YNL031C	YNL246W	0.9249
YNL031C	YOL012C	0.8603
YNL031C	YOL054W	0.7380
YNL031C	YOR064C	1.0000
YNL031C	YOR244W	0.7696
YNL031C	YPL001W	0.9266
YNL031C	YPL138C	0.9991
YNL031C	YPL153C	0.8655
YNL031C	YPL181W	0.9947
YNL031C	YPR018W	0.9790
YNL032W	YNL056W	0.7253
YNL032W	YNL099C	0.9851
YNL037C	YOR136W	1.0000
YNL041C	YNL051W	0.9266
YNL041C	YPR105C	0.7380
YNL044W	YNL263C	0.5774
YNL044W	YNL304W	0.9099
YNL049C	YPL085W	0.7380
YNL049C	YPR181C	0.9985
YNL051W	YPR105C	0.9938
YNL055C	YNL085W	0.7380
YNL056W	YNL099C	0.9811
YNL059C	YNL215W	0.7380
YNL059C	YOR141C	0.9499
YNL059C	YPL129W	0.8659
YNL059C	YPL235W	0.9499
YNL061W	YNL110C	0.7380
YNL061W	YNR051C	0.7380
YNL061W	YOL077C	0.8659
YNL061W	YOR063W	0.7380
YNL061W	YOR206W	0.9313
YNL061W	YOR272W	0.8659
YNL061W	YOR294W	0.7380
YNL061W	YPL043W	0.8659

YNL061W	YPL093W	0.9813
YNL061W	YPL131W	0.7380
YNL061W	YPL198W	0.7380
YNL061W	YPL211W	0.9820
YNL061W	YPL217C	0.7380
YNL061W	YPR016C	0.9743
YNL062C	YNL244C	0.9215
YNL062C	YOR361C	0.9780
YNL064C	YNL073W	0.7380
YNL064C	YNL247W	0.7380
YNL064C	YOL006C	0.7380
YNL064C	YOR361C	0.7380
YNL064C	YPL106C	0.7380
YNL064C	YPL240C	0.9710
YNL068C	YOR372C	0.9941
YNL069C	YNL178W	0.8566
YNL073W	YPL106C	0.7380
YNL075W	YPL126W	0.7380
YNL075W	YPR137W	0.8659
YNL078W	YOL070C	0.9433
YNL082W	YOL090W	0.9138
YNL084C	YOR181W	0.8028
YNL084C	YOR329C	0.9099
YNL090W	YPL161C	0.7909
YNL091W	YNL288W	0.5107
YNL093W	YOR089C	0.7380
YNL093W	YOR370C	0.6672
YNL094W	YPR154W	0.5774
YNL097C	YNL330C	1.0000
YNL097C	YOL004W	0.9990
YNL097C	YPL139C	0.9634
YNL097C	YPL181W	0.9021
YNL098C	YOR101W	0.7380
YNL102W	YNR052C	0.7380
YNL102W	YPL106C	0.7380
YNL102W	YPR019W	0.8953
YNL102W	YPR135W	0.9996
YNL103W	YNL272C	0.5774
YNL103W	YPL038W	0.9932
YNL104C	YOR108W	0.9554
YNL106C	YPR171W	0.9539
YNL107W	YNL136W	0.8659
YNL107W	YOL012C	0.9805
YNL107W	YOR244W	0.9999
YNL107W	YPL235W	0.8087
YNL107W	YPR023C	0.9021
YNL110C	YNL175C	0.7380

YNL110C	YNL182C	0.9313
YNL110C	YOL077C	0.9648
YNL110C	YOL127W	0.7380
YNL110C	YOR063W	0.8659
YNL110C	YOR206W	0.8659
YNL110C	YOR272W	0.9313
YNL110C	YOR294W	0.7380
YNL110C	YPL012W	0.8659
YNL110C	YPL043W	0.8659
YNL110C	YPL093W	0.9648
YNL110C	YPL146C	0.7380
YNL110C	YPL211W	0.7380
YNL110C	YPR016C	0.9313
YNL110C	YPR169W	0.7380
YNL113W	YNL151C	0.7380
YNL113W	YNL248C	0.7380
YNL113W	YNR003C	0.7380
YNL113W	YOR116C	0.9313
YNL113W	YOR207C	0.9904
YNL113W	YOR210W	0.7380
YNL113W	YOR340C	0.9266
YNL113W	YOR341W	0.9499
YNL113W	YPR010C	0.9779
YNL113W	YPR110C	0.9988
YNL113W	YPR187W	0.7380
YNL113W	YPR190C	0.7380
YNL116W	YNL311C	0.7380
YNL118C	YOL149W	1.0000
YNL118C	YPR129W	0.8689
YNL123W	YOR317W	0.9099
YNL131W	YNR017W	0.9994
YNL132W	YNL178W	0.7380
YNL132W	YNL287W	0.7380
YNL132W	YOR078W	0.7380
YNL132W	YOR096W	0.7380
YNL132W	YOR310C	0.8659
YNL132W	YPL043W	0.8659
YNL132W	YPL081W	0.7380
YNL132W	YPL198W	0.7380
YNL132W	YPR137W	0.8659
YNL132W	YPR144C	0.8659
YNL135C	YPR093C	0.6672
YNL136W	YOR244W	0.9794
YNL136W	YPR023C	0.8659
YNL137C	YOR158W	0.7380
YNL137C	YPL013C	0.7380
YNL137C	YPL118W	0.7380

YNL138W	YOR122C	0.8179
YNL139C	YNL189W	0.8659
YNL139C	YNL253W	0.9808
YNL147W	YOR308C	0.8659
YNL147W	YPR082C	0.8659
YNL147W	YPR178W	0.9128
YNL151C	YNR003C	0.9887
YNL151C	YOR116C	0.8659
YNL151C	YOR207C	0.9813
YNL151C	YPR110C	0.9313
YNL151C	YPR187W	0.7380
YNL151C	YPR190C	0.8659
YNL152W	YPL242C	0.8953
YNL161W	YNL272C	0.9567
YNL161W	YOL036W	0.8353
YNL172W	YOR249C	0.9021
YNL178W	YPL204W	0.7543
YNL182C	YNR053C	0.7380
YNL182C	YPL093W	0.8087
YNL182C	YPR016C	0.8659
YNL188W	YOR257W	0.9934
YNL189W	YNL232W	0.7380
YNL189W	YOL004W	0.7380
YNL189W	YOL021C	0.8659
YNL189W	YOR001W	0.9904
YNL189W	YOR098C	1.0000
YNL189W	YOR310C	0.7380
YNL189W	YPL020C	0.9946
YNL189W	YPL088W	0.5774
YNL189W	YPL153C	0.7380
YNL189W	YPL178W	0.8659
YNL189W	YPR119W	0.8628
YNL197C	YOR204W	0.7891
YNL197C	YPL256C	0.7420
YNL199C	YPL075W	0.8965
YNL201C	YPL153C	0.9503
YNL201C	YPR040W	0.8603
YNL207W	YOR056C	0.9986
YNL207W	YOR145C	0.9855
YNL207W	YOR290C	0.7380
YNL207W	YPL012W	0.7380
YNL207W	YPL204W	0.9996
YNL207W	YPL266W	0.9959
YNL209W	YOL006C	0.7380
YNL209W	YOR061W	0.7380
YNL209W	YOR069W	0.8659
YNL209W	YOR140W	0.7380

YNL209W	YOR196C	0.7380
YNL209W	YPL001W	0.7380
YNL209W	YPL009C	0.7380
YNL209W	YPL020C	0.7380
YNL209W	YPL022W	0.7380
YNL209W	YPL106C	0.9992
YNL209W	YPL137C	0.7380
YNL209W	YPL212C	0.7380
YNL209W	YPL231W	0.7380
YNL209W	YPL240C	0.7380
YNL209W	YPR031W	0.7380
YNL209W	YPR045C	0.7380
YNL209W	YPR074C	0.7380
YNL209W	YPR105C	0.7380
YNL209W	YPR110C	0.8659
YNL209W	YPR144C	0.8659
YNL209W	YPR163C	0.7380
YNL209W	YPR189W	0.7380
YNL214W	YOL044W	0.9124
YNL215W	YOR141C	0.8659
YNL215W	YPL235W	0.8659
YNL216W	YPL075W	0.9935
YNL216W	YPL106C	0.7380
YNL221C	YNL282W	0.9983
YNL222W	YNL317W	0.9990
YNL222W	YOR151C	0.9769
YNL222W	YOR179C	0.9863
YNL222W	YPL008W	0.8603
YNL222W	YPR086W	0.9249
YNL222W	YPR107C	0.9813
YNL225C	YOL091W	0.9914
YNL225C	YOR129C	0.7909
YNL225C	YOR373W	0.9747
YNL225C	YPL124W	0.9932
YNL230C	YPL046C	0.9967
YNL232W	YNR024W	0.9021
YNL232W	YOL021C	1.0000
YNL232W	YOL142W	0.9800
YNL232W	YOR001W	0.9991
YNL232W	YOR076C	0.9963
YNL233W	YPL240C	0.6672
YNL236W	YNR010W	0.9266
YNL236W	YOL051W	0.9999
YNL236W	YOL135C	0.9969
YNL236W	YOR174W	0.7380
YNL236W	YPR070W	0.8659
YNL236W	YPR168W	0.7380

YNL238W	YOL031C	0.8659
YNL243W	YOR181W	0.8028
YNL244C	YOR361C	0.9999
YNL244C	YPL237W	0.9249
YNL244C	YPR041W	1.0000
YNL248C	YOR210W	0.7380
YNL248C	YOR224C	0.7380
YNL248C	YOR310C	0.7380
YNL248C	YOR340C	0.9995
YNL248C	YOR341W	0.9998
YNL248C	YPR010C	0.9969
YNL248C	YPR110C	0.9953
YNL248C	YPR187W	0.9313
YNL251C	YOL115W	0.8087
YNL251C	YPL178W	0.9021
YNL251C	YPL190C	0.9999
YNL252C	YNL284C	0.8659
YNL258C	YOR075W	0.9266
YNL261W	YPL001W	0.9800
YNL261W	YPR162C	1.0000
YNL261W	YPR164W	0.9099
YNL262W	YNL299W	0.9710
YNL262W	YOL115W	0.9099
YNL262W	YOL139C	0.7380
YNL262W	YPR175W	1.0000
YNL263C	YOR132W	0.9138
YNL265C	YPR173C	0.9925
YNL271C	YNL293W	0.8258
YNL271C	YOR122C	0.9327
YNL271C	YPR165W	0.7909
YNL273W	YOL006C	0.9099
YNL281W	YPL240C	0.9470
YNL284C	YOR150W	0.7380
YNL284C	YOR201C	0.7380
YNL287W	YPL010W	0.6147
YNL288W	YNR052C	1.0000
YNL288W	YPR072W	0.9991
YNL289W	YOR032C	0.9884
YNL289W	YPL031C	0.9898
YNL290W	YOL094C	0.9932
YNL290W	YOR144C	0.9942
YNL290W	YOR217W	0.8659
YNL293W	YOR089C	0.7696
YNL299W	YOL115W	0.9551
YNL299W	YPR175W	0.8965
YNL304W	YOR326W	0.9099
YNL306W	YOR158W	0.7380

YNL306W	YPL013C	0.7380
YNL308C	YPL012W	0.7380
YNL309W	YPL082C	0.8953
YNL313C	YPL106C	0.7380
YNL317W	YPR107C	0.9313
YNL329C	YOL044W	0.9977
YNL330C	YOL004W	1.0000
YNL330C	YOL006C	0.7380
YNL330C	YPL139C	1.0000
YNL330C	YPL181W	0.9996
YNL330C	YPR023C	0.9998
YNL333W	YNL334C	0.5774
YNR003C	YOR116C	0.9953
YNR003C	YOR207C	0.9951
YNR003C	YOR224C	0.8659
YNR003C	YPR110C	0.9908
YNR003C	YPR187W	0.7380
YNR003C	YPR190C	0.9981
YNR007C	YOR160W	0.7380
YNR010W	YOL051W	0.9794
YNR010W	YOL135C	0.9992
YNR010W	YOR174W	1.0000
YNR010W	YPL042C	0.9266
YNR010W	YPR070W	0.9970
YNR010W	YPR168W	0.9794
YNR011C	YOR148C	0.9919
YNR017W	YPL063W	1.0000
YNR023W	YOR290C	0.9956
YNR023W	YPL016W	0.8659
YNR023W	YPL129W	0.9266
YNR024W	YOL021C	0.9726
YNR024W	YOL142W	0.8087
YNR024W	YOR001W	0.9897
YNR027W	YPL106C	0.7380
YNR035C	YOR181W	0.6147
YNR037C	YOR158W	0.7380
YNR046W	YNR050C	0.9266
YNR046W	YOL124C	0.9972
YNR049C	YOR327C	0.9215
YNR049C	YPL232W	0.9762
YNR050C	YOR164C	0.7380
YNR051C	YOR204W	0.6147
YNR052C	YPR072W	0.9215
YNR053C	YOR063W	0.9021
YNR053C	YPL093W	0.9987
YNR053C	YPL131W	0.9021
YNR053C	YPR016C	0.9499

YNR068C	YNR069C	0.5774
YOL001W	YPL031C	1.0000
YOL004W	YOR061W	0.7380
YOL004W	YPL139C	0.9991
YOL004W	YPL181W	0.9982
YOL004W	YPR023C	0.9993
YOL005C	YOR151C	0.9499
YOL005C	YOR210W	0.9021
YOL005C	YOR224C	0.9733
YOL005C	YPL129W	0.9021
YOL005C	YPR093C	0.8087
YOL005C	YPR187W	0.9021
YOL010W	YPL217C	0.9975
YOL010W	YPR137W	0.7380
YOL012C	YPL127C	0.6147
YOL012C	YPL235W	0.9900
YOL021C	YOL142W	0.9999
YOL021C	YOR001W	0.9999
YOL021C	YOR076C	0.9964
YOL025W	YOL133W	0.9783
YOL038W	YOR157C	0.7380
YOL038W	YOR261C	0.8566
YOL038W	YOR362C	0.9902
YOL038W	YPR103W	0.9992
YOL041C	YOL077C	0.8659
YOL041C	YOR206W	0.7380
YOL041C	YPL043W	0.8659
YOL041C	YPL198W	0.7380
YOL051W	YOL135C	0.9991
YOL051W	YOR174W	0.8659
YOL051W	YPL042C	0.8566
YOL051W	YPL129W	0.9584
YOL051W	YPL248C	1.0000
YOL051W	YPR070W	0.7380
YOL051W	YPR168W	0.9206
YOL062C	YPL259C	0.9215
YOL068C	YOR279C	0.8566
YOL069W	YPR141C	0.5774
YOL072W	YOR257W	0.9997
YOL076W	YPR131C	0.9808
YOL077C	YOL127W	0.7380
YOL077C	YOR063W	0.9266
YOL077C	YOR206W	0.8659
YOL077C	YOR272W	0.9726
YOL077C	YPL043W	0.9808
YOL077C	YPL093W	0.9021
YOL077C	YPL131W	0.7380

YOL077C	YPL198W	0.7380
YOL077C	YPL211W	0.9624
YOL077C	YPR016C	0.8560
YOL081W	YOR371C	0.9215
YOL082W	YPR049C	0.9710
YOL090W	YOR033C	0.9998
YOL090W	YOR323C	0.7380
YOL090W	YPR179C	0.7380
YOL091W	YOR177C	0.9998
YOL091W	YOR373W	0.8965
YOL094C	YOR144C	0.9996
YOL094C	YOR217W	0.8659
YOL100W	YPL004C	0.7543
YOL104C	YPL200W	0.9215
YOL108C	YOR344C	0.9099
YOL111C	YOR007C	0.9964
YOL111C	YOR164C	1.0000
YOL111C	YPL174C	0.9598
YOL115W	YPL146C	0.8628
YOL115W	YPL190C	0.7380
YOL120C	YOR063W	0.7380
YOL127W	YPL037C	0.9555
YOL127W	YPL043W	0.7380
YOL135C	YOR174W	1.0000
YOL135C	YPR070W	1.0000
YOL135C	YPR168W	0.9993
YOL139C	YOR276W	0.9901
YOL142W	YOR001W	0.9951
YOL142W	YOR076C	0.9743
YOL144W	YPL211W	0.9710
YOL145C	YOR039W	0.8659
YOL145C	YOR061W	0.8659
YOL145C	YOR123C	1.0000
YOL146W	YPR019W	0.8953
YOL146W	YPR135W	0.9886
YOL148C	YPL047W	0.9904
YOL148C	YPL254W	0.9980
YOL149W	YOR167C	0.7253
YOL149W	YPL204W	0.6672
YOR001W	YOR076C	0.8087
YOR007C	YOR164C	0.9551
YOR027W	YPL240C	1.0000
YOR028C	YPL046C	0.9138
YOR034C	YOR171C	0.8179
YOR035C	YOR326W	0.9938
YOR036W	YPL151C	0.8640
YOR039W	YOR061W	0.9977

YOR039W	YOR361C	0.7380
YOR056C	YOR145C	0.9707
YOR057W	YPL240C	0.9892
YOR061W	YOR119C	0.9928
YOR063W	YOR206W	0.7380
YOR063W	YPL012W	0.7380
YOR063W	YPL043W	0.7380
YOR063W	YPL093W	0.8087
YOR063W	YPR016C	0.9021
YOR063W	YPR189W	0.7380
YOR069W	YOR132W	1.0000
YOR078W	YOR310C	0.8087
YOR078W	YPL012W	0.7380
YOR078W	YPL126W	0.7380
YOR078W	YPL217C	0.7380
YOR078W	YPR137W	0.9313
YOR080W	YPR019W	0.9099
YOR080W	YPR135W	0.9994
YOR083W	YPL031C	0.6042
YOR085W	YOR103C	0.9962
YOR089C	YOR370C	0.8028
YOR094W	YOR129C	0.9917
YOR096W	YOR310C	0.8659
YOR096W	YPR144C	0.8659
YOR098C	YOR160W	0.9099
YOR098C	YPL169C	0.9897
YOR106W	YPL195W	0.9881
YOR109W	YPR171W	0.9099
YOR110W	YPL007C	0.8659
YOR116C	YOR207C	0.9975
YOR116C	YOR210W	0.7380
YOR116C	YOR224C	0.9568
YOR116C	YPR110C	0.9953
YOR116C	YPR187W	0.9313
YOR116C	YPR190C	0.9820
YOR117W	YOR259C	0.9903
YOR117W	YOR261C	0.7380
YOR117W	YPR108W	0.9266
YOR124C	YOR138C	0.9998
YOR127W	YPL256C	0.8462
YOR127W	YPR165W	0.5774
YOR134W	YPR165W	0.9099
YOR136W	YPL235W	0.7380
YOR140W	YPL203W	0.9503
YOR141C	YOR189W	0.8087
YOR141C	YPL129W	0.9021
YOR141C	YPL235W	0.9813

YOR145C	YPL126W	0.8659
YOR145C	YPL266W	0.8566
YOR151C	YOR210W	0.9926
YOR151C	YOR224C	0.9948
YOR151C	YPL129W	0.9743
YOR151C	YPR093C	0.9759
YOR151C	YPR187W	0.9975
YOR156C	YPR034W	0.7380
YOR157C	YOR362C	0.9266
YOR157C	YPR103W	0.9981
YOR158W	YPL013C	0.7380
YOR159C	YPL213W	0.9021
YOR159C	YPR178W	0.7380
YOR159C	YPR182W	0.9644
YOR165W	YPR028W	0.9215
YOR174W	YPL042C	0.8566
YOR174W	YPR070W	0.9999
YOR174W	YPR168W	0.9157
YOR176W	YOR257W	0.8953
YOR177C	YOR373W	0.9099
YOR181W	YPR154W	0.9911
YOR181W	YPR159W	0.9099
YOR189W	YPL129W	0.9021
YOR189W	YPL235W	0.8087
YOR197W	YPR154W	0.5774
YOR198C	YPL255W	0.9099
YOR204W	YOR361C	0.7380
YOR204W	YPR041W	0.7380
YOR206W	YOR272W	0.9499
YOR206W	YPL043W	0.8659
YOR206W	YPL093W	0.9021
YOR206W	YPL131W	0.7380
YOR206W	YPR016C	0.9266
YOR207C	YOR210W	0.9634
YOR207C	YOR224C	0.9634
YOR207C	YPR110C	0.9987
YOR207C	YPR187W	0.9813
YOR207C	YPR190C	0.9904
YOR210W	YOR224C	0.9634
YOR210W	YOR340C	0.9266
YOR210W	YOR341W	0.9928
YOR210W	YPR010C	0.9157
YOR210W	YPR110C	0.9313
YOR210W	YPR187W	0.7380
YOR210W	YPR190C	0.7380
YOR224C	YOR340C	0.9266
YOR224C	YOR341W	0.9928

YOR224C	YPL129W	0.7380
YOR224C	YPR010C	0.7380
YOR224C	YPR110C	0.8659
YOR224C	YPR187W	0.9800
YOR224C	YPR190C	0.7380
YOR229W	YOR230W	0.9957
YOR229W	YOR283W	0.6672
YOR244W	YPL082C	0.8953
YOR244W	YPR023C	1.0000
YOR254C	YPL094C	1.0000
YOR257W	YPR103W	0.9215
YOR259C	YOR261C	0.7380
YOR259C	YPL248C	0.9917
YOR259C	YPR108W	0.9266
YOR260W	YPL237W	0.9946
YOR261C	YPR108W	0.9624
YOR270C	YOR332W	0.9936
YOR270C	YPR036W	0.9996
YOR272W	YPL043W	0.9313
YOR272W	YPL093W	0.9285
YOR272W	YPL131W	0.7380
YOR272W	YPL198W	0.7380
YOR272W	YPL211W	0.9021
YOR272W	YPR016C	0.9021
YOR275C	YPR173C	0.9327
YOR290C	YPL016W	0.9999
YOR290C	YPL129W	0.9915
YOR290C	YPR034W	1.0000
YOR294W	YPL131W	0.9998
YOR294W	YPR102C	1.0000
YOR304W	YPL082C	0.8087
YOR308C	YPR178W	0.8659
YOR310C	YPL126W	0.9499
YOR310C	YPL235W	0.7380
YOR310C	YPR137W	0.9743
YOR326W	YPL269W	0.9972
YOR326W	YPR032W	0.9215
YOR327C	YPL232W	1.0000
YOR332W	YPL240C	0.6672
YOR332W	YPR036W	0.9866
YOR340C	YOR341W	1.0000
YOR340C	YPR010C	0.9983
YOR340C	YPR110C	0.9808
YOR340C	YPR187W	0.9266
YOR341W	YPR010C	0.9999
YOR341W	YPR110C	0.9998
YOR341W	YPR187W	0.9992

YOR346W	YPL167C	0.9780
YOR347C	YPL203W	0.8714
YOR361C	YPL237W	0.8566
YOR361C	YPR041W	1.0000
YOR362C	YPR103W	0.9984
YOR368W	YPL194W	0.9979
YOR370C	YPR176C	0.9967
YPL001W	YPR162C	0.9285
YPL002C	YPL065W	0.9919
YPL003W	YPR066W	0.9889
YPL011C	YPL129W	0.9157
YPL012W	YPL043W	0.9313
YPL012W	YPL131W	0.7380
YPL012W	YPL266W	0.7380
YPL012W	YPR144C	0.8659
YPL013C	YPL118W	0.7380
YPL016W	YPL129W	0.8566
YPL016W	YPR034W	0.8659
YPL018W	YPL233W	0.6672
YPL031C	YPL219W	0.9992
YPL031C	YPR025C	0.9138
YPL037C	YPR072W	0.9099
YPL042C	YPL248C	0.9980
YPL042C	YPR070W	0.9266
YPL043W	YPL093W	0.8659
YPL043W	YPL198W	0.7380
YPL047W	YPL254W	0.9634
YPL048W	YPR080W	0.8659
YPL059W	YPL106C	0.7380
YPL070W	YPR193C	0.5774
YPL081W	YPR144C	0.7380
YPL082C	YPL129W	0.8715
YPL082C	YPL178W	0.9249
YPL084W	YPR173C	0.9892
YPL085W	YPR181C	0.9990
YPL086C	YPL101W	0.9970
YPL093W	YPL131W	0.9021
YPL093W	YPL211W	0.7380
YPL093W	YPR016C	0.9813
YPL093W	YPR102C	0.7380
YPL106C	YPL137C	0.7380
YPL106C	YPL183C	0.7380
YPL106C	YPL231W	0.7380
YPL106C	YPL240C	0.9756
YPL106C	YPR045C	0.7380
YPL106C	YPR093C	0.7380
YPL106C	YPR108W	0.7380

YPL106C	YPR140W	0.7380
YPL106C	YPR144C	0.7380
YPL106C	YPR162C	0.7380
YPL106C	YPR171W	0.7380
YPL106C	YPR189W	0.7380
YPL115C	YPL256C	0.8999
YPL122C	YPR056W	0.9555
YPL124W	YPL155C	0.5774
YPL124W	YPL255W	0.9138
YPL126W	YPR137W	0.8659
YPL126W	YPR144C	0.8659
YPL127C	YPR052C	0.7718
YPL128C	YPL153C	0.7380
YPL129W	YPL235W	0.8659
YPL129W	YPR034W	0.8566
YPL129W	YPR187W	0.8659
YPL131W	YPL211W	0.7380
YPL131W	YPR016C	0.9499
YPL131W	YPR102C	0.8063
YPL139C	YPL181W	0.9904
YPL139C	YPR023C	0.9964
YPL151C	YPL213W	0.9021
YPL151C	YPR101W	0.8659
YPL161C	YPR165W	0.9908
YPL169C	YPL178W	0.8566
YPL178W	YPL190C	0.9313
YPL193W	YPL235W	0.8965
YPL198W	YPL211W	0.7380
YPL204W	YPL266W	0.9021
YPL204W	YPR007C	0.9696
YPL210C	YPL243W	0.9313
YPL210C	YPR088C	0.9313
YPL213W	YPR101W	0.9021
YPL213W	YPR182W	0.7380
YPL217C	YPR137W	0.8659
YPL217C	YPR144C	0.8659
YPL218W	YPR181C	0.9999
YPL232W	YPR032W	0.9215
YPL237W	YPR041W	1.0000
YPL240C	YPR201W	0.5774
YPL243W	YPR088C	0.9313
YPL248C	YPR086W	0.9983
YPL249C	YPR154W	0.5774
YPL253C	YPR141C	0.8953
YPL259C	YPR029C	0.9648
YPL266W	YPR137W	0.7380
YPL269W	YPR120C	0.8965

YPR010C	YPR110C	0.9970
YPR010C	YPR187W	0.7380
YPR019W	YPR135W	0.9918
YPR025C	YPR056W	0.9772
YPR032W	YPR110C	0.7380
YPR070W	YPR168W	0.9266
YPR101W	YPR182W	0.7380
YPR110C	YPR187W	0.9648
YPR110C	YPR190C	0.9820
YPR137W	YPR144C	0.8659
YPR178W	YPR182W	0.8659
YPR187W	YPR190C	0.8659
