# Supplementary material for: Predicting multiplex subcellular localization of proteins using protein-protein interaction network: a comparative study
Source: BMC Bioinformatics. 2012 Jun 25;13(Suppl 10):S20. doi: 10.1186/1471-2105-13-S10-S20 (PMC3314587; doi:10.1186/1471-2105-13-S10-S20)
Supplement: Additional file 2 — The subcellular localization annotations of 3165 proteins in the PPI network collected by the Yeast Gtp Fusion Localization database. [file 1471-2105-13-S10-S20-S2.pdf]

Supplementary Table 2 The subcellular localization annotations of 3165 proteins in the PPI network collected by the Yeast Gtp Fusion Localization database.
The symbol "T" denotes that the proteins is experimentally observed in this localization.

yORF	ambiguous	mitochondrion	vacuole	spindle pole	cell periphery	punctate composite	vacuolar membrane	ER	nuclear periphery	endosome	bud neck	microtubule	Golgi	late Golgi	peroxisome	actin	nucleolus	cytoplasm	ER to Golgi	early Golgi	lipid particle	nucleus	bud
YAL001C	F	F	F	F	F	F	F	F	F	F	F	F	F	F	F	F	F	T	F	F	F	T	F
YAL002W	F	F	F	F	F	F	F	F	F	T	F	F	F	F	F	F	F	F	F	F	F	F	F
YAL003W	F	F	F	F	F	F	F	F	F	F	F	F	F	F	F	F	F	F	F	F	F	F	F
YAL005C	F	F	F	F	F	F	F	F	F	F	F	F	F	F	F	F	F	T	F	F	F	F	F
YAL007C	F	F	F	F	F	F	F	T	F	F	F	F	F	F	F	F	F	F	F	F	F	F	F
YAL010C	F	T	F	F	F	F	F	F	F	F	F	F	F	F	F	F	F	F	F	F	F	F	F
YAL011W	F	F	F	F	F	F	F	F	F	F	F	F	F	F	F	F	F	F	F	F	F	T	F
YAL013W	F	F	F	F	F	F	F	F	F	F	F	F	F	F	F	F	F	F	F	F	F	F	F
YAL014C	F	F	F	F	F	F	F	F	F	F	F	F	F	F	F	F	F	T	F	F	F	T	F
YAL015C	F	F	F	F	F	F	F	F	F	F	F	F	F	F	F	F	F	T	F	F	F	T	F
YAL016W	F	F	F	F	F	F	F	F	F	F	F	F	F	F	F	F	F	T	F	F	F	T	F
YAL017W	F	F	F	F	F	F	F	F	F	F	F	F	F	F	F	F	F	T	F	F	F	F	F
YAL020C	F	F	F	F	F	F	F	F	F	F	F	F	F	F	F	F	F	F	F	F	F	F	F
YAL021C	F	F	F	F	F	F	F	F	F	F	F	F	F	F	F	F	F	T	F	F	F	F	F
YAL023C	F	F	F	F	F	F	F	T	F	F	F	F	F	F	F	F	F	F	F	F	F	F	F
YAL024C	F	F	F	F	F	F	F	F	F	F	F	F	F	F	F	F	F	T	F	F	F	F	F
YAL026C	F	F	F	F	F	F	F	F	F	F	F	F	F	T	F	F	F	F	F	T	F	F	F
YAL027W	F	F	F	F	F	F	F	F	F	F	F	F	F	F	F	F	F	F	F	F	F	T	F
YAL028W	F	F	F	F	F	F	F	F	F	F	F	F	F	F	F	F	F	F	F	F	F	F	F
YAL029C	T	F	F	F	T	F	F	F	F	F	T	F	F	F	F	F	F	T	F	F	F	F	T
YAL030W	F	F	F	F	F	F	F	F	F	F	F	F	F	F	F	F	F	F	F	F	F	F	F
YAL031C	F	F	F	F	F	F	F	F	F	F	F	F	F	F	F	F	F	T	F	F	F	F	F
YAL032C	F	F	F	F	F	F	F	F	F	F	F	F	F	F	F	F	F	F	F	F	F	T	F
YAL033W	F	F	F	F	F	F	F	F	F	F	F	F	F	F	F	F	F	T	F	F	F	T	F
YAL034C	F	F	F	F	F	F	F	F	F	F	F	F	F	F	F	F	F	F	F	F	F	F	F
YAL034W-A	F	F	F	T	F	F	F	F	F	F	F	F	F	F	F	F	F	F	F	F	F	F	F
YAL035W	F	F	F	F	F	F	F	F	F	F	F	F	F	F	F	F	F	T	F	F	F	F	F
YAL036C	F	F	F	F	F	F	F	F	F	F	F	F	F	F	F	F	F	T	F	F	F	F	F
YAL038W	F	F	F	F	F	F	F	F	F	F	F	F	F	F	F	F	F	T	F	F	F	F	F
YAL040C	F	F	F	F	F	F	F	F	F	F	F	F	F	F	F	F	F	F	F	F	F	F	F
YAL041W	F	F	F	F	F	F	F	F	F	F	F	F	F	F	F	F	F	T	F	F	F	T	F
YAL042W	F	F	F	F	F	F	F	F	F	F	F	F	F	F	F	F	F	F	F	F	F	F	F
YAL043C	F	F	F	F	F	F	F	F	F	F	F	F	F	F	F	F	F	F	F	F	F	T	F
YAL047C	F	F	F	T	F	F	F	F	F	F	F	F	F	F	F	F	F	F	F	F	F	F	F
YAL049C	F	F	F	F	F	F	F	F	F	F	F	F	F	F	F	F	F	T	F	F	F	F	F
YAL056W	F	T	F	F	F	F	F	F	F	F	F	F	F	F	F	F	F	T	F	F	F	F	F
YAL059W	F	F	F	F	F	F	F	F	F	F	F	F	F	F	F	F	F	F	F	F	F	T	F
YAL062W	F	F	F	F	F	F	F	F	F	F	F	F	F	F	F	F	F	F	F	F	F	F	F
YAR002C-A	F	F	F	F	F	F	T	F	F	F	F	F	F	F	F	F	F	F	F	F	F	F	F
YAR002W	F	F	F	F	F	F	F	F	T	F	F	F	F	F	F	F	F	F	F	F	F	F	F
YAR003W	F	F	F	F	F	F	F	F	F	F	F	F	F	F	F	F	F	F	F	F	F	T	F
YAR007C	F	F	F	F	F	F	F	F	F	F	F	F	F	F	F	F	F	F	F	F	F	T	F
YAR009C	F	F	F	F	F	T	F	F	F	F	F	F	F	F	F	F	F	F	F	F	F	F	F
YAR014C	F	F	F	F	F	F	F	F	F	F	F	F	F	F	F	F	F	T	F	F	F	T	F
YAR015W	F	F	F	F	F	F	F	F	F	F	F	F	F	F	F	F	F	T	F	F	F	T	F
YAR018C	F	F	F	F	F	F	F	F	F	F	F	F	F	F	F	F	F	F	F	F	F	F	F
YAR019C	T	F	F	T	F	F	F	F	F	F	F	F	F	F	F	F	F	F	F	F	F	F	F
YAR027W	T	F	F	F	F	F	F	F	F	F	F	F	F	F	F	F	F	F	F	F	F	F	F
YAR028W	T	F	F	F	F	F	F	F	F	F	F	F	F	F	F	F	F	F	F	F	F	F	F
YAR042W	F	F	F	F	F	F	F	F	F	F	F	F	F	F	F	F	F	F	F	F	F	F	F
YBL002W	F	F	F	F	F	F	F	F	F	F	F	F	F	F	F	F	F	F	F	F	F	T	F
YBL003C	F	F	F	F	F	F	F	F	F	F	F	F	F	F	F	F	F	F	F	F	F	T	F
YBL004W	F	F	F	F	F	F	F	F	F	F	F	F	F	F	F	F	T	T	F	F	F	F	F
YBL007C	F	F	F	F	F	F	F	F	F	F	F	F	F	F	F	T	F	T	F	F	F	F	F
YBL008W	F	F	F	F	F	F	F	F	F	F	F	F	F	F	F	F	F	T	F	F	F	T	F
YBL010C	F	F	F	F	F	F	F	F	F	F	F	F	F	T	F	F	F	F	F	F	F	F	F
YBL014C	F	F	F	F	F	F	F	F	F	F	F	F	F	F	F	F	T	T	F	F	F	F	F
YBL016W	F	F	F	F	F	F	F	F	F	F	F	F	F	F	F	F	F	T	F	F	F	T	F
YBL017C	F	F	F	F	F	T	F	F	F	T	F	F	F	F	F	F	F	F	F	F	F	F	F
YBL018C	F	F	F	F	F	F	F	F	F	F	F	F	F	F	F	F	F	F	F	F	F	T	F
YBL019W	F	F	F	F	F	F	F	F	F	F	F	F	F	F	F	F	F	T	F	F	F	T	F
YBL021C	F	F	F	F	F	F	F	F	F	F	F	F	F	F	F	F	F	T	F	F	F	T	F

YBL023C	F	F	F	F	F	F	F	F	F	F	F	F	F	F	F	F	F	T	F	F	F	T	F
YBL024W	F	F	F	F	F	F	F	F	F	F	F	F	F	F	F	F	F	F	F	F	F	T	F
YBL025W	F	F	F	F	F	F	F	F	F	F	F	F	F	F	F	F	T	F	F	F	F	F	F
YBL026W	F	F	F	F	F	F	F	F	F	F	F	F	F	F	F	F	F	T	F	F	F	T	F
YBL027W	F	F	F	F	F	F	F	F	F	F	F	F	F	F	F	F	F	T	F	F	F	F	F
YBL030C	F	F	F	F	F	F	F	F	F	F	F	F	F	F	F	F	F	F	F	F	F	F	F
YBL032W	F	F	F	F	F	F	F	F	F	F	F	F	F	F	F	F	F	T	F	F	F	F	F
YBL034C	T	F	F	T	F	F	F	F	F	F	F	F	F	F	F	F	F	F	F	F	F	F	F
YBL035C	F	F	F	F	F	F	F	F	F	F	F	F	F	F	F	F	F	F	F	F	F	T	F
YBL037W	F	F	F	F	T	F	F	F	F	F	T	F	F	F	F	F	F	F	F	F	F	F	F
YBL038W	F	T	F	F	F	F	F	F	F	F	F	F	F	F	F	F	F	F	F	F	F	F	F
YBL039C	F	F	F	F	F	F	F	F	F	F	F	F	F	F	F	F	F	T	F	F	F	F	F
YBL041W	F	F	F	F	F	F	F	F	F	F	F	F	F	F	F	F	F	F	F	F	F	F	F
YBL045C	F	T	F	F	F	F	F	F	F	F	F	F	F	F	F	F	F	F	F	F	F	F	F
YBL046W	F	F	F	F	F	F	F	F	F	F	F	F	F	F	F	F	F	F	F	F	F	T	F
YBL047C	F	F	F	F	F	T	F	F	F	F	F	F	F	F	F	F	F	F	F	F	F	F	F
YBL049W	F	F	F	F	F	F	F	F	F	F	F	F	F	F	F	F	F	F	F	F	F	F	F
YBL050W	F	F	F	F	F	F	F	F	F	F	F	F	F	F	F	F	F	F	F	F	F	F	F
YBL051C	F	F	F	F	F	F	F	F	F	F	F	F	F	F	F	F	F	T	F	F	F	F	F
YBL052C	F	F	F	F	F	F	F	F	F	F	F	F	F	F	F	F	F	F	F	F	F	T	F
YBL056W	F	F	F	F	F	F	F	F	F	F	F	F	F	F	F	F	F	T	F	F	F	T	F
YBL057C	F	T	F	F	F	F	F	F	F	F	F	F	F	F	F	F	F	F	F	F	F	F	F
YBL058W	F	F	F	F	F	F	F	F	F	F	F	F	F	F	F	F	F	T	F	F	F	T	F
YBL060W	F	F	F	F	F	F	F	F	F	F	T	F	F	F	F	F	F	T	F	F	F	F	F
YBL061C	F	F	F	F	F	F	F	F	F	F	T	F	F	F	F	F	F	T	F	F	F	T	F
YBL066C	F	F	F	F	F	F	F	F	F	F	F	F	F	F	F	F	F	F	F	F	F	F	F
YBL067C	T	F	F	F	F	F	F	F	F	F	F	F	F	F	F	F	F	F	F	F	F	F	F
YBL068W	F	F	F	F	F	F	F	F	F	F	F	F	F	F	F	F	F	T	F	F	F	F	F
YBL071W-A	F	F	F	F	F	F	F	F	F	F	F	F	F	F	F	F	F	T	F	F	F	T	F
YBL072C	F	F	F	F	F	F	F	F	F	F	F	F	F	F	F	F	F	T	F	F	F	F	F
YBL074C	F	F	F	F	F	F	F	F	F	F	F	F	F	F	F	F	F	T	F	F	F	T	F
YBL075C	F	F	F	F	F	F	F	F	F	F	F	F	F	F	F	F	F	T	F	F	F	F	F
YBL076C	F	F	F	F	F	F	F	F	F	F	F	F	F	F	F	F	F	T	F	F	F	F	F
YBL078C	F	F	F	F	F	F	F	F	F	F	F	F	F	F	F	F	F	F	F	F	F	F	F
YBL079W	F	F	F	F	F	F	F	F	T	F	F	F	F	F	F	F	F	F	F	F	F	F	F
YBL084C	F	F	F	F	F	F	F	F	F	F	F	F	F	F	F	F	F	T	F	F	F	T	F
YBL085W	F	F	F	F	T	F	F	F	F	F	T	F	F	F	F	F	F	T	F	F	F	F	T
YBL087C	F	F	F	F	F	F	F	F	F	F	F	F	F	F	F	F	F	F	F	F	F	T	F
YBL088C	F	F	F	F	F	F	F	F	F	F	F	F	F	F	F	F	F	F	F	F	F	F	F
YBL090W	F	T	F	F	F	F	F	F	F	F	F	F	F	F	F	F	F	F	F	F	F	F	F
YBL093C	F	F	F	F	F	F	F	F	F	F	F	F	F	F	F	F	F	F	F	F	F	T	F
YBL097W	F	F	F	F	F	F	F	F	F	F	F	F	F	F	F	F	F	F	F	F	F	T	F
YBL099W	F	T	F	F	F	F	F	F	F	F	F	F	F	F	F	F	F	F	F	F	F	F	F
YBL101C	F	F	F	F	F	F	F	F	F	F	F	F	F	F	F	F	F	T	F	F	F	F	F
YBL103C	F	F	F	F	F	F	F	F	F	F	F	F	F	F	F	F	F	T	F	F	F	T	F
YBL104C	F	F	F	F	F	F	F	F	F	F	F	F	F	F	F	F	F	T	F	F	F	F	F
YBL105C	T	F	F	F	F	F	F	F	F	F	T	F	F	F	F	F	F	T	F	F	F	F	T
YBR003W	F	T	F	F	F	F	F	F	F	F	F	F	F	F	F	F	F	F	F	F	F	F	F
YBR005W	T	F	F	F	F	F	F	F	F	F	F	F	F	F	F	F	F	F	F	F	F	F	F
YBR009C	F	F	F	F	F	F	F	F	F	F	F	F	F	F	F	F	F	F	F	F	F	T	F
YBR010W	F	F	F	F	F	F	F	F	F	F	F	F	F	F	F	F	F	F	F	F	F	T	F
YBR011C	F	F	F	F	F	F	F	F	F	F	F	F	F	F	F	F	F	T	F	F	F	T	F
YBR017C	F	F	F	F	F	F	F	F	F	F	F	F	F	F	F	F	F	T	F	F	F	T	F
YBR020W	F	F	F	F	F	F	F	F	F	F	F	F	F	F	F	F	F	F	F	F	F	F	F
YBR023C	F	F	F	F	F	F	F	F	F	F	F	F	F	T	F	F	F	F	F	F	F	F	F
YBR025C	F	F	F	F	F	F	F	F	F	F	F	F	F	F	F	F	F	T	F	F	F	F	F
YBR026C	F	T	F	F	F	F	F	F	F	F	F	F	F	F	F	F	F	F	F	F	F	F	F
YBR030W	F	F	F	F	F	F	F	F	F	F	F	F	F	F	F	F	F	F	F	F	F	T	F
YBR031W	F	F	F	F	F	F	F	F	F	F	F	F	F	F	F	F	F	T	F	F	F	F	F
YBR034C	F	F	F	F	F	F	F	F	F	F	F	F	F	F	F	F	F	T	F	F	F	T	F
YBR035C	F	F	F	F	F	F	F	F	F	F	F	F	F	F	F	F	F	F	F	F	F	F	F
YBR036C	F	F	T	F	F	F	F	T	F	F	F	F	F	F	F	F	F	F	F	F	F	F	F
YBR037C	F	T	F	F	F	F	F	F	F	F	F	F	F	F	F	F	F	F	F	F	F	F	F
YBR038W	F	F	F	F	F	F	F	F	F	F	F	F	F	F	F	F	F	F	F	F	F	F	F
YBR039W	F	T	F	F	F	F	F	F	F	F	F	F	F	F	F	F	F	F	F	F	F	F	F
YBR041W	F	F	F	F	F	F	F	F	F	F	F	F	F	F	F	F	F	F	F	F	T	F	F
YBR045C	F	F	F	F	F	F	F	F	F	F	F	F	F	F	F	F	F	F	F	F	F	F	F
YBR048W	F	F	F	F	F	F	F	F	F	F	F	F	F	F	F	F	F	T	F	F	F	F	F

YBR049C	F	F	F	F	F	F	F	F	F	F	F	F	F	F	F	F	F	F	F	F	F	T	F
YBR050C	F	F	F	F	F	F	F	F	F	F	F	F	F	F	F	F	F	F	F	F	F	F	F
YBR052C	F	F	F	F	F	T	F	F	F	F	F	F	F	F	F	F	F	F	F	F	F	F	F
YBR055C	F	F	F	F	F	F	F	F	F	F	F	F	F	F	F	F	F	T	F	F	F	T	F
YBR058C-A	F	F	F	F	F	F	F	T	F	F	F	F	F	F	F	F	F	F	F	F	F	F	F
YBR059C	F	F	F	F	T	F	F	F	F	F	T	F	F	F	F	F	F	T	F	F	F	F	F
YBR060C	F	F	F	F	F	F	F	F	F	F	F	F	F	F	F	F	F	T	F	F	F	T	F
YBR061C	F	F	F	F	F	F	F	F	F	F	F	F	F	F	F	F	F	T	F	F	F	F	F
YBR065C	F	F	F	F	F	F	F	F	F	F	F	F	F	F	F	F	F	F	F	F	F	T	F
YBR066C	F	F	F	F	F	F	F	F	F	F	F	F	F	F	F	F	F	T	F	F	F	T	F
YBR068C	F	F	T	F	T	F	F	F	F	F	F	F	F	F	F	F	F	F	F	F	F	F	F
YBR069C	F	F	F	F	T	F	F	F	F	F	F	F	F	F	F	F	F	F	F	F	F	F	T
YBR072W	T	F	F	F	F	F	F	F	F	F	F	F	F	F	F	F	F	F	F	F	F	F	F
YBR073W	F	F	F	F	F	F	F	F	F	F	F	F	F	F	F	F	F	F	F	F	F	T	F
YBR077C	F	F	F	F	F	F	T	F	F	F	F	F	F	F	F	F	F	F	F	F	F	F	F
YBR079C	F	F	F	F	F	F	F	F	F	F	F	F	F	F	F	F	F	T	F	F	F	F	F
YBR080C	F	F	F	F	F	F	F	F	F	F	F	F	T	F	F	F	F	F	F	T	F	F	F
YBR081C	F	F	F	F	F	F	F	F	F	F	F	F	F	F	F	F	F	F	F	F	F	T	F
YBR082C	F	F	F	F	F	F	F	F	F	F	F	F	F	F	F	F	F	T	F	F	F	T	F
YBR083W	F	F	F	F	F	F	F	F	F	F	F	F	F	F	F	F	F	T	F	F	F	T	F
YBR084W	F	T	F	F	F	F	F	F	F	F	F	F	F	F	F	F	F	F	F	F	F	F	F
YBR086C	F	F	F	F	T	F	F	F	F	F	F	F	F	F	F	F	F	F	F	F	F	F	F
YBR087W	F	F	F	F	F	F	F	F	F	F	F	F	F	F	F	F	F	F	F	F	F	T	F
YBR088C	F	F	F	F	F	F	F	F	F	F	F	F	F	F	F	F	F	F	F	F	F	T	F
YBR089C-A	F	F	F	F	F	F	F	F	F	F	F	F	F	F	F	F	F	F	F	F	F	T	F
YBR091C	F	F	F	F	F	F	F	F	F	F	F	F	F	F	F	F	F	F	F	F	F	F	F
YBR094W	F	F	F	F	F	F	F	F	F	F	F	F	F	F	F	F	F	T	F	F	F	F	F
YBR095C	F	F	F	F	F	F	F	F	F	F	F	F	F	F	F	F	F	F	F	F	F	T	F
YBR097W	F	F	F	F	F	F	F	F	F	T	F	F	F	F	F	F	F	F	F	F	F	F	F
YBR098W	F	F	F	F	F	F	F	F	F	F	F	F	F	F	F	F	F	F	F	F	F	F	F
YBR101C	F	F	F	F	F	F	F	F	F	F	F	F	F	F	F	F	F	T	F	F	F	F	F
YBR102C	T	F	F	F	T	F	F	F	F	F	T	F	F	F	F	F	F	F	F	F	F	F	T
YBR103W	F	F	F	F	F	F	F	F	F	F	F	F	F	F	F	F	F	F	F	F	F	T	F
YBR105C	F	F	F	F	F	F	F	F	F	F	F	F	F	F	F	F	F	T	F	F	F	T	F
YBR106W	F	F	F	F	F	F	F	T	F	F	F	F	F	F	F	F	F	F	F	F	F	F	F
YBR107C	F	F	F	T	F	F	F	F	F	F	F	F	F	F	F	F	F	F	F	F	F	T	F
YBR108W	F	F	F	F	F	F	F	F	F	F	F	F	F	F	F	F	F	F	F	F	F	F	F
YBR109C	F	F	F	F	T	F	F	F	F	F	T	F	F	F	F	F	F	F	F	F	F	F	T
YBR112C	F	F	F	F	F	F	F	F	F	F	F	F	F	F	F	F	F	F	F	F	F	T	F
YBR114W	F	F	F	F	F	F	F	F	F	F	F	F	F	F	F	F	F	T	F	F	F	T	F
YBR115C	F	F	F	F	F	F	F	F	F	F	F	F	F	F	F	F	F	T	F	F	F	F	F
YBR117C	F	F	F	F	F	F	F	F	F	F	F	F	F	F	F	F	F	T	F	F	F	T	F
YBR118W	F	F	F	F	F	F	F	F	F	F	F	F	F	F	F	F	F	T	F	F	F	F	F
YBR119W	F	F	F	F	F	F	F	F	F	F	F	F	F	F	F	F	F	F	F	F	F	T	F
YBR121C	F	F	F	F	F	F	F	F	F	F	F	F	F	F	F	F	F	T	F	F	F	F	F
YBR122C	F	T	F	F	F	F	F	F	F	F	F	F	F	F	F	F	F	F	F	F	F	F	F
YBR123C	F	F	F	F	F	F	F	F	F	F	F	F	F	F	F	F	F	F	F	F	F	T	F
YBR125C	F	F	F	F	F	F	F	F	F	F	F	F	F	F	F	F	F	T	F	F	F	F	F
YBR126C	F	F	F	F	F	F	F	F	F	F	F	F	F	F	F	F	F	T	F	F	F	F	F
YBR127C	F	F	F	F	F	F	T	F	F	F	F	F	F	F	F	F	F	F	F	F	F	F	F
YBR128C	F	F	F	F	F	F	F	F	F	F	F	F	F	F	F	F	F	F	F	F	F	F	F
YBR130C	T	F	F	F	T	F	F	F	F	F	F	F	F	F	F	F	F	T	F	F	F	F	T
YBR131W	F	F	F	F	F	F	F	F	F	T	F	F	F	F	F	F	F	F	F	F	F	F	F
YBR133C	F	F	F	F	F	F	F	F	F	F	T	F	F	F	F	F	F	T	F	F	F	F	F
YBR135W	F	F	F	F	F	F	F	F	F	F	F	F	F	F	F	F	F	T	F	F	F	T	F
YBR136W	F	F	F	F	F	F	F	F	F	F	F	F	F	F	F	F	F	F	F	F	F	F	F
YBR137W	F	F	F	F	F	F	F	F	F	F	F	F	F	F	F	F	F	T	F	F	F	F	F
YBR138C	F	F	F	F	F	F	F	F	F	F	F	F	F	F	F	F	F	T	F	F	F	F	F
YBR142W	F	F	F	F	F	F	F	F	F	F	F	F	F	F	F	F	T	F	F	F	F	T	F
YBR143C	F	F	F	F	F	F	F	F	F	F	F	F	F	F	F	F	F	T	F	F	F	F	F
YBR146W	F	F	F	F	F	F	F	F	F	F	F	F	F	F	F	F	F	F	F	F	F	F	F
YBR149W	F	F	F	F	F	F	F	F	F	F	F	F	F	F	F	F	F	T	F	F	F	T	F
YBR152W	F	F	F	F	F	F	F	F	F	F	F	F	F	F	F	F	F	F	F	F	F	T	F
YBR154C	F	F	F	F	F	F	F	F	F	F	F	F	F	F	F	F	F	F	F	F	F	F	F
YBR155W	F	F	F	F	F	F	F	F	F	F	F	F	F	F	F	F	F	T	F	F	F	F	F
YBR156C	F	F	F	F	F	F	F	F	F	F	F	T	F	F	F	F	F	F	F	F	F	T	F
YBR158W	F	F	F	F	F	F	F	F	F	F	F	F	F	F	F	F	F	T	F	F	F	T	F
YBR159W	F	F	F	F	F	F	F	T	F	F	F	F	F	F	F	F	F	F	F	F	F	F	F

YBR160W	F	F	F	F	F	F	F	F	F	F	F	F	F	F	F	F	F	T	F	F	F	T	F
YBR161W	F	F	T	F	F	F	F	F	F	F	F	F	F	F	F	F	F	F	F	F	F	F	F
YBR164C	F	F	F	F	F	T	F	F	F	F	F	F	F	F	F	F	F	F	F	T	F	F	F
YBR167C	F	F	F	F	F	F	F	F	F	F	F	F	F	F	F	F	F	F	F	F	F	T	F
YBR168W	T	F	F	F	F	F	F	F	F	F	F	F	F	F	F	F	F	F	F	F	F	F	F
YBR169C	F	F	F	F	F	F	F	F	F	F	F	F	F	F	F	F	F	T	F	F	F	F	F
YBR170C	F	F	F	F	F	F	F	F	F	F	F	F	F	F	F	F	F	T	F	F	F	T	F
YBR171W	F	F	F	F	F	F	F	T	F	F	F	F	F	F	F	F	F	F	F	F	F	F	F
YBR172C	F	F	F	F	F	F	F	F	F	F	F	F	F	F	F	F	F	T	F	F	F	F	F
YBR173C	F	F	F	F	F	F	F	F	F	F	F	F	F	F	F	F	F	T	F	F	F	T	F
YBR175W	F	F	F	F	F	F	F	F	F	F	F	F	F	F	F	F	F	T	F	F	F	T	F
YBR177C	F	F	F	F	F	F	F	T	F	F	F	F	F	F	F	F	F	F	F	F	F	F	F
YBR179C	F	T	F	F	F	F	F	F	F	F	F	F	F	F	F	F	F	F	F	F	F	F	F
YBR181C	F	F	F	F	F	F	F	F	F	F	F	F	F	F	F	F	F	T	F	F	F	F	F
YBR182C	F	F	F	F	F	F	F	F	F	F	F	F	F	F	F	F	F	F	F	F	F	F	F
YBR185C	F	T	F	F	F	F	F	F	F	F	F	F	F	F	F	F	F	F	F	F	F	F	F
YBR188C	F	F	F	F	F	F	F	F	F	F	F	F	F	F	F	F	F	F	F	F	F	T	F
YBR189W	F	F	F	F	F	F	F	F	F	F	F	F	F	F	F	F	F	T	F	F	F	F	F
YBR191W	F	F	F	F	F	F	F	F	F	F	F	F	F	F	F	F	F	T	F	F	F	F	F
YBR193C	T	F	F	F	F	F	F	F	F	F	F	F	F	F	F	F	F	F	F	F	F	F	F
YBR195C	F	F	F	F	F	F	F	F	F	F	F	F	F	F	F	F	F	T	F	F	F	T	F
YBR196C	F	F	F	F	F	F	F	F	F	F	F	F	F	F	F	F	F	T	F	F	F	F	F
YBR198C	F	F	F	F	F	F	F	F	F	F	F	F	F	F	F	F	F	F	F	F	F	T	F
YBR200W	T	F	F	F	T	F	F	F	F	F	T	F	F	F	F	F	F	F	F	F	F	F	T
YBR201W	F	F	F	F	F	F	F	F	F	F	F	F	F	F	F	F	F	F	F	F	F	F	F
YBR202W	F	F	F	F	F	F	F	F	F	F	F	F	F	F	F	F	F	T	F	F	F	T	F
YBR205W	F	F	T	F	F	F	F	F	F	F	F	F	F	F	F	F	F	F	F	F	F	F	F
YBR208C	F	F	F	F	F	F	F	F	F	F	F	F	F	F	F	F	F	T	F	F	F	F	F
YBR211C	F	F	F	T	F	F	F	F	F	F	F	F	F	F	F	F	F	F	F	F	F	F	F
YBR215W	F	F	F	F	F	F	F	F	F	F	F	F	F	F	F	F	F	F	F	F	F	T	F
YBR216C	F	F	F	F	F	F	F	F	F	F	F	F	F	F	F	F	F	T	F	F	F	F	F
YBR217W	F	F	F	F	F	F	F	F	F	F	F	F	F	F	F	F	F	F	F	F	F	F	F
YBR221C	F	T	F	F	F	F	F	F	F	F	F	F	F	F	F	F	F	F	F	F	F	F	F
YBR223C	F	F	F	F	F	F	F	F	F	F	F	F	F	F	F	F	F	T	F	F	F	T	F
YBR225W	T	F	F	F	F	F	F	F	F	F	F	F	F	F	F	F	F	F	F	F	F	F	F
YBR228W	F	F	F	F	F	F	F	F	F	F	F	F	F	F	F	F	F	F	F	F	F	F	F
YBR229C	F	F	F	F	F	F	F	F	F	F	F	F	F	F	F	F	F	F	F	F	F	F	F
YBR231C	F	F	F	F	F	F	F	F	F	F	F	F	F	F	F	F	F	F	F	F	F	T	F
YBR233W-A	F	F	F	T	F	F	F	F	F	F	F	F	F	F	F	F	F	F	F	F	F	F	F
YBR234C	F	F	F	F	F	F	F	F	F	F	F	F	F	F	F	T	F	F	F	F	F	F	F
YBR236C	F	F	F	F	F	F	F	F	F	F	F	F	F	F	F	F	F	F	F	F	F	T	F
YBR239C	F	F	F	F	F	F	F	F	F	F	F	F	F	F	F	F	F	T	F	F	F	T	F
YBR245C	F	F	F	F	F	F	F	F	F	F	F	F	F	F	F	F	F	F	F	F	F	T	F
YBR247C	F	F	F	F	F	F	F	F	F	F	F	F	F	F	F	F	T	T	F	F	F	T	F
YBR248C	F	F	F	F	F	F	F	F	F	F	F	F	F	F	F	F	F	T	F	F	F	T	F
YBR249C	F	F	F	F	F	F	F	F	F	F	F	F	F	F	F	F	F	T	F	F	F	T	F
YBR251W	F	T	F	F	F	F	F	F	F	F	F	F	F	F	F	F	F	F	F	F	F	F	F
YBR253W	F	F	F	F	F	F	F	F	F	F	F	F	F	F	F	F	F	F	F	F	F	T	F
YBR254C	F	F	F	F	F	F	F	F	F	F	F	F	F	T	F	F	F	F	F	F	F	F	F
YBR257W	F	F	F	F	F	F	F	F	F	F	F	F	F	F	F	F	F	F	F	F	F	F	F
YBR258C	F	F	F	F	F	F	F	F	F	F	F	F	F	F	F	F	F	F	F	F	F	T	F
YBR260C	T	F	F	F	F	F	F	F	F	F	T	F	F	F	F	F	F	T	F	F	F	F	T
YBR263W	F	T	F	F	F	F	F	F	F	F	F	F	F	F	F	F	F	F	F	F	F	F	F
YBR264C	F	F	F	F	F	F	F	F	F	F	F	F	F	F	F	F	F	T	F	F	F	F	F
YBR265W	F	F	F	F	F	F	F	T	F	F	F	F	F	F	F	F	F	F	F	F	F	F	F
YBR267W	F	F	F	F	F	F	F	F	F	F	F	F	F	F	F	F	F	T	F	F	F	F	F
YBR270C	F	F	F	F	F	F	F	F	F	F	F	F	F	F	F	F	F	F	F	F	F	F	F
YBR272C	F	F	F	F	F	F	F	F	F	F	F	F	F	F	F	F	F	T	F	F	F	F	F
YBR273C	F	F	F	F	F	F	F	T	F	F	F	F	F	F	F	F	F	F	F	F	F	F	F
YBR274W	F	F	F	F	F	F	F	F	F	F	F	F	F	F	F	F	F	T	F	F	F	F	F
YBR275C	F	F	F	F	F	F	F	F	F	F	F	F	F	F	F	F	F	F	F	F	F	T	F
YBR278W	F	F	F	F	F	F	F	F	F	F	F	F	F	F	F	F	F	T	F	F	F	T	F
YBR279W	F	F	F	F	F	F	F	F	F	F	F	F	F	F	F	F	F	F	F	F	F	T	F
YBR280C	F	F	F	F	F	F	F	F	F	F	F	F	F	F	F	F	F	F	F	F	F	F	F
YBR281C	F	F	F	F	F	F	F	F	F	F	F	F	F	F	F	F	F	T	F	F	F	T	F
YBR282W	F	T	F	F	F	F	F	F	F	F	F	F	F	F	F	F	F	F	F	F	F	F	F
YBR283C	F	F	F	F	F	F	F	T	F	F	F	F	F	F	F	F	F	F	F	F	F	F	F
YBR285W	F	F	F	F	F	F	F	F	F	F	F	F	F	F	F	F	F	F	F	F	F	F	F

YBR288C	F	F	F	F	F	T	F	F	F	F	F	F	F	F	F	F	F	F	F	T	F	F	F
YBR289W	F	F	F	F	F	F	F	F	F	F	F	F	F	F	F	F	F	F	F	F	F	T	F
YBR290W	F	F	T	F	F	F	F	T	F	F	F	F	F	F	F	F	F	F	F	F	F	F	F
YCL008C	F	F	F	F	F	F	F	F	F	F	F	F	F	F	F	F	F	F	F	F	F	F	F
YCL010C	F	F	F	F	F	F	F	F	F	F	F	F	F	F	F	F	F	F	F	F	F	T	F
YCL011C	F	F	F	F	F	F	F	F	F	F	F	F	F	F	F	F	F	F	F	F	F	T	F
YCL014W	F	F	F	F	F	F	F	F	F	F	T	F	F	F	F	F	F	F	F	F	F	F	F
YCL016C	F	F	F	F	F	F	F	F	F	F	F	F	F	F	F	F	F	F	F	F	F	T	F
YCL017C	F	T	F	F	F	F	F	F	F	F	F	F	F	F	F	F	F	F	F	F	F	F	F
YCL024W	T	F	F	F	T	F	F	F	F	F	T	F	F	F	F	F	F	F	F	F	F	F	T
YCL027W	F	F	T	F	F	F	F	F	F	F	F	F	F	F	F	F	F	F	F	F	F	F	F
YCL028W	F	F	F	F	F	F	F	F	F	F	F	F	F	F	F	F	F	T	F	F	F	T	F
YCL029C	F	F	F	T	F	F	F	F	F	F	F	T	F	F	F	F	F	F	F	F	F	F	F
YCL030C	F	F	F	F	F	F	F	F	F	F	F	F	F	F	F	F	F	T	F	F	F	F	F
YCL031C	F	F	F	F	F	F	F	F	F	F	F	F	F	F	F	F	T	F	F	F	F	T	F
YCL032W	F	F	F	F	F	F	F	F	F	F	F	F	F	F	F	F	F	T	F	F	F	F	F
YCL034W	F	F	F	F	F	F	F	F	F	F	F	F	F	F	F	F	F	T	F	F	F	T	F
YCL037C	F	F	F	F	F	F	F	F	F	F	F	F	F	F	F	F	F	T	F	F	F	F	F
YCL039W	F	F	F	F	F	F	F	F	F	F	F	F	F	F	F	F	F	T	F	F	F	T	F
YCL040W	F	F	F	F	F	T	F	F	F	F	F	F	F	F	F	F	F	T	F	F	F	F	F
YCL044C	F	T	F	F	F	F	F	F	F	F	F	F	F	F	F	F	F	F	F	F	F	F	F
YCL050C	F	F	F	F	F	F	F	F	F	F	F	F	F	F	F	F	F	T	F	F	F	T	F
YCL051W	F	F	F	F	F	F	F	F	F	F	F	F	F	F	F	F	F	F	F	F	F	F	F
YCL054W	F	F	F	F	F	F	F	F	F	F	F	F	F	F	F	F	F	F	F	F	F	F	F
YCL057W	F	F	F	F	F	F	F	F	F	F	F	F	F	F	F	F	F	T	F	F	F	F	F
YCL059C	F	F	F	F	F	F	F	F	F	F	F	F	F	F	F	F	T	T	F	F	F	F	F
YCL061C	F	F	F	F	F	F	F	F	F	F	F	F	F	F	F	F	F	F	F	F	F	T	F
YCL063W	F	F	F	F	F	F	F	F	F	F	F	F	F	F	F	F	F	F	F	F	F	F	F
YCR002C	F	F	F	F	T	F	F	F	F	F	T	F	F	F	F	F	F	F	F	F	F	F	F
YCR003W	F	T	F	F	F	F	F	F	F	F	F	F	F	F	F	F	F	F	F	F	F	F	F
YCR004C	F	F	F	F	F	T	F	F	F	F	F	F	F	F	F	F	F	T	F	F	F	F	F
YCR005C	F	F	F	F	F	F	F	F	F	F	F	F	F	F	F	F	F	T	F	F	F	F	F
YCR008W	F	F	F	F	F	F	F	F	F	F	F	F	F	F	F	F	F	F	F	F	F	F	F
YCR009C	F	F	F	F	F	F	F	F	F	F	F	F	F	F	F	T	F	T	F	F	F	F	F
YCR012W	F	F	F	F	F	F	F	F	F	F	F	F	F	F	F	F	F	T	F	F	F	T	F
YCR015C	F	F	F	F	F	F	F	F	F	F	F	F	F	F	F	F	F	F	F	F	F	F	F
YCR020W-B	F	F	F	F	F	F	F	F	F	F	F	F	F	F	F	F	F	F	F	F	F	T	F
YCR027C	F	F	F	F	F	F	F	F	F	F	F	F	F	F	F	F	F	T	F	F	F	F	F
YCR028C-A	F	T	F	F	F	F	F	F	F	F	F	F	F	F	F	F	F	F	F	F	F	F	F
YCR030C	F	F	F	F	T	T	F	F	F	F	T	F	F	F	F	F	F	F	F	F	F	F	F
YCR031C	F	F	F	F	F	F	F	F	F	F	F	F	F	F	F	F	F	F	F	F	F	F	F
YCR033W	F	F	F	F	F	F	F	F	F	F	F	F	F	F	F	F	F	F	F	F	F	T	F
YCR034W	F	F	F	F	F	F	F	T	F	F	F	F	F	F	F	F	F	F	F	F	F	F	F
YCR035C	F	F	F	F	F	F	F	F	F	F	F	F	F	F	F	F	T	F	F	F	F	T	F
YCR038C	F	F	F	F	T	F	F	F	F	F	T	F	F	F	F	F	F	F	F	F	F	F	F
YCR039C	F	F	F	F	F	F	F	F	F	F	F	F	F	F	F	F	F	F	F	F	F	F	F
YCR042C	F	F	F	F	F	F	F	F	F	F	F	F	F	F	F	F	F	F	F	F	F	T	F
YCR046C	F	T	F	F	F	F	F	F	F	F	F	F	F	F	F	F	F	F	F	F	F	F	F
YCR052W	F	F	F	F	F	F	F	F	F	F	F	F	F	F	F	F	F	F	F	F	F	F	F
YCR057C	F	F	F	F	F	F	F	F	F	F	F	F	F	F	F	F	T	F	F	F	F	F	F
YCR059C	F	F	F	F	F	F	F	F	F	F	F	F	F	F	F	F	F	T	F	F	F	T	F
YCR060W	F	F	F	F	F	F	F	F	F	F	F	F	F	F	F	F	F	T	F	F	F	T	F
YCR063W	F	F	F	F	F	F	F	F	F	F	F	F	F	F	F	F	F	F	F	F	F	T	F
YCR065W	F	F	F	F	F	F	F	F	F	F	F	F	F	F	F	F	F	T	F	F	F	T	F
YCR066W	F	F	F	F	F	F	F	F	F	F	F	F	F	F	F	F	F	F	F	F	F	F	F
YCR067C	F	F	F	F	F	F	F	T	F	F	F	F	F	F	F	F	F	F	F	F	F	F	F
YCR071C	F	T	F	F	F	F	F	F	F	F	F	F	F	F	F	F	F	F	F	F	F	F	F
YCR072C	F	F	F	F	F	F	F	F	F	F	F	F	F	F	F	F	F	F	F	F	F	F	F
YCR073C	F	F	F	F	F	F	F	F	F	F	F	F	F	F	F	F	F	F	F	F	F	F	F
YCR073W-A	F	F	F	F	F	F	F	F	F	F	F	F	F	F	F	F	F	T	F	F	F	F	F
YCR076C	F	F	F	F	F	F	F	F	F	F	F	F	F	F	F	F	F	F	F	F	F	F	F
YCR077C	F	F	F	F	F	F	F	F	F	F	F	F	F	F	F	F	F	T	F	F	F	F	F
YCR079W	F	T	F	F	F	F	F	F	F	F	F	F	F	F	F	F	F	F	F	F	F	F	F
YCR081W	F	F	F	F	F	F	F	F	F	F	F	F	F	F	F	F	F	T	F	F	F	F	F
YCR082W	F	F	F	F	F	F	F	F	F	F	F	F	F	F	F	F	F	T	F	F	F	T	F
YCR084C	F	F	F	F	F	F	F	F	F	F	F	F	F	F	F	F	F	F	F	F	F	T	F
YCR086W	F	F	F	F	F	F	F	F	F	F	F	F	F	F	F	F	T	F	F	F	F	F	F
YCR088W	F	F	F	F	F	F	F	F	F	F	F	F	F	F	F	T	F	F	F	F	F	F	F

YCR092C	F	F	F	F	F	F	F	F	F	F	F	F	F	F	F	F	F	T	F	F	F	T	F
YCR093W	F	F	F	F	F	F	F	F	F	F	F	F	F	F	F	F	F	T	F	F	F	F	F
YCR094W	F	F	F	F	F	F	F	F	F	F	F	F	F	T	F	F	F	F	F	F	F	F	F
YCR095C	F	F	F	F	F	F	F	F	F	F	F	F	F	F	F	F	F	T	F	F	F	F	F
YCR097W	F	F	F	F	F	F	F	F	F	F	F	F	F	F	F	F	F	F	F	F	F	F	F
YDL002C	F	F	F	F	F	F	F	F	F	F	F	F	F	F	F	F	F	F	F	F	F	T	F
YDL003W	F	F	F	F	F	F	F	F	F	F	F	F	F	F	F	F	F	T	F	F	F	T	F
YDL004W	F	F	F	F	F	F	F	F	F	F	F	F	F	F	F	F	F	F	F	F	F	F	F
YDL005C	F	F	F	F	F	F	F	F	F	F	F	F	F	F	F	F	F	F	F	F	F	T	F
YDL006W	F	F	F	F	F	F	F	F	F	F	F	F	F	F	F	F	F	F	F	F	F	F	F
YDL007W	F	F	F	F	F	F	F	F	F	F	F	F	F	F	F	F	F	F	F	F	F	F	F
YDL008W	F	F	F	F	F	F	T	F	F	F	F	F	F	F	F	F	F	F	F	F	F	F	F
YDL013W	F	F	F	F	F	F	F	F	F	F	F	F	F	F	F	F	F	F	F	F	F	F	F
YDL014W	F	F	F	F	F	F	F	F	F	F	F	F	F	F	F	F	F	F	F	F	F	F	F
YDL015C	F	F	F	F	F	F	F	T	F	F	F	F	F	F	F	F	F	F	F	F	F	F	F
YDL017W	F	F	F	F	F	F	F	F	F	F	F	F	F	F	F	F	F	T	F	F	F	T	F
YDL018C	F	F	F	F	F	F	F	T	F	F	F	F	F	F	F	F	F	F	F	F	F	F	F
YDL019C	F	F	F	F	F	T	F	F	F	F	F	F	F	F	F	F	F	F	F	F	F	F	F
YDL020C	F	F	F	F	F	F	F	F	F	F	F	F	F	F	F	F	F	T	F	F	F	T	F
YDL022W	F	F	F	F	F	F	F	F	F	F	F	F	F	F	T	F	F	F	F	F	F	F	F
YDL028C	F	F	F	F	F	F	F	F	F	F	T	F	F	F	F	F	F	F	F	F	F	F	F
YDL029W	F	F	F	F	F	T	F	F	F	F	F	F	F	F	F	F	F	F	F	F	F	F	F
YDL030W	F	F	F	F	F	F	F	F	F	F	F	F	F	F	F	F	F	F	F	F	F	T	F
YDL031W	F	F	F	F	F	F	F	F	F	F	F	F	F	F	F	F	F	F	F	F	F	F	F
YDL035C	F	F	F	F	F	F	F	F	F	F	F	F	F	F	F	F	F	F	F	F	F	F	F
YDL040C	F	F	F	F	F	F	F	F	F	F	F	F	F	F	F	F	F	T	F	F	F	F	F
YDL042C	F	F	F	F	F	F	F	F	T	F	F	F	F	F	F	F	F	F	F	F	F	F	F
YDL043C	F	F	F	F	F	F	F	F	F	F	F	F	F	F	F	F	F	F	F	F	F	T	F
YDL044C	F	T	F	F	F	F	F	F	F	F	F	F	F	F	F	F	F	F	F	F	F	F	F
YDL045W-A	F	T	F	F	F	F	F	F	F	F	F	F	F	F	F	F	F	F	F	F	F	F	F
YDL047W	F	F	F	F	F	F	F	F	F	F	F	F	F	F	F	F	F	T	F	F	F	T	F
YDL051W	F	F	F	F	F	F	F	F	F	F	F	F	F	F	F	F	F	F	F	F	F	T	F
YDL052C	F	F	F	F	F	F	F	T	F	F	F	F	F	F	F	F	F	F	F	F	F	F	F
YDL053C	F	F	F	F	F	F	F	F	F	F	F	F	F	F	F	F	F	T	F	F	F	T	F
YDL055C	F	F	F	F	F	F	F	F	F	F	F	F	F	F	F	F	F	T	F	F	F	F	F
YDL056W	F	F	F	F	F	F	F	F	F	F	F	F	F	F	F	F	F	F	F	F	F	T	F
YDL058W	F	F	F	F	F	T	F	F	F	F	F	F	F	F	F	F	F	F	F	F	F	F	F
YDL059C	F	F	F	F	F	F	F	F	F	F	F	F	F	F	F	F	F	T	F	F	F	T	F
YDL060W	F	F	F	F	F	F	F	F	F	F	F	F	F	F	F	F	F	T	F	F	F	T	F
YDL064W	F	F	F	F	F	F	F	F	F	F	F	F	F	F	F	F	F	T	F	F	F	T	F
YDL065C	F	F	F	F	F	F	F	F	F	F	F	F	F	F	F	F	F	T	F	F	F	F	F
YDL066W	F	T	F	F	F	F	F	F	F	F	F	F	F	F	F	F	F	F	F	F	F	F	F
YDL070W	F	F	F	F	F	F	F	F	F	F	F	F	F	F	F	F	F	T	F	F	F	T	F
YDL072C	F	F	F	F	F	F	F	T	F	F	F	F	F	F	F	F	F	F	F	F	F	F	F
YDL073W	F	F	F	F	F	F	F	F	F	F	F	F	F	F	F	F	F	F	F	F	F	F	F
YDL074C	F	F	F	F	F	F	F	F	F	F	F	F	F	F	F	F	F	F	F	F	F	T	F
YDL075W	F	F	F	F	F	F	F	F	F	F	F	F	F	F	F	F	F	F	F	F	F	F	F
YDL076C	F	F	F	F	F	F	F	F	F	F	F	F	F	F	F	F	F	F	F	F	F	T	F
YDL077C	T	F	F	F	F	F	F	F	F	F	F	F	F	F	F	F	F	F	F	F	F	F	F
YDL078C	F	F	F	F	F	F	F	F	F	F	F	F	F	F	F	F	F	T	F	F	F	T	F
YDL081C	F	F	F	F	F	F	F	F	F	F	F	F	F	F	F	F	F	T	F	F	F	F	F
YDL082W	F	F	F	F	F	F	F	F	F	F	F	F	F	F	F	F	F	T	F	F	F	F	F
YDL083C	F	F	F	F	F	F	F	F	F	F	F	F	F	F	F	F	F	T	F	F	F	F	F
YDL084W	F	F	F	F	F	F	F	F	F	F	F	F	F	F	F	F	F	F	F	F	F	T	F
YDL087C	F	F	F	F	F	F	F	F	F	F	F	F	F	F	F	F	F	F	F	F	F	T	F
YDL088C	F	F	F	F	F	F	F	F	T	F	F	F	F	F	F	F	F	F	F	F	F	F	F
YDL089W	T	F	F	F	F	F	F	F	T	F	F	F	F	F	F	F	F	F	F	F	F	F	F
YDL091C	F	F	F	F	F	T	F	F	F	F	F	F	F	F	F	F	F	F	F	F	F	F	F
YDL092W	F	F	F	F	F	F	F	T	F	F	F	F	F	F	F	F	F	F	F	F	F	F	F
YDL095W	F	F	F	F	F	F	F	T	F	F	F	F	F	F	F	F	F	F	F	F	F	F	F
YDL097C	F	F	F	F	F	F	F	F	F	F	F	F	F	F	F	F	F	T	F	F	F	T	F
YDL098C	F	F	F	F	F	F	F	F	F	F	F	F	F	F	F	F	F	F	F	F	F	T	F
YDL099W	F	F	F	F	F	T	F	F	F	F	F	F	F	F	F	F	F	F	F	T	F	F	F
YDL100C	F	F	F	F	F	F	F	T	F	F	F	F	F	F	F	F	F	F	F	F	F	F	F
YDL101C	F	F	F	F	F	F	F	F	F	F	F	F	F	F	F	F	F	F	F	F	F	T	F
YDL102W	F	F	F	F	F	F	F	F	F	F	F	F	F	F	F	F	F	F	F	F	F	T	F
YDL103C	F	F	F	F	F	F	F	F	F	F	F	F	F	F	F	F	F	T	F	F	F	T	F
YDL105W	F	F	F	F	F	F	F	F	F	F	F	F	F	F	F	F	F	F	F	F	F	T	F

YDL106C	F	F	F	F	F	F	F	F	F	F	F	F	F	F	F	F	F	F	F	F	F	T	F
YDL108W	F	F	F	F	F	F	F	F	F	F	F	F	F	F	F	F	F	F	F	F	F	T	F
YDL111C	F	F	F	F	F	F	F	F	F	F	F	F	F	F	F	F	T	T	F	F	F	T	F
YDL112W	F	F	F	F	F	F	F	F	F	F	F	F	F	F	F	F	F	T	F	F	F	F	F
YDL113C	F	F	F	F	F	F	F	F	F	T	F	F	F	F	F	F	F	F	F	F	F	F	F
YDL115C	F	F	F	F	F	F	F	F	F	F	F	F	F	F	F	F	F	T	F	F	F	T	F
YDL116W	F	F	F	F	F	F	F	F	T	F	F	F	F	F	F	F	F	F	F	F	F	F	F
YDL117W	F	F	F	F	F	F	F	F	F	F	T	F	F	F	F	F	F	T	F	F	F	F	F
YDL120W	F	T	F	F	F	F	F	F	F	F	F	F	F	F	F	F	F	F	F	F	F	F	F
YDL122W	F	F	F	F	F	F	F	F	F	F	F	F	F	F	F	F	F	T	F	F	F	F	F
YDL123W	F	F	F	F	F	F	T	F	F	F	F	F	F	F	F	F	F	F	F	F	F	F	F
YDL125C	F	F	F	F	F	F	F	F	F	F	F	F	F	F	F	F	F	T	F	F	F	T	F
YDL126C	F	F	F	F	F	F	F	F	F	F	F	F	F	F	F	F	F	T	F	F	F	T	F
YDL127W	F	F	F	F	F	F	F	F	F	F	F	F	F	F	F	F	F	F	F	F	F	F	F
YDL130W	F	F	F	F	F	F	F	F	F	F	F	F	F	F	F	F	F	T	F	F	F	F	F
YDL131W	F	F	F	F	F	F	F	F	F	F	F	F	F	F	F	F	F	F	F	F	F	T	F
YDL132W	F	F	F	F	F	F	F	F	F	F	F	F	F	F	F	F	F	T	F	F	F	T	F
YDL134C	F	F	F	F	F	F	F	F	F	F	F	F	F	F	F	F	F	T	F	F	F	T	F
YDL135C	F	F	F	F	F	F	F	F	F	F	F	F	F	F	F	F	F	T	F	F	F	T	F
YDL136W	F	F	F	F	F	F	F	F	F	F	F	F	F	F	F	F	F	T	F	F	F	F	F
YDL137W	F	F	F	F	F	F	F	F	F	F	F	F	F	T	F	F	F	F	F	T	F	F	F
YDL139C	F	F	F	F	F	F	F	F	F	F	F	F	F	F	F	F	F	T	F	F	F	T	F
YDL140C	F	F	F	F	F	F	F	F	F	F	F	F	F	F	F	F	F	F	F	F	F	T	F
YDL143W	F	F	F	F	F	F	F	F	F	F	F	F	F	F	F	F	F	F	F	F	F	F	F
YDL145C	F	F	F	F	F	F	F	F	F	F	F	F	T	F	F	F	F	F	F	T	F	F	F
YDL146W	T	F	F	F	T	F	F	F	F	F	T	F	F	F	F	F	F	T	F	F	F	F	T
YDL147W	F	F	F	F	F	F	F	F	F	F	F	F	F	F	F	F	F	F	F	F	F	T	F
YDL148C	F	F	F	F	F	F	F	F	F	F	F	F	F	F	F	F	F	F	F	F	F	F	F
YDL149W	F	F	F	F	F	F	F	F	F	F	F	F	F	F	F	F	F	F	F	F	F	F	F
YDL150W	F	F	F	F	F	F	F	F	F	F	F	F	F	F	F	F	F	F	F	F	F	T	F
YDL153C	F	F	F	F	F	F	F	F	F	F	F	F	F	F	F	F	F	F	F	F	F	F	F
YDL155W	F	F	F	F	F	F	F	F	F	F	F	F	F	F	F	F	F	T	F	F	F	T	F
YDL156W	F	F	F	F	F	F	F	F	F	F	F	F	F	F	F	F	F	T	F	F	F	T	F
YDL159W	F	F	F	F	F	F	F	F	F	F	F	F	F	F	F	F	F	T	F	F	F	F	F
YDL160C	F	F	F	F	F	F	F	F	F	F	F	F	F	F	F	F	F	T	F	F	F	F	F
YDL161W	F	F	F	F	F	F	F	F	F	F	F	F	F	F	F	T	F	F	F	F	F	F	F
YDL164C	F	T	F	F	F	F	F	F	F	F	F	F	F	F	F	F	F	F	F	F	F	T	F
YDL165W	F	F	F	F	F	F	F	F	F	F	F	F	F	F	F	F	F	T	F	F	F	F	F
YDL166C	F	F	F	F	F	F	F	F	F	F	F	F	F	F	F	F	F	T	F	F	F	T	F
YDL171C	T	F	F	F	F	F	F	F	F	F	F	F	F	F	F	F	F	F	F	F	F	F	F
YDL175C	F	F	F	F	F	F	F	F	F	F	F	F	F	F	F	F	F	F	F	F	F	T	F
YDL176W	T	F	F	F	F	F	F	F	F	F	F	F	F	F	F	F	F	F	F	F	F	F	F
YDL178W	F	T	F	F	F	F	F	F	F	F	F	F	F	F	F	F	F	F	F	F	F	F	F
YDL179W	F	F	F	F	F	F	F	F	F	F	F	F	F	F	F	F	F	F	F	F	F	F	F
YDL181W	F	T	F	F	F	F	F	F	F	F	F	F	F	F	F	F	F	F	F	F	F	F	F
YDL182W	F	F	F	F	F	F	F	F	F	F	F	F	F	F	F	F	F	F	F	F	F	T	F
YDL184C	F	F	F	F	F	F	F	F	F	F	F	F	F	F	F	F	F	T	F	F	F	F	F
YDL185W	F	F	F	F	F	F	T	F	F	F	F	F	F	F	F	F	F	F	F	F	F	F	F
YDL188C	F	F	F	F	F	F	F	F	F	F	F	F	F	F	F	F	F	T	F	F	F	T	F
YDL190C	F	F	F	F	F	F	F	F	F	F	F	F	F	F	F	F	F	T	F	F	F	T	F
YDL191W	F	F	F	F	F	F	F	F	F	F	F	F	F	F	F	F	F	T	F	F	F	F	F
YDL192W	F	F	F	F	F	T	F	F	F	F	F	F	F	T	F	F	F	F	F	T	F	F	F
YDL194W	F	F	F	F	F	F	F	F	F	F	F	F	F	F	F	F	F	F	F	F	F	F	F
YDL195W	F	F	F	F	F	F	F	F	F	F	F	F	F	F	F	F	F	F	T	F	F	F	F
YDL199C	F	F	F	F	F	F	F	F	F	F	F	F	F	F	F	F	F	F	F	F	F	F	F
YDL200C	F	F	F	F	F	F	F	F	F	F	F	F	F	F	F	F	F	F	F	F	F	F	F
YDL203C	T	F	F	F	F	F	F	F	F	F	F	F	F	F	F	F	F	F	F	F	F	F	F
YDL207W	F	F	F	F	F	F	F	F	T	F	F	F	F	F	F	F	F	F	F	F	F	F	F
YDL208W	F	F	F	F	F	F	F	F	F	F	F	F	F	F	F	F	T	F	F	F	F	F	F
YDL209C	F	F	F	F	F	F	F	F	F	F	F	F	F	F	F	F	F	F	F	F	F	T	F
YDL212W	F	F	F	F	F	F	F	T	F	F	F	F	F	F	F	F	F	F	F	F	F	F	F
YDL213C	F	F	F	F	F	F	F	F	F	F	F	F	F	F	F	F	T	F	F	F	F	F	F
YDL215C	F	F	F	F	F	F	F	F	F	F	F	F	F	F	F	F	F	T	F	F	F	F	F
YDL216C	F	F	F	F	F	F	F	F	F	F	F	F	F	F	F	F	F	T	F	F	F	F	F
YDL217C	F	F	F	F	F	F	F	F	F	F	F	F	F	F	F	F	F	F	F	F	F	F	F
YDL220C	F	F	F	F	F	F	F	F	F	F	F	F	F	F	F	F	F	T	F	F	F	T	F
YDL225W	F	F	F	F	T	F	F	F	F	F	T	F	F	F	F	F	F	F	F	F	F	F	F
YDL226C	F	F	F	F	F	F	F	F	F	F	F	F	F	F	F	F	F	T	F	F	F	F	F

YDL227C	F	F	F	F	F	F	F	F	F	F	F	F	F	F	F	F	F	F	F	F	F	T	F
YDL229W	F	F	F	F	F	F	F	F	F	F	F	F	F	F	F	F	F	T	F	F	F	F	F
YDL232W	F	F	F	F	F	F	F	T	F	F	F	F	F	F	F	F	F	F	F	F	F	F	F
YDL235C	F	F	F	F	F	F	F	F	F	F	F	F	F	F	F	F	F	T	F	F	F	T	F
YDL239C	F	F	F	F	F	F	F	F	F	F	F	F	F	F	F	F	F	F	F	F	F	F	F
YDL240W	F	F	F	F	T	F	F	F	F	F	T	F	F	F	F	F	F	T	F	F	F	F	F
YDR001C	F	F	F	F	F	F	F	F	F	F	F	F	F	F	F	F	F	T	F	F	F	F	F
YDR002W	F	F	F	F	F	F	F	F	F	F	F	F	F	F	F	F	F	F	F	F	F	F	F
YDR003W	F	F	T	F	F	F	F	F	F	F	F	F	F	F	F	F	F	T	F	F	F	F	F
YDR004W	F	F	F	F	F	F	F	F	F	F	F	F	F	F	F	F	F	T	F	F	F	T	F
YDR005C	F	F	F	F	F	F	F	F	F	F	F	F	F	F	F	F	F	T	F	F	F	T	F
YDR006C	F	F	F	F	F	F	F	F	F	F	F	F	F	F	F	F	F	F	F	F	F	T	F
YDR009W	F	F	F	F	F	F	F	F	F	F	F	F	F	F	F	F	F	T	F	F	F	F	F
YDR012W	F	F	F	F	F	F	F	F	F	F	F	F	F	F	F	F	F	T	F	F	F	F	F
YDR013W	F	F	F	F	F	F	F	F	F	F	F	F	F	F	F	F	F	F	F	F	F	F	F
YDR014W	F	F	F	F	F	F	F	F	F	F	F	F	F	F	F	F	F	F	F	F	F	T	F
YDR016C	F	F	F	T	F	F	F	F	F	F	F	F	F	F	F	F	F	F	F	F	F	F	F
YDR017C	F	F	F	F	F	F	F	F	F	F	F	F	F	F	F	F	F	T	F	F	F	F	F
YDR020C	F	F	F	F	F	F	F	F	F	F	F	F	F	F	F	F	F	T	F	F	F	T	F
YDR022C	F	F	F	F	F	T	F	F	F	F	F	F	F	F	F	F	F	F	F	F	F	F	F
YDR023W	F	F	F	F	F	F	F	F	F	F	F	F	F	F	F	F	F	T	F	F	F	F	F
YDR025W	F	F	F	F	F	F	F	F	F	F	F	F	F	F	F	F	F	T	F	F	F	F	F
YDR026C	F	F	F	F	F	F	F	F	F	F	F	F	F	F	F	F	T	F	F	F	F	F	F
YDR027C	F	F	F	F	F	T	F	F	F	F	F	F	F	F	F	F	F	F	F	T	F	F	F
YDR028C	F	F	F	F	F	F	F	F	F	F	F	F	F	F	F	F	F	T	F	F	F	F	F
YDR032C	F	F	F	F	F	T	F	F	F	F	F	F	F	F	F	F	F	F	F	F	F	F	F
YDR036C	F	T	F	F	F	F	F	F	F	F	F	F	F	F	F	F	F	F	F	F	F	F	F
YDR037W	F	F	F	F	F	F	F	F	F	F	F	F	F	F	F	F	F	F	F	F	F	F	F
YDR041W	F	T	F	F	F	F	F	F	F	F	F	F	F	F	F	F	F	F	F	F	F	F	F
YDR043C	F	F	F	F	F	F	F	F	F	F	F	F	F	F	F	F	F	T	F	F	F	T	F
YDR044W	F	F	F	F	F	F	F	F	F	F	F	F	F	F	F	F	F	T	F	F	F	T	F
YDR045C	F	F	F	F	F	F	F	F	F	F	F	F	F	F	F	F	F	F	F	F	F	F	F
YDR049W	F	F	F	F	F	F	F	F	F	F	F	F	F	F	F	F	F	T	F	F	F	F	F
YDR050C	F	F	F	F	F	F	F	F	F	F	F	F	F	F	F	F	F	T	F	F	F	T	F
YDR052C	F	F	F	F	F	F	F	F	F	F	F	F	F	F	F	F	F	T	F	F	F	T	F
YDR054C	F	F	F	F	F	F	F	F	F	F	F	F	F	F	F	F	F	T	F	F	F	T	F
YDR057W	F	F	F	F	F	F	F	F	F	F	F	F	F	F	F	F	F	F	F	F	F	F	F
YDR059C	F	F	F	F	F	F	F	F	F	F	F	F	F	F	F	F	F	T	F	F	F	T	F
YDR060W	F	F	F	F	F	F	F	F	F	F	F	F	F	F	F	F	T	F	F	F	F	F	F
YDR061W	F	T	F	F	F	F	F	F	F	F	F	F	F	F	F	F	F	F	F	F	F	F	F
YDR062W	F	F	F	F	F	F	F	T	F	F	F	F	F	F	F	F	F	F	F	F	F	F	F
YDR064W	F	F	F	F	F	F	F	F	F	F	F	F	F	F	F	F	F	F	F	F	F	F	F
YDR069C	T	F	F	F	F	F	F	F	F	T	F	F	F	F	F	F	F	F	F	F	F	F	F
YDR071C	F	F	F	F	F	F	F	F	F	F	F	F	F	F	F	F	F	T	F	F	F	F	F
YDR073W	F	F	F	F	F	F	F	F	F	F	F	F	F	F	F	F	F	F	F	F	F	T	F
YDR074W	F	F	F	F	F	F	F	F	F	F	F	F	F	F	F	F	F	T	F	F	F	F	F
YDR075W	F	F	F	F	F	F	F	F	F	F	F	F	F	F	F	F	F	T	F	F	F	T	F
YDR076W	F	F	F	F	F	F	F	F	F	F	F	F	F	F	F	F	F	T	F	F	F	T	F
YDR077W	F	F	F	F	F	F	F	T	F	F	F	F	F	F	F	F	F	F	F	F	F	F	F
YDR079C-A	F	F	F	F	F	F	F	F	F	F	F	F	F	F	F	F	F	F	F	F	F	T	F
YDR080W	F	F	T	F	F	F	T	F	F	F	F	F	F	F	F	F	F	F	F	F	F	F	F
YDR082W	F	F	F	F	F	F	F	F	F	F	F	F	F	F	F	F	F	F	F	F	F	F	F
YDR083W	F	F	F	F	F	F	F	F	F	F	F	F	F	F	F	F	T	F	F	F	F	T	F
YDR084C	F	F	F	F	F	T	F	F	F	F	F	F	F	T	F	F	F	F	F	T	F	F	F
YDR085C	F	F	F	F	F	F	F	F	F	F	F	F	F	F	F	F	F	F	F	F	F	F	F
YDR086C	F	F	F	F	F	F	F	T	F	F	F	F	F	F	F	F	F	F	F	F	F	F	F
YDR087C	F	F	F	F	F	F	F	F	F	F	F	F	F	F	F	F	T	F	F	F	F	T	F
YDR091C	F	F	F	F	F	F	F	F	F	F	F	F	F	F	F	F	F	T	F	F	F	F	F
YDR092W	F	F	F	F	F	F	F	F	F	F	F	F	F	F	F	F	F	T	F	F	F	F	F
YDR096W	F	F	F	F	F	F	F	F	F	F	F	F	F	F	F	F	F	F	F	F	F	T	F
YDR097C	F	F	F	F	F	F	F	F	F	F	F	F	F	F	F	F	F	F	F	F	F	T	F
YDR098C	F	F	F	F	F	F	F	F	F	F	F	F	F	F	F	F	F	T	F	F	F	T	F
YDR099W	F	F	F	F	F	F	F	F	F	F	F	F	F	F	F	F	F	T	F	F	F	T	F
YDR100W	F	F	F	F	F	F	F	F	F	F	F	F	F	F	F	F	F	F	F	T	F	F	F
YDR101C	F	F	F	F	F	F	F	F	F	F	F	F	F	F	F	F	F	F	F	F	F	T	F
YDR103W	F	F	F	F	F	F	F	F	F	F	F	F	F	F	F	F	F	T	F	F	F	T	F
YDR106W	F	F	F	F	F	F	F	F	F	F	F	F	F	F	F	F	F	F	F	F	F	F	F
YDR108W	F	F	F	F	F	F	F	F	F	F	F	F	F	F	F	F	F	T	F	F	F	F	F

YDR110W	F	F	F	F	F	F	F	F	F	F	F	F	F	F	F	F	T	F	F	F	F	F	F
YDR113C	F	F	F	F	F	F	F	F	F	F	F	F	F	F	F	F	F	F	F	F	F	F	F
YDR115W	F	T	F	F	F	F	F	F	F	F	F	F	F	F	F	F	F	F	F	F	F	F	F
YDR116C	F	T	F	F	F	F	F	F	F	F	F	F	F	F	F	F	F	F	F	F	F	F	F
YDR118W	F	F	F	F	F	F	F	F	F	F	F	F	F	F	F	F	F	T	F	F	F	T	F
YDR121W	F	F	F	F	F	F	F	F	F	F	F	F	F	F	F	F	F	T	F	F	F	T	F
YDR122W	F	F	F	F	F	F	F	F	F	F	F	F	F	F	F	F	F	T	F	F	F	F	F
YDR123C	F	F	F	F	F	F	F	F	F	F	F	F	F	F	F	F	F	F	F	F	F	T	F
YDR127W	F	F	F	F	F	F	F	F	F	F	F	F	F	F	F	F	F	T	F	F	F	F	F
YDR129C	F	F	F	F	F	F	F	F	F	F	F	F	F	F	F	T	F	F	F	F	F	F	F
YDR130C	F	F	F	T	F	F	F	F	F	F	F	F	F	F	F	F	F	T	F	F	F	T	F
YDR131C	F	F	T	F	F	F	F	F	F	F	F	F	F	F	F	F	F	F	F	F	F	F	F
YDR135C	F	F	F	F	F	F	T	F	F	F	F	F	F	F	F	F	F	F	F	F	F	F	F
YDR137W	F	F	F	F	F	F	F	F	F	F	F	F	F	F	F	F	F	F	F	T	F	F	F
YDR138W	F	F	F	F	F	F	F	F	F	F	F	F	F	F	F	F	F	F	F	F	F	T	F
YDR139C	F	F	F	F	F	F	F	F	F	F	F	F	F	F	F	F	F	T	F	F	F	F	F
YDR140W	F	F	F	F	F	F	F	F	F	F	F	F	F	F	F	F	F	T	F	F	F	T	F
YDR141C	F	F	F	F	F	T	F	F	F	F	F	F	F	F	F	F	F	F	F	T	F	F	F
YDR142C	F	F	F	F	F	F	F	F	F	F	F	F	F	F	F	F	F	F	F	F	F	F	F
YDR143C	F	F	F	F	F	F	F	F	F	F	F	F	F	F	F	F	F	F	F	F	F	F	F
YDR145W	F	F	F	F	F	F	F	F	F	F	F	F	F	F	F	F	F	F	F	F	F	T	F
YDR146C	F	F	F	F	F	F	F	F	F	F	F	F	F	F	F	F	F	T	F	F	F	T	F
YDR147W	F	F	F	F	F	F	F	F	F	F	F	F	F	F	F	F	F	T	F	F	F	F	F
YDR148C	F	T	F	F	F	F	F	F	F	F	F	F	F	F	F	F	F	F	F	F	F	F	F
YDR150W	F	F	F	F	F	T	F	F	F	F	F	F	F	F	F	F	F	F	F	F	F	F	F
YDR152W	F	F	F	F	F	F	F	F	F	F	F	F	F	F	F	F	F	T	F	F	F	F	F
YDR153C	F	F	F	F	F	F	F	F	F	F	F	F	F	T	F	F	F	F	F	F	F	F	F
YDR155C	F	F	F	F	F	F	F	F	F	F	F	F	F	F	F	F	F	T	F	F	F	T	F
YDR156W	F	F	F	F	F	F	F	F	F	F	F	F	F	F	F	F	T	F	F	F	F	F	F
YDR158W	F	F	F	F	F	F	F	F	F	F	F	F	F	F	F	F	F	T	F	F	F	T	F
YDR159W	F	F	F	F	F	F	F	F	T	F	F	F	F	F	F	F	F	F	F	F	F	F	F
YDR162C	F	F	F	F	F	F	F	F	F	F	F	F	F	F	F	F	F	F	F	F	F	F	F
YDR164C	T	F	F	F	F	F	F	F	F	F	T	F	F	F	F	F	F	T	F	F	F	F	T
YDR166C	T	F	F	F	T	F	F	F	F	F	T	F	F	F	F	F	F	F	F	F	F	F	T
YDR167W	F	F	F	F	F	F	F	F	F	F	F	F	F	F	F	F	F	F	F	F	F	T	F
YDR168W	F	F	F	F	F	F	F	F	F	F	F	F	F	F	F	F	F	T	F	F	F	F	F
YDR169C	F	F	F	F	F	F	F	F	F	F	F	F	F	F	F	F	F	T	F	F	F	F	F
YDR170C	F	F	F	F	F	F	F	F	F	F	F	F	F	T	F	F	F	F	F	F	F	F	F
YDR171W	F	F	F	F	F	F	F	F	F	F	F	F	F	F	F	F	F	T	F	F	F	F	F
YDR172W	F	F	F	F	F	F	F	F	F	F	F	F	F	F	F	F	F	T	F	F	F	F	F
YDR173C	F	F	F	F	F	F	F	F	F	F	F	F	F	F	F	F	F	T	F	F	F	T	F
YDR174W	F	F	F	F	F	F	F	F	F	F	F	F	F	F	F	F	T	F	F	F	F	F	F
YDR175C	F	T	F	F	F	F	F	F	F	F	F	F	F	F	F	F	F	F	F	F	F	F	F
YDR176W	F	F	F	F	F	F	F	F	F	F	F	F	F	F	F	F	F	F	F	F	F	T	F
YDR179C	F	F	F	F	F	F	F	F	F	F	F	F	F	F	F	F	F	T	F	F	F	F	F
YDR180W	F	F	F	F	F	F	F	F	F	F	F	F	F	F	F	F	F	F	F	F	F	T	F
YDR181C	T	F	F	F	F	F	F	F	F	F	F	F	F	F	F	F	F	T	F	F	F	T	F
YDR186C	F	F	F	F	F	F	F	F	F	F	F	F	F	F	F	F	F	T	F	F	F	F	F
YDR188W	F	F	F	F	F	F	F	F	F	F	F	F	F	F	F	F	F	F	F	F	F	F	F
YDR189W	F	F	F	F	F	F	F	F	F	F	F	F	T	F	F	F	F	F	F	T	F	F	F
YDR190C	F	F	F	F	F	F	F	F	F	F	F	F	F	F	F	F	F	F	F	F	F	T	F
YDR192C	F	F	F	F	F	F	F	F	F	F	F	F	F	F	F	F	F	F	F	F	F	F	F
YDR194C	F	T	F	F	F	F	F	F	F	F	F	F	F	F	F	F	F	F	F	F	F	F	F
YDR195W	F	F	F	F	F	F	F	F	F	F	F	F	F	F	F	F	F	F	F	F	F	T	F
YDR200C	F	F	F	F	F	F	F	F	F	F	F	F	F	F	F	F	F	T	F	F	F	F	F
YDR201W	F	F	F	T	F	F	F	F	F	F	F	F	F	F	F	F	F	F	F	F	F	F	F
YDR202C	T	F	F	F	F	F	F	F	F	F	F	F	F	F	F	F	F	F	F	F	F	F	F
YDR206W	F	F	F	F	F	F	F	F	F	F	F	F	F	F	F	F	F	F	F	F	F	F	F
YDR207C	F	F	F	F	F	F	F	F	F	F	F	F	F	F	F	F	F	F	F	F	F	T	F
YDR211W	F	F	F	F	F	F	F	F	F	F	F	F	F	F	F	F	F	T	F	F	F	F	F
YDR212W	F	F	F	F	F	F	F	F	F	F	F	F	F	F	F	F	F	F	F	F	F	F	F
YDR214W	F	F	F	F	F	F	F	F	F	F	F	F	F	F	F	F	F	T	F	F	F	F	F
YDR216W	F	F	F	F	F	F	F	F	F	F	F	F	F	F	F	F	F	T	F	F	F	F	F
YDR217C	F	F	F	F	F	F	F	F	F	F	F	F	F	F	F	F	F	T	F	F	F	T	F
YDR218C	F	F	F	F	F	F	F	F	F	F	F	F	F	F	F	F	F	F	F	F	F	F	F
YDR219C	F	T	F	F	F	F	F	F	F	F	F	F	F	F	F	F	F	F	F	F	F	F	F
YDR221W	F	F	F	F	F	F	F	T	F	F	F	F	F	F	F	F	F	F	F	F	F	F	F
YDR223W	F	F	F	F	F	F	F	F	F	F	F	F	F	F	F	F	F	F	F	F	F	F	F

YDR224C	F	F	F	F	F	F	F	F	F	F	F	F	F	F	F	F	F	F	F	F	F	T	F
YDR225W	F	F	T	F	F	F	F	F	F	F	F	F	F	F	F	F	F	F	F	F	F	F	F
YDR226W	F	F	F	F	F	F	F	F	F	F	F	F	F	F	F	F	F	T	F	F	F	T	F
YDR227W	F	F	F	F	F	F	F	F	T	F	F	F	F	F	F	F	F	F	F	F	F	F	F
YDR228C	F	F	F	F	F	F	F	F	F	F	F	F	F	F	F	F	F	F	F	F	F	T	F
YDR229W	F	F	F	F	F	F	T	F	F	F	F	F	F	F	F	F	F	F	F	F	F	F	F
YDR233C	F	F	F	F	T	F	F	T	F	F	F	F	F	F	F	F	F	F	F	F	F	F	F
YDR235W	F	F	F	F	F	F	F	F	F	F	F	F	F	F	F	F	F	F	F	F	F	T	F
YDR237W	F	F	F	F	F	F	F	F	F	F	F	F	F	F	F	F	F	F	F	F	F	F	F
YDR238C	F	F	F	F	F	F	F	F	F	F	F	F	T	F	F	F	F	F	F	T	F	F	F
YDR240C	F	F	F	F	F	F	F	F	F	F	F	F	F	F	F	F	F	F	F	F	F	T	F
YDR244W	F	F	F	F	F	F	F	F	F	F	F	F	F	F	T	F	F	F	F	F	F	F	F
YDR245W	F	F	F	F	F	F	F	F	F	F	F	F	T	F	F	F	F	F	F	F	F	F	F
YDR246W	F	F	F	F	F	F	F	F	F	F	F	F	F	T	F	F	F	F	F	F	F	F	F
YDR247W	F	F	F	F	F	F	F	F	F	F	F	F	F	F	F	F	F	T	F	F	F	F	F
YDR252W	F	F	F	F	F	F	F	F	F	F	F	F	F	F	F	F	F	T	F	F	F	F	F
YDR253C	F	F	F	F	F	F	F	F	F	F	F	F	F	F	F	F	F	T	F	F	F	T	F
YDR254W	F	F	F	F	F	F	F	F	F	F	F	T	F	F	F	F	F	F	F	F	F	F	F
YDR255C	F	F	F	F	F	F	F	F	F	F	F	F	F	F	F	F	F	F	F	F	F	F	F
YDR258C	F	T	F	F	F	F	F	F	F	F	F	F	F	F	F	F	F	F	F	F	F	F	F
YDR259C	F	F	F	F	F	F	F	F	F	F	F	F	F	F	F	F	F	F	F	F	F	F	F
YDR260C	F	F	F	F	F	F	F	F	F	F	F	F	F	F	F	F	F	F	F	F	F	F	F
YDR264C	F	F	F	F	F	F	F	F	F	F	F	F	T	F	F	F	F	F	F	T	F	F	F
YDR265W	F	F	F	F	F	F	F	F	F	F	F	F	F	F	T	F	F	F	F	F	F	F	F
YDR267C	F	F	F	F	F	F	F	F	F	F	F	F	F	F	F	F	F	T	F	F	F	F	F
YDR277C	F	F	F	F	F	F	F	F	F	F	F	F	F	F	F	F	F	F	F	F	F	F	F
YDR280W	F	F	F	F	F	F	F	F	F	F	F	F	F	F	F	F	T	F	F	F	F	T	F
YDR283C	F	F	F	F	F	F	F	F	F	F	F	F	F	F	F	F	F	T	F	F	F	F	F
YDR285W	F	F	F	F	F	F	F	F	F	F	F	F	F	F	F	F	F	F	F	F	F	F	F
YDR288W	F	F	F	F	F	F	F	F	F	F	F	F	F	F	F	F	F	T	F	F	F	T	F
YDR289C	F	F	F	F	F	F	F	F	F	F	F	F	F	F	F	F	F	F	F	F	F	F	F
YDR293C	F	F	F	F	F	F	F	F	F	F	F	F	F	F	F	F	F	T	F	F	F	F	F
YDR295C	F	F	F	F	F	F	F	F	F	F	F	F	F	F	F	F	F	F	F	F	F	T	F
YDR296W	F	T	F	F	F	F	F	F	F	F	F	F	F	F	F	F	F	F	F	F	F	F	F
YDR297W	F	F	F	F	F	F	F	T	F	F	F	F	F	F	F	F	F	F	F	F	F	F	F
YDR298C	F	T	F	F	F	F	F	F	F	F	F	F	F	F	F	F	F	F	F	F	F	F	F
YDR299W	F	F	F	F	F	F	F	F	F	F	F	F	F	F	F	F	T	F	F	F	F	F	F
YDR300C	F	F	F	F	F	F	F	F	F	F	F	F	F	F	F	F	F	T	F	F	F	F	F
YDR301W	F	F	F	F	F	F	F	F	F	F	F	F	F	F	F	F	F	F	F	F	F	T	F
YDR303C	F	F	F	F	F	F	F	F	F	F	F	F	F	F	F	F	F	F	F	F	F	T	F
YDR306C	F	F	F	F	F	F	F	F	F	F	F	F	F	F	F	F	F	F	F	F	F	F	F
YDR308C	F	F	F	F	F	F	F	F	F	F	F	F	F	F	F	F	F	F	F	F	F	T	F
YDR309C	T	F	F	F	F	F	F	F	F	F	F	F	F	F	F	F	F	T	F	F	F	F	T
YDR310C	F	F	F	F	F	F	F	F	F	F	F	F	F	F	F	F	F	F	F	F	F	T	F
YDR311W	F	F	F	F	F	F	F	F	F	F	F	F	F	F	F	F	F	F	F	F	F	T	F
YDR313C	T	F	F	F	F	F	F	F	F	T	F	F	F	F	F	F	F	F	F	F	F	F	F
YDR314C	F	F	F	F	F	F	F	F	F	F	F	F	F	F	F	F	F	F	F	F	F	T	F
YDR318W	F	F	F	T	F	F	F	F	F	F	F	F	F	F	F	F	F	F	F	F	F	T	F
YDR320C	T	F	F	F	F	F	F	F	F	F	F	F	F	F	F	F	F	F	F	F	F	F	F
YDR320C-A	F	F	F	T	F	F	F	F	F	F	F	F	F	F	F	F	F	F	F	F	F	F	F
YDR322W	F	T	F	F	F	F	F	F	F	F	F	F	F	F	F	F	F	F	F	F	F	F	F
YDR323C	F	F	F	F	F	F	F	F	F	T	F	F	F	F	F	F	F	F	F	F	F	F	F
YDR324C	F	F	F	F	F	F	F	F	F	F	F	F	F	F	F	F	T	F	F	F	F	F	F
YDR325W	F	F	F	F	F	F	F	F	F	F	F	F	F	F	F	F	T	F	F	F	F	T	F
YDR328C	F	F	F	F	F	F	F	F	F	F	F	F	F	F	F	F	F	T	F	F	F	T	F
YDR329C	F	F	F	F	F	F	F	F	F	F	F	F	F	F	T	F	F	F	F	F	F	F	F
YDR330W	F	F	F	F	F	F	F	F	F	F	F	F	F	F	F	F	F	T	F	F	F	T	F
YDR334W	F	F	F	F	F	F	F	F	F	F	F	F	F	F	F	F	F	F	F	F	F	T	F
YDR335W	F	F	F	F	F	F	F	F	T	F	F	F	F	F	F	F	F	T	F	F	F	T	F
YDR337W	F	T	F	F	F	F	F	F	F	F	F	F	F	F	F	F	F	F	F	F	F	F	F
YDR338C	T	F	F	F	F	F	F	F	F	F	F	F	F	F	F	F	F	F	F	F	F	F	F
YDR341C	F	F	F	F	F	F	F	F	F	F	F	F	F	F	F	F	F	F	F	F	F	F	F
YDR345C	F	F	T	F	T	F	F	F	F	F	F	F	F	F	F	F	F	F	F	F	F	F	F
YDR346C	F	F	F	F	F	F	F	F	F	F	F	F	F	F	F	F	F	T	F	F	F	T	F
YDR347W	F	T	F	F	F	F	F	F	F	F	F	F	F	F	F	F	F	F	F	F	F	F	F
YDR348C	F	F	F	F	T	F	F	F	F	F	T	F	F	F	F	F	F	F	F	F	F	F	F
YDR356W	F	F	F	T	F	F	F	F	F	F	F	F	F	F	F	F	F	F	F	F	F	F	F
YDR357C	F	F	F	F	F	T	F	F	F	F	F	F	F	F	F	F	F	F	F	F	F	F	F

YDR358W	F	F	F	F	F	F	F	F	F	F	F	F	F	T	F	F	F	T	F	F	F	F	F
YDR359C	F	F	F	F	F	F	F	F	F	F	F	F	F	F	F	F	F	F	F	F	F	F	F
YDR361C	F	F	F	F	F	F	F	F	F	F	F	F	F	F	F	F	F	T	F	F	F	T	F
YDR362C	F	F	F	F	F	F	F	F	F	F	F	F	F	F	F	F	F	F	F	F	F	T	F
YDR363W	F	F	F	F	F	F	F	F	F	F	F	F	F	F	F	F	F	T	F	F	F	T	F
YDR363W-A	F	F	F	F	F	F	F	F	F	F	F	F	F	F	F	F	F	F	F	F	F	T	F
YDR364C	F	F	F	F	F	F	F	F	F	F	F	F	F	F	F	F	F	T	F	F	F	T	F
YDR365C	F	F	F	F	F	F	F	F	F	F	F	F	F	F	F	F	T	F	F	F	F	T	F
YDR369C	F	F	F	F	F	F	F	F	F	F	F	F	F	F	F	F	F	T	F	F	F	T	F
YDR372C	F	F	F	F	F	F	F	F	F	F	F	F	F	F	F	F	F	T	F	F	F	T	F
YDR373W	F	F	F	F	F	T	F	F	F	F	F	F	F	F	F	F	F	F	F	F	F	F	F
YDR377W	F	T	F	F	F	F	F	F	F	F	F	F	F	F	F	F	F	F	F	F	F	F	F
YDR378C	F	F	F	F	F	F	F	F	F	F	F	F	F	F	F	F	F	F	F	F	F	F	F
YDR379W	F	F	F	F	F	F	F	F	F	F	F	F	F	F	F	F	F	T	F	F	F	F	F
YDR381W	F	F	F	F	F	F	F	F	F	F	F	F	F	F	F	F	F	F	F	F	F	T	F
YDR382W	F	F	F	F	F	F	F	F	F	F	F	F	F	F	F	F	F	T	F	F	F	F	F
YDR383C	F	F	F	F	F	F	F	F	F	F	F	F	F	F	F	F	F	F	F	F	F	F	F
YDR385W	F	F	F	F	F	F	F	F	F	F	F	F	F	F	F	F	F	T	F	F	F	F	F
YDR386W	F	F	F	F	F	F	F	F	F	F	F	F	F	F	F	F	F	F	F	F	F	F	F
YDR388W	F	F	F	F	F	F	F	F	F	F	F	F	F	F	F	T	F	F	F	F	F	F	F
YDR389W	F	F	F	F	F	F	F	F	F	F	F	F	F	F	F	F	F	T	F	F	F	F	F
YDR390C	F	F	F	F	F	F	F	F	F	F	F	F	F	F	F	F	F	F	F	F	F	T	F
YDR392W	F	F	F	F	F	F	F	F	F	F	F	F	F	F	F	F	F	F	F	F	F	T	F
YDR394W	T	F	F	F	F	F	F	F	F	F	F	F	F	F	F	F	F	F	F	F	F	F	F
YDR395W	F	F	F	F	F	F	F	F	F	F	F	F	F	F	F	F	F	T	F	F	F	T	F
YDR397C	F	F	F	F	F	F	F	F	F	F	F	F	F	F	F	F	F	F	F	F	F	T	F
YDR398W	F	F	F	F	F	F	F	F	F	F	F	F	F	F	F	F	T	F	F	F	F	F	F
YDR404C	F	F	F	F	F	F	F	F	F	F	F	F	F	F	F	F	F	F	F	F	F	T	F
YDR405W	F	T	F	F	F	F	F	F	F	F	F	F	F	F	F	F	F	F	F	F	F	F	F
YDR407C	F	F	F	F	F	F	F	F	F	F	F	F	F	T	F	F	F	F	F	F	F	F	F
YDR409W	F	F	F	F	F	F	F	F	F	F	F	F	F	F	F	F	F	T	F	F	F	T	F
YDR411C	F	F	F	F	F	F	F	T	F	F	F	F	F	F	F	F	F	F	F	F	F	F	F
YDR412W	F	F	F	F	F	F	F	F	F	F	F	F	F	F	F	F	T	F	F	F	F	T	F
YDR415C	F	F	F	F	F	F	F	F	F	F	F	F	F	F	F	F	F	F	F	F	F	F	F
YDR416W	F	F	F	F	F	F	F	F	F	F	F	F	F	F	F	F	F	F	F	F	F	T	F
YDR418W	F	F	F	F	F	F	F	F	F	F	F	F	F	F	F	F	F	T	F	F	F	F	F
YDR419W	F	F	F	F	F	F	F	F	F	F	F	F	F	F	F	F	F	T	F	F	F	T	F
YDR421W	F	F	F	F	F	F	F	F	F	F	F	F	F	F	F	F	F	F	F	F	F	T	F
YDR422C	F	F	F	F	F	F	F	F	F	F	F	F	F	F	F	F	F	T	F	F	F	F	F
YDR423C	F	F	F	F	F	F	F	F	F	F	F	F	F	F	F	F	F	T	F	F	F	T	F
YDR424C	F	F	F	F	F	F	F	T	F	F	F	F	F	F	F	F	F	F	F	F	F	F	F
YDR425W	F	F	F	F	F	F	F	F	F	T	F	F	F	F	F	F	F	T	F	F	T	F	F
YDR427W	F	F	F	F	F	F	F	F	F	F	F	F	F	F	F	F	F	F	F	F	F	T	F
YDR429C	F	F	F	F	F	F	F	F	F	F	F	F	F	F	F	F	F	T	F	F	F	F	F
YDR430C	F	T	F	F	F	F	F	F	F	F	F	F	F	F	F	F	F	F	F	F	F	F	F
YDR432W	F	F	F	F	F	F	F	F	F	F	F	F	F	F	F	F	F	F	F	F	F	T	F
YDR436W	T	F	F	F	F	F	F	F	F	F	F	F	F	F	F	F	F	F	F	F	F	F	F
YDR439W	F	F	F	F	F	F	F	F	F	F	F	F	F	F	F	F	T	F	F	F	F	F	F
YDR440W	F	F	F	F	F	F	F	F	F	F	F	F	F	F	F	F	F	T	F	F	F	T	F
YDR443C	F	F	F	F	F	F	F	F	F	F	F	F	F	F	F	F	F	T	F	F	F	T	F
YDR446W	F	F	F	F	F	F	F	F	F	F	F	F	F	F	F	F	F	F	F	F	F	F	F
YDR447C	F	F	F	F	F	F	F	F	F	F	F	F	F	F	F	F	F	T	F	F	F	F	F
YDR448W	F	F	F	F	F	F	F	F	F	F	F	F	F	F	F	F	F	F	F	F	F	T	F
YDR449C	F	F	F	F	F	F	F	F	F	F	F	F	F	F	F	F	T	F	F	F	F	F	F
YDR450W	F	F	F	F	F	F	F	F	F	F	F	F	F	F	F	F	F	T	F	F	F	F	F
YDR453C	F	F	F	F	F	F	F	F	F	F	F	F	F	F	F	F	F	T	F	F	F	F	F
YDR456W	F	F	F	F	F	F	F	F	F	T	F	F	F	F	F	F	F	F	F	F	F	F	F
YDR457W	F	F	F	F	F	F	F	F	F	F	F	F	F	F	F	F	T	F	F	F	F	T	F
YDR460W	F	F	F	F	F	F	F	F	F	F	F	F	F	F	F	F	F	F	F	F	F	T	F
YDR462W	F	T	F	F	F	F	F	F	F	F	F	F	F	F	F	F	F	F	F	F	F	F	F
YDR464W	F	F	F	F	F	F	F	F	F	F	F	F	F	F	F	F	F	F	F	F	F	T	F
YDR465C	F	F	F	F	F	F	F	F	F	F	F	F	F	F	F	F	F	T	F	F	F	T	F
YDR468C	F	F	F	F	F	F	F	F	F	F	F	F	F	F	F	F	F	F	F	F	F	F	F
YDR469W	F	F	F	F	F	F	F	F	F	F	F	F	F	F	F	F	F	F	F	F	F	T	F
YDR470C	F	F	F	F	F	F	F	F	F	F	F	F	F	F	F	F	F	F	F	F	F	F	F
YDR471W	F	F	F	F	F	F	F	F	F	F	F	F	F	F	F	F	F	T	F	F	F	F	F
YDR472W	F	F	F	F	F	F	F	F	F	F	F	F	F	T	F	F	F	F	F	F	F	F	F
YDR473C	F	F	F	F	F	F	F	F	F	F	F	F	F	F	F	F	F	F	F	F	F	T	F

YDR475C	F	F	F	F	F	F	F	F	F	F	F	F	F	F	F	F	F	F	F	F	F	F	F
YDR477W	F	F	F	F	F	F	F	F	F	F	F	F	F	F	F	F	F	T	F	F	F	F	F
YDR478W	F	F	F	F	F	F	F	F	F	F	F	F	F	F	F	F	F	F	F	F	F	T	F
YDR479C	T	F	F	F	F	T	F	F	F	F	F	F	F	F	F	F	F	T	F	F	F	T	F
YDR480W	F	F	F	F	F	F	F	F	F	F	F	F	F	F	F	F	F	T	F	F	F	T	F
YDR482C	F	F	F	F	F	F	F	F	F	F	F	F	F	F	F	F	F	T	F	F	F	T	F
YDR483W	F	F	T	F	F	F	F	F	F	F	F	F	F	F	F	F	F	F	F	F	F	F	F
YDR484W	F	F	F	F	F	T	F	F	F	F	F	F	F	F	F	F	F	F	F	F	F	F	F
YDR485C	F	F	F	F	F	F	F	F	F	F	F	F	F	F	F	F	F	F	F	F	F	T	F
YDR486C	F	F	F	F	F	F	T	F	F	F	F	F	F	F	F	F	F	F	F	F	F	F	F
YDR488C	F	F	F	F	F	F	F	F	F	F	F	F	F	F	F	F	F	F	F	F	F	F	F
YDR489W	F	F	F	F	F	F	F	F	F	F	F	F	F	F	F	F	F	F	F	F	F	F	F
YDR490C	F	F	F	F	F	F	F	F	F	F	F	F	F	F	F	F	F	F	F	F	F	F	F
YDR495C	F	F	F	F	F	T	F	F	F	F	F	F	F	F	F	F	F	F	F	F	F	F	F
YDR496C	F	F	F	F	F	F	F	F	F	F	F	F	F	F	F	F	T	F	F	F	F	T	F
YDR497C	F	F	T	F	T	F	F	F	F	F	F	F	F	F	F	F	F	F	F	F	F	F	F
YDR498C	F	F	F	F	F	F	F	T	F	F	F	F	F	F	F	F	F	F	F	F	F	F	F
YDR499W	F	F	F	F	F	F	F	F	F	F	F	F	F	F	F	F	F	T	F	F	F	T	F
YDR502C	T	F	F	F	F	F	F	F	F	F	F	F	F	F	F	F	F	F	F	F	F	F	F
YDR505C	F	F	F	F	F	F	F	F	F	F	F	F	F	F	F	F	F	T	F	F	F	F	F
YDR507C	T	F	F	F	F	F	F	F	F	F	T	F	F	F	F	F	F	T	F	F	F	F	T
YDR508C	F	F	F	F	T	F	F	T	F	F	F	F	F	F	F	F	F	F	F	F	F	F	F
YDR510W	F	F	F	F	F	F	F	F	F	F	F	F	F	F	F	F	F	T	F	F	F	T	F
YDR517W	F	F	F	F	F	T	F	F	F	F	F	F	F	F	F	F	F	F	F	F	F	F	F
YDR527W	F	F	F	F	F	F	F	F	F	F	F	F	F	F	F	F	F	T	F	F	F	F	F
YDR532C	T	F	F	T	F	F	F	F	F	F	F	F	F	F	F	F	F	F	F	F	F	F	F
YDR533C	F	F	F	F	F	F	F	F	F	F	F	F	F	F	F	F	F	T	F	F	F	T	F
YEL002C	F	F	F	F	F	F	F	T	F	F	F	F	F	F	F	F	F	F	F	F	F	F	F
YEL003W	F	F	F	F	F	F	F	F	F	F	F	F	F	F	F	F	F	T	F	F	F	F	F
YEL005C	F	F	F	F	F	T	F	F	F	F	F	F	F	F	F	F	F	F	F	F	F	F	F
YEL009C	F	F	F	F	F	F	F	F	F	F	F	F	F	F	F	F	F	T	F	F	F	T	F
YEL013W	F	F	F	F	F	F	T	F	F	F	F	F	F	F	F	F	F	F	F	F	F	F	F
YEL015W	F	F	F	F	F	T	F	F	F	F	F	F	F	F	F	F	F	F	F	F	F	F	F
YEL018W	F	F	F	F	F	F	F	F	F	F	F	F	F	F	F	F	F	F	F	F	F	T	F
YEL019C	F	F	F	F	F	F	F	F	F	F	F	F	F	F	F	F	F	T	F	F	F	T	F
YEL020W-A	F	F	F	F	F	F	F	F	F	F	F	F	F	F	F	F	F	F	F	F	F	F	F
YEL022W	F	F	F	F	F	F	F	F	F	F	F	F	F	F	F	F	F	T	F	T	F	F	F
YEL024W	F	T	F	F	F	F	F	F	F	F	F	F	F	F	F	F	F	F	F	F	F	F	F
YEL025C	F	F	F	F	F	F	F	F	F	F	F	F	F	F	F	F	F	T	F	F	F	T	F
YEL026W	F	F	F	F	F	F	F	F	F	F	F	F	F	F	F	F	F	F	F	F	F	F	F
YEL027W	F	F	F	F	F	F	F	T	F	F	F	F	F	F	F	F	F	F	F	F	F	F	F
YEL030W	F	F	F	F	F	F	F	F	F	F	F	F	F	F	F	F	F	F	F	F	F	F	F
YEL032W	F	F	F	F	F	F	F	F	F	F	F	F	F	F	F	F	F	F	F	F	F	F	F
YEL034W	F	F	F	F	F	F	F	F	F	F	F	F	F	F	F	F	F	F	F	F	F	F	F
YEL036C	F	F	F	F	F	F	F	F	F	F	F	F	T	F	F	F	F	F	F	F	F	F	F
YEL037C	F	F	F	F	F	F	F	F	F	F	F	F	F	F	F	F	F	T	F	F	F	T	F
YEL043W	F	F	F	F	F	F	F	T	F	F	F	F	F	F	F	F	F	F	F	F	F	F	F
YEL044W	F	F	F	F	F	F	F	F	F	F	F	F	F	F	F	F	F	F	F	F	F	T	F
YEL046C	F	F	F	F	F	F	F	F	F	F	F	F	F	F	F	F	F	T	F	F	F	T	F
YEL047C	F	F	F	F	F	F	F	F	F	F	F	F	F	F	F	F	F	T	F	F	F	T	F
YEL048C	F	F	F	F	F	F	F	F	F	F	F	F	F	T	F	F	F	F	F	F	F	F	F
YEL051W	F	F	F	F	F	F	T	F	F	F	F	F	F	F	F	F	F	F	F	F	F	F	F
YEL054C	F	F	F	F	F	F	F	F	F	F	F	F	F	F	F	F	F	T	F	F	F	F	F
YEL055C	F	F	F	F	F	F	F	F	F	F	F	F	F	F	F	F	T	F	F	F	F	T	F
YEL056W	F	F	F	F	F	F	F	F	F	F	F	F	F	F	F	F	F	F	F	F	F	F	F
YEL058W	F	F	F	F	F	F	F	F	F	F	F	F	F	F	F	F	F	T	F	F	F	T	F
YEL062W	T	F	F	F	F	F	F	F	F	F	F	F	F	F	F	F	F	F	F	F	F	F	F
YEL065W	F	F	T	F	F	F	F	F	F	F	F	F	F	F	F	F	F	F	F	F	F	F	F
YEL071W	F	F	F	F	F	F	F	F	F	F	F	F	F	F	F	F	F	T	F	F	F	T	F
YER002W	F	F	F	F	F	F	F	F	F	F	F	F	F	F	F	F	T	F	F	F	F	T	F
YER005W	T	F	F	F	F	F	F	F	F	F	F	F	T	F	F	F	F	F	F	T	F	F	F
YER006W	F	F	F	F	F	F	F	F	F	F	F	F	F	F	F	F	T	F	F	F	F	T	F
YER007C-A	F	F	F	F	F	F	F	F	F	F	F	F	F	F	F	F	F	T	F	F	F	F	F
YER007W	F	F	F	F	F	F	F	F	F	F	F	F	F	F	F	F	F	F	F	F	F	F	F
YER008C	F	F	F	F	T	F	F	F	F	F	T	F	F	F	F	F	F	F	F	F	F	F	T
YER012W	F	F	F	F	F	F	F	F	F	F	F	F	F	F	F	F	F	F	F	F	F	F	F
YER013W	F	F	F	F	F	F	F	F	F	F	F	F	F	F	F	F	F	T	F	F	F	T	F
YER016W	F	F	F	F	F	F	F	F	F	F	F	T	F	F	F	F	F	F	F	F	F	F	F

YER017C	F	T	F	F	F	F	F	F	F	F	F	F	F	F	F	F	F	F	F	F	F	F	F
YER018C	F	F	F	T	F	F	F	F	F	F	F	F	F	F	F	F	F	F	F	F	F	F	F
YER019C-A	F	F	F	F	F	F	F	T	F	F	F	F	F	F	F	F	F	F	F	F	F	F	F
YER020W	F	F	F	F	T	F	F	F	F	F	F	F	F	F	F	F	F	F	F	F	F	F	F
YER021W	F	F	F	F	F	F	F	F	F	F	F	F	F	F	F	F	F	T	F	F	F	T	F
YER022W	F	F	F	F	F	F	F	F	F	F	F	F	F	F	F	F	F	F	F	F	F	T	F
YER023W	F	F	F	F	F	F	F	F	F	F	F	F	F	F	F	F	F	T	F	F	F	F	F
YER025W	F	F	F	F	F	F	F	F	F	F	F	F	F	F	F	F	F	T	F	F	F	F	F
YER027C	F	F	F	F	F	F	F	F	F	F	F	F	F	F	F	F	F	T	F	F	F	F	F
YER029C	F	F	F	F	F	F	F	F	F	F	F	F	F	F	F	F	F	F	F	F	F	T	F
YER030W	F	F	F	F	F	F	F	F	F	F	F	F	F	F	F	F	F	F	F	F	F	T	F
YER031C	F	F	F	F	F	F	F	F	F	F	F	F	F	F	F	F	F	T	F	F	F	T	F
YER032W	F	F	F	F	F	F	F	F	F	F	T	F	F	F	F	F	F	F	F	F	F	F	F
YER033C	F	F	F	F	F	F	F	F	F	F	F	F	F	F	F	F	F	T	F	F	F	F	F
YER036C	F	F	F	F	F	F	F	F	F	F	F	F	F	F	F	F	F	T	F	F	F	F	F
YER038C	F	F	F	F	F	F	F	F	F	F	F	F	F	F	F	F	F	T	F	F	F	T	F
YER040W	F	F	F	F	F	F	F	F	F	F	F	F	F	F	F	F	F	T	F	F	F	T	F
YER041W	F	F	F	F	F	F	F	F	F	F	F	F	F	F	F	F	F	F	F	F	F	F	F
YER042W	F	F	F	F	F	F	F	F	F	F	F	F	F	F	F	F	F	T	F	F	F	T	F
YER043C	F	F	F	F	F	F	F	F	F	F	F	F	F	F	F	F	F	T	F	F	F	F	F
YER044C	F	F	F	F	F	F	F	T	F	F	F	F	F	F	F	F	F	F	F	F	F	F	F
YER047C	F	F	F	F	F	F	F	F	F	F	F	F	F	F	F	F	F	T	F	F	F	F	F
YER048C	F	F	F	F	F	F	F	F	F	F	F	F	F	F	F	F	F	F	F	F	F	F	F
YER048W-A	F	T	F	F	F	F	F	F	F	F	F	F	F	F	F	F	F	F	F	F	F	F	F
YER049W	F	F	F	F	F	F	F	F	F	F	F	F	F	F	F	F	F	F	F	F	F	T	F
YER050C	F	F	F	F	F	F	F	F	F	F	F	F	F	F	F	F	F	F	F	F	F	F	F
YER051W	F	F	F	F	F	F	F	F	F	F	F	F	F	F	F	F	F	F	F	F	F	F	F
YER052C	F	F	F	F	F	F	F	F	F	F	F	F	F	F	F	F	F	T	F	F	F	F	F
YER054C	F	F	F	F	F	T	F	F	F	F	F	F	F	F	F	F	F	T	F	F	F	F	F
YER056C	F	F	T	F	F	F	F	F	F	F	F	F	F	F	F	F	F	F	F	F	F	F	F
YER059W	F	F	F	F	F	F	F	F	F	F	F	F	F	F	F	F	F	T	F	F	F	T	F
YER062C	F	F	F	F	F	F	F	F	F	F	F	F	F	F	F	F	F	T	F	F	F	T	F
YER063W	F	F	F	F	F	F	F	F	F	F	F	F	F	F	F	F	F	F	F	F	F	T	F
YER067W	F	F	F	F	F	F	F	F	F	F	F	F	F	F	F	F	F	T	F	F	F	T	F
YER068W	F	F	F	F	F	F	F	F	F	F	F	F	F	F	F	F	F	T	F	F	F	F	F
YER069W	F	T	F	F	F	F	F	F	F	F	F	F	F	F	F	F	F	F	F	F	F	F	F
YER070W	T	F	F	F	F	F	F	F	F	F	F	F	F	F	F	F	F	F	F	F	F	F	F
YER071C	F	F	F	F	F	T	F	F	F	F	F	F	F	F	F	F	F	F	F	F	F	F	F
YER073W	F	T	F	F	F	F	F	F	F	F	F	F	F	F	F	F	F	F	F	F	F	F	F
YER075C	F	F	F	F	F	F	F	F	F	F	F	F	F	F	F	F	F	T	F	F	F	F	F
YER081W	F	F	F	F	F	F	F	F	F	F	F	F	F	F	F	F	F	T	F	F	F	F	F
YER082C	F	F	F	F	F	F	F	F	F	F	F	F	F	F	F	F	F	F	F	F	F	F	F
YER083C	F	F	F	F	F	F	F	T	F	F	F	F	F	F	F	F	F	F	F	F	F	F	F
YER086W	F	T	F	F	F	F	F	F	F	F	F	F	F	F	F	F	F	F	F	F	F	F	F
YER087C-B	T	F	F	F	F	F	F	F	F	F	F	F	F	F	F	F	F	F	F	F	F	F	F
YER088C	F	F	F	F	F	F	F	F	F	F	F	F	F	F	F	F	F	T	F	F	F	T	F
YER089C	F	F	F	F	F	F	F	F	F	F	F	F	F	F	F	F	F	T	F	F	F	T	F
YER090W	F	F	F	F	F	F	F	F	F	F	F	F	F	F	F	F	F	T	F	F	F	F	F
YER091C	F	F	F	F	F	F	F	F	F	F	F	F	F	F	F	F	F	T	F	F	F	F	F
YER092W	F	F	F	F	F	F	F	F	F	F	F	F	F	F	F	F	F	F	F	F	F	T	F
YER093C	F	F	F	F	F	F	F	F	F	F	F	F	F	F	F	F	F	F	F	F	F	F	F
YER094C	F	F	F	F	F	F	F	F	F	F	F	F	F	F	F	F	F	F	F	F	F	F	F
YER095W	F	F	F	F	F	F	F	F	F	F	F	F	F	F	F	F	F	T	F	F	F	T	F
YER099C	F	F	F	F	F	F	F	F	F	F	F	F	F	F	F	F	F	T	F	F	F	F	F
YER100W	F	F	F	F	F	F	F	T	F	F	F	F	F	F	F	F	F	F	F	F	F	F	F
YER102W	F	F	F	F	F	F	F	F	F	F	F	F	F	F	F	F	F	T	F	F	F	F	F
YER103W	F	F	F	F	F	F	F	F	F	F	F	F	F	F	F	F	F	T	F	F	F	F	F
YER105C	F	F	F	F	F	F	F	F	T	F	F	F	F	F	F	F	F	F	F	F	F	F	F
YER106W	F	F	F	F	F	F	F	F	F	F	F	F	F	F	F	F	F	F	F	F	F	F	F
YER107C	F	F	F	F	F	F	F	F	T	F	F	F	F	F	F	F	F	T	F	F	F	F	F
YER110C	F	F	F	F	F	F	F	F	F	F	F	F	F	F	F	F	F	T	F	F	F	T	F
YER111C	F	F	F	F	F	F	F	F	F	F	F	F	F	F	F	F	F	T	F	F	F	T	F
YER112W	F	F	F	F	F	F	F	F	F	F	F	F	F	F	F	F	F	T	F	F	F	T	F
YER114C	F	F	F	F	T	T	F	F	F	F	T	F	F	F	F	F	F	T	F	F	F	F	T
YER116C	F	F	F	F	F	F	F	F	F	F	F	F	F	F	F	F	F	T	F	F	F	T	F
YER117W	F	F	F	F	F	F	F	F	F	F	F	F	F	F	F	F	F	F	F	F	F	T	F
YER118C	F	F	F	F	T	F	F	F	F	F	T	F	F	F	F	F	F	T	F	F	F	F	F
YER120W	F	F	F	F	F	F	F	T	F	F	F	F	F	F	F	F	F	F	F	F	F	F	F

YER122C	F	F	F	F	F	F	F	F	F	F	F	F	T	F	F	F	F	F	F	T	F	F	F
YER124C	F	F	F	F	F	F	F	F	F	F	T	F	F	F	F	F	F	F	F	F	F	F	F
YER125W	F	F	F	F	F	F	F	F	F	F	F	F	F	F	F	F	F	F	F	F	F	F	F
YER126C	F	F	F	F	F	F	F	F	F	F	F	F	F	F	F	F	F	T	F	F	F	F	F
YER127W	F	F	F	F	F	F	F	F	F	F	F	F	F	F	F	F	F	F	F	F	F	F	F
YER128W	T	F	F	F	F	F	F	F	F	F	F	F	F	F	F	F	F	F	F	F	F	F	F
YER129W	F	F	F	F	F	F	F	F	F	F	F	F	F	F	F	F	F	T	F	F	F	F	F
YER131W	F	F	F	F	F	F	F	F	F	F	F	F	F	F	F	F	F	T	F	F	F	F	F
YER132C	F	F	F	F	F	F	F	F	F	F	F	F	F	F	F	F	F	T	F	F	F	F	F
YER133W	F	F	F	F	F	T	F	F	F	F	F	F	F	F	F	F	F	T	F	F	F	T	F
YER136W	F	F	F	F	F	F	F	F	F	F	F	F	F	F	F	F	F	T	F	F	F	F	F
YER139C	F	F	F	F	F	F	F	F	F	F	F	F	F	F	F	F	F	T	F	F	F	F	F
YER143W	F	F	F	F	F	F	F	F	F	F	F	F	F	F	F	F	F	T	F	F	F	F	F
YER144C	F	F	F	F	F	F	F	F	F	F	F	F	F	F	F	F	F	F	F	F	F	F	F
YER146W	F	F	F	F	F	F	F	F	F	F	F	F	F	F	F	F	F	F	F	F	F	F	F
YER147C	F	F	F	F	F	F	F	F	F	F	F	F	F	F	F	F	F	T	F	F	F	T	F
YER148W	F	F	F	F	F	F	F	F	F	F	F	F	F	F	F	F	F	F	F	F	F	T	F
YER149C	T	F	F	F	T	F	F	F	F	F	F	F	F	F	F	F	F	F	F	F	F	F	T
YER151C	F	F	F	F	F	F	F	F	F	F	F	F	F	F	F	F	F	T	F	F	F	F	F
YER154W	F	T	F	F	F	F	F	F	F	F	F	F	F	F	F	F	F	F	F	F	F	F	F
YER155C	F	F	F	F	F	F	F	F	F	F	T	F	F	F	F	F	F	T	F	F	F	F	F
YER157W	F	F	F	F	F	F	F	F	F	F	F	F	T	F	F	F	F	F	F	T	F	F	F
YER158C	F	F	F	F	F	F	F	F	F	F	F	F	F	F	F	F	F	F	F	F	F	F	F
YER159C	F	F	F	F	F	F	F	F	F	F	F	F	F	F	F	F	F	F	F	F	F	T	F
YER161C	F	F	F	F	F	F	F	F	F	F	F	F	F	F	F	F	F	F	F	F	F	T	F
YER162C	F	F	F	F	F	F	F	F	F	F	F	F	F	F	F	F	F	T	F	F	F	T	F
YER164W	F	F	F	F	F	F	F	F	F	F	F	F	F	F	F	F	F	F	F	F	F	T	F
YER165W	F	F	F	F	F	F	F	F	F	F	F	F	F	F	F	F	F	T	F	F	F	F	F
YER167W	F	F	F	F	F	F	F	F	F	F	F	F	F	F	F	F	F	T	F	F	F	T	F
YER168C	F	F	F	F	F	F	F	F	F	F	F	F	F	F	F	F	F	T	F	F	F	F	F
YER169W	F	F	F	F	F	F	F	F	F	F	F	F	F	F	F	F	F	F	F	F	F	T	F
YER171W	F	F	F	F	F	F	F	F	F	F	F	F	F	F	F	F	F	T	F	F	F	T	F
YER172C	F	F	F	F	F	F	F	F	F	F	F	F	F	F	F	F	F	T	F	F	F	T	F
YER173W	F	F	F	F	F	F	F	F	F	F	F	F	F	F	F	F	F	T	F	F	F	T	F
YER174C	F	F	F	F	F	F	F	F	F	F	F	F	F	F	F	F	F	T	F	F	F	T	F
YER177W	F	F	F	F	F	F	F	F	F	F	F	F	F	F	F	F	F	T	F	F	F	F	F
YER178W	F	T	F	F	F	F	F	F	F	F	F	F	F	F	F	F	F	F	F	F	F	F	F
YER179W	F	F	F	F	F	F	F	F	F	F	F	F	F	F	F	F	F	F	F	F	F	F	F
YFL002C	F	F	F	F	F	F	F	F	F	F	F	F	F	F	F	F	T	F	F	F	F	T	F
YFL004W	F	F	F	F	F	F	F	T	F	F	F	F	F	F	F	F	F	F	F	F	F	F	F
YFL005W	F	F	F	F	F	F	F	F	F	F	F	F	F	F	F	F	F	F	F	F	F	F	F
YFL007W	F	F	F	F	F	F	F	F	F	F	F	F	F	F	F	F	F	T	F	F	F	T	F
YFL008W	F	F	F	F	F	F	F	F	F	F	F	F	F	F	F	F	F	T	F	F	F	T	F
YFL009W	F	F	F	F	F	F	F	F	F	F	F	F	F	F	F	F	F	F	F	F	F	F	F
YFL011W	F	F	F	F	F	F	F	F	F	F	F	F	F	F	F	F	F	F	F	F	F	F	F
YFL013C	F	F	F	F	F	F	F	F	F	F	F	F	F	F	F	F	F	F	F	F	F	T	F
YFL016C	F	T	F	F	F	F	F	F	F	F	F	F	F	F	F	F	F	F	F	F	F	F	F
YFL017W-A	F	F	F	F	F	F	F	F	F	F	F	F	F	F	F	F	F	F	F	F	F	T	F
YFL018C	F	T	F	F	F	F	F	F	F	F	F	F	F	F	F	F	F	F	F	F	F	F	F
YFL022C	F	F	F	F	F	F	F	F	F	F	F	F	F	F	F	F	F	F	F	F	F	F	F
YFL023W	F	F	F	F	F	F	F	F	F	F	F	F	F	F	F	F	F	T	F	F	F	F	F
YFL024C	F	F	F	F	F	F	F	F	F	F	F	F	F	F	F	F	F	F	F	F	F	T	F
YFL025C	F	F	F	F	F	F	F	T	F	F	F	F	F	F	F	F	F	F	F	F	F	F	F
YFL026W	F	F	T	F	F	F	F	F	F	F	F	F	F	F	F	F	F	F	F	F	F	F	F
YFL028C	F	F	F	F	F	F	F	F	F	F	F	F	F	F	F	F	F	T	F	F	F	T	F
YFL029C	F	F	F	F	F	F	F	F	F	F	F	F	F	F	F	F	F	T	F	F	F	F	F
YFL030W	F	F	F	F	F	F	F	F	F	F	F	F	F	F	F	F	F	F	F	F	F	F	F
YFL031W	F	F	F	F	F	F	F	F	F	F	F	F	F	F	F	F	F	T	F	F	F	T	F
YFL033C	F	F	F	F	F	F	F	F	F	F	F	F	F	F	F	F	F	T	F	F	F	F	F
YFL034C-B	F	F	F	F	T	F	F	F	F	F	F	F	F	F	F	F	F	T	F	F	F	F	F
YFL034W	T	F	F	F	F	F	F	F	F	F	F	F	F	F	F	F	F	F	F	F	F	F	F
YFL036W	F	F	F	F	F	T	F	F	F	F	F	F	F	F	F	F	F	F	F	F	F	F	F
YFL037W	F	F	F	F	F	F	F	F	F	F	F	F	F	F	F	F	F	F	F	F	F	F	F
YFL038C	F	F	F	F	F	F	F	F	F	F	F	F	F	F	F	F	F	F	F	F	F	F	F
YFL039C	F	F	F	F	F	F	F	F	F	F	F	F	F	F	F	F	F	F	F	F	F	F	F
YFL044C	F	F	F	F	F	F	F	F	F	F	F	F	F	F	F	F	F	T	F	F	F	T	F
YFL045C	F	F	F	F	F	F	F	F	F	F	F	F	F	F	F	F	F	T	F	F	F	T	F
YFL048C	F	F	F	F	F	F	T	F	F	F	F	F	F	F	F	F	F	F	F	F	F	F	F

YFL049W	F	F	F	F	F	F	F	F	F	F	F	F	F	F	F	F	F	F	F	F	F	T	F
YFL050C	F	F	F	F	F	F	F	F	F	F	F	F	F	F	F	F	F	F	F	F	F	F	F
YFL052W	F	F	F	F	F	F	F	F	F	F	F	F	F	F	F	F	F	F	F	F	F	F	F
YFL059W	F	F	F	F	F	F	F	F	F	F	F	F	F	F	F	F	F	F	F	F	F	F	F
YFL060C	F	F	F	F	F	F	F	F	F	F	F	F	F	F	F	F	F	F	F	F	F	F	F
YFR001W	F	F	F	F	F	F	F	F	F	F	F	F	F	F	F	F	T	F	F	F	F	T	F
YFR002W	F	F	F	F	F	F	F	F	T	F	F	F	F	F	F	F	F	F	F	F	F	F	F
YFR003C	F	F	F	F	F	F	F	F	F	F	F	F	F	F	F	F	F	F	F	F	F	F	F
YFR004W	F	F	F	F	F	F	F	F	F	F	F	F	F	F	F	F	F	T	F	F	F	T	F
YFR006W	F	F	F	F	F	F	F	F	F	F	F	F	F	F	F	F	F	T	F	F	F	F	F
YFR008W	T	F	F	F	F	F	F	F	F	F	F	F	F	F	F	F	F	F	F	F	F	F	F
YFR009W	F	F	F	F	F	F	F	F	F	F	F	F	F	F	F	F	F	T	F	F	F	F	F
YFR010W	F	F	F	F	F	F	F	F	F	F	F	F	F	F	F	F	F	T	F	F	F	T	F
YFR011C	F	T	F	F	F	F	F	F	F	F	F	F	F	F	F	F	F	T	F	F	F	F	F
YFR013W	F	F	F	F	F	F	F	F	F	F	F	F	F	F	F	F	F	F	F	F	F	T	F
YFR014C	F	F	F	F	F	F	F	F	F	F	F	F	F	F	F	F	F	T	F	F	F	F	F
YFR015C	F	F	F	F	F	T	F	F	F	F	F	F	F	F	F	F	F	T	F	F	F	F	F
YFR016C	T	F	F	F	F	F	F	F	F	F	F	F	F	F	F	F	F	T	F	F	F	F	T
YFR017C	F	F	F	F	F	F	F	F	F	F	F	F	F	F	F	F	F	T	F	F	F	F	F
YFR019W	F	F	F	F	F	F	T	F	F	F	F	F	F	F	F	F	F	F	F	F	F	F	F
YFR021W	T	F	F	F	F	F	F	F	F	F	F	F	F	F	F	F	F	F	F	F	F	F	F
YFR022W	F	F	F	F	F	F	F	F	F	F	F	F	F	F	F	F	F	F	F	F	F	F	F
YFR024C-A	F	T	F	F	T	F	F	F	F	F	T	F	F	F	F	F	F	T	F	F	F	F	F
YFR027W	F	F	F	F	F	F	F	F	F	F	F	F	F	F	F	F	F	T	F	F	F	T	F
YFR028C	F	F	F	F	F	F	F	F	F	F	F	F	F	F	F	F	T	F	F	F	F	F	F
YFR030W	F	F	F	F	F	F	F	F	F	F	F	F	F	F	F	F	F	T	F	F	F	F	F
YFR031C	F	F	F	F	F	F	F	F	F	F	F	F	F	F	F	F	F	T	F	F	F	T	F
YFR031C-A	F	F	F	F	F	F	F	F	F	F	F	F	F	F	F	F	F	T	F	F	F	F	F
YFR034C	F	F	F	F	F	F	F	F	F	F	F	F	F	F	F	F	F	T	F	F	F	T	F
YFR036W	F	F	F	F	F	F	F	F	F	F	F	F	F	F	F	F	F	F	F	F	F	T	F
YFR037C	F	F	F	F	F	F	F	F	F	F	F	F	F	F	F	F	F	F	F	F	F	T	F
YFR040W	F	F	F	F	F	F	F	F	F	F	F	F	F	F	F	F	F	T	F	F	F	F	F
YFR042W	F	F	F	F	F	F	F	F	F	F	F	F	F	F	F	F	F	F	F	F	F	F	F
YFR044C	F	T	F	F	F	F	F	F	F	F	F	F	F	F	F	F	F	T	F	F	F	F	F
YFR046C	F	F	F	F	F	F	F	F	F	F	F	F	F	F	F	F	F	F	F	F	F	T	F
YFR047C	F	F	F	F	F	F	F	F	F	F	F	F	F	F	F	F	F	T	F	F	F	T	F
YFR049W	F	T	F	F	F	F	F	F	F	F	F	F	F	F	F	F	F	F	F	F	F	F	F
YFR050C	F	F	F	F	F	F	F	F	F	F	F	F	F	F	F	F	F	T	F	F	F	T	F
YFR051C	F	F	F	F	F	F	F	F	F	F	F	F	T	F	F	F	F	F	F	T	F	F	F
YFR052W	F	F	F	F	F	F	F	F	F	F	F	F	F	F	F	F	F	F	F	F	F	T	F
YGL001C	F	F	F	F	F	F	F	T	F	F	F	F	F	F	F	F	F	F	F	F	F	F	F
YGL003C	F	F	F	F	F	F	F	F	F	F	F	F	F	F	F	F	F	F	F	F	F	F	F
YGL004C	F	F	F	F	F	F	F	F	F	F	F	F	F	F	F	F	F	T	F	F	F	F	F
YGL005C	F	F	F	F	F	F	F	F	F	F	F	F	T	F	F	F	F	F	F	F	F	F	F
YGL008C	F	F	T	F	T	F	F	F	F	F	F	F	F	F	F	F	F	F	F	F	F	F	F
YGL009C	F	F	F	F	F	F	F	F	F	F	F	F	F	F	F	F	F	T	F	F	F	T	F
YGL011C	F	F	F	F	F	F	F	F	F	F	F	F	F	F	F	F	F	T	F	F	F	T	F
YGL012W	F	F	F	F	F	F	F	T	F	F	F	F	F	F	F	F	F	F	F	F	F	F	F
YGL013C	F	F	F	F	F	F	F	F	F	F	F	F	F	F	F	F	F	F	F	F	F	T	F
YGL015C	F	F	F	F	F	F	F	F	F	F	F	F	F	F	F	F	F	F	F	F	F	F	F
YGL016W	F	F	F	F	F	F	F	F	T	F	F	F	F	F	F	F	F	T	F	F	F	T	F
YGL017W	F	F	F	F	F	F	F	F	F	F	F	F	F	F	F	F	F	T	F	F	F	F	F
YGL019W	F	F	F	F	F	F	F	F	F	F	F	F	F	F	F	F	F	T	F	F	F	T	F
YGL020C	F	F	F	F	F	F	F	T	F	F	F	F	F	F	F	F	F	F	F	F	F	F	F
YGL022W	F	F	F	F	F	F	F	T	F	F	F	F	F	F	F	F	F	F	F	F	F	F	F
YGL025C	F	F	F	F	F	F	F	F	F	F	F	F	F	F	F	F	F	F	F	F	F	T	F
YGL026C	F	F	F	F	F	F	F	F	F	F	F	F	F	F	F	F	F	T	F	F	F	T	F
YGL030W	F	F	F	F	F	F	F	F	F	F	F	F	F	F	F	F	F	F	F	F	F	F	F
YGL031C	F	F	F	F	F	F	F	F	F	F	F	F	F	F	F	F	F	T	F	F	F	F	F
YGL035C	F	F	F	F	F	F	F	F	F	F	F	F	F	F	F	F	F	T	F	F	F	T	F
YGL037C	F	F	F	F	F	F	F	F	F	F	F	F	F	F	T	F	F	F	F	F	F	F	F
YGL043W	F	F	F	F	F	F	F	F	F	F	F	F	F	F	F	F	F	F	F	F	F	T	F
YGL044C	F	F	F	F	F	F	F	F	F	F	F	F	F	F	F	F	F	F	F	F	F	F	F
YGL045W	F	F	F	F	F	F	F	F	F	F	F	F	F	F	F	F	F	F	F	F	F	F	F
YGL048C	F	F	F	F	F	F	F	F	F	F	F	F	F	F	F	F	F	F	F	F	F	T	F
YGL049C	F	F	F	F	F	F	F	F	F	F	F	F	F	F	F	F	F	T	F	F	F	F	F
YGL055W	F	F	F	F	F	F	F	T	F	F	F	F	F	F	F	F	F	F	F	F	F	F	F
YGL058W	F	F	F	F	F	F	F	F	F	F	F	F	F	F	F	F	F	T	F	F	F	T	F

YGL060W	F	F	F	F	F	F	F	F	F	F	F	F	F	F	F	F	F	T	F	F	F	F	F
YGL061C	F	F	F	T	F	F	F	F	F	F	F	F	F	F	F	F	F	F	F	F	F	F	F
YGL064C	F	T	F	F	F	F	F	F	F	F	F	F	F	F	F	F	F	F	F	F	F	F	F
YGL066W	F	F	F	F	F	F	F	F	F	F	F	F	F	F	F	F	F	F	F	F	F	T	F
YGL070C	F	F	F	F	F	F	F	F	F	F	F	F	F	F	F	F	F	F	F	F	F	T	F
YGL071W	F	F	F	F	F	F	F	F	F	F	F	F	F	F	F	F	F	T	F	F	F	T	F
YGL073W	F	F	F	F	F	F	F	F	F	F	F	F	F	F	F	F	F	F	F	F	F	T	F
YGL075C	F	F	F	T	F	F	F	F	F	F	F	F	F	F	F	F	F	F	F	F	F	F	F
YGL076C	F	F	F	F	F	F	F	F	F	F	F	F	F	F	F	F	F	T	F	F	F	F	F
YGL078C	F	F	F	F	F	F	F	F	F	F	F	F	F	F	F	F	T	F	F	F	F	T	F
YGL079W	F	F	F	F	F	F	F	F	F	T	F	F	F	F	F	F	F	F	F	F	F	F	F
YGL086W	F	F	F	F	F	F	F	F	T	F	F	F	F	F	F	F	F	F	F	F	F	F	F
YGL087C	F	F	F	F	F	F	F	F	F	F	F	F	F	F	F	F	F	T	F	F	F	T	F
YGL090W	F	F	F	F	F	F	F	F	F	F	F	F	F	F	F	F	F	T	F	F	F	T	F
YGL092W	F	F	F	F	F	F	F	F	T	F	F	F	F	F	F	F	F	F	F	F	F	F	F
YGL093W	F	F	F	T	F	F	F	F	F	F	F	F	F	F	F	F	F	F	F	F	F	F	F
YGL095C	F	F	F	F	F	F	F	F	F	F	F	F	F	T	F	F	F	F	F	T	F	F	F
YGL096W	F	F	F	F	F	F	F	F	F	F	F	F	F	F	F	F	F	F	F	F	F	F	F
YGL097W	F	F	F	F	F	F	F	F	F	F	F	F	F	F	F	F	F	F	F	F	F	T	F
YGL098W	F	F	F	F	F	F	F	F	F	F	F	F	F	F	F	F	F	F	F	F	T	F	F
YGL099W	F	F	F	F	F	F	F	F	F	F	F	F	F	F	F	F	F	T	F	F	F	F	F
YGL100W	F	F	F	F	F	F	F	F	F	F	F	F	F	F	F	F	F	F	F	F	F	F	F
YGL103W	F	F	F	F	F	F	F	F	F	F	F	F	F	F	F	F	F	F	F	F	F	F	F
YGL105W	F	F	F	F	F	F	F	F	F	F	F	F	F	F	F	F	F	T	F	F	F	F	F
YGL106W	F	F	F	F	F	F	F	F	F	F	F	F	F	F	F	F	F	F	F	F	F	F	F
YGL108C	F	F	F	F	T	F	F	F	F	F	F	F	F	F	F	F	F	F	F	F	F	F	F
YGL111W	F	F	F	F	F	F	F	F	F	F	F	F	F	F	F	F	T	F	F	F	F	T	F
YGL112C	F	F	F	F	F	F	F	F	F	F	F	F	F	F	F	F	F	F	F	F	F	T	F
YGL113W	F	F	F	F	F	F	F	F	F	F	F	F	F	F	F	F	F	T	F	F	F	T	F
YGL115W	F	F	F	F	F	F	F	F	F	F	F	F	F	F	F	F	F	T	F	F	F	F	F
YGL116W	F	F	F	F	F	F	F	F	F	F	F	F	F	F	F	F	F	T	F	F	F	T	F
YGL120C	F	F	F	F	F	F	F	F	F	F	F	F	F	F	F	F	T	F	F	F	F	F	F
YGL122C	F	F	F	F	F	F	F	F	F	F	F	F	F	F	F	F	F	F	F	F	F	T	F
YGL123W	F	F	F	F	F	F	F	F	F	F	F	F	F	F	F	F	F	F	F	F	F	F	F
YGL124C	F	F	F	F	F	F	F	F	F	F	F	F	F	T	F	F	F	F	F	F	F	F	F
YGL125W	F	F	F	F	F	F	F	F	F	F	F	F	F	F	F	F	F	T	F	F	F	T	F
YGL127C	F	F	F	F	F	F	F	F	F	F	F	F	F	F	F	F	F	F	F	F	F	T	F
YGL128C	F	F	F	F	F	F	F	F	F	F	F	F	F	F	F	F	F	T	F	F	F	T	F
YGL129C	F	T	F	F	F	F	F	F	F	F	F	F	F	F	F	F	F	F	F	F	F	F	F
YGL130W	F	F	F	F	F	F	F	F	F	F	F	F	F	F	F	F	F	T	F	F	F	T	F
YGL131C	F	F	F	F	F	F	F	F	F	F	F	F	F	F	F	F	F	T	F	F	F	T	F
YGL133W	F	F	F	F	F	F	F	F	F	F	F	F	F	F	F	F	F	F	F	F	F	T	F
YGL134W	F	F	F	F	F	F	F	F	F	F	F	F	F	F	F	F	F	T	F	F	F	F	F
YGL135W	F	F	F	F	F	F	F	F	F	F	F	F	F	F	F	F	F	T	F	F	F	F	F
YGL137W	F	F	F	F	F	F	F	F	F	F	F	F	T	F	F	F	F	F	F	T	F	F	F
YGL144C	F	F	F	F	F	F	F	F	F	F	F	F	F	F	F	F	F	F	F	F	F	F	F
YGL145W	F	F	F	F	F	F	F	T	F	F	F	F	F	F	F	F	F	F	F	F	F	F	F
YGL147C	F	F	F	F	F	F	F	F	F	F	F	F	F	F	F	F	F	T	F	F	F	F	F
YGL148W	F	F	F	F	F	F	F	F	F	F	F	F	F	F	F	F	F	T	F	F	F	F	F
YGL150C	F	F	F	F	F	F	F	F	F	F	F	F	F	F	F	F	F	F	F	F	F	T	F
YGL151W	F	F	F	F	F	F	F	F	F	F	F	F	F	F	F	F	F	F	F	F	F	T	F
YGL153W	F	F	F	F	F	F	F	F	F	F	F	F	F	F	T	F	F	F	F	F	F	F	F
YGL154C	F	F	F	F	F	F	F	F	F	F	F	F	F	F	F	F	F	F	F	F	F	F	F
YGL156W	F	F	F	F	F	T	F	F	F	F	F	F	F	F	F	F	F	F	F	F	F	F	F
YGL157W	F	F	F	F	F	F	F	F	F	F	F	F	F	F	F	F	F	T	F	F	F	T	F
YGL158W	F	F	F	F	F	F	F	F	F	F	F	F	F	F	F	F	F	F	F	F	F	F	F
YGL161C	F	F	F	F	F	F	F	F	F	F	F	F	F	F	F	F	F	T	F	F	F	F	F
YGL162W	F	F	F	F	F	F	F	F	F	F	F	F	F	F	F	F	F	T	F	F	F	T	F
YGL163C	F	F	F	F	F	F	F	F	F	F	F	F	F	F	F	F	F	T	F	F	F	T	F
YGL164C	F	F	F	F	F	F	F	F	F	F	F	F	F	F	F	F	F	T	F	F	F	F	F
YGL170C	F	F	F	F	F	F	F	F	F	F	F	F	F	F	F	F	F	F	F	F	F	F	F
YGL171W	F	F	F	F	F	F	F	F	F	F	F	F	F	F	F	F	F	F	F	F	F	F	F
YGL172W	F	F	F	F	F	F	F	F	T	F	F	F	F	F	F	F	F	F	F	F	F	F	F
YGL173C	F	F	F	F	F	F	F	F	F	F	F	F	F	F	F	F	F	T	F	F	F	F	F
YGL174W	F	F	F	F	F	F	F	F	F	F	F	F	F	F	F	F	F	T	F	F	F	T	F
YGL175C	F	F	F	F	F	F	F	F	F	F	F	F	F	F	F	F	F	T	F	F	F	T	F
YGL178W	F	F	F	F	F	F	F	F	F	F	F	F	F	F	F	F	F	T	F	F	F	F	F
YGL180W	F	F	F	F	F	T	F	F	F	F	F	F	F	F	F	F	F	F	F	F	F	F	F

YGL181W	F	F	F	F	F	F	F	F	F	F	F	F	F	F	F	T	F	T	F	F	F	F	F
YGL187C	F	T	F	F	F	F	F	F	F	F	F	F	F	F	F	F	F	F	F	F	F	F	F
YGL189C	F	F	F	F	F	F	F	F	F	F	F	F	F	F	F	F	F	T	F	F	F	F	F
YGL190C	F	F	F	F	F	F	F	F	F	F	F	F	F	F	F	F	F	T	F	F	F	F	F
YGL194C	F	F	F	F	F	F	F	F	F	F	F	F	F	F	F	F	F	T	F	F	F	T	F
YGL195W	F	F	F	F	F	F	F	F	F	F	F	F	F	F	F	F	F	T	F	F	F	F	F
YGL197W	F	F	F	F	F	F	F	F	F	F	F	F	F	F	F	F	F	T	F	F	F	F	F
YGL198W	F	F	F	F	F	F	F	F	F	F	F	F	F	F	F	F	F	F	F	F	F	F	F
YGL200C	F	F	F	F	F	T	F	F	F	F	F	F	F	F	F	F	F	F	F	F	F	F	F
YGL201C	F	F	F	F	F	F	F	F	F	F	F	F	F	F	F	F	F	T	F	F	F	T	F
YGL202W	F	F	F	F	F	F	F	F	F	F	F	F	F	F	F	F	F	T	F	F	F	F	F
YGL205W	F	F	F	F	F	F	F	F	F	F	F	F	F	F	F	F	F	F	F	F	F	F	F
YGL206C	F	F	F	F	F	F	F	F	F	F	F	F	F	T	F	F	F	F	F	F	F	F	F
YGL207W	F	F	F	F	F	F	F	F	F	F	F	F	F	F	F	F	F	F	F	F	F	T	F
YGL208W	F	F	F	F	F	F	F	F	F	F	F	F	F	F	F	F	F	F	F	F	F	F	F
YGL210W	F	F	F	F	F	F	F	F	F	F	F	F	F	F	F	F	F	T	F	F	F	T	F
YGL211W	F	T	F	F	F	F	F	F	F	F	F	F	F	F	F	F	F	F	F	F	F	F	F
YGL212W	F	F	T	F	F	F	F	F	F	F	F	F	F	F	F	F	F	T	F	F	F	F	F
YGL213C	F	F	F	F	F	F	F	F	F	F	F	F	F	F	F	F	F	T	F	F	F	F	F
YGL215W	F	F	F	F	F	F	F	F	F	F	F	F	F	F	F	F	F	F	F	F	F	F	F
YGL220W	F	F	F	F	F	F	F	F	F	F	F	F	F	F	F	F	F	T	F	F	F	T	F
YGL223C	F	F	F	F	F	F	F	F	F	F	F	F	F	F	F	F	F	F	F	T	F	F	F
YGL226C-A	F	F	F	F	F	F	F	T	F	F	F	F	F	F	F	F	F	F	F	F	F	F	F
YGL227W	F	F	F	F	F	F	F	F	F	F	F	F	F	F	F	F	F	T	F	F	F	T	F
YGL229C	F	F	F	F	F	F	F	F	F	F	F	F	F	F	F	F	F	T	F	F	F	F	F
YGL232W	F	F	F	F	F	F	F	F	F	F	F	F	F	F	F	F	F	T	F	F	F	T	F
YGL233W	F	F	F	F	T	F	F	F	F	F	T	F	F	F	F	F	F	T	F	F	F	F	F
YGL234W	F	F	F	F	F	F	F	F	F	F	F	F	F	F	F	F	F	T	F	F	F	F	F
YGL237C	F	F	F	F	F	F	F	F	F	F	F	F	F	F	F	F	F	F	F	F	F	T	F
YGL238W	F	F	F	F	F	F	F	F	F	F	F	F	F	F	F	F	F	T	F	F	F	T	F
YGL240W	F	F	F	F	F	F	F	F	F	F	F	F	F	F	F	F	F	T	F	F	F	T	F
YGL241W	F	F	F	F	F	F	F	F	F	F	F	F	F	F	F	F	F	T	F	F	F	T	F
YGL242C	F	F	F	F	F	F	F	F	F	F	F	F	F	F	F	F	F	F	F	F	F	F	F
YGL244W	F	F	F	F	F	F	F	F	F	F	F	F	F	F	F	F	F	F	F	F	F	T	F
YGL245W	F	F	F	F	F	F	F	F	F	F	F	F	F	F	F	F	F	T	F	F	F	F	F
YGL246C	F	F	F	F	F	F	F	F	F	F	F	F	F	F	F	F	F	F	F	F	F	T	F
YGL249W	T	F	F	F	F	F	F	F	F	F	F	F	F	F	F	F	F	F	F	F	F	F	F
YGL250W	F	F	F	F	F	F	F	F	F	F	F	F	F	F	F	F	F	T	F	F	F	T	F
YGL252C	F	F	F	F	F	F	F	F	F	F	F	F	F	F	F	F	F	T	F	F	F	F	F
YGL253W	F	F	F	F	F	F	F	F	F	F	F	F	F	F	F	F	F	T	F	F	F	F	F
YGL254W	F	F	F	F	F	F	F	F	F	F	F	F	F	F	F	F	F	T	F	F	F	T	F
YGR002C	F	F	F	F	F	F	F	F	F	F	F	F	F	F	F	F	F	F	F	F	F	T	F
YGR003W	F	F	F	F	F	F	F	F	F	F	F	F	F	F	F	F	F	T	F	F	F	T	F
YGR004W	T	F	F	F	F	F	F	F	F	F	F	F	F	F	F	F	F	F	F	F	F	F	F
YGR005C	F	F	F	F	F	F	F	F	F	F	F	F	F	F	F	F	F	F	F	F	F	F	F
YGR009C	F	F	F	F	F	F	F	F	F	F	F	F	F	F	F	F	F	F	F	F	F	F	F
YGR010W	F	F	F	F	F	F	F	F	F	F	F	F	F	F	F	F	F	T	F	F	F	T	F
YGR013W	F	F	F	F	F	F	F	F	F	F	F	F	F	F	F	F	F	T	F	F	F	T	F
YGR014W	F	F	T	F	F	F	F	F	F	F	F	F	F	F	F	F	F	F	F	F	F	F	F
YGR017W	F	F	F	F	F	F	F	F	F	F	F	F	F	F	F	F	F	T	F	F	F	T	F
YGR020C	T	F	F	F	F	F	T	F	F	F	F	F	F	F	F	F	F	F	F	F	F	F	F
YGR023W	F	F	F	F	F	F	F	F	F	F	F	F	F	F	F	F	F	F	F	F	F	F	F
YGR024C	F	F	F	F	F	F	F	F	F	F	F	F	F	F	F	F	F	T	F	F	F	T	F
YGR027C	F	F	F	F	F	F	F	F	F	F	F	F	F	F	F	F	F	T	F	F	F	F	F
YGR029W	F	F	F	F	F	F	F	F	F	F	F	F	F	F	F	F	F	F	F	F	F	F	F
YGR030C	F	F	F	F	F	F	F	F	F	F	F	F	F	F	F	F	F	F	F	F	F	T	F
YGR032W	F	F	F	F	F	F	F	F	F	F	F	F	F	F	F	F	F	F	F	F	F	F	F
YGR033C	F	T	F	F	F	F	F	F	F	F	F	F	F	F	F	F	F	F	F	F	F	F	F
YGR038W	F	F	F	F	F	F	F	T	F	F	F	F	F	F	F	F	F	F	F	F	F	F	F
YGR040W	F	F	F	F	F	F	F	F	F	F	F	F	F	F	F	F	F	T	F	F	F	T	F
YGR041W	T	F	F	F	F	F	F	F	F	F	T	F	F	F	F	F	F	F	F	F	F	F	T
YGR047C	F	F	F	F	F	F	F	F	F	F	F	F	F	F	F	F	F	T	F	F	F	T	F
YGR048W	F	F	F	F	F	F	F	F	F	F	F	F	F	F	F	F	F	T	F	F	F	T	F
YGR052W	F	F	F	F	F	F	F	F	F	F	F	F	F	F	F	F	F	T	F	F	F	T	F
YGR054W	F	F	F	F	F	F	F	F	F	F	F	F	F	F	F	F	F	F	F	F	F	F	F
YGR056W	F	F	F	F	F	F	F	F	F	F	F	F	F	F	F	F	F	F	F	F	F	F	F
YGR058W	F	F	F	F	F	F	F	F	F	F	F	F	F	F	F	F	F	T	F	F	F	T	F
YGR060W	F	F	T	F	F	F	F	T	F	F	F	F	F	F	F	F	F	F	F	F	F	F	F

YGR061C	F	F	F	F	F	F	F	F	F	F	F	F	F	F	F	F	F	T	F	F	F	F	F
YGR063C	F	F	F	F	F	F	F	F	F	F	F	F	F	F	F	F	F	F	F	F	F	T	F
YGR068C	T	F	F	F	F	F	F	F	F	F	F	F	F	F	F	F	F	F	F	F	F	F	F
YGR071C	F	F	F	F	F	F	F	F	F	F	F	F	F	F	F	F	F	F	F	F	F	T	F
YGR072W	F	F	F	F	F	F	F	F	F	F	F	F	F	F	F	F	F	T	F	F	F	F	F
YGR074W	F	F	F	F	F	F	F	F	F	F	F	F	F	F	F	F	F	F	F	F	F	T	F
YGR075C	F	F	F	F	F	F	F	F	F	F	F	F	F	F	F	F	F	F	F	F	F	T	F
YGR076C	F	T	F	F	F	F	F	F	F	F	F	F	F	F	F	F	F	F	F	F	F	F	F
YGR077C	F	F	F	F	F	F	F	F	F	F	F	F	F	F	F	F	F	F	F	F	F	F	F
YGR078C	F	F	F	F	F	F	F	F	F	F	F	F	F	F	F	F	F	T	F	F	F	F	F
YGR080W	F	F	F	F	F	F	F	F	F	F	F	F	F	F	F	T	F	F	F	F	F	F	F
YGR081C	F	F	F	F	F	F	F	F	F	F	F	F	F	F	F	F	T	F	F	F	F	T	F
YGR082W	F	T	F	F	F	F	F	F	F	F	F	F	F	F	F	F	F	F	F	F	F	F	F
YGR083C	F	F	F	F	F	F	F	F	F	F	F	F	F	F	F	F	F	T	F	F	F	F	F
YGR084C	F	T	F	F	F	F	F	F	F	F	F	F	F	F	F	F	F	F	F	F	F	F	F
YGR085C	F	F	F	F	F	F	F	F	F	F	F	F	F	F	F	F	F	T	F	F	F	F	F
YGR086C	F	F	F	F	F	T	F	F	F	F	F	F	F	F	F	F	F	F	F	F	F	F	F
YGR089W	F	F	F	F	F	F	F	T	F	F	F	F	F	F	F	F	F	F	F	F	F	F	F
YGR090W	F	F	F	F	F	F	F	F	F	F	F	F	F	F	F	F	T	F	F	F	F	T	F
YGR091W	F	F	F	F	F	F	F	F	F	F	F	F	F	F	F	F	F	T	F	F	F	T	F
YGR092W	F	F	F	F	F	F	F	F	F	F	T	F	F	F	F	F	F	T	F	F	F	F	F
YGR094W	F	F	F	F	F	F	F	F	F	F	F	F	F	F	F	F	F	T	F	F	F	F	F
YGR095C	F	F	F	F	F	F	F	F	F	F	F	F	F	F	F	F	T	T	F	F	F	T	F
YGR097W	F	F	F	F	F	F	F	F	F	F	F	F	F	F	F	F	F	T	F	F	F	F	F
YGR098C	F	F	F	F	F	F	F	F	F	F	F	F	F	F	F	F	F	T	F	F	F	T	F
YGR099W	F	F	F	F	F	F	F	F	F	F	F	F	F	F	F	F	F	F	F	F	F	F	F
YGR100W	F	F	F	F	F	F	F	F	F	F	F	F	F	F	F	F	F	T	F	F	F	F	F
YGR103W	F	F	F	F	F	F	F	F	F	F	F	F	F	F	F	F	T	F	F	F	F	T	F
YGR104C	F	F	F	F	F	F	F	F	F	F	F	F	F	F	F	F	F	F	F	F	F	T	F
YGR105W	F	F	T	F	F	F	F	F	F	F	F	F	F	F	F	F	F	F	F	F	F	F	F
YGR106C	F	F	F	F	F	F	T	F	F	F	F	F	F	F	F	F	F	F	F	F	F	F	F
YGR108W	F	F	F	F	F	F	F	F	F	F	F	F	F	F	F	F	F	F	F	F	F	T	F
YGR112W	F	T	F	F	F	F	F	F	F	F	F	F	F	F	F	F	F	F	F	F	F	F	F
YGR113W	F	F	F	F	F	F	F	F	F	F	F	F	F	F	F	F	F	F	F	F	F	F	F
YGR116W	F	F	F	F	F	F	F	F	F	F	F	F	F	F	F	F	F	F	F	F	F	T	F
YGR119C	F	F	F	F	F	F	F	F	T	F	F	F	F	F	F	F	F	F	F	F	F	F	F
YGR120C	F	F	F	F	F	F	F	F	F	F	F	F	T	F	F	F	F	F	F	T	F	F	F
YGR122W	F	F	F	F	F	F	F	F	F	F	F	F	F	F	F	F	F	T	F	F	F	F	F
YGR123C	F	F	F	F	F	F	F	F	F	F	F	F	F	F	F	F	F	T	F	F	F	F	F
YGR124W	F	F	F	F	F	F	F	F	F	F	F	F	F	F	F	F	F	T	F	F	F	F	F
YGR128C	F	F	F	F	F	F	F	F	F	F	F	F	F	F	F	F	T	F	F	F	F	F	F
YGR129W	F	F	F	F	F	F	F	F	F	F	F	F	F	F	F	F	F	F	F	F	F	T	F
YGR130C	F	F	F	F	F	T	F	F	F	F	F	F	F	F	F	F	F	F	F	F	F	F	F
YGR132C	F	T	F	F	F	F	F	F	F	F	F	F	F	F	F	F	F	F	F	F	F	F	F
YGR134W	F	F	F	F	F	F	F	F	F	F	F	F	F	F	F	F	F	T	F	F	F	F	F
YGR135W	F	F	F	F	F	F	F	F	F	F	F	F	F	F	F	F	F	T	F	F	F	T	F
YGR136W	F	F	F	F	F	F	F	F	F	F	F	F	F	F	F	F	F	T	F	F	F	T	F
YGR140W	F	F	F	T	F	F	F	F	F	F	F	T	F	F	F	F	F	F	F	F	F	T	F
YGR142W	F	F	F	F	F	F	F	F	F	F	F	F	F	F	F	F	F	T	F	F	F	F	F
YGR143W	F	F	F	F	F	F	F	F	F	F	F	F	F	F	F	F	F	F	F	F	F	F	F
YGR144W	F	F	F	F	F	F	F	F	F	F	F	F	F	F	F	F	F	F	F	F	F	F	F
YGR145W	F	F	F	F	F	F	F	F	F	F	F	F	F	F	F	F	F	F	F	F	F	F	F
YGR148C	F	F	F	F	F	F	F	F	F	F	F	F	F	F	F	F	F	T	F	F	F	F	F
YGR152C	F	F	F	F	F	F	F	F	F	F	F	F	F	F	F	F	F	T	F	F	F	T	F
YGR155W	F	F	F	F	F	F	F	F	F	F	F	F	F	F	F	F	F	T	F	F	F	F	F
YGR156W	F	F	F	F	F	F	F	F	F	F	F	F	F	F	F	F	F	F	F	F	F	T	F
YGR158C	F	F	F	F	F	F	F	F	F	F	F	F	F	F	F	F	F	T	F	F	F	T	F
YGR159C	F	F	F	F	F	F	F	F	F	F	F	F	F	F	F	F	T	F	F	F	F	F	F
YGR161C	F	F	F	F	F	F	F	F	F	F	F	F	F	F	F	F	F	T	F	F	F	T	F
YGR162W	F	F	F	F	F	F	F	F	F	F	F	F	F	F	F	F	F	T	F	F	F	F	F
YGR163W	F	F	F	F	F	F	T	F	F	F	F	F	F	F	F	F	F	F	F	F	F	F	F
YGR165W	F	T	F	F	F	F	F	F	F	F	F	F	F	F	F	F	F	F	F	F	F	F	F
YGR166W	F	F	F	F	F	T	F	F	F	F	F	F	F	F	F	F	F	F	F	F	F	F	F
YGR167W	F	F	F	F	F	F	F	F	F	F	F	F	F	T	F	F	F	F	F	F	F	F	F
YGR170W	F	F	F	F	F	F	F	F	F	F	F	F	F	F	F	F	F	F	F	F	F	F	F
YGR172C	F	F	F	F	F	F	F	F	F	F	F	F	F	F	F	F	F	F	F	F	F	F	F
YGR173W	F	F	F	F	F	F	F	F	F	F	F	F	F	F	F	F	F	T	F	F	F	F	F
YGR175C	F	F	F	F	F	F	F	T	F	F	F	F	F	F	F	F	F	F	F	F	F	F	F

YGR178C	F	F	F	F	F	F	F	F	F	F	F	F	F	F	F	F	F	T	F	F	F	T	F
YGR179C	F	F	F	T	F	F	F	F	F	F	F	F	F	F	F	F	F	F	F	F	F	F	F
YGR180C	F	F	F	F	F	F	F	F	F	F	F	F	F	F	F	F	F	T	F	F	F	T	F
YGR181W	F	F	F	F	F	F	F	F	F	F	F	F	F	F	F	F	F	F	F	F	F	F	F
YGR184C	F	F	F	F	F	F	F	F	F	F	F	F	F	F	F	F	F	F	F	F	F	F	F
YGR185C	F	F	F	F	F	F	F	F	F	F	F	F	F	F	F	F	F	T	F	F	F	F	F
YGR186W	F	F	F	F	F	F	F	F	F	F	F	F	F	F	F	F	F	F	F	F	F	T	F
YGR187C	F	F	F	F	F	F	F	F	F	F	F	F	F	F	F	F	F	T	F	F	F	F	F
YGR188C	F	F	F	T	F	F	F	F	F	F	F	T	F	F	F	F	F	F	F	F	F	T	F
YGR191W	F	F	T	F	T	F	F	T	F	F	F	F	F	F	F	F	F	F	F	F	F	F	T
YGR192C	F	F	F	F	F	F	F	F	F	F	F	F	F	F	F	F	F	T	F	F	F	T	F
YGR193C	F	T	F	F	F	F	F	F	F	F	F	F	F	F	F	F	F	F	F	F	F	F	F
YGR195W	F	F	F	F	F	F	F	F	F	F	F	F	F	F	F	F	T	F	F	F	F	T	F
YGR196C	F	F	F	F	F	F	F	F	F	F	F	F	F	F	F	F	F	F	F	F	F	F	F
YGR198W	F	F	F	F	F	T	F	F	F	F	F	F	F	F	F	F	F	F	F	F	F	F	F
YGR200C	F	F	F	F	F	F	F	F	F	F	F	F	F	F	F	F	F	T	F	F	F	F	F
YGR202C	F	F	F	F	F	F	F	F	T	F	F	F	F	F	F	F	F	F	F	F	F	F	F
YGR203W	F	F	F	F	F	F	F	F	F	F	F	F	F	F	F	F	F	T	F	F	F	T	F
YGR204W	F	F	F	F	F	F	F	F	F	F	F	F	F	F	F	F	F	T	F	F	F	T	F
YGR205W	F	F	F	F	F	F	F	F	F	F	F	F	F	F	F	F	F	T	F	F	F	T	F
YGR206W	F	F	F	F	F	F	F	F	F	T	F	F	F	F	F	F	F	F	F	F	F	F	F
YGR209C	F	F	F	F	F	F	F	F	F	F	F	F	F	F	F	F	F	T	F	F	F	T	F
YGR211W	F	F	F	F	F	F	F	F	F	F	F	F	F	F	F	F	F	T	F	F	F	T	F
YGR214W	F	F	F	F	F	F	F	F	F	F	F	F	F	F	F	F	F	T	F	F	F	F	F
YGR215W	F	T	F	F	F	F	F	F	F	F	F	F	F	F	F	F	F	F	F	F	F	F	F
YGR218W	F	F	F	F	F	F	F	F	F	F	F	F	F	F	F	F	F	F	F	F	F	T	F
YGR220C	F	T	F	F	F	F	F	F	F	F	F	F	F	F	F	F	F	F	F	F	F	F	F
YGR221C	F	F	F	F	F	F	F	F	F	F	F	F	F	F	F	F	F	F	F	F	F	F	F
YGR229C	F	F	F	F	F	T	F	F	F	F	F	F	F	T	F	F	F	F	F	F	F	F	F
YGR231C	F	T	F	F	F	F	F	F	F	F	F	F	F	F	F	F	F	F	F	F	F	F	F
YGR232W	F	F	F	F	F	F	F	F	F	F	F	F	F	F	F	F	F	T	F	F	F	T	F
YGR233C	F	F	F	F	F	F	F	F	F	F	F	F	F	F	F	F	F	T	F	F	F	T	F
YGR234W	F	F	F	F	F	F	F	F	F	F	F	F	F	F	F	F	F	T	F	F	F	F	F
YGR237C	F	F	F	F	F	F	F	F	F	F	F	F	F	F	F	F	F	T	F	F	F	F	F
YGR238C	T	F	F	F	T	F	F	F	F	F	F	F	F	F	F	F	F	F	F	F	F	F	T
YGR239C	F	F	F	F	F	F	F	F	F	F	F	F	F	F	T	F	F	F	F	F	F	F	F
YGR240C	F	F	F	F	F	F	F	F	F	F	F	F	F	F	F	F	F	T	F	F	F	F	F
YGR241C	T	F	F	F	T	F	F	F	F	F	T	F	F	F	F	F	F	F	F	F	F	F	T
YGR245C	F	F	F	F	F	F	F	F	F	F	F	F	F	F	F	F	F	F	F	F	F	F	F
YGR246C	F	F	F	F	F	F	F	F	F	F	F	F	F	F	F	F	F	F	F	F	F	T	F
YGR249W	F	F	F	F	F	F	F	F	F	F	F	F	F	F	F	F	F	F	F	F	F	F	F
YGR250C	F	F	F	F	F	F	F	F	F	F	F	F	F	F	F	F	F	T	F	F	F	F	F
YGR252W	F	F	F	F	F	F	F	F	F	F	F	F	F	F	F	F	F	F	F	F	F	T	F
YGR253C	F	F	F	F	F	F	F	F	F	F	F	F	F	F	F	F	F	T	F	F	F	T	F
YGR254W	F	F	F	F	F	F	F	F	F	F	F	F	F	F	F	F	F	T	F	F	F	F	F
YGR258C	F	F	F	F	F	F	F	F	F	F	F	F	F	F	F	F	F	T	F	F	F	T	F
YGR261C	F	F	F	F	F	T	F	F	F	F	F	F	T	F	F	F	F	F	F	T	F	F	F
YGR262C	F	F	F	F	F	F	F	F	F	F	F	F	F	F	F	F	F	T	F	F	F	T	F
YGR264C	F	F	F	F	F	F	F	F	F	F	F	F	F	F	F	F	F	T	F	F	F	F	F
YGR267C	F	F	F	F	F	F	F	F	F	F	F	F	F	F	F	F	F	T	F	F	F	T	F
YGR268C	F	F	F	F	F	F	F	F	F	F	F	F	F	F	F	F	F	T	F	F	F	T	F
YGR270W	F	F	F	F	F	F	F	F	F	F	F	F	F	F	F	F	F	F	F	F	F	T	F
YGR274C	F	F	F	F	F	F	F	F	F	F	F	F	F	F	F	F	F	F	F	F	F	T	F
YGR275W	F	F	F	F	F	F	F	F	F	F	F	F	F	F	F	F	F	F	F	F	F	T	F
YGR278W	F	F	F	F	F	F	F	F	F	F	F	F	F	F	F	F	F	T	F	F	F	T	F
YGR280C	F	F	F	F	F	F	F	F	F	F	F	F	F	F	F	F	T	F	F	F	F	F	F
YGR281W	F	F	F	F	T	F	F	F	F	F	F	F	F	F	F	F	F	F	F	F	F	F	F
YGR283C	F	F	F	F	F	F	F	F	F	F	F	F	F	F	F	F	T	F	F	F	F	F	F
YGR284C	F	F	F	F	F	F	F	T	F	F	F	F	F	F	F	F	F	F	F	F	F	F	F
YGR285C	F	F	F	F	F	F	F	F	F	F	F	F	F	F	F	F	F	T	F	F	F	F	F
YHL001W	F	F	F	F	F	F	F	F	F	F	F	F	F	F	F	F	F	T	F	F	F	F	F
YHL002W	F	F	F	F	F	F	F	F	F	T	F	F	F	F	F	F	F	F	F	F	F	F	F
YHL003C	F	F	F	F	F	F	F	T	F	F	F	F	F	F	F	F	F	F	F	F	F	F	F
YHL004W	F	T	F	F	F	F	F	F	F	F	F	F	F	F	F	F	F	F	F	F	F	F	F
YHL007C	F	F	F	F	F	F	F	F	F	F	F	F	F	F	F	F	F	T	F	F	F	F	T
YHL008C	F	F	T	F	F	F	F	F	F	F	F	F	F	F	F	F	F	F	F	F	F	F	F
YHL009C	F	F	F	F	F	F	F	F	F	F	F	F	F	F	F	F	F	T	F	F	F	T	F
YHL010C	F	F	F	F	F	F	F	F	F	F	F	F	F	F	F	F	F	F	F	F	F	F	F

YHL011C	F	F	F	F	F	F	F	F	F	F	F	F	F	F	F	F	F	T	F	F	F	F	F
YHL014C	F	T	F	F	F	F	F	F	F	F	F	F	F	F	F	F	F	F	F	F	F	F	F
YHL015W	F	F	F	F	F	F	F	F	F	F	F	F	F	F	F	F	F	F	F	F	F	F	F
YHL018W	F	T	F	F	F	F	F	F	F	F	F	F	F	F	F	F	F	F	F	F	F	F	F
YHL019C	T	F	F	F	F	F	F	F	F	F	F	F	F	T	F	F	F	F	F	F	F	F	F
YHL020C	F	F	F	F	F	F	F	F	T	F	F	F	F	F	F	F	F	F	F	F	F	F	F
YHL022C	F	F	F	F	F	F	F	F	F	F	F	F	F	F	F	F	F	F	F	F	F	F	F
YHL024W	F	F	F	F	F	F	F	F	F	F	F	F	F	F	F	F	F	F	F	F	F	F	F
YHL025W	F	F	F	F	F	F	F	F	F	F	F	F	F	F	F	F	F	F	F	F	F	T	F
YHL027W	F	F	F	F	F	F	F	F	F	F	F	F	F	F	F	F	F	T	F	F	F	F	F
YHL029C	F	F	F	F	F	F	F	F	F	F	F	F	F	F	F	F	F	T	F	F	F	F	F
YHL030W	F	F	F	F	F	F	F	F	F	F	F	F	F	F	F	F	F	T	F	F	F	T	F
YHL031C	F	F	F	F	F	F	F	F	F	F	F	F	F	F	F	F	F	F	F	F	F	F	F
YHL033C	F	F	F	F	F	F	F	F	F	F	F	F	F	F	F	F	F	T	F	F	F	F	F
YHL034C	F	F	F	F	F	F	F	F	F	F	F	F	F	F	F	F	F	T	F	F	F	F	F
YHL039W	F	F	F	F	F	F	F	F	F	F	F	F	F	F	F	F	F	T	F	F	F	F	F
YHR001W	F	F	F	F	F	F	F	F	F	F	F	F	F	F	F	F	F	F	F	F	F	F	F
YHR005C	F	F	F	F	T	F	F	F	F	F	F	F	F	F	F	F	F	F	F	F	F	F	F
YHR005C-A	F	F	F	F	F	F	F	F	F	F	F	F	F	F	F	F	F	F	F	F	F	F	F
YHR007C	F	F	F	F	F	F	F	T	F	F	F	F	F	F	F	F	F	F	F	F	F	F	F
YHR009C	F	F	F	F	F	F	F	F	F	F	F	F	F	F	F	F	F	T	F	F	F	T	F
YHR010W	F	F	F	F	F	F	F	F	F	F	F	F	F	F	F	F	T	T	F	F	F	F	F
YHR012W	T	F	F	F	F	F	F	F	F	F	F	F	F	F	F	F	F	F	F	F	F	F	F
YHR013C	F	F	F	F	F	F	F	F	F	F	F	F	F	F	F	F	F	T	F	F	F	F	F
YHR014W	F	F	F	F	F	F	F	F	F	F	F	F	F	F	F	F	F	F	F	F	F	F	F
YHR016C	F	F	F	F	F	F	F	F	F	F	F	F	F	F	F	F	F	F	F	F	F	F	F
YHR019C	F	F	F	F	F	F	F	F	F	F	F	F	F	F	F	F	F	F	F	F	F	F	F
YHR020W	F	F	F	F	F	F	F	F	F	F	F	F	F	F	F	F	F	F	F	F	F	F	F
YHR021C	F	F	F	F	F	F	F	F	F	F	F	F	F	F	F	F	F	T	F	F	F	F	F
YHR023W	F	F	F	F	F	F	F	F	F	F	T	F	F	F	F	F	F	F	F	F	F	F	F
YHR024C	F	T	F	F	F	F	F	F	F	F	F	F	F	F	F	F	F	F	F	F	F	F	F
YHR025W	T	F	F	F	F	F	F	F	F	F	F	F	F	F	F	F	F	F	F	F	F	F	F
YHR026W	F	F	T	F	F	F	F	T	F	F	F	F	F	F	F	F	F	F	F	F	F	F	F
YHR027C	F	F	F	F	F	F	F	F	F	F	F	F	F	F	F	F	F	T	F	F	F	T	F
YHR030C	F	F	F	F	F	F	F	F	F	F	F	F	F	F	F	F	F	T	F	F	F	T	F
YHR031C	F	F	F	F	F	F	F	F	F	F	F	F	F	F	F	F	F	T	F	F	F	T	F
YHR034C	F	F	F	F	F	F	F	F	F	F	F	F	F	F	F	F	F	T	F	F	F	T	F
YHR035W	F	F	F	F	F	F	F	F	F	F	F	F	F	F	F	F	F	F	F	F	F	F	F
YHR039C	F	F	F	F	F	F	F	T	F	F	F	F	F	F	F	F	F	F	F	F	F	F	F
YHR039C-A	F	F	F	F	F	F	T	F	F	F	F	F	F	F	F	F	F	F	F	F	F	F	F
YHR041C	F	F	F	F	F	F	F	F	F	F	F	F	F	F	F	F	F	T	F	F	F	T	F
YHR042W	F	F	F	F	F	F	F	T	F	F	F	F	F	F	F	F	F	F	F	F	F	F	F
YHR047C	F	F	F	F	F	F	F	F	F	F	F	F	F	F	F	F	F	T	F	F	F	T	F
YHR051W	F	T	F	F	F	F	F	F	F	F	F	F	F	F	F	F	F	F	F	F	F	F	F
YHR052W	F	F	F	F	F	F	F	F	F	F	F	F	F	F	F	F	T	F	F	F	F	T	F
YHR056C	F	F	F	F	F	F	F	F	F	F	F	F	F	F	F	F	F	F	F	F	F	F	F
YHR057C	T	F	F	F	F	F	F	F	F	F	F	F	F	F	F	F	F	F	F	F	F	F	F
YHR058C	F	F	F	F	F	F	F	F	F	F	F	F	F	F	F	F	F	T	F	F	F	T	F
YHR060W	F	F	F	F	F	F	F	T	F	F	F	F	F	F	F	F	F	F	F	F	F	F	F
YHR061C	F	F	F	F	F	F	F	F	F	F	F	F	F	F	F	F	F	T	F	F	F	T	F
YHR062C	F	F	F	F	F	F	F	F	F	F	F	F	F	F	F	F	F	F	F	F	F	T	F
YHR063C	F	F	F	F	F	F	F	F	F	F	F	F	F	F	F	F	F	T	F	F	F	F	F
YHR064C	F	F	F	F	F	F	F	F	F	F	F	F	F	F	F	F	F	T	F	F	F	F	F
YHR066W	F	F	F	F	F	F	F	F	F	F	F	F	F	F	F	F	T	F	F	F	F	T	F
YHR068W	F	F	F	F	F	F	F	F	F	F	F	F	F	F	F	F	F	T	F	F	F	F	F
YHR069C	F	F	F	F	F	F	F	F	F	F	F	F	F	F	F	F	T	T	F	F	F	T	F
YHR071W	F	F	F	F	F	F	F	F	F	F	F	F	F	F	F	F	F	F	F	F	F	F	F
YHR072W	F	F	F	F	F	F	F	F	F	F	F	F	F	F	F	F	F	F	F	F	T	F	F
YHR072W-A	F	F	F	F	F	F	F	F	F	F	F	F	F	F	F	F	T	F	F	F	F	F	F
YHR074W	F	F	F	F	F	F	F	F	F	F	F	F	F	F	F	F	F	T	F	F	F	T	F
YHR076W	F	F	F	F	F	F	F	F	F	F	F	F	F	F	F	F	F	F	F	F	F	F	F
YHR077C	F	F	F	F	F	F	F	F	F	F	F	F	F	F	F	F	F	T	F	F	F	F	F
YHR079C	F	F	F	F	F	F	F	T	F	F	F	F	F	F	F	F	F	F	F	F	F	F	F
YHR079C-A	F	F	F	F	F	F	F	F	F	F	F	F	F	F	F	F	F	F	F	F	F	F	F
YHR080C	F	F	F	F	F	F	F	F	F	F	F	F	F	F	F	F	F	F	F	F	F	F	F
YHR081W	F	F	F	F	F	F	F	F	F	F	F	F	F	F	F	F	F	F	F	F	F	F	F
YHR082C	F	F	F	F	F	F	F	F	F	F	F	F	F	F	F	F	F	T	F	F	F	F	F
YHR083W	F	T	F	F	F	F	F	F	F	F	F	F	F	F	F	F	F	F	F	F	F	F	F

YHR084W	F	F	F	F	F	F	F	F	F	F	F	F	F	F	F	F	F	F	F	F	F	T	F
YHR085W	F	F	F	F	F	F	F	F	F	F	F	F	F	F	F	F	F	F	F	F	F	T	F
YHR086W	F	F	F	F	F	F	F	F	F	F	F	F	F	F	F	F	F	T	F	F	F	T	F
YHR088W	F	F	F	F	F	F	F	F	F	F	F	F	F	F	F	F	F	F	F	F	F	F	F
YHR089C	F	F	F	F	F	F	F	F	F	F	F	F	F	F	F	F	T	F	F	F	F	F	F
YHR090C	F	F	F	F	F	F	F	F	F	F	F	F	F	F	F	F	F	F	F	F	F	T	F
YHR094C	F	F	F	F	F	F	F	F	F	F	F	F	F	F	F	F	F	F	F	F	F	F	F
YHR097C	F	F	F	F	F	F	F	F	F	F	F	F	F	F	F	F	F	T	F	F	F	T	F
YHR098C	F	F	F	F	F	F	F	F	F	F	F	F	F	F	F	F	F	F	T	F	F	F	F
YHR099W	F	F	F	F	F	F	F	F	F	F	F	F	F	F	F	F	F	F	F	F	F	F	F
YHR102W	F	F	F	F	F	F	F	F	F	F	F	F	F	F	F	F	F	T	F	F	F	F	F
YHR104W	F	F	F	F	F	F	F	F	F	F	F	F	F	F	F	F	F	T	F	F	F	T	F
YHR105W	F	F	F	F	F	F	F	F	F	T	F	F	F	F	F	F	F	F	F	F	F	F	F
YHR107C	F	F	F	F	T	F	F	F	F	F	T	F	F	F	F	F	F	F	F	F	F	F	F
YHR108W	F	F	F	F	F	F	F	F	F	F	F	F	F	T	F	F	F	F	F	F	F	F	F
YHR110W	F	F	F	F	F	F	F	T	F	F	F	F	F	F	F	F	F	F	F	F	F	F	F
YHR111W	F	F	F	F	F	F	F	F	F	F	F	F	F	F	F	F	F	T	F	F	F	F	F
YHR113W	F	F	F	F	F	F	F	F	F	F	F	F	F	F	F	F	F	T	F	F	F	F	F
YHR114W	F	F	F	F	F	F	F	F	F	F	F	F	F	F	F	T	F	F	F	F	F	F	F
YHR115C	T	F	F	F	F	F	F	F	F	F	F	F	F	F	F	F	F	F	F	F	F	F	F
YHR117W	F	T	F	F	F	F	F	F	F	F	F	F	F	F	F	F	F	F	F	F	F	F	F
YHR118C	F	F	F	F	F	F	F	F	F	F	F	F	F	F	F	F	F	T	F	F	F	T	F
YHR119W	T	F	F	F	F	F	F	F	F	F	F	F	F	F	F	F	F	F	F	F	F	T	F
YHR121W	F	F	F	F	F	F	F	F	F	F	F	F	F	F	F	F	F	T	F	F	F	T	F
YHR122W	F	F	F	F	F	F	F	F	F	F	F	F	F	F	F	F	F	T	F	F	F	F	F
YHR124W	F	F	F	F	F	F	F	F	F	F	F	F	F	F	F	F	F	F	F	F	F	F	F
YHR127W	F	F	F	F	F	F	F	F	F	F	F	F	F	F	F	F	F	F	F	F	F	T	F
YHR128W	F	F	F	F	F	F	F	F	F	F	F	F	F	F	F	F	F	F	F	F	F	F	F
YHR129C	T	F	F	F	F	F	F	F	F	F	F	F	F	F	F	F	F	F	F	F	F	F	F
YHR131C	F	F	F	F	F	F	F	F	F	F	F	F	F	F	F	F	F	T	F	F	F	F	F
YHR133C	F	F	F	F	F	F	F	F	T	F	F	F	F	F	F	F	F	F	F	F	F	F	F
YHR134W	F	F	F	F	F	F	F	F	F	F	F	F	F	F	F	F	F	F	F	F	F	F	F
YHR135C	F	F	F	F	F	F	F	F	F	F	F	F	F	F	F	F	F	T	F	F	F	T	F
YHR143W-A	F	F	F	F	F	F	F	F	F	F	F	F	F	F	F	F	T	T	F	F	F	F	F
YHR144C	F	F	F	F	F	F	F	F	F	F	F	F	F	F	F	F	F	T	F	F	F	F	F
YHR146W	F	F	F	F	T	F	F	F	F	F	F	F	F	F	F	F	F	F	F	F	F	F	F
YHR147C	F	F	F	F	T	F	F	F	F	F	F	F	F	F	F	F	F	F	F	F	F	F	F
YHR148W	F	F	F	F	F	F	F	F	F	F	F	F	F	F	F	F	F	F	F	F	F	F	F
YHR150W	F	F	F	F	F	F	F	F	F	F	F	F	F	F	F	F	F	F	F	F	F	F	F
YHR152W	F	F	F	F	F	F	F	F	F	F	F	F	F	F	F	F	F	F	F	F	F	T	F
YHR154W	F	F	F	F	F	F	F	F	F	F	F	F	F	F	F	F	F	F	F	F	F	T	F
YHR156C	F	F	F	F	F	F	F	F	F	F	F	F	F	F	F	F	F	F	F	F	F	T	F
YHR157W	F	F	F	F	F	F	F	F	F	F	F	F	F	F	F	F	F	F	F	F	F	F	F
YHR158C	T	F	F	F	T	F	F	F	F	F	T	F	F	F	F	F	F	F	F	F	F	F	T
YHR160C	F	F	F	F	F	F	F	F	F	F	F	F	F	F	F	F	F	F	F	F	F	F	F
YHR161C	T	F	F	F	T	F	F	F	F	F	T	F	F	F	F	F	F	F	F	F	F	F	T
YHR164C	F	F	F	F	F	F	F	F	F	F	F	F	F	F	F	F	F	F	F	F	F	T	F
YHR165C	F	F	F	F	F	F	F	F	F	F	F	F	F	F	F	F	F	T	F	F	F	T	F
YHR166C	F	F	F	F	F	F	F	F	F	F	F	F	F	F	F	F	F	F	F	F	F	T	F
YHR167W	F	F	F	F	F	F	F	F	F	F	F	F	F	F	F	F	F	T	F	F	F	T	F
YHR168W	F	F	F	F	F	F	F	F	F	F	F	F	F	F	F	F	F	F	F	F	F	F	F
YHR169W	F	F	F	F	F	F	F	F	F	F	F	F	F	F	F	F	T	F	F	F	F	T	F
YHR170W	F	F	F	F	F	F	F	F	F	F	F	F	F	F	F	F	F	T	F	F	F	F	F
YHR171W	F	F	F	F	F	F	F	F	F	F	F	F	F	F	F	F	F	F	F	F	F	F	F
YHR172W	F	F	F	T	F	F	F	F	F	F	F	F	F	F	F	F	F	F	F	F	F	F	F
YHR174W	F	F	F	F	F	F	F	F	F	F	F	F	F	F	F	F	F	T	F	F	F	F	F
YHR178W	F	F	F	F	F	F	F	F	F	F	F	F	F	F	F	F	F	T	F	F	F	T	F
YHR179W	F	F	F	F	F	F	F	F	F	F	F	F	F	F	F	F	F	T	F	F	F	T	F
YHR181W	F	F	F	F	F	F	F	T	F	F	F	F	F	F	F	F	F	F	F	F	F	F	F
YHR183W	T	F	F	F	F	F	F	F	F	F	F	F	F	F	F	F	F	T	F	F	F	F	F
YHR184W	F	F	F	F	F	F	F	F	F	F	F	F	F	F	F	F	F	F	F	F	F	F	F
YHR185C	F	F	F	F	F	F	F	F	F	F	F	F	F	F	F	F	F	F	F	F	F	F	F
YHR186C	F	F	F	F	F	F	T	F	F	F	F	F	F	F	F	F	F	F	F	F	F	F	F
YHR187W	F	F	F	F	F	F	F	F	F	F	F	F	F	F	F	F	F	T	F	F	F	F	F
YHR190W	F	F	F	F	F	F	F	T	F	F	F	F	F	F	F	F	F	F	F	F	F	F	F
YHR191C	F	F	F	F	F	F	F	F	F	F	F	F	F	F	F	F	F	F	F	F	F	F	F
YHR193C	F	F	F	F	F	F	F	F	F	F	F	F	F	F	F	F	F	T	F	F	F	F	F
YHR195W	F	F	F	T	F	F	F	F	F	F	F	F	F	F	F	F	F	F	F	F	F	T	F

YHR196W	F	F	F	F	F	F	F	F	F	F	F	F	F	F	F	F	T	F	F	F	F	F	F
YHR197W	F	F	F	F	F	F	F	F	F	F	F	F	F	F	F	F	F	F	F	F	F	T	F
YHR200W	F	F	F	F	F	F	F	F	F	F	F	F	F	F	F	F	F	T	F	F	F	T	F
YHR203C	F	F	F	F	F	F	F	F	F	F	F	F	F	F	F	F	F	T	F	F	F	T	F
YHR205W	F	F	F	F	F	F	F	F	F	F	F	F	F	F	F	F	F	T	F	F	F	F	F
YHR206W	F	F	F	F	F	F	F	F	F	F	F	F	F	F	F	F	F	F	F	F	F	T	F
YHR208W	F	F	F	F	F	F	F	F	F	F	F	F	F	F	F	F	F	F	F	F	F	F	F
YHR216W	F	F	F	F	F	F	F	F	F	F	F	F	F	F	F	F	F	T	F	F	F	F	F
YIL002C	F	F	F	F	F	F	F	F	F	F	F	F	F	F	F	F	F	T	F	F	F	F	F
YIL004C	T	F	F	F	F	F	F	F	F	F	F	F	F	F	F	F	F	F	F	F	F	F	F
YIL005W	F	F	T	F	F	F	F	F	F	F	F	F	F	F	F	F	F	F	F	F	F	F	F
YIL007C	F	F	F	F	F	F	F	F	F	F	F	F	F	F	F	F	F	T	F	F	F	F	F
YIL008W	F	F	F	F	F	F	F	F	F	F	F	F	F	F	F	F	F	T	F	F	F	T	F
YIL017C	F	F	F	F	F	F	F	F	F	F	F	F	F	F	F	F	F	T	F	F	F	T	F
YIL018W	F	F	F	F	F	F	F	F	F	F	F	F	F	F	F	F	F	F	F	F	F	F	F
YIL019W	F	F	F	F	F	F	F	F	F	F	F	F	F	F	F	F	F	T	F	F	F	T	F
YIL021W	F	F	F	F	F	F	F	F	F	F	F	F	F	F	F	F	F	F	F	F	F	T	F
YIL022W	F	T	F	F	F	F	F	F	F	F	F	F	F	F	F	F	F	F	F	F	F	F	F
YIL026C	F	F	F	F	F	F	F	F	F	F	F	F	F	F	F	F	F	F	F	F	F	T	F
YIL030C	F	F	F	F	F	F	F	T	F	F	F	F	F	F	F	F	F	F	F	F	F	F	F
YIL033C	F	F	F	F	F	F	F	F	F	F	F	F	F	F	F	F	F	T	F	F	F	T	F
YIL034C	F	F	F	F	F	F	F	F	F	F	F	F	F	F	F	T	F	F	F	F	F	F	F
YIL035C	F	F	F	F	F	F	F	F	F	F	F	F	F	F	F	F	F	T	F	F	F	T	F
YIL036W	F	F	F	F	F	F	F	F	F	F	F	F	F	F	F	F	F	F	F	F	F	T	F
YIL038C	F	F	F	F	F	F	F	F	F	F	F	F	F	F	F	F	F	T	F	F	F	F	F
YIL041W	F	F	F	F	F	F	F	F	F	F	F	F	F	F	F	F	F	T	F	F	F	F	F
YIL042C	F	T	F	F	F	F	F	F	F	F	F	F	F	F	F	F	F	F	F	F	F	F	F
YIL043C	F	F	F	F	F	F	F	T	F	F	F	F	F	F	F	F	F	F	F	F	F	F	F
YIL045W	F	F	F	F	F	T	F	F	F	F	F	F	F	F	F	F	F	T	F	F	F	F	F
YIL046W	F	F	F	F	F	F	F	F	F	F	F	F	F	F	F	F	F	T	F	F	F	T	F
YIL050W	F	F	F	F	F	F	F	F	F	F	F	F	F	F	F	F	F	T	F	F	F	F	F
YIL053W	T	F	F	F	F	F	F	F	F	F	F	F	F	F	F	F	F	T	F	F	F	T	F
YIL061C	F	F	F	F	F	F	F	F	F	F	F	F	F	F	F	F	F	T	F	F	F	T	F
YIL062C	F	F	F	F	F	F	F	F	F	F	F	F	F	F	F	T	F	F	F	F	F	F	F
YIL063C	F	F	F	F	F	F	F	F	F	F	F	F	F	F	F	F	F	F	F	F	F	T	F
YIL065C	T	F	F	F	F	F	F	F	F	F	F	F	F	F	F	F	F	F	F	F	F	F	F
YIL066C	F	F	F	F	F	F	F	F	F	F	F	F	F	F	F	F	F	T	F	F	F	F	F
YIL068C	T	F	F	F	T	F	F	F	F	F	T	F	F	F	F	F	F	F	F	F	F	F	T
YIL069C	F	F	F	F	F	F	F	F	F	F	F	F	F	F	F	F	F	T	F	F	F	F	F
YIL070C	F	T	F	F	F	F	F	F	F	F	F	F	F	F	F	F	F	F	F	F	F	F	F
YIL071C	F	F	F	F	F	F	F	F	F	F	F	F	F	F	F	F	F	T	F	F	F	F	F
YIL072W	F	F	F	F	F	F	F	F	F	F	F	F	F	F	F	F	F	F	F	F	F	F	F
YIL074C	F	F	F	F	F	F	F	F	F	F	F	F	F	F	F	F	F	T	F	F	F	F	F
YIL075C	F	F	F	F	F	F	F	F	F	F	F	F	F	F	F	F	F	T	F	F	F	T	F
YIL076W	F	F	F	F	F	F	F	F	F	F	F	F	T	F	F	F	F	F	F	T	F	F	F
YIL078W	F	F	F	F	F	F	F	F	F	F	F	F	F	F	F	F	F	T	F	F	F	F	F
YIL079C	F	F	F	F	F	F	F	F	F	F	F	F	F	F	F	F	F	T	F	F	F	T	F
YIL084C	F	F	F	F	F	F	F	F	F	F	F	F	F	F	F	F	F	F	F	F	F	T	F
YIL091C	F	F	F	F	F	F	F	F	F	F	F	F	F	F	F	F	T	F	F	F	F	T	F
YIL092W	F	F	F	F	F	F	F	F	F	F	F	F	F	F	F	F	F	T	F	F	F	T	F
YIL093C	F	T	F	F	F	F	F	F	F	F	F	F	F	F	F	F	F	F	F	F	F	F	F
YIL094C	F	T	F	F	F	F	F	F	F	F	F	F	F	F	F	F	F	F	F	F	F	F	F
YIL095W	F	F	F	F	F	F	F	F	F	F	F	F	F	F	F	T	F	F	F	F	F	F	F
YIL097W	F	F	F	F	F	F	F	F	F	F	F	F	F	F	F	F	F	T	F	F	F	T	F
YIL098C	F	T	F	F	F	F	F	F	F	F	F	F	F	F	F	F	F	F	F	F	F	F	F
YIL103W	F	F	F	F	F	F	F	F	F	F	F	F	F	F	F	F	F	T	F	F	F	F	F
YIL104C	F	F	F	F	F	F	F	F	F	F	F	F	F	F	F	F	F	F	F	F	F	T	F
YIL105C	F	F	F	F	F	T	F	F	F	F	F	F	F	F	F	F	F	F	F	F	F	F	F
YIL106W	F	F	F	T	F	F	F	F	F	F	F	F	F	F	F	F	F	T	F	F	F	F	F
YIL108W	F	F	F	F	F	F	F	F	F	F	F	F	F	F	F	F	F	T	F	F	F	F	F
YIL109C	F	F	F	F	F	F	F	F	F	F	F	F	F	F	F	F	F	F	T	F	F	F	F
YIL112W	F	F	F	F	F	F	F	F	F	F	F	F	F	F	F	F	F	F	F	F	F	T	F
YIL113W	F	T	F	F	F	F	F	F	F	F	F	F	F	F	F	F	F	F	F	F	F	F	F
YIL115C	F	F	F	F	F	F	F	F	T	F	F	F	F	F	F	F	F	F	F	F	F	F	F
YIL118W	F	F	F	F	F	F	F	F	F	F	F	F	F	F	F	F	F	F	F	F	F	F	F
YIL122W	F	F	F	F	F	F	F	F	F	F	F	F	F	F	F	F	F	F	F	F	F	T	F
YIL124W	F	F	F	F	F	F	F	T	F	F	F	F	F	F	F	F	F	F	F	F	F	F	F
YIL125W	F	T	F	F	F	F	F	F	F	F	F	F	F	F	F	F	F	F	F	F	F	F	F

YIL126W	F	F	F	F	F	F	F	F	F	F	F	F	F	F	F	F	F	F	F	F	F	T	F
YIL128W	F	F	F	F	F	F	F	F	F	F	F	F	F	F	F	F	F	T	F	F	F	F	F
YIL129C	T	F	F	F	F	F	F	F	F	F	F	F	F	F	F	F	F	F	F	F	F	F	F
YIL133C	F	F	F	F	F	F	F	F	F	F	F	F	F	F	F	F	F	T	F	F	F	F	F
YIL135C	F	F	F	F	F	F	F	F	F	F	F	F	F	F	F	F	F	T	F	F	F	F	F
YIL138C	F	F	F	F	F	F	F	F	F	F	F	F	F	F	F	F	F	T	F	F	F	F	F
YIL139C	F	F	F	F	F	F	F	F	F	F	F	F	F	F	F	F	F	F	F	F	F	F	F
YIL140W	T	F	T	F	T	F	F	F	F	F	T	F	F	F	F	F	F	F	F	F	F	F	T
YIL142W	F	F	F	F	F	F	F	F	F	F	F	F	F	F	F	F	F	F	F	F	F	F	F
YIL143C	F	F	F	F	F	F	F	F	F	F	F	F	F	F	F	F	F	F	F	F	F	T	F
YIL144W	F	F	F	T	F	F	F	F	F	F	F	F	F	F	F	F	F	F	F	F	F	F	F
YIL146C	F	F	F	F	F	F	F	F	F	F	F	F	F	F	F	F	F	F	F	F	F	F	F
YIL147C	F	F	F	F	T	F	F	F	F	F	F	F	F	F	F	F	F	F	F	F	F	F	F
YIL149C	F	F	F	F	F	F	F	F	T	F	F	F	F	F	F	F	F	F	F	F	F	F	F
YIL150C	F	F	F	F	F	F	F	F	F	F	F	F	F	F	F	F	F	T	F	F	F	T	F
YIL153W	F	F	F	F	F	F	F	F	F	F	F	F	F	F	F	F	F	T	F	F	F	T	F
YIL156W	F	F	F	F	F	F	F	F	F	F	F	F	F	F	F	F	F	T	F	F	F	F	F
YIL157C	F	T	F	F	F	F	F	F	F	F	F	F	F	F	F	F	F	F	F	F	F	F	F
YIL159W	F	F	F	F	F	F	F	F	F	F	T	F	F	F	F	F	F	F	F	F	F	F	F
YIL160C	T	F	F	F	F	F	F	F	F	F	F	F	F	F	F	F	F	F	F	F	F	F	F
YIL161W	F	F	F	F	F	F	F	F	F	F	F	F	F	F	F	F	F	T	F	F	F	F	F
YIL173W	F	F	F	F	F	F	F	F	F	T	F	F	F	F	F	F	F	F	F	F	F	F	F
YIR001C	F	F	F	F	F	F	F	F	F	F	F	F	F	F	F	F	F	T	F	F	F	F	F
YIR002C	F	F	F	F	F	F	F	F	F	F	F	F	F	F	F	F	F	T	F	F	F	T	F
YIR003W	F	F	F	F	F	F	F	F	F	F	F	F	F	F	F	T	F	F	F	F	F	F	F
YIR004W	F	F	F	F	F	F	F	T	F	F	F	F	F	F	F	F	F	F	F	F	F	F	F
YIR005W	F	F	F	F	F	F	F	F	F	F	F	F	F	F	F	F	F	T	F	F	F	T	F
YIR006C	F	F	F	F	F	T	F	F	F	F	F	F	F	F	F	F	F	F	F	F	F	F	F
YIR008C	F	F	F	F	F	F	F	F	F	F	F	F	F	F	F	F	F	F	F	F	F	F	F
YIR009W	F	F	F	F	F	F	F	F	F	F	F	F	F	F	F	F	F	T	F	F	F	T	F
YIR010W	F	F	F	T	F	F	F	F	F	F	F	F	F	F	F	F	F	F	F	F	F	F	F
YIR011C	F	F	F	F	F	F	F	F	F	F	F	F	F	F	F	F	F	T	F	F	F	T	F
YIR012W	F	F	F	F	F	F	F	F	F	F	F	F	F	F	F	F	F	T	F	F	F	T	F
YIR015W	F	F	F	F	F	F	F	F	F	F	F	F	F	F	F	F	F	F	F	F	F	T	F
YIR017C	F	F	F	F	F	F	F	F	F	F	F	F	F	F	F	F	F	T	F	F	F	T	F
YIR022W	F	F	F	F	F	F	F	T	F	F	F	F	F	F	F	F	F	F	F	F	F	F	F
YIR025W	F	F	F	F	F	F	F	F	F	F	F	F	F	F	F	F	F	F	F	F	F	F	F
YIR026C	F	F	F	F	F	F	F	F	F	F	F	F	F	F	F	F	F	T	F	F	F	F	F
YIR033W	F	F	F	F	F	F	F	F	F	F	F	F	F	F	F	F	F	F	F	F	F	F	F
YIR034C	F	F	F	F	F	F	F	F	F	F	F	F	F	F	F	F	F	T	F	F	F	T	F
YIR035C	F	F	F	F	F	F	F	F	F	F	F	F	F	F	F	F	F	T	F	F	F	F	F
YIR037W	F	F	F	F	F	F	F	F	F	F	F	F	F	F	F	F	F	T	F	F	F	F	F
YIR039C	F	F	F	F	F	F	F	F	F	F	F	F	F	F	F	F	F	F	F	F	F	F	F
YJL001W	F	F	F	F	F	F	F	F	F	F	F	F	F	F	F	F	F	F	F	F	F	T	F
YJL002C	F	F	F	F	F	F	F	T	F	F	F	F	F	F	F	F	F	F	F	F	F	F	F
YJL005W	T	F	F	F	F	F	F	F	F	F	F	F	F	F	F	F	F	F	F	F	F	F	F
YJL006C	F	F	F	F	F	F	F	F	F	F	F	F	F	F	F	F	F	F	F	F	F	T	F
YJL008C	F	F	F	F	F	F	F	F	F	F	F	F	F	F	F	F	F	F	F	F	F	F	F
YJL010C	F	F	F	F	F	F	F	F	F	F	F	F	F	F	F	F	T	F	F	F	F	T	F
YJL011C	F	F	F	F	F	F	F	F	F	F	F	F	F	F	F	F	F	F	F	F	F	T	F
YJL012C	F	F	F	F	F	F	F	F	F	F	F	F	F	F	F	F	F	F	F	F	F	F	F
YJL013C	F	F	F	F	F	F	F	F	F	F	F	F	F	F	F	F	F	T	F	F	F	T	F
YJL014W	F	F	F	F	F	F	F	F	F	F	F	F	F	F	F	F	F	F	F	F	F	F	F
YJL019W	F	F	F	F	F	F	F	F	F	F	F	F	F	F	F	F	F	F	F	F	F	F	F
YJL020C	F	F	F	F	F	F	F	F	F	F	F	F	F	F	F	F	F	F	F	F	F	F	F
YJL023C	F	F	F	F	F	F	F	F	F	F	F	F	F	F	F	F	F	F	F	F	F	F	F
YJL024C	F	F	F	F	F	T	F	F	F	F	F	F	F	F	F	F	F	F	F	T	F	F	F
YJL025W	F	F	F	F	F	F	F	F	F	F	F	F	F	F	F	F	T	F	F	F	F	F	F
YJL026W	F	F	F	F	F	F	F	F	F	F	F	F	F	F	F	F	F	T	F	F	F	T	F
YJL029C	F	F	F	F	F	T	F	F	F	F	F	F	F	F	F	F	F	F	F	T	F	F	F
YJL030W	F	F	F	F	F	F	F	F	F	F	F	F	F	F	F	F	F	T	F	F	F	T	F
YJL031C	F	F	F	F	F	F	F	F	F	F	F	F	F	F	F	F	F	F	F	F	F	F	F
YJL033W	F	F	F	F	F	F	F	F	F	F	F	F	F	F	F	F	T	F	F	F	F	F	F
YJL034W	T	F	F	F	F	F	F	F	F	F	F	F	F	F	F	F	F	F	F	F	F	F	F
YJL036W	F	F	F	F	F	F	F	F	F	T	F	F	F	F	F	F	F	F	F	F	F	F	F
YJL039C	F	F	F	F	F	F	F	F	T	F	F	F	F	F	F	F	F	F	F	F	F	F	F
YJL041W	F	F	F	F	F	F	F	F	T	F	F	F	F	F	F	F	F	F	F	F	F	F	F
YJL042W	F	F	F	F	F	F	F	F	F	F	F	F	F	F	F	F	F	T	F	F	F	F	F

YJL044C	F	F	F	F	F	F	F	F	F	F	F	F	F	T	F	F	F	F	F	F	F	F	F
YJL047C	F	F	F	F	F	F	F	F	F	F	F	F	F	F	F	F	F	T	F	F	F	T	F
YJL048C	F	F	F	F	F	F	F	F	T	F	F	F	F	F	F	F	F	F	F	F	F	F	F
YJL050W	F	F	F	F	F	F	F	F	F	F	F	F	F	F	F	F	T	F	F	F	F	T	F
YJL052W	F	F	F	F	F	F	F	F	F	F	F	F	F	F	F	F	F	T	F	F	F	T	F
YJL053W	F	F	F	F	F	F	F	F	F	T	F	F	F	F	F	F	F	F	F	F	F	F	F
YJL054W	F	T	F	F	F	F	F	F	F	F	F	F	F	F	F	F	F	F	F	F	F	F	F
YJL058C	F	F	F	F	F	F	F	F	F	F	F	F	F	F	F	F	F	T	F	F	F	F	F
YJL061W	F	F	F	F	F	F	F	F	T	F	F	F	F	F	F	F	F	F	F	F	F	F	F
YJL062W-A	F	T	F	F	F	F	F	F	F	F	F	F	F	F	F	F	F	F	F	F	F	F	F
YJL063C	F	T	F	F	F	F	F	F	F	F	F	F	F	F	F	F	F	F	F	F	F	F	F
YJL065C	F	F	F	F	F	F	F	F	F	F	F	F	F	F	F	F	F	F	F	F	F	T	F
YJL069C	F	F	F	F	F	F	F	F	F	F	F	F	F	F	F	F	T	F	F	F	F	F	F
YJL070C	F	F	F	F	F	F	F	F	F	F	F	F	F	F	F	F	F	T	F	F	F	F	F
YJL072C	F	F	F	F	F	F	F	F	F	F	F	F	F	F	F	F	F	F	F	F	F	F	F
YJL073W	F	F	F	F	F	F	F	F	F	F	F	F	F	F	F	F	F	F	F	F	F	F	F
YJL074C	F	F	F	F	F	F	F	F	F	F	F	F	F	F	F	F	F	F	F	F	F	T	F
YJL076W	F	F	F	F	F	F	F	F	F	F	F	F	F	F	F	F	T	F	F	F	F	F	F
YJL080C	F	F	F	F	F	F	F	T	F	F	F	F	F	F	F	F	F	F	F	F	F	F	F
YJL081C	F	F	F	F	F	F	F	F	F	F	F	F	F	F	F	F	F	F	F	F	F	T	F
YJL082W	F	F	F	F	F	F	F	F	F	F	F	F	F	F	F	F	F	T	F	F	F	T	F
YJL084C	F	F	F	F	F	F	F	F	F	F	F	F	F	F	F	F	F	T	F	F	F	F	F
YJL085W	F	F	F	F	T	F	F	F	F	F	T	F	F	F	F	F	F	F	F	F	F	F	T
YJL088W	F	F	F	F	F	F	F	F	F	F	F	F	F	F	F	F	F	T	F	F	F	T	F
YJL089W	F	F	F	F	F	F	F	F	F	F	F	F	F	F	F	F	F	F	F	F	F	F	F
YJL090C	F	F	F	F	F	F	F	F	F	F	F	F	F	F	F	F	F	T	F	F	F	T	F
YJL092W	F	F	F	F	F	F	F	F	F	F	F	F	F	F	F	F	F	F	F	F	F	T	F
YJL093C	F	F	F	F	F	F	F	F	F	F	F	F	F	F	F	F	F	F	F	F	F	F	F
YJL095W	F	F	F	F	F	F	F	F	F	F	F	F	F	F	F	F	F	T	F	F	F	F	F
YJL098W	F	F	F	F	F	F	F	F	F	F	F	F	F	F	F	F	F	T	F	F	F	F	F
YJL099W	F	F	F	F	F	F	F	F	F	F	F	F	F	T	F	F	F	F	F	F	F	F	F
YJL100W	F	F	F	F	F	F	F	F	F	F	F	F	F	F	F	F	F	F	F	F	F	F	F
YJL101C	F	F	F	F	F	F	F	F	F	F	F	F	F	F	F	F	F	T	F	F	F	F	F
YJL104W	F	T	F	F	F	F	F	F	F	F	F	F	F	F	F	F	F	F	F	F	F	F	F
YJL106W	F	F	F	F	F	F	F	F	F	F	F	F	F	F	F	F	F	F	F	F	F	F	F
YJL108C	F	F	F	F	F	F	F	F	F	F	F	F	F	F	F	F	F	F	F	F	F	F	F
YJL109C	F	F	F	F	F	F	F	F	F	F	F	F	F	F	F	F	T	F	F	F	F	F	F
YJL110C	F	F	F	F	F	F	F	F	F	F	F	F	F	F	F	F	F	T	F	F	F	T	F
YJL111W	F	F	F	F	F	F	F	F	F	F	F	F	F	F	F	F	F	F	F	F	F	F	F
YJL112W	F	T	F	F	F	F	F	F	F	F	F	F	F	F	F	F	F	F	F	F	F	F	F
YJL115W	F	F	F	F	F	F	F	F	F	F	F	F	F	F	F	F	F	F	F	F	F	T	F
YJL117W	F	F	F	F	F	F	F	T	F	F	F	F	F	F	F	F	F	F	F	F	F	F	F
YJL122W	F	F	F	F	F	F	F	F	F	F	F	F	F	F	F	F	F	F	F	F	F	T	F
YJL124C	F	F	F	F	F	F	F	F	F	F	F	F	F	F	F	F	F	T	F	F	F	F	F
YJL125C	F	F	F	F	F	F	F	F	F	F	F	F	F	F	F	F	F	F	F	F	F	T	F
YJL128C	F	F	F	F	F	F	F	F	F	F	F	F	F	F	F	F	F	T	F	F	F	F	F
YJL137C	F	F	F	F	F	F	F	F	F	F	F	F	F	F	F	F	F	T	F	F	F	F	F
YJL138C	F	F	F	F	F	F	F	F	F	F	F	F	F	F	F	F	F	T	F	F	F	F	F
YJL140W	F	F	F	F	F	F	F	F	F	F	F	F	F	F	F	F	F	F	F	F	F	T	F
YJL141C	F	F	F	F	F	F	F	F	F	F	F	F	F	F	F	F	F	F	F	F	F	F	F
YJL143W	F	F	F	F	F	F	F	F	F	F	F	F	F	F	F	F	F	T	F	F	F	T	F
YJL146W	F	F	F	F	F	F	F	F	F	F	F	F	F	F	F	F	F	T	F	F	F	T	F
YJL148W	F	F	F	F	F	F	F	F	F	F	F	F	F	F	F	F	T	F	F	F	F	F	F
YJL149W	F	F	F	F	F	F	F	F	F	F	F	F	F	F	F	F	F	F	F	F	F	F	F
YJL151C	F	F	T	F	F	F	F	F	F	F	F	F	F	F	F	F	F	F	F	F	F	F	F
YJL153C	F	F	F	F	F	F	F	F	F	F	F	F	F	F	F	F	F	T	F	F	F	F	F
YJL154C	F	F	F	F	F	F	T	F	F	T	F	F	F	F	F	F	F	F	F	F	F	F	F
YJL155C	F	F	F	F	F	F	F	F	F	F	F	F	F	F	F	F	F	T	F	F	F	F	F
YJL157C	F	F	F	F	F	F	F	F	F	F	F	F	F	F	F	F	F	F	F	F	F	T	F
YJL164C	F	F	F	F	F	F	F	F	F	F	F	F	F	F	F	F	F	T	F	F	F	T	F
YJL167W	F	F	F	F	F	F	F	F	F	F	F	F	F	F	F	F	F	F	F	F	F	F	F
YJL168C	F	F	F	F	F	F	F	F	F	F	F	F	F	F	F	F	F	F	F	F	F	T	F
YJL172W	F	F	F	F	F	F	F	F	F	F	F	F	F	F	F	F	F	F	F	F	F	F	F
YJL173C	F	F	F	F	F	F	F	F	F	F	F	F	F	F	F	F	F	F	F	F	F	T	F
YJL176C	F	F	F	F	F	F	F	F	F	F	F	F	F	F	F	F	F	F	F	F	F	T	F
YJL178C	F	F	F	F	F	T	F	F	F	F	F	F	F	F	F	F	F	F	F	F	F	F	F
YJL179W	F	F	F	F	F	F	F	F	F	F	F	F	F	F	F	F	F	T	F	F	F	F	F
YJL180C	F	F	F	F	F	F	F	F	F	F	F	F	F	F	F	F	F	T	F	F	F	F	F

YJL183W	F	F	F	F	F	F	F	F	F	F	F	F	T	F	F	F	F	F	F	F	F	F	F
YJL184W	F	F	F	F	F	F	F	F	F	F	F	F	F	F	F	F	F	F	F	F	F	F	F
YJL187C	F	F	F	F	F	F	F	F	F	F	F	F	F	F	F	F	F	F	F	F	F	F	F
YJL190C	F	F	F	F	F	F	F	F	F	F	F	F	F	F	F	F	T	F	F	F	F	T	F
YJL191W	F	F	F	F	F	F	F	F	F	F	F	F	F	F	F	F	T	T	F	F	F	T	F
YJL194W	F	F	F	F	F	F	F	F	F	F	F	F	F	F	F	F	F	T	F	F	F	T	F
YJL201W	F	F	F	F	F	F	F	F	F	F	F	F	F	F	F	F	F	T	F	F	F	F	F
YJL203W	F	F	F	F	F	F	F	F	F	F	F	F	F	F	F	F	F	F	F	F	F	T	F
YJL204C	F	F	F	F	F	F	F	F	F	F	F	F	F	F	F	F	F	T	F	F	F	F	F
YJL207C	F	F	F	F	F	F	F	F	F	F	F	F	F	T	F	F	F	F	F	F	F	F	F
YJL208C	F	T	F	F	F	F	F	F	F	F	F	F	F	F	F	F	F	F	F	F	F	F	F
YJL210W	F	F	F	F	F	F	F	F	F	F	F	F	F	F	T	F	F	F	F	F	F	F	F
YJL217W	F	F	F	F	F	F	F	F	F	F	F	F	F	F	F	F	F	T	F	F	F	F	F
YJR002W	F	F	F	F	F	F	F	F	F	F	F	F	F	F	F	F	F	F	F	F	F	F	F
YJR005W	F	F	F	F	T	F	F	F	F	F	T	F	F	F	F	F	F	F	F	F	F	F	F
YJR006W	F	F	F	F	F	F	F	F	F	F	F	F	F	F	F	F	F	F	F	F	F	T	F
YJR007W	F	F	F	F	F	F	F	F	F	F	F	F	F	F	F	F	F	T	F	F	F	F	F
YJR009C	F	F	F	F	F	F	F	F	F	F	F	F	F	F	F	F	F	T	F	F	F	T	F
YJR010C-A	F	F	F	F	F	F	F	T	F	F	F	F	F	F	F	F	F	F	F	F	F	F	F
YJR010W	F	F	F	F	F	F	F	F	F	F	F	F	F	F	F	F	F	T	F	F	F	F	F
YJR011C	T	F	F	F	F	F	F	F	F	F	F	F	F	F	F	F	F	F	F	F	F	F	F
YJR012C	F	F	F	F	F	F	F	F	F	F	F	F	F	F	F	F	F	F	F	F	F	F	F
YJR014W	F	F	F	F	F	F	F	F	F	F	F	F	F	F	F	F	F	T	F	F	F	F	F
YJR017C	F	F	F	F	F	F	F	F	F	F	F	F	F	F	F	F	F	T	F	F	F	T	F
YJR022W	F	F	F	F	F	F	F	F	F	F	F	F	F	F	F	F	F	T	F	F	F	T	F
YJR025C	F	F	F	F	F	F	F	F	F	F	F	F	F	F	F	F	F	T	F	F	F	T	F
YJR031C	F	F	F	F	F	F	F	F	F	F	F	F	T	F	F	F	F	F	F	F	F	F	F
YJR032W	F	F	F	F	F	F	F	F	F	F	F	F	F	F	F	F	F	T	F	F	F	F	F
YJR033C	T	F	F	F	F	F	F	F	F	F	F	F	F	F	F	F	F	F	F	F	F	F	F
YJR040W	F	F	F	F	F	F	F	F	F	F	F	F	F	F	F	F	F	F	F	F	F	F	F
YJR041C	F	F	F	F	F	F	F	F	F	F	F	F	F	F	F	F	T	F	F	F	F	F	F
YJR042W	F	F	F	F	F	F	F	F	T	F	F	F	F	F	F	F	F	F	F	F	F	F	F
YJR043C	F	F	F	F	F	F	F	F	F	F	F	F	F	F	F	F	F	T	F	F	F	T	F
YJR045C	F	F	F	F	F	F	F	F	F	F	F	F	F	F	F	F	F	F	F	F	F	F	F
YJR046W	F	F	F	F	F	F	F	F	F	F	F	F	F	F	F	F	F	T	F	F	F	T	F
YJR048W	F	F	F	F	F	F	F	F	F	F	F	F	F	F	F	F	F	T	F	F	F	T	F
YJR049C	F	F	F	F	F	F	F	F	F	F	F	F	F	F	F	F	F	T	F	F	F	T	F
YJR050W	F	F	F	F	F	F	F	F	F	F	F	F	F	F	F	F	F	T	F	F	F	T	F
YJR052W	F	F	F	F	F	F	F	F	F	F	F	F	F	F	F	F	F	T	F	F	F	T	F
YJR053W	F	F	F	T	F	F	F	F	F	F	F	F	F	F	F	F	F	F	F	F	F	F	F
YJR056C	F	F	F	F	F	F	F	F	F	F	F	F	F	F	F	F	F	T	F	F	F	T	F
YJR057W	F	F	F	F	F	F	F	F	F	F	F	F	F	F	F	F	F	T	F	F	F	F	F
YJR058C	F	F	F	F	T	F	F	F	F	F	T	F	F	F	F	F	F	F	F	F	F	F	F
YJR060W	F	F	F	F	F	F	F	F	F	F	F	F	F	F	F	F	F	F	F	F	F	T	F
YJR062C	F	T	F	F	F	F	F	F	F	F	F	F	F	F	F	F	F	F	F	F	F	F	F
YJR063W	F	F	F	F	F	F	F	F	F	F	F	F	F	F	F	F	T	F	F	F	F	F	F
YJR064W	F	F	F	F	F	F	F	F	F	F	F	F	F	F	F	F	F	F	F	F	F	F	F
YJR065C	F	F	F	F	F	F	F	F	F	F	F	F	F	F	F	F	F	F	F	F	F	F	F
YJR066W	F	F	F	F	F	F	F	F	F	F	F	F	F	F	F	F	F	F	F	F	F	F	F
YJR067C	F	F	F	F	F	F	F	F	F	F	F	F	F	F	F	F	F	T	F	F	F	T	F
YJR068W	F	F	F	F	F	F	F	F	F	F	F	F	F	F	F	F	F	F	F	F	F	T	F
YJR070C	F	F	F	F	F	F	F	F	F	F	F	F	F	F	F	F	F	T	F	F	F	T	F
YJR072C	F	F	F	F	F	F	F	F	F	F	F	F	F	F	F	F	F	T	F	F	F	F	F
YJR073C	F	F	T	F	F	F	F	T	F	F	F	F	F	F	F	F	F	F	F	F	F	F	F
YJR074W	F	F	F	F	F	F	F	F	F	F	F	F	F	F	F	F	F	T	F	F	F	F	F
YJR075W	F	F	F	F	F	F	F	F	F	F	F	F	T	F	F	F	F	F	F	F	F	F	F
YJR076C	F	F	F	F	T	F	F	F	F	F	T	F	F	F	F	F	F	F	F	F	F	F	F
YJR077C	F	T	F	F	F	F	F	F	F	F	F	F	F	F	F	F	F	F	F	F	F	F	F
YJR082C	F	F	F	F	F	F	F	F	F	F	F	F	F	F	F	F	F	F	F	F	F	T	F
YJR083C	T	F	F	F	F	F	F	F	F	F	F	F	F	F	F	F	F	F	F	F	F	F	F
YJR084W	F	F	F	F	F	F	F	F	F	F	F	F	F	F	F	F	F	T	F	F	F	T	F
YJR086W	F	F	F	F	F	T	F	F	F	F	F	F	F	F	F	F	F	T	F	F	F	F	F
YJR089W	F	F	F	T	F	F	F	F	F	F	F	T	F	F	F	F	F	F	F	F	F	T	F
YJR090C	F	F	F	F	F	F	F	F	F	F	F	F	F	F	F	F	F	F	F	F	F	F	F
YJR091C	T	F	F	F	F	F	F	F	F	F	F	F	F	F	F	F	F	F	F	F	F	F	F
YJR092W	F	F	F	F	F	F	F	F	F	F	T	F	F	F	F	F	F	F	F	F	F	F	F
YJR093C	F	F	F	F	F	F	F	F	F	F	F	F	F	F	F	F	F	F	F	F	F	F	F
YJR094C	F	F	F	F	F	F	F	F	F	F	F	F	F	F	F	F	F	F	F	F	F	F	F

YJR096W	F	F	F	F	F	F	F	F	F	F	F	F	F	F	F	F	F	T	F	F	F	T	F
YJR099W	F	F	F	F	F	F	F	F	F	F	F	F	F	F	F	F	F	F	F	F	F	F	F
YJR101W	F	T	F	F	F	F	F	F	F	F	F	F	F	F	F	F	F	F	F	F	F	F	F
YJR102C	F	F	F	F	F	F	F	F	F	F	F	F	F	F	F	F	F	F	F	F	F	F	F
YJR103W	F	F	F	F	F	F	F	F	F	F	F	F	F	F	F	F	F	T	F	F	F	F	F
YJR104C	T	F	F	F	F	F	F	F	F	F	F	F	F	F	F	F	F	T	F	F	F	T	F
YJR105W	F	F	F	F	F	F	F	F	F	F	F	F	F	F	F	F	F	T	F	F	F	T	F
YJR109C	F	F	F	F	F	F	F	F	F	F	F	F	F	F	F	F	F	T	F	F	F	F	F
YJR112W	F	F	F	T	F	F	F	F	F	F	F	F	F	F	F	F	F	F	F	F	F	F	F
YJR113C	F	T	F	F	F	F	F	F	F	F	F	F	F	F	F	F	F	F	F	F	F	F	F
YJR117W	F	F	F	F	F	F	F	T	F	F	F	F	F	F	F	F	F	F	F	F	F	F	F
YJR119C	F	F	F	F	F	F	F	F	F	F	F	F	F	F	F	F	F	T	F	F	F	T	F
YJR121W	F	T	F	F	F	F	F	F	F	F	F	F	F	F	F	F	F	F	F	F	F	F	F
YJR123W	F	F	F	F	F	F	F	F	F	F	F	F	F	F	F	F	F	F	F	F	F	F	F
YJR125C	F	F	F	F	F	F	F	F	F	F	F	F	F	F	F	F	F	F	F	F	F	F	F
YJR132W	F	F	F	F	F	F	F	F	F	F	F	F	F	F	F	F	F	T	F	F	F	T	F
YJR134C	F	F	F	F	F	F	F	F	F	F	F	F	T	F	F	F	F	F	F	T	F	F	F
YJR135C	F	F	F	T	F	F	F	F	F	F	F	F	F	F	F	F	F	F	F	F	F	T	F
YJR135W-A	F	F	F	F	F	F	F	F	F	F	F	F	F	F	F	F	F	F	F	F	F	F	F
YJR136C	F	F	F	F	F	F	F	F	F	F	F	F	F	F	F	F	F	T	F	F	F	F	F
YJR137C	F	F	F	F	F	F	F	F	F	F	F	F	F	F	F	F	F	T	F	F	F	F	F
YJR138W	F	F	F	F	F	F	T	F	F	F	F	F	F	F	F	F	F	F	F	F	F	F	F
YJR140C	F	F	F	F	F	F	F	F	F	F	F	F	F	F	F	F	F	F	F	F	F	T	F
YJR141W	F	F	F	F	F	F	F	F	F	F	F	F	F	F	F	F	F	F	F	F	F	F	F
YJR144W	F	F	F	F	F	F	F	F	F	F	F	F	F	F	F	F	F	F	F	F	F	F	F
YJR145C	F	F	F	F	F	F	F	F	F	F	F	F	F	F	F	F	F	T	F	F	F	F	F
YJR148W	F	F	F	F	F	F	F	F	F	F	F	F	F	F	F	F	F	T	F	F	F	T	F
YKL001C	F	F	F	F	F	F	F	F	F	F	F	F	F	F	F	F	F	T	F	F	F	F	F
YKL002W	F	F	F	F	F	F	F	F	F	T	F	F	F	F	F	F	F	F	F	F	F	F	F
YKL003C	F	T	F	F	F	F	F	F	F	F	F	F	F	F	F	F	F	F	F	F	F	F	F
YKL005C	F	F	F	F	F	F	F	F	F	F	F	F	F	F	F	F	F	F	F	F	F	F	F
YKL006C-A	F	F	F	F	F	F	F	F	F	F	F	F	F	F	F	F	F	T	F	F	F	T	F
YKL007W	F	F	F	F	F	F	F	F	F	F	F	F	F	F	F	T	F	F	F	F	F	F	F
YKL008C	F	F	F	F	F	F	F	T	F	F	F	F	F	F	F	F	F	F	F	F	F	F	F
YKL009W	F	F	F	F	F	F	F	F	F	F	F	F	F	F	F	F	T	F	F	F	F	T	F
YKL010C	F	F	F	F	F	F	F	F	F	F	F	F	F	F	F	F	F	T	F	F	F	T	F
YKL012W	F	F	F	F	F	F	F	F	F	F	F	F	F	F	F	F	F	F	F	F	F	T	F
YKL013C	F	F	F	F	F	F	F	F	F	F	F	F	F	F	F	T	F	F	F	F	F	F	F
YKL014C	F	F	F	F	F	F	F	F	F	F	F	F	F	F	F	F	T	F	F	F	F	F	F
YKL016C	F	T	F	F	F	F	F	F	F	F	F	F	F	F	F	F	F	F	F	F	F	F	F
YKL018W	F	F	F	F	F	F	F	F	F	F	F	F	F	F	F	F	F	F	F	F	F	F	F
YKL020C	F	F	F	F	F	F	F	F	F	F	F	F	F	F	F	F	F	T	F	F	F	F	F
YKL021C	F	F	F	F	F	F	F	F	F	F	F	F	F	F	F	F	T	F	F	F	F	T	F
YKL022C	F	F	F	F	F	F	F	F	F	F	F	F	F	F	F	F	F	F	F	F	F	T	F
YKL023W	F	F	F	F	F	F	F	F	F	F	F	F	F	F	F	F	F	T	F	F	F	F	F
YKL028W	F	F	F	F	F	F	F	F	F	F	F	F	F	F	F	F	F	F	F	F	F	T	F
YKL029C	F	T	F	F	F	F	F	F	F	F	F	F	F	F	F	F	F	F	F	F	F	F	F
YKL032C	F	F	F	F	F	F	F	F	F	F	F	F	F	F	F	F	F	F	F	F	F	T	F
YKL033W	F	F	F	F	F	F	F	F	F	F	F	F	F	F	F	F	F	T	F	F	F	F	F
YKL034W	F	F	F	F	F	F	F	F	F	F	F	F	F	F	F	F	F	F	F	F	F	F	F
YKL035W	F	F	F	F	F	F	F	F	F	F	F	F	F	F	F	F	F	T	F	F	F	F	F
YKL038W	F	F	F	F	F	F	F	F	F	F	F	F	F	F	F	F	F	T	F	F	F	T	F
YKL041W	F	F	F	F	F	T	F	F	F	T	F	F	F	F	F	F	F	F	F	F	F	F	F
YKL042W	F	F	F	T	F	F	F	F	F	F	F	F	F	F	F	F	F	F	F	F	F	F	F
YKL043W	F	F	F	F	F	F	F	F	F	F	F	F	F	F	F	F	F	F	F	F	F	T	F
YKL045W	F	F	F	F	F	F	F	F	F	F	F	F	F	F	F	F	F	F	F	F	F	F	F
YKL048C	F	F	F	F	F	F	F	F	F	F	T	F	F	F	F	F	F	F	F	F	F	F	F
YKL049C	F	F	F	T	F	F	F	F	F	F	F	F	F	F	F	F	F	F	F	F	F	T	F
YKL051W	F	F	F	F	T	F	F	F	F	F	F	F	F	F	F	F	F	F	F	F	F	F	F
YKL052C	F	F	F	T	F	F	F	F	F	F	F	F	F	F	F	F	F	F	F	F	F	F	F
YKL054C	F	F	F	F	F	F	F	F	F	F	F	F	F	F	F	F	F	T	F	F	F	F	F
YKL056C	F	F	F	F	F	F	F	F	F	F	F	F	F	F	F	F	F	T	F	F	F	F	F
YKL057C	F	F	F	F	F	F	F	F	F	F	F	F	F	F	F	F	F	F	F	F	F	F	F
YKL058W	F	F	F	F	F	F	F	F	F	F	F	F	F	F	F	F	F	F	F	F	F	T	F
YKL059C	F	F	F	F	F	F	F	F	F	F	F	F	F	F	F	F	F	F	F	F	F	T	F
YKL060C	F	F	F	F	F	F	F	F	F	F	F	F	F	F	F	F	F	T	F	F	F	F	F
YKL061W	F	F	F	F	F	F	F	F	F	T	F	F	F	F	F	F	F	F	F	F	F	F	F
YKL065C	F	F	F	F	F	F	F	T	F	F	F	F	F	F	F	F	F	F	F	F	F	F	F

YKL067W	F	F	F	F	F	F	F	F	F	F	F	F	F	F	F	F	F	T	F	F	F	T	F
YKL068W	F	F	F	F	F	F	F	F	T	F	F	F	F	F	F	F	F	F	F	F	F	F	F
YKL073W	F	F	T	F	F	F	F	F	F	F	F	F	F	F	F	F	F	F	F	F	F	F	F
YKL074C	F	F	F	F	F	F	F	F	F	F	F	F	F	F	F	F	F	T	F	F	F	T	F
YKL075C	F	F	F	F	F	F	F	F	F	F	F	F	F	F	F	F	F	T	F	F	F	F	F
YKL078W	F	F	F	F	F	F	F	F	F	F	F	F	F	F	F	F	T	F	F	F	F	F	F
YKL079W	T	F	F	F	F	F	F	F	F	F	F	F	F	F	F	F	F	T	F	F	F	F	T
YKL080W	T	F	F	F	F	F	T	F	F	F	F	F	F	F	F	F	F	F	F	F	F	F	F
YKL081W	F	F	F	F	F	F	F	F	F	F	F	F	F	F	F	F	F	T	F	F	F	F	F
YKL082C	F	F	F	F	F	F	F	F	F	F	F	F	F	F	F	F	F	F	F	F	F	F	F
YKL084W	F	F	F	F	F	F	F	F	F	F	F	F	F	F	F	F	F	F	F	F	F	F	F
YKL088W	F	F	F	F	F	F	F	F	F	F	F	F	F	F	F	F	F	T	F	F	F	F	F
YKL089W	F	F	F	T	F	F	F	F	F	F	F	F	F	F	F	F	F	F	F	F	F	F	F
YKL090W	F	F	F	F	F	F	F	F	F	F	F	F	F	F	F	F	F	F	F	F	F	F	F
YKL094W	F	F	F	F	F	F	F	T	F	F	F	F	F	F	F	F	F	F	F	F	F	F	F
YKL095W	F	F	F	F	F	F	F	F	F	F	F	F	F	F	F	F	F	F	F	F	F	T	F
YKL099C	F	F	F	F	F	F	F	F	F	F	F	F	F	F	F	F	F	F	F	F	F	F	F
YKL101W	F	F	F	F	F	F	F	F	F	F	T	F	F	F	F	F	F	F	F	F	F	F	F
YKL103C	F	F	F	F	F	T	F	F	F	F	F	F	F	F	F	F	F	T	F	F	F	F	F
YKL104C	F	F	F	F	F	F	F	F	F	F	F	F	F	F	F	F	F	F	F	F	F	F	F
YKL105C	T	F	F	F	F	F	F	F	F	F	F	F	F	F	F	F	F	F	F	F	F	F	F
YKL108W	F	F	F	F	F	F	F	F	F	F	F	F	F	F	F	F	F	F	F	F	F	T	F
YKL109W	F	F	F	F	F	F	F	F	F	F	F	F	F	F	F	F	F	F	F	F	F	F	F
YKL110C	F	F	F	F	F	F	F	F	F	F	F	F	F	F	F	F	F	T	F	F	F	F	F
YKL112W	F	F	F	F	F	F	F	F	F	F	F	F	F	F	F	F	F	F	F	F	F	T	F
YKL113C	F	F	F	F	F	F	F	F	F	F	F	F	F	F	F	F	F	F	F	F	F	T	F
YKL116C	F	F	F	F	F	F	F	F	F	F	F	F	F	F	F	F	F	T	F	F	F	F	F
YKL117W	F	F	F	F	F	F	F	F	F	F	F	F	F	F	F	F	F	T	F	F	F	T	F
YKL119C	F	F	F	F	F	F	F	T	F	F	F	F	F	F	F	F	F	F	F	F	F	F	F
YKL120W	F	T	F	F	F	F	F	F	F	F	F	F	F	F	F	F	F	F	F	F	F	F	F
YKL122C	F	F	F	F	F	F	F	T	F	F	F	F	F	F	F	F	F	F	F	F	F	F	F
YKL125W	F	F	F	F	F	F	F	F	F	F	F	F	F	F	F	F	T	F	F	F	F	T	F
YKL126W	F	F	F	F	F	F	F	F	F	F	F	F	F	F	F	F	F	T	F	F	F	F	F
YKL129C	F	F	F	F	F	F	F	F	F	F	F	F	F	F	F	T	F	T	F	F	F	F	F
YKL130C	F	F	F	F	F	F	F	F	F	F	F	F	F	F	F	F	F	T	F	F	F	T	F
YKL135C	F	F	F	F	F	F	F	F	F	F	F	F	F	T	F	F	F	F	F	F	F	F	F
YKL139W	F	F	F	F	F	F	F	F	F	F	F	F	F	F	F	F	F	T	F	F	F	T	F
YKL140W	F	F	F	F	F	T	F	F	F	F	F	F	F	F	F	F	F	F	F	F	F	F	F
YKL142W	F	F	F	F	F	F	F	F	F	F	F	F	F	F	F	F	F	T	F	F	F	F	F
YKL143W	F	F	F	F	F	F	F	F	F	F	F	F	F	F	F	F	F	T	F	F	F	F	F
YKL144C	F	F	F	F	F	F	F	F	F	F	F	F	F	F	F	F	F	F	F	F	F	F	F
YKL145W	F	F	F	F	F	F	F	F	F	F	F	F	F	F	F	F	F	F	F	F	F	T	F
YKL152C	F	F	F	F	F	F	F	F	F	F	F	F	F	F	F	F	F	T	F	F	F	F	F
YKL155C	F	T	F	F	F	F	F	F	F	F	F	F	F	F	F	F	F	F	F	F	F	F	F
YKL157W	F	T	F	F	F	F	F	F	F	F	F	F	F	F	F	F	F	T	F	F	F	F	F
YKL159C	F	F	F	F	F	F	F	F	F	F	F	F	F	F	F	F	F	F	F	F	F	F	F
YKL160W	F	F	F	F	F	F	F	F	F	F	F	F	F	F	F	F	F	F	F	F	F	T	F
YKL161C	F	F	F	F	F	F	F	F	F	F	F	F	F	F	F	F	F	F	F	F	F	F	F
YKL166C	F	F	F	F	F	F	F	F	F	F	F	F	F	F	F	F	F	T	F	F	F	T	F
YKL167C	F	T	F	F	F	F	F	F	F	F	F	F	F	F	F	F	F	F	F	F	F	F	F
YKL168C	F	F	F	F	F	F	F	F	F	F	F	F	F	F	F	F	F	T	F	F	F	F	F
YKL171W	F	F	F	F	F	F	F	F	F	F	F	F	F	F	F	F	F	F	F	F	F	F	F
YKL172W	F	F	F	F	F	F	F	F	F	F	F	F	F	F	F	F	F	F	F	F	F	F	F
YKL173W	F	F	F	F	F	F	F	F	F	F	F	F	F	F	F	F	F	F	F	F	F	T	F
YKL179C	F	F	F	F	F	F	F	F	F	F	F	F	F	F	F	F	F	F	F	F	T	F	F
YKL180W	F	F	F	F	F	F	F	F	F	F	F	F	F	F	F	F	F	T	F	F	F	F	F
YKL181W	F	F	F	F	F	F	F	F	F	F	F	F	F	F	F	F	F	T	F	F	F	F	F
YKL182W	F	F	F	F	F	F	F	F	F	F	F	F	F	F	F	F	F	T	F	F	F	F	F
YKL185W	F	F	F	F	F	F	F	F	F	F	F	F	F	F	F	F	F	F	F	F	F	T	F
YKL186C	F	F	F	F	F	F	F	F	T	F	F	F	F	F	F	F	F	F	F	F	F	F	F
YKL189W	F	F	F	F	F	F	F	F	F	F	F	F	F	F	F	F	F	T	F	F	F	T	F
YKL190W	F	F	F	F	F	F	F	F	F	F	F	F	F	F	F	F	F	T	F	F	F	F	F
YKL191W	F	F	F	F	F	F	F	F	F	F	F	F	F	F	F	F	F	T	F	F	F	F	F
YKL193C	F	F	F	F	F	F	F	F	F	F	F	F	F	F	F	F	F	T	F	F	F	T	F
YKL195W	F	T	F	F	F	F	F	F	F	F	F	F	F	F	F	F	F	F	F	F	F	F	F
YKL196C	F	F	F	F	F	F	F	F	F	F	F	F	F	F	F	F	F	F	F	F	F	F	F
YKL197C	F	F	F	F	F	F	F	F	F	F	F	F	F	F	T	F	F	F	F	F	F	F	F
YKL203C	F	F	F	F	F	F	F	F	F	F	F	F	F	F	F	F	F	F	F	F	F	F	F

YKL204W	F	F	F	F	F	F	F	F	F	F	F	F	F	F	F	F	F	T	F	F	F	F	F
YKL205W	F	F	F	F	F	F	F	F	T	F	F	F	F	F	F	F	F	F	F	F	F	F	F
YKL206C	F	F	F	F	F	F	F	F	F	F	F	F	F	F	F	F	F	T	F	F	F	F	F
YKL209C	F	F	T	F	F	F	F	F	F	F	F	F	F	F	F	F	F	F	F	F	F	F	F
YKL210W	F	F	F	F	F	F	F	F	F	F	F	F	F	F	F	F	F	T	F	F	F	T	F
YKL211C	F	F	F	F	F	F	F	F	F	F	F	F	F	F	F	F	F	T	F	F	F	F	F
YKL212W	F	F	T	F	F	F	F	T	F	F	F	F	F	F	F	F	F	F	F	F	F	F	F
YKL213C	F	F	F	F	F	F	F	F	F	F	F	F	F	F	F	F	F	T	F	F	F	T	F
YKL214C	F	F	F	F	F	F	F	F	F	F	F	F	F	F	F	F	F	T	F	F	F	T	F
YKL215C	F	F	F	F	F	F	F	F	F	F	F	F	F	F	F	F	F	T	F	F	F	F	F
YKL217W	F	F	F	F	F	F	F	F	F	F	F	F	F	F	F	F	F	F	F	F	F	F	F
YKR001C	F	F	F	F	F	T	F	F	F	T	F	F	F	F	F	F	F	T	F	F	F	F	F
YKR002W	F	F	F	F	F	F	F	F	F	F	F	F	F	F	F	F	F	F	F	F	F	T	F
YKR006C	F	T	F	F	F	F	F	F	F	F	F	F	F	F	F	F	F	F	F	F	F	F	F
YKR007W	F	F	F	F	F	F	T	F	F	F	F	F	F	F	F	F	F	F	F	F	F	F	F
YKR008W	F	F	F	F	F	F	F	F	F	F	F	F	F	F	F	F	F	F	F	F	F	T	F
YKR010C	F	F	F	F	F	F	F	F	F	F	F	F	F	F	F	F	T	F	F	F	F	F	F
YKR014C	F	F	F	F	F	F	F	F	F	F	F	F	F	F	F	F	F	T	F	F	F	T	F
YKR020W	F	F	F	F	F	T	F	F	F	F	F	F	F	F	F	F	F	F	F	F	F	F	F
YKR021W	F	F	F	F	F	F	F	F	F	F	F	F	F	F	F	F	F	T	F	F	F	F	F
YKR022C	F	F	F	F	F	F	F	T	F	F	F	F	F	F	F	F	F	T	F	F	F	T	F
YKR024C	F	F	F	F	F	F	F	F	F	F	F	F	F	F	F	F	T	F	F	F	F	T	F
YKR025W	F	F	F	F	F	F	F	F	F	F	F	F	F	F	F	F	F	F	F	F	F	T	F
YKR026C	F	F	F	F	F	F	F	F	F	F	F	F	F	F	F	F	F	T	F	F	F	F	F
YKR027W	F	F	F	F	F	F	F	F	F	F	F	F	F	T	F	F	F	F	F	F	F	F	F
YKR028W	F	F	F	F	F	F	F	F	F	F	F	F	F	F	F	F	F	F	F	F	F	F	F
YKR029C	F	F	F	F	F	F	F	F	F	F	F	F	F	F	F	F	F	F	F	F	F	T	F
YKR030W	F	F	F	F	F	F	F	F	F	F	F	F	F	F	F	F	F	F	F	F	F	F	F
YKR035W-A	F	F	F	F	F	F	F	F	F	T	F	F	F	F	F	F	F	F	F	F	F	F	F
YKR036C	F	T	F	F	F	F	F	F	F	F	F	F	F	F	F	F	F	F	F	F	F	F	F
YKR037C	F	F	F	T	F	F	F	F	F	F	F	F	F	F	F	F	F	F	F	F	F	F	F
YKR038C	F	F	F	F	F	F	F	F	F	F	F	F	F	F	F	F	F	T	F	F	F	T	F
YKR039W	F	F	T	F	F	F	F	F	F	F	F	F	F	F	F	F	F	F	F	F	F	F	F
YKR048C	F	F	F	F	F	F	F	F	F	F	F	F	F	F	F	F	F	T	F	F	F	F	F
YKR054C	F	F	F	F	F	F	F	F	F	F	F	F	F	F	F	F	F	T	F	F	F	F	F
YKR055W	F	F	F	F	F	F	F	F	F	F	F	F	F	F	F	F	F	T	F	F	F	T	F
YKR056W	F	F	F	F	F	F	F	F	F	F	F	F	F	F	F	F	F	F	F	F	F	F	F
YKR058W	F	F	F	F	F	F	F	F	F	F	F	F	F	F	F	F	F	F	F	F	F	F	F
YKR059W	F	F	F	F	F	F	F	F	F	F	F	F	F	F	F	F	F	T	F	F	F	F	F
YKR060W	F	F	F	F	F	F	F	F	F	F	F	F	F	F	F	F	T	F	F	F	F	T	F
YKR062W	F	F	F	F	F	F	F	F	F	F	F	F	F	F	F	F	F	F	F	F	F	T	F
YKR065C	F	T	F	F	F	F	F	F	F	F	F	F	F	F	F	F	F	F	F	F	F	F	F
YKR066C	F	T	F	F	F	F	F	F	F	F	F	F	F	F	F	F	F	F	F	F	F	F	F
YKR067W	F	F	F	F	F	F	F	T	F	F	F	F	F	F	F	F	F	F	F	F	F	F	F
YKR068C	F	F	F	F	F	F	F	F	F	F	F	F	F	T	F	F	F	F	F	F	F	F	F
YKR071C	F	F	F	F	F	F	F	F	F	F	F	F	F	F	F	F	F	T	F	F	F	F	F
YKR072C	F	F	F	F	F	F	F	F	F	F	F	F	F	F	F	F	F	T	F	F	F	F	F
YKR079C	F	F	F	F	F	F	F	F	F	F	F	F	F	F	F	F	F	T	F	F	F	T	F
YKR081C	F	F	F	F	F	F	F	F	F	F	F	F	F	F	F	F	T	F	F	F	F	T	F
YKR082W	F	F	F	F	F	F	F	F	T	F	F	F	F	F	F	F	F	F	F	F	F	F	F
YKR083C	F	F	F	T	F	F	F	F	F	F	F	F	F	F	F	F	F	F	F	F	F	F	F
YKR085C	F	T	F	F	F	F	F	F	F	F	F	F	F	F	F	F	F	F	F	F	F	F	F
YKR086W	F	F	F	F	F	F	F	F	F	F	F	F	F	F	F	F	F	T	F	F	F	T	F
YKR088C	F	F	F	F	F	T	F	F	F	T	F	F	F	T	F	F	F	F	F	T	F	F	F
YKR091W	F	F	F	F	F	F	F	F	F	F	F	F	F	F	F	F	F	F	F	F	F	F	F
YKR092C	F	F	F	F	F	F	F	F	F	F	F	F	F	F	F	F	T	F	F	F	F	F	F
YKR094C	F	F	F	F	F	F	F	F	F	F	F	F	F	F	F	F	F	T	F	F	F	F	F
YKR095W	F	F	F	F	F	F	F	F	T	F	F	F	F	F	F	F	F	F	F	F	F	F	F
YKR096W	F	F	F	F	F	F	F	F	F	F	F	F	F	F	F	F	F	T	F	F	F	T	F
YKR099W	F	F	F	F	F	F	F	F	F	F	F	F	F	F	F	F	F	F	F	F	F	T	F
YKR101W	F	F	F	F	F	F	F	F	F	F	F	F	F	F	F	F	F	F	F	F	F	F	F
YLL001W	F	T	F	F	F	T	F	F	F	F	F	F	F	F	F	F	F	F	F	F	F	F	F
YLL002W	F	F	F	F	F	F	F	F	F	F	F	F	F	F	F	F	F	F	F	F	F	T	F
YLL003W	F	F	F	T	F	F	F	F	F	F	F	F	F	F	F	F	F	F	F	F	F	F	F
YLL004W	F	F	F	F	F	F	F	F	F	F	F	F	F	F	F	F	F	F	F	F	F	F	F
YLL006W	F	T	F	F	F	T	F	F	F	F	F	F	F	F	F	F	F	F	F	F	F	F	F
YLL008W	F	F	F	F	F	F	F	F	F	F	F	F	F	F	F	F	T	T	F	F	F	T	F
YLL009C	F	F	F	F	F	F	F	F	F	F	F	F	F	F	F	F	F	T	F	F	F	T	F

YLL010C	F	F	F	F	T	F	F	F	F	F	F	F	F	F	F	F	F	F	F	F	F	F	F
YLL011W	F	F	F	F	F	F	F	F	F	F	F	F	F	F	F	F	T	F	F	F	F	F	F
YLL013C	F	F	F	F	F	F	F	F	F	F	F	F	F	F	F	F	F	T	F	F	F	F	F
YLL017W	F	F	F	F	F	F	F	F	F	F	F	F	F	F	F	F	F	F	F	F	F	F	F
YLL018C	F	F	F	F	F	F	F	F	F	F	F	F	F	F	F	F	F	T	F	F	F	F	F
YLL018C-A	F	F	F	F	F	F	F	F	F	F	F	F	F	F	F	F	F	T	F	F	F	T	F
YLL021W	F	F	F	F	F	F	F	F	F	F	F	F	F	F	F	F	F	F	F	F	F	F	F
YLL022C	F	F	F	F	F	F	F	F	F	F	F	F	F	F	F	F	F	F	F	F	F	T	F
YLL023C	F	F	F	F	F	F	F	T	F	F	F	F	F	F	F	F	F	F	F	F	F	F	F
YLL024C	F	F	F	F	F	F	F	F	F	F	F	F	F	F	F	F	F	T	F	F	F	T	F
YLL026W	F	F	F	F	F	F	F	F	F	F	F	F	F	F	F	F	F	T	F	F	F	F	F
YLL029W	F	F	F	F	F	F	F	F	F	F	F	F	F	F	F	F	F	T	F	F	F	F	F
YLL034C	F	F	F	F	F	F	F	F	F	F	F	F	F	F	F	F	T	F	F	F	F	T	F
YLL035W	F	F	F	F	F	F	F	F	F	F	F	F	F	F	F	F	F	F	F	F	F	F	F
YLL036C	F	F	F	F	F	F	F	F	F	F	F	F	F	F	F	F	F	F	F	F	F	T	F
YLL039C	F	F	F	F	F	F	F	F	F	F	F	F	F	F	F	F	F	T	F	F	F	T	F
YLL040C	F	F	F	F	F	F	F	F	F	T	F	F	F	F	F	F	F	T	F	F	F	F	F
YLL042C	F	F	F	F	F	F	F	F	F	F	F	F	F	F	F	F	F	F	F	F	F	F	F
YLL043W	F	F	T	F	T	F	F	F	F	F	F	F	F	F	F	F	F	F	F	F	F	F	F
YLL045C	F	F	F	F	F	F	F	F	F	F	F	F	F	F	F	F	F	T	F	F	F	F	F
YLL049W	F	F	F	F	F	F	F	F	F	F	F	F	F	F	F	F	F	F	F	F	F	F	F
YLL050C	F	F	F	F	F	F	F	F	F	F	F	F	F	F	F	F	F	F	F	F	F	F	F
YLR002C	F	F	F	F	F	F	F	F	F	F	F	F	F	F	F	F	T	F	F	F	F	T	F
YLR003C	F	F	F	F	F	F	F	F	F	F	F	F	F	F	F	F	F	F	F	F	F	T	F
YLR005W	F	F	F	F	F	F	F	F	F	F	F	F	F	F	F	F	F	F	F	F	F	T	F
YLR006C	F	F	F	F	F	F	F	F	F	F	F	F	F	F	F	F	F	T	F	F	F	F	F
YLR007W	F	F	F	F	F	F	F	F	F	F	F	F	F	F	F	F	F	F	F	F	F	F	F
YLR008C	F	F	F	F	F	F	F	F	F	F	F	F	F	F	F	F	F	F	F	F	F	F	F
YLR009W	F	F	F	F	F	F	F	F	F	F	F	F	F	F	F	F	F	F	F	F	F	F	F
YLR010C	F	F	F	F	F	F	F	F	F	F	F	F	F	F	F	F	F	F	F	F	F	F	F
YLR015W	F	F	F	F	F	F	F	F	F	F	F	F	F	F	F	F	F	F	F	F	F	T	F
YLR016C	F	F	F	F	F	F	F	F	F	F	F	F	F	F	F	F	F	T	F	F	F	T	F
YLR018C	F	F	F	F	F	F	F	F	T	F	F	F	F	F	F	F	F	F	F	F	F	F	F
YLR021W	F	F	F	F	F	F	F	F	F	F	F	F	F	F	F	F	F	F	F	F	F	F	F
YLR022C	F	F	F	F	F	F	F	F	F	F	F	F	F	F	F	F	F	T	F	F	F	T	F
YLR024C	F	F	F	F	F	F	F	F	F	F	F	F	F	F	F	F	F	T	F	F	F	F	F
YLR025W	F	F	F	F	F	F	F	F	F	T	F	F	F	F	F	F	F	F	F	F	F	F	F
YLR026C	F	F	F	F	F	F	F	F	F	F	F	F	F	F	F	F	F	F	F	F	F	F	F
YLR028C	F	F	F	F	F	F	F	F	F	F	F	F	F	F	F	F	F	T	F	F	F	F	F
YLR029C	F	F	F	F	F	F	F	F	F	F	F	F	F	F	F	F	F	F	F	F	F	F	F
YLR031W	F	F	F	F	F	F	F	F	F	F	F	F	F	F	F	F	F	F	F	F	F	F	F
YLR032W	F	F	F	F	F	F	F	F	F	F	F	F	F	F	F	F	F	T	F	F	F	T	F
YLR033W	F	F	F	F	F	F	F	F	F	F	F	F	F	F	F	F	F	F	F	F	F	T	F
YLR035C	F	F	F	F	F	F	F	F	F	F	F	F	F	F	F	F	F	T	F	F	F	T	F
YLR039C	F	F	F	F	F	F	F	F	F	F	F	F	T	F	F	F	F	F	F	F	F	F	F
YLR043C	F	F	F	F	F	F	F	F	F	F	F	F	F	F	F	F	F	T	F	F	F	T	F
YLR044C	T	F	F	F	F	F	F	F	F	F	F	F	F	F	F	F	F	T	F	F	F	T	F
YLR045C	F	F	F	T	F	F	F	F	F	F	F	F	F	F	F	F	F	F	F	F	F	F	F
YLR052W	F	F	F	F	F	F	F	F	F	F	F	F	F	F	F	F	F	F	F	F	F	T	F
YLR055C	F	F	F	F	F	F	F	F	F	F	F	F	F	F	F	F	F	F	F	F	F	T	F
YLR056W	F	F	F	F	F	F	F	T	F	F	F	F	F	F	F	F	F	F	F	F	F	F	F
YLR058C	F	F	F	F	F	F	F	F	F	F	F	F	F	F	F	F	F	T	F	F	F	T	F
YLR060W	F	F	F	F	F	F	F	F	F	F	F	F	F	F	F	F	F	T	F	F	F	F	F
YLR064W	F	F	F	F	F	F	F	T	F	F	F	F	F	F	F	F	F	F	F	F	F	F	F
YLR069C	F	T	F	F	F	F	F	F	F	F	F	F	F	F	F	F	F	F	F	F	F	F	F
YLR071C	F	F	F	F	F	F	F	F	F	F	F	F	F	F	F	F	F	F	F	F	F	T	F
YLR074C	F	F	F	F	F	F	F	T	F	F	F	F	F	F	F	F	F	F	F	F	F	T	F
YLR075W	F	F	F	F	F	F	F	F	F	F	F	F	F	F	F	F	F	T	F	F	F	F	F
YLR078C	F	F	F	F	F	F	F	F	F	F	F	F	F	F	F	F	F	F	F	F	F	F	F
YLR079W	F	F	F	F	F	F	F	F	F	F	F	F	F	F	F	F	F	T	F	F	F	T	F
YLR080W	F	F	T	F	F	F	F	F	F	F	F	F	F	F	F	F	F	F	F	F	F	F	F
YLR085C	F	F	F	F	F	F	F	F	F	F	F	F	F	F	F	F	F	T	F	F	F	T	F
YLR086W	F	F	F	F	F	F	F	F	F	F	F	F	F	F	F	F	F	F	F	F	F	T	F
YLR090W	F	T	F	F	F	F	F	F	F	F	F	F	F	F	F	F	F	F	F	F	F	T	F
YLR093C	F	F	T	F	F	F	F	F	F	F	F	F	F	F	F	F	F	F	F	F	F	F	F
YLR095C	F	F	F	F	F	F	F	F	F	F	F	F	F	F	F	F	F	F	F	F	F	T	F
YLR096W	T	F	F	F	F	F	F	F	F	F	F	F	F	F	F	F	F	F	F	F	F	F	F
YLR097C	F	F	F	F	F	F	F	F	F	F	F	F	F	F	F	F	F	F	F	F	F	F	F

YLR098C	F	F	F	F	F	F	F	F	F	F	F	F	F	F	F	F	F	T	F	F	F	T	F
YLR100W	F	F	F	F	F	F	F	F	F	F	F	F	F	F	F	F	F	F	F	F	T	F	F
YLR102C	F	F	F	F	F	F	F	F	F	F	F	F	F	F	F	F	F	T	F	F	F	T	F
YLR103C	F	F	F	F	F	F	F	F	F	F	F	F	F	F	F	F	F	F	F	F	F	T	F
YLR105C	F	T	F	F	F	F	F	F	F	F	F	F	F	F	F	F	F	F	F	F	F	F	F
YLR106C	F	F	F	F	F	F	F	F	F	F	F	F	F	F	F	F	F	T	F	F	F	T	F
YLR109W	F	F	F	F	F	F	F	F	F	F	F	F	F	F	F	F	F	T	F	F	F	T	F
YLR113W	F	F	F	F	F	F	F	F	F	F	F	F	F	F	F	F	F	T	F	F	F	F	F
YLR115W	F	F	F	F	F	F	F	F	F	F	F	F	F	F	F	F	F	F	F	F	F	T	F
YLR116W	F	F	F	F	F	F	F	F	F	F	F	F	F	F	F	F	F	F	F	F	F	T	F
YLR117C	F	F	F	F	F	F	F	F	F	F	F	F	F	F	F	F	F	F	F	F	F	T	F
YLR119W	F	F	F	F	F	F	F	F	F	F	F	F	F	F	F	F	F	F	F	F	F	F	F
YLR127C	F	F	F	F	F	F	F	F	F	F	F	F	F	F	F	F	F	T	F	F	F	T	F
YLR128W	F	F	F	F	F	F	F	F	F	F	F	F	F	F	F	F	F	F	F	F	F	F	F
YLR129W	F	F	F	F	F	F	F	F	F	F	F	F	F	F	F	F	F	T	F	F	F	T	F
YLR131C	F	F	F	F	F	F	F	F	F	F	F	F	F	F	F	F	F	T	F	F	F	T	T
YLR132C	F	T	F	F	F	F	F	F	F	F	F	F	F	F	F	F	F	T	F	F	F	T	F
YLR133W	F	F	F	F	F	F	F	F	F	F	F	F	F	F	F	F	F	T	F	F	F	F	F
YLR135W	F	F	F	F	F	F	F	F	F	F	F	F	F	F	F	F	F	T	F	F	F	T	F
YLR138W	F	F	F	F	T	F	F	F	F	F	F	F	F	F	F	F	F	F	F	F	F	F	F
YLR139C	F	T	F	F	F	F	F	F	F	F	F	F	F	F	F	F	F	F	F	F	F	F	F
YLR141W	F	F	F	F	F	F	F	F	F	F	F	F	F	F	F	F	T	F	F	F	F	F	F
YLR144C	F	F	F	F	F	F	F	F	F	F	F	F	F	F	F	F	F	T	F	F	F	T	F
YLR145W	F	F	F	F	F	F	F	F	F	F	F	F	F	F	F	F	F	T	F	F	F	T	F
YLR146C	F	F	F	F	F	F	F	F	F	F	F	F	F	F	F	F	F	T	F	F	F	F	F
YLR147C	F	F	F	F	F	F	F	F	F	F	F	F	F	F	F	F	F	F	F	F	F	F	F
YLR148W	F	F	F	F	F	F	F	F	F	T	F	F	F	F	F	F	F	F	F	F	F	F	F
YLR150W	F	F	F	F	F	F	F	F	F	F	F	F	F	F	F	F	F	T	F	F	F	F	F
YLR153C	F	F	F	F	F	F	F	F	F	F	F	F	F	F	F	F	F	F	F	F	F	T	F
YLR163C	F	T	F	F	F	F	F	F	F	F	F	F	F	F	F	F	F	F	F	F	F	F	F
YLR166C	T	F	F	F	T	F	F	F	F	F	T	F	F	F	F	F	F	F	F	F	F	F	T
YLR167W	F	F	F	F	F	F	F	F	F	F	F	F	F	F	F	F	F	F	F	F	F	F	F
YLR170C	F	F	F	F	F	F	F	F	F	F	F	F	F	F	F	F	F	F	F	F	F	F	F
YLR172C	F	F	F	F	F	F	F	F	F	F	F	F	F	F	F	F	F	T	F	F	F	F	F
YLR175W	F	F	F	F	F	F	F	F	F	F	F	F	F	F	F	F	T	F	F	F	F	F	F
YLR176C	F	F	F	F	F	F	F	F	F	F	F	F	F	F	F	F	F	F	F	F	F	T	F
YLR177W	F	F	F	F	F	F	F	F	F	F	F	F	F	F	F	F	F	T	F	F	F	F	F
YLR178C	F	F	F	F	F	F	F	F	F	F	F	F	F	F	F	F	F	T	F	F	F	T	F
YLR179C	F	F	F	F	F	F	F	F	F	F	F	F	F	F	F	F	F	T	F	F	F	T	F
YLR180W	F	F	F	F	F	F	F	F	F	F	F	F	F	F	F	F	F	T	F	F	F	F	F
YLR181C	F	F	F	F	F	F	F	F	F	T	F	F	F	F	F	F	F	F	F	F	F	F	F
YLR182W	F	F	F	F	F	F	F	F	F	F	F	F	F	F	F	F	F	F	F	F	F	T	F
YLR186W	F	F	F	F	F	F	F	F	F	F	F	F	F	F	F	F	F	T	F	F	F	T	F
YLR187W	T	F	F	F	T	F	F	F	F	F	T	F	F	F	F	F	F	T	F	F	F	F	T
YLR188W	F	T	F	F	F	F	F	F	F	F	F	F	F	F	F	F	F	F	F	F	F	F	F
YLR189C	F	F	F	F	F	F	F	F	F	F	F	F	F	F	F	F	F	T	F	F	F	F	F
YLR190W	T	F	F	F	F	F	F	F	F	F	F	F	F	F	F	F	F	F	F	F	F	F	F
YLR191W	F	F	F	F	F	F	F	F	F	F	F	F	F	F	T	F	F	F	F	F	F	F	F
YLR192C	F	F	F	F	F	F	F	F	F	F	F	F	F	F	F	F	F	T	F	F	F	F	F
YLR195C	F	F	F	F	F	F	F	F	F	F	F	F	F	F	F	F	F	T	F	F	F	F	F
YLR196W	F	F	F	F	F	F	F	F	F	F	F	F	F	F	F	F	T	T	F	F	F	T	F
YLR197W	F	F	F	F	F	F	F	F	F	F	F	F	F	F	F	F	T	F	F	F	F	F	F
YLR199C	F	F	F	F	F	F	F	F	F	F	F	F	F	F	F	F	F	T	F	F	F	F	F
YLR200W	F	F	F	F	F	F	F	F	F	F	F	F	F	F	F	F	F	T	F	F	F	F	F
YLR203C	F	T	F	F	F	F	F	F	F	F	F	F	F	F	F	F	F	F	F	F	F	F	F
YLR206W	F	F	F	F	F	F	F	F	F	F	F	F	F	F	F	T	F	F	F	F	F	F	F
YLR207W	F	F	F	F	F	F	F	F	F	F	F	F	F	F	F	F	F	F	F	F	F	F	F
YLR208W	F	F	F	F	F	F	F	F	F	F	F	F	F	F	F	F	F	F	T	F	F	F	F
YLR209C	F	F	F	F	F	F	F	F	F	F	F	F	F	F	F	F	F	F	F	F	F	F	F
YLR210W	F	F	F	T	F	F	F	F	F	F	F	F	F	F	F	F	F	F	F	F	F	T	F
YLR212C	F	F	F	T	F	F	F	F	F	F	F	F	F	F	F	F	F	F	F	F	F	F	F
YLR215C	F	F	F	F	F	F	F	F	F	F	F	F	F	F	F	F	F	T	F	F	F	F	F
YLR216C	F	F	F	F	F	F	F	F	F	F	F	F	F	F	F	F	F	T	F	F	F	F	F
YLR221C	F	F	F	F	F	F	F	F	F	F	F	F	F	F	F	F	T	F	F	F	F	F	F
YLR222C	F	F	F	F	F	F	F	F	F	F	F	F	F	F	F	F	T	F	F	F	F	F	F
YLR223C	F	F	F	F	F	F	F	F	F	F	F	F	F	F	F	F	F	F	F	F	F	T	F
YLR224W	F	F	F	F	F	F	F	F	F	F	F	F	F	F	F	F	F	F	F	F	F	F	F
YLR226W	F	F	F	F	F	F	F	F	F	F	F	F	F	F	F	F	F	F	F	F	F	T	F

YLR229C	F	F	F	F	F	F	F	F	F	F	F	F	F	F	F	F	F	F	F	F	F	F	F
YLR233C	F	F	F	F	F	F	F	F	F	F	F	F	F	F	F	F	F	F	F	F	F	F	F
YLR234W	F	F	F	F	F	F	F	F	F	F	F	F	F	F	F	F	F	T	F	F	F	T	F
YLR238W	F	F	F	F	F	F	F	F	F	F	F	F	F	F	F	F	F	F	F	F	F	F	F
YLR240W	F	F	F	F	F	F	F	F	F	T	F	F	F	F	F	F	F	F	F	F	F	F	F
YLR244C	F	F	F	F	F	F	F	F	F	F	F	F	F	F	F	F	F	T	F	F	F	T	F
YLR245C	F	F	F	F	F	F	F	F	F	F	F	F	F	F	F	F	F	T	F	F	F	T	F
YLR248W	F	F	F	F	F	F	F	F	F	F	F	F	F	F	F	F	F	T	F	F	F	F	F
YLR249W	F	F	F	F	F	F	F	F	F	F	F	F	F	F	F	F	F	T	F	F	F	F	F
YLR254C	F	F	F	F	F	F	F	F	F	F	F	F	F	F	F	F	F	T	F	F	F	T	F
YLR256W	F	F	F	F	F	F	F	F	F	F	F	F	F	F	F	F	F	F	F	F	F	F	F
YLR257W	F	F	F	F	F	F	F	F	F	F	F	F	F	F	F	F	F	T	F	F	F	F	F
YLR258W	F	F	F	F	F	T	F	F	F	F	F	F	F	F	F	F	F	T	F	F	F	T	F
YLR259C	F	T	F	F	F	F	F	F	F	F	F	F	F	F	F	F	F	F	F	F	F	F	F
YLR262C	T	F	F	F	F	F	F	F	F	F	F	F	F	F	F	F	F	F	F	F	F	F	F
YLR263W	F	F	F	F	F	F	F	F	F	F	F	F	F	F	F	F	F	T	F	F	F	F	F
YLR264W	F	F	F	F	F	F	F	F	F	F	F	F	F	F	F	F	F	T	F	F	F	F	F
YLR265C	F	F	F	F	F	F	F	F	F	F	F	F	F	F	F	F	F	T	F	F	F	T	F
YLR267W	F	F	F	F	F	F	F	F	F	F	F	F	F	F	F	F	F	F	F	F	F	F	F
YLR268W	F	F	F	F	F	F	F	F	F	F	F	F	T	F	F	F	F	F	F	F	F	F	F
YLR270W	F	F	F	F	F	F	F	F	F	F	F	F	F	F	F	F	F	T	F	F	F	T	F
YLR272C	F	F	F	F	F	F	F	F	F	F	F	F	F	F	F	F	T	T	F	F	F	T	F
YLR274W	F	F	F	F	F	F	F	F	F	F	F	F	F	F	F	F	F	F	F	F	F	F	F
YLR275W	F	F	F	F	F	F	F	F	F	F	F	F	F	F	F	F	F	F	F	F	F	T	F
YLR276C	F	F	F	F	F	F	F	F	F	F	F	F	F	F	F	F	F	F	F	F	F	F	F
YLR277C	F	F	F	F	F	F	F	F	F	F	F	F	F	F	F	F	F	F	F	F	F	T	F
YLR287C	F	F	F	F	F	F	F	F	F	F	F	F	F	F	F	F	F	T	F	F	F	F	F
YLR288C	F	F	F	F	F	F	F	F	F	F	F	F	F	F	F	F	F	F	F	F	F	F	F
YLR291C	F	F	F	F	F	T	F	F	F	F	F	F	F	F	F	F	F	T	F	F	F	F	F
YLR292C	F	F	F	F	F	F	F	T	F	F	F	F	F	F	F	F	F	F	F	F	F	F	F
YLR293C	F	F	F	F	F	F	F	F	F	F	F	F	F	F	F	F	F	F	F	F	F	F	F
YLR295C	F	T	F	F	F	F	F	F	F	F	F	F	F	F	F	F	F	F	F	F	F	F	F
YLR298C	F	F	F	F	F	F	F	F	F	F	F	F	F	F	F	F	F	F	F	F	F	T	F
YLR300W	F	F	T	F	F	F	F	F	F	F	F	F	F	F	F	F	F	F	F	F	F	F	F
YLR304C	F	F	F	F	F	F	F	F	F	F	F	F	F	F	F	F	F	F	F	F	F	F	F
YLR305C	F	F	F	F	F	F	F	F	F	F	F	F	F	F	F	F	F	F	F	F	F	F	F
YLR306W	F	F	F	F	F	F	F	F	F	F	F	F	F	F	F	F	F	F	F	F	F	F	F
YLR309C	F	F	F	F	F	F	F	F	F	F	F	F	F	F	F	F	F	T	F	F	F	F	F
YLR310C	F	F	F	F	F	F	F	F	F	F	F	F	F	F	F	F	F	T	F	F	F	F	F
YLR312W-A	F	T	F	F	F	F	F	F	F	F	F	F	F	F	F	F	F	F	F	F	F	F	F
YLR313C	T	F	F	F	F	F	F	F	F	F	T	F	F	F	F	F	F	F	F	F	F	F	T
YLR314C	F	F	F	F	F	F	F	F	F	F	F	F	F	F	F	F	F	F	F	F	F	F	F
YLR315W	F	F	F	T	F	F	F	F	F	F	F	F	F	F	F	F	F	F	F	F	F	F	F
YLR318W	F	F	F	F	F	F	F	F	F	F	F	F	F	F	F	F	F	F	F	F	F	F	F
YLR319C	F	F	F	F	T	F	F	F	F	F	T	F	F	F	F	F	F	T	F	F	F	F	F
YLR320W	F	F	F	F	F	F	F	F	F	F	F	F	F	F	F	F	F	F	F	F	F	F	F
YLR321C	F	F	F	F	F	F	F	F	F	F	F	F	F	F	F	F	F	F	F	F	F	T	F
YLR323C	F	F	F	F	F	F	F	F	F	F	F	F	F	F	F	F	F	F	F	F	F	T	F
YLR324W	F	F	F	F	F	F	F	F	F	F	F	F	F	F	T	F	F	F	F	F	F	F	F
YLR325C	F	F	F	F	F	F	F	F	F	F	F	F	F	F	F	F	F	T	F	F	F	F	F
YLR328W	F	F	F	F	F	F	F	F	F	F	F	F	F	F	F	F	F	T	F	F	F	T	F
YLR329W	F	F	F	F	F	F	F	F	F	F	F	F	F	F	F	F	F	F	F	F	F	F	F
YLR330W	F	F	F	F	F	F	F	F	F	F	F	F	F	T	F	F	F	F	F	F	F	F	F
YLR332W	F	F	F	F	T	F	F	F	F	F	F	F	F	F	F	F	F	F	F	F	F	F	F
YLR335W	F	F	F	F	F	F	F	F	T	F	F	F	F	F	F	F	F	F	F	F	F	F	F
YLR336C	F	F	F	F	F	F	F	F	F	F	F	F	F	F	F	F	T	F	F	F	F	F	F
YLR337C	F	F	F	F	F	T	F	F	F	F	F	F	F	F	F	T	F	F	F	F	F	F	F
YLR340W	F	F	F	F	F	F	F	F	F	F	F	F	F	F	F	F	F	F	F	F	F	F	F
YLR342W	F	F	T	F	F	F	F	F	F	F	F	F	F	F	F	F	F	F	F	F	F	F	F
YLR345W	F	F	F	F	F	F	F	F	F	F	F	F	F	F	F	F	F	T	F	F	F	F	F
YLR347C	F	F	F	F	F	T	F	F	T	F	F	F	F	F	F	F	F	T	F	F	F	F	F
YLR350W	F	F	F	F	F	F	F	T	F	F	F	F	F	F	F	F	F	F	F	F	F	F	F
YLR352W	F	F	F	F	F	F	F	F	F	F	F	F	F	F	F	F	F	F	F	F	F	F	F
YLR353W	T	F	F	F	T	F	F	F	F	F	T	F	F	F	F	F	F	F	F	F	F	F	T
YLR354C	F	F	F	F	F	F	F	F	F	F	F	F	F	F	F	F	F	F	F	F	F	T	F
YLR355C	F	F	F	F	F	F	F	F	F	F	F	F	F	F	F	F	F	F	F	F	F	F	F
YLR357W	F	F	F	F	F	F	F	F	F	F	F	F	F	F	F	F	F	F	F	F	F	T	F
YLR359W	F	F	F	F	F	F	F	F	F	F	F	F	F	F	F	F	F	F	F	F	F	F	F

YLR360W	F	F	F	F	F	F	F	F	F	T	F	F	F	F	F	F	F	F	F	F	F	F	F
YLR361C	F	F	F	F	F	F	F	F	F	F	F	F	F	F	F	F	F	F	F	F	F	F	F
YLR362W	F	F	F	F	F	F	F	F	F	F	F	F	F	F	F	F	F	T	F	F	F	F	F
YLR367W	F	F	F	F	F	F	F	F	F	F	F	F	F	F	F	F	T	F	F	F	F	F	F
YLR368W	F	F	F	F	F	F	F	F	F	F	F	F	F	F	F	F	F	F	F	F	F	F	F
YLR369W	F	T	F	F	F	F	F	F	F	F	F	F	F	F	F	F	F	F	F	F	F	F	F
YLR370C	F	F	F	F	F	F	F	F	F	F	F	F	F	F	F	T	F	F	F	F	F	F	F
YLR371W	F	F	F	F	F	F	F	F	F	F	T	F	F	F	F	F	F	T	F	F	F	F	T
YLR372W	F	F	F	F	F	F	F	T	F	F	F	F	F	F	F	F	F	F	F	F	F	F	F
YLR373C	F	F	F	F	F	F	F	F	F	F	F	F	F	F	F	F	F	F	F	F	F	T	F
YLR377C	F	F	F	F	F	F	F	F	F	F	F	F	F	F	F	F	F	F	F	F	F	F	F
YLR378C	F	F	F	F	F	F	F	T	F	F	F	F	F	F	F	F	F	F	F	F	F	F	F
YLR380W	T	F	F	F	F	F	F	F	F	F	F	F	F	F	F	F	F	F	F	F	F	F	F
YLR381W	F	F	F	T	F	F	F	F	F	F	F	F	F	F	F	F	F	F	F	F	F	F	F
YLR382C	F	T	F	F	F	F	F	F	F	F	F	F	F	F	F	F	F	F	F	F	F	F	F
YLR383W	F	F	F	F	F	F	F	F	F	F	F	F	F	F	F	F	F	T	F	F	F	T	F
YLR384C	F	F	F	F	F	F	F	F	F	F	F	F	F	F	F	F	F	T	F	F	F	F	F
YLR385C	F	F	F	F	F	F	F	F	F	F	F	F	F	F	F	F	F	F	F	F	F	T	F
YLR386W	F	F	T	F	F	F	F	F	F	F	F	F	F	F	F	F	F	F	F	F	F	F	F
YLR387C	F	F	F	F	F	F	F	F	F	F	F	F	F	F	F	F	F	T	F	F	F	F	F
YLR392C	F	F	F	F	F	F	F	F	F	F	F	F	F	F	F	F	F	T	F	F	F	F	F
YLR394W	F	F	F	F	F	F	F	F	F	F	F	F	F	F	F	F	F	F	F	F	F	F	F
YLR396C	F	F	F	F	F	F	T	F	F	F	F	F	F	F	F	F	F	F	F	F	F	F	F
YLR398C	F	F	F	F	F	F	F	F	F	F	F	F	F	F	F	F	F	T	F	F	F	F	F
YLR399C	F	F	F	F	F	F	F	F	F	F	F	F	F	F	F	F	F	F	F	F	F	T	F
YLR403W	F	F	F	F	F	F	F	F	F	F	F	F	F	F	F	F	F	T	F	F	F	T	F
YLR406C	F	F	F	F	F	F	F	F	F	F	F	F	F	F	F	F	F	T	F	F	F	F	F
YLR407W	F	F	F	F	T	F	F	F	F	F	F	F	F	F	F	F	F	F	F	F	F	F	F
YLR409C	F	F	F	F	F	F	F	F	F	F	F	F	F	F	F	F	F	F	F	F	F	T	F
YLR410W	F	F	F	F	F	F	F	F	F	F	F	F	F	F	F	F	F	T	F	F	F	F	F
YLR417W	F	F	F	F	F	F	F	F	F	F	F	F	F	F	F	F	F	T	F	F	F	F	F
YLR418C	F	F	F	F	F	F	F	F	F	F	F	F	F	F	F	F	F	F	F	F	F	T	F
YLR421C	F	F	F	F	F	F	F	F	F	F	F	F	F	F	F	F	F	T	F	F	F	T	F
YLR423C	F	F	F	F	F	T	F	F	F	F	F	F	F	F	F	F	F	F	F	F	F	F	F
YLR424W	F	F	F	F	F	F	F	F	F	F	F	F	F	F	F	F	F	T	F	F	F	T	F
YLR425W	F	F	F	F	F	F	F	F	F	F	F	F	F	F	F	F	F	F	F	F	F	F	F
YLR429W	F	F	F	F	F	F	F	F	F	F	F	F	F	F	F	T	F	F	F	F	F	F	F
YLR430W	F	F	F	F	F	F	F	F	F	F	F	F	F	F	F	F	F	F	F	F	F	T	F
YLR431C	F	F	F	F	F	F	F	F	F	F	F	F	F	F	F	F	F	F	F	F	F	F	F
YLR432W	F	F	F	F	F	F	F	F	F	F	F	F	F	F	F	F	F	T	F	F	F	F	F
YLR433C	T	F	F	F	F	F	F	F	F	F	F	F	F	F	F	F	F	F	F	F	F	F	F
YLR435W	F	F	F	F	F	F	F	F	F	F	F	F	F	F	F	F	F	T	F	F	F	T	F
YLR436C	F	F	F	F	F	F	F	F	F	F	F	F	F	F	F	F	F	T	F	F	F	F	F
YLR437C	F	F	F	F	F	F	F	F	F	F	F	F	F	F	F	F	F	F	F	F	F	F	F
YLR438C-A	F	F	F	F	F	F	F	F	F	F	F	F	F	F	F	F	F	T	F	F	F	T	F
YLR438W	F	F	F	F	F	F	F	F	F	F	F	F	F	F	F	F	F	T	F	F	F	T	F
YLR439W	F	T	F	F	F	F	F	F	F	F	F	F	F	F	F	F	F	F	F	F	F	F	F
YLR440C	F	F	F	F	F	F	F	T	F	F	F	F	F	F	F	F	F	F	F	F	F	F	F
YLR441C	F	F	F	F	F	F	F	F	F	F	F	F	F	F	F	F	F	T	F	F	F	T	F
YLR442C	F	F	F	F	F	F	F	F	T	F	F	F	F	F	F	F	F	F	F	F	F	F	F
YLR447C	F	F	F	F	F	F	T	F	F	F	F	F	F	F	F	F	F	F	F	F	F	F	F
YLR448W	F	F	F	F	F	F	F	F	F	F	F	F	F	F	F	F	F	T	F	F	F	F	F
YLR449W	F	F	F	F	F	F	F	F	F	F	F	F	F	F	F	F	T	F	F	F	F	T	F
YLR450W	F	F	F	F	F	F	F	F	T	F	F	F	F	F	F	F	F	F	F	F	F	F	F
YLR451W	F	F	F	F	F	F	F	F	F	F	F	F	F	F	F	F	F	F	F	F	F	T	F
YLR452C	F	F	F	F	F	F	F	F	F	F	F	F	F	F	F	F	F	T	F	F	F	F	F
YLR453C	F	F	F	F	F	F	F	F	F	F	F	F	F	F	F	F	F	T	F	F	F	T	F
YLR455W	F	F	F	F	F	F	F	F	F	F	F	F	F	F	F	F	F	F	F	F	F	T	F
YLR457C	F	F	F	T	F	F	F	F	F	F	F	F	F	F	F	F	F	F	F	F	F	F	F
YML001W	F	F	F	F	F	F	F	F	F	F	F	F	F	F	F	F	F	T	F	F	F	T	F
YML004C	F	F	F	F	F	F	F	F	F	F	F	F	F	F	F	F	F	T	F	F	F	T	F
YML006C	F	F	F	F	F	F	F	F	F	F	F	F	F	F	F	F	F	T	F	F	F	F	F
YML007W	F	F	F	F	F	F	F	F	F	F	F	F	F	F	F	F	F	T	F	F	F	T	F
YML008C	F	F	F	F	F	F	F	F	F	F	F	F	F	F	F	F	F	F	F	F	T	F	F
YML009C	F	T	F	F	F	F	F	F	F	F	F	F	F	F	F	F	F	F	F	F	F	F	F
YML010W	F	F	F	F	F	F	F	F	F	F	F	F	F	F	F	F	F	F	F	F	F	T	F
YML012W	F	F	F	F	F	F	F	T	F	F	F	F	F	F	F	F	F	F	F	F	F	F	F
YML013W	F	F	F	F	F	F	F	T	F	F	F	F	F	F	F	F	F	F	F	F	F	F	F

YML014W	F	F	F	F	F	F	F	F	F	F	F	F	F	F	F	F	F	T	F	F	F	F	F
YML015C	F	F	F	F	F	F	F	F	F	F	F	F	F	F	F	F	F	F	F	F	F	F	F
YML016C	F	F	F	F	T	F	F	F	F	F	F	F	F	F	F	F	F	T	F	F	F	F	F
YML019W	F	F	F	F	F	F	F	F	F	F	F	F	F	F	F	F	F	F	F	F	F	F	F
YML022W	F	F	F	F	F	F	F	F	F	F	F	F	F	F	F	F	F	T	F	F	F	T	F
YML023C	T	F	F	F	F	F	F	F	F	F	F	F	F	F	F	F	F	F	F	F	F	F	F
YML024W	F	F	F	F	F	F	F	F	F	F	F	F	F	F	F	F	F	T	F	F	F	F	F
YML025C	F	T	F	F	F	F	F	F	F	F	F	F	F	F	F	F	F	F	F	F	F	F	F
YML026C	F	F	F	F	F	F	F	F	F	F	F	F	F	F	F	F	F	T	F	F	F	F	F
YML027W	F	F	F	F	F	F	F	F	F	F	F	F	F	F	F	F	F	T	F	F	F	T	F
YML028W	F	F	F	F	F	T	F	F	F	F	F	F	F	F	F	F	F	T	F	F	F	F	F
YML029W	T	F	F	F	F	F	F	F	F	F	F	F	F	F	F	F	F	F	F	F	F	F	F
YML031W	F	F	F	F	F	F	F	F	T	F	F	F	F	F	F	F	F	F	F	F	F	F	F
YML032C	F	F	F	F	F	F	F	F	F	F	F	F	F	F	F	F	F	F	F	F	F	T	F
YML034W	F	F	F	F	F	F	F	F	T	F	F	F	F	F	F	F	F	F	F	F	F	F	F
YML035C	F	F	F	F	F	F	F	F	F	F	F	F	F	F	F	F	F	T	F	F	F	F	F
YML036W	F	F	F	F	F	F	F	F	F	F	F	F	F	F	F	F	F	F	F	F	F	F	F
YML038C	F	F	F	F	F	F	F	F	F	F	F	F	F	F	F	F	F	F	F	T	F	F	F
YML041C	F	F	F	F	F	F	F	F	F	F	F	F	F	F	F	F	F	F	F	F	F	T	F
YML042W	F	F	F	F	F	F	F	F	F	F	F	F	F	F	F	F	F	F	F	F	F	F	F
YML043C	F	F	F	F	F	F	F	F	F	F	F	T	F	F	F	F	T	F	F	F	F	F	F
YML046W	F	F	F	F	F	F	F	F	F	F	F	F	F	F	F	F	F	F	F	F	F	T	F
YML048W	F	F	F	F	F	F	F	T	F	F	F	F	F	F	F	F	F	F	F	F	F	F	F
YML049C	F	F	F	F	F	F	F	F	F	F	F	F	F	F	F	F	F	T	F	F	F	F	F
YML051W	F	F	F	F	F	F	F	F	F	F	F	F	F	F	F	F	F	T	F	F	F	T	F
YML053C	F	F	F	F	F	F	F	F	F	F	F	F	F	F	F	F	F	T	F	F	F	T	F
YML055W	F	F	F	F	F	F	F	T	F	F	F	F	F	F	F	F	F	F	F	F	F	F	F
YML056C	F	F	F	F	F	F	F	F	F	F	F	F	F	F	F	F	F	T	F	F	F	F	F
YML057W	F	F	F	F	F	F	F	F	F	F	F	F	F	F	F	F	F	T	F	F	F	F	F
YML058W	F	F	F	F	F	F	F	F	F	F	F	F	F	F	F	F	F	T	F	F	F	T	F
YML062C	F	F	F	F	F	F	F	F	F	F	F	F	F	F	F	F	F	F	F	F	F	T	F
YML063W	F	F	F	F	F	F	F	F	F	F	F	F	F	F	F	F	F	T	F	F	F	F	F
YML064C	F	F	F	T	F	T	F	F	F	F	F	F	F	F	F	F	F	F	F	F	F	F	F
YML065W	F	F	F	F	F	F	F	F	F	F	F	F	F	F	F	F	F	F	F	F	F	F	F
YML067C	F	F	F	F	F	F	F	T	F	F	F	F	F	F	F	F	F	F	F	F	F	F	F
YML069W	F	F	F	F	F	F	F	F	F	F	F	F	F	F	F	F	F	F	F	F	F	T	F
YML070W	F	F	F	F	F	F	F	F	F	F	F	F	F	F	F	F	F	T	F	F	F	F	F
YML071C	F	F	F	F	F	T	F	F	F	F	F	F	F	F	F	F	F	F	F	F	F	F	F
YML072C	F	F	F	F	T	F	F	F	F	F	F	F	F	F	F	F	F	F	F	F	F	F	F
YML073C	F	F	F	F	F	F	F	F	F	F	F	F	F	F	F	F	F	T	F	F	F	F	F
YML074C	F	F	F	F	F	F	F	F	F	F	F	F	F	F	F	F	T	F	F	F	F	T	F
YML077W	F	F	F	F	F	F	F	F	F	F	F	F	F	F	F	F	F	F	F	F	F	F	F
YML085C	F	F	F	T	F	T	F	F	F	F	F	T	F	F	F	F	F	T	F	F	F	F	F
YML086C	F	T	F	F	F	F	F	F	F	F	F	F	F	F	F	F	F	F	F	F	F	F	F
YML088W	F	F	F	F	F	F	F	F	F	F	F	F	F	F	F	F	F	T	F	F	F	F	F
YML091C	F	T	F	F	F	F	F	F	F	F	F	F	F	F	F	F	F	F	F	F	F	F	F
YML092C	F	F	F	F	F	F	F	F	F	F	F	F	F	F	F	F	F	F	F	F	F	T	F
YML094W	F	F	F	F	F	F	F	F	F	F	F	F	F	F	F	F	F	T	F	F	F	F	F
YML095C	F	F	F	F	F	F	F	F	F	F	F	F	F	F	F	F	F	T	F	F	F	T	F
YML096W	F	F	F	F	F	F	F	F	F	F	F	F	F	F	F	F	F	T	F	F	F	F	F
YML097C	F	F	F	F	F	F	F	F	F	F	F	F	F	F	F	F	F	T	F	F	F	F	F
YML098W	F	F	F	F	F	F	F	F	F	F	F	F	F	F	F	F	F	T	F	F	F	T	F
YML099C	F	F	F	F	F	F	F	F	F	F	F	F	F	F	F	F	F	T	F	F	F	T	F
YML100W	F	F	F	F	F	F	F	F	F	F	F	F	F	F	F	F	F	T	F	F	F	F	F
YML101C	F	F	F	F	F	F	F	T	F	F	F	F	F	F	F	F	F	F	F	F	F	F	F
YML102W	F	F	F	F	F	F	F	F	F	F	F	F	F	F	F	F	F	F	F	F	F	T	F
YML103C	F	F	F	F	F	F	F	F	T	F	F	F	F	F	F	F	F	F	F	F	F	F	F
YML105C	F	F	F	F	F	F	F	T	F	F	F	F	F	F	F	F	F	F	F	F	F	F	F
YML106W	F	F	F	F	F	F	F	F	F	F	F	F	F	F	F	F	F	T	F	F	F	T	F
YML109W	F	F	F	F	F	F	F	F	F	F	F	F	F	F	F	F	F	T	F	F	F	F	F
YML112W	F	F	F	F	F	F	F	F	F	F	F	F	F	F	F	F	F	T	F	F	F	T	F
YML114C	F	F	F	F	F	F	F	F	F	F	F	F	F	F	F	F	F	F	F	F	F	T	F
YML115C	F	F	T	F	F	F	F	F	F	F	F	F	T	F	F	F	F	F	F	F	F	F	F
YML117W	F	F	F	F	F	F	F	F	F	F	F	F	F	F	F	F	F	T	F	F	F	F	F
YML121W	F	F	F	F	F	F	T	F	F	F	F	F	F	F	F	F	F	F	F	F	F	F	F
YML123C	F	F	F	F	F	F	F	F	F	F	F	F	F	F	F	F	F	F	F	F	F	F	F
YML124C	F	F	F	T	F	F	F	F	F	F	F	F	F	F	F	F	F	T	F	F	F	T	F
YML126C	F	F	F	F	F	F	F	F	F	F	F	F	F	F	F	F	F	F	F	F	F	T	F

YML127W	F	F	F	F	F	F	F	F	F	F	F	F	F	F	F	F	F	F	F	F	F	T	F
YML129C	F	T	F	F	F	F	F	F	F	F	F	F	F	F	F	F	F	F	F	F	F	F	F
YML130C	F	F	F	F	F	F	F	F	F	F	F	F	F	F	F	F	F	F	F	F	F	F	F
YML132W	F	F	T	F	F	F	F	F	F	F	F	F	F	F	F	F	F	F	F	F	F	F	F
YMR001C	F	F	F	F	F	F	F	F	F	F	T	F	F	F	F	F	F	F	F	F	F	T	F
YMR005W	F	F	F	F	F	F	F	F	F	F	F	F	F	F	F	F	F	F	F	F	F	F	F
YMR012W	F	F	F	F	F	F	F	F	F	F	F	F	F	F	F	F	F	T	F	F	F	F	F
YMR014W	F	F	F	F	F	F	F	F	F	F	F	F	F	F	F	F	T	F	F	F	F	F	F
YMR015C	F	F	F	F	F	F	F	T	F	F	F	F	F	F	F	F	F	F	F	F	F	F	F
YMR017W	F	F	F	F	F	F	F	F	F	F	F	F	F	F	F	F	F	F	F	F	F	F	F
YMR021C	F	F	F	F	F	F	F	F	F	F	F	F	F	F	F	F	F	T	F	F	F	T	F
YMR022W	F	F	F	F	F	F	F	T	F	F	F	F	F	F	F	F	F	F	F	F	F	F	F
YMR024W	F	T	F	F	F	F	F	F	F	F	F	F	F	F	F	F	F	F	F	F	F	F	F
YMR025W	F	F	F	F	F	F	F	F	F	F	F	F	F	F	F	F	F	F	F	F	F	F	F
YMR026C	F	F	F	F	F	F	F	F	F	F	F	F	F	F	T	F	F	F	F	F	F	F	F
YMR027W	F	F	F	F	F	F	F	F	F	F	F	F	F	F	F	F	F	T	F	F	F	T	F
YMR028W	F	F	F	F	F	F	F	F	F	F	F	F	F	F	F	F	F	F	F	F	F	F	F
YMR029C	T	F	F	F	F	F	F	T	F	F	F	F	F	F	F	F	F	T	F	F	F	F	F
YMR031C	F	F	F	F	F	T	F	F	F	F	F	F	F	F	F	F	F	F	F	F	F	F	F
YMR032W	F	F	F	F	F	F	F	F	F	F	T	F	F	F	F	F	F	F	F	F	F	F	F
YMR033W	F	F	F	F	F	F	F	F	F	F	F	F	F	F	F	F	F	F	F	F	F	F	F
YMR036C	F	F	F	F	F	F	F	F	F	F	F	F	F	F	F	F	F	T	F	F	F	T	F
YMR038C	F	F	F	F	F	F	F	F	F	F	F	F	F	F	F	F	F	T	F	F	F	T	F
YMR039C	F	F	F	F	F	F	F	F	F	F	F	F	F	F	F	F	F	F	F	F	F	T	F
YMR042W	F	F	F	F	F	F	F	F	F	F	F	F	F	F	F	F	F	F	F	F	F	T	F
YMR043W	F	F	F	F	F	F	F	F	F	F	F	F	F	F	F	F	F	F	F	F	F	T	F
YMR044W	F	F	F	F	F	F	F	F	F	F	F	F	F	F	F	F	F	F	F	F	F	T	F
YMR047C	F	F	F	F	F	F	F	F	F	F	F	F	F	F	F	F	F	T	F	F	F	T	F
YMR048W	F	F	F	F	F	F	F	F	F	F	F	F	F	F	F	F	F	F	F	F	F	T	F
YMR049C	F	F	F	F	F	F	F	F	F	F	F	F	F	F	F	F	T	F	F	F	F	T	F
YMR052W	F	F	F	F	F	F	F	T	F	F	F	F	F	F	F	F	F	F	F	F	F	F	F
YMR054W	F	F	F	F	F	F	F	F	F	F	F	F	F	T	F	F	F	F	F	T	F	F	F
YMR055C	F	F	F	T	F	F	F	F	F	F	F	F	F	F	F	F	F	F	F	F	F	F	F
YMR060C	F	T	F	F	F	F	F	F	F	F	F	F	F	F	F	F	F	F	F	F	F	F	F
YMR061W	F	F	F	F	F	F	F	F	F	F	F	F	F	F	F	F	F	F	F	F	F	T	F
YMR064W	F	T	F	F	F	F	F	F	F	F	F	F	F	F	F	F	F	F	F	F	F	F	F
YMR066W	F	T	F	F	F	F	F	F	F	F	F	F	F	F	F	F	F	F	F	F	F	F	F
YMR067C	F	F	F	F	F	F	F	F	F	F	F	F	F	F	F	F	F	T	F	F	F	T	F
YMR068W	F	F	F	F	F	F	F	F	F	F	F	F	F	F	F	F	F	F	F	F	F	F	F
YMR071C	F	F	F	F	F	F	F	F	F	F	F	F	F	T	F	F	F	F	F	F	F	F	F
YMR072W	F	T	F	F	F	F	F	F	F	F	F	F	F	F	F	F	F	F	F	F	F	F	F
YMR075W	F	F	F	F	F	F	F	F	F	F	F	F	F	F	F	F	F	F	F	F	F	T	F
YMR076C	F	F	F	F	F	F	F	F	F	F	F	F	F	F	F	F	F	F	F	F	F	T	F
YMR077C	F	F	F	F	F	F	T	F	F	T	F	F	F	F	F	F	F	F	F	F	F	F	F
YMR078C	F	F	F	F	F	F	F	F	F	F	F	F	F	F	F	F	F	T	F	F	F	T	F
YMR079W	F	F	F	F	F	F	F	F	F	F	F	F	F	F	F	F	F	T	F	F	F	T	F
YMR080C	F	F	F	F	F	T	F	F	F	F	F	F	F	F	F	F	F	T	F	F	F	F	F
YMR086W	F	F	F	F	T	F	F	F	F	F	F	F	F	F	F	F	F	F	F	F	F	F	F
YMR089C	F	T	F	F	F	F	F	F	F	F	F	F	F	F	F	F	F	F	F	F	F	F	F
YMR091C	F	F	F	F	F	F	F	F	F	F	F	F	F	F	F	F	F	F	F	F	F	T	F
YMR092C	F	F	F	F	F	F	F	F	F	F	F	F	F	F	F	T	F	F	F	F	F	F	F
YMR093W	F	F	F	F	F	F	F	F	F	F	F	F	F	F	F	F	T	F	F	F	F	T	F
YMR094W	F	F	F	F	F	F	F	F	F	F	F	F	F	F	F	F	F	F	F	F	F	F	F
YMR095C	F	F	F	F	F	F	F	F	F	F	F	F	F	F	F	F	F	T	F	F	F	F	F
YMR096W	F	F	F	F	F	F	F	F	F	F	F	F	F	F	F	F	F	F	F	F	F	F	F
YMR099C	F	F	F	F	F	F	F	F	F	F	F	F	F	F	F	F	F	T	F	F	F	T	F
YMR100W	F	F	F	F	F	F	F	F	F	F	F	F	F	F	F	F	F	F	F	F	F	F	F
YMR104C	F	F	F	F	F	F	F	F	F	F	F	F	F	F	F	F	F	T	F	F	F	T	F
YMR106C	F	F	F	F	F	F	F	F	F	F	F	F	F	F	F	F	F	F	F	F	F	T	F
YMR109W	F	F	F	F	F	F	F	F	F	F	F	F	F	F	F	T	F	F	F	F	F	F	F
YMR111C	F	F	F	F	F	F	F	F	F	F	F	F	F	F	F	F	F	F	F	F	F	T	F
YMR112C	F	F	F	F	F	F	F	F	F	F	F	F	F	F	F	F	F	F	F	F	F	T	F
YMR116C	F	F	F	F	F	F	F	F	F	F	F	F	F	F	F	F	F	F	F	F	F	F	F
YMR117C	F	F	F	T	F	F	F	F	F	F	F	F	F	F	F	F	F	F	F	F	F	F	F
YMR120C	F	F	F	F	F	F	F	F	F	F	F	F	F	F	F	F	F	T	F	F	F	F	F
YMR121C	F	F	F	F	F	F	F	F	F	F	F	F	F	F	F	F	T	F	F	F	F	T	F
YMR124W	T	F	F	F	T	F	F	F	F	F	T	F	F	F	F	F	F	T	F	F	F	F	T
YMR125W	F	F	F	F	F	F	F	F	F	F	F	F	F	F	F	F	F	F	F	F	F	T	F

YMR127C	F	F	F	F	F	F	F	F	F	F	F	F	F	F	F	F	F	T	F	F	F	T	F
YMR128W	F	F	F	F	F	F	F	F	F	F	F	F	F	F	F	F	T	F	F	F	F	F	F
YMR129W	F	F	F	F	F	F	F	F	T	F	F	F	F	F	F	F	F	F	F	F	F	F	F
YMR131C	F	F	F	F	F	F	F	F	F	F	F	F	F	F	F	F	T	F	F	F	F	T	F
YMR135C	F	F	F	F	F	F	F	F	F	F	F	F	F	F	F	F	F	T	F	F	F	T	F
YMR139W	F	F	F	F	F	F	F	F	F	F	F	F	F	F	F	F	F	T	F	F	F	F	F
YMR140W	F	F	F	F	F	F	F	F	F	F	F	F	F	F	F	F	F	T	F	F	F	F	F
YMR142C	F	F	F	F	F	F	F	F	F	F	F	F	F	F	F	F	F	T	F	F	F	F	F
YMR143W	F	F	F	F	F	F	F	F	F	F	F	F	F	F	F	F	F	T	F	F	F	F	F
YMR146C	F	F	F	F	F	F	F	F	F	F	F	F	F	F	F	F	F	F	F	F	F	F	F
YMR149W	F	F	F	F	F	F	F	T	F	F	F	F	F	F	F	F	F	F	F	F	F	F	F
YMR153W	F	F	F	F	F	F	F	F	T	F	F	F	F	F	F	F	F	F	F	F	F	F	F
YMR154C	F	F	F	F	F	F	F	F	F	F	F	F	F	F	F	F	F	F	F	F	F	F	F
YMR158W	F	F	F	F	F	F	F	F	F	F	F	F	F	F	F	F	F	F	F	F	F	F	F
YMR159C	T	F	F	F	F	F	F	F	F	F	F	F	F	F	F	F	F	F	F	F	F	F	F
YMR163C	T	F	F	F	F	T	F	F	F	F	F	F	F	F	F	F	F	F	F	F	F	F	T
YMR165C	F	F	F	F	F	F	F	F	F	F	F	F	F	F	F	F	F	T	F	F	F	F	F
YMR167W	F	F	F	F	F	F	F	F	F	F	F	F	F	F	F	F	F	T	F	F	F	T	F
YMR168C	F	F	F	F	F	F	F	F	F	F	F	T	F	F	F	F	F	F	F	F	F	F	F
YMR171C	F	F	F	F	F	F	F	F	F	T	F	F	F	F	F	F	F	F	F	F	F	F	F
YMR172W	F	F	F	F	F	F	F	F	F	F	F	F	F	F	F	F	F	F	F	F	F	T	F
YMR176W	F	F	F	F	F	F	F	F	F	F	F	F	F	F	F	F	F	F	F	F	F	T	F
YMR179W	F	F	F	F	F	F	F	F	F	F	F	F	F	F	F	F	F	F	F	F	F	T	F
YMR180C	F	F	F	F	F	F	F	F	F	F	F	F	F	F	F	F	F	F	F	F	F	F	F
YMR183C	F	F	F	F	F	T	F	F	F	T	F	F	F	F	F	F	F	F	F	F	F	F	F
YMR184W	F	F	F	F	F	F	F	F	F	F	F	F	F	F	F	F	F	T	F	F	F	F	F
YMR186W	F	F	F	F	F	F	F	F	F	F	F	F	F	F	F	F	F	T	F	F	F	F	F
YMR188C	F	T	F	F	F	F	F	F	F	F	F	F	F	F	F	F	F	F	F	F	F	F	F
YMR190C	F	F	F	F	F	F	F	F	F	F	F	F	F	F	F	F	F	T	F	F	F	T	F
YMR191W	F	F	F	F	F	F	F	F	F	F	F	F	F	F	F	F	F	F	F	F	F	F	F
YMR192W	T	F	F	F	F	F	F	F	F	F	T	F	F	F	F	F	F	T	F	F	F	F	T
YMR193W	F	F	F	F	F	F	F	F	F	F	F	F	F	F	F	F	T	F	F	F	F	F	F
YMR194W	F	F	F	F	F	F	F	F	F	F	F	F	F	F	F	F	F	T	F	F	F	F	F
YMR196W	F	F	F	F	F	F	F	F	F	F	F	F	F	F	F	F	F	T	F	F	F	F	F
YMR197C	F	F	F	F	F	F	F	F	F	F	F	F	F	F	F	F	F	F	F	F	F	F	F
YMR198W	F	F	F	F	F	F	F	F	F	F	F	T	F	F	F	F	F	F	F	F	F	F	F
YMR199W	F	F	F	F	F	F	F	F	F	F	F	F	F	F	F	F	F	T	F	F	F	T	F
YMR200W	F	F	F	F	F	F	F	T	F	F	F	F	F	F	F	F	F	F	F	F	F	F	F
YMR201C	F	F	F	F	F	F	F	F	F	F	F	F	F	F	F	F	F	T	F	F	F	T	F
YMR202W	T	F	F	F	F	F	F	F	F	F	F	F	F	F	F	F	F	F	F	F	F	F	F
YMR203W	F	F	F	F	F	F	F	F	F	F	F	F	F	F	F	F	F	F	F	F	F	F	F
YMR204C	F	F	F	F	F	F	F	F	F	F	F	F	F	F	T	F	F	F	F	F	F	F	F
YMR205C	F	F	F	F	F	F	F	F	F	F	F	F	F	F	F	F	F	T	F	F	F	F	F
YMR210W	F	F	F	F	F	F	F	F	F	F	F	F	F	F	F	F	F	F	F	F	F	F	F
YMR212C	F	F	F	F	T	F	F	F	F	F	F	F	F	F	F	F	F	F	F	F	F	F	F
YMR213W	F	F	F	F	F	F	F	F	F	F	F	F	F	F	F	F	F	T	F	F	F	T	F
YMR214W	T	F	F	F	F	F	F	F	F	F	F	F	F	F	F	F	F	F	F	F	F	F	F
YMR216C	F	F	F	F	F	F	F	F	F	F	F	F	F	F	F	F	F	T	F	F	F	F	F
YMR217W	F	F	F	F	F	F	F	F	F	F	F	F	F	F	F	F	F	F	F	F	F	F	F
YMR218C	F	F	F	F	F	F	F	F	F	F	F	F	F	T	F	F	F	F	F	F	F	F	F
YMR219W	F	F	F	F	F	F	F	F	T	F	F	F	F	F	F	F	F	F	F	F	F	F	F
YMR223W	F	F	F	F	F	F	F	F	F	F	F	F	F	F	F	F	F	F	F	F	F	T	F
YMR224C	F	F	F	F	F	F	F	F	F	F	F	F	F	F	F	F	F	T	F	F	F	T	F
YMR225C	F	T	F	F	F	F	F	F	F	F	F	F	F	F	F	F	F	F	F	F	F	F	F
YMR226C	F	F	F	F	F	F	F	F	F	F	F	F	F	F	F	F	F	T	F	F	F	T	F
YMR227C	F	F	F	F	F	F	F	F	F	F	F	F	F	F	F	F	F	T	F	F	F	T	F
YMR228W	F	F	F	F	F	F	F	F	F	F	F	F	F	F	F	F	F	F	F	F	F	F	F
YMR229C	F	F	F	F	F	F	F	F	F	F	F	F	F	F	F	F	T	F	F	F	F	F	F
YMR231W	F	F	F	F	F	F	F	F	F	T	F	F	F	F	F	F	F	F	F	F	F	F	F
YMR232W	F	F	F	F	F	F	F	F	F	F	F	F	F	F	F	F	F	T	F	F	F	F	F
YMR233W	F	F	F	F	F	F	F	F	F	F	F	F	F	F	F	F	T	T	F	F	F	T	F
YMR235C	F	F	F	F	F	F	F	F	F	F	F	F	F	F	F	F	F	T	F	F	F	F	F
YMR236W	F	F	F	F	F	F	F	F	F	F	F	F	F	F	F	F	F	F	F	F	F	T	F
YMR237W	F	F	F	F	F	F	F	F	F	F	F	F	F	T	F	F	F	F	F	F	F	F	F
YMR239C	F	F	F	F	F	F	F	F	F	F	F	F	F	F	F	F	T	F	F	F	F	T	F
YMR240C	F	F	F	F	F	F	F	F	F	F	F	F	F	F	F	F	F	F	F	F	F	T	F
YMR242C	F	F	F	F	F	F	F	F	F	F	F	F	F	F	F	F	F	T	F	F	F	F	F
YMR243C	F	F	F	F	F	F	T	F	F	F	F	F	F	F	F	F	F	F	F	F	F	F	F

YMR246W	F	F	F	F	F	F	F	F	F	F	F	F	F	F	F	F	F	F	F	F	T	F	F
YMR255W	F	F	F	F	F	F	F	F	F	F	F	F	F	F	F	F	F	T	F	F	F	F	F
YMR258C	F	F	F	F	F	F	F	F	F	F	F	F	F	F	F	F	F	T	F	F	F	T	F
YMR259C	F	F	F	F	F	F	F	F	F	F	F	F	F	F	F	F	F	T	F	F	F	F	F
YMR260C	F	F	F	F	F	F	F	F	F	F	F	F	F	F	F	F	F	T	F	F	F	F	F
YMR261C	F	F	F	F	F	F	F	F	F	F	F	F	F	F	F	F	F	T	F	F	F	F	F
YMR263W	F	F	F	F	F	F	F	F	F	F	F	F	F	F	F	F	F	F	F	F	F	T	F
YMR264W	F	F	F	F	F	F	F	T	F	F	F	F	F	F	F	F	F	T	F	F	F	F	F
YMR267W	F	T	F	F	F	F	F	F	F	F	F	F	F	F	F	F	F	F	F	F	F	F	F
YMR268C	F	F	F	F	F	F	F	F	F	F	F	F	F	F	F	F	F	T	F	F	F	T	F
YMR270C	F	F	F	F	F	F	F	F	F	F	F	F	F	F	F	F	T	F	F	F	F	F	F
YMR273C	F	F	F	F	F	F	F	F	F	F	F	F	F	F	F	F	F	T	F	F	F	F	F
YMR275C	F	F	F	F	F	F	F	F	F	F	F	F	F	F	F	F	F	T	F	F	F	F	F
YMR276W	F	F	F	F	F	F	F	F	F	F	F	F	F	F	F	F	F	T	F	F	F	T	F
YMR277W	F	F	F	F	F	F	F	F	F	F	F	F	F	F	F	F	F	F	F	F	F	T	F
YMR280C	F	F	F	F	F	F	F	F	F	F	F	F	F	F	F	F	F	T	F	F	F	T	F
YMR284W	F	F	F	F	F	F	F	F	F	F	F	F	F	F	F	F	F	T	F	F	F	T	F
YMR285C	F	F	F	F	F	F	F	F	F	F	F	F	F	F	F	F	F	T	F	F	F	F	F
YMR287C	F	T	F	F	F	F	F	F	F	F	F	F	F	F	F	F	F	F	F	F	F	F	F
YMR288W	F	F	F	F	F	F	F	F	F	F	F	F	F	F	F	F	F	T	F	F	F	T	F
YMR290C	F	F	F	F	F	F	F	F	F	F	F	F	F	F	F	F	F	F	F	F	F	F	F
YMR294W	F	F	F	F	F	F	F	F	F	F	F	F	F	F	F	F	F	F	F	F	F	F	F
YMR296C	F	F	F	F	F	F	F	T	F	F	F	F	F	F	F	F	F	F	F	F	F	F	F
YMR297W	F	F	T	F	F	F	F	F	F	F	F	F	F	F	F	F	F	F	F	F	F	F	F
YMR298W	F	F	F	F	F	F	F	F	F	F	F	F	F	F	F	F	F	F	F	F	F	F	F
YMR304W	F	F	F	F	F	F	F	F	F	F	F	F	F	F	F	F	F	T	F	F	F	F	F
YMR307W	F	T	F	F	F	F	F	T	T	F	F	F	F	F	F	F	F	F	F	F	F	F	F
YMR308C	F	F	F	F	F	F	F	F	F	F	F	F	F	F	F	F	F	T	F	F	F	T	F
YMR309C	F	F	F	F	F	F	F	F	F	F	F	F	F	F	F	F	F	T	F	F	F	F	F
YMR310C	F	F	F	F	F	F	F	F	F	F	F	F	F	F	F	F	F	F	F	F	F	T	F
YMR311C	F	F	F	F	F	F	F	F	F	F	F	F	F	F	F	F	F	T	F	F	F	T	F
YMR312W	F	F	F	F	F	F	F	F	F	F	F	F	F	F	F	F	F	F	F	F	F	F	F
YMR314W	F	F	F	F	F	F	F	F	F	F	F	F	F	F	F	F	F	T	F	F	F	T	F
YMR315W	F	F	F	F	F	F	F	F	F	F	F	F	F	F	F	F	F	T	F	F	F	T	F
YMR316W	F	F	F	F	F	F	F	F	F	F	F	F	F	F	F	F	F	F	F	F	F	F	F
YMR318C	F	F	F	F	F	F	F	F	F	F	F	F	F	F	F	F	F	T	F	F	F	T	F
YNL002C	F	F	F	F	F	F	F	F	F	F	F	F	F	F	F	F	F	F	F	F	F	F	F
YNL004W	F	F	F	F	F	F	F	F	F	F	F	F	F	F	F	F	F	F	F	F	F	T	F
YNL005C	F	T	F	F	F	F	F	F	F	F	F	F	F	F	F	F	F	F	F	F	F	F	F
YNL006W	F	F	F	F	F	T	F	F	F	F	F	F	F	F	F	F	F	F	F	F	F	F	F
YNL007C	F	F	F	F	F	F	F	F	F	F	F	F	F	F	F	F	F	T	F	F	F	T	F
YNL010W	F	F	F	F	F	F	F	F	F	F	F	F	F	F	F	F	F	T	F	F	F	T	F
YNL014W	F	F	F	F	F	F	F	F	F	F	F	F	F	F	F	F	F	F	F	F	F	F	F
YNL016W	F	F	F	F	F	F	F	F	F	F	F	F	F	F	F	F	F	T	F	F	F	F	F
YNL020C	F	F	F	F	F	F	F	F	F	F	F	F	F	F	F	T	F	F	F	F	F	F	F
YNL021W	F	F	F	F	F	F	F	F	F	F	F	F	F	F	F	F	F	F	F	F	F	T	F
YNL023C	F	F	F	F	F	F	F	F	F	F	F	F	F	F	F	F	F	F	F	F	F	F	F
YNL025C	F	F	F	F	F	F	F	F	F	F	F	F	F	F	F	F	F	F	F	F	F	T	F
YNL026W	F	F	F	F	F	F	F	F	F	F	F	F	F	F	F	F	F	F	F	F	F	F	F
YNL027W	F	F	F	F	F	F	F	F	F	F	F	F	F	F	F	F	F	T	F	F	F	F	F
YNL030W	F	F	F	F	F	F	F	F	F	F	F	F	F	F	F	F	F	F	F	F	F	T	F
YNL031C	T	F	F	F	F	F	F	F	F	F	F	F	F	F	F	F	F	F	F	F	F	F	F
YNL032W	F	F	F	F	F	F	F	F	F	F	F	F	F	F	F	F	F	T	F	F	F	F	F
YNL037C	F	T	F	F	F	F	F	F	F	F	F	F	F	F	F	F	F	F	F	F	F	F	F
YNL039W	F	F	F	F	F	F	F	F	F	F	F	F	F	F	F	F	F	F	F	F	F	T	F
YNL041C	F	F	F	F	F	F	F	F	F	F	F	F	T	F	F	F	F	F	F	F	F	F	F
YNL042W	F	F	F	F	F	F	F	F	F	F	F	F	F	F	F	F	F	T	F	F	F	T	F
YNL044W	F	F	F	F	F	F	F	F	F	F	F	F	T	F	F	F	F	F	F	T	F	F	F
YNL047C	F	F	F	F	F	F	F	F	F	F	F	F	F	F	F	F	F	F	F	F	F	F	F
YNL049C	F	F	F	F	F	F	F	F	F	F	F	F	F	F	F	F	F	F	T	F	F	F	F
YNL050C	F	F	F	F	F	F	F	F	F	F	F	F	F	F	F	F	F	F	F	F	F	F	F
YNL051W	F	F	F	F	F	F	F	F	F	F	F	F	T	F	F	F	F	F	F	T	F	F	F
YNL052W	F	T	F	F	F	F	F	F	F	F	F	F	F	F	F	F	F	F	F	F	F	F	F
YNL053W	F	F	F	F	F	F	F	F	F	F	F	F	F	F	F	F	F	T	F	F	F	F	F
YNL054W	F	F	F	F	F	F	F	F	F	F	F	F	F	F	F	F	F	F	F	F	F	F	F
YNL055C	F	T	F	F	F	F	F	F	F	F	F	F	F	F	F	F	F	T	F	F	F	F	F
YNL056W	F	F	F	F	F	F	F	F	F	F	F	F	F	F	F	F	F	T	F	F	F	F	F
YNL059C	F	F	F	F	F	F	F	F	F	F	F	F	F	F	F	F	F	T	F	F	F	T	F

YNL061W	F	F	F	F	F	F	F	F	F	F	F	F	F	F	F	F	T	F	F	F	F	T	F
YNL062C	F	F	F	F	F	F	F	F	F	F	F	F	F	F	F	F	F	F	F	F	F	T	F
YNL064C	F	F	F	F	F	F	F	F	F	F	F	F	F	F	F	F	F	T	F	F	F	T	F
YNL067W	F	F	F	F	F	F	F	F	F	F	F	F	F	F	F	F	F	T	F	F	F	F	F
YNL068C	F	F	F	F	F	F	F	F	F	F	F	F	F	F	F	F	F	F	F	F	F	T	F
YNL069C	F	F	F	F	F	F	F	F	F	F	F	F	F	F	F	F	F	F	F	F	F	F	F
YNL071W	F	T	F	F	F	F	F	F	F	F	F	F	F	F	F	F	F	F	F	F	F	F	F
YNL073W	F	T	F	F	F	F	F	F	F	F	F	F	F	F	F	F	F	F	F	F	F	F	F
YNL075W	F	F	F	F	F	F	F	F	F	F	F	F	F	F	F	F	F	F	F	F	F	F	F
YNL076W	F	F	F	F	F	F	F	F	F	F	F	F	F	F	F	F	F	T	F	F	F	F	F
YNL078W	F	F	F	F	T	F	F	F	F	F	T	F	F	F	F	F	F	F	F	F	F	F	F
YNL079C	F	F	F	F	F	F	F	F	F	F	F	F	F	F	F	F	F	F	F	F	F	F	F
YNL081C	F	T	F	F	F	F	F	F	F	F	F	F	F	F	F	F	F	F	F	F	F	F	F
YNL082W	F	F	F	F	F	F	F	F	F	F	F	F	F	F	F	F	F	T	F	F	F	T	F
YNL084C	F	F	F	F	F	T	F	F	F	F	F	F	F	F	F	F	F	F	F	F	F	F	F
YNL085W	F	F	F	F	F	T	F	F	F	F	F	F	F	F	F	F	F	T	F	F	F	F	F
YNL086W	F	F	F	F	F	F	F	F	F	T	F	F	F	F	F	F	F	F	F	F	F	F	F
YNL088W	F	F	F	F	F	F	F	F	F	F	F	F	F	F	F	F	F	F	F	F	F	T	F
YNL090W	F	F	F	F	F	F	F	F	F	F	F	F	F	F	F	F	F	T	F	F	F	T	F
YNL091W	F	F	F	F	F	F	F	F	F	F	F	F	F	F	F	F	F	T	F	F	F	F	F
YNL093W	F	F	F	F	F	F	F	F	F	F	F	F	F	F	F	F	F	F	F	F	F	F	F
YNL094W	F	F	F	F	F	F	F	F	F	F	F	F	F	F	F	F	F	T	F	F	F	F	F
YNL097C	F	F	F	F	F	F	F	F	F	F	F	F	F	F	F	F	F	F	F	F	F	T	F
YNL098C	F	F	F	F	F	F	F	F	F	F	F	F	F	F	F	F	F	T	F	F	F	T	F
YNL099C	F	F	F	F	F	F	F	F	F	F	F	F	F	F	F	F	F	T	F	F	F	F	F
YNL101W	F	F	F	F	F	F	F	F	F	F	F	F	F	F	F	F	F	F	F	F	F	F	F
YNL102W	F	F	F	F	F	F	F	F	F	F	F	F	F	F	F	F	F	F	F	F	F	T	F
YNL103W	F	F	F	F	F	F	F	F	F	F	F	F	F	F	F	F	F	F	F	F	F	T	F
YNL104C	F	T	F	F	F	F	F	F	F	F	F	F	F	F	F	F	F	T	F	F	F	F	F
YNL106C	F	F	F	F	F	F	F	F	F	F	F	F	F	F	F	T	F	F	F	F	F	F	F
YNL107W	F	F	F	F	F	F	F	F	F	F	F	F	F	F	F	F	F	T	F	F	F	T	F
YNL110C	F	F	F	F	F	F	F	F	F	F	F	F	F	F	F	F	T	F	F	F	F	T	F
YNL112W	F	F	F	F	F	F	F	F	F	F	F	F	F	F	F	F	F	T	F	F	F	T	F
YNL113W	F	F	F	F	F	F	F	F	F	F	F	F	F	F	F	F	T	F	F	F	F	F	F
YNL116W	F	F	F	F	F	F	F	F	F	F	F	F	F	F	F	F	F	T	F	F	F	F	F
YNL118C	F	F	F	F	F	T	F	F	F	F	F	F	F	F	F	F	F	F	F	F	F	F	F
YNL119W	F	F	F	F	F	F	F	F	F	F	F	F	F	F	F	F	F	T	F	F	F	F	F
YNL121C	F	T	F	F	F	F	F	F	F	F	F	F	F	F	F	F	F	F	F	F	F	F	F
YNL123W	F	F	F	F	F	F	F	F	F	F	F	F	F	F	F	F	F	F	F	F	F	T	F
YNL124W	F	F	F	F	F	F	F	F	F	F	F	F	F	F	F	F	F	F	F	F	F	T	F
YNL126W	F	F	F	T	F	T	F	F	F	F	F	F	F	F	F	F	F	F	F	F	F	F	F
YNL127W	T	F	F	F	F	F	F	F	F	F	F	F	F	F	F	F	F	F	F	F	F	F	F
YNL131W	T	F	F	F	F	F	F	F	F	F	F	F	F	F	F	F	F	F	F	F	F	F	F
YNL132W	F	F	F	F	F	F	F	F	F	F	F	F	F	F	F	F	T	F	F	F	F	F	F
YNL135C	F	F	F	F	F	F	F	F	F	F	F	F	F	F	F	F	F	T	F	F	F	T	F
YNL136W	F	F	F	F	F	F	F	F	F	F	F	F	F	F	F	F	F	F	F	F	F	T	F
YNL137C	F	T	F	F	F	F	F	F	F	F	F	F	F	F	F	F	F	F	F	F	F	F	F
YNL138W	F	F	F	F	F	F	F	F	F	F	F	F	F	F	F	T	F	F	F	F	F	F	F
YNL139C	F	F	F	F	F	F	F	F	F	F	F	F	F	F	F	F	F	F	F	F	F	T	F
YNL141W	F	F	F	F	F	F	F	F	F	F	F	F	F	F	F	F	F	T	F	F	F	T	F
YNL145W	F	F	F	F	F	F	F	F	F	F	F	F	F	F	F	F	F	F	F	F	F	F	F
YNL147W	F	F	F	F	F	F	F	F	F	F	F	F	F	F	F	F	F	T	F	F	F	F	F
YNL148C	T	F	F	F	F	F	F	F	F	F	F	F	F	F	F	F	F	F	F	F	F	F	F
YNL151C	F	F	F	F	F	F	F	F	F	F	F	F	F	F	F	F	F	F	F	F	F	F	F
YNL152W	F	F	F	F	F	F	F	F	F	F	F	F	F	F	F	F	F	T	F	F	F	F	F
YNL153C	F	F	F	F	F	F	F	F	F	F	F	F	F	F	F	F	F	T	F	F	F	F	F
YNL154C	F	F	F	F	F	F	F	F	F	F	F	F	F	F	F	F	F	T	F	F	F	T	F
YNL155W	F	F	F	F	F	F	F	F	F	F	F	F	F	F	F	F	F	T	F	F	F	T	F
YNL157W	F	F	F	F	F	F	F	F	F	F	F	F	F	F	F	F	F	T	F	F	F	T	F
YNL161W	F	F	F	F	F	F	F	F	F	F	T	F	F	F	F	F	F	T	F	F	F	F	F
YNL166C	T	F	F	F	F	F	F	F	F	F	T	F	F	F	F	F	F	T	F	F	F	F	T
YNL167C	F	F	F	F	F	F	F	F	F	F	F	F	F	F	F	F	F	T	F	F	F	T	F
YNL172W	F	F	F	T	F	F	F	F	F	F	F	F	F	F	F	F	F	T	F	F	F	T	F
YNL175C	F	F	F	F	F	F	F	F	F	F	F	F	F	F	F	F	T	F	F	F	F	T	F
YNL177C	F	T	F	F	F	F	F	F	F	F	F	F	F	F	F	F	F	F	F	F	F	F	F
YNL178W	F	F	F	F	F	F	F	F	F	F	F	F	F	F	F	F	F	F	F	F	F	F	F
YNL182C	F	F	F	F	F	F	F	F	F	F	F	F	F	F	F	F	F	F	F	F	F	F	F
YNL183C	F	F	F	F	F	F	F	F	F	F	F	F	F	F	F	F	F	T	F	F	F	F	F

YNL186W	F	F	F	F	F	F	F	F	F	F	F	F	F	F	F	F	F	T	F	F	F	T	F
YNL188W	F	F	F	F	F	F	F	F	F	F	F	F	F	F	F	F	F	F	F	F	F	F	F
YNL189W	F	F	F	F	F	F	F	F	T	F	F	F	F	F	F	F	F	T	F	F	F	F	F
YNL191W	F	F	F	F	F	F	F	F	F	F	F	F	F	F	F	F	F	T	F	F	F	F	F
YNL197C	F	F	F	F	F	F	F	F	F	F	F	F	F	F	F	F	F	T	F	F	F	F	F
YNL199C	F	F	F	F	F	F	F	F	F	F	F	F	F	F	F	F	F	F	F	F	F	F	F
YNL201C	F	F	F	F	F	F	F	F	F	F	F	F	F	F	F	F	F	F	F	F	F	T	F
YNL206C	F	F	F	F	F	F	F	F	F	F	F	F	F	F	F	F	F	F	F	F	F	T	F
YNL207W	F	F	F	F	F	F	F	F	F	F	F	F	F	F	F	F	F	T	F	F	F	F	F
YNL209W	F	F	F	F	F	F	F	F	F	F	F	F	F	F	F	F	F	T	F	F	F	F	F
YNL210W	F	F	F	F	F	F	F	F	F	F	F	F	F	F	F	F	F	F	F	F	F	F	F
YNL212W	F	F	F	F	F	F	F	F	F	F	F	F	F	F	F	F	F	T	F	F	F	F	F
YNL214W	F	F	F	F	F	F	F	F	F	F	F	F	F	F	T	F	F	F	F	F	F	F	F
YNL215W	F	F	F	F	F	F	F	F	F	F	F	F	F	F	F	F	F	F	F	F	F	T	F
YNL216W	F	F	F	F	F	F	F	F	F	F	F	F	F	F	F	F	F	F	F	F	F	T	F
YNL218W	F	F	F	F	F	F	F	F	F	F	F	F	F	F	F	F	F	T	F	F	F	T	F
YNL220W	F	F	F	F	F	F	F	F	F	F	F	F	F	F	F	F	F	T	F	F	F	F	F
YNL221C	F	F	F	F	F	F	F	F	F	F	F	F	F	F	F	F	F	T	F	F	F	T	F
YNL222W	F	F	F	F	F	F	F	F	F	F	F	F	F	F	F	F	F	F	F	F	F	F	F
YNL223W	F	F	F	F	F	F	F	F	F	F	F	F	F	F	F	F	F	T	F	F	F	T	F
YNL224C	F	F	F	F	F	F	F	F	F	F	F	F	F	F	F	F	F	T	F	F	F	T	F
YNL225C	F	F	F	T	F	F	F	F	F	F	F	F	F	F	F	F	F	F	F	F	F	F	F
YNL227C	F	F	F	F	F	F	F	F	F	F	F	F	F	F	F	F	F	T	F	F	F	F	F
YNL229C	F	F	F	F	F	F	F	F	F	F	F	F	F	F	F	F	F	T	F	F	F	F	F
YNL230C	F	F	F	F	F	F	F	F	F	F	F	F	F	F	F	F	F	F	F	F	F	T	F
YNL232W	F	F	F	F	F	F	F	F	F	F	F	F	F	F	F	F	T	T	F	F	F	T	F
YNL233W	F	F	F	F	T	F	F	F	F	F	T	F	F	F	F	F	F	T	F	F	F	F	F
YNL236W	F	F	F	F	F	F	F	F	F	F	F	F	F	F	F	F	F	F	F	F	F	T	F
YNL238W	F	F	F	F	F	T	F	F	F	F	F	F	F	F	F	F	F	F	F	T	F	F	F
YNL239W	F	T	F	F	F	F	F	F	F	F	F	F	F	F	F	F	F	T	F	F	F	F	F
YNL241C	F	F	F	F	F	F	F	F	F	F	F	F	F	F	F	F	F	T	F	F	F	F	F
YNL242W	T	F	F	F	F	F	F	F	F	F	F	F	F	F	F	F	F	F	F	F	F	F	F
YNL243W	F	F	F	F	F	T	F	F	F	F	F	F	F	F	F	F	F	F	F	F	F	F	F
YNL244C	F	F	F	F	F	F	F	F	F	F	F	F	F	F	F	F	F	F	F	F	F	F	F
YNL245C	F	F	F	F	F	F	F	F	F	F	F	F	F	F	F	F	F	F	F	F	F	T	F
YNL246W	F	F	F	F	F	F	F	F	F	F	F	F	F	F	F	F	F	F	F	F	F	T	F
YNL247W	F	F	F	F	F	F	F	F	F	F	F	F	F	F	F	F	F	T	F	F	F	F	F
YNL248C	F	F	F	F	F	F	F	F	F	F	F	F	F	F	F	F	T	F	F	F	F	F	F
YNL250W	F	F	F	F	F	F	F	F	F	F	F	F	F	F	F	F	F	T	F	F	F	T	F
YNL251C	F	F	F	F	F	F	F	F	F	F	F	F	F	F	F	F	F	F	F	F	F	T	F
YNL252C	F	T	F	F	F	F	F	F	F	F	F	F	F	F	F	F	F	F	F	F	F	F	F
YNL253W	F	F	F	F	F	F	F	F	F	F	F	F	F	F	F	F	F	F	F	F	F	F	F
YNL257C	T	F	F	F	F	F	F	F	F	F	F	F	F	F	F	F	F	F	F	F	F	F	F
YNL258C	F	F	F	F	F	F	F	T	F	F	F	F	F	F	F	F	F	F	F	F	F	F	F
YNL260C	F	F	F	F	F	F	F	F	F	F	F	F	F	F	F	F	F	F	F	F	F	F	F
YNL261W	F	F	F	F	F	F	F	F	F	F	F	F	F	F	F	F	F	F	F	F	F	T	F
YNL262W	F	F	F	F	F	F	F	F	F	F	F	F	F	F	F	F	F	T	F	F	F	T	F
YNL263C	F	F	F	F	F	F	F	F	F	F	F	F	F	F	F	F	F	F	F	F	F	F	F
YNL264C	F	F	F	F	F	F	F	F	F	F	F	F	F	F	F	F	F	T	F	F	F	F	F
YNL265C	F	F	F	F	F	F	F	F	F	T	F	F	F	F	F	F	F	F	F	F	F	F	F
YNL267W	F	F	F	F	F	F	F	F	F	F	F	F	F	F	F	F	F	T	F	F	F	F	F
YNL271C	F	F	F	F	F	F	F	F	F	F	T	F	F	F	F	F	F	T	F	F	F	F	F
YNL272C	T	F	F	F	F	F	F	F	F	F	T	F	F	F	F	F	F	T	F	F	F	F	T
YNL273W	F	F	F	F	F	F	F	F	F	F	F	F	F	F	F	F	F	F	F	F	F	T	F
YNL280C	F	F	F	F	F	F	F	T	F	F	F	F	F	F	F	F	F	F	F	F	F	F	F
YNL281W	F	F	F	F	F	F	F	F	F	F	F	F	F	F	F	F	F	T	F	F	F	T	F
YNL282W	F	F	F	F	F	F	F	F	F	F	F	F	F	F	F	F	F	F	F	F	F	T	F
YNL284C	F	T	F	F	F	F	F	F	F	F	F	F	F	F	F	F	F	F	F	F	F	F	F
YNL286W	F	F	F	F	F	F	F	F	F	F	F	F	F	F	F	F	F	F	F	F	F	T	F
YNL287W	F	F	F	F	F	F	F	F	F	F	F	F	T	F	F	F	F	F	F	T	F	F	F
YNL288W	F	F	F	F	F	F	F	F	F	F	F	F	F	F	F	F	F	T	F	F	F	F	F
YNL289W	F	F	F	F	F	F	F	F	F	F	F	F	F	F	F	F	F	F	F	F	F	F	F
YNL290W	F	F	F	F	F	F	F	F	F	F	F	F	F	F	F	F	F	F	F	F	F	T	F
YNL293W	T	F	F	F	F	F	F	F	F	F	F	F	F	F	F	F	F	F	F	F	F	F	F
YNL297C	F	F	F	F	F	T	F	F	F	F	F	F	F	F	F	F	F	F	F	T	F	F	F
YNL298W	T	F	F	F	T	F	F	F	F	F	F	F	F	F	F	F	F	T	F	F	F	F	T
YNL299W	F	F	F	F	F	F	F	F	F	F	F	F	F	F	F	F	T	F	F	F	F	F	F
YNL301C	F	F	F	F	F	F	F	F	F	F	F	F	F	F	F	F	F	T	F	F	F	F	F

YNL304W	F	F	F	F	F	F	F	F	F	F	F	F	F	F	F	F	F	T	F	F	F	F	F
YNL306W	F	T	F	F	F	F	F	F	F	F	F	F	F	F	F	F	F	F	F	F	F	F	F
YNL307C	F	F	F	F	F	F	F	F	F	F	F	F	F	F	F	F	F	T	F	F	F	T	F
YNL308C	F	F	F	F	F	F	F	F	F	F	F	F	F	F	F	F	T	T	F	F	F	F	F
YNL309W	F	F	F	F	F	F	F	F	F	F	F	F	F	F	F	F	F	T	F	F	F	T	F
YNL310C	F	T	F	F	F	F	F	F	F	F	F	F	F	F	F	F	F	F	F	F	F	F	F
YNL311C	F	F	F	F	F	F	F	F	F	F	F	F	F	F	F	F	F	T	F	F	F	F	F
YNL312W	F	F	F	F	F	F	F	F	F	F	F	F	F	F	F	F	F	F	F	F	F	T	F
YNL313C	F	F	F	F	F	F	F	F	F	F	F	F	F	F	F	F	F	T	F	F	F	T	F
YNL315C	F	T	F	F	F	F	F	F	F	F	F	F	F	F	F	F	F	F	F	F	F	F	F
YNL317W	F	F	F	F	F	F	F	F	F	F	F	F	F	F	F	F	F	F	F	F	F	T	F
YNL321W	F	F	F	F	F	F	F	F	F	F	F	F	F	F	F	F	F	F	F	F	F	F	F
YNL325C	T	F	F	F	F	F	F	F	F	F	F	F	F	F	F	F	F	F	F	F	F	F	F
YNL326C	F	F	T	F	F	F	F	F	F	F	F	F	F	F	F	F	F	F	F	F	F	F	F
YNL329C	F	F	F	F	F	F	F	F	F	F	F	F	F	F	T	F	F	F	F	F	F	F	F
YNL330C	F	F	F	F	F	F	F	F	F	F	F	F	F	F	F	F	F	T	F	F	F	T	F
YNL333W	F	F	F	F	F	F	F	F	F	F	F	F	F	F	F	F	F	F	F	F	F	F	F
YNL334C	F	F	F	F	F	F	F	F	F	F	F	F	F	F	F	F	F	F	F	F	F	F	F
YNR001C	F	T	F	F	F	F	F	F	F	F	F	F	F	F	F	F	F	F	F	F	F	F	F
YNR003C	F	F	F	F	F	F	F	F	F	F	F	F	F	F	F	F	F	F	F	F	F	F	F
YNR006W	F	F	F	F	F	F	F	F	F	T	F	F	F	F	F	F	F	F	F	F	F	F	F
YNR007C	F	F	F	F	F	F	F	F	F	F	F	F	F	F	F	F	F	T	F	F	F	F	F
YNR009W	F	F	F	F	F	F	F	F	F	F	F	F	F	F	F	F	F	T	F	F	F	T	F
YNR010W	F	F	F	F	F	F	F	F	F	F	F	F	F	F	F	F	F	F	F	F	F	T	F
YNR011C	F	F	F	F	F	F	F	F	F	F	F	F	F	F	F	F	F	T	F	F	F	T	F
YNR012W	F	F	F	F	F	F	F	F	F	F	F	F	F	F	F	F	F	T	F	F	F	T	F
YNR016C	F	F	F	F	F	T	F	F	F	F	F	F	F	F	F	F	F	T	F	F	F	F	F
YNR017W	F	T	F	F	F	F	F	F	F	F	F	F	F	F	F	F	F	F	F	F	F	F	F
YNR023W	F	F	F	F	F	F	F	F	F	F	F	F	F	F	F	F	F	F	F	F	F	T	F
YNR024W	F	F	F	F	F	F	F	F	F	F	F	F	F	F	F	F	F	F	F	F	F	T	F
YNR027W	F	F	F	F	F	F	F	F	F	F	F	F	F	F	F	F	F	T	F	F	F	T	F
YNR029C	F	F	F	F	F	F	F	F	F	F	F	F	F	F	F	F	F	T	F	F	F	F	F
YNR031C	F	F	F	F	F	F	F	F	F	F	F	F	F	F	F	F	F	T	F	F	F	F	F
YNR034W	F	F	F	F	F	F	F	F	F	F	F	F	F	F	F	F	F	T	F	F	F	T	F
YNR035C	F	F	F	F	F	F	F	F	F	F	F	F	F	F	F	T	F	F	F	F	F	F	F
YNR037C	F	F	F	F	F	F	F	F	F	F	F	F	F	F	F	F	F	F	F	F	F	F	F
YNR038W	F	F	F	F	F	F	F	F	F	F	F	F	F	F	F	F	T	F	F	F	F	T	F
YNR043W	F	F	F	F	F	F	F	F	F	F	F	F	F	F	F	F	F	T	F	F	F	F	F
YNR046W	F	F	F	F	F	F	F	F	F	F	F	F	F	F	F	F	F	T	F	F	F	T	F
YNR047W	F	F	F	F	F	F	F	F	F	F	F	F	F	F	F	F	F	T	F	F	F	F	F
YNR049C	T	F	F	F	F	F	F	F	F	F	T	F	F	F	F	F	F	T	F	F	F	F	T
YNR050C	F	F	F	F	F	F	F	F	F	F	F	F	F	F	F	F	F	T	F	F	F	F	F
YNR051C	F	F	F	F	F	F	F	F	F	F	F	F	F	F	F	F	F	T	F	F	F	F	F
YNR052C	F	T	F	F	F	F	F	F	F	F	F	F	F	F	F	F	F	T	F	F	F	F	F
YNR053C	F	F	F	F	F	F	F	F	F	F	F	F	F	F	F	F	F	F	F	F	F	T	F
YNR054C	F	F	F	F	F	F	F	F	F	F	F	F	F	F	F	F	T	T	F	F	F	F	F
YNR068C	F	F	F	F	F	F	F	F	F	F	F	F	F	F	F	F	F	F	F	F	F	F	F
YNR069C	F	F	F	F	F	F	F	F	F	F	F	F	F	F	F	F	F	F	F	F	F	F	F
YOL001W	F	F	F	F	F	F	F	F	F	F	F	F	F	F	F	F	F	T	F	F	F	T	F
YOL004W	F	T	F	F	F	F	F	F	F	F	F	F	F	F	F	F	F	F	F	F	F	F	F
YOL005C	F	F	F	F	F	F	F	F	F	F	F	F	F	F	F	F	F	F	F	F	F	T	F
YOL006C	F	F	F	F	F	F	F	F	F	F	F	F	F	F	F	F	T	F	F	F	F	T	F
YOL009C	F	T	F	F	F	F	F	F	F	F	F	F	F	F	F	F	F	F	F	F	F	F	F
YOL010W	F	F	F	F	F	F	F	F	F	F	F	F	F	F	F	F	T	F	F	F	F	T	F
YOL012C	F	F	F	F	F	F	F	F	F	F	F	F	F	F	F	F	F	F	F	F	F	T	F
YOL013C	F	F	F	F	F	F	F	T	F	F	F	F	F	F	F	F	F	F	F	F	F	F	F
YOL016C	F	F	F	F	F	F	F	F	F	F	F	F	F	F	F	F	F	T	F	F	F	F	F
YOL017W	F	F	F	F	F	F	F	F	F	F	F	F	F	F	F	F	F	T	F	F	F	T	F
YOL018C	F	F	F	F	F	F	F	F	F	F	F	F	F	F	F	F	F	F	F	F	F	F	F
YOL021C	F	F	F	F	F	F	F	F	F	F	F	F	F	F	F	F	T	T	F	F	F	T	F
YOL023W	F	T	F	F	F	F	F	F	F	F	F	F	F	F	F	F	F	F	F	F	F	F	F
YOL025W	F	F	F	F	F	F	F	F	F	F	F	F	F	F	F	F	F	F	F	F	F	F	F
YOL027C	F	T	F	F	F	F	F	F	F	F	F	F	F	F	F	F	F	F	F	F	F	F	F
YOL031C	T	F	F	F	F	F	F	F	F	F	F	F	F	F	F	F	F	F	F	F	F	F	F
YOL033W	F	T	F	F	F	F	F	F	F	F	F	F	F	F	F	F	F	F	F	F	F	F	F
YOL034W	F	F	F	F	F	F	F	F	F	F	F	F	F	F	F	F	F	T	F	F	F	T	F
YOL036W	F	F	F	F	F	F	F	F	F	F	F	F	F	F	F	F	F	F	F	F	F	F	F
YOL038W	F	F	F	F	F	F	F	F	F	F	F	F	F	F	F	F	F	T	F	F	F	T	F

YOL039W	F	F	F	F	F	F	F	F	F	F	F	F	F	F	F	F	F	T	F	F	F	F	F
YOL040C	F	F	F	F	F	F	F	F	F	F	F	F	F	F	F	F	F	F	F	F	F	F	F
YOL041C	F	F	F	F	F	F	F	F	F	F	F	F	F	F	F	F	T	F	F	F	F	F	F
YOL043C	F	T	F	F	F	F	F	F	F	F	F	F	F	F	F	F	F	F	F	F	F	F	F
YOL044W	F	F	F	F	F	T	F	F	F	F	F	F	F	F	F	F	F	F	F	F	F	F	F
YOL045W	F	F	F	F	F	F	F	F	F	F	F	F	F	F	F	F	F	T	F	F	F	F	F
YOL051W	F	F	F	F	F	F	F	F	F	F	F	F	F	F	F	F	F	F	F	F	F	T	F
YOL054W	F	F	F	F	F	F	F	F	F	F	F	F	F	F	F	F	F	F	F	F	F	T	F
YOL056W	F	F	F	F	F	F	F	F	F	F	F	F	F	F	F	F	F	T	F	F	F	F	F
YOL057W	F	F	F	F	F	F	F	F	F	F	F	F	F	F	F	F	F	T	F	F	F	T	F
YOL058W	F	F	F	F	F	F	F	F	F	F	F	F	F	F	F	F	F	T	F	F	F	F	F
YOL061W	F	F	F	F	F	F	F	F	F	F	F	F	F	F	F	F	F	T	F	F	F	F	F
YOL062C	F	F	F	F	T	F	F	F	F	F	T	F	F	F	F	F	F	F	F	F	F	F	F
YOL063C	F	F	F	F	F	F	F	F	F	F	F	F	F	F	F	F	F	F	F	F	F	F	F
YOL067C	F	F	F	F	F	F	F	F	F	F	F	F	F	F	F	F	F	T	F	F	F	T	F
YOL068C	F	F	F	F	F	F	F	F	F	F	F	F	F	F	F	F	F	T	F	F	F	T	F
YOL069W	F	F	F	T	F	F	F	F	F	F	F	F	F	F	F	F	F	F	F	F	F	F	F
YOL070C	F	F	F	F	T	F	F	F	F	F	T	F	F	F	F	F	F	T	F	F	F	F	F
YOL072W	F	F	F	F	F	F	F	F	T	F	F	F	F	F	F	F	F	F	F	F	F	F	F
YOL076W	F	F	F	F	F	F	F	F	F	F	F	F	F	F	F	F	F	T	F	F	F	F	F
YOL077C	F	F	F	F	F	F	F	F	F	F	F	F	F	F	F	F	T	F	F	F	F	T	F
YOL078W	F	F	F	F	F	F	F	F	F	F	F	F	F	F	F	F	F	F	F	F	F	F	F
YOL080C	F	F	F	F	F	F	F	F	F	F	F	F	F	F	F	F	T	F	F	F	F	T	F
YOL081W	F	F	F	F	F	F	F	F	F	F	F	F	F	F	F	F	F	T	F	F	F	F	F
YOL082W	F	F	F	F	F	T	F	F	F	F	F	F	F	F	F	F	F	F	F	F	F	F	F
YOL083W	F	F	F	F	F	F	F	F	F	F	F	F	F	F	F	F	F	F	F	F	F	F	F
YOL086C	F	F	F	F	F	F	F	F	F	F	F	F	F	F	F	F	F	F	F	F	F	F	F
YOL087C	F	F	F	F	F	F	F	F	F	F	F	F	F	F	F	F	F	T	F	F	F	F	F
YOL090W	F	F	F	F	F	F	F	F	F	F	F	F	F	F	F	F	F	F	F	F	F	T	F
YOL091W	F	F	F	F	F	F	F	F	F	F	F	F	F	F	F	F	F	F	F	F	F	F	F
YOL094C	F	F	F	F	F	F	F	F	F	F	F	F	F	F	F	F	F	F	F	F	F	T	F
YOL097C	F	F	F	F	F	F	F	F	F	F	F	F	F	F	F	F	F	T	F	F	F	F	F
YOL098C	F	F	F	F	F	F	F	F	F	F	F	F	F	F	F	F	F	T	F	F	F	F	F
YOL100W	F	F	F	F	F	F	F	F	F	F	F	F	F	F	F	F	F	T	F	F	F	F	F
YOL104C	F	F	F	F	F	F	F	F	F	F	F	F	F	F	F	F	F	F	F	F	F	F	F
YOL108C	F	F	F	F	F	F	F	F	F	F	F	F	F	F	F	F	F	T	F	F	F	T	F
YOL109W	F	F	F	F	F	F	F	F	F	F	F	F	F	F	F	F	F	F	F	F	F	F	F
YOL111C	F	F	F	F	F	F	F	F	F	F	F	F	F	F	F	F	F	T	F	F	F	F	F
YOL112W	T	F	F	F	T	F	F	F	F	F	T	F	F	F	F	F	F	F	F	F	F	F	T
YOL113W	T	F	F	F	F	F	F	F	F	F	F	F	F	F	F	F	F	F	F	F	F	F	F
YOL115W	F	F	F	F	F	F	F	F	F	F	F	F	F	F	F	F	F	F	F	F	F	T	F
YOL117W	F	F	F	F	F	F	F	F	F	F	F	F	F	F	F	F	F	T	F	F	F	F	F
YOL120C	F	F	F	F	F	F	F	F	F	F	F	F	F	F	F	F	F	F	F	F	F	F	F
YOL122C	F	F	F	F	F	F	F	F	F	F	F	F	F	F	F	F	F	F	F	F	F	F	F
YOL123W	F	F	F	F	F	F	F	F	F	F	F	F	F	F	F	F	F	F	F	F	F	T	F
YOL124C	F	F	F	F	F	F	F	F	F	F	F	F	F	F	F	F	F	T	F	F	F	F	F
YOL126C	F	F	F	F	F	F	F	F	F	F	F	F	F	F	T	F	F	F	F	F	F	F	F
YOL127W	F	F	F	F	F	F	F	F	F	F	F	F	F	F	F	F	F	F	F	F	F	F	F
YOL130W	T	F	F	F	F	F	F	F	F	F	F	F	F	F	F	F	F	F	F	F	F	F	F
YOL133W	F	F	F	F	F	F	F	F	F	F	F	F	F	F	F	F	F	F	F	F	F	F	F
YOL135C	F	F	F	F	F	F	F	F	F	F	F	F	F	F	F	F	F	F	F	F	F	T	F
YOL139C	F	F	F	F	F	F	F	F	F	F	F	F	F	F	F	F	F	T	F	F	F	F	F
YOL142W	F	F	F	F	F	F	F	F	F	F	F	F	F	F	F	F	T	F	F	F	F	T	F
YOL144W	F	F	F	F	F	F	F	F	F	F	F	F	F	F	F	F	T	F	F	F	F	F	F
YOL145C	F	F	F	F	F	F	F	F	F	F	F	F	F	F	F	F	F	F	F	F	F	T	F
YOL146W	F	F	F	F	F	F	F	F	F	F	F	F	F	F	F	F	F	F	F	F	F	F	F
YOL147C	F	F	F	F	F	F	F	F	F	F	F	F	F	F	T	F	F	F	F	F	F	F	F
YOL148C	F	F	F	F	F	F	F	F	F	F	F	F	F	F	F	F	F	F	F	F	F	T	F
YOL149W	F	F	F	F	F	T	F	F	F	F	F	F	F	F	F	F	F	F	F	F	F	F	F
YOL151W	F	F	F	F	F	F	F	F	F	F	F	F	F	F	F	F	F	T	F	F	F	T	F
YOR001W	F	F	F	F	F	F	F	F	F	F	F	F	F	F	F	F	T	F	F	F	F	T	F
YOR005C	F	F	F	F	F	F	F	F	F	F	F	F	F	F	F	F	F	F	F	F	F	F	F
YOR007C	F	F	F	F	F	F	F	F	F	F	F	F	F	F	F	F	F	T	F	F	F	F	F
YOR014W	F	F	F	F	F	F	F	F	F	F	F	F	F	F	F	F	F	T	F	F	F	T	F
YOR016C	F	F	F	F	F	F	F	T	F	F	F	F	F	F	F	F	F	F	F	F	F	F	F
YOR018W	F	F	F	F	F	F	F	F	F	F	F	F	F	F	F	F	F	T	F	F	F	F	F
YOR020C	F	F	F	F	F	F	F	F	F	F	F	F	F	F	F	F	F	F	F	F	F	F	F
YOR023C	F	F	F	F	F	F	F	F	F	F	F	F	F	F	F	F	F	T	F	F	F	T	F

YOR026W	F	F	F	F	F	F	F	F	F	F	F	F	F	F	F	F	F	T	F	F	F	T	F
YOR027W	F	F	F	F	F	F	F	F	F	F	F	F	F	F	F	F	F	T	F	F	F	F	F
YOR028C	F	F	F	F	F	F	F	F	F	F	F	F	F	F	F	F	F	T	F	F	F	T	F
YOR032C	F	F	F	F	F	F	F	F	F	F	F	F	F	F	F	F	F	F	F	F	F	F	F
YOR033C	F	F	F	F	F	F	F	F	F	F	F	F	F	F	F	F	F	F	F	F	F	T	F
YOR034C	T	F	F	F	F	F	F	F	F	F	F	F	F	F	F	F	F	F	F	F	F	F	F
YOR035C	F	F	F	F	F	F	F	F	F	F	F	F	F	F	F	F	F	T	F	F	F	F	F
YOR036W	F	F	F	F	F	F	F	F	F	F	F	F	F	F	F	F	F	F	F	F	F	F	F
YOR038C	F	F	F	F	F	F	F	F	F	F	F	F	F	F	F	F	F	F	F	F	F	T	F
YOR039W	F	F	F	F	F	F	F	F	F	F	F	F	F	F	F	F	F	T	F	F	F	T	F
YOR042W	F	F	F	F	F	T	F	F	F	F	F	F	F	F	F	F	F	F	F	F	F	F	F
YOR043W	F	F	F	F	T	F	F	F	F	F	F	F	F	F	F	F	F	F	F	F	F	F	F
YOR046C	F	F	F	F	F	F	F	F	T	F	F	F	F	F	F	F	F	F	F	F	F	F	F
YOR047C	F	F	F	F	F	F	F	F	F	F	F	F	F	F	F	F	F	F	F	F	F	F	F
YOR048C	F	F	F	F	F	F	F	F	F	F	F	F	F	F	F	F	F	F	F	F	F	T	F
YOR054C	F	F	F	F	F	F	F	F	F	F	F	F	F	F	F	F	F	F	F	F	F	F	F
YOR056C	F	F	F	F	F	F	F	F	F	F	F	F	F	F	F	F	F	T	F	F	F	F	F
YOR057W	F	F	F	F	F	F	F	F	F	F	F	F	F	F	F	F	F	T	F	F	F	T	T
YOR058C	F	F	F	F	F	F	F	F	F	F	F	T	F	F	F	F	F	F	F	F	F	F	F
YOR059C	T	F	F	F	F	F	F	F	F	F	F	F	F	F	F	F	F	F	F	F	F	F	F
YOR061W	F	F	F	F	F	F	F	F	F	F	F	F	F	F	F	F	F	T	F	F	F	T	F
YOR063W	F	F	F	F	F	F	F	F	F	F	F	F	F	F	F	F	F	T	F	F	F	F	F
YOR064C	F	F	F	F	F	F	F	F	F	F	F	F	F	F	F	F	F	F	F	F	F	T	F
YOR065W	F	T	F	F	F	F	F	F	F	F	F	F	F	F	F	F	F	F	F	F	F	F	F
YOR066W	F	F	F	F	F	F	F	F	F	F	F	F	F	F	F	F	F	F	F	F	F	F	F
YOR069W	F	F	F	F	F	F	F	F	F	T	F	F	F	F	F	F	F	F	F	F	F	F	F
YOR070C	F	F	F	F	F	T	F	F	F	F	F	F	F	F	F	F	F	T	F	F	F	F	F
YOR075W	T	F	F	F	F	F	F	F	F	F	F	F	F	F	F	F	F	F	F	F	F	F	F
YOR076C	T	F	F	F	F	F	F	F	F	F	F	F	F	F	F	F	F	F	F	F	F	F	F
YOR078W	F	F	F	F	F	F	F	F	F	F	F	F	F	F	F	F	T	F	F	F	F	T	F
YOR080W	F	F	F	F	F	F	F	F	F	F	F	F	F	F	F	F	F	F	F	F	F	F	F
YOR083W	F	F	F	F	F	F	F	F	F	F	F	F	F	F	F	F	F	T	F	F	F	T	F
YOR085W	T	F	F	F	F	F	F	F	F	F	F	F	F	F	F	F	F	F	F	F	F	F	F
YOR086C	F	F	F	F	T	F	F	F	F	F	F	F	F	F	F	F	F	F	F	F	F	F	F
YOR089C	F	F	F	F	F	F	F	F	F	F	F	F	F	F	F	F	F	T	F	F	F	T	F
YOR090C	F	F	F	F	F	F	F	F	F	F	F	F	F	F	F	F	F	F	F	F	F	F	F
YOR091W	F	F	F	F	F	F	F	F	F	F	F	F	F	F	F	F	F	T	F	F	F	F	F
YOR094W	F	F	F	F	T	F	F	F	F	F	F	F	F	F	F	F	F	F	F	F	F	F	F
YOR096W	F	F	F	F	F	F	F	F	F	F	F	F	F	F	F	F	F	F	F	F	F	F	F
YOR098C	F	F	F	F	F	F	F	F	T	F	F	F	F	F	F	F	F	F	F	F	F	F	F
YOR101W	F	F	F	F	F	F	F	F	F	F	F	F	F	F	F	F	F	T	F	F	F	T	F
YOR103C	F	F	F	F	F	F	F	F	F	F	F	F	F	F	F	F	F	F	F	F	F	F	F
YOR106W	F	F	F	F	F	F	F	F	F	F	F	F	F	F	F	F	F	F	F	F	F	F	F
YOR108W	F	T	F	F	F	F	F	F	F	F	F	F	F	F	F	F	F	F	F	F	F	F	F
YOR109W	F	F	F	F	F	T	F	F	F	F	F	F	F	F	F	F	F	F	F	F	F	F	F
YOR110W	F	F	F	F	F	F	F	F	F	F	F	F	F	F	F	F	F	T	F	F	F	T	F
YOR111W	F	F	F	F	F	F	F	F	F	F	F	F	F	F	F	F	F	F	F	F	F	F	F
YOR112W	F	F	F	F	F	F	F	F	F	F	F	F	F	F	F	F	F	T	F	F	F	F	F
YOR115C	F	F	F	F	F	T	F	F	F	F	F	F	F	F	F	F	F	F	F	F	F	F	F
YOR116C	F	F	F	F	F	F	F	F	F	F	F	F	F	F	F	F	F	F	F	F	F	T	F
YOR117W	F	F	F	F	F	F	F	F	F	F	F	F	F	F	F	F	F	F	F	F	F	T	F
YOR119C	F	F	F	F	F	F	F	F	F	F	F	F	F	F	F	F	F	F	F	F	F	F	F
YOR122C	F	F	F	F	F	F	F	F	F	F	F	F	F	F	F	F	F	T	F	F	F	T	F
YOR123C	F	F	F	F	F	F	F	F	F	F	F	F	F	F	F	F	F	F	F	F	F	T	F
YOR124C	F	F	F	F	F	F	F	F	F	F	F	F	F	F	F	F	F	T	F	F	F	F	F
YOR127W	F	F	F	F	T	F	F	F	F	F	T	F	F	F	F	F	F	T	F	F	F	F	F
YOR129C	F	F	F	F	F	F	F	F	F	F	F	F	F	F	F	F	F	T	F	F	F	F	F
YOR132W	F	F	F	F	F	F	F	F	F	T	F	F	F	F	F	F	F	F	F	F	F	F	F
YOR133W	F	F	F	F	F	F	F	F	F	F	F	F	F	F	F	F	F	T	F	F	F	F	F
YOR134W	F	F	F	F	F	F	F	F	F	F	F	F	F	F	F	F	F	F	F	F	F	F	F
YOR136W	F	T	F	F	F	F	F	F	F	F	F	F	F	F	F	F	F	F	F	F	F	F	F
YOR138C	F	F	F	F	F	F	F	F	F	F	F	F	F	F	F	F	F	T	F	F	F	T	F
YOR140W	F	F	F	F	F	F	F	F	F	F	F	F	F	F	F	F	F	F	F	F	F	T	F
YOR141C	F	F	F	F	F	F	F	F	F	F	F	F	F	F	F	F	F	F	F	F	F	T	F
YOR144C	F	F	F	F	F	F	F	F	F	F	F	F	F	F	F	F	F	T	F	F	F	T	F
YOR145C	F	F	F	F	F	F	F	F	F	F	F	F	F	F	F	F	F	F	F	F	F	F	F
YOR148C	F	F	F	F	F	F	F	F	F	F	F	F	F	F	F	F	F	T	F	F	F	T	F
YOR150W	F	T	F	F	F	F	F	F	F	F	F	F	F	F	F	F	F	F	F	F	F	F	F

YOR151C	F	F	F	F	F	F	F	F	F	F	F	F	F	F	F	F	F	T	F	F	F	T	F
YOR155C	F	F	F	F	F	F	F	F	F	F	F	F	F	F	F	F	F	F	F	F	F	F	F
YOR156C	F	F	F	F	F	F	F	F	F	F	F	F	F	F	F	F	F	T	F	F	F	T	F
YOR157C	F	F	F	F	F	F	F	F	F	F	F	F	F	F	F	F	F	T	F	F	F	T	F
YOR158W	F	T	F	F	F	F	F	F	F	F	F	F	F	F	F	F	F	F	F	F	F	F	F
YOR159C	F	F	F	F	F	F	F	F	F	F	F	F	F	F	F	F	F	F	F	F	F	F	F
YOR160W	F	F	F	F	F	F	F	F	F	F	F	F	F	F	F	F	F	T	F	F	F	T	F
YOR164C	F	F	F	F	F	F	F	F	F	F	F	F	F	F	F	F	F	T	F	F	F	F	F
YOR165W	F	F	F	F	F	T	F	F	F	F	F	F	F	F	F	F	F	F	F	F	F	F	F
YOR167C	F	F	F	F	F	F	F	F	F	F	F	F	F	F	F	F	F	T	F	F	F	F	F
YOR168W	F	F	F	F	F	F	F	F	F	F	F	F	F	F	F	F	F	T	F	F	F	F	F
YOR171C	T	F	F	F	F	F	F	F	F	F	F	F	F	F	F	F	F	F	F	F	F	F	F
YOR172W	F	F	F	F	F	F	F	F	F	F	F	F	F	F	F	F	F	T	F	F	F	T	F
YOR173W	F	F	F	F	F	F	F	F	F	F	F	F	F	F	F	F	F	T	F	F	F	F	F
YOR174W	F	F	F	F	F	F	F	F	F	F	F	F	F	F	F	F	F	F	F	F	F	T	F
YOR176W	F	T	F	F	F	F	F	F	F	F	F	F	F	F	F	F	F	F	F	F	F	F	F
YOR177C	F	F	F	F	F	F	F	F	F	F	F	F	F	F	F	F	F	F	F	F	F	F	F
YOR178C	F	F	F	F	F	F	F	F	F	F	F	F	F	F	F	F	F	F	F	F	F	F	F
YOR179C	F	F	F	F	F	F	F	F	F	F	F	F	F	F	F	F	F	F	F	F	F	T	F
YOR181W	F	F	F	F	F	F	F	F	F	F	F	F	F	F	F	T	F	F	F	F	F	F	F
YOR184W	F	F	F	F	F	F	F	F	F	F	F	F	F	F	F	F	F	T	F	F	F	F	F
YOR185C	F	F	F	F	F	F	F	F	F	F	F	F	F	F	F	F	F	T	F	F	F	T	F
YOR187W	F	T	F	F	F	F	F	F	F	F	F	F	F	F	F	F	F	F	F	F	F	F	F
YOR189W	F	F	F	F	F	F	F	F	F	F	F	F	F	F	F	F	F	F	F	F	F	T	F
YOR191W	F	F	F	F	F	F	F	F	F	F	F	F	F	F	F	F	F	F	F	F	F	T	F
YOR194C	F	F	F	F	F	F	F	F	F	F	F	F	F	F	F	F	F	F	F	F	F	F	F
YOR195W	F	F	F	T	F	F	F	F	F	F	F	F	F	F	F	F	F	F	F	F	F	F	F
YOR196C	F	T	F	F	F	F	F	F	F	F	F	F	F	F	F	F	F	F	F	F	F	F	F
YOR197W	F	F	F	F	F	F	F	F	F	F	F	F	F	F	F	F	F	T	F	F	F	T	F
YOR198C	F	F	F	F	F	F	F	T	F	F	F	F	F	F	F	F	F	F	F	F	F	F	F
YOR201C	F	T	F	F	F	F	F	F	F	F	F	F	F	F	F	F	F	F	F	F	F	F	F
YOR204W	F	F	F	F	F	F	F	F	F	F	F	F	F	F	F	F	F	F	F	F	F	F	F
YOR206W	F	F	F	F	F	F	F	F	F	F	F	F	F	F	F	F	T	F	F	F	F	T	F
YOR207C	F	F	F	F	F	F	F	F	F	F	F	F	F	F	F	F	F	F	F	F	F	T	F
YOR208W	F	F	F	F	F	F	F	F	F	F	F	F	F	F	F	F	F	T	F	F	F	T	F
YOR210W	F	F	F	F	F	F	F	F	F	F	F	F	F	F	F	F	T	F	F	F	F	T	F
YOR211C	F	F	F	F	F	F	F	F	F	F	F	F	F	F	F	F	F	F	F	F	F	F	F
YOR212W	F	F	F	F	F	F	F	F	F	F	F	F	F	F	F	F	F	T	F	F	F	F	F
YOR213C	F	F	F	F	F	F	F	F	F	F	F	F	F	F	F	F	F	T	F	F	F	T	F
YOR216C	F	F	F	F	F	T	F	F	F	F	F	F	T	F	F	F	F	F	F	F	F	F	F
YOR217W	F	F	F	F	F	F	F	F	F	F	F	F	F	F	F	F	F	F	F	F	F	T	F
YOR224C	F	F	F	F	F	F	F	F	F	F	F	F	F	F	F	F	F	F	F	F	F	T	F
YOR227W	F	F	F	F	F	F	F	F	F	F	F	F	F	F	F	F	F	T	F	F	F	F	F
YOR229W	F	F	F	F	F	F	F	F	F	F	F	F	F	F	F	F	F	F	F	F	F	T	F
YOR230W	F	F	F	F	F	F	F	F	F	F	F	F	F	F	F	F	F	F	F	F	F	T	F
YOR231W	T	F	F	F	F	F	F	F	F	F	F	F	F	F	F	F	F	F	F	F	F	F	F
YOR232W	F	F	F	F	F	F	F	F	F	F	F	F	F	F	F	F	F	T	F	F	F	F	F
YOR233W	F	F	F	F	T	F	F	F	F	F	F	F	F	F	F	F	F	F	F	F	F	F	F
YOR243C	F	F	F	F	F	F	F	F	F	F	F	F	F	F	F	F	F	F	F	F	F	T	F
YOR244W	F	F	F	F	F	F	F	F	F	F	F	F	F	F	F	F	F	F	F	F	F	T	F
YOR247W	F	F	T	F	F	F	F	F	F	F	F	F	F	F	F	F	F	T	F	F	F	F	F
YOR249C	F	F	F	F	F	F	F	F	F	F	F	F	F	F	F	F	F	F	F	F	F	T	F
YOR250C	F	F	F	F	F	F	F	F	F	F	F	F	F	F	F	F	F	F	F	F	F	F	F
YOR253W	F	F	F	F	F	F	F	F	F	F	F	F	F	F	F	F	F	T	F	F	F	F	F
YOR254C	F	F	F	F	F	F	F	T	F	F	F	F	F	F	F	F	F	F	F	F	F	F	F
YOR257W	F	F	F	F	F	F	F	F	F	F	F	F	F	F	F	F	F	F	F	F	F	F	F
YOR259C	F	F	F	F	F	F	F	F	F	F	F	F	F	F	F	F	F	F	F	F	F	T	F
YOR260W	F	F	F	F	F	F	F	F	F	F	F	F	F	F	F	F	F	T	F	F	F	F	F
YOR261C	F	F	F	F	F	F	F	F	F	F	F	F	F	F	F	F	F	T	F	F	F	T	F
YOR264W	F	F	F	F	F	F	F	F	F	F	T	F	F	F	F	F	F	F	F	F	F	F	F
YOR265W	F	F	F	F	F	F	F	F	F	F	F	F	F	F	F	F	F	T	F	F	F	F	F
YOR267C	F	F	F	F	F	F	F	F	F	F	F	F	F	F	F	F	F	T	F	F	F	F	F
YOR269W	F	F	F	F	F	F	F	F	F	F	F	F	F	F	F	F	F	T	F	F	F	T	F
YOR270C	F	F	F	F	F	F	T	F	F	F	F	F	F	F	F	F	F	F	F	F	F	F	F
YOR272W	F	F	F	F	F	F	F	F	F	F	F	F	F	F	F	F	T	F	F	F	F	T	F
YOR275C	F	F	F	F	F	F	F	F	F	F	F	F	F	F	F	F	F	T	F	F	F	T	F
YOR276W	F	F	F	F	F	F	F	F	F	F	F	F	F	F	F	F	F	T	F	F	F	F	F
YOR279C	F	F	F	F	F	F	F	F	F	F	F	F	F	F	F	F	F	T	F	F	F	T	F

YOR281C	F	F	F	F	F	F	F	F	F	F	F	F	F	F	F	F	F	T	F	F	F	F	F
YOR283W	F	F	F	F	F	F	F	F	F	F	F	F	F	F	F	F	F	T	F	F	F	T	F
YOR284W	F	F	F	F	F	T	F	F	F	F	F	F	F	F	F	F	F	F	F	F	F	F	F
YOR290C	F	F	F	F	F	F	F	F	F	F	F	F	F	F	F	F	F	F	F	F	F	T	F
YOR294W	F	F	F	F	F	F	F	F	F	F	F	F	F	F	F	F	F	F	F	F	F	F	F
YOR297C	F	T	F	F	F	F	F	F	F	F	F	F	F	F	F	F	F	F	F	F	F	F	F
YOR298C-A	F	F	F	F	F	F	F	F	F	F	F	F	F	F	F	F	F	T	F	F	F	F	F
YOR299W	F	F	F	F	F	F	F	F	F	F	F	F	F	T	F	F	F	F	F	F	F	F	F
YOR301W	F	F	F	F	F	F	F	F	F	F	F	F	F	F	F	F	F	F	F	F	F	F	F
YOR303W	F	F	F	F	F	F	F	F	F	F	F	F	F	F	F	F	F	T	F	F	F	F	F
YOR304W	F	F	F	F	F	F	F	F	F	F	F	F	F	F	F	F	F	F	F	F	F	T	F
YOR308C	F	F	F	F	F	F	F	F	F	F	F	F	F	F	F	F	F	F	F	F	F	T	F
YOR310C	F	F	F	F	F	F	F	F	F	F	F	F	F	F	F	F	T	F	F	F	F	F	F
YOR312C	F	F	F	F	F	F	F	F	F	F	F	F	F	F	F	F	F	T	F	F	F	F	F
YOR315W	F	F	F	F	F	F	F	F	F	F	F	F	F	F	F	F	F	T	F	F	F	T	F
YOR317W	F	F	F	F	F	F	F	T	F	F	F	F	F	F	F	F	F	F	F	F	F	F	F
YOR319W	F	F	F	F	F	F	F	F	F	F	F	F	F	F	F	F	F	F	F	F	F	T	F
YOR322C	F	F	F	F	F	F	F	F	F	F	F	F	F	T	F	F	F	T	F	F	F	F	F
YOR323C	F	F	F	F	F	F	F	F	F	F	F	F	F	F	F	F	F	T	F	F	F	T	F
YOR324C	T	F	F	F	F	F	F	F	F	F	F	F	F	F	F	F	F	F	F	F	F	F	F
YOR326W	T	F	F	F	T	F	F	F	F	F	T	F	F	F	F	F	F	T	F	F	F	F	T
YOR327C	F	F	T	F	F	F	F	F	F	F	F	F	F	F	F	F	F	F	F	F	F	F	F
YOR329C	T	F	F	F	F	F	F	F	F	F	F	F	F	F	F	F	F	F	F	F	F	F	F
YOR330C	F	T	F	F	F	F	F	F	F	F	F	F	F	F	F	F	F	F	F	F	F	F	F
YOR332W	F	F	F	F	F	F	T	F	F	F	F	F	F	F	F	F	F	F	F	F	F	F	F
YOR335C	F	F	F	F	F	F	F	F	F	F	F	F	F	F	F	F	F	T	F	F	F	F	F
YOR340C	F	F	F	F	F	F	F	F	F	F	F	F	F	F	F	F	T	F	F	F	F	F	F
YOR341W	F	F	F	F	F	F	F	F	F	F	F	F	F	F	F	F	T	F	F	F	F	F	F
YOR344C	F	F	F	F	F	F	F	F	F	F	F	F	F	F	F	F	F	T	F	F	F	T	F
YOR346W	F	F	F	F	F	F	F	F	F	F	F	F	F	F	F	F	F	T	F	F	F	T	F
YOR347C	F	F	F	F	F	F	F	F	F	F	F	F	F	F	F	F	F	T	F	F	F	F	F
YOR349W	T	F	F	F	F	F	F	F	F	F	F	F	F	F	F	F	F	F	F	F	F	F	F
YOR351C	F	F	F	F	F	F	F	F	F	F	F	F	F	F	F	F	F	F	F	F	F	F	F
YOR352W	F	F	F	F	F	F	F	F	F	F	F	F	F	F	F	F	F	T	F	F	F	T	F
YOR353C	F	F	F	F	F	F	F	F	F	F	F	F	F	F	F	F	F	T	F	F	F	F	F
YOR355W	F	F	F	F	F	F	F	F	F	F	F	F	F	F	F	F	F	T	F	F	F	T	F
YOR358W	F	F	F	F	F	F	F	F	F	F	F	F	F	F	F	F	F	T	F	F	F	T	F
YOR361C	F	F	F	F	F	F	F	F	F	F	F	F	F	F	F	F	F	T	F	F	F	F	F
YOR362C	F	F	F	F	F	F	F	F	F	F	F	F	F	F	F	F	F	T	F	F	F	T	F
YOR367W	F	F	F	F	F	F	F	F	F	F	F	F	F	F	F	T	F	F	F	F	F	F	F
YOR368W	F	F	F	F	F	F	F	F	F	F	F	F	F	F	F	F	F	F	F	F	F	F	F
YOR369C	F	F	F	F	F	F	F	F	F	F	F	F	F	F	F	F	F	F	F	F	F	F	F
YOR370C	F	F	F	F	F	F	F	F	F	F	F	F	F	F	F	F	F	T	F	F	F	T	F
YOR371C	F	F	F	F	F	F	F	F	F	F	F	F	F	F	F	F	F	T	F	F	F	F	F
YOR372C	F	F	F	F	F	F	F	F	F	F	F	F	F	F	F	F	F	T	F	F	F	T	F
YOR373W	F	F	F	T	F	F	F	F	F	F	F	F	F	F	F	F	F	F	F	F	F	F	F
YOR375C	F	F	F	F	F	F	F	F	F	F	F	F	F	F	F	F	F	T	F	F	F	T	F
YOR385W	F	F	F	F	F	F	F	F	F	F	F	F	F	F	F	F	F	T	F	F	F	F	F
YPL001W	F	F	F	F	F	F	F	F	F	F	F	F	F	F	F	F	F	F	F	F	F	F	F
YPL002C	T	F	F	F	F	F	F	F	F	F	F	F	F	F	F	F	F	F	F	F	F	F	F
YPL003W	F	F	F	F	F	F	F	F	F	F	F	F	F	F	F	F	F	F	F	F	F	F	F
YPL004C	F	F	F	F	F	T	F	F	F	F	F	F	F	F	F	F	F	F	F	F	F	F	F
YPL007C	F	F	F	F	F	F	F	F	F	F	F	F	F	F	F	F	F	F	F	F	F	T	F
YPL008W	F	F	F	F	F	F	F	F	F	F	F	F	F	F	F	F	F	T	F	F	F	T	F
YPL009C	F	F	F	F	F	F	F	F	F	F	F	F	F	F	F	F	F	T	F	F	F	F	F
YPL010W	F	F	F	F	F	T	F	F	F	F	F	F	F	F	F	F	F	F	F	F	F	F	F
YPL011C	F	F	F	F	F	F	F	F	F	F	F	F	F	F	F	F	F	F	F	F	F	T	F
YPL012W	F	F	F	F	F	F	F	F	F	F	F	F	F	F	F	F	F	T	F	F	F	T	F
YPL013C	F	T	F	F	F	F	F	F	F	F	F	F	F	F	F	F	F	F	F	F	F	F	F
YPL016W	F	F	F	F	F	F	F	F	F	F	F	F	F	F	F	F	F	F	F	F	F	T	F
YPL018W	T	F	F	T	F	F	F	F	F	F	F	F	F	F	F	F	F	F	F	F	F	F	F
YPL019C	F	F	T	F	F	F	F	F	F	F	F	F	F	F	F	F	F	F	F	F	F	F	F
YPL020C	F	F	F	F	F	F	F	F	T	F	F	F	F	F	F	F	F	F	F	F	F	F	F
YPL022W	F	F	F	F	F	F	F	F	F	F	F	F	F	F	F	F	F	F	F	F	F	T	F
YPL023C	F	F	F	F	F	F	F	F	F	F	F	F	F	F	F	F	F	F	F	F	F	F	F
YPL024W	F	F	F	F	F	F	F	F	F	F	F	F	F	F	F	F	F	T	F	F	F	T	F
YPL026C	F	F	F	F	F	F	F	F	F	F	F	F	F	F	F	F	F	T	F	F	F	F	F
YPL028W	F	F	F	F	F	F	F	F	F	F	F	F	F	F	F	F	F	F	F	F	F	T	F

YPL031C	F	F	F	F	F	F	F	F	F	F	F	F	F	F	F	F	F	T	F	F	F	T	F
YPL032C	T	F	F	F	T	F	F	F	F	F	T	F	F	F	F	F	F	T	F	F	F	F	T
YPL037C	F	F	F	F	F	F	F	F	F	F	F	F	F	F	F	F	F	T	F	F	F	F	F
YPL038W	F	F	F	F	F	F	F	F	F	F	F	F	F	F	F	F	F	T	F	F	F	T	F
YPL042C	F	F	F	F	F	F	F	F	F	F	F	F	F	F	F	F	F	F	F	F	F	F	F
YPL043W	F	F	F	F	F	F	F	F	F	F	F	F	F	F	F	F	T	F	F	F	F	T	F
YPL045W	F	F	F	F	F	F	T	F	F	F	F	F	F	F	F	F	F	F	F	F	F	F	F
YPL046C	F	F	F	F	F	F	F	F	F	F	F	F	F	F	F	F	F	T	F	F	F	T	F
YPL047W	F	F	F	F	F	F	F	F	F	F	F	F	F	F	F	F	F	F	F	F	F	T	F
YPL048W	F	F	F	F	F	F	F	F	F	F	F	F	F	F	F	F	F	T	F	F	F	F	F
YPL049C	F	F	F	F	F	F	F	F	F	F	F	F	F	F	F	F	F	F	F	F	F	T	F
YPL050C	F	F	F	F	F	F	F	F	F	F	F	F	T	F	F	F	F	F	F	F	F	F	F
YPL055C	F	F	F	F	F	F	F	F	F	F	F	F	F	F	F	F	F	F	F	F	F	T	F
YPL059W	F	T	F	F	F	F	F	F	F	F	F	F	F	F	F	F	F	F	F	F	F	F	F
YPL061W	F	F	F	F	F	F	F	F	F	F	F	F	F	F	F	F	F	T	F	F	F	T	F
YPL063W	F	T	F	F	F	F	F	F	F	F	F	F	F	F	F	F	F	F	F	F	F	F	F
YPL064C	F	F	F	F	F	F	F	F	F	F	F	F	F	F	F	F	F	T	F	F	F	T	F
YPL065W	F	F	F	F	F	T	F	F	F	F	F	F	F	F	F	F	F	F	F	F	F	F	F
YPL066W	F	F	F	F	F	F	F	F	F	F	T	F	F	F	F	F	F	T	F	F	F	F	F
YPL070W	F	F	F	F	F	F	F	F	F	F	F	F	F	F	F	F	F	T	F	F	F	F	F
YPL074W	F	F	F	F	F	F	F	F	F	F	F	F	F	F	F	F	F	T	F	F	F	F	F
YPL075W	F	F	F	F	F	F	F	F	F	F	F	F	F	F	F	F	F	T	F	F	F	T	F
YPL077C	F	F	F	F	F	F	F	F	F	F	F	F	F	F	F	F	F	F	F	F	F	F	F
YPL078C	F	T	F	F	F	F	F	F	F	F	F	F	F	F	F	F	F	F	F	F	F	F	F
YPL081W	F	F	F	F	F	F	F	F	F	F	F	F	F	F	F	F	F	T	F	F	F	F	F
YPL082C	F	F	F	F	F	F	F	F	F	F	F	F	F	F	F	F	F	F	F	F	F	T	F
YPL083C	F	T	F	F	F	F	F	F	F	F	F	F	F	F	F	F	F	F	F	F	F	F	F
YPL084W	F	F	F	F	F	F	F	F	F	T	F	F	F	F	F	F	F	T	F	F	F	F	F
YPL085W	F	F	F	F	F	F	F	F	F	F	F	F	F	F	F	F	F	F	T	F	F	F	F
YPL086C	F	F	F	F	F	F	F	F	F	F	F	F	F	F	F	F	F	T	F	F	F	F	F
YPL088W	F	F	F	F	F	F	F	F	F	F	F	F	F	F	F	F	F	F	F	F	F	F	F
YPL089C	F	F	F	F	F	F	F	F	F	F	F	F	F	F	F	F	F	T	F	F	F	T	F
YPL093W	F	F	F	F	F	F	F	F	F	F	F	F	F	F	F	F	F	F	F	F	F	T	F
YPL094C	F	F	F	F	F	F	F	T	F	F	F	F	F	F	F	F	F	F	F	F	F	F	F
YPL096W	F	F	F	F	F	F	F	F	F	F	F	F	F	F	F	F	F	T	F	F	F	F	F
YPL101W	F	F	F	F	F	F	F	F	F	F	F	F	F	F	F	F	F	F	F	F	F	F	F
YPL104W	F	T	F	F	F	F	F	F	F	F	F	F	F	F	F	F	F	F	F	F	F	F	F
YPL105C	F	F	F	F	F	F	F	F	F	F	F	F	F	F	F	F	F	T	F	F	F	F	F
YPL106C	F	F	F	F	F	F	F	F	F	F	F	F	F	F	F	F	F	T	F	F	F	F	F
YPL108W	F	F	F	F	F	F	F	F	F	F	F	F	F	F	F	F	F	T	F	F	F	F	F
YPL110C	F	F	F	F	F	F	F	F	F	F	F	F	F	F	F	F	F	T	F	F	F	F	F
YPL111W	F	F	F	F	F	F	F	F	F	F	F	F	F	F	F	F	F	T	F	F	F	T	F
YPL112C	F	F	F	F	F	F	F	F	F	F	F	F	F	F	T	F	F	F	F	F	F	F	F
YPL115C	F	F	F	F	F	F	F	F	F	F	T	F	F	F	F	F	F	T	F	F	F	F	F
YPL118W	F	T	F	F	F	F	F	F	F	F	F	F	F	F	F	F	F	F	F	F	F	F	F
YPL120W	F	F	T	F	F	F	F	F	F	F	F	F	F	F	F	F	F	F	F	F	F	F	F
YPL121C	F	F	F	F	F	F	F	F	F	F	F	F	F	F	F	F	F	F	F	F	F	F	F
YPL122C	F	F	F	F	F	F	F	F	F	F	F	F	F	F	F	F	F	F	F	F	F	T	F
YPL124W	F	F	F	T	F	F	F	F	F	F	F	F	F	F	F	F	F	F	F	F	F	F	F
YPL125W	F	F	F	F	F	F	F	F	F	F	F	F	F	F	F	F	F	T	F	F	F	T	F
YPL126W	F	F	F	F	F	F	F	F	F	F	F	F	F	F	F	F	T	F	F	F	F	F	F
YPL127C	F	F	F	F	F	F	F	F	F	F	F	F	F	F	F	F	F	F	F	F	F	T	F
YPL128C	F	F	F	F	F	F	F	F	F	F	F	F	F	F	F	F	F	F	F	F	F	T	F
YPL129W	F	F	F	F	F	F	F	F	F	F	F	F	F	F	F	F	F	F	F	F	F	T	F
YPL131W	F	F	F	F	F	F	F	F	F	F	F	F	F	F	F	F	F	T	F	F	F	F	F
YPL133C	F	F	F	F	F	F	F	F	F	F	F	F	F	F	F	F	F	T	F	F	F	T	F
YPL135W	F	T	F	F	F	F	F	F	F	F	F	F	F	F	F	F	F	F	F	F	F	F	F
YPL137C	F	F	F	F	F	F	F	T	F	F	F	F	F	F	F	F	F	T	F	F	F	F	F
YPL138C	F	F	F	F	F	F	F	F	F	F	F	F	F	F	F	F	F	F	F	F	F	T	F
YPL139C	F	F	F	F	F	F	F	F	F	F	F	F	F	F	F	F	F	T	F	F	F	T	F
YPL140C	F	F	F	F	F	F	F	F	F	F	F	F	F	F	F	F	F	T	F	F	F	F	F
YPL141C	F	F	F	F	F	F	F	F	F	F	F	F	F	F	F	F	F	T	F	F	F	F	F
YPL143W	F	F	F	F	F	F	F	F	F	F	F	F	F	F	F	F	F	F	F	F	F	F	F
YPL144W	F	F	F	F	F	F	F	F	F	F	F	F	F	F	F	F	F	T	F	F	F	F	F
YPL145C	F	F	F	F	F	F	F	F	F	F	F	F	F	F	F	F	F	T	F	F	F	F	F
YPL146C	F	F	F	F	F	F	F	F	F	F	F	F	F	F	F	F	F	F	F	F	F	T	F
YPL149W	F	F	F	F	F	F	F	F	F	F	F	F	F	F	F	F	F	F	F	F	F	F	F
YPL150W	T	F	F	F	F	F	F	F	F	F	F	F	F	F	F	F	F	F	F	F	F	F	F

YPL151C	F	F	F	F	F	F	F	F	F	F	F	F	F	F	F	F	F	T	F	F	F	T	F
YPL152W	F	F	F	F	F	F	F	F	F	F	F	F	F	F	F	F	F	T	F	F	F	F	F
YPL153C	F	F	F	F	F	F	F	F	F	F	F	F	F	F	F	F	F	F	F	F	F	T	F
YPL154C	F	F	T	F	F	F	F	F	F	F	F	F	F	F	F	F	F	F	F	F	F	F	F
YPL155C	F	F	F	T	F	F	F	F	F	F	F	T	F	F	F	F	F	F	F	F	F	F	F
YPL160W	F	F	F	F	F	F	F	F	F	F	F	F	F	F	F	F	F	T	F	F	F	F	F
YPL161C	F	F	F	F	F	F	F	F	F	F	F	F	F	F	F	F	F	T	F	F	F	T	F
YPL164C	F	F	F	F	F	F	F	F	F	F	F	F	F	F	F	F	F	F	F	F	F	F	F
YPL165C	F	F	F	F	F	F	F	F	F	F	F	F	F	F	F	F	F	F	F	F	F	F	F
YPL166W	F	F	F	F	F	T	F	F	F	F	F	F	F	F	F	F	F	T	F	F	F	T	F
YPL167C	F	F	F	F	F	F	F	F	F	F	F	F	F	F	F	F	F	F	F	F	F	F	F
YPL169C	F	F	F	F	F	F	F	F	T	F	F	F	F	F	F	F	F	F	F	F	F	F	F
YPL173W	F	F	F	F	F	F	F	F	F	F	F	F	F	F	F	F	F	F	F	F	F	F	F
YPL174C	F	F	F	F	F	F	F	F	F	F	F	F	F	F	F	F	F	F	F	F	F	F	F
YPL176C	F	F	F	F	F	F	F	F	F	F	F	F	F	F	F	F	F	F	F	F	F	F	F
YPL177C	F	F	F	F	F	F	F	F	F	F	F	F	F	F	F	F	F	T	F	F	F	T	F
YPL178W	F	F	F	F	F	F	F	F	F	F	F	F	F	F	F	F	F	F	F	F	F	T	F
YPL179W	F	F	F	F	F	F	F	F	F	F	F	F	F	F	F	F	F	T	F	F	F	F	F
YPL180W	F	F	F	F	F	F	T	F	F	F	F	F	F	F	F	F	F	F	F	F	F	F	F
YPL181W	F	F	F	F	F	F	F	F	F	F	F	F	F	F	F	F	F	F	F	F	F	T	F
YPL183C	F	F	F	F	F	F	F	F	F	F	F	F	F	F	F	F	F	T	F	F	F	F	F
YPL183W-A	F	T	F	F	F	F	F	F	F	F	F	F	F	F	F	F	F	F	F	F	F	F	F
YPL187W	F	F	F	F	F	F	F	F	F	F	F	F	F	F	F	F	F	F	F	F	F	F	F
YPL190C	F	F	F	F	F	F	F	F	F	F	F	F	F	F	F	F	F	F	F	F	F	T	F
YPL193W	F	F	F	F	F	F	F	F	F	F	F	F	F	F	F	F	F	T	F	F	F	T	F
YPL194W	F	F	F	F	F	F	F	F	F	F	F	F	F	F	F	F	F	T	F	F	F	T	F
YPL195W	F	F	F	F	F	F	F	F	F	F	F	F	F	F	F	F	F	F	F	T	F	F	F
YPL198W	F	F	F	F	F	F	F	F	F	F	F	F	F	F	F	F	T	T	F	F	F	F	F
YPL200W	F	F	F	F	F	F	F	F	F	F	F	F	F	F	F	F	F	F	F	F	F	F	F
YPL201C	F	F	F	F	F	F	F	F	F	F	F	F	F	F	F	F	F	F	F	F	F	F	F
YPL203W	F	F	F	F	F	F	F	F	F	F	F	F	F	F	F	F	F	F	F	F	F	T	F
YPL204W	T	F	F	F	F	F	F	F	F	F	T	F	F	F	F	F	F	T	F	F	F	T	T
YPL207W	F	F	F	F	F	F	F	T	F	F	F	F	F	F	F	F	F	F	F	F	F	F	F
YPL208W	F	F	F	F	F	F	F	F	F	F	F	F	F	F	F	F	F	T	F	F	F	T	F
YPL209C	F	F	F	F	F	F	F	F	F	F	F	T	F	F	F	F	F	F	F	F	F	F	F
YPL210C	F	F	F	F	F	F	F	T	F	F	F	F	F	F	F	F	F	F	F	F	F	F	F
YPL211W	F	F	F	F	F	F	F	F	F	F	F	F	F	F	F	F	T	F	F	F	F	T	F
YPL212C	F	F	F	F	F	F	F	F	F	F	F	F	F	F	F	F	F	F	F	F	F	T	F
YPL213W	F	F	F	F	F	F	F	F	F	F	F	F	F	F	F	F	F	F	F	F	F	T	F
YPL217C	F	F	F	F	F	F	F	F	F	F	F	F	F	F	F	F	F	F	F	F	F	F	F
YPL218W	F	F	F	F	F	F	F	F	F	F	F	F	F	F	F	F	F	F	F	F	F	F	F
YPL219W	F	F	F	F	F	F	F	F	F	F	F	F	F	F	F	F	F	T	F	F	F	T	F
YPL220W	F	F	F	F	F	F	F	F	F	F	F	F	F	F	F	F	F	T	F	F	F	F	F
YPL226W	F	F	F	F	F	F	F	F	F	F	F	F	F	F	F	F	F	T	F	F	F	F	F
YPL227C	F	F	F	F	F	F	F	T	F	F	F	F	F	F	F	F	F	F	F	F	F	F	F
YPL228W	F	F	F	F	F	F	F	F	F	F	F	F	F	F	F	F	F	F	F	F	F	T	F
YPL231W	F	F	F	F	F	F	F	F	F	F	F	F	F	F	F	F	F	T	F	F	F	F	F
YPL232W	F	F	F	F	F	F	F	F	F	F	F	F	F	F	F	F	F	F	F	F	T	F	F
YPL233W	F	F	F	T	F	F	F	F	F	F	F	F	F	F	F	F	F	F	F	F	F	F	F
YPL234C	F	F	F	F	F	F	F	T	F	F	F	F	F	F	F	F	F	F	F	F	F	F	F
YPL235W	F	F	F	F	F	F	F	F	F	F	F	F	F	F	F	F	F	F	F	F	F	F	F
YPL237W	F	F	F	F	F	F	F	F	F	F	F	F	F	F	F	F	F	F	F	F	F	F	F
YPL239W	F	F	F	F	F	F	F	F	F	F	F	F	F	F	F	F	F	T	F	F	F	F	F
YPL240C	F	F	F	F	F	F	F	F	F	F	F	F	F	F	F	F	F	T	F	F	F	F	F
YPL242C	F	F	F	F	F	F	F	F	F	F	T	F	F	F	F	F	F	F	F	F	F	F	F
YPL243W	F	F	F	F	F	F	F	F	F	F	F	F	F	F	F	F	F	T	F	F	F	F	F
YPL246C	F	F	F	F	F	F	F	F	F	F	F	F	T	F	F	F	F	F	F	T	F	F	F
YPL247C	F	F	F	F	F	F	F	F	F	F	F	F	F	F	F	F	F	T	F	F	F	T	F
YPL248C	F	F	F	F	F	F	F	F	F	F	F	F	F	F	F	F	F	F	F	F	F	F	F
YPL249C	T	F	F	F	T	F	F	F	F	F	T	F	F	F	F	F	F	T	F	F	F	F	T
YPL249C-A	F	F	F	F	F	F	F	F	F	F	F	F	F	F	F	F	F	T	F	F	F	F	F
YPL253C	F	F	F	F	F	F	F	F	F	F	F	F	F	F	F	F	F	F	F	F	F	F	F
YPL254W	F	F	F	F	F	F	F	F	F	F	F	F	F	F	F	F	F	F	F	F	F	T	F
YPL255W	F	F	F	T	F	F	F	F	F	F	F	F	F	F	F	F	F	F	F	F	F	F	F
YPL256C	F	F	F	F	F	F	F	F	F	F	F	F	F	F	F	F	F	T	F	F	F	T	F
YPL259C	F	F	F	F	F	F	F	F	F	F	F	F	F	T	F	F	F	F	F	F	F	F	F
YPL262W	F	T	F	F	F	F	F	F	F	F	F	F	F	F	F	F	F	T	F	F	F	F	F
YPL266W	F	F	F	F	F	F	F	F	F	F	F	F	F	F	F	F	F	F	F	F	F	F	F

YPL267W	F	F	F	F	F	F	F	F	F	F	F	F	F	F	F	F	F	F	F	F	F	F	F
YPL268W	F	F	F	F	F	F	F	F	F	F	F	F	F	F	F	F	F	F	F	F	F	F	F
YPL269W	T	F	F	T	F	F	F	F	F	F	F	F	F	F	F	F	F	F	F	F	F	F	F
YPL271W	F	T	F	F	F	F	F	F	F	F	F	F	F	F	F	F	F	F	F	F	F	F	F
YPR007C	F	F	F	F	F	F	F	F	F	F	F	F	F	F	F	F	F	F	F	F	F	F	F
YPR008W	F	F	F	F	F	F	F	F	F	F	F	F	F	F	F	F	F	T	F	F	F	F	F
YPR010C	F	F	F	F	F	F	F	F	F	F	F	F	F	F	F	F	T	F	F	F	F	F	F
YPR016C	F	F	F	F	F	F	F	F	F	F	F	F	F	F	F	F	T	F	F	F	F	T	F
YPR017C	F	F	F	F	F	F	F	F	F	F	F	F	F	F	F	F	F	T	F	F	F	T	F
YPR018W	F	F	F	F	F	F	F	F	F	F	F	F	F	F	F	F	F	F	F	F	F	F	F
YPR019W	F	F	F	F	F	F	F	F	F	F	F	F	F	F	F	F	F	T	F	F	F	T	F
YPR023C	F	F	F	F	F	F	F	F	F	F	F	F	F	F	F	F	F	F	F	F	F	T	F
YPR024W	F	T	F	F	F	F	F	F	F	F	F	F	F	F	F	F	F	F	F	F	F	F	F
YPR025C	F	F	F	F	F	F	F	F	F	F	F	F	F	F	F	F	F	F	F	F	F	T	F
YPR026W	F	F	F	F	F	F	F	F	F	F	F	F	F	F	F	F	F	F	F	F	F	F	F
YPR028W	F	F	F	F	F	F	F	T	F	F	F	F	F	F	F	F	F	F	F	F	F	F	F
YPR029C	F	F	F	F	F	T	F	F	F	F	F	F	F	F	F	F	F	F	F	F	F	F	F
YPR030W	F	F	F	F	F	F	F	F	F	F	F	F	F	F	F	F	F	F	F	F	F	F	F
YPR031W	F	F	F	F	F	F	F	F	F	F	F	F	F	F	F	F	F	T	F	F	F	T	F
YPR032W	F	F	F	F	T	F	F	F	F	F	F	F	F	F	F	F	F	T	F	F	F	F	T
YPR033C	F	F	F	F	F	F	F	F	F	F	F	F	F	F	F	F	F	T	F	F	F	F	F
YPR034W	F	F	F	F	F	F	F	F	F	F	F	F	F	F	F	F	F	F	F	F	F	T	F
YPR035W	F	F	F	F	F	F	F	F	F	F	F	F	F	F	F	F	F	T	F	F	F	F	F
YPR036W	F	F	F	F	F	F	T	F	F	F	F	F	F	F	F	F	F	F	F	F	F	F	F
YPR040W	F	F	F	F	F	F	F	F	F	F	F	F	F	F	F	F	F	T	F	F	F	T	F
YPR041W	F	F	F	F	F	F	F	F	F	F	F	F	F	F	F	F	F	T	F	F	F	F	F
YPR045C	F	F	F	F	F	F	F	F	F	F	F	F	F	F	F	F	F	F	F	F	F	T	F
YPR046W	F	F	F	F	F	F	F	F	F	F	F	F	F	F	F	F	F	F	F	F	F	T	F
YPR048W	F	F	F	F	F	F	F	F	F	F	F	F	F	F	F	F	F	F	F	F	F	F	F
YPR049C	F	F	F	F	F	T	F	F	F	F	F	F	F	F	F	F	F	F	F	F	F	F	F
YPR052C	F	F	F	F	F	F	F	F	F	F	F	F	F	F	F	F	F	F	F	F	F	T	F
YPR054W	F	F	F	F	F	F	F	F	F	F	F	F	F	F	F	F	F	F	F	F	F	F	F
YPR055W	T	F	F	F	T	F	F	F	F	F	T	F	F	F	F	F	F	T	F	F	F	F	T
YPR056W	F	F	F	F	F	F	F	F	F	F	F	F	F	F	F	F	F	F	F	F	F	T	F
YPR057W	F	F	F	F	F	F	F	F	F	F	F	F	F	F	F	F	F	F	F	F	F	T	F
YPR066W	F	F	F	F	F	T	F	F	F	F	F	F	F	F	F	F	F	T	F	F	F	F	F
YPR068C	F	F	F	F	F	F	F	F	F	F	F	F	F	F	F	F	F	F	F	F	F	F	F
YPR069C	F	F	F	F	F	F	F	F	F	F	F	F	F	F	F	F	F	T	F	F	F	T	F
YPR070W	F	F	F	F	F	F	F	F	F	F	F	F	F	F	F	F	F	F	F	F	F	T	F
YPR072W	F	F	F	F	F	F	F	F	F	F	F	F	F	F	F	F	F	T	F	F	F	F	F
YPR073C	F	F	F	F	F	F	F	F	F	F	F	F	F	F	F	F	F	T	F	F	F	T	F
YPR074C	F	F	F	F	F	F	F	F	F	F	F	F	F	F	F	F	F	T	F	F	F	T	F
YPR075C	F	F	T	F	F	F	F	F	F	F	F	F	F	F	F	F	F	F	F	F	F	F	F
YPR080W	F	F	F	F	F	F	F	F	F	F	F	F	F	F	F	F	F	T	F	F	F	F	F
YPR082C	F	F	F	F	F	F	F	F	F	F	F	F	F	F	F	F	F	F	F	F	F	F	F
YPR086W	F	F	F	F	F	F	F	F	F	F	F	F	F	F	F	F	F	F	F	F	F	T	F
YPR088C	F	F	F	F	F	F	F	F	F	F	F	F	F	F	F	F	F	F	F	F	F	F	F
YPR089W	F	F	F	F	F	F	F	F	F	F	F	F	F	F	F	F	F	F	F	F	F	F	F
YPR091C	F	F	F	F	F	F	F	T	F	F	F	F	F	F	F	F	F	F	F	F	F	F	F
YPR093C	F	F	F	F	F	F	F	F	F	F	F	F	F	F	F	F	F	F	F	F	F	F	F
YPR094W	F	F	F	F	F	F	F	F	F	F	F	F	F	F	F	F	F	F	F	F	F	F	F
YPR095C	F	F	F	F	F	F	F	F	F	F	F	F	F	F	F	F	F	F	F	F	F	F	F
YPR100W	F	T	F	F	F	F	F	F	F	F	F	F	F	F	F	F	F	F	F	F	F	F	F
YPR101W	F	F	F	F	F	F	F	F	F	F	F	F	F	F	F	F	F	F	F	F	F	T	F
YPR102C	F	F	F	F	F	F	F	F	F	F	F	F	F	F	F	F	F	T	F	F	F	F	F
YPR103W	F	F	F	F	F	F	F	F	F	F	F	F	F	F	F	F	F	T	F	F	F	T	F
YPR104C	F	F	F	F	F	F	F	F	F	F	F	F	F	F	F	F	F	F	F	F	F	T	F
YPR105C	F	F	F	F	F	F	F	F	F	F	F	F	F	F	F	F	F	F	F	T	F	F	F
YPR106W	F	F	F	F	F	F	F	F	F	F	F	F	F	F	F	F	F	F	F	F	F	F	F
YPR107C	F	F	F	F	F	F	F	F	F	F	F	F	F	F	F	F	F	F	F	F	F	T	F
YPR108W	F	F	F	F	F	F	F	F	F	F	F	F	F	F	F	F	F	F	F	F	F	T	F
YPR110C	F	F	F	F	F	F	F	F	F	F	F	F	F	F	F	F	T	F	F	F	F	T	F
YPR111W	F	F	F	F	F	F	F	F	F	F	F	F	F	F	F	F	F	T	F	F	F	F	F
YPR112C	F	F	F	F	F	F	F	F	F	F	F	F	F	F	F	F	F	F	F	F	F	F	F
YPR115W	F	F	F	F	F	F	F	F	F	F	F	F	F	F	F	F	F	T	F	F	F	F	F
YPR119W	T	F	F	F	F	F	F	F	F	F	F	F	F	F	F	F	F	F	F	F	F	T	F
YPR120C	F	F	F	F	F	F	F	F	F	F	F	F	F	F	F	F	F	F	F	F	F	F	F
YPR125W	F	T	F	F	F	F	F	F	F	F	F	F	F	F	F	F	F	F	F	F	F	F	F

YPR129W	F	F	F	F	F	F	F	F	F	F	F	F	F	F	F	F	F	T	F	F	F	F	F
YPR131C	F	F	F	F	F	F	F	F	F	F	F	F	F	F	F	F	F	T	F	F	F	F	F
YPR133C	F	F	F	F	F	F	F	F	F	F	F	F	F	F	F	F	F	F	F	F	F	F	F
YPR133W-A	F	F	F	F	F	F	F	F	F	F	F	F	F	F	F	F	F	F	F	F	F	F	F
YPR135W	F	F	F	F	F	F	F	F	F	F	F	F	F	F	F	F	F	F	F	F	F	T	F
YPR137W	F	F	F	F	F	F	F	F	F	F	F	F	F	F	F	F	T	F	F	F	F	F	F
YPR140W	F	T	F	F	F	F	F	F	F	F	F	F	F	F	F	F	F	F	F	F	F	F	F
YPR141C	F	F	F	T	F	F	F	F	F	F	F	T	F	F	F	F	F	F	F	F	F	T	F
YPR143W	F	F	F	F	F	F	F	F	F	F	F	F	F	F	F	F	T	F	F	F	F	T	F
YPR144C	F	F	F	F	F	F	F	F	F	F	F	F	F	F	F	F	F	F	F	F	F	T	F
YPR145W	F	F	F	F	F	F	F	F	F	F	F	F	F	F	F	F	F	T	F	F	F	F	F
YPR154W	F	F	F	F	F	F	F	F	F	F	F	F	F	F	F	F	F	T	F	F	F	T	F
YPR156C	F	F	F	F	T	F	F	F	F	F	F	F	F	F	F	F	F	F	F	F	F	F	F
YPR159W	F	F	T	F	F	F	F	F	F	F	F	F	F	F	F	F	F	F	F	F	F	F	F
YPR160W	F	F	F	F	F	F	F	F	F	F	F	F	F	F	F	F	F	T	F	F	F	F	F
YPR161C	F	F	F	F	F	F	F	F	F	F	F	F	F	F	F	F	F	F	F	F	F	T	F
YPR162C	F	F	F	F	F	F	F	F	F	F	F	F	F	F	F	F	F	F	F	F	F	F	F
YPR163C	F	F	F	F	F	F	F	F	F	F	F	F	F	F	F	F	F	T	F	F	F	F	F
YPR164W	F	F	F	F	F	F	F	F	F	F	F	F	F	F	F	F	F	F	F	F	F	F	F
YPR165W	F	F	F	F	F	F	F	F	F	F	F	F	F	F	F	F	F	F	F	F	F	F	F
YPR167C	F	F	F	F	F	F	F	F	F	F	F	F	F	F	F	F	F	F	F	F	F	F	F
YPR168W	F	F	F	F	F	F	F	F	F	F	F	F	F	F	F	F	F	T	F	F	F	T	F
YPR169W	F	F	F	F	F	F	F	F	F	F	F	F	F	F	F	F	T	F	F	F	F	T	F
YPR171W	F	F	F	F	F	F	F	F	F	F	F	F	F	F	F	T	F	F	F	F	F	F	F
YPR173C	F	F	F	F	F	F	F	F	F	T	F	F	F	F	F	F	F	F	F	F	F	F	F
YPR174C	F	F	F	F	F	F	F	F	T	F	F	F	F	F	F	F	F	F	F	F	F	F	F
YPR175W	F	F	F	F	F	F	F	F	F	F	F	F	F	F	F	F	F	T	F	F	F	T	F
YPR176C	F	F	F	F	F	F	F	F	F	F	F	F	F	F	F	F	F	F	F	F	F	F	F
YPR178W	F	F	F	F	F	F	F	F	F	F	F	F	F	F	F	F	F	T	F	F	F	T	F
YPR179C	F	F	F	F	F	F	F	F	F	F	F	F	F	F	F	F	F	F	F	F	F	T	F
YPR180W	F	F	F	F	F	F	F	F	F	F	F	F	F	F	F	F	F	F	F	F	F	T	F
YPR181C	F	F	F	F	F	F	F	F	F	F	F	F	F	F	F	F	F	F	F	F	F	F	F
YPR182W	F	F	F	F	F	F	F	F	F	F	F	F	F	F	F	F	F	F	F	F	F	T	F
YPR183W	F	F	F	F	F	F	F	T	F	F	F	F	F	F	F	F	F	F	F	F	F	F	F
YPR184W	F	F	F	F	F	F	F	F	F	F	F	F	F	F	F	F	F	T	F	F	F	F	F
YPR185W	F	F	F	F	F	F	F	F	F	F	F	F	F	F	F	F	F	T	F	F	F	F	F
YPR187W	F	F	F	F	F	F	F	F	F	F	F	F	F	F	F	F	F	T	F	F	F	T	F
YPR188C	F	F	F	F	F	F	F	F	F	F	T	F	F	F	F	F	F	F	F	F	F	F	F
YPR189W	F	F	F	F	F	F	F	F	F	F	F	F	F	F	F	F	F	T	F	F	F	F	F
YPR190C	F	F	F	F	F	F	F	F	F	F	F	F	F	F	F	F	F	T	F	F	F	T	F
YPR191W	F	T	F	F	F	F	F	F	F	F	F	F	F	F	F	F	F	F	F	F	F	F	F
YPR193C	F	F	F	F	F	F	F	F	F	F	F	F	F	F	F	F	F	F	F	F	F	F	F
YPR201W	F	F	F	F	F	F	F	F	F	F	F	F	F	F	F	F	F	F	F	F	F	F	F
