# Supplementary material for: Predicting multiplex subcellular localization of proteins using protein-protein interaction network: a comparative study
Source: BMC Bioinformatics. 2012 Jun 25;13(Suppl 10):S20. doi: 10.1186/1471-2105-13-S10-S20 (PMC3314587; doi:10.1186/1471-2105-13-S10-S20)
Supplement: Additional file 3 — The subnetwork consists of 72 proteins and 204 interactions. [file 1471-2105-13-S10-S20-S3.pdf]

Supplementary File 3 The subnetwork consists of 72 proteins and 204 interactions. These proteins are the ones labeled with localization “ER to Golgi” and “Lipid particle” as well as their immediate neighbors.

Vertices

1	YAL030W	NONE
2	YBR041W	;lipid particle
3	YBR177C	;ER
4	YCR067C	;ER
5	YDL052C	;ER
6	YDL116W	;nuclear periphery
7	YDL132W	;cytoplasm;nucleus
8	YDL195W	;ER to Golgi
9	YDL229W	;cytoplasm
10	YDR164C	;ambiguous;bud neck;cytoplasm;bud
11	YDR337W	;mitochondrion
12	YDR382W	;cytoplasm
13	YDR425W	;endosome;cytoplasm;lipid particle
14	YDR498C	;ER
15	YDR517W	;punctate composite
16	YER044C	;ER
17	YER143W	;cytoplasm
18	YFR004W	;cytoplasm;nucleus
19	YGL001C	;ER
20	YGL012W	;ER
21	YGL092W	;nuclear periphery
22	YGL098W	;lipid particle
23	YGL100W	NONE
24	YGL145W	;ER
25	YGR009C	NONE
26	YGR060W	;vacuole;ER
27	YHR007C	;ER
28	YHR023W	;bud neck
29	YHR072W	;lipid particle
30	YHR098C	;ER to Golgi
31	YHR190W	;ER
32	YIL004C	;ambiguous
33	YIL109C	;ER to Golgi
34	YIL124W	;ER
35	YJL036W	;endosome
36	YJL164C	;cytoplasm;nucleus
37	YJR042W	;nuclear periphery
38	YKL057C	NONE
39	YKL094W	;ER

40	YKL140W	;punctate composite
41	YKL179C	;lipid particle
42	YKL196C	NONE
43	YKR082W	;nuclear periphery
44	YLL039C	;cytoplasm;nucleus
45	YLR026C	NONE
46	YLR056W	;ER
47	YLR100W	;lipid particle
48	YLR208W	;ER to Golgi
49	YLR268W	;Golgi
50	YLR440C	;ER
51	YML008C	;lipid particle
52	YML130C	NONE
53	YMR015C	;ER
54	YMR017W	NONE
55	YMR183C	;punctate composite;endosome
56	YMR202W	;ambiguous
57	YMR246W	;lipid particle
58	YNL049C	;ER to Golgi
59	YNL112W	;cytoplasm;nucleus
60	YNL258C	;ER
61	YNL280C	;ER
62	YNR049C	;ambiguous;bud neck;cytoplasm;bud
63	YOR075W	;ambiguous
64	YOR216C	;punctate composite;Golgi
65	YOR250C	NONE
66	YOR317W	;ER
67	YOR327C	;vacuole
68	YPL085W	;ER to Golgi
69	YPL218W	NONE
70	YPL232W	;lipid particle
71	YPR032W	;cell periphery;cytoplasm;bud
72	YPR181C	NONE

Edges

YAL030W	YER143W
YAL030W	YGR009C
YAL030W	YJL036W
YAL030W	YLL039C
YAL030W	YMR183C
YAL030W	YNR049C
YAL030W	YPL232W
YBR041W	YBR177C
YBR041W	YDL052C
YBR041W	YHR072W

YBR041W	YIL124W
YBR041W	YKL094W
YBR041W	YKL140W
YBR041W	YMR246W
YBR041W	YOR317W
YBR177C	YFR004W
YCR067C	YPL085W
YDL116W	YGL092W
YDL116W	YGL100W
YDL116W	YJR042W
YDL116W	YKL057C
YDL116W	YKR082W
YDL116W	YLR208W
YDL132W	YLR100W
YDL195W	YDR337W
YDL195W	YDR382W
YDL195W	YIL004C
YDL195W	YIL109C
YDL195W	YLR208W
YDL195W	YNL112W
YDL195W	YOR250C
YDL195W	YPL085W
YDL195W	YPR181C
YDL229W	YDR337W
YDL229W	YFR004W
YDL229W	YLL039C
YDL229W	YLR268W
YDL229W	YNL049C
YDL229W	YNL112W
YDR164C	YGR009C
YDR164C	YMR183C
YDR164C	YNR049C
YDR164C	YOR327C
YDR164C	YPL232W
YDR425W	YJL036W
YDR498C	YGL098W
YDR498C	YGL145W
YDR498C	YLR268W
YDR498C	YLR440C
YDR498C	YNL258C
YDR498C	YOR075W
YDR517W	YIL109C
YDR517W	YPR181C
YER044C	YGL001C
YER044C	YGL012W
YER044C	YGR060W

YER044C	YHR007C
YER044C	YHR072W
YER044C	YHR190W
YER044C	YLR056W
YER044C	YLR100W
YER044C	YML008C
YER044C	YMR202W
YER044C	YNL280C
YER143W	YFR004W
YER143W	YLL039C
YER143W	YOR327C
YER143W	YPL232W
YFR004W	YHR190W
YFR004W	YIL109C
YFR004W	YML008C
YFR004W	YMR202W
YFR004W	YMR246W
YFR004W	YNL112W
YFR004W	YOR317W
YFR004W	YPL218W
YFR004W	YPR181C
YGL001C	YGL012W
YGL001C	YGR060W
YGL001C	YHR007C
YGL001C	YHR072W
YGL001C	YHR190W
YGL001C	YLR056W
YGL001C	YLR100W
YGL001C	YML008C
YGL001C	YMR202W
YGL001C	YNL280C
YGL012W	YGR060W
YGL012W	YHR007C
YGL012W	YLR100W
YGL012W	YML008C
YGL012W	YMR015C
YGL012W	YNL280C
YGL092W	YGL100W
YGL092W	YJR042W
YGL092W	YKL057C
YGL092W	YKR082W
YGL092W	YLR208W
YGL098W	YGL145W
YGL098W	YKL196C
YGL098W	YLR268W
YGL098W	YLR440C

YGL098W	YNL258C
YGL098W	YOR075W
YGL100W	YJR042W
YGL100W	YKL057C
YGL100W	YKR082W
YGL100W	YLR208W
YGL145W	YLR268W
YGL145W	YLR440C
YGL145W	YNL258C
YGL145W	YOR075W
YGR009C	YMR183C
YGR009C	YNR049C
YGR009C	YOR327C
YGR009C	YPL232W
YGR009C	YPR032W
YGR060W	YHR007C
YGR060W	YHR072W
YGR060W	YHR190W
YGR060W	YLR056W
YGR060W	YLR100W
YGR060W	YML008C
YGR060W	YMR015C
YGR060W	YMR202W
YHR007C	YHR072W
YHR007C	YHR190W
YHR007C	YLL039C
YHR007C	YLR056W
YHR007C	YLR100W
YHR007C	YML008C
YHR007C	YNL280C
YHR023W	YKL179C
YHR072W	YLR056W
YHR072W	YLR100W
YHR072W	YML008C
YHR072W	YMR015C
YHR098C	YPR181C
YHR190W	YLR056W
YHR190W	YLR100W
YHR190W	YML008C
YIL004C	YIL109C
YIL004C	YKL196C
YIL004C	YLR026C
YIL004C	YLR268W
YIL004C	YPL218W
YIL004C	YPR181C
YIL109C	YLR026C

YIL109C	YLR268W
YIL109C	YPL085W
YIL109C	YPL218W
YIL109C	YPR181C
YJL164C	YPL232W
YJR042W	YKL057C
YJR042W	YKR082W
YJR042W	YLR208W
YKL057C	YKR082W
YKL057C	YLR208W
YKL179C	YOR216C
YKL196C	YLR026C
YKL196C	YLR268W
YKL196C	YLR440C
YKL196C	YPL232W
YKR082W	YLR208W
YLL039C	YMR015C
YLL039C	YMR246W
YLR026C	YLR268W
YLR026C	YOR327C
YLR026C	YPL218W
YLR056W	YLR100W
YLR056W	YML008C
YLR056W	YNL280C
YLR100W	YML008C
YLR100W	YMR015C
YLR100W	YMR202W
YLR100W	YNL280C
YLR208W	YML130C
YLR208W	YPL085W
YLR208W	YPR181C
YLR268W	YMR183C
YLR268W	YOR075W
YLR268W	YPL218W
YLR268W	YPL232W
YLR268W	YPR181C
YLR440C	YNL258C
YLR440C	YOR075W
YML008C	YMR015C
YML008C	YMR202W
YML008C	YNL280C
YMR017W	YOR327C
YMR017W	YPL232W
YMR183C	YNR049C
YMR183C	YOR327C
YMR183C	YPL232W

YMR202W	YNL280C
YNL049C	YPL085W
YNL049C	YPR181C
YNL258C	YOR075W
YNR049C	YOR327C
YNR049C	YPL232W
YOR327C	YPL232W
YPL085W	YPR181C
YPL218W	YPR181C
YPL232W	YPR032W
