# Supplementary material for: Predicting multiplex subcellular localization of proteins using protein-protein interaction network: a comparative study
Source: BMC Bioinformatics. 2012 Jun 25;13(Suppl 10):S20. doi: 10.1186/1471-2105-13-S10-S20 (PMC3314587; doi:10.1186/1471-2105-13-S10-S20)
Supplement: Additional file 4 — The subnetwork consists of 83 proteins and 164 interactions. [file 1471-2105-13-S10-S20-S4.pdf]

Supplementary File 4 The subnetwork consists of 83 proteins and 164 interactions. These proteins are the one labeled with localization “bud” as well as their immediate neighbors.

Vertices

1	YPL256C	cytoplasm;nucleus
2	YLR131C	cytoplasm;nucleus;bud
3	YBR109C	bud neck;cell periphery;bud
4	YPL242C	bud neck
5	YBR102C	ambiguous;bud neck;cell periphery;bud
6	YHR030C	cytoplasm;nucleus
7	YPL240C	cytoplasm
8	YGR191W	ER;cell periphery;vacuole;bud
9	YGR009C	NONE
10	YKL129C	cytoplasm;actin
11	YPL232W	lipid particle
12	YJR104C	ambiguous;cytoplasm;nucleus
13	YOR057W	cytoplasm;nucleus;bud
14	YNL078W	bud neck;cell periphery
15	YFL005W	NONE
16	YPL218W	NONE
17	YFL004W	ER
18	YPR110C	nucleolus;nucleus
19	YDL155W	cytoplasm;nucleus
20	YPL019C	vacuole
21	YGL242C	NONE
22	YBR069C	cell periphery;bud
23	YDR085C	NONE
24	YER008C	bud neck;cell periphery;bud
25	YJR077C	mitochondrion
26	YOL016C	cytoplasm
27	YER177W	cytoplasm
28	YGL233W	bud neck;cytoplasm;cell periphery
29	YOR212W	cytoplasm
30	YLR433C	ambiguous
31	YMR186W	cytoplasm
32	YJR056C	cytoplasm;nucleus
33	YER158C	NONE
34	YIL118W	NONE
35	YJL085W	bud neck;cell periphery;bud
36	YLR229C	NONE
37	YGL012W	ER
38	YHL007C	cytoplasm;bud
39	YML109W	cytoplasm
40	YPR055W	ambiguous;bud neck;cytoplasm;cell periphery;bud
41	YBR200W	ambiguous;bud neck;cell periphery;bud

42	YBR011C	cytoplasm;nucleus
43	YNR035C	actin
44	YKL042W	spindle pole
45	YER124C	bud neck
46	YAL041W	cytoplasm;nucleus
47	YER114C	bud neck;cytoplasm;cell periphery;punctate composite;bud
48	YPR032W	cytoplasm;cell periphery;bud
49	YIL068C	ambiguous;bud neck;cell periphery;bud
50	YGL162W	cytoplasm;nucleus
51	YLR371W	bud neck;cytoplasm;bud
52	YFR028C	nucleolus
53	YAL029C	ambiguous;bud neck;cytoplasm;cell periphery;bud
54	YOR326W	ambiguous;bud neck;cytoplasm;cell periphery;bud
55	YNL161W	bud neck;cytoplasm
56	YLR362W	cytoplasm
57	YDR356W	spindle pole
58	YFR014C	cytoplasm
59	YLR166C	ambiguous;bud neck;cell periphery;bud
60	YBL085W	bud neck;cytoplasm;cell periphery;bud
61	YIL043C	ER
62	YMR109W	actin
63	YJL012C	NONE
64	YFR004W	cytoplasm;nucleus
65	YDR166C	ambiguous;bud neck;cell periphery;bud
66	YLL040C	cytoplasm;endosome
67	YML057W	cytoplasm
68	YJL005W	ambiguous
69	YBR143C	cytoplasm
70	YDL229W	cytoplasm
71	YDR162C	NONE
72	YMR094W	NONE
73	YDR155C	cytoplasm;nucleus
74	YBR133C	bud neck;cytoplasm
75	YDR328C	cytoplasm;nucleus
76	YBR130C	ambiguous;cytoplasm;cell periphery;bud
77	YLL024C	cytoplasm;nucleus
78	YPR165W	NONE
79	YGR220C	mitochondrion
80	YGR218W	nucleus
81	YDL212W	ER
82	YGL106W	NONE
83	YDL203C	ambiguous

Edges

YAL029C	YBR109C
YAL029C	YBR130C
YAL029C	YGL106W
YAL041W	YBL085W
YAL041W	YBR200W
YAL041W	YER114C
YAL041W	YHL007C
YAL041W	YLR229C
YAL041W	YOR212W
YBL085W	YBR200W
YBL085W	YDR085C
YBL085W	YER124C
YBL085W	YER158C
YBL085W	YFR028C
YBL085W	YHL007C
YBL085W	YJR056C
YBL085W	YML109W
YBL085W	YNL078W
YBR011C	YBR109C
YBR011C	YDL203C
YBR011C	YFR004W
YBR011C	YLR371W
YBR069C	YJR077C
YBR102C	YDR166C
YBR102C	YER008C
YBR102C	YGL233W
YBR102C	YIL068C
YBR102C	YJL085W
YBR102C	YLR166C
YBR102C	YPR032W
YBR102C	YPR055W
YBR109C	YBR130C
YBR109C	YDR155C
YBR109C	YDR356W
YBR109C	YFL004W
YBR109C	YFR004W
YBR109C	YFR014C
YBR109C	YGL106W
YBR109C	YGL242C
YBR109C	YJL012C
YBR109C	YJR104C
YBR109C	YKL042W
YBR109C	YKL129C
YBR109C	YLL040C

YBR109C	YLR433C
YBR109C	YML057W
YBR109C	YMR109W
YBR109C	YNR035C
YBR109C	YOL016C
YBR109C	YOR326W
YBR109C	YPL019C
YBR109C	YPL242C
YBR130C	YGL106W
YBR130C	YPL240C
YBR133C	YHL007C
YBR143C	YFR004W
YBR143C	YLR371W
YBR200W	YDR166C
YBR200W	YER114C
YBR200W	YGL233W
YBR200W	YHL007C
YBR200W	YLR166C
YBR200W	YLR229C
YBR200W	YPR055W
YDL155W	YFR028C
YDL155W	YLR131C
YDL203C	YLR371W
YDL212W	YGR191W
YDL229W	YFR004W
YDL229W	YHL007C
YDL229W	YIL043C
YDL229W	YLL024C
YDL229W	YLR371W
YDL229W	YMR186W
YDL229W	YPL240C
YDL229W	YPR110C
YDR155C	YFR004W
YDR162C	YHL007C
YDR166C	YER008C
YDR166C	YGL233W
YDR166C	YIL068C
YDR166C	YJL085W
YDR166C	YLR166C
YDR166C	YPR055W
YDR328C	YLL024C
YDR328C	YMR094W
YDR328C	YOR057W
YDR328C	YPL256C
YDR356W	YKL042W
YER008C	YGL233W

YER008C	YIL068C
YER008C	YJL085W
YER008C	YLR166C
YER008C	YLR229C
YER008C	YPR055W
YER008C	YPR165W
YER114C	YER124C
YER114C	YER177W
YER114C	YHL007C
YER114C	YML109W
YER124C	YLR362W
YER177W	YFR004W
YFL005W	YFR004W
YFL005W	YGL233W
YFL005W	YGR009C
YFL005W	YOR326W
YFL005W	YPR032W
YFR004W	YIL118W
YFR004W	YJR077C
YFR004W	YLL024C
YFR004W	YML057W
YFR004W	YMR186W
YFR004W	YNR035C
YFR004W	YPL218W
YFR004W	YPL240C
YFR028C	YNL161W
YGL012W	YHL007C
YGL106W	YOR326W
YGL106W	YPL242C
YGL162W	YHL007C
YGL233W	YIL068C
YGL233W	YJL085W
YGL233W	YLR166C
YGL233W	YPR055W
YGL242C	YMR109W
YGR009C	YPL232W
YGR009C	YPR032W
YGR191W	YPL218W
YGR218W	YJR077C
YGR218W	YLR131C
YGR220C	YLR371W
YHL007C	YIL043C
YHL007C	YLR229C
YHL007C	YLR362W
YHL007C	YNL161W
YHL007C	YOR212W

YHL007C	YPL256C
YHR030C	YLR371W
YHR030C	YPL240C
YIL068C	YJL085W
YIL068C	YLR166C
YIL068C	YPR055W
YIL118W	YJL085W
YIL118W	YOR326W
YJL005W	YOR057W
YJL085W	YLR166C
YJL085W	YLR229C
YJL085W	YPR055W
YKL129C	YMR109W
YLL024C	YMR186W
YLL024C	YOR057W
YLR131C	YNL161W
YLR166C	YPR055W
YLR229C	YPL242C
YLR362W	YMR186W
YLR362W	YPL240C
YLR433C	YML057W
YMR094W	YOR057W
YMR186W	YOR057W
YMR186W	YPL240C
YOR057W	YPL240C
YOR326W	YPR032W
YPL232W	YPR032W
YPR032W	YPR110C
