# Supplementary material for: Predicting multiplex subcellular localization of proteins using protein-protein interaction network: a comparative study
Source: BMC Bioinformatics. 2012 Jun 25;13(Suppl 10):S20. doi: 10.1186/1471-2105-13-S10-S20 (PMC3314587; doi:10.1186/1471-2105-13-S10-S20)
Supplement: Additional file 5 — Prediction for the first group of 60 "ambiguous" proteins. [file 1471-2105-13-S10-S20-S5.pdf]

Supplementary Table 5 Prediction for the first group of 60 "ambiguous" annotated proteins.

In this table, "Annotation" denotes the experimentally observed subcellular localizations in Yeast Gtp Fusion Localization Database [17]. Predictions in different color regions correspond to different types: blue (correct), orange (partial correct), red (mismatch) and white (unknown).

ORF	Annotation	Prediction	Uniprot sucellular localization	SGD cellular component
YBL034C	ambiguous;spindle pole	spindle pole	Nucleus. Cytoplasm › cytoskeleton › spindle. Cytoplasm › cytoskeleton. Chromosome › centromere › kinetochore	spindle pole body (IDA)
YDR181C	ambiguous;cytoplasm;nucleus	cytoplasm;nucleus	Nucleus	nuclear chromatin (IDA)<br>nuclear chromosome, telomeric region (IC)<br>SAS acetyltransferase complex (IDA)
YGR020C	ambiguous;vacuolar membrane	vacuolar membrane		fungal-type vacuole membrane (TAS)<br>vacuolar proton-transporting V-type ATPase, V1 domain (TAS)
YHR119W	ambiguous;nucleus	nucleus	Nucleus (Probable). Chromosome (Probable).	Set1C/COMPASS complex (IPI)
YHR183W	ambiguous;cytoplasm	cytoplasm	Cytoplasm	cytoplasm (IDA)<br>mitochondrion (IDA)
YIL053W	ambiguous;cytoplasm;nucleus	cytoplasm;nucleus	Cytoplasm.	cytoplasm (IDA)<br>nucleus (IDA)
YJR104C	ambiguous;cytoplasm;nucleus	cytoplasm;nucleus;bud	Cytoplasm. Mitochondrion intermembrane space.	cytosol (IDA)<br>mitochondrial intermembrane space (IDA)<br>nucleus (IDA)<br>mitochondrion (IDA)

YKL080W	ambiguous;vacuolar membrane	vacuolar membrane	Vacuole membrane; Peripheral membrane protein	fungal-type vacuole membrane (TAS)<br>vacuolar proton-transporting V-type ATPase, V1 domain (TAS)
YLR044C	ambiguous;cytoplasm;nucleus	cytoplasm;nucleus	Cytoplasm. Nucleus	cytosol (IDA)<br>nucleus (IDA)<br>cytoplasm (IDA)
YAL029C	ambiguous;cell periphery;bud neck;cytoplasm;bud	bud	Bud.	cellular bud (IDA)<br>cellular bud tip (IDA)<br>filamentous actin (IDA)<br>mitochondrion (IDA)
YBR102C	ambiguous;cell periphery;bud neck;bud	cytoplasm;bud	Cytoplasmic vesicle › secretory vesicle. Bud. Bud neck.	cellular bud neck (IDA)<br>cellular bud tip (IDA)
YBR130C	ambiguous;cell periphery;cytoplasm;bud	bud		actin cap (TAS)<br>cellular bud tip (IDA)<br>cytoplasm (IDA)
YBR200W	ambiguous;cell periphery;bud neck;bud	actin;cytoplasm;bud	Cytoplasm › cytoskeleton.	cellular bud neck (IDA)<br>cellular bud tip (IDA)<br>incipient cellular bud site (IDA)<br>mating projection tip (TAS)<br>mating projection tip (IDA)
YBR260C	ambiguous;bud neck;cytoplasm;bud	cytoplasm	Cytoplasm.	actin cortical patch (IDA)<br>cellular bud (IDA)<br>mating projection tip (IDA)
YDR166C	ambiguous;cell periphery;bud neck;bud	bud		cellular bud neck (IDA)<br>cellular bud tip (IDA)<br>exocyst (IDA)<br>incipient cellular bud site (IDA)<br>mating projection tip (IDA)
YDR479C	ambiguous;punctate composite;cytoplasm	peroxisome	Peroxisome membrane; Multi-pass membrane protein	peroxisomal membrane (IDA)
YFR016C	ambiguous;cytoplasm;bud	cytoplasm		cellular bud (IDA)<br>cytoplasm (IDA)
YGR041W	ambiguous;bud neck;bud	cell periphery;bud neck	Cell membrane; Multi-pass membrane protein.	cellular bud neck (IDA)<br>plasma membrane (IMP)

YIL068C	ambiguous;cell periphery;bud neck;bud	bud	Cytoplasm.	exocyst (IDA)<br>mating projection tip (IDA)
YLR166C	ambiguous;cell periphery;bud neck;bud	bud		exocyst (IDA)<br>mating projection tip (IDA)
YLR187W	ambiguous;cell periphery;bud neck;cytoplasm;bud	cytoplasm	Cytoplasm. Bud neck. Cell membrane; Peripheral membrane protein; Cytoplasmic side	cellular bud (IDA)<br>cellular bud neck (IDA)<br>cytoplasm (IDA)
YMR192W	ambiguous;bud neck;cytoplasm;bud	actin;cytoplasm		cellular bud neck (IDA)<br>cellular bud tip (IDA)<br>Golgi-associated vesicle (IDA)<br>incipient cellular bud site (IDA)<br>plasma membrane (IDA)<br>cellular bud (IDA)<br>cytoplasm (IDA)<br>mitochondrion (IDA)
YNL272C	ambiguous;bud neck;cytoplasm;bud	cytoplasm	Bud neck. Bud tip. Cytoplasmic vesicle › secretory vesicle.	cellular bud neck (IDA)<br>cellular bud tip (IDA)<br>cytosol (IDA)<br>transport vesicle (IDA)<br>mating projection tip (IDA)
YNL298W	ambiguous;cell periphery;cytoplasm;bud	cytoplasm		actin cap (TAS)<br>cellular bud (IDA)<br>fungal-type vacuole (IDA)<br>nucleus (IMP)
YOR326W	ambiguous;cell periphery;bud neck;cytoplasm;bud	early Golgi;bud	Bud neck. Bud tip.	actin filament bundle (IMP)<br>cellular bud neck (IDA)<br>cellular bud tip (IDA)<br>filamentous actin (IDA)<br>colocalizes_with fungal-type vacuole membrane (IDA)<br>incipient cellular bud site (IDA)<br>mating projection tip (IDA)<br>myosin V complex (ISS)<br>vesicle (IDA)

YPL018W	ambiguous;spindle pole	spindle pole;microtubule	Nucleus. Chromosome › centromere › kinetochore	COMA complex (IDA)<br>condensed nuclear chromosome kinetochore (IDA, IGI)<br>nucleus (IDA)
YPL204W	ambiguous;bud neck;cytoplasm;nucleus;bud	cytoplasm	Cytoplasm. Nucleus › nucleolus. Nucleus › nucleoplasm	cellular bud neck (IDA)<br>cellular bud tip (IDA)<br>chromosome, centromeric region (IDA)<br>monopolin complex (IDA, IMP, IPI)<br>nucleus (IDA)<br>plasma membrane (IDA)<br>spindle pole body (IDA)
YPL249C	ambiguous;cell periphery;bud neck;cytoplasm;bud	actin;cytoplasm	Cytoplasm. Bud. Bud neck	cellular bud neck (IDA)<br>cellular bud tip (IDA)<br>cytosol (IDA)<br>Golgi-associated vesicle (IDA)<br>incipient cellular bud site (IDA)<br>membrane fraction (IDA)<br>plasma membrane (IDA)<br>soluble fraction (IDA)
YPR055W	ambiguous;cell periphery;bud neck;cytoplasm;bud	actin;bud	Cytoplasm. Cell membrane; Peripheral membrane protein.	cellular bud tip (IDA)<br>exocyst (IDA)<br>incipient cellular bud site (TAS)<br>site of polarized growth (IDA)<br>mating projection tip (IDA)
YPR119W	ambiguous;nucleus	cytoplasm;nucleus		cellular bud neck (IDA)<br>cytoplasm (IDA, IMP, ISS)<br>nucleus (IDA)<br>spindle (IDA)<br>spindle pole body (IDA)
YAR019C	ambiguous;spindle pole	cytoplasm		cellular bud neck (TAS)<br>spindle pole body (IDA)
YBL105C	ambiguous;bud neck;cytoplasm;bud	actin		cytoplasm (IDA)<br>cytoskeleton (IDA)<br>nucleus (IDA)

YDL146W	ambiguous;cell periphery;bud neck;cytoplasm;bud	actin	Cytoplasm. Bud. Bud neck	colocalizes_with actin cortical patch (IDA)<br>cellular bud (IDA)<br>cellular bud neck (IDA)<br>cytoplasm (IDA)
YDR164C	ambiguous;bud neck;cytoplasm;bud	lipid particle		cellular bud neck (IDA)<br>cellular bud tip (IDA)<br>plasma membrane (IDA)
YDR309C	ambiguous;cytoplasm;bud	actin	Bud neck (By similarity). Bud tip (By similarity). Cytoplasm › cell cortex (By similarity). Cytoplasm › cytoskeleton (By similarity).	actin cap (TAS)<br>cellular bud tip (IDA)<br>incipient cellular bud site (IDA)<br>mating projection tip (IDA)<br>plasma membrane (IGI)
YDR313C	ambiguous;endosome	cytoplasm	Endosome membrane; Peripheral membrane protein. Vacuole membrane; Peripheral membrane protein	fungal-type vacuole membrane (IDA)<br>late endosome (IDA)
YER005W	ambiguous;Golgi;early Golgi	vacuolar membrane	Golgi apparatus. Membrane; Single-pass membrane protein	membrane (IDA)<br>microsome (IDA)<br>colocalizes_with COPI-coated vesicle (IDA)<br>Golgi apparatus (IDA)
YER149C	ambiguous;cell periphery;bud	cytoplasm		actin cap (TAS)<br>polarisome (TAS)<br>mating projection tip (IDA)
YGR238C	ambiguous;cell periphery;bud	cytoplasm		cellular bud neck (IDA)<br>cellular bud tip (IDA)<br>mating projection tip (IDA)
YGR241C	ambiguous;cell periphery;bud neck;bud	actin	Bud. Bud neck. Cell membrane; Peripheral membrane protein; Cytoplasmic side. Cytoplasm	actin cortical patch (TAS)

YHR158C	ambiguous;cell periphery;bud neck;bud	cytoplasm;nucleus		cellular bud neck (IDA)<br>cellular bud tip (IDA)<br>mating projection tip (IDA)<br>cytoplasm (IDA)
YHR161C	ambiguous;cell periphery;bud neck;bud	actin	Bud. Bud neck. Cell membrane; Peripheral membrane protein; Cytoplasmic side. Cytoplasm	actin cortical patch (TAS)
YIL140W	ambiguous;vacuole;cell periphery;bud neck;bud	cytoplasm	Cell membrane; Single-pass type I membrane protein.	cellular bud neck (IDA)<br>integral to plasma membrane (TAS)<br>septin ring (TAS)
YLR313C	ambiguous;bud neck;bud	cytoplasm	Bud tip. Bud neck. Cytoplasm › cytoskeleton.	cellular bud neck (IDA)<br>cellular bud tip (IDA)<br>incipient cellular bud site (IDA)<br>mating projection (IDA)<br>polarisome (TAS)
YNR049C	ambiguous;bud neck;cytoplasm;bud	lipid particle		cellular bud membrane (IDA)<br>cellular bud neck (IDA)<br>cellular bud tip (IDA)<br>microsome (IDA)<br>plasma membrane (IDA)<br>prospore membrane (IDA)<br>SNARE complex (IDA)
YOL112W	ambiguous;cell periphery;bud neck;bud	cytoplasm	Cytoplasm. Bud. Bud neck.	cellular bud tip (IDA)<br>incipient cellular bud site (IDA)<br>polarisome (IPI)
YPL032C	ambiguous;cell periphery;bud neck;cytoplasm;bud	actin	Cytoplasm. Bud. Bud neck. Cytoplasm › cell cortex.	cellular bud (IDA)<br>cellular bud neck (IDA)<br>cytoplasm (IDA)
YPL269W	ambiguous;spindle pole	microtubule	Nucleus. Cytoplasm › cytoskeleton.	cell cortex (IDA)<br>mating projection tip (IDA)<br>spindle pole body (IDA)

YCL024W	ambiguous;cell periphery;bud neck;bud	Bud neck	cellular bud neck (IDA)<br>cellular bud neck septin collar (IDA)<br>incipient cellular bud site (IDA)<br>septin ring (IDA)
YDL089W	ambiguous;nuclear periphery	Membrane; Multi-pass membrane protein.	nuclear periphery (IDA)
YDR069C	ambiguous;endosome	Cytoplasm. Late endosome membrane; Peripheral membrane protein.	endosome (IDA)<br>membrane fraction (IDA)<br>proteasome complex (IPI)<br>mitochondrion (IDA)
YDR507C	ambiguous;bud neck;cytoplasm;bud	Cytoplasm. Bud neck.	cellular bud neck (IDA)
YDR532C	ambiguous;spindle pole	Cytoplasm › cytoskeleton › spindle pole body. Nucleus membrane; Peripheral membrane protein; Nucleoplasmic side. Chromosome › centromere › kinetochore	chromosome, centromeric region (IDA)<br>condensed nuclear chromosome kinetochore (IDA)<br>colocalizes_with spindle pole body (IDA)
YHL019C	ambiguous;late Golgi	Membrane › coated pit.	AP-1 adaptor complex (IPI)
YKL079W	ambiguous;cytoplasm;bud	Cytoplasm › cytoskeleton (Probable)	cellular bud neck (IDA)<br>cellular bud tip (IDA)<br>incipient cellular bud site (IDA)<br>mating projection tip (IDA)
YLR353W	ambiguous;cell periphery;bud neck;bud	Cell membrane; Multi-pass membrane protein.	cellular bud tip (IDA)<br>incipient cellular bud site (IDA)<br>plasma membrane (IMP)
YMR029C	ambiguous;ER;cytoplasm	Cytoplasm. Endoplasmic reticulum	
YMR124W	ambiguous;cell periphery;bud neck;cytoplasm;bud		cytoplasm (IDA)

YMR163C	ambiguous;punctate composite;bud	Peroxisome membrane; Single-pass membrane protein	integral to peroxisomal membrane (IDA)<br>colocalizes_with peroxisome (IDA)<br>cytoplasm (IDA)
YNL166C	ambiguous;bud neck;cytoplasm;bud	Cytoplasm. Bud.	cellular bud neck (IDA)<br>cellular bud neck septin ring (IDA, IPI)
