# Supplementary material for: Predicting multiplex subcellular localization of proteins using protein-protein interaction network: a comparative study
Source: BMC Bioinformatics. 2012 Jun 25;13(Suppl 10):S20. doi: 10.1186/1471-2105-13-S10-S20 (PMC3314587; doi:10.1186/1471-2105-13-S10-S20)
Supplement: Additional file 6 — Prediction for the 606 proteins without prior knowledge. [file 1471-2105-13-S10-S20-S6.pdf]

Supplementary Table 6 Prediction for the 606 proteins without prior knowledge.

In this table, predictions in different color regions correspond to different types: blue (correct), orange (partial correct), red (mismatch) and white (unknown).

ORF	Prediction	Uniprot subcellular location	SGD cellular component
Q0045	mitochondrion	Mitochondrion inner membrane; Multi-pass membrane protein.	mitochondrion (IDA)
Q0080	mitochondrion	Mitochondrion membrane; Single-pass membrane protein.	mitochondrion (IDA)
Q0085	mitochondrion	Mitochondrion inner membrane; Multi-pass membrane protein.	mitochondrion (IDA)
Q0105	mitochondrion	Mitochondrion inner membrane; Multi-pass membrane protein.	mitochondrion (IDA)
Q0130	mitochondrion	Mitochondrion membrane; Multi-pass membrane protein.(Potential)	mitochondrion (IDA)
Q0250	mitochondrion	Mitochondrion inner membrane; Multi-pass membrane protein.	mitochondrion (IDA)
Q0275	mitochondrion	Mitochondrion inner membrane; Multi-pass membrane protein.(By similarity)	mitochondrion (IDA)
YAL013W	nucleus	Cytoplasm. Nucleus.	nucleus (IC)
YAL020C	cytoplasm		cytoplasm (IDA, IPI)
YAL029C	bud	Bud.	cellular bud (IDA)<br>cellular bud tip (IDA)<br>filamentous actin (IDA)<br>mitochondrion (IDA)
YBL005W-A	cytoplasm	Cytoplasm (By similarity)	
YBL030C	mitochondrion	Mitochondrion inner membrane; Multi-pass membrane protein.	mitochondrial inner membrane (IDA)<br>mitochondrion (IPI)
YBL034C	spindle pole	Nucleus. Cytoplasm › cytoskeleton › spindle. Cytoplasm › cytoskeleton. Chromosome › centromere › kinetochore	spindle pole body (IDA)
YBL041W	cytoplasm;nucleus	Cytoplasm. Nucleus.	endoplasmic reticulum membrane (IC)<br>nucleus (IC)

YBL078C	cytoplasm	Cytoplasmic vesicle › cvt vesicle membrane; Lipid-anchor. Cytoplasmic vesicle › autophagosome membrane; Lipid-anchor. Vacuole membrane; Lipid-anchor	autophagic vacuole (IDA)<br>cytosol (IDA)<br>vacuolar membrane (IDA, IPI)
YBR091C	mitochondrion	Mitochondrion inner membrane; Peripheral membrane protein	mitochondrion (IDA)
YBR102C	cytoplasm;bud	Cytoplasmic vesicle › secretory vesicle. Bud. Bud neck.	cellular bud neck (IDA)<br>cellular bud tip (IDA)
YBR111W-A	nucleus	Nucleus › nucleoplasm. Nucleus › nuclear pore complex	DUBm complex (IDA)<br>nuclear pore (IDA)<br>SAGA complex (IDA)<br>transcription export complex 2 (IDA)
YBR136W	nucleus	Nucleus.	nucleus (IC)<br>mitochondrion (IDA)
YBR146W	mitochondrion	Mitochondrion (Potential)	mitochondrial small ribosomal subunit (IPI)<br>mitochondrion (IDA)
YBR154C	nucleus	Nucleus.	DNA-directed RNA polymerase I complex (IDA)<br>DNA-directed RNA polymerase II, core complex (IDA)<br>DNA-directed RNA polymerase III complex (IDA, TAS)
YBR168W	peroxisome		integral to peroxisomal membrane (IDA)
YBR193C	nucleus	Nucleus (Probable)	mediator complex (IDA)
YBR217W	cytoplasm	Cytoplasm (Probable)	Atg12-Atg5-Atg16 complex (IDA)<br>membrane fraction (IDA)
YBR229C	ER	Endoplasmic reticulum	alpha-glucosidase II complex (IPI)<br>endoplasmic reticulum (ISS)<br>endoplasmic reticulum lumen (IDA)<br>mitochondrion (IDA)
YBR257W	nucleus	Nucleus (Potential)	nucleolar ribonuclease P complex (IDA)<br>ribonuclease MRP complex (IDA)
YBR260C	cytoplasm	Cytoplasm.	actin cortical patch (IDA)<br>cellular bud (IDA)<br>mating projection tip (IDA)
YCL054W	nucleus	Nucleus › nucleolus	nucleolus (IDA)<br>nucleus (IDA)<br>preribosome, large subunit precursor (IDA)
YCR031C	cytoplasm	Cytoplasm (By similarity). Nucleus › nucleolus	90S preribosome (IDA)<br>cytosolic small ribosomal subunit (NAS)<br>small-subunit processome (IDA)
YCR039C	nucleus	Nucleus.	nucleus (TAS)
YCR052W	nucleus	Nucleus.	RSC complex (IDA)
YCR072C	nucleus		nucleolus (IDA)<br>colocalizes_with ribosome (IDA)
YCR097W	nucleus	Nucleus.	nucleus (IDA)

YDL004W	mitochondrion	Mitochondrion. Mitochondrion inner membrane.	mitochondrial proton-transporting ATP synthase, central stalk (IDA, IMP, IPI, ISS)<br>proton-transporting ATP synthase complex, catalytic core F(1) (IDA)<br>mitochondrion (IDA)
YDL007W	cytoplasm;nucleus	Cytoplasm (Potential). Nucleus (Potential)	nucleus (IDA)<br>proteasome regulatory particle, base subcomplex (IDA)
YDL031W	nucleolus;nucleus	Nucleus › nucleolus	nucleolus (IDA)
YDL075W	cytoplasm	Cytoplasm (By similarity).	cytosolic large ribosomal subunit (IDA)
YDL143W	cytoplasm	Cytoplasm.	chaperonin-containing T-complex (IDA, IPI)
YDL153C	nucleolus	Nucleus › nucleolus	nucleolus (IDA)<br>nucleus (IDA)<br>small-subunit processome (IDA)
YDL217C	mitochondrion	Mitochondrion inner membrane; Multi-pass membrane protein.	mitochondrial inner membrane protein insertion complex (IDA)<br>mitochondrion (IDA)
YDR045C	nucleus	Nucleus (Probable).	DNA-directed RNA polymerase III complex (IDA)
YDR064W	cytoplasm	Cytoplasm (By similarity).	90S preribosome (IDA)<br>cytosolic small ribosomal subunit (IDA)
YDR113C	cytoplasm;nucleus	Cytoplasm. Nucleus.	cytoplasm (IDA)<br>nucleus (IDA)<br>spindle (IDA)
YDR143C	cytoplasm		cytoplasm (IMP)<br>nucleus (IDA)
YDR188W	cytoplasm	Cytoplasm.	chaperonin-containing T-complex (IDA, IPI)
YDR237W	mitochondrion	Mitochondrion.	mitochondrial large ribosomal subunit (IDA)<br>mitochondrion (IDA)
YDR260C	cytoplasm;nucleus		anaphase-promoting complex (IDA)<br>nucleus (IDA)
YDR285W	nucleus	Nucleus. Chromosome.	transverse filament (IDA)
YDR289C	nucleus	Nucleus.	
YDR341C	cytoplasm	Cytoplasm.	cytoplasm (NAS)<br>mitochondrion (IDA)
YDR359C	nucleus	Nucleus (Probable).	histone acetyltransferase complex (IPI)<br>NuA4 histone acetyltransferase complex (IPI)
YDR378C	cytoplasm;nucleus	Cytoplasm. Nucleus › nucleolus.	cytoplasmic mRNA processing body (IDA)<br>nucleolus (IDA)<br>small nucleolar ribonucleoprotein complex (IPI)<br>U4/U6 x U5 tri-snRNP complex (IDA)<br>U6 snRNP (IDA, TAS)
YDR383C	spindle pole	Nucleus. Chromosome › centromere › kinetochore.	kinetochore (IDA)
YDR394W	cytoplasm;nucleus	Cytoplasm (Potential). Nucleus (Potential).	proteasome regulatory particle, base subcomplex (IDA)

YDR468C	early Golgi	Golgi apparatus › trans-Golgi network membrane; Single-pass type IV membrane protein (Probable). Early endosome membrane; Single-pass type IV membrane protein (Probable). Late endosome membrane; Single-pass type IV membrane protein (Probable).	early endosome (TAS)<br>Golgi trans cisterna (TAS)<br>SNARE complex (IPI)<br>trans-Golgi network (TAS)
YDR470C	mitochondrion	Mitochondrion outer membrane; Single-pass type II membrane protein	mitochondrial outer membrane (IDA)<br>mitochondrion (IDA)
YDR479C	peroxisome	Peroxisome membrane; Multi-pass membrane protein	peroxisomal membrane (IDA)
YDR489W	nucleus	Nucleus.	DNA replication preinitiation complex (IGI, IMP)<br>GINS complex (IPI)<br>replication fork protection complex (IDA)
YEL020W-A	mitochondrion	Mitochondrion inner membrane; Peripheral membrane protein; Intermembrane side	mitochondrial inner membrane protein insertion complex (IDA)<br>mitochondrial intermembrane space protein transporter complex (IDA)<br>mitochondrion (IDA)
YEL034W	cytoplasm	Cytoplasm.	cytosolic ribosome (IPI)<br>cytoplasm (IDA)<br>mitochondrion (IDA)
YER012W	cytoplasm;nucleus	Cytoplasm. Nucleus.	endoplasmic reticulum membrane (IDA)<br>nucleus (IDA)<br>proteasome core complex, beta-subunit complex (IDA)<br>proteasome storage granule (IC)
YER041W	cytoplasm;nucleus		cytoplasm (IDA)<br>nucleus (IDA)
YER050C	mitochondrion	Mitochondrion.	mitochondrial small ribosomal subunit (IPI)<br>mitochondrion (IDA)
YER051W	nucleus	Nucleus (By similarity)	
YER087C-B	ER	Endoplasmic reticulum membrane; Single-pass membrane protein.	Sec61 translocon complex (IDA)
YER094C	cytoplasm;nucleus	Cytoplasm. Nucleus.	endoplasmic reticulum membrane (IC)<br>nucleus (IC)<br>proteasome core complex, beta-subunit complex (IDA)<br>proteasome storage granule (IC)
YER127W	nucleolus	Nucleus › nucleolus.	small nuclear ribonucleoprotein complex (TAS)<br>small nucleolar ribonucleoprotein complex (TAS)
YFL022C	cytoplasm	Cytoplasm.	cytoplasm (TAS)<br>phenylalanine-tRNA ligase complex (IDA)

YFL038C	cytoplasm;early Golgi	Endoplasmic reticulum membrane; Peripheral membrane protein. Golgi apparatus membrane; Peripheral membrane protein. Cytoplasm.	endoplasmic reticulum membrane (IDA)<br>Golgi membrane (IDA)<br>Golgi stack (IDA)<br>pre-autophagosomal structure (IDA)<br>trans-Golgi network (IDA)<br>mitochondrion (IDA)
YGL003C	cytoplasm;nucleus	Cytoplasm. Nucleus.	anaphase-promoting complex (IPI)<br>cytoplasm (IDA)<br>nucleus (IDA)
YGL044C	nucleus	Nucleus.	mRNA cleavage factor complex (IPI)
YGL205W	peroxisome	Peroxisome.	peroxisomal matrix (TAS)
YGR004W	peroxisome		integral to peroxisomal membrane (IDA)
YGR005C	nucleus	Nucleus.	transcription factor TFIIF complex (TAS)
YGR020C	vacuolar membrane		fungal-type vacuole membrane (TAS)<br>vacuolar proton-transporting V-type ATPase, V1 domain (TAS)
YGR054W	cytoplasm		cytosolic ribosome (IDA)<br>cytosolic small ribosomal subunit (IDA)<br>colocalizes_with ribosome (IDA)
YGR056W	nucleus	Nucleus.	RSC complex (IDA)
YGR077C	peroxisome	Peroxisome matrix (Potential).	extrinsic to intraperoxisomal membrane (IDA)<br>peroxisomal matrix (TAS)<br>peroxisomal membrane (IDA)
YGR113W	spindle pole;microtubule	Nucleus. Cytoplasm › cytoskeleton › spindle. Chromosome › centromere › kinetochore.	condensed nuclear chromosome kinetochore (IDA, IPI)<br>DASH complex (IDA, IPI)<br>kinetochore microtubule (IDA)<br>spindle (IPI)<br>spindle midzone (IDA)
YGR145W	nucleolus	Nucleus › nucleolus	90S preribosome (IDA)<br>nucleolus (IDA)
YGR181W	mitochondrion	Mitochondrion inner membrane; Peripheral membrane protein; Intermembrane side	mitochondrial intermembrane space (IDA)<br>mitochondrial intermembrane space protein transporter complex (IDA, IPI)<br>mitochondrion (IDA)
YGR196C	cytoplasm		cytoplasm (IDA)
YGR245C	nucleus	Nucleus › nucleolus	nucleus (IDA)
YHL010C	cytoplasm	Cytoplasm (By similarity).	
YHL015W	cytoplasm	Cytoplasm (By similarity).	cytosolic small ribosomal subunit (IDA)
YHL024W	cytoplasm		cytoplasm (IDA)
YHR005C-A	mitochondrion	Mitochondrion inner membrane; Peripheral membrane protein; Intermembrane side	mitochondrial inner membrane protein insertion complex (IDA)<br>mitochondrial intermembrane space protein transporter complex (IDA)<br>mitochondrion (IDA)

YHR012W	endosome		endosome (IPI)<br>retromer complex (IMP, IPI)<br>retromer complex, inner shell (IPI)
YHR014W	nucleus	Nucleus (Potential).	condensed nuclear chromosome, centromeric region (IDA)<br>nucleus (IDA)
YHR016C	actin	Cytoplasm › cytoskeleton › actin patch.	actin cortical patch (IDA)
YHR056C	nucleus	Nucleus.	RSC complex (IDA)
YHR071W	cytoplasm;nucleus		cyclin-dependent protein kinase holoenzyme complex (IPI)<br>nucleus (IDA)
YHR079C-A	nucleus	Nucleus.	condensed nuclear chromosome (IDA)
YHR099W	nucleus	Nucleus (Probable)	histone acetyltransferase complex (IPI)<br>NuA4 histone acetyltransferase complex (IPI)<br>nucleus (IDA)<br>SAGA complex (IDA)<br>SLIK (SAGA-like) complex (IDA)<br>ASTRA complex (IDA)
YHR115C	cytoplasm	Cytoplasm	cytoplasm (IDA)
YHR119W	nucleus	Nucleus (Probable). Chromosome (Probable).	Set1C/COMPASS complex (IPI)
YHR148W	nucleolus	Nucleus › nucleolus	90S preribosome (IDA)<br>Mpp10 complex (IDA)<br>small-subunit processome (IDA, IMP)
YHR160C	peroxisome	Cytoplasm. Peroxisome membrane; Peripheral membrane protein; Cytoplasmic side	cytosol (IDA)<br>peroxisome (IDA)
YHR171W	cytoplasm	Cytoplasm	cytosol (IDA)<br>membrane (IDA)<br>pre-autophagosomal structure (IDA)<br>mitochondrion (IDA)
YHR183W	cytoplasm	Cytoplasm	cytoplasm (IDA)<br>mitochondrion (IDA)
YHR191C	nucleus	Nucleus.	Ctf18 RFC-like complex (IPI)
YHR199C-A	microtubule;nucleus	Nucleus. Cytoplasm › cytoskeleton › spindle.	chromosome passenger complex (IDA)
YIL004C	ER to Golgi;early Golgi	Golgi apparatus membrane; Single-pass type IV membrane protein (Probable). Endoplasmic reticulum membrane; Single-pass type IV membrane protein (Probable).	endoplasmic reticulum membrane (IDA)<br>ER to Golgi transport vesicle (IDA)<br>integral to membrane (IDA)<br>SNARE complex (IDA)
YIL053W	cytoplasm;nucleus	Cytoplasm.	cytoplasm (IDA)<br>nucleus (IDA)
YIL065C	mitochondrion	Mitochondrion outer membrane; Single-pass membrane protein	mitochondrial outer membrane (IDA)<br>peroxisome (IDA)<br>mitochondrion (IDA)
YIL142W	cytoplasm	Cytoplasm.	chaperonin-containing T-complex (IDA, IPI)<br>cytoplasm (IDA)

YIL160C	peroxisome	Peroxisome.	peroxisomal matrix (TAS)<br>peroxisome (IDA)
YJL008C	cytoplasm	Cytoplasm (By similarity).	chaperonin-containing T-complex (IDA, IPI)<br>cytoplasm (IDA)
YJL014W	cytoplasm	Cytoplasm.	chaperonin-containing T-complex (IDA, IPI)
YJL019W	nucleus	Nucleus membrane; Single-pass type II membrane protein. Cytoplasm › cytoskeleton › spindle pole body.	half bridge of spindle pole body (IDA)<br>integral to membrane (IDA)<br>nuclear chromosome, telomeric region (IDA)<br>nuclear envelope (IDA)<br>nuclear periphery (IDA)
YJL020C	actin	Cytoplasm › cytoskeleton › actin patch.	actin cortical patch (IDA)
YJL111W	cytoplasm	Cytoplasm (By similarity).	chaperonin-containing T-complex (IDA, IPI)<br>cytoplasm (IDA)
YJL141C	cytoplasm;nucleus	Cytoplasm. Nucleus.	cytoplasm (IDA)<br>nucleus (IDA)
YJR045C	mitochondrion	Mitochondrion matrix. Nucleus.	mitochondrial inner membrane (IDA)<br>mitochondrial nucleoid (IDA)<br>presequence translocase-associated import motor (IDA)<br>TRC complex (IDA)<br>mitochondrion (IDA)
YJR064W	cytoplasm	Cytoplasm.	chaperonin-containing T-complex (IDA, IPI)
YJR065C	actin	Cytoplasm › cytoskeleton › actin patch	Arp2/3 protein complex (IDA)<br>plasma membrane enriched fraction (IDA)
YJR090C	cytoplasm	Membrane.	cellular bud neck contractile ring (IDA)<br>cytoplasm (IDA)<br>nucleus (IDA)<br>SCF ubiquitin ligase complex (IMP)
YJR093C	nucleus	Nucleus	mRNA cleavage and polyadenylation specificity factor complex (IPI)
YJR099W	cytoplasm		cytoplasm (IC)
YJR135W-A	mitochondrion	Mitochondrion inner membrane; Peripheral membrane protein; Intermembrane side.	mitochondrial intermembrane space protein transporter complex (IDA, IPI)<br>mitochondrion (IDA)
YKL005C	nucleus	Nucleus (By similarity).	nucleus (TAS)
YKL018W	nucleus	Nucleus. Chromosome › telomere	mRNA cleavage and polyadenylation specificity factor complex (IPI)<br>Set1C/COMPASS complex (IPI)
YKL080W	vacuolar membrane	Vacuole membrane; Peripheral membrane protein	fungal-type vacuole membrane (TAS)<br>vacuolar proton-transporting V-type ATPase, V1 domain (TAS)
YKL084W	mitochondrion	Mitochondrion intermembrane space. Mitochondrion membrane; Peripheral membrane protein	mitochondrial intermembrane space (IDA)

YKL138C-A	spindle pole	Nucleus. Cytoplasm › cytoskeleton › spindle. Chromosome › centromere › kinetochore.	DASH complex (IDA, IPI)<br>kinetochore (IPI)<br>spindle (IDA)
YKL144C	nucleus	Nucleus.	DNA-directed RNA polymerase III complex (IDA)
YKL159C	cytoplasm		cytoplasm (IC)
YKL171W	cytoplasm		cytoplasm (IDA)
YKR028W	cytoplasm	Cytoplasm (Probable).	cytoplasm (IPI)
YKR030W	Golgi	Golgi apparatus membrane; Multi-pass membrane protein. Endoplasmic reticulum membrane; Multi-pass membrane protein	integral to Golgi membrane (IDA)
YLL004W	nucleus	Nucleus.	DNA replication preinitiation complex (IDA)<br>nuclear origin of replication recognition complex (IDA, IMP)<br>pre-replicative complex (IDA)
YLL042C	cytoplasm	Cytoplasm (Probable).	
YLR008C	mitochondrion	Mitochondrion inner membrane; Single-pass membrane protein	mitochondrial inner membrane presequence translocase complex (IDA)<br>presequence translocase-associated import motor (IDA)<br>mitochondrion (IDA)
YLR026C	ER to Golgi;early Golgi	Membrane; Single-pass type IV membrane protein (Potential). Golgi apparatus membrane; Single-pass type IV membrane protein (Potential).	cis-Golgi network (IDA)<br>integral to membrane (IDA)<br>SNARE complex (IDA)
YLR044C	cytoplasm;nucleus	Cytoplasm. Nucleus	cytosol (IDA)<br>nucleus (IDA)<br>cytoplasm (IDA)
YLR096W	cytoplasm	Cytoplasm. Cell membrane; Peripheral membrane protein; Cytoplasmic side	plasma membrane (IDA, IPI)
YLR229C	cytoplasm;bud	Cell membrane; Lipid-anchor; Cytoplasmic side (Potential).	cellular bud neck (TAS)<br>cellular bud tip (TAS)<br>mating projection tip (IDA)<br>plasma membrane (IDA)<br>soluble fraction (IDA)
YLR238W	ER	Membrane; Single-pass type IV membrane protein (Potential).	endoplasmic reticulum (IDA)
YLR274W	cytoplasm;nucleus	Nucleus.	cytoplasm (IDA)<br>DNA replication preinitiation complex (IPI)<br>MCM complex (IDA)<br>nuclear chromosome, telomeric region (IDA)<br>nucleus (IDA)<br>pre-replicative complex (IDA)<br>replication fork protection complex (IDA)

YLR340W	cytoplasm	Cytoplasm (By similarity).	90S preribosome (IDA)<br>cytoplasm (IDA)<br>cytosolic large ribosomal subunit (TAS)<br>colocalizes_with preribosome, large subunit precursor (IDA)
YLR437C	cytoplasm;nucleus	Cytoplasm. Nucleus	cytoplasm (IDA)
YML015C	nucleus	Nucleus.	transcription factor TFIID complex (IDA)
YML019W	ER	Endoplasmic reticulum membrane; Multi-pass membrane protein (Probable).	oligosaccharyltransferase complex (IPI)
YML042W	peroxisome	Peroxisome. Mitochondrion inner membrane; Peripheral membrane protein; Matrix side.	mitochondrion (IDA)
YML065W	nucleus	Nucleus.	DNA replication preinitiation complex (IDA)<br>nuclear origin of replication recognition complex (IDA, IMP)<br>nucleus (IDA)<br>pre-replicative complex (IDA)
YML077W	late Golgi	Golgi apparatus › cis-Golgi network. Endoplasmic reticulum (Probable).	TRAPP complex (IDA)
YMR005W	nucleus	Nucleus	transcription factor TFIID complex (IDA)
YMR025W	cytoplasm	Cytoplasm (Probable). Nucleus (Probable).	signalosome (IDA)
YMR033W	nucleus	Nucleus.	nucleus (IDA)<br>RSC complex (IDA)<br>SWI/SNF complex (IDA, IPI)
YMR068W	cytoplasm	Cell membrane; Peripheral membrane protein; Cytoplasmic side. Vacuole membrane; Peripheral membrane protein; Cytoplasmic side	TORC2 complex (IPI)<br>cytoplasm (IDA)
YMR094W	microtubule;nucleus;bud	Nucleus. Chromosome › centromere.	CBF3 complex (IDA)<br>condensed nuclear chromosome kinetochore (TAS)
YMR116C	cytoplasm	Cytoplasm.	cytosolic small ribosomal subunit (IDA)<br>cytoplasm (IDA)
YMR146C	cytoplasm	Cytoplasm (Probable).	eukaryotic translation initiation factor 3 complex (IDA, IPI)<br>multi-eIF complex (IDA)
YMR158W	mitochondrion	Mitochondrion	mitochondrial small ribosomal subunit (IPI)<br>mitochondrion (IDA)
YMR203W	mitochondrion	Mitochondrion outer membrane; Multi-pass membrane protein.	mitochondrial outer membrane translocase complex (IDA)<br>mitochondrial outer membrane (IDA)<br>mitochondrion (IDA)
YMR217W	cytoplasm	Cytoplasm (By similarity).	

YMR290C	nucleolus;nucleus	Nucleus › nucleolus	90S preribosome (IDA)<br>nuclear envelope (IDA)<br>nucleolus (IDA)<br>preribosome, large subunit precursor (IDA)
YMR298W	ER	Endoplasmic reticulum membrane; Single-pass type II membrane protein	endoplasmic reticulum membrane (IDA)<br>endoplasmic reticulum (IDA)<br>nuclear envelope (IDA)
YMR316W	cytoplasm	Cytoplasm	cytoplasm (IDA)
YNL023C	cytoplasm	Cytoplasm. Nucleus.	cytoplasm (IDA)
YNL026W	mitochondrion	Mitochondrion outer membrane; Multi-pass membrane protein (By similarity).	integral to mitochondrial outer membrane (IDA)<br>mitochondrial sorting and assembly machinery complex (IDA)<br>mitochondrial outer membrane (IDA)<br>mitochondrion (IDA)
YNL031C	nucleus	Nucleus. Chromosome.	nuclear nucleosome (TAS)
YNL047C	cytoplasm	Cell membrane; Peripheral membrane protein; Cytoplasmic side	plasma membrane (IDA)
YNL069C	cytoplasm	Cytoplasm (By similarity).	cytosolic large ribosomal subunit (TAS)
YNL131W	mitochondrion	Mitochondrion outer membrane; Single-pass type II membrane protein.	integral to mitochondrial outer membrane (IDA)<br>mitochondrial outer membrane translocase complex (IDA)<br>mitochondrial outer membrane (IDA)<br>mitochondrion (IDA)
YNL148C	microtubule;cytoplasm	Cytoplasm. Cytoplasm › cytoskeleton.	cytoplasm (IDA)<br>nucleus (IDA)
YNL151C	nucleus	Nucleus.	DNA-directed RNA polymerase III complex (IDA)
YNL178W	cytoplasm	Cytoplasm (By similarity).	90S preribosome (IDA)<br>cytosolic small ribosomal subunit (IDA)<br>preribosome, small subunit precursor (IDA)
YNL182C	nucleus	Nucleus	nucleoplasm (IDA)<br>nucleus (IDA)
YNL210W	nucleus		nucleus (IDA)<br>colocalizes_with U1 snRNP (IDA)
YNL222W	nucleus	Nucleus	mRNA cleavage and polyadenylation specificity factor complex (IPI)<br>nucleus (IGI, IPI)
YNL253W	nucleus	Nucleus (Probable).	transcription export complex (IPI)
YNL260C	cytoplasm;nucleus		cytoplasm (IDA)<br>nucleus (IDA)
YNL263C	early Golgi	Endoplasmic reticulum membrane; Multi-pass membrane protein. Golgi apparatus membrane; Multi-pass membrane protein. Cytoplasmic vesicle › COPII-coated vesicle.	endoplasmic reticulum membrane (IPI)<br>ER to Golgi transport vesicle (IDA)<br>integral to Golgi membrane (IDA)

YNL321W	early Golgi	Vacuole membrane; Multi-pass membrane protein	endoplasmic reticulum membrane (IDA)<br>fungal-type vacuole (IDA)<br>fungal-type vacuole membrane (IDA)
YNR003C	nucleus	Nucleus.	DNA-directed RNA polymerase III complex (IDA)<br>mitochondrion (IPI)<br>cytoplasm (IDA)<br>nucleus (IDA)
YNR037C	mitochondrion	Mitochondrion	mitochondrial small ribosomal subunit (IPI)<br>mitochondrion (IDA)
YOL018C	early Golgi	Golgi apparatus › trans-Golgi network membrane; Single-pass type IV membrane protein. Endosome membrane; Single-pass type IV membrane protein.	early endosome (TAS)<br>Golgi trans cisterna (TAS)<br>SNARE complex (IPI)<br>trans-Golgi network (TAS)
YOL086C	cytoplasm;nucleus	Cytoplasm.	cytosol (TAS)<br>plasma membrane enriched fraction (IDA)
YOL104C	nucleus	Nucleus. Chromosome › telomere	nuclear chromosome, telomeric region (IDA)
YOL120C	cytoplasm	Cytoplasm	cytosolic large ribosomal subunit (TAS)
YOL133W	cytoplasm;nucleus	Cytoplasm. Nucleus	cytoplasm (IDA)<br>nucleus (IDA)<br>SCF ubiquitin ligase complex (IDA, IPI)
YOR066W	cytoplasm;nucleus		cytoplasm (IDA)<br>nucleus (IDA)
YOR085W	ER	Endoplasmic reticulum membrane; Multi-pass membrane protein (Probable).	integral to membrane (IMP)<br>oligosaccharyltransferase complex (IPI)
YOR145C	cytoplasm	Cytoplasm. Nucleus › nucleolus	90S preribosome (IDA)<br>nucleolus (IDA)<br>nucleus (IDA)
YOR194C	nucleus	Nucleus.	transcription factor TFIIA complex (IDA)
YOR204W	cytoplasm	Cytoplasm.	cytoplasm (IC)
YOR211C	mitochondrion	Mitochondrion inner membrane; Single-pass type II membrane protein; Intermembrane side	extrinsic to mitochondrial inner membrane (IDA)<br>intrinsic to mitochondrial inner membrane (IDA)<br>mitochondrial crista (IDA)<br>mitochondrial inner boundary membrane (IDA)<br>mitochondrial intermembrane space (IDA)
YOR294W	nucleus	Nucleus	nucleolus (IDA)<br>nucleoplasm (IDA)<br>preribosome, large subunit precursor (IDA)
YOR369C	cytoplasm	Cytoplasm (By similarity).	cytosolic small ribosomal subunit (IDA)
YPL018W	spindle pole;microtubule	Nucleus. Chromosome › centromere › kinetochore	COMA complex (IDA)<br>condensed nuclear chromosome kinetochore (IDA, IGI)<br>nucleus (IDA)

YPL042C	nucleus	Nucleus (Probable).	mediator complex (IDA)
YPL143W	cytoplasm	Cytoplasm (By similarity).	cytosolic large ribosomal subunit (IDA)
YPL149W	cytoplasm	Cytoplasm. Membrane; Peripheral membrane protein. Preautophagosomal structure membrane; Peripheral membrane protein.	Atg12-Atg5-Atg16 complex (IDA)<br>autophagic vacuole (IDA)<br>cytosol (IDA)<br>pre-autophagosomal structure (IDA)
YPL173W	mitochondrion	Mitochondrion	mitochondrial large ribosomal subunit (IDA, IPI)<br>mitochondrion (IDA)
YPL201C	cytoplasm;nucleus	Cytoplasm. Nucleus	cytosol (IDA)<br>nucleus (IDA)
YPL235W	nucleus	Nucleus › nucleoplasm	chromatin remodeling complex (IDA)<br>Ino80 complex (IPI)<br>nucleus (IDA)<br>Swr1 complex (IDA)<br>ASTRA complex (IDA)
YPL237W	cytoplasm		eukaryotic translation initiation factor 2 complex (IMP, ISS)<br>multi-eIF complex (IDA)<br>ribosome (TAS)
YPL248C	nucleus	Nucleus.	nucleus (IDA)
YPL253C	microtubule;nucleus	Cytoplasm › cytoskeleton › spindle pole body. Nucleus	kinesin complex (IDA)<br>spindle pole body (IDA)
YPL268W	microtubule		chromosome, centromeric region (IDA)<br>condensed nuclear chromosome kinetochore (IPI)
YPL269W	microtubule	Nucleus. Cytoplasm › cytoskeleton.	cell cortex (IDA)<br>mating projection tip (IDA)<br>spindle pole body (IDA)
YPR007C	nucleus	Nucleus. Chromosome. Chromosome › centromere.	condensed nuclear chromosome (IPI)<br>condensed nuclear chromosome, centromeric region (IDA, IPI)
YPR018W	nucleus	Nucleus.	CAF-1 complex (IDA)<br>chromosome, centromeric region (IDA)<br>nucleus (IDA)
YPR068C	nucleus	Nucleus (By similarity).	histone deacetylase complex (TAS)
YPR133C	nucleus	Nucleus	transcription elongation factor complex (IPI)
YPR133W-A	mitochondrion	Mitochondrion outer membrane; Single-pass membrane protein (Potential).	integral to mitochondrial outer membrane (IDA)<br>mitochondrial outer membrane translocase complex (IDA)<br>mitochondrial outer membrane (IDA)<br>mitochondrion (IDA)

YPR181C	ER to Golgi	Cytoplasm. Cytoplasmic vesicle › COPII-coated vesicle membrane; Peripheral membrane protein; Cytoplasmic side. Endoplasmic reticulum membrane; Peripheral membrane protein; Cytoplasmic side. Golgi apparatus membrane; Peripheral membrane protein; Cytoplasmic side (Potential).	COPII vesicle coat (IDA)
YPR193C	cytoplasm		cytoplasm (IDA)
YAL042W	ER	Endoplasmic reticulum membrane; Multi-pass membrane protein. Golgi apparatus membrane; Multi-pass membrane protein	ER to Golgi transport vesicle (IDA)<br>integral to endoplasmic reticulum membrane (IDA)<br>integral to Golgi membrane (IDA)
YBL088C	cytoplasm;nucleus	Nucleus. Chromosome › telomere	nucleus (IC)<br>mitochondrion (IDA)
YBR020W	cytoplasm;nucleus		cytoplasm (IGI)
YBR072W	cytoplasm		cytoplasm (IDA)<br>nucleus (IDA)
YBR098W	cytoplasm;nucleus	Nucleus	nucleus (ISS)
YBR108W	actin;cytoplasm	Membrane raft; Peripheral membrane protein	actin cortical patch (IDA)<br>colocalizes_with membrane raft (IDA)
YBR130C	bud		actin cap (TAS)<br>cellular bud tip (IDA)<br>cytoplasm (IDA)
YBR182C	cytoplasm	Nucleus (Potential)	cytoplasm (IDA)<br>nucleus (IDA)
YBR200W	actin;cytoplasm;bud	Cytoplasm › cytoskeleton.	cellular bud neck (IDA)<br>cellular bud tip (IDA)<br>incipient cellular bud site (IDA)<br>mating projection tip (TAS)<br>mating projection tip (IDA)
YBR228W	cytoplasm;nucleus	Nucleus	nucleus (IC)<br>Slx1-Slx4 complex (IPI)
YCR066W	cytoplasm;nucleus	Nucleus	nuclear chromatin (IDA)<br>nucleus (IDA)
YDL006W	cytoplasm		cytoplasm (IDA)<br>nucleus (IDA)
YDL013W	microtubule;nucleus	Nucleus › nucleolus	nucleus (IDA)<br>ubiquitin ligase complex (IDA)
YDL127W	actin;cytoplasm;nucleus	Cytoplasm. Nucleus.	cellular bud neck (IDA)<br>cellular bud tip (IDA)<br>cyclin-dependent protein kinase holoenzyme complex (IPI)<br>incipient cellular bud site (IDA)<br>nucleus (IDA)
YDL146W	actin	Cytoplasm. Bud. Bud neck	colocalizes_with actin cortical patch (IDA)<br>cellular bud (IDA)<br>cellular bud neck (IDA)<br>cytoplasm (IDA)
YDL200C	cytoplasm;nucleus	Nucleus.	nucleus (IC)

YDR013W	cytoplasm;nucleus	Nucleus.	DNA replication preinitiation complex (IGI, IMP)<br>GINS complex (IPI)<br>replication fork protection complex (IDA)
YDR014W-A	cytoplasm;nucleus	Nucleus. Chromosome.	condensed nuclear chromosome (IDA)
YDR037W	cytoplasm;nucleus	Cytoplasm.	cytoplasm (IDA)
YDR082W	cytoplasm;nucleus	Chromosome › telomere (Probable)	nuclear telomere cap complex (IPI)
YDR085C	cytoplasm;bud		mating projection base (IDA)
YDR142C	peroxisome	Cytoplasm. Peroxisome.	cytosol (IDA)<br>peroxisome (IDA)
YDR162C	cytoplasm;bud	Cytoplasm	cytoplasm (IDA, TAS)<br>nucleus (TAS)
YDR166C	bud		cellular bud neck (IDA)<br>cellular bud tip (IDA)<br>exocyst (IDA)<br>incipient cellular bud site (IDA)<br>mating projection tip (IDA)
YDR181C	cytoplasm;nucleus	Nucleus	nuclear chromatin (IDA)<br>nuclear chromosome, telomeric region (IC)<br>SAS acetyltransferase complex (IDA)
YDR212W	cytoplasm;nucleus	Cytoplasm.	chaperonin-containing T-complex (IDA, IPI)<br>plasma membrane enriched fraction (IDA)
YDR223W	nucleus	Cytoplasm. Nucleus.	cytoplasm (IDA)<br>nuclear chromatin (IDA)<br>nucleus (IDA)
YDR255C	cytoplasm;nucleus	Cytoplasm.	cytosol (IDA)<br>GID complex (IDA)
YDR309C	actin	Bud neck (By similarity). Bud tip (By similarity). Cytoplasm › cell cortex (By similarity). Cytoplasm › cytoskeleton (By similarity).	actin cap (TAS)<br>cellular bud tip (IDA)<br>incipient cellular bud site (IDA)<br>mating projection tip (IDA)<br>plasma membrane (IGI)
YDR386W	cytoplasm;nucleus	Nucleus	Holliday junction resolvase complex (IDA)<br>nucleus (IMP, IPI)
YDR446W	cytoplasm;nucleus	Nucleus.	nucleus (IDA)
YEL026W	nucleus	Nucleus › nucleolus.	box C/D snoRNP complex (IDA)<br>nucleolus (IDA)<br>small-subunit processome (IDA)<br>U4/U6 x U5 tri-snRNP complex (IDA)
YEL032W	cytoplasm;nucleus	Nucleus.	cytoplasm (IDA)<br>DNA replication preinitiation complex (IPI)<br>MCM complex (IDA)<br>nucleus (IDA)<br>pre-replicative complex (IDA)<br>replication fork protection complex (IDA)
YEL056W	nucleus	Cytoplasm. Nucleus.	cytoplasm (IDA)<br>histone acetyltransferase complex (IDA, IPI)<br>nucleus (IDA)

YER005W	vacuolar membrane	Golgi apparatus. Membrane; Single-pass membrane protein	membrane (IDA)<br>microsome (IDA)<br>colocalizes_with COPI-coated vesicle (IDA)<br>Golgi apparatus (IDA)
YER007W	microtubule;cytoplasm	Cytoplasm › cytoskeleton (Probable)	
YER070W	cytoplasm;nucleus	Cytoplasm	cytoplasm (IDA)<br>ribonucleoside-diphosphate reductase complex (IDA)
YER093C	cytoplasm	Cell membrane; Peripheral membrane protein; Cytoplasmic side. Vacuole membrane; Peripheral membrane protein; Cytoplasmic side	membrane fraction (IDA)<br>TORC2 complex (IPI)
YER125W	actin;cytoplasm	Cytoplasm (Potential). Nucleus (Potential).	cellular bud tip (IDA)<br>cytoplasm (IDA)<br>endosome membrane (IDA)<br>extrinsic to internal side of plasma membrane (IDA)<br>Golgi apparatus (IDA)<br>nucleus (IDA)<br>ubiquitin ligase complex (IPI)<br>mitochondrion (IDA)<br>plasma membrane enriched fraction (IDA)
YER146W	cytoplasm;nucleus	Nucleus.	nucleolus (IDA)<br>small nucleolar ribonucleoprotein complex (IPI)<br>U4/U6 x U5 tri-snRNP complex (IDA)<br>U6 snRNP (IDA, TAS)
YFL005W	cytoplasm;early Golgi;bud	Cytoplasmic vesicle › secretory vesicle membrane; Lipid-anchor; Cytoplasmic side. Cell membrane; Lipid-anchor; Cytoplasmic side. Cytoplasm.	actin cap (TAS)<br>incipient cellular bud site (IDA)<br>transport vesicle (TAS)<br>vesicle (IDA)<br>mitochondrial outer membrane (IDA)<br>mitochondrion (IDA)<br>plasma membrane enriched fraction (IDA)
YFL009W	cytoplasm;nucleus	Nucleus	nuclear matrix (IDA)<br>nuclear SCF ubiquitin ligase complex (IDA)<br>nucleus (IDA)<br>SCF ubiquitin ligase complex (IDA, IPI)
YFL037W	microtubule;cytoplasm	Cytoplasm › cytoskeleton.	cytoplasmic microtubule (TAS)<br>kinetochore microtubule (TAS)<br>nuclear microtubule (TAS)<br>polar microtubule (TAS)<br>spindle pole body (IDA)<br>tubulin complex (TAS)
YFR016C	cytoplasm		cellular bud (IDA)<br>cytoplasm (IDA)
YGL096W	cytoplasm;nucleus	Nucleus (Probable).	nuclear chromatin (IDA)
YGL106W	actin;bud	Bud neck. Bud tip.	cellular bud neck (IDA)<br>cellular bud tip (IDA)<br>vesicle (IDA)

YGL123W	cytoplasm	Cytoplasm. Nucleus › nucleolus	cytosolic small ribosomal subunit (IDA)<br>small-subunit processome (IDA)
YGL154C	cytoplasm;nucleus		cytoplasm (TAS)
YGL198W	early Golgi	Golgi apparatus membrane; Multi-pass membrane protein	Golgi apparatus (IDA)
YGL208W	cytoplasm	Cytoplasm. Cell membrane; Peripheral membrane protein; Cytoplasmic side.	AMP-activated protein kinase complex (IDA, IPI)<br>cytoplasm (IDA)<br>plasma membrane (IDA)
YGL249W	cytoplasm;nucleus	Nucleus. Chromosome.	synaptonemal complex (IDA)
YGR041W	cell periphery;bud neck	Cell membrane; Multi-pass membrane protein.	cellular bud neck (IDA)<br>plasma membrane (IMP)
YGR143W	late Golgi	Membrane; Single-pass type II membrane protein.	integral to membrane (IDA)
YGR144W	cytoplasm;nucleus	Mitochondrion (Potential).	cytosol (IDA)<br>mitochondrion (IMP)
YGR184C	cytoplasm;nucleus		cytoplasm (IGI, IMP)<br>colocalizes_with proteasome regulatory particle, base subcomplex (IPI)
YGR241C	actin	Bud. Bud neck. Cell membrane; Peripheral membrane protein; Cytoplasmic side. Cytoplasm	actin cortical patch (TAS)
YGR249W	cytoplasm;nucleus	Nucleus (Potential).	nucleus (IC)
YHR019C	cytoplasm;nucleus	Cytoplasm.	cytoplasm (TAS)
YHR081W	nucleolus;cytoplasm;nucleus	Nucleus	colocalizes_with nuclear exosome (RNase complex) (IDA)<br>nuclear exosome (RNase complex) (IDA)
YHR088W	nucleus	Nucleus › nucleolus	nucleolus (IDA, IPI)<br>preribosome, large subunit precursor (IDA)
YHR124W	cytoplasm;nucleus	Nucleus	nuclear chromosome (IDA)
YHR134W	cytoplasm;nucleus	Nucleus.	nuclear envelope (IDA)
YHR158C	cytoplasm;nucleus		cellular bud neck (IDA)<br>cellular bud tip (IDA)<br>mating projection tip (IDA)<br>cytoplasm (IDA)
YHR161C	actin	Bud. Bud neck. Cell membrane; Peripheral membrane protein; Cytoplasmic side. Cytoplasm	actin cortical patch (TAS)
YHR185C	cytoplasm	Nucleus. Cytoplasm › cytoskeleton › spindle pole body	
YIL068C	bud	Cytoplasm.	exocyst (IDA)<br>mating projection tip (IDA)
YIL118W	bud	Cell membrane; Lipid-anchor; Cytoplasmic side (Potential).	cellular bud (IDA)<br>cytosol (IDA)<br>membrane fraction (IDA)<br>plasma membrane enriched fraction (IDA)
YIR008C	cytoplasm;nucleus		alpha DNA polymerase:primase complex (IDA)<br>nuclear replication fork (IDA)

YIR025W	cytoplasm;nucleus		anaphase-promoting complex (IDA, IPI)<br>nucleus (IDA)
YJL072C	cytoplasm;nucleus	Nucleus	DNA replication preinitiation complex (IPI)<br>GINS complex (IPI)<br>nuclear replication fork (IDA)<br>nucleus (IDA)<br>replication fork protection complex (IDA)
YJL089W	cytoplasm;nucleus	Nucleus (Probable).	nucleus (IDA)
YJL106W	cytoplasm;nucleus		nucleus (IDA)
YJL184W	cytoplasm;nucleus	Nucleus (Potential). Chromosome › telomere (Probable).	EKC/KEOPS protein complex (IDA)<br>colocalizes_with nuclear chromatin (IDA)
YJR066W	microtubule;cytoplasm	Cell membrane; Peripheral membrane protein; Cytoplasmic side. Vacuole membrane; Peripheral membrane protein; Cytoplasmic side.	cytoplasm (IDA)<br>endosome membrane (IDA)<br>extrinsic to internal side of plasma membrane (IDA)<br>Golgi membrane (IDA)<br>membrane fraction (IDA)<br>nucleus (IDA)<br>plasma membrane (IDA)<br>TORC1 complex (IPI)<br>vacuolar membrane (IDA)
YJR104C	cytoplasm;nucleus;bud	Cytoplasm. Mitochondrion intermembrane space.	cytosol (IDA)<br>mitochondrial intermembrane space (IDA)<br>nucleus (IDA)<br>mitochondrion (IDA)
YKL045W	cytoplasm;nucleus		alpha DNA polymerase:primase complex (IDA)<br>nuclear envelope (IDA)<br>nucleus (IDA)
YKL109W	cytoplasm;nucleus	Nucleus.	CCAAT-binding factor complex (TAS)
YKL172W	nucleus	Nucleus › nucleolus	nucleolus (IDA)<br>preribosome, large subunit precursor (IDA)
YKL196C	early Golgi;lipid particle	Cell membrane; Lipid-anchor; Cytoplasmic side (Potential).	fungal-type vacuole (IDA)<br>membrane (IDA)<br>SNARE complex (IDA)<br>soluble fraction (IDA)<br>mitochondrion (IDA)
YKL203C	cytoplasm	Cell membrane; Peripheral membrane protein; Cytoplasmic side. Vacuole membrane; Peripheral membrane protein; Cytoplasmic side.	extrinsic to internal side of plasma membrane (IDA)<br>membrane fraction (IDA)<br>plasma membrane (IDA)<br>TORC1 complex (IPI)<br>TORC2 complex (IPI)<br>vacuolar membrane (IDA)<br>mitochondrion (IDA)
YKR091W	cytoplasm;nucleus	Cytoplasm	cytoplasm (IDA)
YKR095W-A	cytoplasm;nucleus	Nucleus (Probable). Chromosome › telomere (Probable).	EKC/KEOPS protein complex (IDA)<br>colocalizes_with nuclear chromatin (IDA)

YLL050C	actin	Cytoplasm. Cytoplasm › cytoskeleton. Nucleus matrix.	actin cortical patch (IDA)<br>plasma membrane enriched fraction (IDA)
YLR007W	cytoplasm;nucleus	Nucleus	nucleus (IDA)<br>Smc5-Smc6 complex (IPI)
YLR009W	nucleus	Cytoplasm. Nucleus.	nucleolus (IDA)<br>preribosome, large subunit precursor (IDA)
YLR010C	cytoplasm;nucleus	Nucleus (Potential). Chromosome › telomere (Potential).	nuclear telomere cap complex (IPI)
YLR078C	early Golgi	Golgi apparatus membrane; Single-pass type IV membrane protein (Probable). Endoplasmic reticulum membrane; Single-pass type IV membrane protein (Probable).	endoplasmic reticulum membrane (IDA)<br>ER to Golgi transport vesicle membrane (IDA)<br>integral to membrane (IDA)<br>SNARE complex (IDA)
YLR097C	cytoplasm;nucleus		SCF ubiquitin ligase complex (IPI, ISS)
YLR147C	nucleus	Cytoplasm. Nucleus	commitment complex (IPI)<br>U1 snRNP (IDA)<br>U2-type prespliceosome (IDA)<br>U4/U6 x U5 tri-snRNP complex (IDA)<br>U5 snRNP (IDA)
YLR166C	bud		exocyst (IDA)<br>mating projection tip (IDA)
YLR167W	cytoplasm	Cytoplasm (By similarity). Nucleus (By similarity).	cytosolic small ribosomal subunit (IDA)<br>cytoplasm (IDA)
YLR187W	cytoplasm	Cytoplasm. Bud neck. Cell membrane; Peripheral membrane protein; Cytoplasmic side	cellular bud (IDA)<br>cellular bud neck (IDA)<br>cytoplasm (IDA)
YLR233C	cytoplasm;nucleus	Nucleus. Chromosome › telomere (Probable).	nucleolus (IDA)<br>nucleus (IDA)<br>telomerase holoenzyme complex (IDA, IPI)
YLR262C	early Golgi	Cell membrane; Lipid-anchor; Cytoplasmic side (Potential).	Golgi apparatus (TAS)
YLR276C	nucleus	Nucleus › nucleolus	nucleolus (TAS)
YLR288C	cytoplasm;nucleus	Nucleus (Potential).	checkpoint clamp complex (IDA)<br>nucleus (TAS)
YLR304C	cytoplasm	Mitochondrion. Cytoplasm.	cytosol (TAS)<br>mitochondrial matrix (TAS)<br>mitochondrial nucleoid (IDA)<br>mitochondrion (IDA)
YLR313C	cytoplasm	Bud tip. Bud neck. Cytoplasm › cytoskeleton.	cellular bud neck (IDA)<br>cellular bud tip (IDA)<br>incipient cellular bud site (IDA)<br>mating projection (IDA)<br>polarisome (TAS)
YLR318W	cytoplasm;nucleus	Nucleus. Chromosome › telomere.	nucleolus (IDA)<br>nucleus (IDA)<br>telomerase catalytic core complex (IDA, IPI)<br>telomerase holoenzyme complex (IDA, IPI)

YLR320W	cytoplasm;nucleus	Nucleus.	Cul8-RING ubiquitin ligase complex (IDA)<br>nucleus (IDA)
YLR368W	cytoplasm;nucleus	Cytoplasm. Mitochondrion	mitochondrion (IPI)<br>SCF ubiquitin ligase complex (IPI)
YLR394W	cytoplasm;nucleus	Nucleus. Chromosome.	condensed nuclear chromosome (IDA)<br>nuclear chromosome (IDA)
YLR433C	cytoplasm;bud		calcineurin complex (IDA, IPI)<br>cytoplasm (TAS)
YML023C	cytoplasm;nucleus	Nucleus	Smc5-Smc6 complex (IPI)<br>nucleus (IDA)
YML036W	cytoplasm;nucleus	Nucleus (Probable). Chromosome › telomere (Probable).	EKC/KEOPS protein complex (IDA)
YML130C	ER to Golgi	Endoplasmic reticulum membrane; Peripheral membrane protein; Lumenal side	endoplasmic reticulum (IDA)
YMR100W	cytoplasm;nucleus	Cytoplasm (By similarity).	
YMR159C	cytoplasm	Vacuole (Potential). Cytoplasmic vesicle (Potential)	Atg12-Atg5-Atg16 complex (IDA, IPI)<br>membrane fraction (IDA)<br>pre-autophagosomal structure (IDA)
YMR192W	actin;cytoplasm		cellular bud neck (IDA)<br>cellular bud tip (IDA)<br>Golgi-associated vesicle (IDA)<br>incipient cellular bud site (IDA)<br>plasma membrane (IDA)<br>cellular bud (IDA)<br>cytoplasm (IDA)<br>mitochondrion (IDA)
YMR202W	ER;lipid particle	Endoplasmic reticulum membrane; Single-pass membrane protein (Potential).	endoplasmic reticulum (TAS)
YMR312W	cytoplasm	Cytoplasm. Nucleus	Elongator holoenzyme complex (IDA)
YNL002C	nucleus	Nucleus › nucleolus	nucleolus (IDA)<br>preribosome, large subunit precursor (IDA)
YNL014W	cytoplasm;nucleus		cytosolic ribosome (IPI)
YNL093W	cytoplasm;nucleus	Cell membrane; Lipid-anchor; Cytoplasmic side (Potential).	late endosome (IMP)
YNL145W	cytoplasm	Cell membrane; Lipid-anchor; Cytoplasmic side (Potential).	extracellular region (IDA)
YNL199C	cytoplasm;nucleus	Nucleus (Probable).	nuclear envelope (IDA)<br>nucleus (IDA)
YNL272C	cytoplasm	Bud neck. Bud tip. Cytoplasmic vesicle › secretory vesicle.	cellular bud neck (IDA)<br>cellular bud tip (IDA)<br>cytosol (IDA)<br>transport vesicle (IDA)<br>mating projection tip (IDA)
YNL289W	cytoplasm	Cytoplasm. Nucleus.	cyclin-dependent protein kinase holoenzyme complex (IPI)<br>incipient cellular bud site (IDA)<br>nucleus (IDA)

YNL293W	cytoplasm	Cytoplasm. Bud. Bud neck.	cellular bud tip (IDA)<br>incipient cellular bud site (IDA)<br>polarisome (IPI)
YNR049C	lipid particle		cellular bud membrane (IDA)<br>cellular bud neck (IDA)<br>cellular bud tip (IDA)<br>microsome (IDA)<br>plasma membrane (IDA)<br>prospore membrane (IDA)<br>SNARE complex (IDA)
YOL040C	cytoplasm;nucleus	Cytoplasm (By similarity).	cytosolic small ribosomal subunit (NAS)
YOL078W	cytoplasm	Cell membrane; Peripheral membrane protein; Cytoplasmic side. Vacuole membrane; Peripheral membrane protein; Cytoplasmic side	cytoplasm (IPI)<br>plasma membrane (IDA)<br>TORC2 complex (IPI)
YOL083W	cytoplasm	Preautophagosomal structure membrane; Peripheral membrane protein	colocalizes_with CVT complex (IDA)
YOL112W	cytoplasm	Cytoplasm. Bud. Bud neck.	cellular bud tip (IDA)<br>incipient cellular bud site (IDA)<br>polarisome (IPI)
YOL146W	cytoplasm;nucleus	Nucleus	DNA replication preinitiation complex (IPI)<br>GINS complex (IPI)<br>replication fork protection complex (IDA)
YOR005C	cytoplasm;nucleus	Nucleus (Potential).	DNA ligase IV complex (IPI)<br>colocalizes_with nuclear chromatin (IPI)
YOR036W	early Golgi	Membrane; Single-pass type IV membrane protein (Potential).	endosome (IMP)<br>Golgi apparatus (IMP)
YOR047C	cytoplasm;nucleus		nucleus (IDA)<br>plasma membrane (IDA)
YOR076C	cytoplasm;nucleus	Cytoplasm	cytoplasm (IDA)<br>colocalizes_with cytoplasmic exosome (RNase complex) (IDA)<br>colocalizes_with Ski complex (IDA)
YOR080W	cytoplasm;nucleus	Nucleus (Probable).	colocalizes_with nuclear replication fork (IDA)<br>nucleus (IDA)<br>SCF ubiquitin ligase complex (IDA)
YOR119C	nucleus	Cytoplasm	cytoplasm (IDA)<br>nucleus (IDA)
YOR159C	nucleus	Cytoplasm. Nucleus	U1 snRNP (IDA)<br>U2-type prespliceosome (IDA)<br>U4/U6 x U5 tri-snRNP complex (IDA)<br>U5 snRNP (IDA)
YOR250C	ER to Golgi;nucleus	Nucleus (Probable).	mRNA cleavage factor complex (IPI)

YOR326W	early Golgi;bud	Bud neck. Bud tip.	actin filament bundle (IMP)<br>cellular bud neck (IDA)<br>cellular bud tip (IDA)<br>filamentous actin (IDA)<br>colocalizes_with fungal-type vacuole membrane (IDA)<br>incipient cellular bud site (IDA)<br>mating projection tip (IDA)<br>myosin V complex (ISS)<br>vesicle (IDA)<br>mating projection tip (IDA)
YOR368W	cytoplasm;nucleus	Nucleus (Potential).	checkpoint clamp complex (IDA)<br>nucleus (IPI)
YPL001W	nucleus	Cytoplasm. Nucleus	cytoplasm (IDA)<br>histone acetyltransferase complex (IDA, IPI)<br>nucleus (IDA)
YPL101W	cytoplasm	Cytoplasm. Nucleus	Elongator holoenzyme complex (IDA)<br>cytoplasm (IDA)
YPL121C	cytoplasm;nucleus	Nucleus	condensed nuclear chromosome (IDA)
YPL164C	cytoplasm;nucleus	Nucleus (Probable).	MutLbeta complex (IPI)<br>nucleus (IPI)
YPL167C	cytoplasm;nucleus	Mitochondrion. Nucleus (Potential).	mitochondrion (IDA)<br>colocalizes_with nuclear chromatin (IPI)<br>zeta DNA polymerase complex (IDA)
YPL200W	nucleus	Endoplasmic reticulum membrane; Single-pass membrane protein. Nucleus membrane; Single-pass membrane protein (Probable).	endoplasmic reticulum membrane (IDA)<br>nuclear chromosome, telomeric region (IDA)
YPL204W	cytoplasm	Cytoplasm. Nucleus › nucleolus. Nucleus › nucleoplasm	cellular bud neck (IDA)<br>cellular bud tip (IDA)<br>chromosome, centromeric region (IDA)<br>monopolin complex (IDA, IMP, IPI)<br>nucleus (IDA)<br>plasma membrane (IDA)<br>spindle pole body (IDA)
YPL217C	nucleolus	Cytoplasm. Nucleus › nucleolus	90S preribosome (IDA)<br>cytoplasm (IDA)<br>nucleolus (IDA)<br>nucleus (IDA)<br>mitochondrion (IDA)
YPL218W	ER to Golgi;bud	Cytoplasmic vesicle › COPII-coated vesicle membrane; Peripheral membrane protein; Cytoplasmic side. Endoplasmic reticulum membrane; Peripheral membrane protein; Cytoplasmic side. Golgi apparatus membrane; Peripheral membrane protein; Cytoplasmic side (Potential).	COPII vesicle coat (IDA)

YPL249C	actin;cytoplasm	Cytoplasm. Bud. Bud neck	cellular bud neck (IDA)<br>cellular bud tip (IDA)<br>cytosol (IDA)<br>Golgi-associated vesicle (IDA)<br>incipient cellular bud site (IDA)<br>membrane fraction (IDA)<br>plasma membrane (IDA)<br>soluble fraction (IDA)
YPL266W	cytoplasm	Cytoplasm. Nucleus › nucleolus	90S preribosome (IDA)<br>nucleolus (TAS)
YPL267W	cytoplasm		cytoplasm (IDA)<br>nucleus (IDA)
YPR030W	cytoplasm	Cytoplasm. Nucleus	nucleus (IDA)
YPR055W	actin;bud	Cytoplasm. Cell membrane; Peripheral membrane protein.	cellular bud tip (IDA)<br>exocyst (IDA)<br>incipient cellular bud site (TAS)<br>site of polarized growth (IDA)<br>mating projection tip (IDA)
YPR082C	cytoplasm;nucleus	Nucleus.	U4/U6 x U5 tri-snRNP complex (IDA)<br>U5 snRNP (IDA)
YPR093C	nucleus	Cytoplasm. Nucleus.	cytoplasm (IDA)<br>nucleus (IDA)
YPR094W	cytoplasm;nucleus	Nucleus	colocalizes_with U2 snRNP (IDA)<br>U2 snRNP (IDA)
YPR095C	early Golgi	Cytoplasm	Golgi apparatus part (IDA)<br>mitochondrion (IDA)
YPR119W	cytoplasm;nucleus		cellular bud neck (IDA)<br>cytoplasm (IDA, IMP, ISS)<br>nucleus (IDA)<br>spindle (IDA)<br>spindle pole body (IDA)
YPR120C	cytoplasm;nucleus		nucleus (IDA)
YPR162C	cytoplasm;nucleus	Nucleus.	DNA replication preinitiation complex (IDA)<br>nuclear origin of replication recognition complex (IDA, IMP)<br>pre-replicative complex (IDA)
YPR164W	cytoplasm;nucleus	Nucleus (Probable).	Cul8-RING ubiquitin ligase complex (IDA)
YPR165W	cytoplasm;bud	Cell membrane; Lipid-anchor. Endosome membrane; Lipid-anchor. Peroxisome membrane; Lipid-anchor.	1,3-beta-D-glucan synthase complex (IDA)<br>cellular bud neck (IDA)<br>cellular bud tip (IDA)<br>Golgi apparatus (IDA)<br>incipient cellular bud site (IDA)<br>mating projection tip (IDA)<br>membrane fraction (IDA)<br>peroxisome (IDA)<br>mitochondrial outer membrane (IDA)<br>mitochondrion (IDA)<br>plasma membrane enriched fraction (IDA)
YPR167C	cytoplasm;nucleus		cytoplasm (TAS)<br>intracellular (TAS)
YAL003W	cytoplasm		ribosome (TAS)
YAL028W	cytoplasm;nucleus	Endoplasmic reticulum membrane; Single-pass membrane protein.	endoplasmic reticulum (IDA)

YAL030W	lipid particle	Endomembrane system; Single-pass type IV membrane protein.	cellular bud neck (IDA)<br>endosome (IDA)<br>plasma membrane (IDA)<br>SNARE complex (IDA)<br>trans-Golgi network (IDA)<br>transport vesicle (IDA)
YAL040C	cytoplasm		nucleus (IDA, IMP)
YAL062W	actin;cytoplasm		nucleus (IDA)<br>mitochondrion (IDA)
YAR019C	cytoplasm		cellular bud neck (TAS)<br>spindle pole body (IDA)
YBL050W	early Golgi	Membrane; Peripheral membrane protein.	Golgi apparatus;endoplasmic reticulum (InterPro)
YBL066C	cytoplasm	Nucleus (Probable)	
YBL105C	actin		cytoplasm (IDA)<br>cytoskeleton (IDA)<br>nucleus (IDA)
YBR005W	cytoplasm	Endoplasmic reticulum membrane; Single-pass type I membrane protein	integral to endoplasmic reticulum membrane (IDA, ISS)
YBR038W	cytoplasm;nucleus	Membrane; Multi-pass membrane protein.	cellular bud neck (IDA)
YBR045C	cytoplasm;nucleus		prospore membrane (IDA)
YDL171C	cytoplasm;nucleus		mitochondrion (IDA)
YDL179W	cytoplasm;nucleus		cellular bud neck (IDA)<br>cyclin-dependent protein kinase holoenzyme complex (TAS)<br>incipient cellular bud site (IDA)
YDL199C	cytoplasm	Membrane; Multi-pass membrane protein.	membrane (ISS)
YDL203C	cytoplasm;bud		mitochondrion (IDA)
YDR164C	lipid particle		cellular bud neck (IDA)<br>cellular bud tip (IDA)<br>plasma membrane (IDA)
YDR313C	cytoplasm	Endosome membrane; Peripheral membrane protein. Vacuole membrane; Peripheral membrane protein	fungal-type vacuole membrane (IDA)<br>late endosome (IDA)
YEL030W	cytoplasm	Mitochondrion matrix › mitochondrion nucleoid	mitochondrial nucleoid (IDA)<br>mitochondrion (IDA)<br>mitochondrion (IDA)
YER048C	cytoplasm		nucleus (TAS)
YER149C	cytoplasm		actin cap (TAS)<br>polarisome (TAS)<br>mating projection tip (IDA)
YER179W	cytoplasm	Nucleus	condensed nuclear chromosome (IDA)<br>nucleus (IDA)
YFL011W	cytoplasm	Membrane; Multi-pass membrane protein.	plasma membrane (ISS)<br>mitochondrion (IDA)
YFL030W	cytoplasm;nucleus		mitochondrion (IDA)

YFL039C	actin	Cytoplasm › cytoskeleton.	actin cortical patch (IDA)<br>actin filament (TAS)<br>actin filament bundle (IDA)<br>cellular bud neck contractile ring (IDA)<br>histone acetyltransferase complex (IDA)<br>Ino80 complex (IPI)<br>NuA4 histone acetyltransferase complex (IPI)<br>Swr1 complex (IDA)
YFL050C	cytoplasm;nucleus	Membrane; Multi-pass membrane protein.	plasma membrane (IMP)
YFL052W	cytoplasm	Nucleus (Probable).	
YFR003C	cytoplasm	Nucleus	nucleus (IDA)
YFR042W	vacuole	Endoplasmic reticulum membrane; Multi-pass membrane protein	integral to endoplasmic reticulum membrane (IDA)
YGL030W	nucleus	Cytoplasm (By similarity).	cytoplasm (IDA)<br>cytosolic large ribosomal subunit (TAS)
YGL100W	ER to Golgi	Nucleus › nuclear pore complex. Cell membrane; Peripheral membrane protein; Cytoplasmic side. Cell membrane; Peripheral membrane protein; Nucleoplasmic side.	nuclear pore (IDA)<br>Nup107-160 complex (IDA)
YGR023W	cytoplasm	Membrane; Single-pass type I membrane protein (Potential).	integral to plasma membrane (ISS)
YGR032W	cytoplasm	Membrane; Multi-pass membrane protein	1,3-beta-D-glucan synthase complex (IDA)<br>actin cap (TAS)<br>membrane fraction (IDA)<br>prospore membrane (IDA)
YGR099W	cytoplasm	Nucleus (Potential). Chromosome › telomere (Probable).	nuclear telomere cap complex (IDA)<br>ASTRA complex (IDA)
YGR221C	cytoplasm	Cell membrane; Single-pass membrane protein (Potential). Bud membrane; Single-pass membrane protein (Potential).	cellular bud neck (IDA)<br>cellular bud tip (IDA)
YGR238C	cytoplasm		cellular bud neck (IDA)<br>cellular bud tip (IDA)<br>mating projection tip (IDA)
YHL022C	cytoplasm	Nucleus	nuclear chromosome (IPI)
YHR001W	endosome		cortical endoplasmic reticulum (IDA)<br>cytoplasm (IDA)<br>soluble fraction (IDA)
YHR076W	cytoplasm;nucleus	Mitochondrion	mitochondrion (IDA)<br>nuclear envelope (IDA)
YHR080C	cytoplasm	Membrane; Single-pass membrane protein (Potential).	mitochondrion (IDA)<br>colocalizes_with ribosome (IDA)
YHR094C	ER	Membrane; Multi-pass membrane protein.	membrane fraction (IDA)<br>plasma membrane (IDA)
YHR157W	cytoplasm		condensed nuclear chromosome (IDA)

YHR168W	cytoplasm	Mitochondrion inner membrane; Peripheral membrane protein; Matrix side	extrinsic to membrane (IDA)<br>mitochondrial inner membrane (IDA)
YHR208W	cytoplasm;nucleus	Mitochondrion matrix.	mitochondrial matrix (IDA)<br>mitochondrion (IDA)
YIL018W	nucleus	Cytoplasm	cytosolic large ribosomal subunit (IDA)
YIL072W	cytoplasm	Nucleus. Chromosome.	condensed nuclear chromosome (IDA)<br>lateral element (IPI)
YIL129C	cytoplasm		cellular bud (IDA)<br>incipient cellular bud site (IDA)<br>mating projection tip (IDA)<br>mitochondrion (IDA)
YIL139C	cytoplasm;nucleus	Mitochondrion	mitochondrion (IDA)<br>colocalizes_with nuclear chromatin (IDA)<br>zeta DNA polymerase complex (IDA)
YIL140W	cytoplasm	Cell membrane; Single-pass type I membrane protein.	cellular bud neck (IDA)<br>integral to plasma membrane (TAS)<br>septin ring (TAS)
YIR033W	cytoplasm	Membrane; Single-pass membrane protein (Potential).	endoplasmic reticulum membrane (IDA)<br>nucleus (IDA)
YIR039C	cytoplasm	Cell membrane; Lipid-anchor › GPI-anchor (Probable)	fungal-type cell wall (IDA)
YJL005W	actin;cytoplasm;bud		plasma membrane (IDA)<br>mitochondrion (IDA)
YJL012C	bud	Vacuole membrane; Multi-pass membrane protein	endoplasmic reticulum (IDA)<br>intrinsic to vacuolar membrane (IDA)<br>vacuolar transporter chaperone complex (IPI)
YJL023C	cytoplasm	Mitochondrion matrix Potential.	mitochondrion (IDA)
YJL093C	cytoplasm	Membrane; Multi-pass membrane protein.	plasma membrane (IDA, IMP)
YJL100W	actin	Cell membrane; Peripheral membrane protein. Vacuole membrane; Peripheral membrane protein.	fungal-type vacuole membrane (IDA)<br>plasma membrane (IDA)<br>cytoplasm (IDA)
YJL108C	cytoplasm	Membrane; Multi-pass membrane protein.	integral to membrane (ISS)
YJL167W	nucleus	Cytoplasm.	cytoplasm (IDA)<br>endoplasmic reticulum (IDA)
YJL172W	cytoplasm	Vacuole membrane; Single-pass membrane protein.	fungal-type vacuole lumen (IDA)
YJL187C	cytoplasm	Bud neck. Nucleus.	cellular bud neck (IDA)<br>nucleus (IDA)
YJR033C	vacuolar membrane	Endomembrane system.	cytoplasm (IDA)<br>extrinsic to membrane (IDA)<br>RAVE complex (IPI)
YJR091C	cytoplasm		external side of mitochondrial outer membrane (IDA)
YJR094C	cytoplasm	Nucleus.	nucleus (IDA)

YJR144W	cytoplasm;nucleus	Mitochondrion matrix › mitochondrion nucleoid	mitochondrial chromosome (IDA)<br>mitochondrial nucleoid (IDA)<br>mitochondrion (IDA)
YKL034W	cytoplasm;nucleus	Golgi apparatus membrane; Multi-pass membrane protein	Golgi apparatus (IDA)
YKL057C	ER to Golgi	Nucleus › nuclear pore complex. Nucleus membrane; Peripheral membrane protein; Cytoplasmic side. Nucleus membrane; Peripheral membrane protein; Nucleoplasmic side.	nuclear pore (IDA)<br>Nup107-160 complex (IDA)
YKL082C	cytoplasm	Nucleus › nucleolus	colocalizes_with cytosolic large ribosomal subunit (IDA)<br>nucleolus (IDA)
YKL099C	cytoplasm	Nucleus › nucleolus	nucleolus (IDA)<br>small-subunit processome (IDA)
YKL217W	cytoplasm	Membrane; Multi-pass membrane protein.	plasma membrane (IMP, ISS)<br>mitochondrion (IDA)
YLL017W	cytoplasm;nucleus		membrane (IDA)
YLL021W	cytoplasm	Cell tip.	cellular bud neck (IDA)<br>cellular bud tip (IDA)<br>incipient cellular bud site (IDA)<br>mating projection tip (IDA)<br>polarisome (TAS)
YLL035W	cytoplasm	Nucleus › nucleolus	nucleolar chromatin (IPI)
YLR256W	cytoplasm	Nucleus (By similarity).	nucleus (IDA)
YLR306W	cytoplasm		cellular bud neck (IDA)<br>cellular bud tip (IDA)<br>incipient cellular bud site (IDA)<br>mating projection (IDA)<br>polarisome (TAS)
YLR329W	cytoplasm	Nucleus.	condensed nuclear chromosome (IDA)<br>nucleus (IPI)
YLR355C	cytoplasm	Mitochondrion.	mitochondrial nucleoid (IDA)<br>mitochondrion (TAS)
YLR361C	nucleus	Cytoplasm	
YLR431C	punctate composite	Cytoplasm. Preautophagosomal structure membrane; Peripheral membrane protein. Membrane; Peripheral membrane protein.	peripheral to membrane of membrane fraction (IDA)<br>pre-autophagosomal structure (IDA)
YML123C	cytoplasm	Membrane; Multi-pass membrane protein.	integral to plasma membrane (IDA, ISS)
YMR017W	lipid particle	Cell membrane. Prospore membrane.	prospore membrane (IMP, ISS)
YNL101W	cytoplasm	Vacuole membrane; Multi-pass membrane protein	fungal-type vacuole (IDA)<br>fungal-type vacuole membrane (IMP)
YNL257C	cytoplasm	Membrane; Multi-pass membrane protein.	
YNL298W	cytoplasm		actin cap (TAS)<br>cellular bud (IDA)<br>fungal-type vacuole (IDA)<br>nucleus (IMP)

YOL025W	cytoplasm;nucleus		mitochondrion (IDA)
YOL031C	early Golgi	Endoplasmic reticulum lumen.	endoplasmic reticulum (IPI)
YOL109W	cytoplasm	Cell membrane; Peripheral membrane protein	extrinsic to plasma membrane (IDA)<br>mitochondrial outer membrane (IDA)<br>mitochondrion (IDA)<br>plasma membrane enriched fraction (IDA)
YOL113W	cytoplasm		nucleus (IDA, IMP)<br>plasma membrane (IDA)
YOL122C	cytoplasm	Cell membrane; Multi-pass membrane protein.	plasma membrane (IDA)
YOL127W	nucleus	Cytoplasm (By similarity).	cytosolic large ribosomal subunit (TAS)
YOL130W	cytoplasm;nucleus	Membrane; Multi-pass membrane protein (Potential).	plasma membrane (IMP)
YOR032C	cytoplasm	Nucleus (Potential).	
YOR059C	nucleolus	Lipid droplet. Membrane; Single-pass membrane protein (Potential).	lipid particle (IDA)
YOR075W	early Golgi;lipid particle	Endoplasmic reticulum membrane; Single-pass type IV membrane protein.	endoplasmic reticulum (IDA)<br>integral to membrane (ISM)<br>SNARE complex (IDA)
YOR090C	cytoplasm;nucleus	Mitochondrion membrane; Single-pass membrane protein (Potential).	mitochondrion (IDA)
YOR103C	ER	Membrane; Multi-pass membrane protein.	oligosaccharyltransferase complex (IPI)
YOR106W	early Golgi	Vacuole membrane; Single-pass type IV membrane protein.	fungal-type vacuole (IDA)<br>vacuolar membrane (IDA)
YOR231W	cytoplasm		cellular bud tip (IDA)
YOR324C	cytoplasm	Endoplasmic reticulum membrane; Single-pass membrane protein	endoplasmic reticulum (IDA)
YOR351C	cytoplasm		nucleus (IDA)
YPL032C	actin	Cytoplasm. Bud. Bud neck. Cytoplasm › cell cortex.	cellular bud (IDA)<br>cellular bud neck (IDA)<br>cytoplasm (IDA)
YPR026W	cytoplasm	Vacuole.	cell wall-bounded periplasmic space (IDA)<br>fungal-type cell wall (IDA)<br>fungal-type vacuole (IMP)
YPR048W	cytoplasm		mitochondrion (IDA)
YPR054W	cytoplasm;nucleus		mitochondrion (IDA)
YPR089W	cytoplasm	Golgi apparatus	
YPR201W	cytoplasm	Membrane; Multi-pass membrane protein (Potential).	plasma membrane (IDA)
YAL034C	nucleus		
YAR018C	spindle pole		
YBL049W	cytoplasm;nucleus		
YBL067C	cytoplasm		
YBR035C	cytoplasm		
YBR050C	cytoplasm;nucleus		

YBR225W	cytoplasm		
YBR270C	cytoplasm		
YBR280C	cytoplasm;nucleus		SCF ubiquitin ligase complex (IMP, IPI, ISS)
YBR285W	cytoplasm		
YCL051W	cytoplasm		fungal-type cell wall (TAS)
YCR008W	cytoplasm		
YCR015C	vacuolar membrane		
YCR073C	cytoplasm		
YCR076C	cytoplasm;nucleus		
YDL073W	cytoplasm		
YDL176W	cytoplasm;nucleus		
YDR306C	cytoplasm;nucleus		ubiquitin ligase complex (IDA)
YDR338C	cytoplasm		
YDR415C	cytoplasm;nucleus		
YDR436W	cytoplasm		
YDR475C	cytoplasm;nucleus		
YDR502C	cytoplasm		
YEL062W	cytoplasm		
YER128W	endosome		
YER158C	cytoplasm;bud		
YFL059W	cytoplasm		
YFL060C	cytoplasm		
YFR022W	cytoplasm		
YGL015C	cytoplasm		
YGL144C	cytoplasm		
YGL158W	cytoplasm;nucleus		
YGL215W	cytoplasm;nucleus		cyclin-dependent protein kinase holoenzyme complex (IPI)
YGL242C	actin;bud		
YGR009C	lipid particle;bud		extrinsic to plasma membrane (IDA)<br>SNARE complex (IDA)
YGR068C	cytoplasm;nucleus		
YHR025W	cytoplasm;nucleus		
YHR035W	cytoplasm;nucleus		
YHR128W	cytoplasm;nucleus		intracellular (IC)
YJL031C	cytoplasm;nucleus		Rab-protein geranylgeranyltransferase complex (IDA, IPI)
YJL149W	cytoplasm;nucleus		SCF ubiquitin ligase complex (IPI)
YJR011C	cytoplasm		
YJR012C	peroxisome	Membrane; Single-pass membrane protein (Potential).	
YJR083C	actin;cytoplasm		
YJR141W	cytoplasm;nucleus		
YKL090W	cytoplasm		
YKL104C	cytoplasm		

YKL161C	cytoplasm		
YKR056W	cytoplasm		
YKR058W	cytoplasm;nucleus		
YLR021W	cytoplasm;nucleus		intracellular (IDA)
YLR128W	cytoplasm;nucleus		
YLR209C	cytoplasm		intracellular (IC)
YLR224W	cytoplasm;nucleus		ubiquitin ligase complex (IPI, ISS)
YLR267W	cytoplasm;nucleus		
YLR352W	cytoplasm;nucleus		
YLR359W	cytoplasm		
YLR425W	cytoplasm		
YMR028W	cytoplasm;nucleus		peripheral to membrane of membrane fraction (IDA) soluble fraction (IDA)
YMR096W	cytoplasm		
YMR154C	endosome		
YMR191W	cytoplasm;nucleus		
YMR210W	cytoplasm		
YNL050C	cytoplasm;nucleus		
YNL127W	cytoplasm		
YNL244C	cytoplasm		multi-eIF complex (IDA)
YNL333W	cytoplasm		
YNL334C	cytoplasm		
YOL036W	cytoplasm		
YOL063C	cytoplasm;nucleus		
YOR054C	cytoplasm		phosphopantothenoylcysteine decarboxylase complex (IDA)
YOR134W	cytoplasm		intracellular (TAS)
YOR155C	cytoplasm		
YOR178C	nucleus		protein phosphatase type 1 complex (IDA)
YOR349W	cytoplasm		
YPL003W	punctate composite;cytoplasm		
YPL023C	cytoplasm;nucleus		
YPL088W	cytoplasm;nucleus		
YPL150W	cytoplasm		
YPL165C	cytoplasm		
YPR106W	cytoplasm;nucleus		
YPR176C	cytoplasm;nucleus		Rab-protein geranylgeranyltransferase complex (IDA, IPI)
Q0120		Mitochondrion.	mitochondrion (IDA)
YAR027W		Nucleus membrane; Multi-pass membrane protein. Cell membrane; Multi-pass membrane protein	nuclear envelope (IDA)
YAR028W		Membrane; Multi-pass membrane protein	

		(Potential)	
YAR042W		Cytoplasm. Golgi apparatus membrane. Nucleus outer membrane	early endosome (IDA)<br>endoplasmic reticulum (IDA)<br>Golgi trans cisterna (IDA)<br>nuclear envelope (IDA)
YBR128C		Membrane; Peripheral membrane protein	Manually curated fungal-type vacuole membrane (IDA)<br>peripheral to membrane of membrane fraction (IDA)
YBR201W		Endoplasmic reticulum membrane; Multi-pass membrane protein	endoplasmic reticulum membrane (IDA)<br>Hrd1p ubiquitin ligase ERAD-L complex (IDA)
YCL008C		Cytoplasm. Endosome. Late endosome membrane; Peripheral membrane protein (Probable)	endosome (IDA, IPI)<br>ESCRT I complex (IDA, IPI)<br>internal side of plasma membrane (IDA)
YCL024W		Bud neck	cellular bud neck (IDA)<br>cellular bud neck septin collar (IDA)<br>incipient cellular bud site (IDA)<br>septin ring (IDA)
YCL063W		Vacuole membrane; Peripheral membrane protein; Cytoplasmic side	vacuolar membrane (IDA)
YDL014W		Nucleus › nucleolus	90S preribosome (IDA)<br>box C/D snoRNP complex (IDA)<br>nucleolus (IDA)<br>small-subunit processome (IDA)<br>colocalizes_with ribosome (IDA)
YDL035C		Cell membrane; Multi-pass membrane protein	plasma membrane (IDA)
YDL077C		Vacuole membrane; Peripheral membrane protein (Potential).	fungal-type vacuole (IDA)<br>fungal-type vacuole membrane (IDA)<br>HOPS complex (IPI)
YDL089W		Membrane; Multi-pass membrane protein.	nuclear periphery (IDA)
YDL148C		Nucleus › nucleolus	90S preribosome (IDA)<br>Noc4p-Nop14p complex (IPI)<br>nucleolus (IDA)<br>small-subunit processome (IDA)<br>mitochondrion (IDA)
YDL149W		Preautophagosomal structure membrane; Multi-pass membrane protein. Cytoplasmic vesicle membrane; Multi-pass membrane protein.	integral to membrane (ISM)<br>membrane fraction (IDA)<br>mitochondrion (IDA)<br>pre-autophagosomal structure (IDA)
YDL194W		Cell membrane; Multi-pass membrane protein	plasma membrane (IDA)
YDL239C		Prospore membrane. Cytoplasm › cytoskeleton › spindle pole body	prospore membrane (IDA)<br>colocalizes_with spindle pole body (IDA, IPI)
YDR002W		Cytoplasm. Nucleus.	cytoplasm (IDA)<br>nucleus (IDA)
YDR057W		Endoplasmic reticulum membrane; Peripheral membrane protein; Lumenal side	endoplasmic reticulum lumen (IDA)<br>colocalizes_with Hrd1p ubiquitin ligase ERAD-L complex (IPI)

			luminal surveillance complex (IDA)
YDR069C		Cytoplasm. Late endosome membrane; Peripheral membrane protein.	endosome (IDA)<br>membrane fraction (IDA)<br>proteasome complex (IPI)<br>mitochondrion (IDA)
YDR106W		Cytoplasm › cytoskeleton (Probable)	cytoplasm (IDA)<br>dynactin complex (IPI)
YDR192C		Nucleus › nuclear pore complex. Nucleus membrane; Peripheral membrane protein; Cytoplasmic side	nuclear pore (IDA)
YDR202C		Cytoplasm (Probable). Early endosome membrane; Peripheral membrane protein; Cytoplasmic side (Potential).	RAVE complex (IPI)
YDR206W		Nucleus (Probable). Chromosome › telomere (Probable).	cytoplasm (IDA)<br>cytoplasmic mRNA processing body (IDA)
YDR218C		Membrane; Peripheral membrane protein (By similarity). Bud neck (By similarity).	cellular bud neck septin ring (TAS)
YDR259C		Nucleus.	nucleus (IDA)
YDR320C		Cytoplasm. Endoplasmic reticulum membrane; Peripheral membrane protein.	endoplasmic reticulum membrane (IDA)
YDR488C		Cytoplasm › cytoskeleton	cytoplasmic dynein complex (TAS)<br>cytoplasmic microtubule (IDA, IPI)
YDR490C			cell cortex (IDA)<br>cytosol (IDA)
YDR507C		Cytoplasm. Bud neck.	cellular bud neck (IDA)
YDR532C		Cytoplasm › cytoskeleton › spindle pole body. Nucleus membrane; Peripheral membrane protein; Nucleoplasmic side. Chromosome › centromere › kinetochore	chromosome, centromeric region (IDA)<br>condensed nuclear chromosome kinetochore (IDA)<br>colocalizes_with spindle pole body (IDA)
YER082C		Nucleus › nucleolus	Manually curated 90S preribosome (IDA)<br>nucleolus (IDA)<br>small-subunit processome (IDA)
YER106W		Nucleus.	condensed nuclear chromosome kinetochore (IDA)<br>monopolin complex (IDA)
YER144C			cellular bud neck (IDA)<br>incipient cellular bud site (IDA)
YFL034W		Membrane; Multi-pass membrane protein.	integral to membrane (ISS)

YFR021W		Preautophagosomal structure membrane; Peripheral membrane protein. Vacuole membrane; Peripheral membrane protein.	cytosol (IDA)<br>endosome (IDA)<br>fungal-type vacuole membrane (IDA)<br>PAS complex (IDA, IPI)<br>pre-autophagosomal structure (IDA)
YGL045W			internal side of plasma membrane (IDA)
YGL103W		Cytoplasm (By similarity).	cytosolic large ribosomal subunit (TAS)<br>nucleus (IMP)
YGL170C		Cytoplasm › cytoskeleton › spindle pole body.	spindle pole body (IDA, IMP)
YGL171W		Nucleus › nucleolus	90S preribosome (IDA)<br>nucleolus (TAS)
YGR029W		Mitochondrion intermembrane space	mitochondrial intermembrane space (IDA)<br>mitochondrion (IDA)
YGR170W		Golgi apparatus › Golgi stack. Vacuole	endosome (IDA)
YGR172C		Endoplasmic reticulum membrane; Multi-pass membrane protein. Golgi apparatus membrane; Multi-pass membrane protein	endoplasmic reticulum membrane (IDA)<br>ER to Golgi transport vesicle (IDA, IPI)<br>integral to Golgi membrane (IDA)
YHL019C		Membrane › coated pit.	AP-1 adaptor complex (IPI)
YHL031C		Golgi apparatus membrane; Single-pass type IV membrane protein	Golgi medial cisterna (IDA)<br>integral to membrane (ISS, NAS)<br>SNARE complex (IDA)
YHR020W			colocalizes_with ribosome (IDA)
YHR129C		Cytoplasm › cytoskeleton (Probable). Membrane	colocalizes_with astral microtubule (IDA)<br>colocalizes_with cell cortex (IDA)<br>dynactin complex (IDA)<br>colocalizes_with spindle pole body (IDA)
YHR150W		Peroxisome membrane; Multi-pass membrane protein	peroxisomal membrane (IDA)
YHR184W		Prospore membrane.	prospore membrane (IDA)
YIL146C		Mitochondrion outer membrane; Single-pass membrane protein. Vacuole.	integral to mitochondrial outer membrane (IDA)
YJL034W		Endoplasmic reticulum lumen.	endoplasmic reticulum (IDA)<br>luminal surveillance complex (IDA)
YJL073W		Endoplasmic reticulum membrane. Nucleus membrane	endoplasmic reticulum (IDA)<br>nuclear membrane-endoplasmic reticulum network (IDA)<br>peripheral to membrane of membrane fraction (IDA)
YJR002W		Nucleus › nucleolus	90S preribosome (IDA)<br>Mpp10 complex (IDA)<br>small-subunit processome (IDA, IMP)<br>nucleolus (IDA)<br>nucleus (IDA)

YJR040W		Membrane; Multi-pass membrane protein.	endoplasmic reticulum (IDA)<br>endosome (IDA)<br>fungal-type vacuole (IDA)<br>Golgi apparatus (IDA)<br>Golgi medial cisterna (IDA)<br>plasma membrane (IDA)
YJR102C		Cytoplasm. Endosome membrane; Peripheral membrane protein	ESCRT II complex (IDA)
YJR123W		Cytoplasm (By similarity)	90S preribosome (IDA)<br>cytosolic small ribosomal subunit (IDA)
YJR125C		Cytoplasm. Golgi apparatus › trans-Golgi network membrane; Peripheral membrane protein. Cytoplasmic vesicle › clathrin-coated vesicle membrane; Peripheral membrane protein.	actin cortical patch (TAS)<br>clathrin vesicle coat (IDA)
YKL079W		Cytoplasm › cytoskeleton (Probable)	cellular bud neck (IDA)<br>cellular bud tip (IDA)<br>incipient cellular bud site (IDA)<br>mating projection tip (IDA)
YKR101W		Nucleus. Chromosome › centromere	chromatin silencing complex (IDA)
YLL049W			colocalizes_with astral microtubule (IDA)<br>colocalizes_with cell cortex (IDA)<br>dynactin complex (IDA)<br>colocalizes_with spindle pole body (IDA)
YLR029C		Cytoplasm (By similarity).	cytosolic large ribosomal subunit (IDA)
YLR119W		Cytoplasm. Endosome (Probable). Late endosome membrane; Peripheral membrane protein (Probable).	endosome (IPI)<br>ESCRT I complex (IDA, IPI)
YLR170C		Golgi apparatus. Cytoplasmic vesicle membrane; Peripheral membrane protein; Cytoplasmic side. Membrane › clathrin-coated pit.	AP-1 adaptor complex (IPI, ISS)
YLR190W		Bud tip. Bud neck. Mitochondrion outer membrane; Peripheral membrane protein	cellular bud (IDA)<br>cellular bud neck (IDA)<br>incipient cellular bud site (IDA)<br>mitochondrial outer membrane (IDA)<br>mitochondrial outer membrane (IDA)<br>mitochondrion (IDA)
YLR207W		Endoplasmic reticulum membrane; Single-pass membrane protein (Potential).	endoplasmic reticulum membrane (IPI)<br>Hrd1p ubiquitin ligase ERAD-L complex (IDA)<br>Hrd1p ubiquitin ligase ERAD-M complex (IDA)<br>luminal surveillance complex

		(IDA)
YLR293C	Nucleus.	cytoplasm (TAS)<br>nucleus (IDA)
YLR305C		plasma membrane (IDA)<br>mitochondrion (IDA)
YLR314C	Membrane; Peripheral membrane protein. Bud neck	ascospore wall (TAS)<br>cellular bud neck septin ring (TAS)<br>mating projection base (IDA)<br>prospore membrane (TAS)<br>septin complex (IDA, IPI)
YLR353W	Cell membrane; Multi-pass membrane protein.	cellular bud tip (IDA)<br>incipient cellular bud site (IDA)<br>plasma membrane (IMP)
YLR377C		cytosol (IDA)
YLR380W	Cytoplasm. Endosome	cytoplasm (IDA)<br>cytosol (IDA)<br>lipid particle (IDA)<br>microsome (IDA)<br>mitochondrion (IDA)
YML029W	Membrane; Multi-pass membrane protein.	endoplasmic reticulum membrane (IPI)<br>Hrd1p ubiquitin ligase ERAD-L complex (IDA, IMP)
YMR029C	Cytoplasm. Endoplasmic reticulum	
YMR124W		cytoplasm (IDA)
YMR163C	Peroxisome membrane; Single-pass membrane protein	integral to peroxisomal membrane (IDA)<br>colocalizes_with peroxisome (IDA)<br>cytoplasm (IDA)
YMR180C	Cytoplasm. Nucleus.	cytoplasm (IDA)<br>nucleus (IDA)
YMR197C	Prevacuolar compartment membrane; Single-pass type IV membrane protein. Golgi apparatus membrane; Single-pass type IV membrane protein.	integral to Golgi membrane (IDA)<br>SNARE complex (IPI)
YMR214W	Endoplasmic reticulum lumen	endoplasmic reticulum lumen (IDA)
YMR228W	Mitochondrion.	mitochondrial DNA-directed RNA polymerase complex (IDA)<br>mitochondrial intermembrane space (IDA)<br>mitochondrial matrix (IDA)<br>mitochondrion (IDA)
YMR294W	Cytoplasm › cytoskeleton (Probable).	colocalizes_with astral microtubule (IDA)<br>colocalizes_with cell cortex (IDA)<br>dynactin complex (IDA)<br>colocalizes_with spindle pole body (IDA)

YNL054W		Vacuole membrane; Single-pass type II membrane protein	fungal-type vacuole membrane (IDA)<br>integral to membrane (IDA)<br>PAS complex (IDA, IPI)<br>cytoplasm (IDA)
YNL075W		Nucleus › nucleolus	90S preribosome (IDA)<br>Mpp10 complex (IDA)<br>small-subunit processome (IDA)
YNL079C		Cytoplasm › cytoskeleton (Probable).	actin filament bundle (IDA)<br>cellular bud neck contractile ring (IDA)
YNL166C		Cytoplasm. Bud.	cellular bud neck (IDA)<br>cellular bud neck septin ring (IDA, IPI)
YNL188W		Cytoplasm › cytoskeleton › spindle pole body.	half bridge of spindle pole body (IDA)
YNL242W			cytoplasm (IDA)<br>extrinsic to membrane (IDA)<br>pre-autophagosomal structure (IDA)
YNL325C		Vacuole membrane; Peripheral membrane protein.	extrinsic to membrane (IDA)<br>fungal-type vacuole membrane (IDA)<br>PAS complex (IDA, IPI)
YOL091W		Prospore membrane. Cytoplasm › cytoskeleton › spindle pole.	spindle pole body (IDA)
YOR020C		Mitochondrion matrix.	mitochondrial matrix (IDA)<br>mitochondrion (IDA)
YOR034C		Membrane; Multi-pass membrane protein.	Golgi apparatus (IDA)
YOR096W		Cytoplasm. Nucleus › nucleolus	90S preribosome (IDA)<br>cytosolic small ribosomal subunit (NAS)<br>small-subunit processome (IDA)
YOR111W		Cytoplasm (Potential).	
YOR171C		Cell membrane; Peripheral membrane protein. Endoplasmic reticulum membrane; Peripheral membrane protein. Late endosome membrane; Peripheral membrane protein. Golgi apparatus membrane; Peripheral membrane protein	cortical endoplasmic reticulum (IDA)<br>endoplasmic reticulum (IDA)<br>Golgi apparatus (IDA)<br>membrane fraction (IDA)<br>plasma membrane (IDA)<br>soluble fraction (IDA)
YOR177C		Prospore membrane. Cytoplasm › cytoskeleton › spindle pole body. Cytoplasm › cytoskeleton › spindle pole.	spindle pole body (IDA)
YOR257W		Nucleus › nuclear pore complex. Cytoplasm › cytoskeleton › spindle pole body.	half bridge of spindle pole body (IDA)<br>nuclear pore (TAS)<br>colocalizes_with nucleotide-excision repair factor 2 complex (IPI)<br>transcription export complex 2 (IDA)
YOR301W		Cell membrane; Multi-pass membrane protein. Bud neck. Bud tip.	cellular bud neck (IDA)<br>integral to membrane (ISS)

YOR329C		Membrane; Peripheral membrane protein.	actin cortical patch (IDA)<br>nucleus (IMP)<br>peripheral to membrane of membrane fraction (IDA)
YPL002C		Cytoplasm. Endosome membrane; Peripheral membrane protein	ESCRT II complex (IDA)
YPL174C		Cytoplasm. Cytoplasm › cytoskeleton (By similarity). Cytoplasm › cytoskeleton › spindle pole	colocalizes_with astral microtubule (IDA)<br>colocalizes_with cell cortex (IDA)<br>dynactin complex (IDA)<br>colocalizes_with spindle pole body (IDA)
YPL176C		Cell membrane; Single-pass type II membrane protein (Potential).	fungal-type vacuole (IDA)
YPL187W			extracellular region (TAS)
YPR088C		Cytoplasm.	signal recognition particle, endoplasmic reticulum targeting (IDA)
YPR112C		Nucleus	90S preribosome (IDA)<br>nucleolus (IDA)
YDR277C			
YFR008W			
YHR057C			
YKL105C			
YLR031W			
YNR068C			
YNR069C			
YPL077C			
