# Supplementary material for: Predicting multiplex subcellular localization of proteins using protein-protein interaction network: a comparative study
Source: BMC Bioinformatics. 2012 Jun 25;13(Suppl 10):S20. doi: 10.1186/1471-2105-13-S10-S20 (PMC3314587; doi:10.1186/1471-2105-13-S10-S20)
Supplement: Additional file 7 — Comparison of the four basic classifiers and the ensemble classifier for 46 "ambiguous" proteins that was annotated with more than one subcellular localization sites. [file 1471-2105-13-S10-S20-S7.pdf]

Supplementary Table 7 Comparison of the four basic classifiers and the ensemble classifiers for 46 “ambiguous” proteins that was annotated with more than one subcellular localization sites.

ORF	GFP annotation	Majority	$\chi 2$ score	GenMultiCut	FunFlow	Ensemble
YAL029C	cell periphery;bud neck;cytoplasm;bud	bud neck;cytoplasm;nucleus	cell periphery;bud neck;bud	nucleus	cytoplasm	bud neck;cytoplasm;nucleus;bud
YBL105C	bud neck;cytoplasm;bud	ER;actin;cytoplasm	ER;actin;nucleus	cytoplasm		ER;actin;cytoplasm
YBR102C	cell periphery;bud neck;bud	cell periphery;cytoplasm;bud	cell periphery;bud neck;bud	cell periphery;bud neck;bud	cell periphery;bud neck;bud	cell periphery;bud neck;bud
YBR130C	cell periphery;cytoplasm;bud	cell periphery;cytoplasm;nucleus	cell periphery;bud neck;bud		cytoplasm	cell periphery;cytoplasm;bud
YBR200W	cell periphery;bud neck;bud	bud neck;cytoplasm;nucleus	cell periphery;bud neck;bud	cytoplasm		bud neck;cytoplasm;bud
YBR260C	bud neck;cytoplasm;bud	bud neck;cytoplasm	mitochondrion;bud neck;nucleus	cytoplasm		bud neck;cytoplasm

YCL024W	cell periphery;bud neck;bud	cell periphery;bud neck;cytoplasm	cell periphery;bud neck;nucleus	bud neck	cell periphery;bud neck;cytoplasm	cell periphery;bud neck;cytoplasm
YDL146W	cell periphery;bud neck;cytoplasm;bud	mitochondrion;actin;cytoplasm	cell periphery;bud neck;actin	actin;cytoplasm	actin	actin;cytoplasm
YDR164C	bud neck;cytoplasm;bud	vacuole;punctate composite;lipid particle	vacuole;endosome;lipid particle		cytoplasm	ipid particle
YDR166C	cell periphery;bud neck;bud	cell periphery;bud neck;bud	cell periphery;bud neck;bud	cell periphery;bud neck;bud	cell periphery;bud neck;cytoplasm;bud	cell periphery;bud neck;bud
YDR181C	cytoplasm;nucleus	cytoplasm;nucleus	mitochondrion;nucleolus;nucleus	cytoplasm;nucleus	nucleus	cytoplasm;nucleus
YDR309C	cytoplasm;bud	cell periphery;bud neck;actin	cell periphery;bud neck;actin		cell periphery;bud neck;cytoplasm	cell periphery;bud neck

YDR479C	punctate composite;cytoplasm	peroxisome	peroxisome;cytoplasm;nucleus	peroxisome	peroxisome;cytoplasm	peroxisome;cytoplasm
YDR507C	bud neck;cytoplasm;bud	cell periphery;bud neck;cytoplasm	cell periphery;bud neck;nucleus	bud neck	bud neck;cytoplasm	cell periphery;bud neck;cytoplasm
YER005W	Golgi;early Golgi	vacuolar membrane	vacuolar membrane;cytoplasm;nucleus	vacuolar membrane	vacuolar membrane;cytoplasm	vacuolar membrane;cytoplasm
YER149C	cell periphery;bud	cell periphery;bud neck;cytoplasm	vacuole;cell periphery;bud neck	cytoplasm;nucleus		cell periphery;bud neck;cytoplasm
YFR016C	cytoplasm;bud	cytoplasm	mitochondrion;cytoplasm;nucleus	cytoplasm		mitochondrion;cytoplasm
YGR041W	bud neck;bud	cell periphery;bud neck	cell periphery;bud neck;cytoplasm	cell periphery;bud neck;cytoplasm	cell periphery;bud neck;cytoplasm	cell periphery;bud neck;cytoplasm

YGR238C	cell periphery;bud	cytoplasm;nucleus	mitochondrion;nucleolus;cytoplasm	cytoplasm		mitochondrion;cytoplasm
YGR241C	cell periphery;bud neck;bud	punctate composite;late Golgi;actin	punctate composite;late Golgi;actin		cytoplasm	punctate composite;late Golgi;actin
YHR158C	cell periphery;bud neck;bud	punctate composite;cytoplasm;nucleus	mitochondrion;punctate composite;cytoplasm	cytoplasm	nucleus	mitochondrion;punctate composite;cytoplasm;nucleus
YHR161C	cell periphery;bud neck;bud	punctate composite;late Golgi;actin	punctate composite;late Golgi;actin		cytoplasm	punctate composite;late Golgi;actin
YIL053W	cytoplasm;nucleus	cytoplasm;nucleus	mitochondrion;nucleolus;cytoplasm	cytoplasm;nucleus		mitochondrion;cytoplasm;nucleus

YIL068C	cell periphery;bud neck;bud	cell periphery;bud neck;bud	cell periphery;bud neck;bud	cell periphery;bud neck;bud	cell periphery;bud neck;cytoplasm;bud	cell periphery;bud neck;bud
YIL140W	vacuole;cell periphery;bud neck;bud		mitochondrion;cytoplasm;nucleus	cytoplasm		mitochondrion;cytoplasm
YJR104C	cytoplasm;nucleus	cell periphery;cytoplasm;nucleus	cell periphery;bud neck;bud	cytoplasm;nucleus	nucleus	cell periphery;cytoplasm;nucleus;bud
YKL079W	cytoplasm;bud	punctate composite;bud neck;cytoplasm	punctate composite;bud neck;nucleus	cytoplasm	cytoplasm	punctate composite;bud neck;cytoplasm
YLR044C	cytoplasm;nucleus	cytoplasm;nucleus	mitochondrion;cytoplasm;nucleus	cytoplasm;nucleus	nucleus	mitochondrion;cytoplasm;nucleus
YLR166C	cell periphery;bud neck;bud	cell periphery;bud neck;bud	cell periphery;bud neck;bud	cell periphery;bud neck;bud	cell periphery;bud neck;cytoplasm;bud	cell periphery;bud neck;bud

YLR187W	cell periphery;bud neck;cytoplasm;bud	mitochondrion;cytoplasm;nucleus	mitochondrion;cytoplasm;nucleus	cytoplasm		mitochondrion;cytoplasm;nucleus
YLR313C	bud neck;bud	cytoplasm	mitochondrion;cytoplasm;nucleus	cytoplasm		mitochondrion;cytoplasm
YLR353W	cell periphery;bud neck;bud	cell periphery;bud neck;cytoplasm	cell periphery;bud neck;nucleus	cell periphery;bud neck	cell periphery;bud neck;cytoplasm	cell periphery;bud neck;cytoplasm
YMR029C	ER;cytoplasm		mitochondrion;cytoplasm;nucleus		cytoplasm	mitochondrion;cytoplasm
YMR124W	cell periphery;bud neck;cytoplasm;bud	nucleus	mitochondrion;nucleolus;cytoplasm	nucleus	cytoplasm;nucleus	mitochondrion;cytoplasm;nucleus
YMR163C	punctate composite;bud	cytoplasm	mitochondrion;nucleolus;nucleus	cytoplasm		mitochondrion;cytoplasm
YMR192W	bud neck;cytoplasm;bud	mitochondrion;actin;cytoplasm	cell periphery;bud neck;actin	cytoplasm	actin	mitochondrion;actin;cytoplasm

YNL166C	bud neck;cytoplasm;bud	cell periphery;bud neck	cell periphery;bud neck;cytoplasm	cell periphery;bud neck	cell periphery;bud neck;cytoplasm	cell periphery;bud neck;cytoplasm
YNL272C	bud neck;cytoplasm;bud	bud neck;cytoplasm;nucleus	mitochondrion;punctate composite;bud neck	cytoplasm		mitochondrion;bud neck;cytoplasm
YNL298W	cell periphery;cytoplasm;bud	cell periphery;cytoplasm;nucleus	cell periphery;bud neck;cytoplasm	cytoplasm		cell periphery;cytoplasm
YNR049C	bud neck;cytoplasm;bud	punctate composite;endosome;lipid particle	vacuole;endosome;lipid particle		cytoplasm	endosome;lipid particle
YOL112W	cell periphery;bud neck;bud		mitochondrion;cytoplasm;nucleus	cytoplasm;nucleus		mitochondrion;cytoplasm;nucleus

YOR326W	cell periphery;bud neck;cytoplasm;bud	cell periphery;cytoplasm;nucleus	cell periphery;bud neck;bud			cell periphery;bud
YPL032C	cell periphery;bud neck;cytoplasm;bud	actin	actin;cytoplasm;nucleus	actin	actin;cytoplasm	actin;cytoplasm
YPL204W	bud neck;cytoplasm;nucleus;bud	punctate composite;cytoplasm;nucleus	punctate composite;cytoplasm;nucleus	cytoplasm		punctate composite;cytoplasm;nucleus
YPL249C	cell periphery;bud neck;cytoplasm;bud	actin;cytoplasm;nucleus	mitochondrion;actin;nucleolus	actin;cytoplasm	actin	mitochondrion;actin;cytoplasm
YPR055W	cell periphery;bud neck;cytoplasm;bud	cell periphery;bud neck;bud	cell periphery;bud neck;bud	cell periphery;bud neck;bud	cell periphery;bud neck;cytoplasm;bud	cell periphery;bud neck;bud
